# An Iron‐Catalyzed Sustainable Functional Group Transfer Strategy for In‐Water Transformation of Organic Sulfides to Sulfoxides by a Hydroxylamine‐Derived Oxidant

**DOI:** 10.1002/cssc.202500032

**Published:** 2025-04-11

**Authors:** Arya Singh, Yashdeep Maurya, Akhilesh Sharma, Swetha Vasanthdamodar Sivapreetha, Mehar Ul Nisa, Vishal Kumar, Puneet Gupta, Kartikay Tyagi, Sayanti Chatterjee

**Affiliations:** ^1^ Department of Chemistry Indian Institute of Technology Roorkee Roorkee Uttarakhand 247667 India; ^2^ Department of Inorganic Spectrosopy Max Planck Institute for Chemical Energy Conversion Stiftstrasse 34‐36 45470 Mülheim an der Ruhr Germany

**Keywords:** biocompatible, functional group transfer, iron, organic sulfides, sulfilimine, sulfoxides, sustainable catalysis, water

## Abstract

Herein, an unprecedented strategy for unlocking a new and efficient ‐*NH* to ‐*O* functional group transfer protocol to synthesize a variety of complex organic sulfoxides chemoselectively, starting from organic sulfides, in water is presented. This new functional group transfer protocol is based on harnessing the potential of metalloradical‐assisted intermolecular functional group transposition or ‘*InterGroupXfer’* to replace highly sensitive and reactive high‐valent metal intermediates, [M = X] intermediates (X = O, NH). This sustainable functional group transfer strategy employs Earth‐abundant iron catalyst and bench‐stable and convenient‐to‐handle hydroxyl amine‐derived surrogate, operates under mild conditions in water or even under solvent‐free conditions, exhibits broad functional group tolerance, as well as versatility of reaction scale and proceeds without the use of any precious metal catalyst or additional oxidant. A comprehensive electronic and mechanistic investigation, supported by computaional calculations, has been conducted to elucidate the reaction mechanism. The utility of the developed methodology as well as studies of biological activity foster future pathways for exploring uncharted chemical space. The reported work with exceptional synthetic flexibility and operational simplicity aligns well with the prospect of green and sustainable chemistry and is expected to unlock new concepts in the emerging research area of catalytic functional group transfer reactivity.

## Graphical abstract

An iron‐catalyzed ecofriendly reaction protocol is demonstrated to chemoselectively convert readily available organic sulfides to valuable sulfoxides, under mild reaction conditions in water. The functional group tolerance, scalability, reaction compatibility, and potential application make the protocol highly attractive in terms of green and sustainable chemistry.

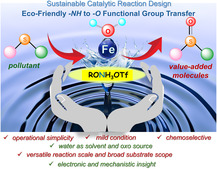

## 1 Introduction

Nature‐inspired chemistry has served as a promising approach for chemists toward the discovery of new catalytic strategies and methodologies for production of vital fuels and fine chemicals. Though recent years have seen significant progress in the state‐of‐the‐art developments in sustainable catalysis,^[^ ^1^ ^]^ most of the chemical industries and processes rely on the use of noble metal catalysts as well as the use of hazardous/alarming reagents or solvents and possess a serious threat to the natural reserves and healthy environment.^[^ ^2^ ^]^ To address these challenges, one of the foci of our research group^[^ ^3^ ^]^ is to explore opportunities for discovery of new reactions that utilize Earth‐abundant elements as catalysts, reduce waste, energy consumption, and the use of toxic solvents and reagents, thereby lowering the environmental impact of chemical synthesis.

**Scheme 1:**
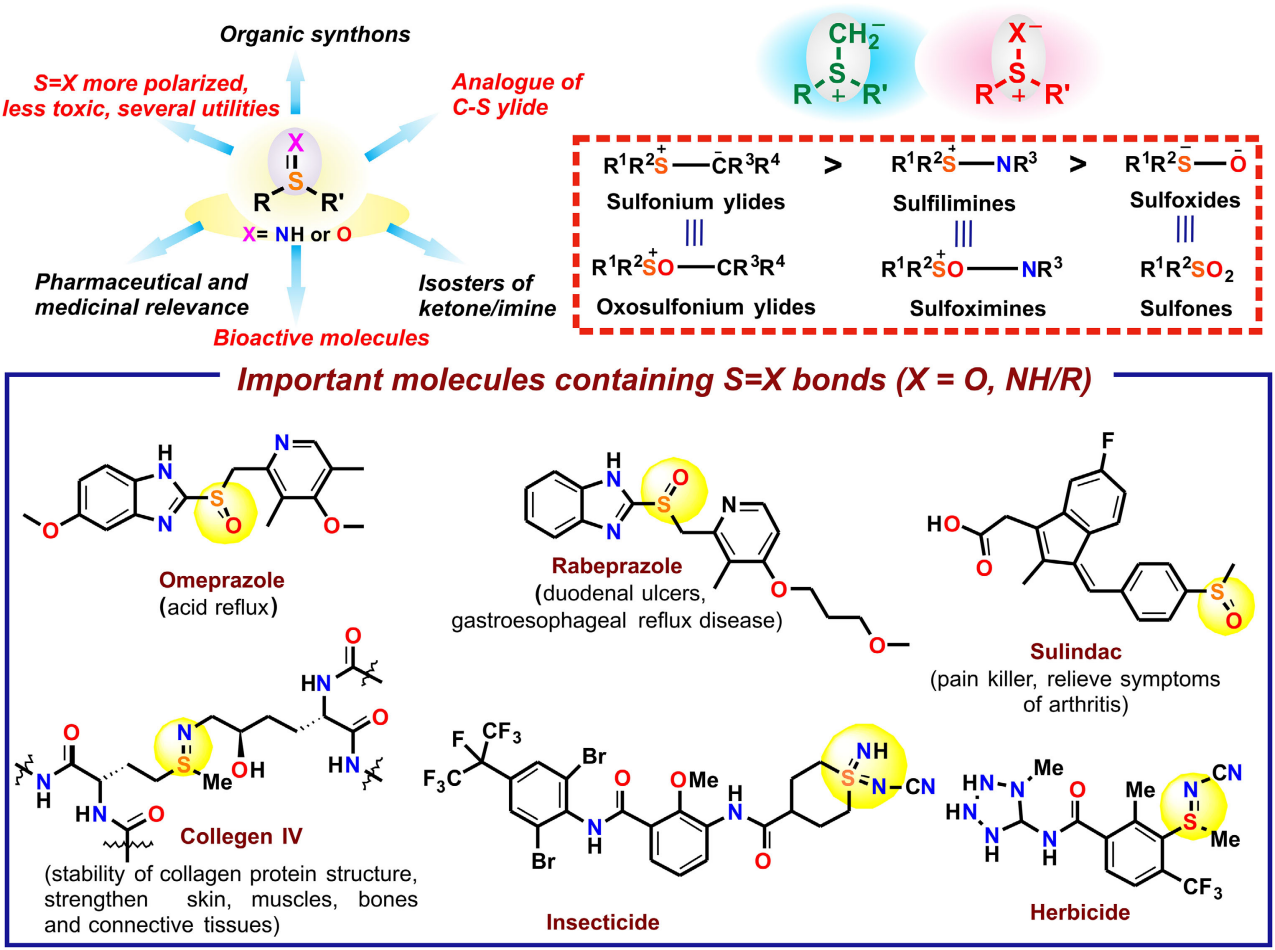
Importance of S=X (X = O, NH)‐containing molecules in natural and chemical realm.

In our search for developing sustainable catalytic solutions for converting waste to value‐added products, the abundant organic sulfides have piqued our interest. Wastewater and seawater have long been considered as potential sources to produce freshwater after proper treatment.^[^ ^4^ ^]^ Among sulfur‐containing molecules which have significant alarming impact on the environment, the organic sulfides, particularly the volatile ones, are potential impurities found in wastewater. Besides, organic sulfides have significant health hazard effects and need remedial strategies.^[^ ^5^, ^6^, ^7^ ^]^ One potential way of removing organic sulfide impurities (R—S—R′) would be their catalytic functionalization (—S=X, X = O or NH group, oxygenation, or imination), involving an overall oxidation on the S atom. The —S=X (X = O or NH) moieties being more polarized have several utilities that can lower the overall lipophilicity and improve the aqueous solubility and metabolic stability of a molecule, which can lead to an improvement of absorption, distribution, metabolism, and excretion properties (**Scheme** **1**).^[^ ^8^ ^]^ Molecules containing S—O and S—N bonds are of considerable significance in synthetic and industrial chemistry, as well as highly relevant for medicinal chemistry and crop protection because they exhibit interesting bioactivities (Scheme 1).^[^ ^9^, ^10^, ^11^, ^12^, ^13^, ^14^, ^15^, ^16^, ^17^ ^]^ The —S=X (X = O or NH) molecules have the potential to be employed as ketone/imine bond isosteres. They often find use as building blocks for chiral ligands^[^ ^18^ ^]^ and as potential pseudopeptides.^[^ ^19^ ^]^ Organic sulfoxides or sulfilimines having —S=X (X = O, NH) can be compared to analogous S—C ylides which are shown to be crucial agents in synthesis, with an overall positive charge density on sulfur and negative charge on the heteroatom (X) (Scheme 1).^[^ ^20^ ^]^ Recent years have seen a rise in interest in creating a comparable sulfur–heteroatom (sulfur–oxygen/sulfur–nitrogen ylide, sulfoxide/sulfilimine respectively) molecules. Based on the relative electronegativities of the atoms involved, sulfilimines (S—N moieties) should be intermediate between those of sulfonium ylides (S—C) and of sulfoxides (S—O). Sulfilimines should also be similar in properties to the corresponding sulfoximines but more reactive, just as the sulfoxides are more reactive than sulfones and sulfonium ylides are more reactive than the corresponding oxosulfonium ylides (Scheme 1).^[^ ^21^ ^]^

As far as —S=X molecule syntheses are concerned (X = O, sulfoxides and X=NH sulfilimine), the traditional approaches particularly for the sulfoxides involve either nonoxidative route using sulfonyl chloride‐based reagents or environmentally nonbenign anhydrous aluminum chloride catalyst.^[^ ^22^ ^]^ An oxidative approach for sulfoxide/sulfilimine synthesis uses corresponding sulfides and hazardous oxo or nitrogen sources either chemically or electrochemically.^[^ ^23^, ^24^, ^25^, ^26^, ^27^, ^28^ ^]^ Unfortunately, most of the reported methods are not satisfactory from an economical and environmental standpoint; thus, greener processes for catalytic synthesis of sulfilimines/sulfoxides with a broad substrate scope, particularly using benign nitrogen source or water as the source of oxygen atom respectively would offer a promising alternative. For the synthesis of —S=X where X = NH, the direct synthesis process of free sulfilimine is still at its infancy,^[^ ^29^, ^30^ ^]^ likely due to the sensitivity of —S=NH fragment. *N*‐protected sulfilimine synthesis process mostly uses hazardous nitrogen sources, along with heavy metal congeners to generate the putative metal‐based *N*‐transfer intermediates and are limited in catalytic efficiency, selectivity, and substrate scope.^[^ ^15^, ^31^, ^32^ ^]^

A simple procedure to synthesize organic sulfoxide/sulfilimine (S=X moiety, where X = O or NH) is further challenged, because of the over‐oxidation products like sulfone/sulfoximine or several other sulfur species (sulfinic ester, sulfenamide, sulfonic acids, sulfinamide, or sulfonamide) as side products under the known reaction conditions.^[^ ^15^, ^33^ ^]^

**Scheme 2:**
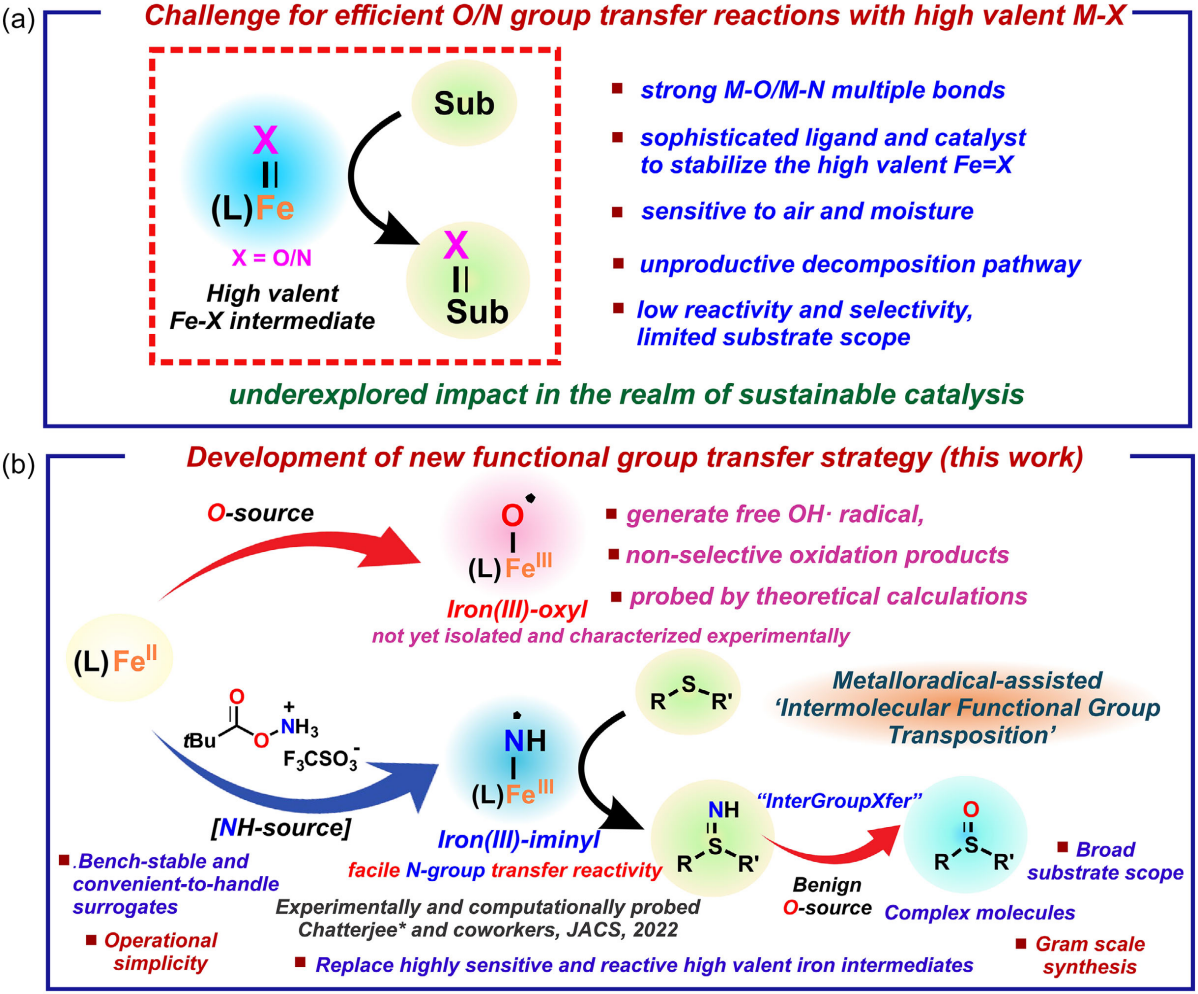
a) Challenge in group transfer reactions by putative high‐valent metal intermediates. b) Context of the present work.

In nature there are several heme and nonheme‐based metalloenzymes which oxygenates organic sulfide to the corresponding sulfoxide forming S=O bond using dioxygen as the terminal electron acceptor following either an electron transfer or an oxygen atom transfer mechanism by putative high‐valent metal‐oxo intermediate with very high selectivity (**Scheme** **2**).^[^ ^34^ ^]^ Catalytic reactions by natural enzymes occur in aqueous medium under ambient conditions.^[^ ^35^ ^]^ Synthetic chemists have designed several bioinspired catalysts based on first row transition metals^[^ ^36^, ^37^ ^]^ or their heavier congeners to emulate sulfide oxidation using suitable “oxo” source as well as corresponding sulfide imination which is a *new to nature* reaction, using potential NH sources.^[^ ^32^, ^38^, ^39^, ^40^, ^41^, ^42^ ^]^ Such oxidation/imination reactions by the synthetic community have been proposed to be invoked by putative high‐valent metal oxo/imino intermediates using sophisticated ligand and catalyst design, mostly under nonaqueous organic solvent medium and the reactivity and selectivity is often limited due to the instability of the proposed metal‐oxo/metal–nitrogen intermediates (Scheme 2). Even if stabilized, many of the reported metal–oxygen or metal−nitrogen intermediates lack *O* or *N*‐group transfer reactivity due to the strong metal–oxygen/metal–nitrogen multiple bonds, thereby decreasing the propensity for *O‐* or *N*‐group transfer activity (Scheme 2). Thus oxo/nitrogen group transfer reactions with a versatile and broad substrate scope under ambient conditions remains a challenge, particularly in aqueous medium or under solvent‐free conditions, and imposes a barrier for these reactions to create an impact in the realm of sustainable catalysis.^[^ ^43^ ^]^ The development of functional group transfer strategies employing bench‐stable and convenient‐to‐handle surrogates to replace highly sensitive and reactive intermediates is expected to unlock novel synthetic pathways with the prospect of green and sustainable chemistry.

We have recently reported a very intense spectroscopic and computational study of an Fe^III^‐iminyl radical species derived from hydroxlamine‐derived reagent as the nitrogen source for aminofunctionalization of alkenes^[^ ^42^ ^]^ as well as other hydrocarbons and heteroatom.^[^ ^44^, ^45^, ^46^, ^47^, ^48^, ^49^ ^]^ The interesting electronic structure of the putative iron‐iminyl radical intermediate, having an elongated Fe—N bond, facilitated the efficient *N*‐group transfer activity, circumventing the need to generate high‐valent iron intermediates for group transfer reactivity and highlighting the potential of utilizing metal coordinated nitrogen‐centered radicals^[^ ^50^, ^51^ ^]^ as a standard strategy in chemical synthesis and catalysis (Scheme 2).^[^ ^38^, ^42^ ^]^ In this work, we envision to harness the reactivity of the easily accessible Fe^III^‐iminyl radical intermediate for developing a metalloradical‐assisted “intermolecular functional group transposition” or InterGroupXfer through a well‐designed sustainable reaction medium for discovering new and elusive reactions (Scheme 2).

Herein, we present an unprecedented strategy for unlocking a new and efficient –*NH* group transfer followed by its transposition to –*O* functional group, for the synthesis of a variety of organic sulfoxides chemoselectively, starting from organic sulfides, using water as the source of oxygen atom and solvent (**Table** **1** and Scheme 2b). The functional group transfer strategy employs Earth‐abundant iron catalyst and bench‐stable and convenient‐to‐handle hydroxylamine‐derived triflic acid ammonium salt PivONH_3_OTf (*O*‐pivaloyl hydroxylamine triflic acid), (Table 1), which operates under mild conditions in water or under solvent‐free condition, exhibits broad functional group tolerance, is scalable, and proceeds without the use of any precious metal catalyst or additional oxidant. The concept for the development of this new functional group transfer protocol is based on harnessing the potential of metalloradical‐assisted intermolecular functional group transposition or InterGroupXfer to replace highly sensitive and reactive high‐valent iron intermediates, Fe(IV)/Fe(V) = X intermediates (X = O, NH) (Scheme 2).

**Table 1:** Selected examples for optimization.

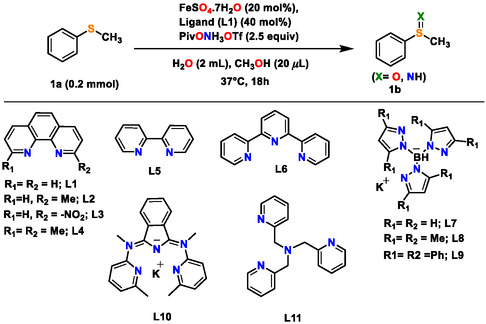				
Entrya)	Deviation from standard conditions	Product **1b** b)	Relative ratio of S=X (X = O, NH)a), c)	
			S=O	S=NH
1	No deviation from standard condition	98%	98%	trace
2	Fe(acac)_2_, no L1	59%	58%	trace
3	FeCl_2_, no L1	50%	49%	trace
4	FeSO_4_.7H_2_O, no L1	70%	68%	trace
5	FePc, no L1	95%	94%	trace
6	L5 instead of L1	98%	96%	trace
7	L6 instead of L1	97%	95%	trace
8	L7 instead of L1	55%	17%	39%
9	L10 instead of L1	75%	37%	38%
10	L11 instead of L1	90%	70%	20%
11	Fe(acac)_3_, no L1	20%	19%	trace
12	H_2_O	95%	93%	trace
13	CH_2_Cl_2_	26%	7%	19%
14	1 equiv of PivONH_3_OTf	20%	19%	trace
15	No catalyst	22%	13%	7%
16	Under Argon	95%	93%	trace

a) See SI for further information.

b) ^1^H NMR yield.

c) HRMS and GC‐MS

## 2 Results and Discussion

### 2.1 Reaction Optimization

We initiated our investigation using methyl phenyl sulfide (1a) as a model substrate along with PivONH_3_OTf (Ox_1_) as the aminating reagent and oxidant to obtain the corresponding oxidized form of sulfides, having —S=X moiety (When X = O, sulfoxide, when X = NH, sulfilimine) (Table 1). Testing various first row transition metals (see Supporting Information) revealed simple iron(II) salts as promising candidates to catalyze this reaction (Table 1 and also Table S1, Scheme S4, Supporting Information). Fe(acac)_2_ provided the desired product methyl phenyl sulfoxide/sulfilimine (—S=X) in 59% yield (Entry 2, Table 1). Interestingly, Fe^II^Pc (Iron(II) Phthalocyanine) increased the yield of product formation (95%, Table 1, entry 5), which prompted us to further investigate the ligand effect on the iron‐catalyzed reaction (Table 1). Simple 1,10 phenanthroline monohydrate (L1) in combination with FeSO_4_.7H_2_O in a 2 : 1 ligand : metal catalyst ratio with an optimum catalyst loading of 20 mol % and 2.5 equiv. of PivONH_3_OTf (Ox_1_) resulted in almost quantitative (98%) conversion of methyl phenyl sulfide (**1a**) to methyl phenyl sulfoxide/sulfilimine (—S=X, X = O, sulfoxide, S=NH, sulfilimine) (Table 1, entry 1). Other bidentate as well as tridentate and tetradentate ligands were screened but turned out to be less efficient over a wide substrate scope range in some cases (Table 1, entry 6–10 and also Scheme S7, Supporting Information, Table S3, Supporting Information). Iron(III) salts showed reduced activity compared to iron(II) salts (Table 1, entry 11). Moving to other first row transition metals like Mn, Co, Ni, or Cu‐based salts gave reduced yield (Table S1, Scheme S4, Supporting Information). Various other iron (II) catalysts in combination with suitable ligand were found to catalyze the sulfide oxidation/imination reaction efficiently (Table 1 and its Scheme, also Scheme S7, Table S3, Supporting Information), and in absence of any metal catalyst there was a significant decrease in reaction yield implicating the key role of iron in the process (Table 1, entry 15).

**Figure 1:**
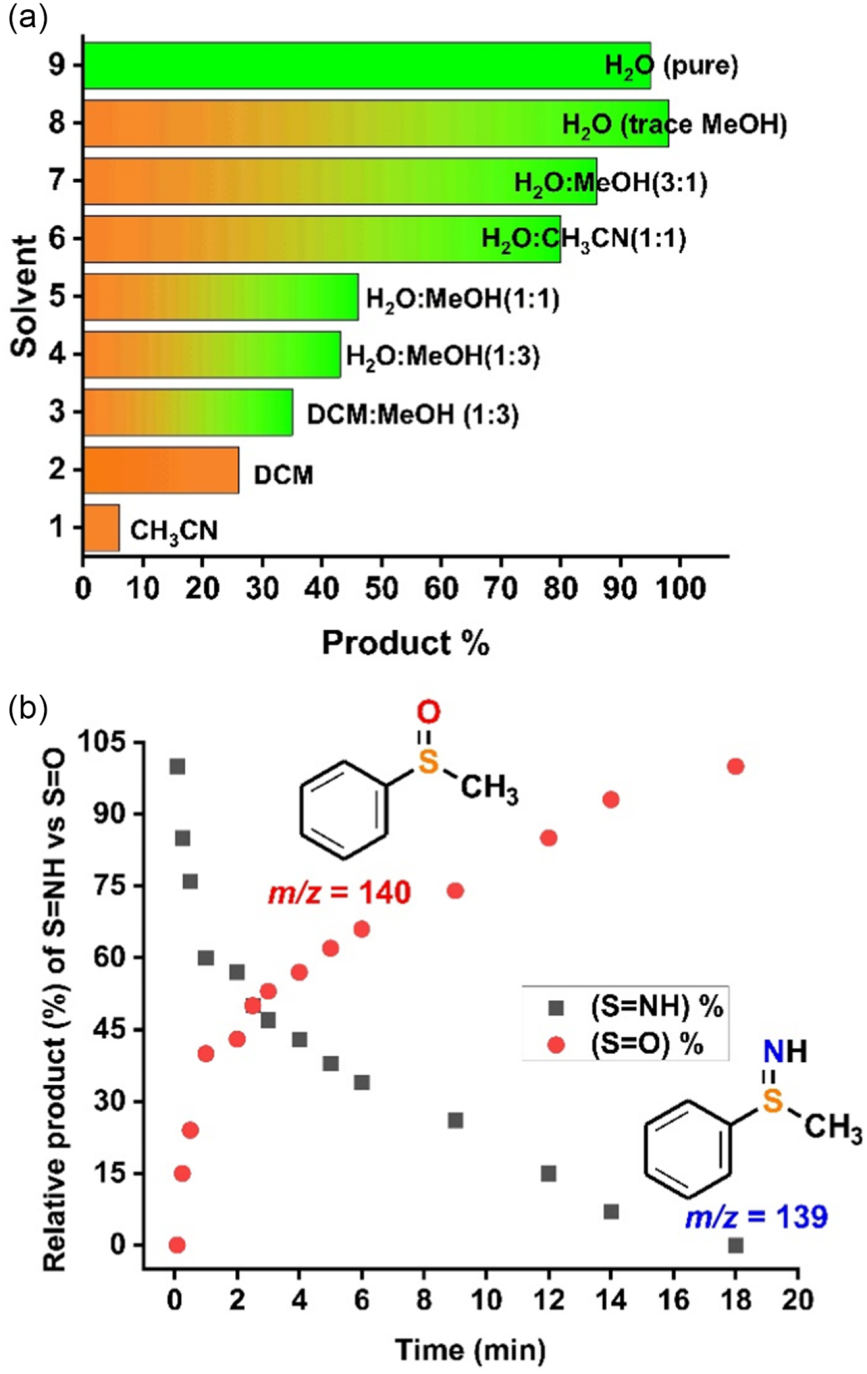
a) Solvent screening for the iron‐catalyzed sulfide oxidation reaction using PivONH_3_OTf (Ox_1_). b) Time trace plot for —S=NH versus —S=O product formation during the reaction of methyl phenyl sulfide with iron catalyst and PivONH_3_OTf under standard reaction condition in water.

Screening of different solvents revealed that the reaction operates most efficiently in water as the solvent and in fact a solvent mixture of water with trace amount of methanol gave the best yield (Table 1, entry 1, **Figure** **1**a, and also Table S8, Figure S5, Supporting Information) Interestingly in dry organic solvents like CH_3_CN, CH_2_Cl_2_ the yield of the reaction reduced dramatically (see SI). Increasing the equivalence of aminating agent led to higher conversion of methyl phenyl sulfide (1a) and with 2.5 equiv. of the reagent (Ox_1_), maximum methyl phenyl sulfoxide/sulfilimine (—S=X, X = O, sulfoxide, S = NH, sulfilimine) formation was observed (Figure S3, Table S5, Supporting Information).

Remarkably, under the optimized reaction conditions, no other byproduct or overoxidized sulfone or disulfilimine products were detected and methyl phenyl sulfide was chemoselectively converted to its corresponding mono —S=X product (methyl phenyl sulfoxide/sulfilimine (—S=X, X = O, sulfoxide, S = NH, sulfilimine). The reaction can be run open to air, in aqueous medium, without the need for any pretreatment or precaution. To optimize the time of reaction, we monitored the reaction progress at different time intervals. Analyses of the reaction profile by High Resolution Mass Spectroscopy (HR‐MS) and Gas Chromatography Mass Spectrometry (GC‐MS) revealed that initially methyl phenyl sulfide (1a) is converted to the corresponding sulfilimine as evident from the GC‐MS peak at *m/z* = 139 (Figure 1b. also Figure S23, Supporting Information). However, with increase in the reaction time, there is a concomitant decrease in sulfilimine product formation peak at *m/z* = 139 and a gradual increase of the corresponding sulfoxide (*m/z* = 140) (Figure 1b. also Figure S24, Supporting Information). At the end of 18 h, there was almost quantitative conversion of methyl phenyl sulfide to the corresponding methyl phenyl sulfoxide selectively (Figure 1b, Table 1, Table S9, Supporting Information).

### 2.2 Substrate Scope

**Scheme 3:**
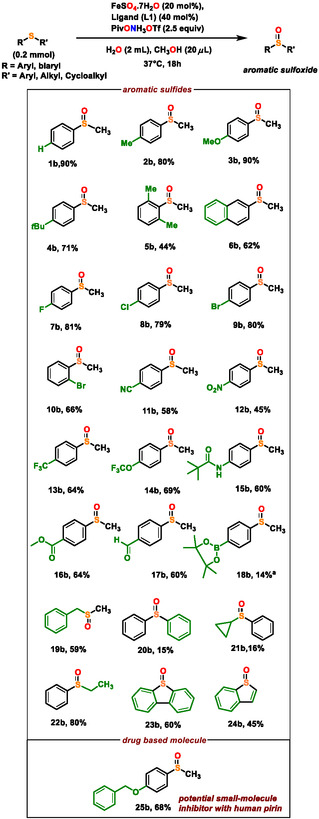
Scope of sulfoxidation for aryl/benzyl alkyl sulfides. Yields are of isolated products. Note: ^a^denotes crude yield.

**Scheme 4:**
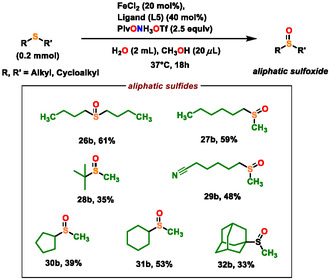
Scope of sulfoxidation for alkyl/cycloalkyl sulfides. Yields are of isolated products.

**Scheme 5:**
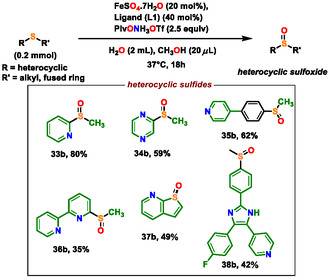
Scope of sulfoxidation for heterocylic sulfides. Yields are of isolated products.

With the optimized conditions in hand, we next explored the substrate scope. Starting from both aromatic and aliphatic sulfides as well as heterocyclic sulfides, a wide range of starting materials were selectively converted to their corresponding sulfoxides in high yields (**Scheme** **3**, **4** and **5**).

Organic sulfides with different substitution patterns having a combination of aromatic and methyl substituents (R—S—R′ where R = aromatic, R′ = methyl) (**1a‐19a,**) were efficiently transformed, regardless of steric bulk or substitution at the *ortho*‐position, to their corresponding methyl aryl sulfoxides (**1b**‐**19b**) (Scheme 3).

Considering electronic effects, aromatic methyl sulfides with halogen substituents (**7a‐10a**) and other electron‐ poor substrates with varied functional groups such as pseudohalides –CN, –NO_2_, –CF_3_, –OCF_3_ (**11a‐14a**) were tolerated, as well as electron‐rich arenes (**2a‐6a**) gave excellent yield. Electron‐rich substrates generally gave slightly higher yields when compared to electron‐poor substrates, a result consistent with the electrophilic nature of the postulated reactive iron intermediate *vide infra*. The reaction scope could also be successfully extended to biarylic system (**6b**), as well as other alkyl (R′) counterparts instead of methyl (**20b‐24b**). The reaction was further tolerant to sensitive functional groups like aromatic amide functionality (**15b**), ester (**16b**), aldehyde (**17b**), or boronate ester groups (**18b**). Interestingly, benzylic methyl sulfide was also transformed in excellent yield (**19b**) (Scheme 3). Thus, activated benzylic C—H bond remains unaffected under the mild conditions of this selective oxo transfer to sulfides.

The reaction was also harnessed for synthesis of fused benzothiophene molecules (**23b‐24b**) (Scheme 3). (4‐(Benzyloxy)‐phenyl) methyl sulfane (**25b**) is converted to drug‐based molecule (4‐(Benzyloxy)‐phenyl) methyl sulfoxide (Scheme 3) that acts as a potential small‐molecule inhibitor with human pirin, a nuclear protein of unknown function, thereby providing insight for understanding of the function of pirin^[^ ^52^ ^]^ (Scheme 3).

With further regard to aliphatic sulfides, we slightly modified the optimization condition (Scheme 4, also see section XIX′ of SI) and a variety of unactivated, aliphatic primary (**26a‐27a, 29a**), secondary (**30a‐31a**), and tertiary sulfides (**28a, 32a**) could be transformed in good yields, showing the efficiency of this protocol and tolerance toward various functional groups such as nitriles (**29b**) (Scheme 4).

Encouraged by these results, we evaluated more complex and biologically relevant substrates with our methodology. The broad scope of the reaction was demonstrated by the successful application of this synthetic procedure to a wide range of heterocyclic molecules (**33a‐38a**) in good‐to‐moderate yield.

Sulfoxides‐bearing pyridine moieties have evolved as first‐line treatment of peptic ulcers and gastroesophageal reflux disease.^[^ ^53^ ^]^ Adezmapomid could be synthesized in one step from the corresponding sulfide. These pyridylimidazole‐type sulfoxide can act as kinase inhibitors, and the sulfoxide derivative was identified as a potent inhibitor of stress‐activated p38 mitogen‐activated protein kinase.^[^ ^54^, ^55^ ^]^ Further studies showed that these sulfoxide compounds can also inhibit COX‐1, COX‐2, and thromboxane synthase.^[^ ^55^ ^]^ Adezmapomid has the ability to efficiently decrease platelet aggregation and is a potential drug candidate for the prevention of postsurgery adhesions and is currently under clinical trials.^[^ ^53^ ^]^

### 2.3 Chemoselectivity and Biocompatibility Studies: Versatility of Reaction Scale and Reaction Medium

**Scheme 6:**
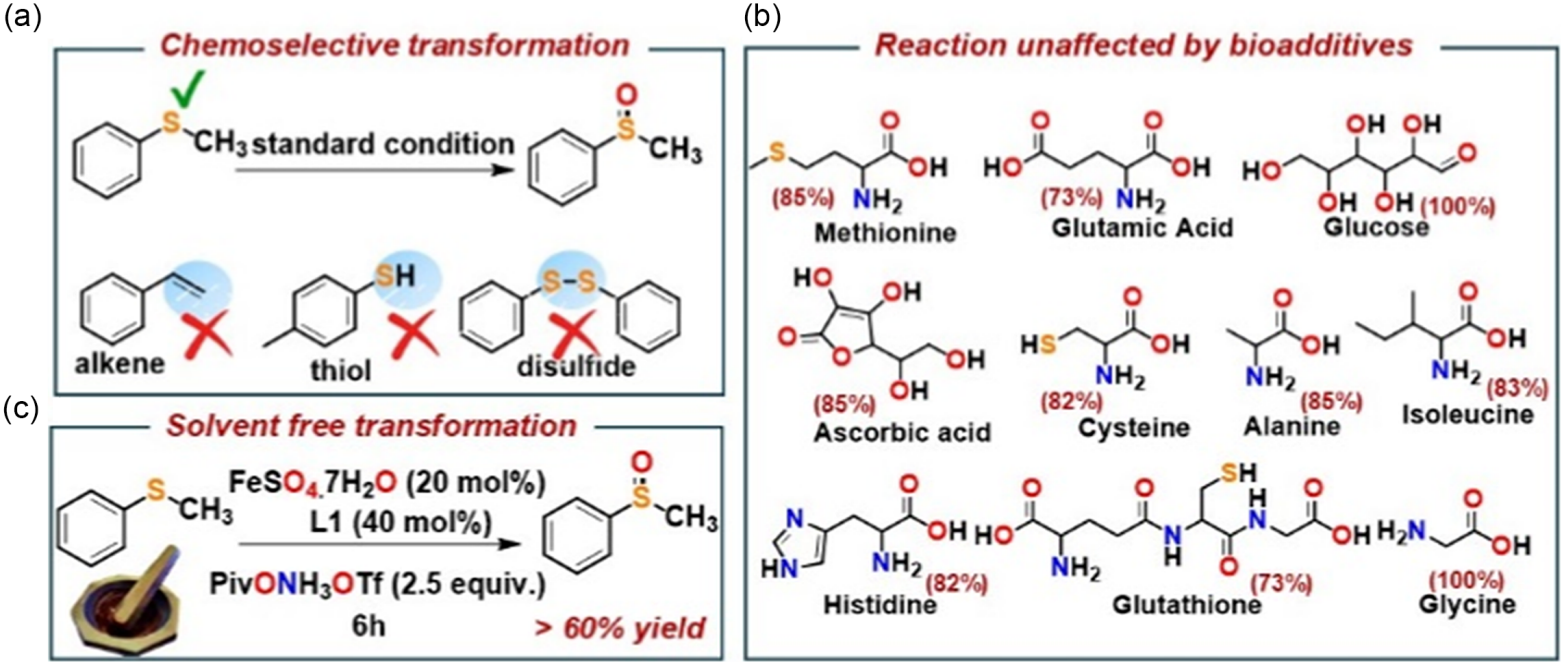
a) Chemoselectivity of the developed methodology. b) Stability of the developed reaction toward biomimetic additives. c) Extension of developed methodology to solvent‐free condition. (1 equiv. of H_2_O was added as a reagent for best yield).

Further to explore the chemoselectivity of the optimized reaction, intermolecular competition experiments in the presence of various other functional groups (alkenes, thiols, disulfides) were performed (**Scheme** **6**a, also see section XVI in SI). Previous reports have shown that under organic solvent, alkenes or thiols underwent iron‐catalyzed aminofunctionalization reactions in the presence of the hydroxyl amine‐derived triflic acid salt (Ox1).^[^ ^45^, ^46^, ^47^ ^]^ However, under the standard reaction condition proposed in this study (Table 1, entry 1), a competitive reaction of organic sulfide (1a) in the presence of either styrene, or aromatic thiol, or disulfide, underwent chemoselective conversion of organic sulfide to the corresponding sulfoxide (Scheme 6a, Scheme S33–35, Supporting Information).

We also successfully performed Gram‐scale experiments to test the robustness and applicability of this synthetic procedure for industrial and pharmaceutical applications (Scheme S37, Supporting Information).

Further to efficiently apply the catalytic system in biological settings, for intracellular applications, a very low concentration range study (micromolar range) of the catalytic system also proved to be compatible (Scheme S36, Supporting Information). The reaction operates well in a wide range of aqueous buffer medium at ambient conditions (Figure S9, Supporting Information) and under O_2_ atmosphere. The yield of sulfoxide remains unaffected under biomimetic conditions (in the presence of salts and biomolecules, Scheme 6b, and also Figure S10, Supporting Information) maintaining all fitness factors required for assessing the catalytic reaction protocol as bio‐orthogonal chemical probes.^[^ ^56^ ^]^

Knowing about the importance of enantioenriched sulfoxides, we tested chiral ligand on our model substrate methyl phenyl sulfide. Unfortunately, moderate yields and no enantiomeric excess were obtained with the chiral ligand (**L12**) used in this study (Table S3 and also section XXIV, Supporting Information). However, this lack of stereo control in the transformation of aromatic sulfides can be addressed by combining our methodology with a biocatalytic racemate resolution^[^ ^57^, ^58^ ^]^ or by kinetic resolution.^[^ ^59^, ^60^ ^]^

### 2.4 Solvent‐Free Condition: Mechanochemical Approach

Over the past few decades, substantial advancements have been achieved in the field of “solvent‐free” solid–solid chemical reactions. Mechanochemical techniques, such as grinding or ball milling, have emerged as particularly effective methodologies, often surpassing the efficiency and sustainability of conventional solvent‐based processes.^[^ ^61^, ^62^, ^63^ ^]^ Despite the growing interest and success in this area, examples of oxidation reactions performed under solvent‐free conditions remain relatively limited,^[^ ^64^ ^]^ highlighting an area with significant potential for further exploration and innovation. We have extended our reaction compatibility and further optimized reaction for iron‐catalyzed transformation of organic sulfides to organic sulfoxide selectively using hydroxyl acid‐derived reagent PivONH_3_OTf (Ox_1_) under solvent‐free condition, using mechanochemical grinding in a mortar pestle (Scheme 6c also Section XXI, Supporting Information). The reaction yielded around 60% product over a period of 6 h, absorbing moisture from the environment. However, adding 1 equivalent of water as a reactant enhanced the yield of corresponding sulfoxide product significantly (Scheme 6c also Scheme S46, Supporting Information). To the best of our knowledge, this is the first report of a mechanochemical functional group transformation to organic sulfides for the catalytic synthesis of sulfoxides, utilizing water as the oxygen atom source. This breakthrough marks a promising advancement in the pursuit of sustainable catalytic research.

### 2.5 Application of the Developed Methodology

**Scheme 7:**
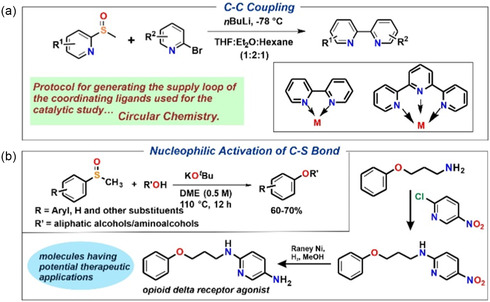
Potential application of the products derived from the developed methodology for a) C—C crosscoupling reactions. b) In crosscoupling of aryl methyl sulfoxides and alcohols via nucleophilic activation of C—S bond for synthesis of molecules having potential therapeutic applications.

The utility of the developed methodology was employed for various C—C bond forming reactions. In fact, the isolated heterocyclic sulfoxides (**33b** and **36b**) were used to synthesize important ligands used in coordination chemistry and catalysis. Starting from pyridine‐2‐sulfoxide (**33b**) and bipyridine‐2‐sulfoxide (**36b**), bipyridine ligand (**L5**) and terpyridine ligand (**L6**) respectively were isolated in good yield (**Scheme** **7**a and S51–S53, Supporting Information), showing the application of the developed methodology and also aligning the concept of circular chemistry to generate the ligands used for the catalytic study proposed in this work.

Additionally, we extended the scope of the developed methodology for crosscoupling of aryl methyl sulfoxides and alcohols via nucleophilic C—S bond activation^[^ ^12^ ^]^ and further functionalization to synthesize molecules having potential therapeutic applications. (Scheme 7b and Scheme S54–S58, Supporting Information).

### 2.6 Biological Implications of the Synthesized Sulfoxides

As discussed, based from previous literature studies, sulfoxide has potential applications in drug design and is found in living systems, contributing to unique bioactive properties (Scheme 1). Isolation, identification, and the mechanism of their action are important for future design of pharmaceuticals. The compatibility of a few of the selected synthesized sulfoxide molecules (**1b, 6b, 11b, 13b, 25b, 35b**, Scheme S48, Supporting Information) was assessed over HEK‐293 cells with the help of 3‐(4,5‐dimethylthiazolyl‐2)‐2,5‐diphenyltetrazolium bromide (MTT) assay over a wide range of concentrations (Figure S20, Supporting Information). The selected sulfoxides (**1b, 6b, 11b, 13b, 25b, 35b**) were found to be highly biocompatible, aligning their use as efficient proton pump inhibitors.^[^ ^53^ ^]^

Existing literature provides evidence for antioxidant properties of some sulfoxides and their significance in the biological system.^[^ ^53^, ^65^ ^]^ Oxidative stresses in biological systems initiate inflammatory responses and antioxidants can act as anti‐inflammatory agents.^[^ ^66^, ^67^ ^]^ Drugs containing sulfoxide moieties have been reported to serve as effective anti‐inflammatory agents. We investigated the antioxidant property of some of the selected synthesized sulfoxides (**1b, 3b, 6b, 10b, 11b, 12b, 14b, 23b, 25b, 29b**) using the Ferric Reducing Antioxidant Power (FRAP) assay,^[^ ^68^ ^]^ which exhibited their antioxidant potential, paving the way for exploring new chemical spaces. (Scheme S49–S50, Figure S21, Supporting Information).

## 3 Electronic Effect on Sulfide Oxidation Reaction

**Scheme 8:**
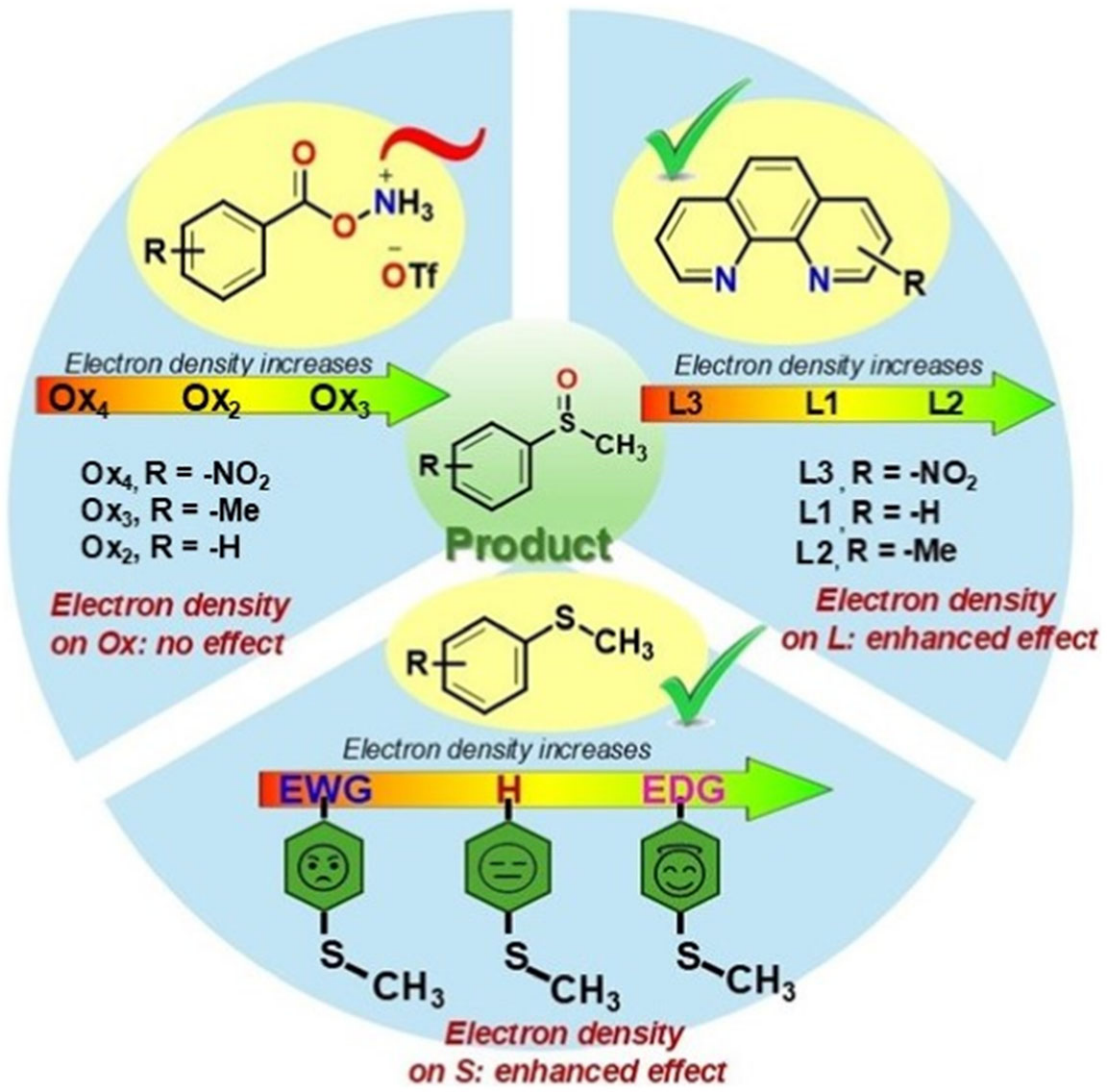
Stereoelectronic effects of different reaction components modulating the overall reactivity of sulfide transformation catalyzed by iron in the presence of suitable oxidant in water.

We next investigated the electronic effects influencing our developed functional group transfer protocol, which uncovered a new and promising reactivity trend. Specifically, we examined the roles of the supporting ligand used for the catalytic study, the amino source/oxidant, as well as the organic sulfide substrate, in shaping the observed outcomes (**Scheme** **8**). Changes in the ligand's electronic and steric properties were explored to evaluate their impact on the metal center's electronic environment and overall catalytic performance. Increased electron density on the supporting ligand (L) enhances the overall product formation (Scheme 8, see the reactivity trend of L1, L2, L3, and section XX, and Scheme S44, Supporting Information). However, with sterically hindered ligand backbone (L4), the yield of product decreased likely because of the steric hindrance of the amino source/oxidant (PivONH_3_OTf, Ox_1_) coordinated to the iron catalyst to generate the active iron–nitrogen intermediate for –*N* group transfer reactivity to organic sulfides (proposed mechanism, Scheme 10). Among other ligands, phenanthroline backbone gave the best result for a wider substrate scope. (Table 1, also see Supporting Information). Varying electron density of the amino source/oxidant (Ox_2_, Ox_3_, and Ox_4_, Scheme 8, section XX, Scheme S42, Supporting Information) and analyzing product formation from initial rate studies revealed that the electron density of amino source/oxidant has no significant role in modulating the overall reactivity.

Variations in the electronic nature of substituents on the sulfide seemed to play a significant role in modulating the efficiency of the group transfer reaction. Electron‐donating and electron‐withdrawing effects played a prominent role and in fact a competitive Hammett analyses with *p*‐substituted methyl phenyl sulfide versus normal methyl phenyl sulfide revealed a negative *ρ* value (*ρ* = −0.58) implicating the build‐up of positive charge in the transition state or involvement of electrophilic intermediate. (Scheme 8, also Scheme S45, and Figure S17, Supporting Information) These studies revealed valuable insights into the interplay of electronic factors of the reaction components, shedding light on the mechanism of the overall catalytic cycle (*vide infra*).

### 3.1 Mechanistic Experiments and Control Studies

**Scheme 9:**
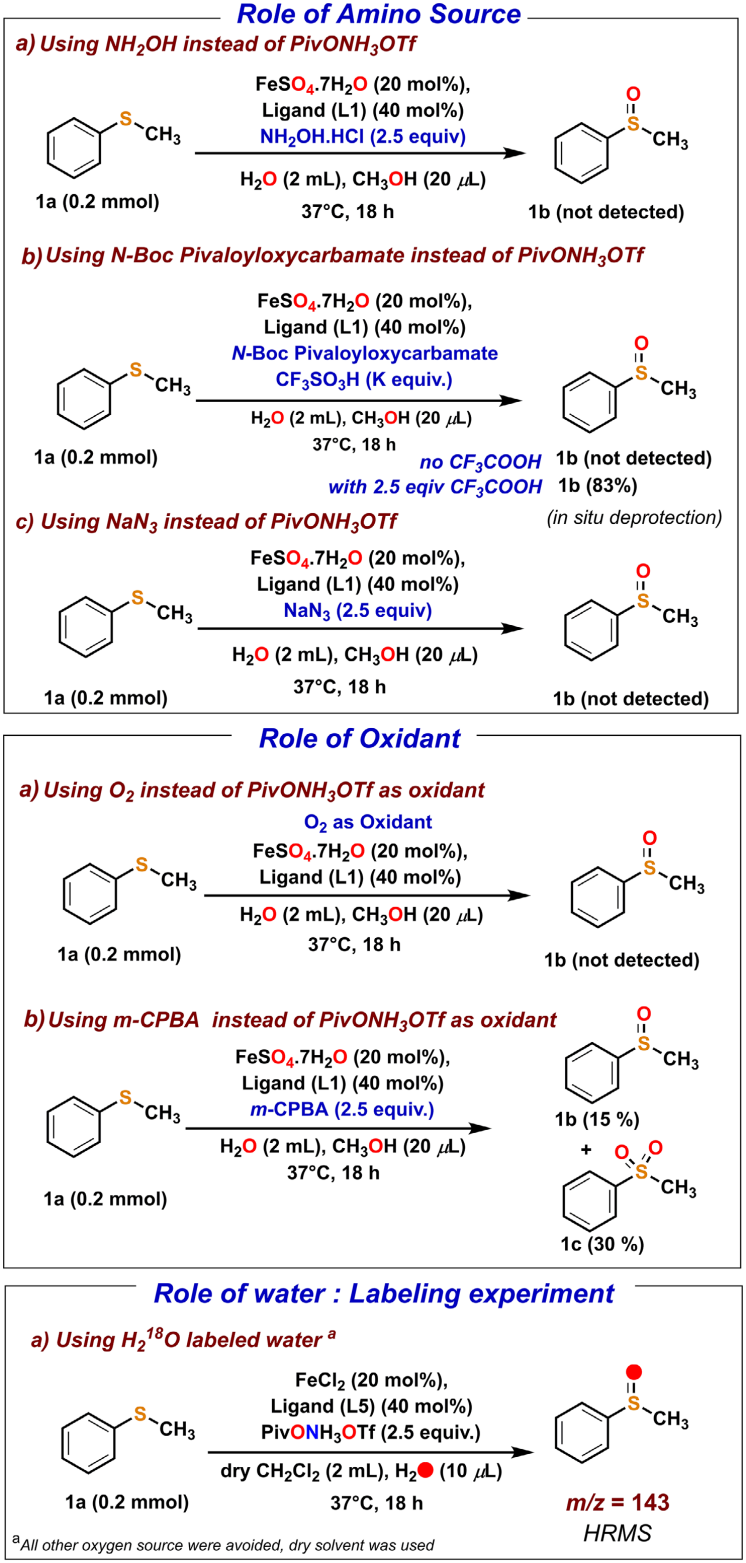
Experiments to assess the role of amino source (top box), role of oxidant (middle box) and the source of oxygen (bottom box) in product sulfoxide. Yield refer to ^1^H NMR yield.

The methodology involves the formation of new bonds on —S— (—S=O) via putative —S=NH species (Figure 1b) in a one‐pot reaction, directly starting from simple organic sulfide bonds, raising questions about the mechanism of this intriguing process. In the absence of iron catalyst, under standard reaction conditions, the yield of the reaction decreases drastically, highlighting the important role of iron in the reaction pathway (Table 1, entry 15). Under oxygen‐free environment (under N_2_), the standard optimized reaction proceeds with nearly equal efficiency, ruling out the involvement of molecular oxygen as the source of oxygen atom or oxidant (Table 1, entry 16, Scheme 9a, middle box). This was further confirmed using dioxygen as the oxidant, instead of PivONH_3_OTf (Ox_1_) where no conversion of sulfide (1a) was detected. (**Scheme** **9**, also section XII, Supporting Information).

To further understand the role of PivONH_3_OTf (Ox_1_) in the reaction, we performed a few control experiments (Scheme 9, section XI, Supporting Information). Using hydroxyl amine (Scheme 9a top box) or *N*‐Boc‐protected pivaloxycarbamate (Scheme 9b, top box) as reagents instead of PivONH_3_OTf (Ox_1_) did not yield any product formation (Scheme 9). However, *N*‐Boc pivaloxycarbamate along with triflic acid resulted in 83% of selective methyl phenyl sulfoxide (1b) formation, implicating an in situ deprotection of *N*‐Boc pivaloxycarbamate, that generates PivONH_3_OTf (Ox_1_), one of the essential components of the reaction (Scheme 9b, top box, section XI, Scheme S21, Table S10, Supporting Information). Using sodium azide (NaN_3_) instead of PivONH_3_OTf (Ox_1_) gave no product (Scheme 9c, top box). Other potent oxidants like meta‐chloroperbenzoic acid (*m*‐CPBA) under the standard reaction condition in water gave an overall reduced conversion of sulfide (40%) with almost no selectivity (Scheme 9b, middle box). Thus, all these mechanistic and control experiments as well as preliminary spectroscopic studies (see Electron Paramagnetic Resonance, (EPR) experiment, Figure S22, Supporting Information) along with previous literature precedents, implicate the involvement of iron–nitrogen intermediate as the facile *N*‐transfer platform for chemoselective transformation of sulfide to sulfilimine during the course of reaction. While the origin of the N atom can be confidently assigned to the aminating reagent in the putative sulfilimine species, further transformation of sulfilimine to sulfoxide and the source of the oxygen atom in the final sulfoxide product raises additional questions regarding the subsequent mechanistic steps.

To assess the source of oxygen into the sulfoxide product, labeling experiments with ^18^O labeled water (H_2_^18^O) confirmed that H_2_O acts as the source of oxygen (in ESI‐MS, *m/z* = 141 for sulfoxide (1b) shifted to *m/z* = 143) (Scheme 9a, bottom box, Figure S8, Supporting Information). This further validates the crucial role of water as a driving force for this reaction development. Reactions were also performed with the isolated catalyst [(L_1_)_2_FeCl_2_] (M1) which gave high yield of the product sulfoxide (Scheme S15, Table S10, Supporting Information) ensuring that a 1:2 metal catalyst: ligand composition is likely involved in the reaction pathway to generate the active catalytic species.

Oxygen–atom transfer reactions often use high‐energy oxygen–atom donor reagents. Metal–oxyl complexes, which are electronically equivalent species of the corresponding metal–oxo complexes, for example, Fe(IV)=O vs Fe(III)‐oxyl radical, with one‐electron‐reduced metal centers, have not been reported as often, though the species is expected to exhibit different reactivities in substrate oxidation reactions compared to the metal–oxo complexes.^[^ ^69^ ^]^ Infact, Iron–oxyl complexes have not yet been isolated and characterized experimentally, most studies rely on DFT calculations (Scheme 2b).^[^ ^69^ ^]^ Added to that, these metal‐oxo/oxyl intermediates often generate free OH· radical, leading to nonselective oxidation products. The strategy reported in this work enables to achieve selective oxo group transfer protocol under ambient conditions circumventing the need for generating high‐valent metal intermediates, albeit maintaining a high level of reactivity and selectivity.

## 4 Computational Calculations

**Figure 2:**
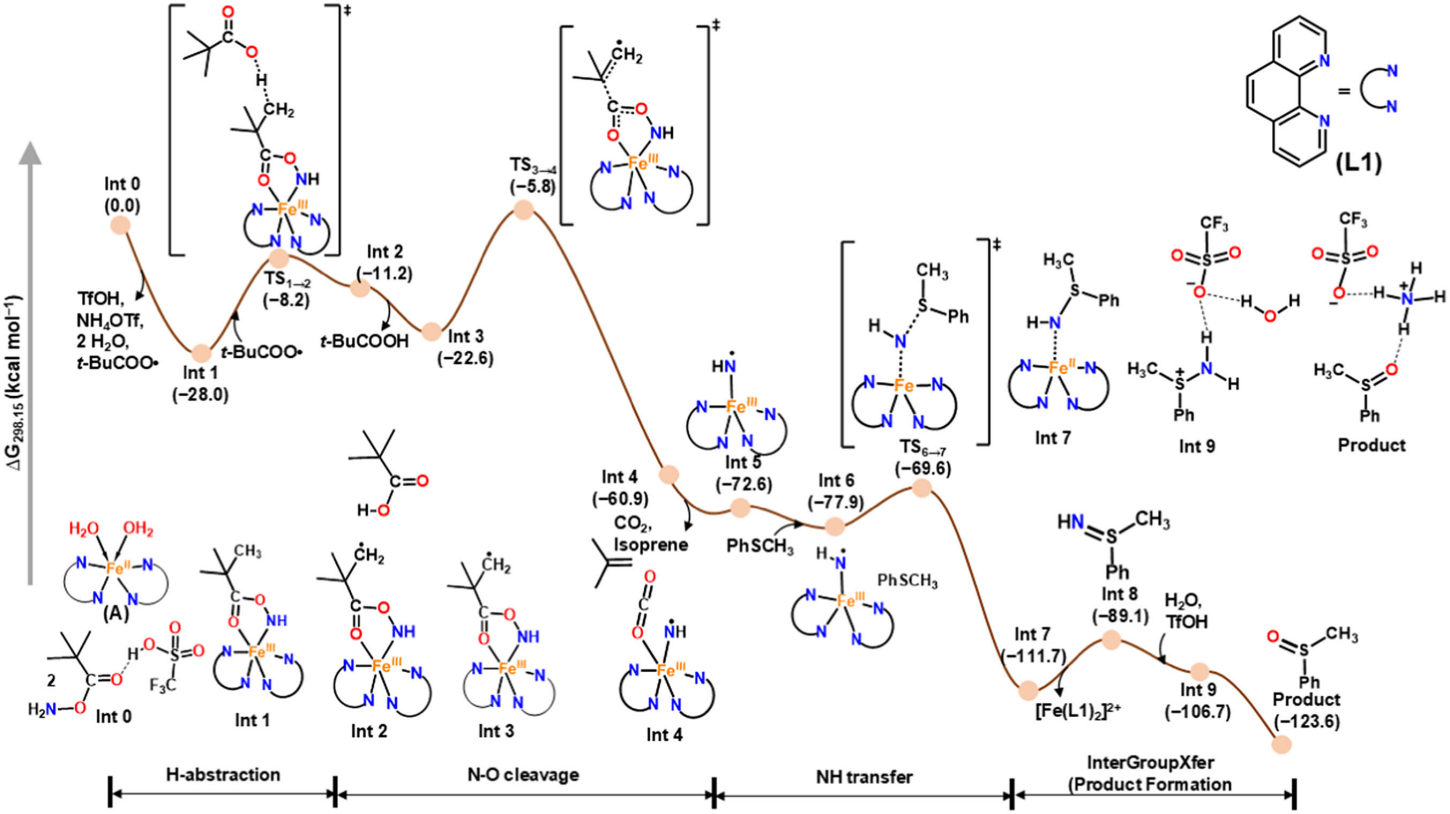
Calculated mechanistic pathway for the iron‐catalyzed chemoselective sulfoxidation of organic sulfides by complex A and PivONH_3_OTf acts as the oxidant and amino source. All the DFT computations were performed at the CPCM(H_2_O)−B3LYP‐D3BJ/def2‐TZVP//B3LYP‐D3BJ/def2‐SVP level of theory. Values in parenthesis are relative Gibbs energy values given in kcalmol.^−1^

A detailed computational calculation (see computational section of SI for details) was performed to correlate the experimental observations with Density Functional Theory (DFT) calculations and get a holistic understanding of the intriguing reaction pathway utilizing ORCA5.0.3 quantum chemical program^[^ ^70^, ^71^ ^]^ (**Figure** **2**). Density functional theory calculations were performed using B3LYP density functional^[^ ^72^, ^73^, ^74^, ^75^ ^]^ incorporating Grimme's D3 dispersion^[^ ^76^ ^]^ with Becke–Johnson damping.^[^ ^77^ ^]^ Implicit solvation was considered using the Conductor‐like Polarizable Continuum Model (CPCM model)^[^ ^78^ ^]^ (water as a solvent) to account for the solvation effect. DFT calculation reveals one of the lowest‐energy plausible reaction pathways as depicted in Figure 2, consisting of multiple reaction intermediates.

From the calculated spin‐state energetics, in aqueous medium, high‐spin Fe^II^ catalyst (**A**) [S_*t*_ = 2, (2S_*t*_ + 1) = 5] in the *cis*‐configuration coordinated by two phenanthroline ligands (**L1**) and two labile *cis*‐water molecules is the most stable conformer (see SI for details). The high‐spin Fe^II^ catalyst (**A**) is activated by the first equivalent of the oxidant PivONH_3_OTf, (**Ox**_**1**_) to undergo one‐electron oxidation of Fe^II^ to Fe^III^ with the removal of TfOH, NH_4_OTf, and two molecules of H_2_O, as well as *t*BuCOO^•^ radical, along with the coordination of the second equivalent of the oxidant PivONH_3_OTf, (**Ox**_**1**_) to form **Int I**, an Fe^III^‐*N*‐acyloxy [S_*t*_ = 5/2, (2S_*t*_ + 1) = 6] species (Figure 2). Other possible spin states and binding motifs were also computed for **Int 1** to determine the most stable geometry and configuration (See SI for details). However, **Int 1** having high‐spin Fe^III^ (S_*t*_ = 5/2) state, in the six‐coordinate distorted octahedral geometry where the oxidant (**Ox**_**1**_) coordinates to the iron center both through the nitrogen and keto oxygen Fe(N + O), has emerged as most thermodynamically favorable (for more information see SI). Subsequently, **Int 1** undergoes decomposition via a two‐step pathway. The *t‐*BuCOO^•^ radical which is generated during the activation of the Fe^II^ catalyst (**A**) by the hydroxyl amine‐derived N − O oxidant (Ox_1_) abstracts a hydrogen atom from **Int 1** via TS_1→2_, leading to the formation of a transient radical intermediate **Int 2** with an activation barrier of 20.2 kcal mol^−1^ and aligning with the experimental reaction temperature of 25–30 °C. In the next step, facile N—O bond cleavage occurs via TS_3→4_ with an activation barrier of 22.2 kcal mol^−1^. Calculation reveals that the hydrogen abstraction process by *t*BuCOO^•^ radical lowers the barrier for the cleavage of the N—O bond of **Int 3** leading to formation of **Int 4** with relative Gibbs energy of −60.9 kcal mol^−1^ (relative to **Int 0**). Subsequently, CO_2_ and isoprene are released and **Int 5** (ΔG = −72.6 kcal mol^−1^) is formed. In the following step, **Int 5**, an Fe^III^‐iminyl radical species (Fe ^III^−NH•) with Fe^III^ in intermediate (S_*1*_ = 3/2) spin state and a nitrogen‐based radical NH• (S_*2*_ = 1/2), couples antiferromagnetically to form an overall singlet‐spin state [S_*t*_ = 1, (2S_*t*_ + 1) = 3] and is energetically the most stable conformer (Table S26, Figure S27, Supporting Information). **Int 5**, an Fe^III^‐iminyl radical species (Fe^III^−NH•), thus formed upon N—O bond cleavage has a labile Fe—N bond (Table S26, Supporting Information) for facile *N*‐transfer reaction (see computational details of SI).

Interestingly in the next step, calculation reveals that the –“*NH*” group transfer process to the organic sulfide (PhSCH_3_) proceeds via a doublet spin state [S_*t*_ = 2; (2S_*t*_ + 1) = 5], instead of the singlet spin state, involving iron in the intermediate spin state (S_*1*_ = 3/2), formed likely by a ferromagnetic coupling between the S_*1*_ = 3/2 spin state of Fe^III^ and NH• radical (S_*2*_ = 1/2). An Fe^II^‐sulfilimine adduct (**Int 7**) is thus formed via a transition state TS_6→7_ with an activation barrier of 8.3 kcal mol^−1^ through a chemoselective *N*‐transfer reaction from **Int 5** (Figure 2). The activation energy of **Int 7** is quite small, thereby regenerating the Fe^II^ catalyst (**A**) for the next cycle. The putative sulfilimine product (**Int 8**) thus released in the aqueous reaction environment in the presence of triflic acid is held in space through H‐bonding interaction in such a disposition (**Int 8**) that it is spontaneously converted to the corresponding sulfoxide selectively, with no scope for overoxidation to the corresponding sulfone or sulfoximine, thereby making the conversion chemoselective.

Thus based on DFT calculation presented in this work, it is evident that though the precursor Fe^II^ catalyst (**A)** (S_*t*_ = 2) as well as the putative Fe^III^‐*N* acyloxy intermediate (**Int 1)** (S_*t*_ = 5/2) are high spin, a spin crossover of iron to an intermediate spin S_*1*_ = 3/2 in **Int 5**, the active “*NH*” transfer species occurs for selective *N*‐transfer reaction.(See computational section, SI).

Unlike the previous reported literature of “all‐high‐spin states of iron”, involving Fe^II^ catalyst based on acetyl acetonate ligand, which formed a putative Fe^III^–iminyl species where the high‐spin Fe^III^ (S_*1*_ = 5/2) was antiferromagnetically coupled to a nitrogen based radical (S_2_ = 1/2) to form an overall S_*t*_  = 2 spin state (2S_*t*_  + 1 = 5) for *N*‐transfer reaction to alkenes,^[^ ^42^ ^]^ the mechanism proposed in this work involves an intermediate spin state of Fe^III^ as one of the proposed lowest‐energy pathways for successful *N*‐transfer reaction to organic sulfides. This further unveils that modulating electronics and steric of ligand architecture and thus fine tuning iron spin states as well as reaction environment (water as solvent in this work) can unfold a plethora of new reactivities and selectivites, which are yet to be explored with the hydroxyl amine‐derived reagent and iron catalysis.

In this work, the choice of phenanthroline (**L1**) ligand clearly highlights its role in controlling the catalytic pathway. Phenanthroline (**L1**) although a poor *σ*‐donor ligand, being a rigid planar, hydrophobic, system with two inward‐pointing nitrogen donor atoms being held juxtaposed and, therefore, preorganized for strong metal binding,^[^ ^79^ ^]^ provides the optimum electronic (Scheme 8) and steric environment around the iron center for oxidation and amine transfer reaction in the presence of PivONH_3_OTf (**Ox**_**1**_). A similar disposition of the nitrogen donors in bipyridine (**L5**) and terpyridine (**L6**) as well as other ligands used in this study (**L10, L11**) (See Table 1) might be disrupted by the free rotation about the bond(s) linking the heteroaromatic six‐membered rings, thereby reducing the overall catalytic efficiency. The experimental results and DFT calculations based on the iron‐phenanthroline system clearly point out that apart from providing the right steric around iron, the *π*‐electron deficiency of phenanthroline enhances its *π* acceptor capability,^[^ ^79^ ^]^ thereby stabilizing iron in lower oxidation state of Fe(III) to form the active Fe^III^‐iminyl radical intermediate for a metalloradical‐assisted functional group transfer reaction, without the need for traversing to the sensitive high‐valent regime.

### 4.1 Proposed Mechanism

**Scheme 10:**
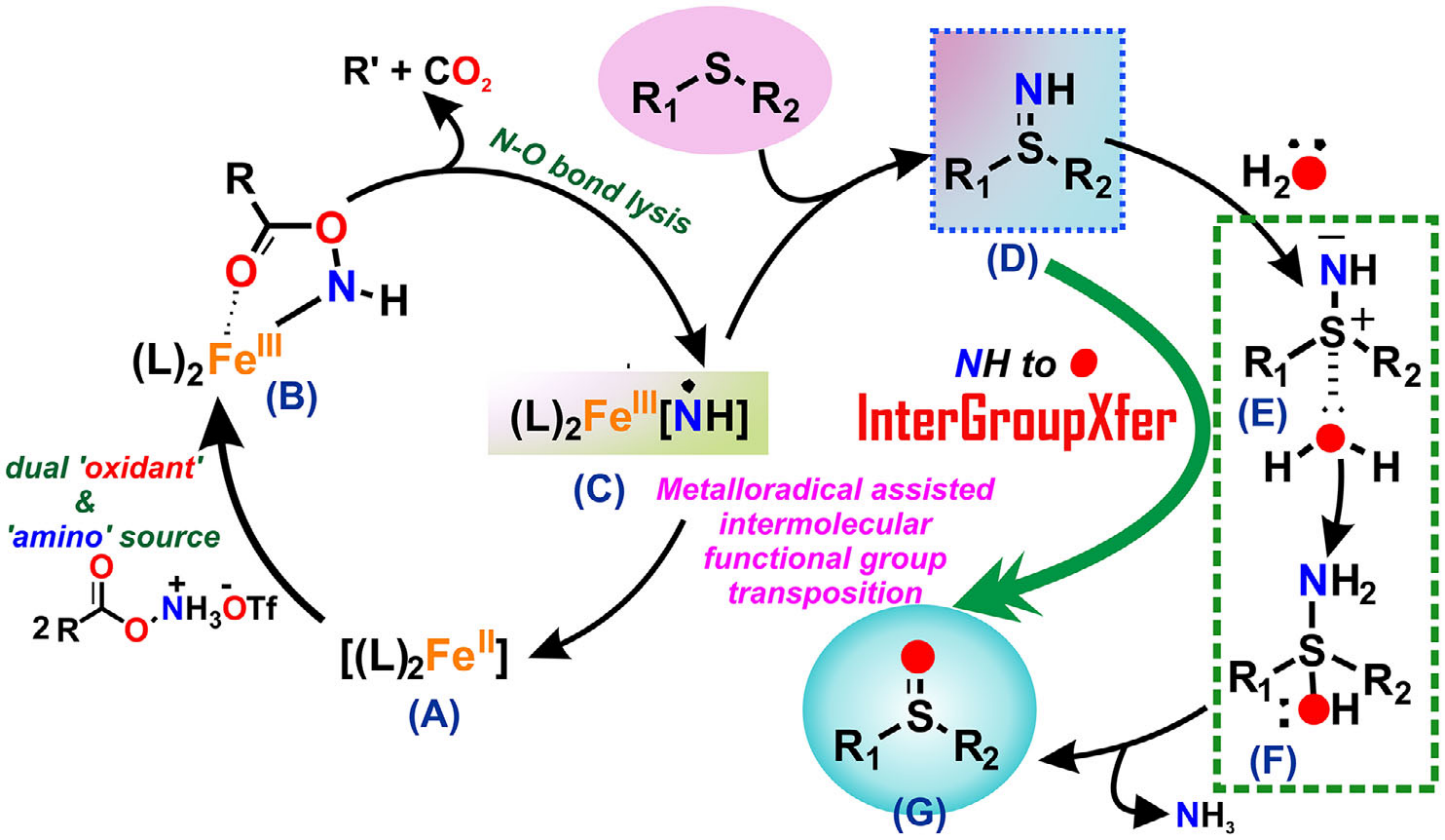
Proposed mechanism for iron‐catalyzed sulfide oxidation in water.

Based on the mechanistic experiments and detailed DFT calculations (Figure 2), we propose a new mechanism for the sensitive functional group transfer protocol. An hydroxyl amine‐derived reagent acts as an oxidant and amino source to activate the Fe(II) catalyst (**A**) and generate a putative Fe^III^‐iminyl intermediate (**C**) (**Scheme** **10**), with an unusually long Fe—N bond length (1.71 Å, see DFT calculation).This iron coordinated nitrogen radical intermediate has no *N*‐substitution to stabilize the electron density on metal–nitrogen vector, ideally poised for transferring the *N*‐functionality. A facile *N*‐transfer reaction to organic sulfides produces the corresponding sulfilimine (**D**) in situ (probed experimentally and supported by DFT calculations). This free NH‐sulfilimine (**D**) undergoes an intermolecular functional group transposition or *InterGroupXfer* in the presence of water as the nucleophile and benign source of oxygen atom to form the corresponding sulfoxide (**G**) chemoselectively with excellent yield and broad substrate scope (Scheme 10). The transfer of X group (here ‐*O* transfer via ‐*NH*) to sulfides regenerates the Fe(II) catalyst thereby making the overall reaction catalytically efficient and selective.

## 5 Conclusion

In summary we have designed a sustainable catalytic protocol, unlocking an ecofriendly ‐*NH* to ‐*O* functional group transfer strategy for selective oxidation of sulfides to sulfoxides in a one‐step process, utilizing Earth‐abundant iron catalyst and bench‐stable and convenient‐to‐handle surrogate hydroxyl amine‐derived aminating agent PivONH_3_OTf, (Ox_1_) under mild reaction conditions with a broad substrate scope, excellent functional group tolerance, and versatile scalability, maintaining all fitness factors required for assessing the catalytic reaction protocol as the bio‐orthogonal chemical probe. The subtle interplay between the iron‐coordinated *N*‐centered radical and water as solvent and oxo source plays a key role for the metalloradical‐assisted Intermolecular Functional Group Transposition or InterGroupXfer, to selectively transform readily available sulfides to valuable sulfoxides, circumventing the need for the use of any external oxidant, precious metal catalyst, or any sensitive and reactive high‐valent iron intermediates. A comprehensive electronic and mechanistic investigation, supported by DFT calculations, has been conducted to elucidate the reaction mechanism. The utility of the developed reaction methodology has been exploited in designing synthetic protocols of various C—C coupling as well as C—S bond activation reactions, of synthetic and pharmaceutical relevance. Additional biocompatibility studies and investigations into the antioxidant reactivity of the synthesized complex sulfoxide molecules have been conducted, paving the way for the exploration of new chemical space. The discovery of more unusual and challenging transformations different from the conventional approaches presented in this work, with unprecedented synthetic flexibility and operational simplicity, is expected to contribute interesting development with the prospect of green and sustainable chemistry and is expected to unlock new concepts in the emerging research area of catalytic functional group transfer reactivity.

## Conflict of Interest

The authors declare no conflict of interest.

## Supporting information

Supplementary Material

## Data Availability

The data that support the findings of this study are available in the supplementary material of this article.
