# Supplementary material for: An Iron‐Catalyzed Sustainable Functional Group Transfer Strategy for In‐Water Transformation of Organic Sulfides to Sulfoxides by a Hydroxylamine‐Derived Oxidant
Source: ChemSusChem. 2025 Apr 11;18(12):e202500032. doi: 10.1002/cssc.202500032 (PMC12175040; doi:10.1002/cssc.202500032)
Supplement: Supplementary file 1 — Supplementary Material [file CSSC-18-e202500032-s001.pdf]

Supporting Information

An Iron-Catalyzed Sustainable Functional Group Transfer Strategy for In-Water Transformation of Organic Sulfides to Sulfoxides by a Hydroxylamine Derived Oxidant

Arya Singh# [a], Yashdeep Maurya# [a], Akhilesh Sharma[a], Swetha V.S.,[a], Mehar Ul Nisa[a], Vishal Kumar[a], Puneet Gupta[a], Kartikey Tyagi[a] and Sayanti Chatterjee*[a,b]

# - both these authors contributed equally

Department of Chemistry, Indian Institute of Technology Roorkee,

Roorkee, Uttarakhand, India. PIN-247667

Email: sayanti.chatterjee@cy.iitr.ac.in

Table of contents

1) Abbreviations……………………………………………………………………………….4
2) Experimental Section: Materials and Methods…………………………………………...6
3) Synthesis of Oxidants……………………………………………………………………...8
4) Reaction optimization…………………………………………….................................12
5) Analysis of Reaction Profile by HR-MS………………………………………………..30
6) Synthesis procedure for Iron (II) catalyst……………………………………………..32
7) Catalytic study with Isolated Iron (II) catalyst…………………………….............32
8) Control experiments…………………………………………………………………....32
9) Effect of other Oxidants……………………………………………………………….42
10) Radical Scavenger reactions…………………………………………………………44
11) ^{18}O Labelling experiments…………………………………….......................46
12) Reaction in aqueous buffer solution…………………………………………………48
13) Competitive Reaction……………………………………………………….............50
14) Variation of scale of developed methodology…………………………………...…52
15) Reaction compatibility in presence of bio-additives…………………………...…53
16) General procedures for the sulfide oxidation………………………………………57
17) Mechanistic study………………………………………………………………….…...58
18) Mechanochemical approach: Solvent free condition……………………………….67
19) Biocompatibility study for cell viability analysis……………………………………70
20) Antioxidant study……………………………………………………………………….71
21) Asymmetric analysis…………………………………………………………………...74
22) Application of developed methodology……………………………………………….74
23) EPR and GC-MS Experiments…………………………………………………………79
24) Unsuccessful substrates…………………………………………………….........81
25) X-ray crystallographic files……………………………………………….............82
26) Synthesis and characterization of sulfoxides………………………………………...91

27) Synthesis of sulfides……………………………………………………………………………….110

28) Spectral data…………………………………………………………………………….........120

29) Computational Calculations………………………………………………………………………229

Abbreviations

Technical terms

Equiv.	equivalent
r.t.	room temperature
J	coupling constant (cm^{-1})

Techniques

IR	infrared spectroscopy
NMR	nuclear magnetic spectroscopy
UV- Vis	ultraviolet- visible spectroscopy

Units

cm	centimeter
h	hour
℃	degree Celsius
M	molar
mM	milimolar
*μ*L	microlitre
min	minute
m	metre
G	gauss

Latin expressions

et al.	and co- workers
e.g.	for example
i.e.	namely

tert	tertiary

Solvents and reagents

CH_2Cl_2/ DCM	dichloromethane
$CHCl_3$	chloroform
m-CPBA	*m*-chloroperoxybenzoic acid
Et_2O	diethyl ether
DME	1,2-dimethoxyethane
EtOAc	ethylacetate
MeCN	acetonitrile
DMSO	dimethyl sulfoxide
DMF	Dimethyl formamide
THF	tetrahydrofuran
HFIP	hexafluoroisopropanol
NaN_3	sodium azide
MeOH	methanol
NEt_3	triethylamine
K_2CO_3	potassium carbonate
Boc	*tert*-Butyloxycarbonyl
*t*Bu	*tert*-Butyl
HCl	hydrogen chloride
H_2SO_4	sulfuric acid
NaCl	sodium chloride
Na_2SO_4	sodium sulfate
$NaHCO_3$	sodium bicarbonate
$FeCl_2$	ferrous chloride
Obn	O-benzylic
KBr	Potassium bromide
Bipy	bipyridine
$Fe(acac)_2$	iron(II) acetylacetonate
TEMPO	2,2,6,6-Tetramethylpiperidinyloxyl
KO*t*Bu	Potassium *tert* -butoxide

Experimental Section

Materials and Methods

All chemicals and reagents were obtained from commercial sources and were used without further purification unless otherwise noted. Anhydrous $FeCl_2$ and $FeSO_4.7H_2O$ was purchased from TCI and Sisco Research laboratories Pvt. Ltd. (SRL). Solvents were purchased from commercial sources and used as it is. For reactions under dry solvent, solvents were distilled and degassed using standard procedure. All chemicals used in the research were purchased from commercial sources and used without further purification unless otherwise mentioned. Metal catalysts and ligands were purchased from TCI or BLD and used without purification. The catalysts which were synthesized has been included in the experimental portion.

The reagent $PivONH_3OTf$ (Ox_1) and *O*-benzoyl Aminating Agents (Ox_2, Ox_3, Ox_4) were synthesized following slight modification of the literature procedure.[1, 2]

NMR spectroscopy: Nuclear Magnetic Resonance spectra were measured using a Jeol spectrometer (500MHz). ^{1}H-NMR was reported as follows: chemical shift, multiplicity (s = singlet, d = doublet, t = triplet, q = quadruplet, m =multiplet and br = broad), coupling constant (J values) in Hz and integration. Chemical shifts (δ) were reported with respect to the corresponding solvent residual peak at 7.26 ppm for $CDCl_3$ and 1.94 for CD_3CN for ^{1}H-NMR. ^{13}C-NMR spectra (^{1}H-broadband decoupled) were reported in ppm using the central peak of $CDCl_3$ (77.16 ppm), and CD_3CN (118.26). Spectra were processed with MestReNova 16.0.0 and coupling constants are reported as observed.

UV-Vis Absorption Spectroscopy [UV-Vis ABS] - The Agilent Spectrophotometer (–Cary 60) instrument was used to record UV- Visible spectra using a pair of quartz cuvette of pathlength 1 cm at 25 – 30℃.

Mass Spectrometry: High resolution mass spectrometry (HR-MS) was performed on an Agilent 6545 LC/Q-TOF in ESI mode. The ionization method and mode of detection employed is indicated for the corresponding experiment and all masses are reported in atomic units per elementary charge (*m/z*) with an intensity normalized to the most intense peak.

Single Crystal X-Ray Diffractometer: Single Crystal X-Ray Diffraction Data collection was collected in Bruker APEX-II CCD diffractometer (See crystallographic section for details). The structure was solved and refined by Software package APEX-4 and Olex2.

Chromatography - Thin Layer Chromatography analyses were performed on silica gel coated glass plates (0.25 mm) with fluorescence-indicator UV254 (Macherey-Nagel, TLC plates SIL G 60 F254) purchased from Merck. For detection of spots, irradiation of UV light at 254 nm, and staining reagents as needed. Solvent mixture of ethyl acetate and hexane of up to 5% to 80% polarity were used as eluent depending on the polarity of products.

EPR instrumentation: Electron paramagnetic resonance (EPR) spectra were obtained using Bruker Biospin, EMXmicro A200-9.5/12/S/W at 100K. The instrument parameters for EPR were set as at 100 kHz modulation frequency, 1×10^3 receiver gain, 3.00 G modulation amplitude, 5.120 ms time constant, 75.00 ms conversion time, 3300 G center field, 100 G sweep width, 4 dB microwave attenuation.

Polarimeter: Optical rotations were measured in a Rudolph Polarimeter AUTOPOL-V PLUS.

GC-MS: GC-MS was recorded using Perkin-Elmer Mass Spectrometer and Agilent Gas Chromatography Mass Spectrometry.

Experimental Procedures

1. Synthesis of Oxidant (Ox_1)

Step 1: Synthesis of *tert*-Butyl pivaloyloxy carbamate (B)

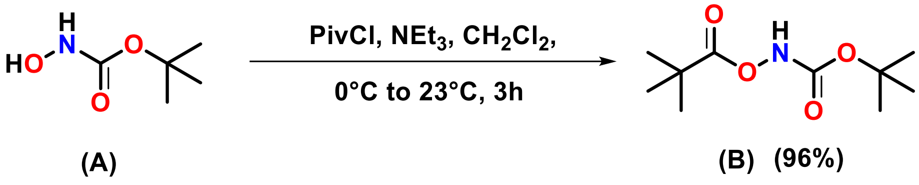

Scheme S1. Synthesis of *tert*-Butyl pivaloyloxy carbamate.

A 250-mL round-bottomed flask, equipped with a magnetic stirbar was charged under air subsequently with *N*-Boc hydroxylamine (A) (15 g, 0.115 mol, 1.0 equiv.), dichloromethane (CH_2Cl_2, 0.2 L, 0.6 M) and triethylamine (16 mL, 11.5 g, 0.115 mol, 1.0 equiv.). The reaction vessel was placed in an ice-water bath and stirred until all solids dissolved. Then, the round-bottomed flask was equipped with a 50-mL dropping funnel which was filled with pivaloyl chloride (PivCl, 14 mL, 14 g, 0.115 mol, 1.0 equiv.). The dropping funnel was sealed with a rubber septum equipped with a balloon. PivCl was added dropwise to the stirred, clear reaction mixture over 30 minutes. After complete addition, the reaction mixture was stirred for 30 minutes before the ice bath was removed. After additional 2 hours stirring at room temperature, the reaction mixture was worked up. For that, the resulting suspension was vacuum filtered and the filter cake was washed with CH_2Cl_2 (2 x 20 mL). The filtrate was transferred to a separation funnel and washed subsequently with distilled water (100 mL), a saturated aqueous solution of $NaHCO_3$ (100 mL) and a saturated aqueous solution of NaCl (50 mL). The final organic layer was dried over anhydrous Na_2SO_4 for 15 min of stirring in a 1000 mL beaker and vacuum filtered. The filter residue was washed with CH_2Cl_2 (2 x 10 mL). Subsequent removal of solvent on a rotary evaporator afforded 23.5 g (96 %) of *tert*-butyl pivaloyloxy carbamate **(B)** as a white solid.

The white solid has the following characteristics which match with the literature report[2, 3].

^{1}H NMR (500 MHz, CDCl$_3$) δ 7.76 (s, 1H), 1.49 (s, 9H), 1.30 (s, 9H).

^{13}C NMR (126 MHz, CDCl$_3$) δ 178.0, 155.8, 83.2, 38.3, 28.2, 27.1.

Step 2. Synthesis of *O*-pivaloyl hydroxylamine triflic acid (Ox_1)

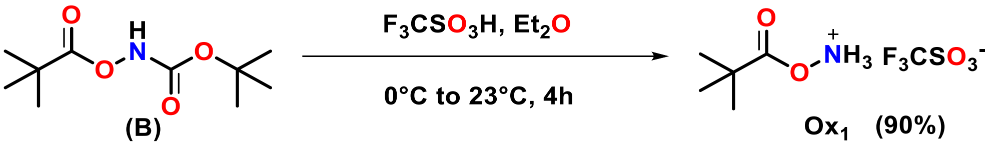

Scheme S2. Synthesis of Pivaloylhydroxylamine triflic acid salt.

In air, a 500-mL round-bottomed flask equipped with a magnetic stirbar is charged with 23 g *tert*-butyl pivaloyloxy carbamate (**B**) (0.1059 mol, 1.0 equiv.) followed by 0.2 L of diethylether (Et_2O) (0.6 M). The reaction vessel was placed in an ice/water bath and stirred until all solids have dissolved. Then, the round-bottomed flask was equipped with a 50-mL dropping funnel which was filled with triflic acid (9.2 mL, 15.6 g, 0.104 mmol, 1.0 equiv). The acid was added over 30 min and left for further 30 min before the ice/water bath was removed. During additional 3 hours of stirring at room temperature, a white precipitate was formed. Additional colorless precipitate formed upon addition of *n*-pentane (75 mL). Subsequently, the suspension was vacuum filtered, the reaction vessel rinsed with *n*-pentane (3 x 25 mL) and the filter cake carefully washed with ice-cold CH_2Cl_2 (3 x 25 mL). Upon drying under high vacuum for 16 h, *O*-pivaloyl hydroxylamine triflic acid (**Ox_1**) was obtained as a colorless, crystalline, free flowing solid (25 g, 0.094 mol, 90%) whose NMR matches with the literature report.[2, 3]

^{1}H NMR (500 MHz, CD_3CN) *δ* 9.89 (s, 3H), 1.29 (s, 9H).

^{13}C NMR (126 MHz, CD_3CN) *δ* 175.2, 121.6 (q, *J* = 319.3 Hz), 39.1, 26.8.

^{19}F NMR (471 MHz, CD_3CN) *δ* -79.36.

2. Synthesis of Different *O*-benzoylhydroxylamine triflic acid Aminating Agents.

All the aminating reagents (Ox_2, Ox_3, Ox_4) were synthesized following a general procedure as follows: The corresponding acyl chlorides (1.1 equiv.) and trimethylamine (1.1 equiv.) were added to the solution of *tert*-butyl hydroxycarbamate (13.3g, 100 mmol, 1.0 equiv.) in Et_2O (800 ml) at 0°C. The reaction mixture was stirred for 4h-8h until the *tert*-butyl hydroxycarbamate was completed consumed as monitored by TLC. Then the content was filtered. The filtrate was collected and washed with saturated $NaHCO_3$ three times. The combined organic layers were washed with brine and dried over anhydrous Na_2SO_4. After removal of the solvent, the crude product was recrystallized to afford the white solid. The solid obtained was dissolved in Et_2O (500 ml). Then trifluoromethanesulfonic acid (1.1 equiv. 11.25 mL was added dropwise over 30 min at

0°C in ice/water bath to acidify the reaction mixture and left to stir overnight at room temperature. Whiteprecipitate was formed. Subsequently, the precipitate was vacuum filtered and washed with petroleum ether (3 x 25 mL) to obtain the desired reagents which were dried under high vacuum for 16 h. The following yields were calculated: 76 % for Ox_2 using benzoyl chloride, 77 % for Ox_3 using 4-Methyl benzoyl chloride, 80 % for Ox_4 using 4-Nitro benzoyl chloride.

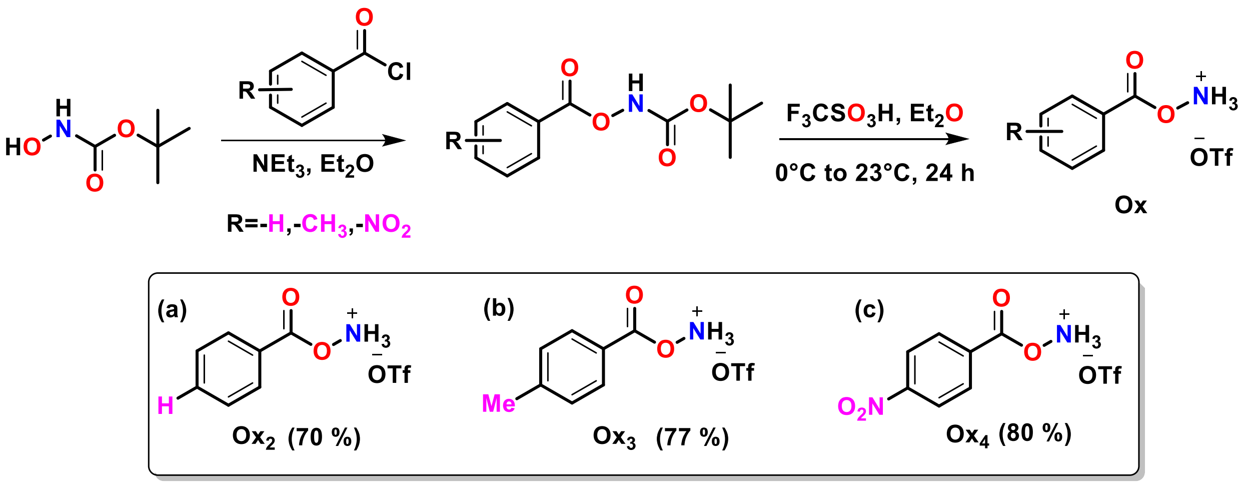

Scheme S3. Synthesis of different *O*-benzoylhydroxylamine triflic acid salt. a) *O*-benzoylhydroxylamine triflic acid (Ox_2). b) *O*-(4-methyl benzoyl)hydroxylamine triflic acid(Ox_3). c) *O*-(4-nitrobenzoyl)hydroxylamine triflic acid(Ox_4).

The isolated solids have the following characteristics which match with the literature reports.[1]

a) NMR of *O*-benzoylhydroxylamine triflic acid (Ox_2)

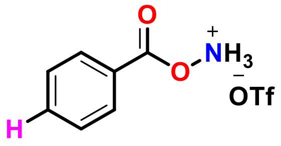

Ox_2

^1H NMR (500 MHz, CD_3CN) δ 8.05 – 8.00 (m, 2H), 7.78 (ddt, *J* = 8.7, 7.2, 1.3 Hz, 1H), 7.62 – 7.56 (m, 2H).

^{13}C NMR (126 MHz, CD_3CN) δ 162.89, 135.88, 130.07, 129.48, 124.24, 117.44.

^{19}F NMR (471 MHz, CD_3CN) δ -79.37.

b) NMR of *O*-(4-Methyl benzoyl)hydroxylamine triflic acid (Ox_3)

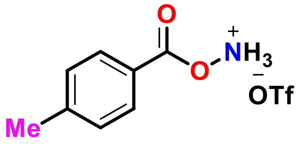

Ox_3

^{1}H NMR (500 MHz, CD_3CN) δ 10.18 (s, 3H), 7.91 – 7.87 (m, 2H), 7.39 – 7.35 (m, 2H), 2.40 (s, 3H).

^{13}C NMR (126 MHz, CD_3CN) δ 162.84, 147.50, 130.12, 130.07, 121.30, 117.52, 21.05.

^{19}F NMR (471 MHz, CD_3CN) δ -79.37.

c) NMR of *O*-(4-Nitro benzoyl) hydroxylamine triflic acid (Ox_4)

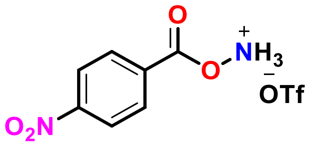

Ox_4

^{1}H NMR (500 MHz, CD_3CN) δ 8.39-8.26 (d, 2H), 8.29 – 8.16 (d, 2H).

^{13}C NMR (126 MHz, CD_3CN) δ 161.58, 152.00, 131.52, 124.36,129.85, 117.48.

^{19}F NMR (471 MHz, CD_3CN) δ -79.37.

Choice of metal catalyst(I):

A screw-cap vial was charged with a metal catalyst (0.02 mmol) (mentioned in table S1), and a stirring bar. To it solid oxidant $PivONH_3OTf$ (Ox_1) (133.6 mg, 0.5 mmol, 2.5 equiv.) was added followed by immediate addition of 2.0 mL of H_2O and 20 μL CH_3OH via syringe. Methyl phenyl sulfide,**1a** (23.5 μL, 0.2 mmol) substrate was added to the reaction mixture immediately after this and the reaction vial was sealed and stirred at 37 °C for 18 h. After 18 h the reaction mixture was diluted with saturated $NaHCO_3$ (2 mL) and 1,3,5-trimethoxybenzene (33.6 mg, 0.2 mmol,1 equiv. w.r.t the substrate was added as ^{1}H NMR internal standard. and stirred for 20 mins. The water phase was then extracted with CH_2Cl_2 (3 x 5 mL). The combined organic phases were washed with brine and dried over anhydrous Na_2SO_4, filtered, and concentrated in *vacuo*.

The yield of Methyl phenyl sulfoxide product (1b) was determined by ^{1}H NMR with respect to 1,3,5-trimethoxybenzene as internal standard.

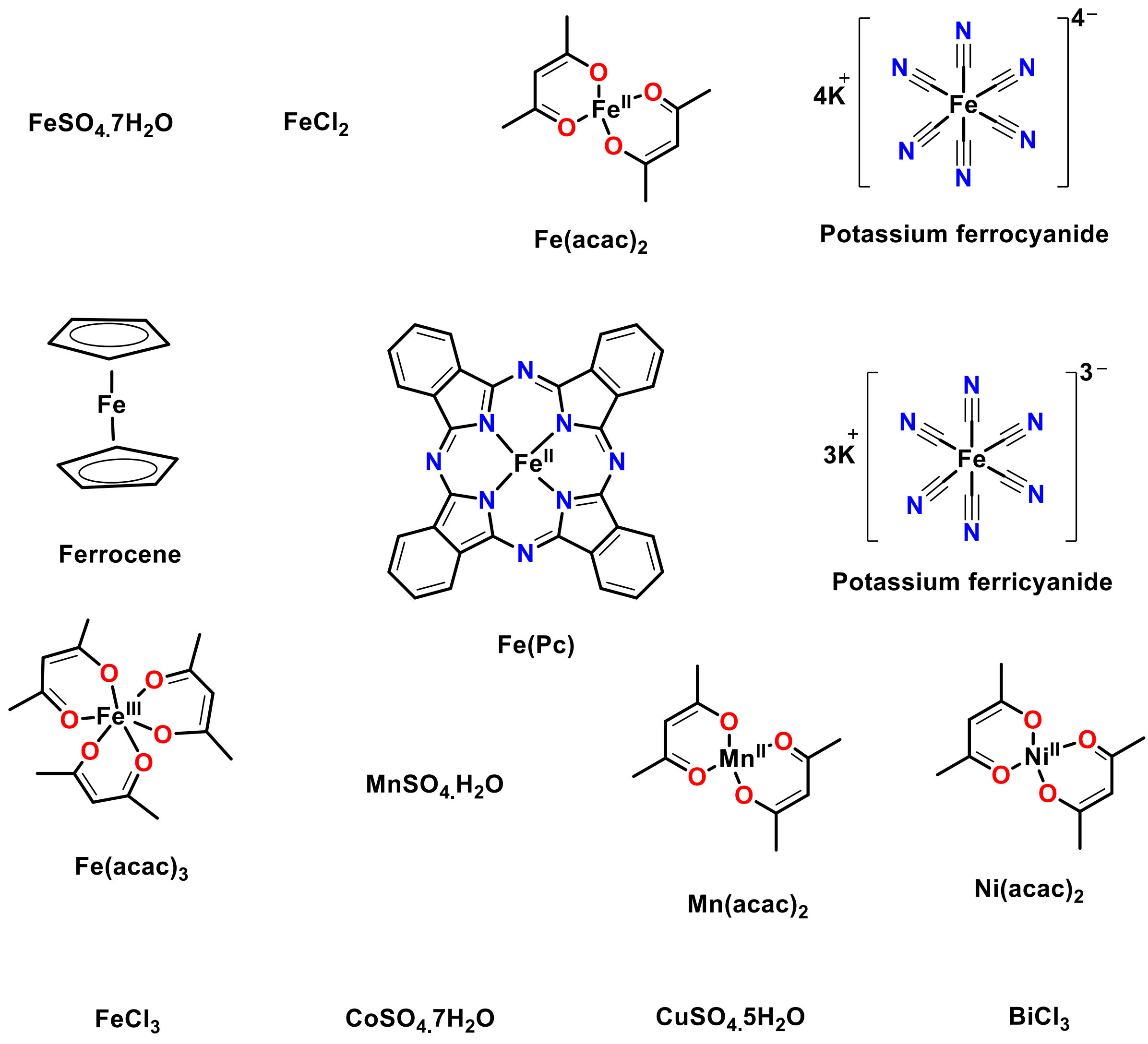

Scheme S4. Catalysts used for screening oxidative deamination of sulfide.

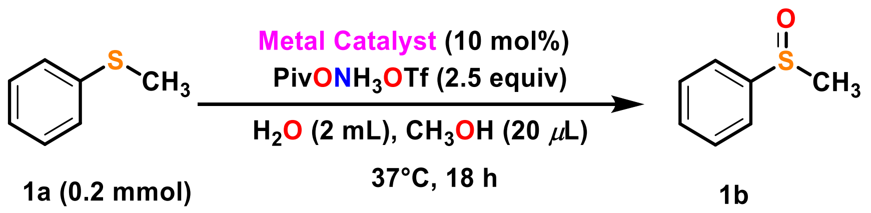

Scheme S5. Choice of metal catalysts for the reaction of Methyl phenyl sulfide and $PivONH_3OTf$ (Ox_1).

Table S1. Screening of different metal catalysts for the oxidative deamination of sulfides.

Entry	Catalyst Selection (0.02 mmol)[a]	Product 1b
1	$FeSO_4.7H_2O$	71%
2	$FeCl_2$	50%
3	$Fe(acac)_2$	59%
4	$K_4[Fe(CN)_6]\cdot 3H_2O$.	50%
5	$[Fe(Cp)_2]$	38%
6	[FePc]	95%
7	$[K_3[Fe(CN)_6]$	46%
8	$Fe(acac)_3$	20%
9	$FeCl_3$	58%
10	$MnSO_4.H_2O$	61%
11	$Mn(acac)_2$	20%
12	$Ni(acac)_2$	13%
13	$CoSO_4.7H_2O$	21%
14	$CuSO_4.5H_2O$	10%
15	$BiCl_3$	20%
16	No catalyst	22%

a) Methyl phenyl sulfide (1a) (23.5 μL, 0.20 mmol) and $PivONH_3OTf$ (133.6 mg, 0.50 mmol).

Optimization of catalyst concentration (II):

A screw-cap vial was charged with $FeSO_4.7H_2O$ (x mol%), and a stirring bar. To it solid oxidant $PivONH_3OTf$ (Ox_1) (133.6 mg, 0.5 mmol, 2.5 equiv.) was added followed by immediate addition of 2.0 mL of H_2O and 20 *μ*L CH_3OH via syringe. Methyl phenyl sulfide,**1a** (23.5 *μ*L, 0.2 mmol) substrate was added to the reaction mixture immediately after this and the reaction vial was sealed and stirred at 37 °C for 18 h. After 18 h the reaction mixture was diluted with saturated $NaHCO_3$ (2 mL) and 1,3,5-trimethoxybenzene (33.6 mg, 0.2 mmol,1 equiv.) w.r.t. the substrate was added as ^1H NMR internal standard and stirred for 20 mins. The water phase was then

extracted with CH_2Cl_2 (3 x 5 mL). The combined organic phases were washed with brine and dried over anhydrous Na_2SO_4, filtered, and concentrated in *vacuo*.

The yield of Methyl phenyl sulfoxide product (1b) was determined by ^{1}H NMR with respect to 1,3,5-trimethoxybenzene as internal standard.

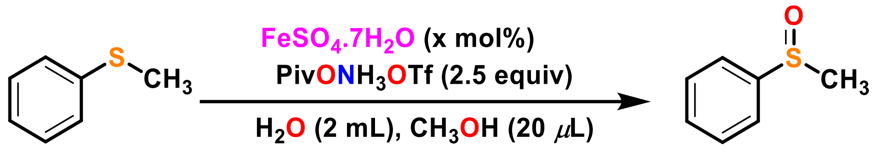

Scheme S6. Varying concentration of catalyst for the reaction of Methyl phenyl sulfide and $PivONH_3OTf$ (Ox_1).

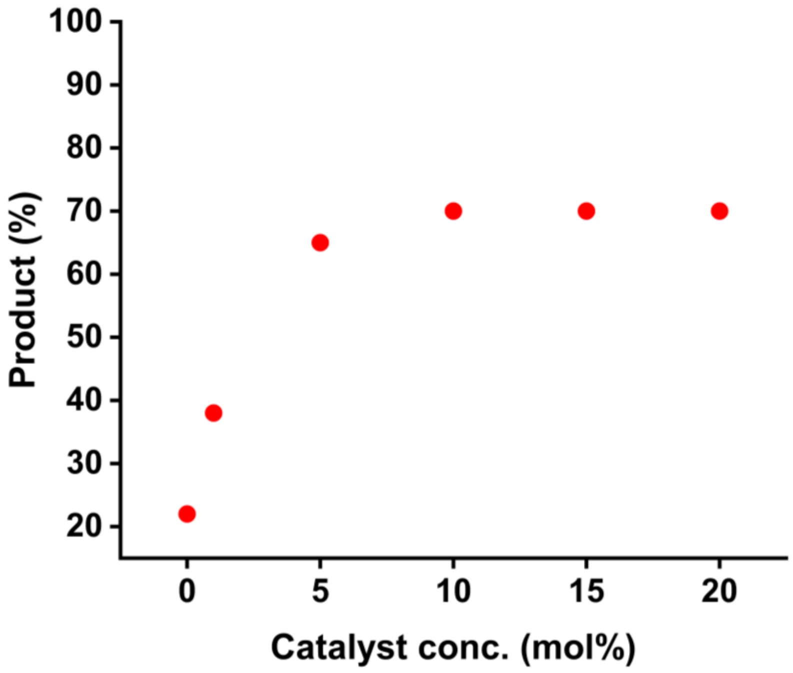

Figure S1. Optimization of catalyst concentration ($FeSO_4.7H_2O$) for oxidative deamination of sulfide by $PivONH_3OTf$ (Ox_1) in water.

Table S2. Optimization of catalyst concentration.

**Entry No.**	**$FeSO_4.7H_2O^a$ (mol%)**	**Product Yield %**
1.	1%	38%
2.	5%	~65%
3.	10%	~ 70%
4.	15%	~70%
5.	20%	~70%

a) Methyl phenyl sulfide (1a) (23.5 μL, 0.20 mmol) and $PivONH_3OTf$ (133.6 mg, 0.50 mmol).

Ligand screening for oxidative deamination of sulfides(III):

A screw-cap vial was charged with $FeSO_4.7H_2O$ (11 mg, 0.04 mmol, 20 mol%), and respective ligands (L) (20 mol%) (Scheme S7, Table S2) along with a stirring bar. To it solid oxidant $PivONH_3OTf$ (Ox_1) (133.6 mg, 0.5 mmol, 2.5 equiv.) was added followed by immediate addition of 2.0 mL of H_2O and 20 μL CH_3OH via syringe. Methyl phenyl sulfide,**1a** (23.5 μL, 0.2 mmol) substrate was added to the reaction mixture immediately after this and the reaction vial was sealed and stirred at 37 °C for 18 h. After 18 h the reaction mixture was diluted with saturated $NaHCO_3$ (2 mL) and 1,3,5-trimethoxybenzene (33.6 mg, 0.2 mmol,1 equiv.) w.r.t. the substrate was added as ^{1}H NMR internal standard and stirred for 20 mins. The water phase was then extracted with CH_2Cl_2 (3 x 5 mL). The combined organic phases were washed with brine and dried over anhydrous Na_2SO_4, filtered, and concentrated in *vacuo*.

The yield of Methyl phenyl sulfoxide product (1b) was determined by ^{1}H NMR with respect to 1,3,5-trimethoxybenzene as internal standard.

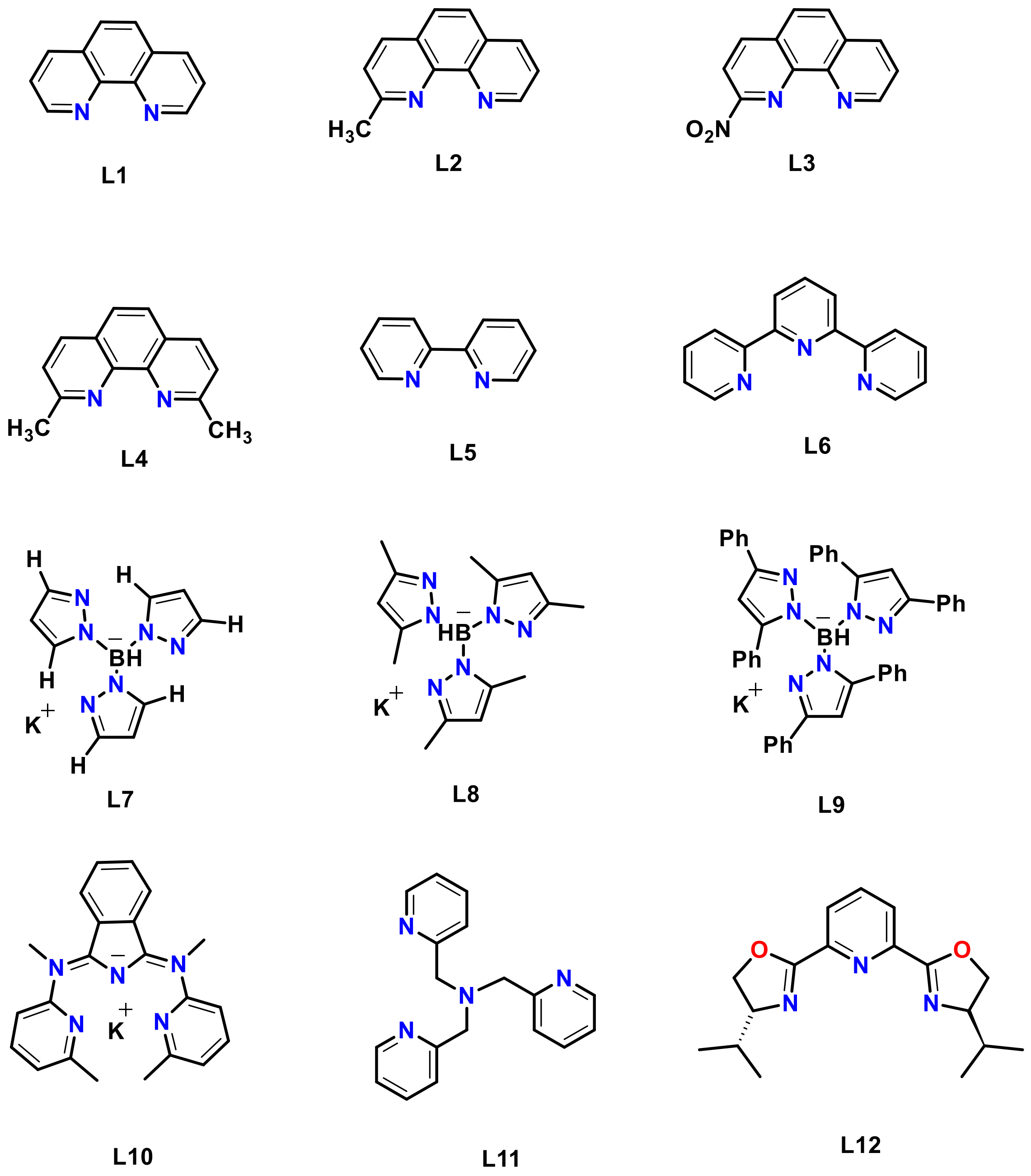

Scheme S7. Different ligands used in the study.

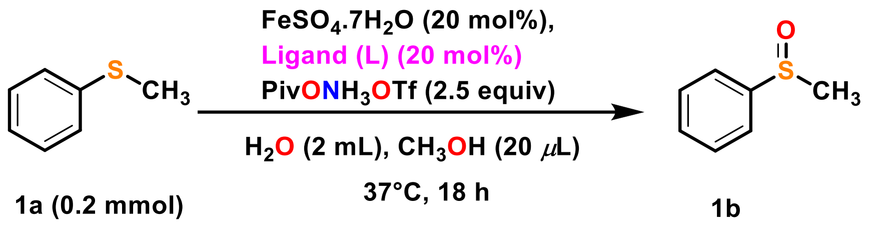

Scheme S8. Choice of ligand for a fixed catalyst for the reaction of Methyl phenyl sulfide and PivONH$_3$OTf (Ox$_1$).

Table S3. Optimization study with different ligands.

**S.no**	**Catalyst[a] (20 mol%)**	**Ligand (20 mol%)**	**Yield %**
1.	$FeSO_4.7H_2O$	L1	98%
2.	$FeSO_4.7H_2O$	L2	98%
3.	$FeSO_4.7H_2O$	L3	76%
4.	$FeSO_4.7H_2O$	L4	51%
5.	$FeSO_4.7H_2O$	L5	98%
6.	$FeSO_4.7H_2O$	L6	97%
7.	$FeSO_4.7H_2O$	L7	55%
8.	$FeSO_4.7H_2O$	L8	44%
9.	$FeSO_4.7H_2O$	L9	60%
10.	$FeSO_4.7H_2O$	L10	75%
11.	$FeSO_4.7H_2O$	L11	90%
12.	$FeSO_4.7H_2O$	L12	62%

a) Methyl phenyl sulfide (1a) (23.5 µL, 0.20 mmol) and PivONH$_3$OTf (133.6 mg, 0.50 mmol).

Optimization of catalyst:ligand loading (IV):

A screw-cap vial was charged with $FeSO_4.7H_2O$ (11 mg, 0.04 mmol, 20 mol%), and ligand (L1) (X mol%) (Scheme S9, Table S3) along with a stirring bar. To it solid oxidant $PivONH_3OTf$ (Ox_1) (133.6 mg, 0.5 mmol, 2.5 equiv.) was added followed by immediate addition of 2.0 mL of H_2O and 20 μL CH_3OH via syringe. Methyl phenyl sulfide,**1a** (23.5 μL, 0.2 mmol) substrate was added to the reaction mixture immediately after this and the reaction vial was sealed and stirred at 37 °C for 18 h. After 18 h the reaction mixture was diluted with saturated $NaHCO_3$ (2 mL) and 1,3,5-trimethoxybenzene (33.6 mg, 0.2 mmol,1 equiv.) w.r.t. the substrate was added as ^1H NMR internal standard and stirred for 20 mins. The water phase was then extracted with CH_2Cl_2 (3 x 5 mL). The combined organic phases were washed with brine and dried over anhydrous Na_2SO_4, filtered, and concentrated in *vacuo*.

The yield of Methyl phenyl sulfoxide product (1b) was determined by ^1H NMR with respect to 1,3,5-trimethoxybenzene as internal standard.

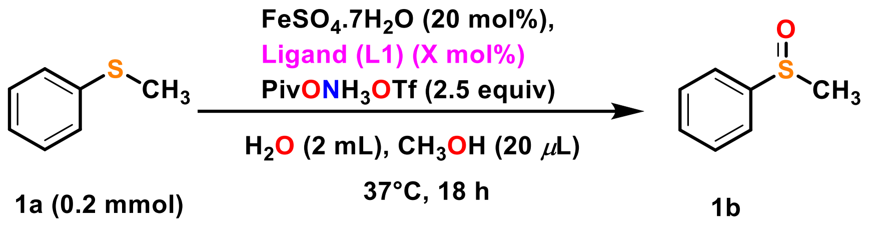

Scheme S9. Catalyst:Ligand loading for the reaction of Methyl phenyl sulfide and $PivONH_3OTf$ (Ox_1).

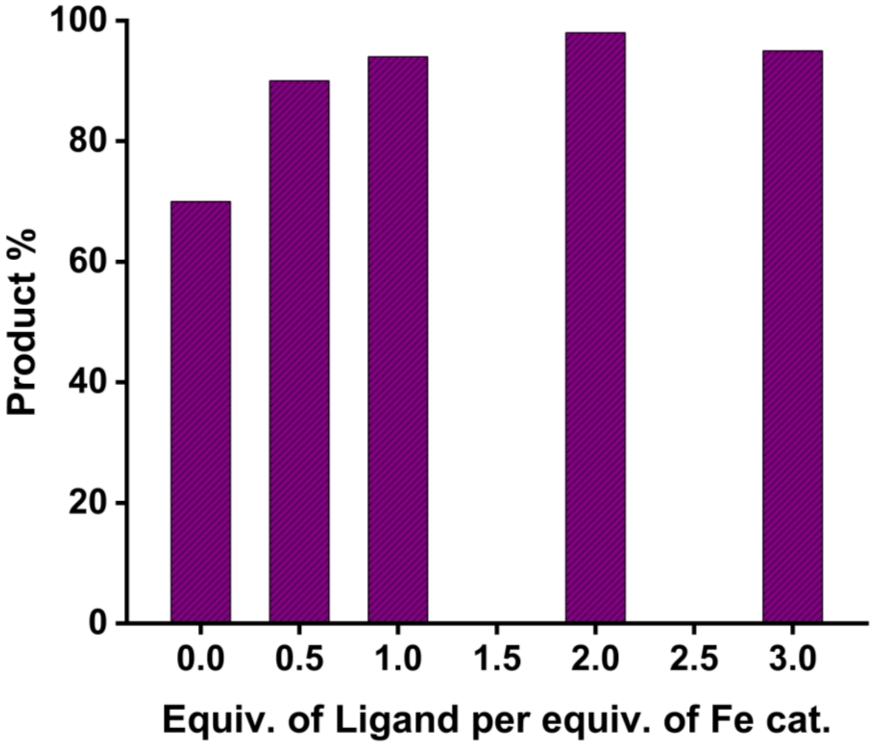

Figure S2. Plot of Catalyst: ligand loading for the reaction of $PivONH_3OTf$ with sulfides in the presence of iron catalyst.

Table S4. Optimization of Catalyst: ligand (L1) loading for the iron catalyzed oxidative deamination of sulfides.

S.no	$FeSO_4.7H_2O$ (mol%)[a]	Ligand (L1) (X mol%)	Yield %
1.	20%	No ligand	70%
2.	20%	10%	90%
3.	20%	20%	94%
4.	20%	40%	98%
5.	20%	60%	95%

a) Methyl phenyl sulfide (1a) (23.5 μL, 0.20 mmol) and $PivONH_3OTf$ (133.6 mg, 0.50 mmol).

Optimization of equivalence of $PivONH_3OTf$ (Ox_1) (V):

A clean screw cap vial was equipped with a magnetic bar in a stirrer. Then $FeSO_4.7H_2O$ (11 mg, 0.04 mmol, 20 mol%) and 1,10-phenanthroline (L1) (15.8 mg, 0.08 mmol, 40 mol%) was added followed by immediate addition of solid oxidant $PivONH_3OTf$ (xx equiv.). To the reaction mixture 2.0 mL of H_2O and 20 μL CH_3OH via syringe followed by immediate addition of Methyl phenyl sulfide (23.5 μL, 0.2 mmol) substrate. The reaction mixture was sealed and stirred for 18 h at 37℃. After 18 h, the reaction mixture was diluted with saturated $NaHCO_3$ (2 mL) and 1,3,5-trimethoxybenzene (33.6 mg, 0.2 mmol,1 equiv.) w.r.t. the substrate was added as ^1H NMR internal standard and stirred for 20 mins. The water phase was then extracted with CH_2Cl_2 (3 x 5 mL). The combined organic phases were washed with brine and dried over anhydrous Na_2SO_4, filtered, and concentrated in *vacuo*.

The yield of Methyl phenyl sulfoxide product (1b) was determined by ^1H NMR with respect to 1,3,5-trimethoxybenzene as internal standard.

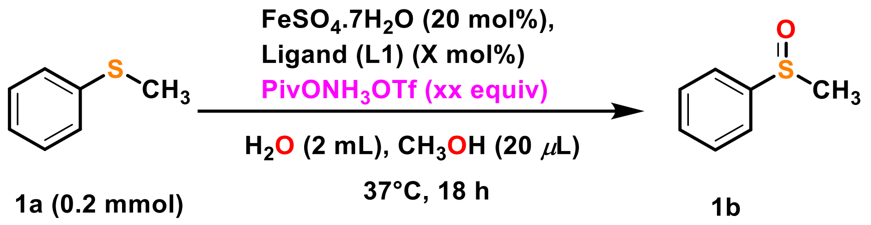

Scheme S10. Varying equivalence of $PivONH_3OTf$ (Ox_1) for the iron catalyzed oxidative deamination of sulfide.

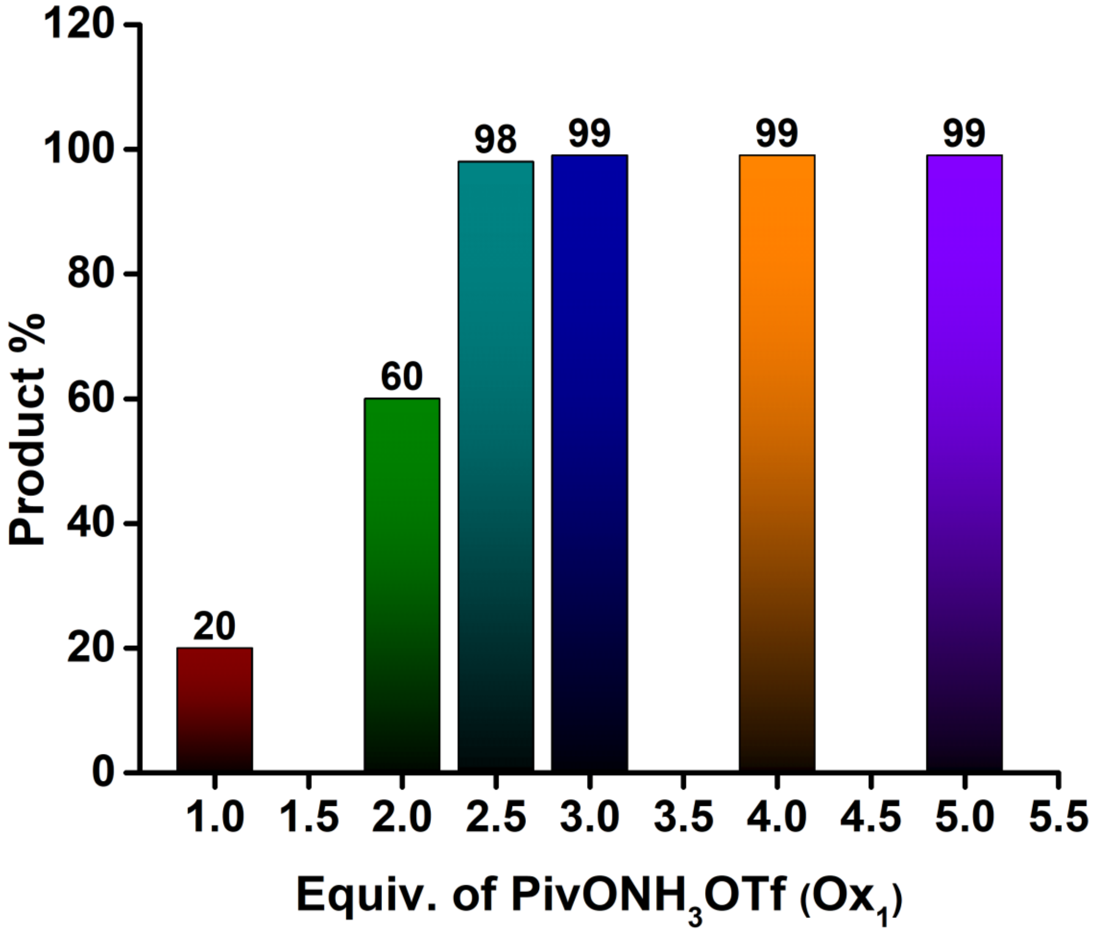

Figure S3. Plot showing product % vs equivalence of $PivONH_3OTf$ (Ox_1) for the iron catalyzed oxidative deamination of sulfide after 18 h.

Table S5. Product % vs equivalence of $PivONH_3OTf$ (Ox_1) for the iron catalysed oxidative deamination of sulfide.

S. No.	Equiv. of $PivONH_3OTf$ (Ox_1) (xx) [a]	Yield %
1.	1	20%
2.	2	60%
3.	2.5	98%
4.	3	99%
5.	4	99%
6.	5	99%

a) $FeSO_4.7H_2O$ (11 mg, 0.04 mmol, 20 mol%) and 1,10-phenanthroline (15.8 mg, 0.08 mmol, 40 mol%) Methyl phenyl sulfide (1a) (23.5 µL, 0.20 mmol) and $PivONH_3OTf$ (xx equiv.).

Optimization of reaction time (VI):

A clean screw cap vial was equipped with a magnetic bar in a stirrer. Then $FeSO_4.7H_2O$ (11 mg, 0.04 mmol, 20 mol%) and 1,10-phenanthroline (15.8 mg, 0.08 mmol, 40 mol%) was added followed by immediate addition of solid oxidant $PivONH_3OTf$ (133.6 mg, 0.5 mmol, 2.5 equiv.). 2.0 mL of H_2O and 20 µL CH_3OH was added via syringe followed by immediate addition of Methyl phenyl sulfide (23.5 µL, 0.2 mmol) substrate The resulting mixture was sealed and stirred for different time intervals (as mentioned in Table S6) at 37 ℃. After the required time period (y min), the reaction mixture was diluted with saturated $NaHCO_3$ (2 mL) and 1,3,5-trimethoxybenzene (33.6 mg, 0.2 mmol,1 equiv. w.r.t. the substrate was added as ^1H NMR internal standard and stirred for 20 mins. The water phase was then extracted with CH_2Cl_2 (3 x 5 mL). The combined organic phases were washed with brine and dried over anhydrous Na_2SO_4, filtered, and concentrated in *vacuo*.

The yield of Methyl phenyl sulfoxide product (1b) was determined by ^1H NMR with respect to 1,3,5-trimethoxybenzene as internal standard.

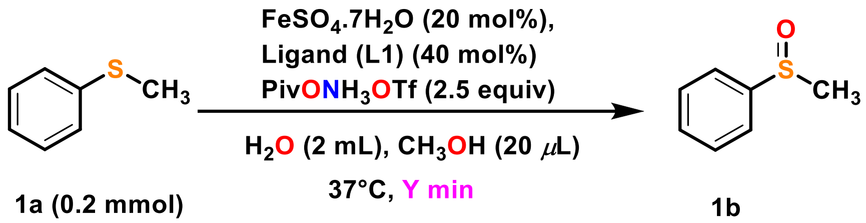

Scheme S11. Plot of time dependent study for the reaction of $PivONH_3OTf$ (Ox_1) with sulfides in the presence of iron catalyst.

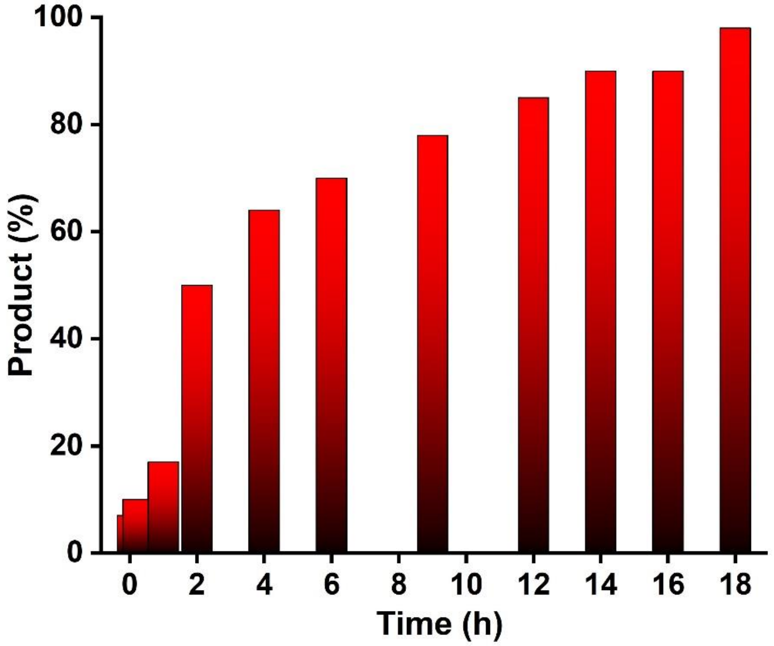

Figure S4. Time dependent study for the reaction of $PivONH_3OTf$ (Ox_1) with sulfides in the presence of iron catalyst.

Table S6. Time dependent study for the reaction of $PivONH_3OTf$ with sulfides in the presence of iron catalyst.

Entry	Time [a]	% Yield
1	5 min	7 %
2	15 min	10%
3	1 h	17%
4	2 h	50%
5	4 h	64%
6	6 h	70%
7	9 h	78%
8	12 h	85%
9	14 h	90%
10	16 h	90%
11	18 h	98%

a) $FeSO_4.7H_2O$ (11 mg, 0.04 mmol, 20 mol%) and 1,10-phenanthroline (15.8 mg, 0.08 mmol, 40 mol%) Methyl phenyl sulfide (1a) (23.5 μL, *0.20 mmol) and $PivONH_3OTf$ (133.6 mg, 0.50 mmol).*

Solvent Screening (VII):

A clean screw cap vial was equipped with a magnetic bar in a stirrer. Then $FeSO_4.7H_2O$ (11 mg, 0.04 mmol, 20 mol%) and 1,10-phenanthroline (L1) (15.8 mg, 0.08 mmol, 40 mol%) was added followed by immediate addition of solid oxidant $PivONH_3OTf$ (133.6 mg, 0.5 mmol, 2.5 equiv.). To the reaction mixture respective solvent/solvent mixtures (Table S7) X mL was added via syringe followed by immediate addition of Methyl phenyl sulfide (23.5 *μ*L, 0.2 mmol) substrate The reaction mixture was sealed and stirred for 18 h at 37 ℃. After 18 h, the reaction mixture was diluted with saturated $NaHCO_3$ (2 mL) and 1,3,5-trimethoxybenzene (33.6 mg, 0.2 mmol,1 equiv. w.r.t. the substrate was added as ^1H NMR internal standard and stirred for 20 mins. The water phase was then extracted with CH_2Cl_2 (3 x 5 mL). The combined organic phases were washed with brine and dried over anhydrous Na_2SO_4, filtered, and concentrated in *vacuo*.

The yield of Methyl phenyl sulfoxide product (1b) was determined by ^{1}H NMR with respect to 1,3,5-trimethoxybenzene as internal standard.

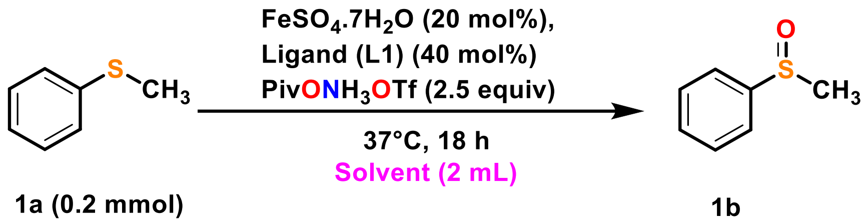

Scheme S12. Screening different solvent for the iron catalysed oxidative deamination of sulfides by the oxidant $PivONH_3OTf$ (Ox_1).

Table S7. Screening different solvent for the iron catalysed oxidative deamination of sulfides by the oxidant $PivONH_3OTf$ (Ox_1).

Entry	Solvent[a]	% Yield
1.	CH_3CN	8%
2.	CH_2Cl_2	26%
3.	CH_3OH	58%
4.	1,4 Dioxane	trace
5.	HFIP	trace
6.	H_2O	95%

a) $FeSO_4.7H_2O$ (11 mg, 0.04 mmol, 20 mol%) and 1,10-phenanthroline (15.8 mg, 0.08 mmol, 40 mol%) Methyl phenyl sulfide (1a) (23.5 µL, 0.20 mmol) and $PivONH_3OTf$ (133.6 mg, 0.50 mmol). Solvents were used without drying.

Screening in Water/Organic Solvent mixture (VIII):

A clean screw cap vial was equipped with a magnetic bar in a stirrer. Then $FeSO_4.7H_2O$ (11 mg, 0.04 mmol, 20 mol%) and 1,10-phenanthroline (L1) (15.8 mg, 0.08 mmol, 40 mol%) was added followed by immediate addition of solid oxidant $PivONH_3OTf$ (133.6 mg, 0.5 mmol, 2.5 equiv.). To the reaction mixture water: organic solvent mixtures (x:y) (Table S8) was added via syringe followed by immediate addition of Methyl phenyl sulfide (23.5 μL, 0.2 mmol) substrate The reaction mixture was sealed and stirred for 18 h at 37℃. After 18 h, the reaction mixture was diluted with saturated $NaHCO_3$ (2 mL) and 1,3,5-trimethoxybenzene (33.6 mg, 0.2 mmol,1 equiv. w.r.t. the substrate was added as ^1H NMR internal standard and stirred for 20 mins. The water phase was then extracted with CH_2Cl_2 (3 x 5 mL). The combined organic phases were washed with brine and dried over anhydrous Na_2SO_4, filtered, and concentrated in *vacuo*.

The yield of Methyl phenyl sulfoxide product (1b) was determined by ^1H NMR with respect to 1,3,5-trimethoxybenzene as internal standard.

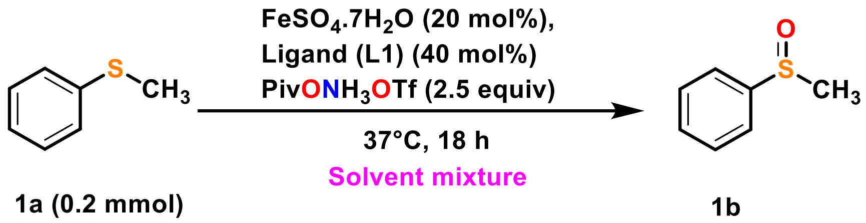

Scheme S13. Screening water: organic solvent ratio for the iron catalysed oxidative deamination of sulfides by the oxidant $PivONH_3OTf$ (Ox_1).

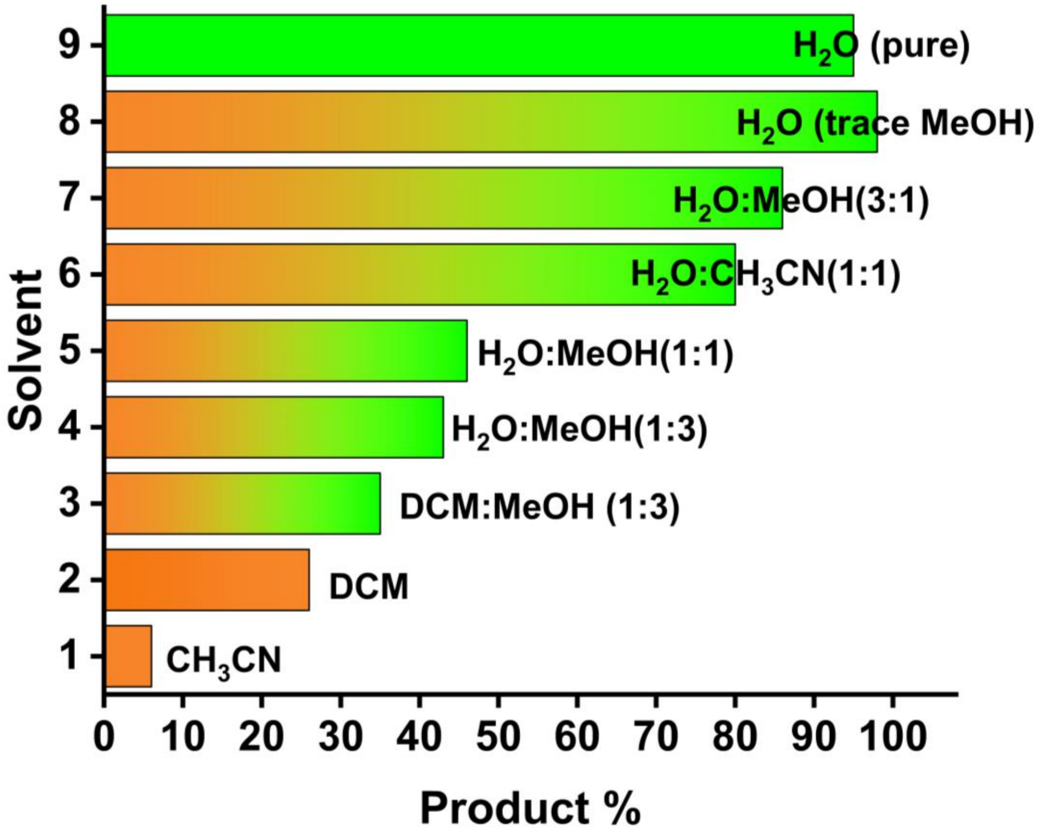

Figure S5. Screening water: organic solvent ratio for the iron catalysed oxidative deamination of sulfides by the oxidant $PivONH_3OTf$ (Ox_1).

Table S8. Screening water: organic solvent ratio for the iron catalysed oxidative deamination of sulfides by the oxidant $PivONH_3OTf$ (Ox_1).

**Entry**	**Solvent mixture** [a]	**% Yield**
1.	CH_3CN	8%
2.	CH_2Cl_2	26%
3.	CH_2Cl_2:CH_3OH (1:3)	35%
4.	H_2O: CH_3OH (1:3)	43%
5	H_2O: CH_3OH (1:1)	46%
**6**	H_2O: CH_3CN (1:1)	80%
7	H_2O: CH_3OH (3:1)	86%
8	H_2O:CH_3OH (99:1)	98%
**9**	H_2O: CH_3CN (99:1)	93%
10	H_2O	95%

a) *$FeSO_4.7H_2O$ (11 mg, 0.04 mmol, 20 mol%) and 1,10-phenanthroline (15.8 mg, 0.08 mmol, 40 mol%) Methyl phenyl sulfide (1a)* (23.5 μL*, 0.20 mmol) and $PivONH_3OTf$ (133.6 mg, 0.50 mmol).*

Analyses of the reaction profile by HRMS and GC-MS :

A clean screw cap vial was equipped with a magnetic bar in a stirrer. Then catalyst, ligand (L), solid oxidant $PivONH_3OTf$ as shown in respective entries of Table S9 was added in 2.0 mL of H_2O. Then 20 μL CH_3OH were added via syringe followed by immediate addition of Methyl phenyl sulfide (23.5 μL, 0.2 mmol) substrate. The resulting mixture was sealed and stirred for different time intervals (as mentioned in Table S9) at 37 ℃. After the required time period (n h), the reaction mixture was analyzed by mass spectrometry.

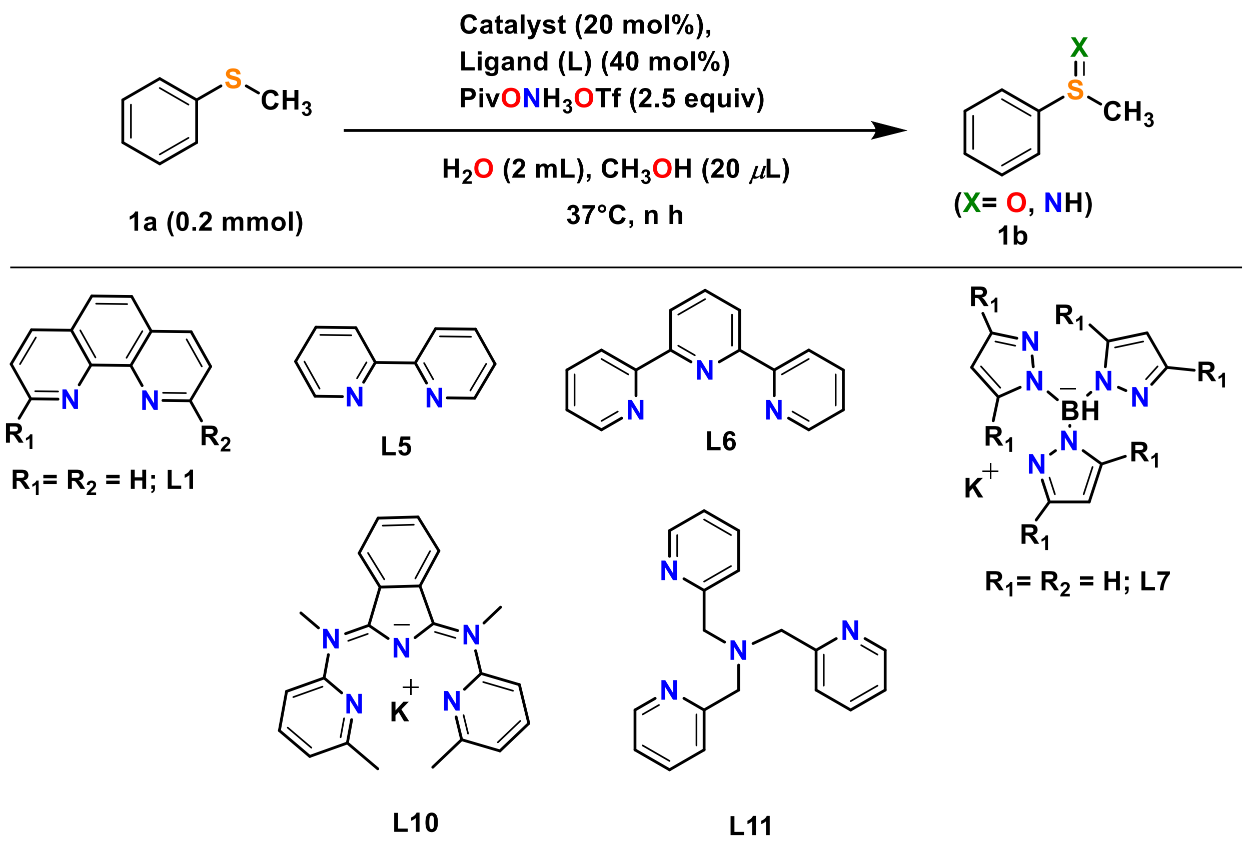

Scheme S14. Screening reaction mixture of the iron catalyzed sulfide oxidation/imination under different reaction conditions to track the relative ratio of sulfoxide (X = O) vs sulfilimine (X =NH) in water.

Table S9. Analyses of the reaction profile by HRMS AND GC-MS.

Entry	Deviation from standard conditions	Product 1b[b]	15 min		3 h		18 h	
			NH	O	NH	O	NH	O
1	No deviation from standard condition	98%	85%	15%	50%	50%	<1%	>99%
2	$Fe(acac)_2$, no L1	59%	100%	0%	90%	10%	<1%	>99%
3	$FeCl_2$, no L1	50%	72%	28%	30%	60%	<1%	>99%
4	$FeSO_4.7H_2O$, no L1	70%	91%	9%	81%	19%	<1%	>99%
5	FePc, no L1	95%	81%	19%	63%	37%	<1%	>99%
6	L5 instead of L1	98%	82%	18%	73%	27%	2%	>98%
7	L6 instead of L1	97%	83%	17%	78%	22%	<2%	>98%
8	L7 instead of L1	55%	84%	16%	84%	16%	70%	30%
9	L10 instead of L1	75%	90%	10%	89%	11%	51%	49%
10	L11 instead of L1	90%	84%	16%	80%	20%	12%	78%
11	$Fe(acac)_3$, no L1	20%	96%	4%	87%	13%	<2%	>98%
12	H_2O as solvent	95%	84%	16%	76%	24%	<2%	>98%
13	CH_2Cl_2 as solvent	26%	88%	12%	84%	16%	72%	28%
14	1 equiv. of $PivONH_3OTf$	20%	85%	15%	73%	27%	<2%	>98%
15	No catalyst	22%	93%	7%	65%	45%	40%	60%
16	Under Argon	95%	89%	11%	84%	16%	34%	66%

a) *$FeSO_4.7H_2O$ (11 mg, 0.04 mmol, 20 mol%) and L (40 mol%), Methyl phenyl sulfide (1a)* (23.5 µL, *0.20 mmol) and $PivONH_3OTf$ (133.6 mg, 0.50 mmol).*

b) *Corresponding NMR yield.*

Synthesis procedure for isolated iron(II) catalysts(IX):

a) Synthesis of iron(II) catalyst derived from L1 (1, 10 phenanthroline)

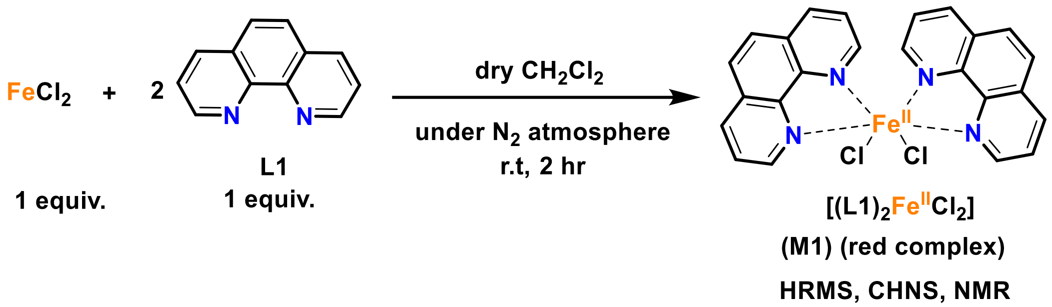

Scheme S15. Synthesis of [$(L1)_2Fe(Cl_2)$] Complex (M1).

1,10-phenanthroline (L1) (396 mg, 2 mmol) and anhydrous $FeCl_2$ (254 mg, 2 mmol) were taken in a in a round bottomed flask under N_2 atmosphere. To it dry degassed CH_2Cl_2 (10 mL) was added when a red coloration was formed. The red solution was stirred at room temperature for 2 h under N_2 atmosphere. The solvent was removed under reduced pressure, the residue was filtered, washed with diethyl ether (5–10 mL), and dried in high vacuum to provide the iron (II) complex as a red solid (200 mg, 28%). The composition of the complex was confirmed by elemental analysis calcd (%) for $C_{24}H_{16}Cl_2FeN_4.(CH_2Cl_2)_2(H_2O)_2$ (693.04 g/mol): Theoretical: C, 45.06; H, 3.49; N, 8.08. Found: C, 44.15; H, 3.089; N, 8.16 as well as HR-MS(ESI$^+$) calc. for $C_{24}H_{16}Cl_2FeN_4$ $[M+H]^+$ 486.0101, found 486.0089.

Catalytic study with isolated catalyst M1(X):

A clean screw cap vial was equipped with a magnetic bar in a stirrer. Then catalyst [(L1)$FeCl_2$] (XX mol%) was added followed by immediate addition of solid oxidant $PivONH_3OTf$ (133.6 mg, 0.5 mmol, 2.5 equiv.). 2.0 mL of H_2O and 20 μL CH_3OH was added via syringe followed by immediate addition of Methyl phenyl sulfide (23.5 μL, 0.2 mmol) substrate The resulting mixture was sealed and stirred for 18 hr at 37℃. After 18 hr, the reaction mixture was diluted with saturated $NaHCO_3$ (2 mL) and 1,3,5-trimethoxybenzene (33.6 mg, 0.2 mmol,1 equiv.) w.r.t. the substrate was added as ^1H NMR internal standard and stirred for 20 mins. The water phase was then extracted with CH_2Cl_2 (3 x 5 mL). The combined organic phases were washed with brine and dried over anhydrous Na_2SO_4, filtered, and concentrated in *vacuo*.

The yield of Methyl phenyl sulfoxide product (1b) was determined by ^1H NMR with respect to 1,3,5-trimethoxybenzene as internal standard.

Table S10. Oxidative deamination of sulfides in the presence of catalyst [(L1)FeCl$_2$] (M1)

Entry	Catalyst	Catalyst conc.(XX mol%)[a]	Product (1b) %
1.	**[(L1)$_2$FeCl$_2$] (M1)**	10%	82%
2.	**[(L1)$_2$FeCl$_2$] (M1)**	20%	>95%

a) Methyl phenyl sulfide (1a) (23.5 µL, 0.20 mmol) and PivONH$_3$OTf (133.6 mg, 0.50 mmol).

Control experiments (XI):

1) Sulfide oxidation under standard optimized condition in the absence of iron catalyst:

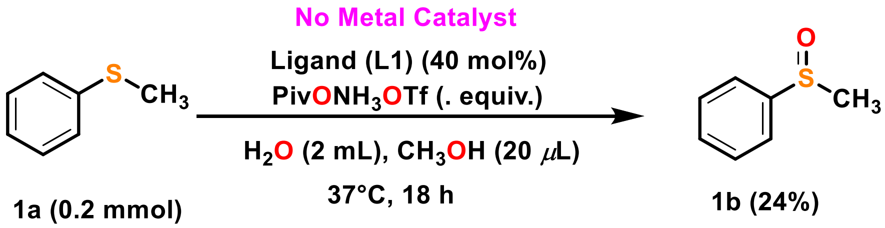

Scheme S16. Sulfide oxidation by oxidant PivONH$_3$OTf under standard condition in the absence of any metal catalyst.

A clean screw cap vial was equipped with a magnetic bar in a stirrer. To it ligand 1,10-phenanthroline (L1) (15.8 mg, 0.08 mmol, 40 mol%) was added followed by immediate addition of solid oxidant PivONH$_3$OTf (Ox$_1$) (133.6 mg, 0.5 mmol, 2.5 equiv.). 2.0 mL of H_2O and 20 µL CH_3OH was added via syringe followed by immediate addition of Methyl phenyl sulfide (23.5 µL, 0.2 mmol) substrate The resulting mixture was sealed and stirred for 18 h at 37 ℃. After 18 h, the reaction mixture was diluted with saturated $NaHCO_3$ (2 mL) and 1,3,5-trimethoxybenzene (33.6 mg, 0.2 mmol,1 equiv.) w.r.t. the substrate was added as ^{1}H NMR internal standard and stirred for 20 mins. The water phase was then extracted with CH_2Cl_2 (3 x 5 mL). The combined organic phases were washed with brine and dried over anhydrous Na_2SO_4, filtered, and concentrated in *vacuo*.

The yield of Methyl phenyl sulfoxide product (1b) was determined by ^{1}H NMR with respect to 1,3,5-trimethoxybenzene as internal standard, which revealed significantly low amount of product formation was observed in the absence of metal catalyst.

2) Sulfide oxidation under standard optimized condition in the absence of ligand L1:

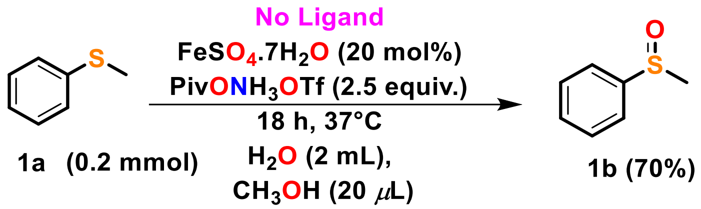

Scheme S17. Sulfide oxidation by oxidant $PivONH_3OTf$ under standard condition catalyzed by iron catalyst in the absence of any added ligand.

A clean screw cap vial was equipped with a magnetic bar in a stirrer. Then $FeSO_4.7H_2O$ (11 mg, 0.04 mmol, 20 mol%) was added followed by immediate addition of solid oxidant $PivONH_3OTf$ (Ox_1) (133.6 mg, 0.5 mmol, 2.5 equiv.). 2.0 mL of H_2O and 20 μL CH_3OH was added via syringe followed by immediate addition of Methyl phenyl sulfide (23.5 μL, 0.2 mmol) substrate. The resulting mixture was sealed and stirred for 18 h at 37℃. After 18 h, the reaction mixture was diluted with saturated $NaHCO_3$ (2 mL) and 1,3,5-trimethoxybenzene (33.6 mg, 0.2 mmol,1 equiv.) w.r.t. the substrate was added as ^1H NMR internal standard and stirred for 20 mins. The water phase was then extracted with CH_2Cl_2 (3 x 5 mL). The combined organic phases were washed with brine and dried over anhydrous Na_2SO_4, filtered, and concentrated in *vacuo*.

The yield of Methyl phenyl sulfoxide product (1b) was determined by ^1H NMR with respect to 1,3,5-trimethoxybenzene as internal standard.

3) Sulfide oxidation under standard optimized condition in the absence of metal catalyst and ligand:

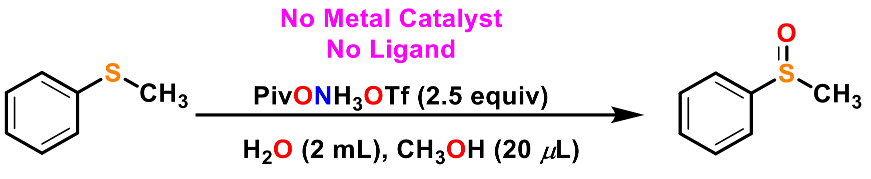

1a (0.2 mmol) 1b (22%)

Scheme S18. Sulfide oxidation by oxidant $PivONH_3OTf$ (Ox_1) under standard condition in the absence of any metal catalyst and ligand.

A clean screw cap vial was equipped with a magnetic bar in a stirrer. Then solid oxidant $PivONH_3OTf$ (Ox_1) (133.6 mg, 0.5 mmol, 2.5 equiv.) was added. 2.0 mL of H_2O and 20 μL CH_3OH was added via syringe followed by immediate addition of Methyl phenyl sulfide (23.5 μL, 0.2 mmol) substrate The resulting mixture was sealed and stirred for 18 h at 37 ℃. After 18 h, the reaction mixture was diluted with saturated $NaHCO_3$ (2 mL) and 1,3,5-trimethoxybenzene (33.6 mg, 0.2 mmol,1 equiv.) w.r.t. the substrate was added as ^{1}H NMR internal standard and stirred for 20 mins. The water phase was then extracted with CH_2Cl_2 (3 x 5 mL). The combined organic phases were washed with brine and dried over anhydrous Na_2SO_4, filtered, and concentrated in *vacuo*. The yield of Methyl phenyl sulfoxide product (1b) was determined by ^{1}H NMR with respect to 1,3,5-trimethoxybenzene as internal standard.

4) Sulfide oxidation under standard optimized condition in the absence of oxidant $PivONH_3OTf$ (Ox_1):

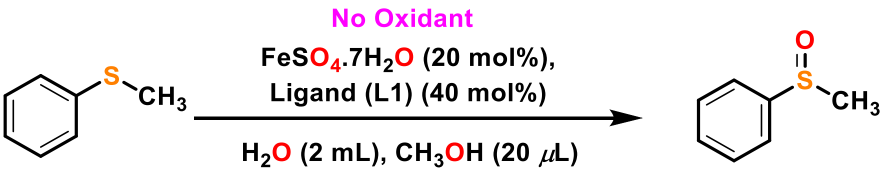

1a (0.2 mmol) 1b (not detected)

Scheme S19. Reaction of Sulfide in the absence of any oxidant under standard condition in the presence of iron catalyst and ligand.

A clean screw cap vial was equipped with a magnetic bar in a stirrer. Then $FeSO_4.7H_2O$ (11 mg, 0.04 mmol, 20 mol%) and ligand 1,10-phenanthroline (L1) (15.8 mg, 0.08 mmol, 40 mol%) was added followed by 2.0 mL of H_2O and 20 μL CH_3OH added via syringe. To the reaction vessel Methyl phenyl sulfide (23.5 μL, 0.2 mmol) substrate was added. The resulting mixture was sealed and stirred for 18 h at 37℃. After 18 h, the reaction mixture was diluted with saturated $NaHCO_3$ (2 mL) and 1,3,5-trimethoxybenzene (33.6 mg, 0.2 mmol,1 equiv.) w.r.t. the substrate was added as ^{1}H NMR internal standard and stirred for 20 mins. The water phase was then extracted with CH_2Cl_2 (3 x 5 mL). The combined organic phases were washed with brine and dried over anhydrous Na_2SO_4, filtered, and concentrated in *vacuo*. Product Methyl phenyl sulfoxide (1b) was not detected in the ^{1}H NMR.

5) Using Hydroxylamine hydrochloride ($NH_2OH.HCl$) instead of $PivONH_3OTf$ under standard reaction condition.

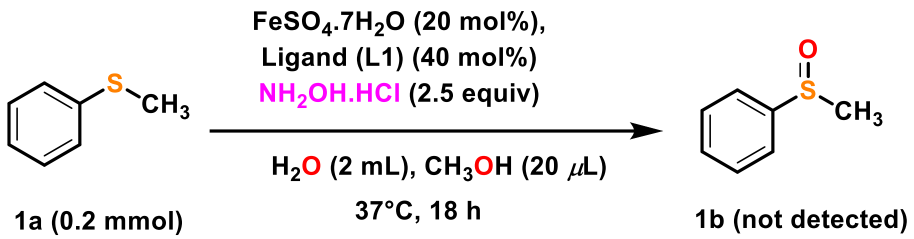

Scheme S20. Reaction of sulfide in the presence of $NH_2OH.HCl$ instead of $PivONH_3OTf$ under standard reaction condition.

A clean screw cap vial was equipped with a magnetic bar in a stirrer. Then $FeSO_4.7H_2O$ (11 mg, 0.04 mmol, 20 mol%) and 1,10-phenanthroline (L1) (15.8 mg, 0.08 mmol, 40 mol%) was added followed by immediate addition of solid hydroxylamine hydrochloride ($NH_2OH.HCl$) (34.74 mg, 0.5 mmol, 2.5 equiv.) (133.6 mg, 0.5 mmol, 2.5 equiv.). 2.0 mL of H_2O and 20 μL CH_3OH was added via syringe followed by addition of Methyl phenyl sulfide (23.5 μL, 0.2 mmol) substrate. The resulting mixture was sealed and stirred for 18 h at 37℃. After 18 h, the reaction mixture was diluted with saturated $NaHCO_3$ (2 mL) and 1,3,5-trimethoxybenzene (33.6 mg, 0.2 mmol,1 equiv.) w.r.t. the substrate was added as ^{1}H NMR internal standard and stirred for 20 mins. The water phase was then extracted with CH_2Cl_2 (3 x 5 mL). The combined organic phases were washed with brine and dried over anhydrous Na_2SO_4, filtered, and concentrated in *vacuo*. Product Methyl phenyl sulfoxide (1b) was not detected in the ^{1}H NMR.

6) Using *N*-Boc protected pivaloyl hydroxylamine (*N*-Boc Pivaloyloxycarbamate) instead of $PivONH_3OTf$ under standard reaction condition.

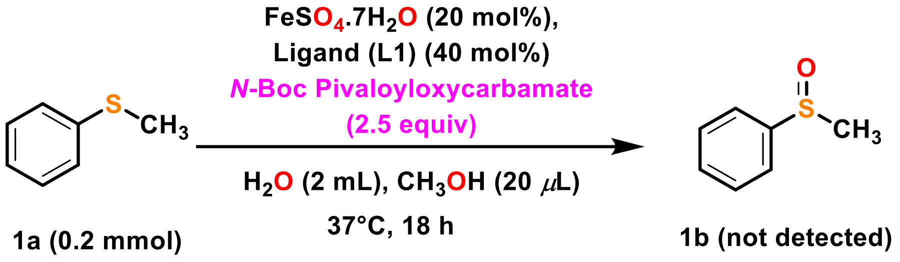

Scheme S21. Reaction of sulfide in the presence of *N*-Boc Pivaloyloxycarbamate instead of $PivONH_3OTf$ under standard reaction condition.

A clean screw cap vial was equipped with a magnetic bar in a stirrer. Then $FeSO_4.7H_2O$ (11 mg, 0.04 mmol, 20 mol%) and 1,10-phenanthroline (15.8 mg, 0.08 mmol, 40 mol%) was added followed by immediate addition of *N*-Boc Pivaloyloxycarbamate (108.5 mg, 0.5 mmol, 2.5 equiv.) 2.0 mL of H_2O and 20 μL CH_3OH was added via syringe followed by addition of Methyl phenyl sulfide (23.5 μL, 0.2 mmol) substrate. The resulting mixture was sealed and stirred for 18 h at 37 ℃. After 18 h, the reaction mixture was diluted with saturated $NaHCO_3$ (2 mL) and 1,3,5-trimethoxybenzene (33.6 mg, 0.2 mmol,1 equiv.) w.r.t. the substrate was added as ^{1}H NMR internal standard and stirred for 20 mins. The water phase was then extracted with CH_2Cl_2 (3 x 5 mL). The combined organic phases were washed with brine and dried over anhydrous Na_2SO_4, filtered, and concentrated in *vacuo*. Product Methyl phenyl sulfoxide (1b) was not detected in the ^{1}H NMR.

7) In situ deprotection of *N*-Boc Pivaloyloxycarbamate

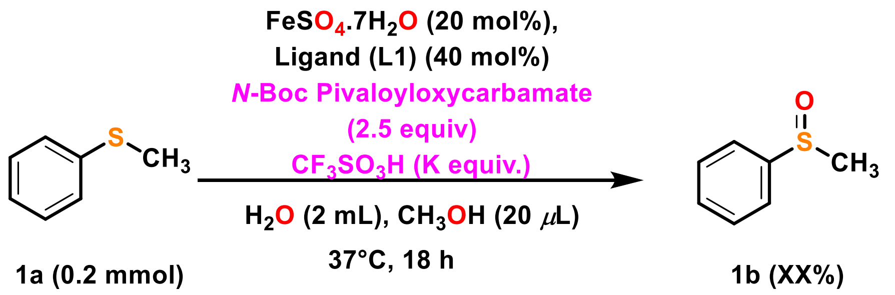

Scheme S22. Reaction of sulfide in the presence of *N*-Boc Pivaloyloxycarbamate and triflic acid for insitu deprotection to generate $PivONH_3OTf$ under standard reaction condition.

A clean screw cap vial was equipped with a magnetic bar in a stirrer. Then $FeSO_4.7H_2O$ (11 mg, 0.04 mmol, 20 mol%) and 1,10-phenanthroline (15.8 mg, 0.08 mmol, 40 mol%) was added followed by immediate addition of *N*-Boc Pivaloyloxycarbamate (108.5 mg, 0.5 mmol, 2.5 equiv.) and varying amount of triflic acid (CF_3SO_3H) (K equiv.). 2.0 mL of H_2O and 20 μL CH_3OH was added via syringe followed by addition of Methyl phenyl sulfide (23.5 μL, 0.2 mmol) substrate. The resulting mixture was sealed and stirred for 18 h at 37 ℃. After 18 h, the reaction mixture was diluted with saturated $NaHCO_3$ (2 mL) and 1,3,5-trimethoxybenzene (33.6 mg, 0.2 mmol,1 equiv.) w.r.t. the substrate was added as ^{1}H NMR internal standard and stirred for 20 mins. The water phase was then extracted with CH_2Cl_2 (3 x 5 mL). The combined organic phases were washed with brine and dried over anhydrous Na_2SO_4, filtered, and concentrated in *vacuo*. Product Methyl phenyl sulfoxide (1b) was not detected in the ^{1}H NMR.

Table S11. In situ deprotection of *N*-Boc Pivaloyloxycarbamate iron catalysed reaction with sulphides.

**Entry**	**Equivalence of *N*-Boc Pivaloyloxycarbamate**[a]	**Equivalence of Triflic acid (CF_3SO_3H) (K equiv)**	**Product (1b) (XX %)**
1.	2.5	0	Not detected
2.	2.5	1.5	33%
3.	2.5	3	83%

a) $FeSO_4.7H_2O$ (11 mg, 0.04 mmol, 20 mol%) and 1,10-phenanthroline (L1) (15.8 mg, 0.08 mmol, 40 mol%) Methyl phenyl sulfide (1a) (23.5 μL, 0.20 mmol), N-Boc pivaloyloxy carbamate (2.5 equiv. w.r.t. 1a) and triflic acid (K equiv.w.r.t. 1a).

8) Reaction with NaN_3 instead of $PivONH_3OTf$

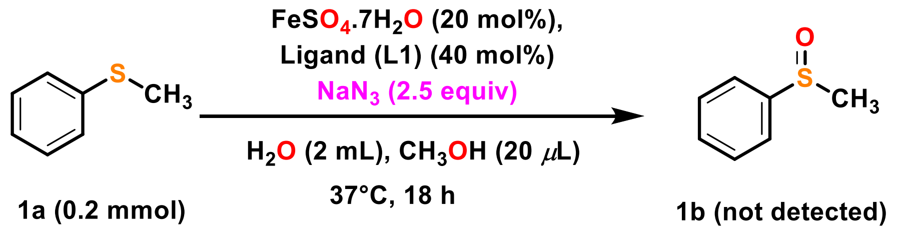

Scheme S23. Reaction of sulfide in the presence of NaN_3 *instead of* $PivONH_3OTf$ in the presence of iron catalyst and ligand under standard reaction condition.

A clean screw cap vial was equipped with a magnetic bar in a stirrer. Then $FeSO_4.7H_2O$ (11 mg, 0.04 mmol, 20 mol%) and 1,10-phenanthroline (15.8 mg, 0.08 mmol, 40 mol%) was added followed by immediate addition of NaN_3 (32.50 mg, 0.5 mmol, 2.5 equiv.). 2.0 mL of H_2O and 20 μL CH_3OH was added via syringe followed by addition of Methyl phenyl sulfide (23.5 μL, 0.2 mmol) substrate. The resulting mixture was sealed and stirred for 18 h at 37℃. After 18 h, the reaction mixture was diluted with saturated $NaHCO_3$ (2 mL) and 1,3,5-trimethoxybenzene (33.6 mg, 0.2 mmol,1 equiv.) w.r.t. the substrate was added as ^{1}H NMR internal standard and stirred for 20 mins. The water phase was then extracted with CH_2Cl_2 (3 x 5 mL). The combined organic phases were washed with brine and dried over anhydrous Na_2SO_4, filtered, and concentrated in *vacuo*.

9) Fate of Oxidant $PivONH_3OTf$ (Ox_1) in water

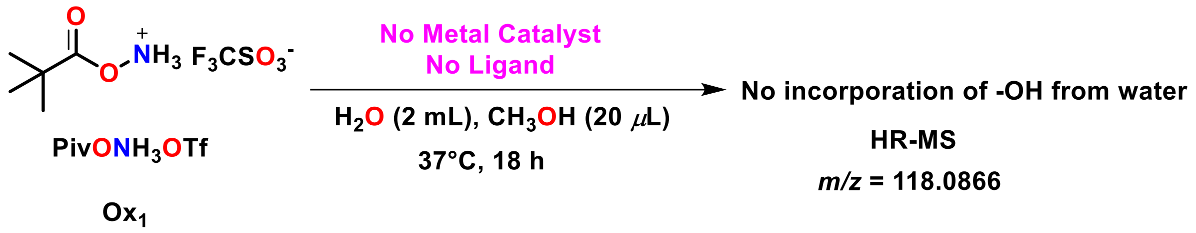

Scheme S24. Fate of oxidant PivONH3OTf (Ox1).

A clean screw cap vial was equipped with a magnetic bar in a stirrer. Then solid oxidant $PivONH_3OTf$ (Ox_1) (133.6 mg, 0.5 mmol, 2.5 equiv.) was added. 2.0 mL of H_2O and 20 μL CH_3OH was added via syringe and resulting mixture was sealed and stirred for 18 h at 37℃. After 18 h, the reaction mixture was submitted for HRMS, which suggests no exchange of NH_3^+ to OH and the reagent remains intact.

HR-MS(ESI^+) calc. for ($PivONH_3^+$) i.e $M^+ = [C_5H_{12}NO_2]^+$ is 118.0874, found 118.0866.

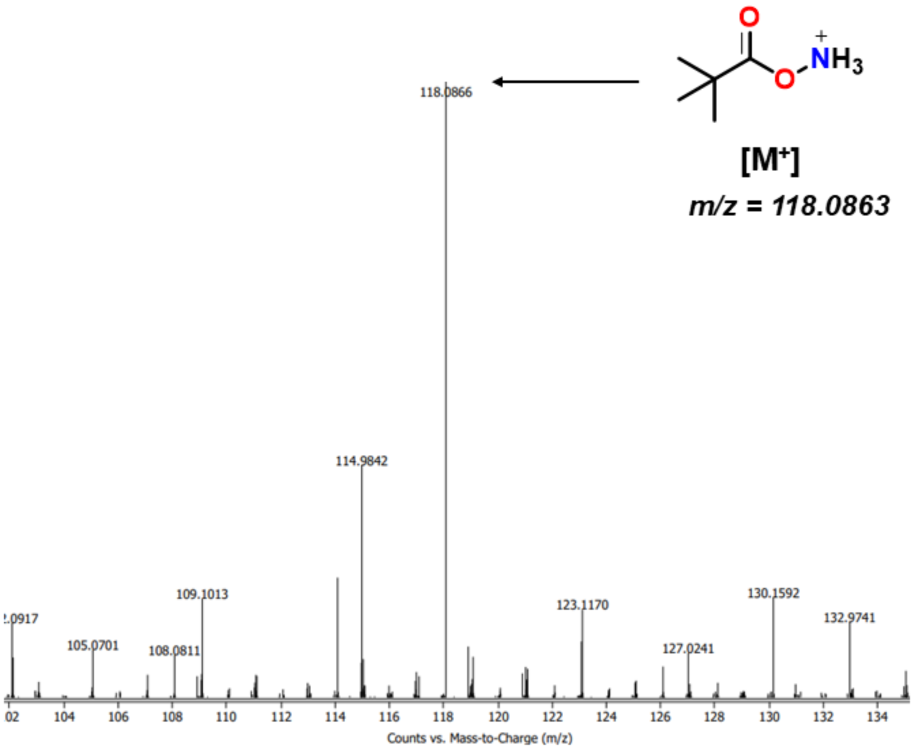

Figure S6. HRMS ESI positive mode of the Oxidant $PivONH_3OTf$ (Ox_1) in CH_3OH.

10) Reaction of Thiol instead of Methyl phenyl sulphide under standard reaction condition

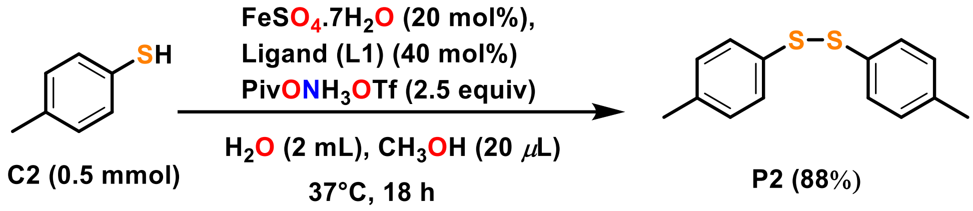

Scheme S25. Reaction of thiol instead of disulphide under standard reaction condition.

A clean screw cap vial was equipped with a magnetic bar in a stirrer. Then $FeSO_4.7H_2O$ (11 mg, 0.04 mmol, 20 mol%) and 1,10-phenanthroline (15.8 mg, 0.08 mmol, 40 mol%) was added followed by immediate addition of solid oxidant $PivONH_3OTf$ (Ox_1) (133.6 mg, 0.5 mmol, 2.5 equiv.). 2.0 mL of H_2O and 20 μL CH_3OH was added via syringe followed by immediate addition of 4-Methyl benzene thiol (24.8 mg, 0.2 mmol) substrate. The resulting mixture was sealed and stirred for 18 h at 37℃. After 18 h, the reaction mixture was diluted with saturated $NaHCO_3$ (2 mL) and 1,3,5-trimethoxybenzene (33.6 mg, 0.2 mmol,1 equiv.) w.r.t. the substrate was added as

^{1}H NMR internal standard and stirred for 20 mins. The water phase was then extracted with CH_2Cl_2 (3 x 5 mL). The combined organic phases were washed with brine and dried over anhydrous Na_2SO_4, filtered, and concentrated in *vacuo*.

The yield of disulfide product (1b) was determined by ^{1}H NMR with respect to 1,3,5-trimethoxybenzene as internal standard.

11) Reaction of Methyl phenyl sulfoxide (1b) instead of Methyl phenyl sulfide under standard reaction condition

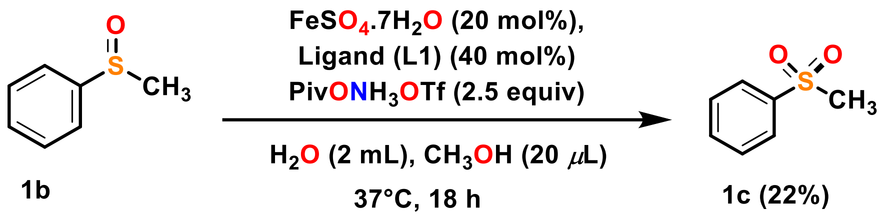

Scheme S26. Reaction of methyl phenyl sulfoxide with $PivONH_3OTf$ under standard condition in the presence of iron catalyst and ligand.

A clean screw cap vial was equipped with a magnetic bar in a stirrer. Then $FeSO_4.7H_2O$ (11 mg, 0.04 mmol, 20 mol%) and 1,10-phenanthroline (15.8 mg, 0.08 mmol, 40 mol%) was added followed by immediate addition of solid oxidant $PivONH_3OTf$ (Ox_1) (133.6 mg, 0.5 mmol, 2.5 equiv.). 2.0 mL of H_2O and 20 μL CH_3OH was added via syringe followed by immediate addition of 4-Methyl phenyl sulfoxide (23.5 μL, 0.2 mmol) substrate. The resulting mixture was sealed and stirred for 18 h at 37℃. After 18 h, the reaction mixture was diluted with saturated $NaHCO_3$ (2 mL) and 1,3,5-trimethoxybenzene (33.6 mg, 0.2 mmol,1 equiv.) w.r.t. the substrate was added as ^{1}H NMR internal standard and stirred for 20 mins. The water phase was then extracted with CH_2Cl_2 (3 x 5 mL). The combined organic phases were washed with brine and dried over anhydrous Na_2SO_4, filtered, and concentrated in *vacuo*.

The yield of Methyl phenyl sulfone (1c) was determined by ^{1}H NMR with respect to 1,3,5-trimethoxybenzene as internal standard.

Effect of other Oxidants (XII):

1) Sulfide oxidation under standard optimized condition using dioxygen (O_2) as oxidant

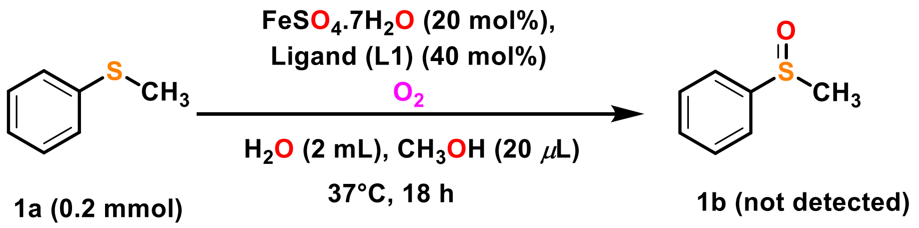

Scheme S27. Reaction of Sulfide using dioxygen as an oxidant instead of $PivONH_3OTf$ under standard condition in the presence of iron catalyst and ligand.

A clean screw cap vial was equipped with a magnetic bar in a stirrer. Then $FeSO_4.7H_2O$ (11 mg, 0.04 mmol, 20 mol%) and 1,10-phenanthroline (L1) (15.8 mg, 0.08 mmol, 40 mol%) was added To it 2.0 mL of H_2O and 20 μL CH_3OH was added via syringe followed by immediate addition of Methyl phenyl sulfide (23.5 μL, 0.2 mmol) substrate The resulting mixture was sealed and purged with dioxygen for 15 min and stirred for 18 h at 37℃ under oxygen atmosphere. After 18 h, the reaction mixture was diluted with saturated $NaHCO_3$ (2 mL) and 1,3,5-trimethoxybenzene (33.6 mg, 0.2 mmol,1 equiv.) w.r.t. the substrate was added as ^{1}H NMR internal standard and stirred for 20 mins. The water phase was then extracted with CH_2Cl_2 (3 x 5 mL). The combined organic phases were washed with brine and dried over anhydrous Na_2SO_4, filtered, and concentrated in *vacuo*. Product Methyl phenyl sulfoxide (1b) was not detected in the ^{1}H NMR.

2) Sulfide oxidation under standard optimized condition using *m*-CPBA as oxidant

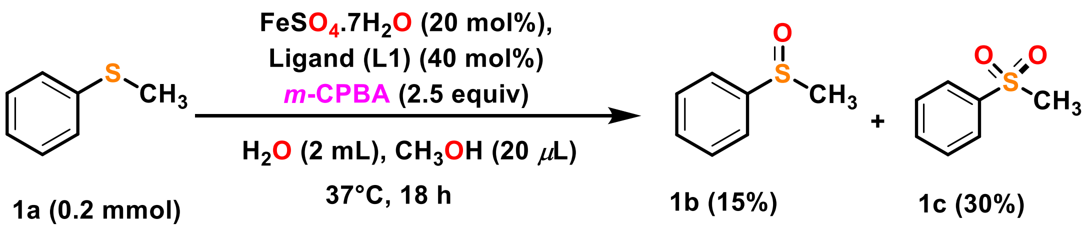

Scheme S28. Sulfide oxidation using *m*-CPBA as an oxidant instead of $PivONH_3OTf$ for the iron catalysed oxidative deamination of sulfide.

A clean screw cap vial was equipped with a magnetic bar in a stirrer. Then $FeSO_4.7H_2O$ (11 mg, 0.04 mmol, 20 mol%) and 1,10-phenanthroline (L1) (15.8 mg, 0.08 mmol, 40 mol%) was added followed by immediate addition of solid oxidant *m*-CPBA (86.385 mg, 0.5 mmol, 2.5 equiv.). 2.0 mL of H_2O and 20 μL CH_3OH was added via syringe followed by immediate addition of Methyl phenyl sulfide (23.5 μL, 0.2 mmol) substrate. The resulting mixture was sealed and stirred for 18 h at 37 ℃. After 18 h, the reaction mixture was diluted with saturated $NaHCO_3$ (2 mL) and 1,3,5-trimethoxybenzene (33.6 mg, 0.2 mmol,1 equiv.) w.r.t. the substrate was added as ^{1}H NMR internal standard and stirred for 20 mins. The water phase was then extracted with CH_2Cl_2 (3 x 5 mL). The combined organic phases were washed with brine and dried over anhydrous Na_2SO_4, filtered, and concentrated in *vacuo*.

The yield of Methyl phenyl sulfoxide product (**1b**) and Methyl phenyl sulfone (**1c**) was determined by ^{1}H NMR with respect to 1,3,5-trimethoxybenzene as internal standard.

3) Sulfide oxidation using *m*-CPBA as oxidant in the absence of metal catalyst and ligand

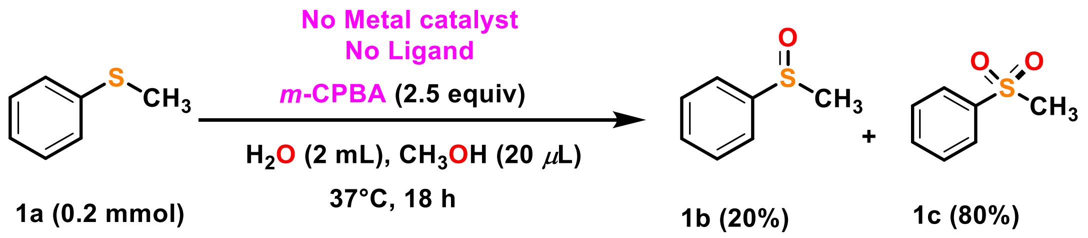

Scheme S29. Sulfide oxidation using *m*-CPBA as an oxidant instead of $PivONH_3OTf$ for the in the absence of any metal catalyst or ligand.

Radical scavenger reactions (XIII):

1) Iron catalysed sulfide oxidation under standard optimized condition in the presence of TEMPO radical.

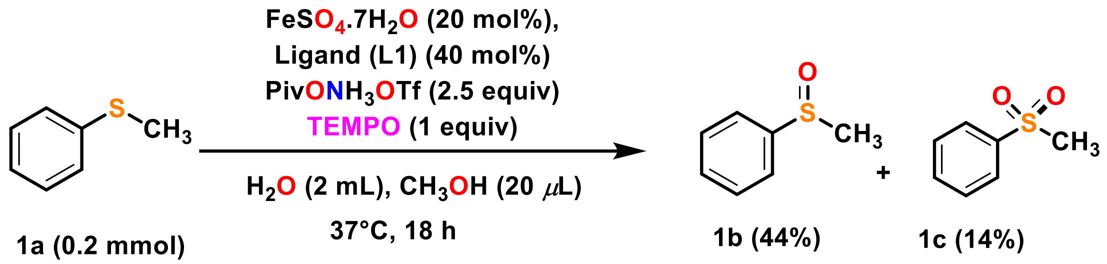

Scheme S30. Iron catalyzed sulfide oxidation under standard optimized condition in the presence of TEMPO radical (1 equiv).

A clean screw cap vial was equipped with a magnetic bar in a stirrer. Then $FeSO_4.7H_2O$ (11 mg, 0.04 mmol, 20 mol%) and 1,10-phenanthroline (15.8 mg, 0.08 mmol, 40 mol%) was added followed by immediate addition of solid oxidant $PivONH_3OTf$ (Ox_1) (133.6 mg, 0.5 mmol, 2.5 equiv.) and TEMPO (31.25 mg, 0.2 mmol, 1 equiv.). 2.0 mL of H_2O and 20 μL CH_3OH was added via syringe followed by immediate addition of Methyl phenyl sulfide (23.5 μL, 0.2 mmol) substrate. The resulting mixture was sealed and stirred for 18 h at 37 ℃. After 18 h, the reaction mixture was diluted with saturated $NaHCO_3$ (2 mL) and 1,3,5-trimethoxybenzene (33.6 mg, 0.2 mmol,1 equiv.) w.r.t. the substrate was added as ^1H NMR internal standard and stirred for 20 mins. The water phase was then extracted with CH_2Cl_2 (3 x 5 mL). The combined organic phases were washed with brine and dried over anhydrous Na_2SO_4, filtered, and concentrated in *vacuo*.

The yield of Methyl phenyl sulfoxide product (1b) and Methyl phenyl sulfone (1c) was determined by ^1H NMR with respect to 1,3,5-trimethoxybenzene as internal standard.

HR-MS(ESI$^+$) calc. for ($C_9H_{19}NO$) i.e $[M+H]^+ = [C_9H_{19}NO]^+[H]^+$ is 158.1545, found 158.16.

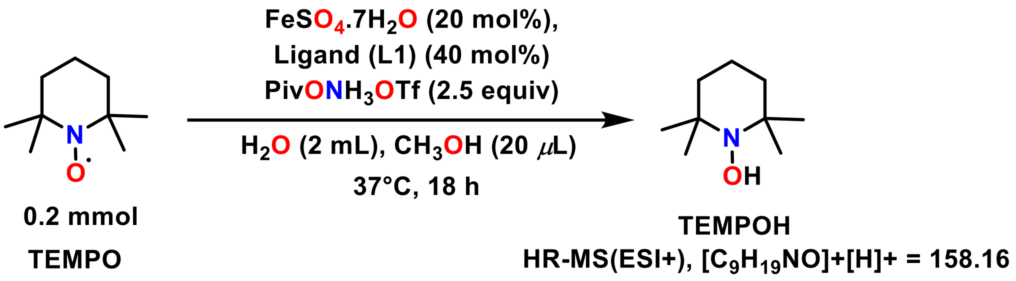

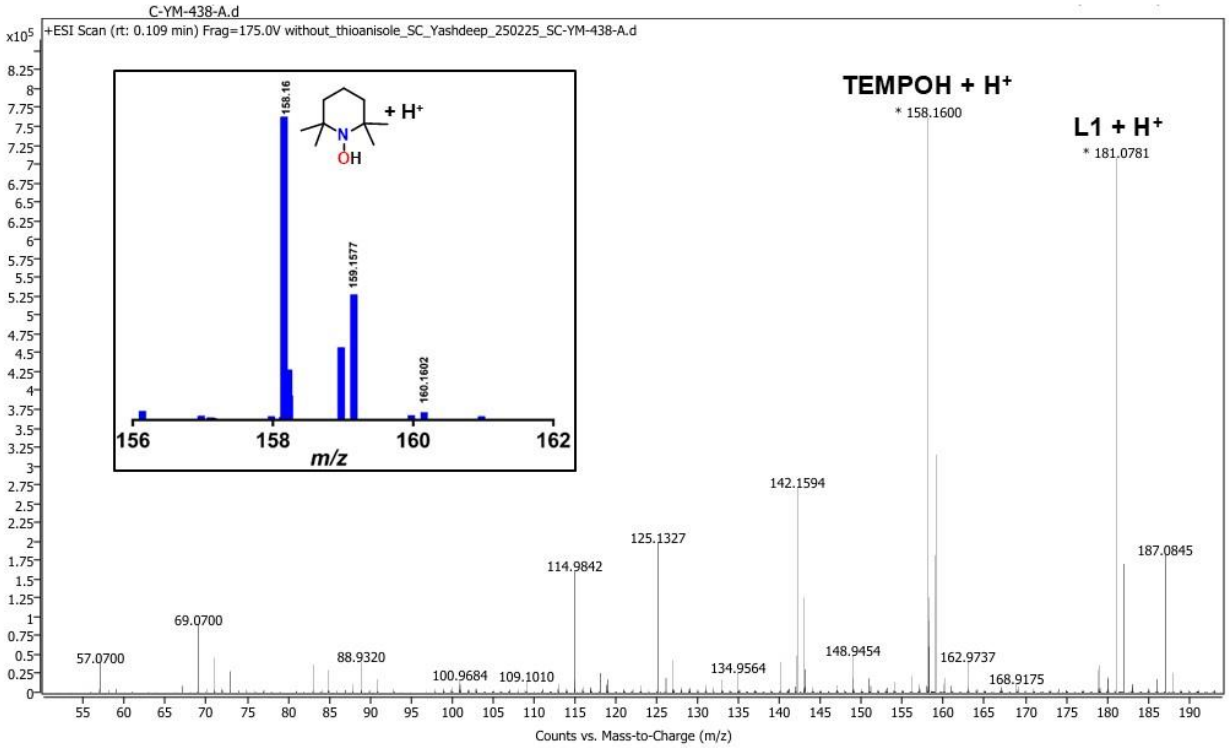

Figure S7. HRMS ESI positive ion mode from the reaction solution $FeSO_4.7H_2O$, L1 and Ox1 in the presence of 1 equiv. of TEMPO after stirring for 15 mins.

Labelling Experiments: Source of Oxygen in the Sulfoxide product (XIV):

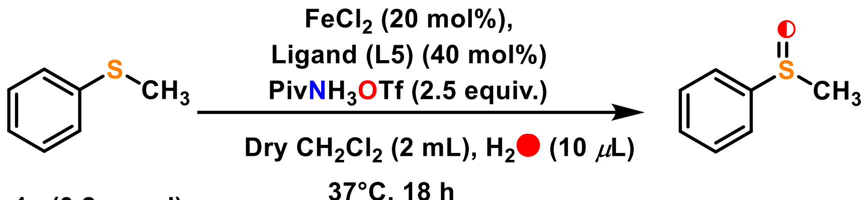

Scheme S31. $FeCl_2$ (5.06 mg, 0.04 mmol, 20 mol%) and Bipyridine (L5) (12.49 mg, 0.08 mmol, 40 mol%) Methyl phenyl sulfide (1a) (23.5 μL, 0.20 mmol) and $PivONH_3OTf$ (133.6 mg, 0.50 mmol), $H_2{}^{18}O$ (10 μL) and Dry DCM (2 mL).

A clean screw cap vial was equipped with a magnetic bar in a stirrer. Then anhydrous $FeCl_2$ (1.26 mg, 0.04 mmol, 20 mol%) and bipyridine (L5) (15.8 mg, 0.08 mmol, 40 mol%) was added followed by immediate addition of solid oxidant $PivONH_3OTf$ (133.6 mg, 0.5 mmol, 2.5 equiv.). 2.0 mL of dry dichloromethane and 10 μL labelled water ($H_2{}^{18}O$) was added via syringe followed by immediate addition of Methyl phenyl sulfide (23.5 μL, 0.2 mmol) substrate The resulting mixture was sealed and stirred for 18h at 37 ℃. The crude reaction was analyzed by HRMS. The remaining amount of the reaction mixture was quenched following standard work up procedure and the organic product was analysed by HRMS.

Peak at *m/z* = 143 appeared along with peak at *m/z* =141 for methyl phenyl sulfoxide in both the cases, confirming the source of oxygen to be from water.

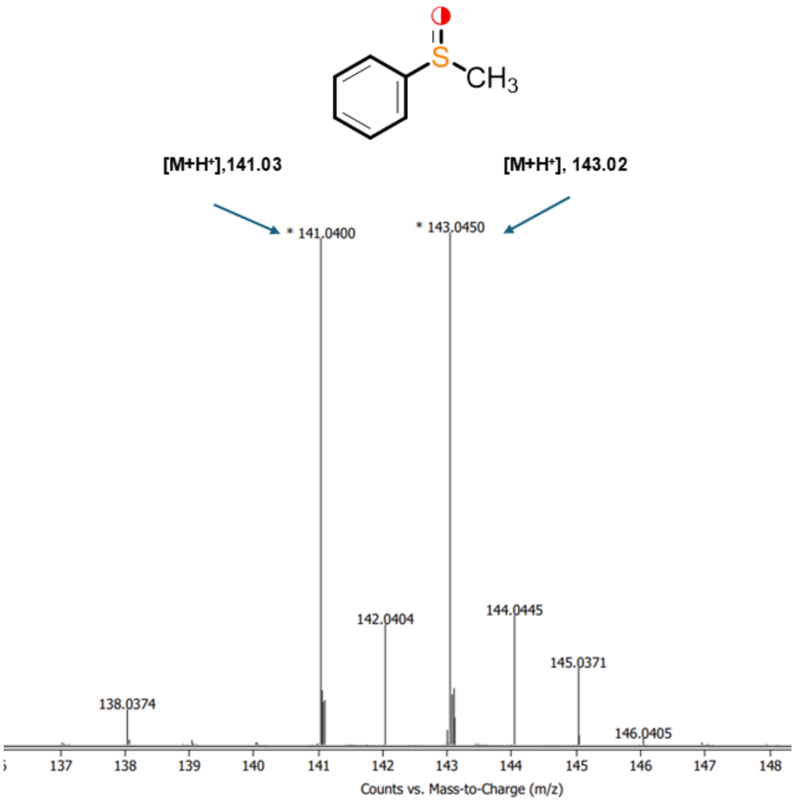

Figure S8. HRMS data from water labelling experiment.

Reaction in aqueous buffer solution (XV):

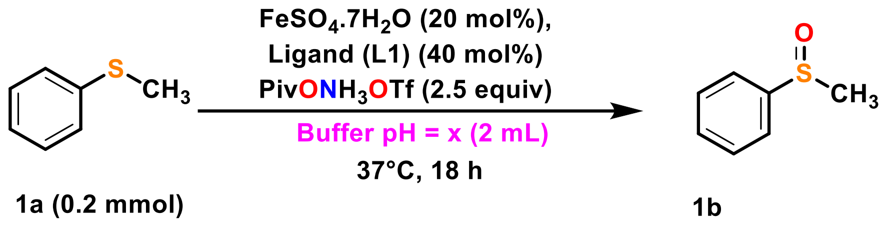

Scheme S32. Product derived from iron catalysed sulfide oxidation in buffer solution at different pH under standard condition.

A clean screw cap vial was equipped with a magnetic bar in a stirrer. Then $FeSO_4.7H_2O$ (11 mg, 0.04 mmol, 20 mol%) and 1,10-phenanthroline (15.8 mg, 0.08 mmol, 40 mol%) was added followed by immediate addition of solid oxidant $PivONH_3OTf$ (Ox_1) (133.6 mg, 0.5 mmol, 2.5 equiv.). 2 ml of respective buffer (of different pH) was added via syringe followed by immediate addition of Methyl phenyl sulfide (23.5 μL, 0.2 mmol) substrate. The resulting mixture was sealed and stirred for 18 h at 37 ℃. After 18 h, the reaction mixture was diluted with saturated $NaHCO_3$ (2 mL) and 1,3,5-trimethoxybenzene (33.6 mg, 0.2 mmol,1 equiv.) w.r.t. the substrate was added as ^{1}H NMR internal standard and stirred for 20 mins. The water phase was then extracted with CH_2Cl_2 (3 x 5 mL). The combined organic phases were washed with brine and dried over anhydrous Na_2SO_4, filtered, and concentrated in *vacuo*.

The yield of Methyl phenyl sulfoxide product (1b) was determined by ^{1}H NMR with respect to 1,3,5-trimethoxybenzene as internal standard.

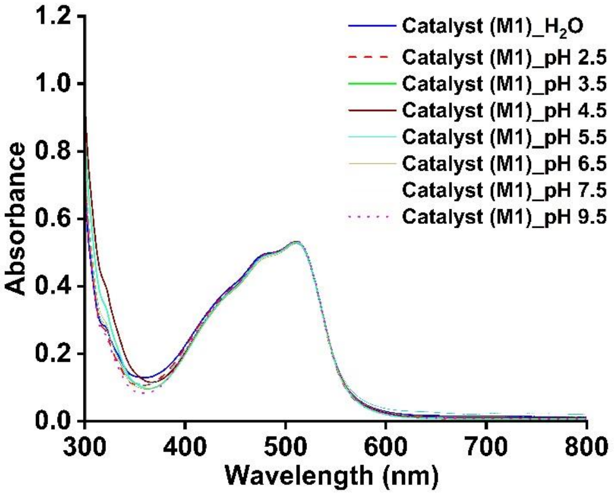

Figure S9a. Stability and homogeneity of iron catalyst $[Fe(L1)(H_2O)_2]^{2+}$ (**M1**) in buffer solution at different pH monitored by UV-Vis spectroscopy at 25°C. (concentration = 0.1 mM, pathlength = 1 cm.

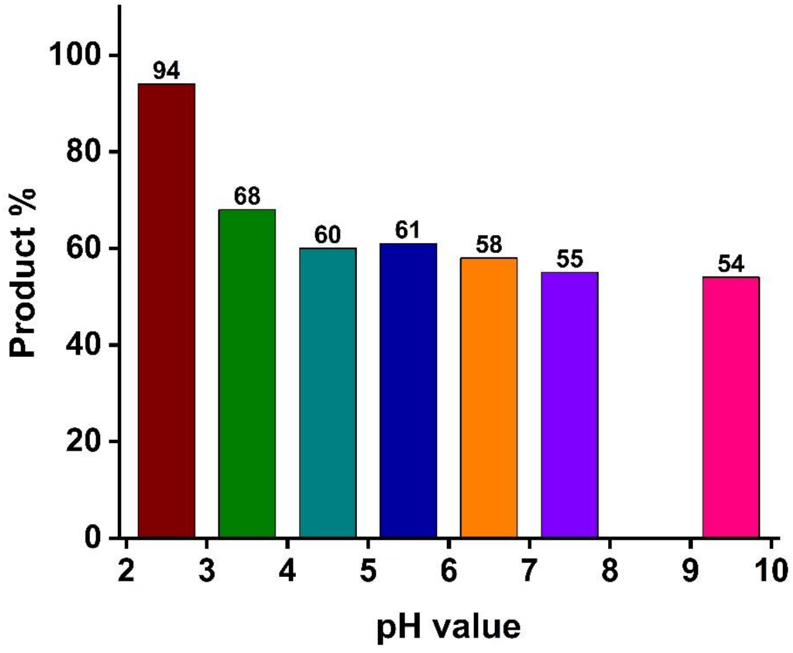

Figure S9b. Iron catalyzed sulfide oxidation using Ox1 in buffer solution of different pH under standard reaction condition

Table S12. Sulfide oxidation in buffer solution under standard condition.

**Entry**	**pH**[a]	**Yield (1b) %**
**1.**	2.5[b]	94%
**2.**	3.5[c]	68%
**2.**	4.5[c]	60%
**3.**	5.5[c]	61%
**4.**	6.5[c]	58%
**5.**	7.5[c]	55%
**7**	9.5[c]	54% (56%)[d]

a) $FeSO_4.7H_2O$ (11 mg, 0.04 mmol, 20 mol%) and 1,10-phenanthroline (L1) (15.8 mg, 0.08 mmol, 40 mol%) Methyl phenyl sulfide (1a) (23.5 µL, 0.20 mmol) and $PivONH_3OTf$ (133.6 mg, 0.50 mmol, 2.5 equiv). b) citrate buffer was used c) M9 buffer was used [4] d) alkaline phosphate buffer was used.

Competitive Reaction: Chemoselectivity of the reaction (XVI):

a) Competition reaction with alkene:

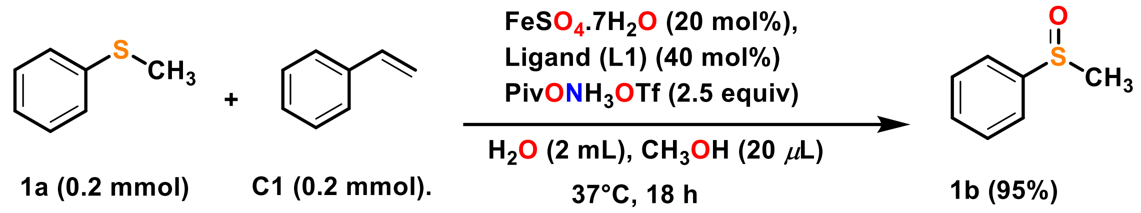

Scheme S33. Competition reaction of methyl phenyl sulfide and styrene catalyzed by iron(II) catalyst in the presence of oxidant $PivONH_3OTf$ (Ox_1).

A clean screw cap vial was equipped with a magnetic bar in a stirrer. Then $FeSO_4.7H_2O$ (11 mg, 0.04 mmol, 20 mol%) and 1,10-phenanthroline (L1) (15.8 mg, 0.08 mmol, 40 mol%) was added followed by immediate addition of solid oxidant $PivONH_3OTf$ (Ox_1) (133.6 mg, 0.5 mmol, 2.5 equiv.). 2.0 mL of H_2O and 20 μL CH_3OH was added via syringe followed by immediate addition of Methyl phenyl sulfide (23.5 μL, 0.2 mmol) substrate and Styrene (23 μL, 0.2 mmol). The resulting mixture was sealed and stirred for 18 h at 37 ℃. After 18 h, the reaction mixture was diluted with saturated $NaHCO_3$ (2 mL) and 1,3,5-trimethoxybenzene (33.6 mg, 0.2 mmol,1 equiv.) w.r.t. the substrate was added as ^1H NMR internal standard and stirred for 20 mins. The water phase was then extracted with CH_2Cl_2 (3 x 5 mL). The combined organic phases were washed with brine and dried over anhydrous Na_2SO_4, filtered, and concentrated in *vacuo*.

The yield of Methyl phenyl sulfoxide product (1b) was determined by ^1H NMR with respect to 1,3,5-trimethoxybenzene as internal standard. No significant product derived from styrene were detected, it was recovered almost quantitatively.

b) Competition reaction with thiols:

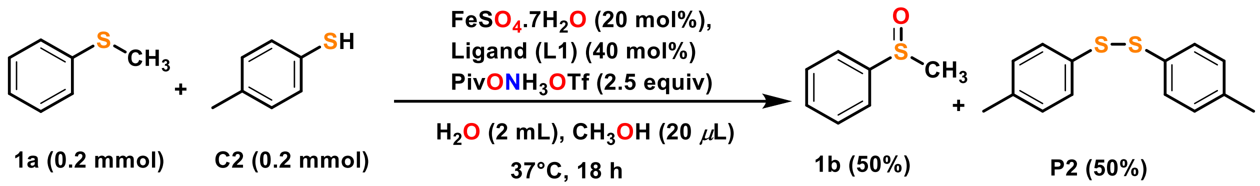

Scheme S34. Competition reaction of methyl phenyl sulfide and 4-Methyl benzene thiol catalyzed by iron(II) catalyst in the presence of oxidant $PivONH_3OTf$ (Ox_1).

A clean screw cap vial was equipped with a magnetic bar in a stirrer. Then $FeSO_4.7H_2O$ (11 mg, 0.04 mmol, 20 mol%) and 1,10-phenanthroline (L1) (15.8 mg, 0.08 mmol, 40 mol%) was added followed by immediate addition of solid oxidant $PivONH_3OTf$ (Ox_1) (133.6 mg, 0.5 mmol, 2.5 equiv.). 2.0 mL of H_2O and 20 μL CH_3OH was added via syringe followed by immediate addition of Methyl phenyl sulfide (23.5 μL, 0.2 mmol) substrate and 4-Methyl benzenethiol (C2) (24.84 mg gm, 0.2 mmol). The resulting mixture was sealed and stirred for 18 h at 37℃. After 18 h, the reaction mixture was diluted with saturated $NaHCO_3$ (2 mL) and 1,3,5-trimethoxybenzene (33.6 mg, 0.2 mmol,1 equiv.) w.r.t. the substrate was added as ^1H NMR internal standard and stirred for 20 mins. The water phase was then extracted with CH_2Cl_2 (3 x 5 mL). The combined organic phases were washed with brine and dried over anhydrous Na_2SO_4, filtered, and concentrated in *vacuo*.

The yield of Methyl phenyl sulfoxide product (1b) was determined by ^1H NMR with respect to 1,3,5-trimethoxybenzene as internal standard. Product disulphide (P2) derived from 4-Methyl benzenethiol was also determined by ^1H NMR with respect to 1,3,5-trimethoxybenzene as internal standard.

c) Competition reaction with disulfides:

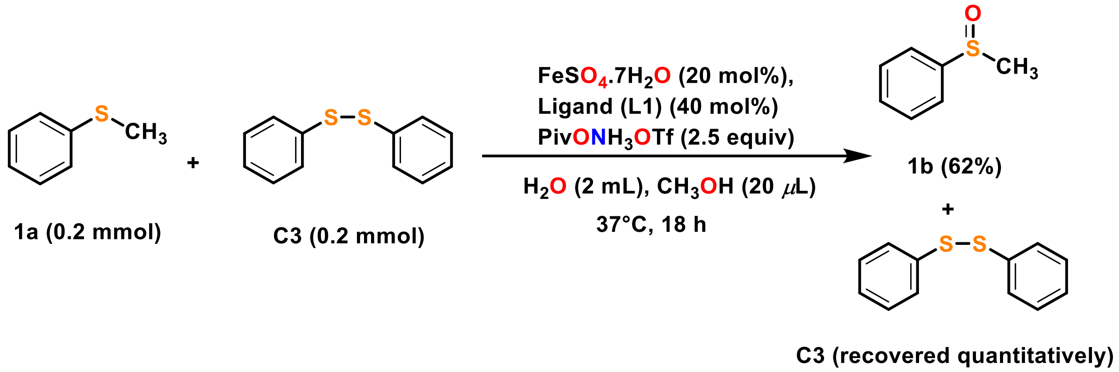

Scheme S35. Competition reaction of methyl phenyl sulfide and Benzene disulfide catalyzed by iron(II) catalyst in the presence of oxidant $PivONH_3OTf$ (Ox_1).

A clean screw cap vial was equipped with a magnetic bar in a stirrer. Then $FeSO_4.7H_2O$ (11 mg, 0.04 mmol, 20 mol%) and 1,10-phenanthroline (L1) (15.8 mg, 0.08 mmol, 40 mol%) was added followed by immediate addition of solid oxidant $PivONH_3OTf$ (Ox_1) (133.6 mg, 0.5 mmol, 2.5 equiv.). 2.0 mL of H_2O and 20 μL CH_3OH was added via syringe followed by immediate addition of Methyl phenyl sulfide (23.5 μL, 0.2 mmol) substrate and Benzene disulfide (43.6 mg, 0.2 mmol). The resulting mixture was sealed and stirred for 18 h at 37℃. After 18 h, the reaction mixture was diluted with saturated $NaHCO_3$ (2 mL) and 1,3,5-trimethoxybenzene (33.6 mg, 0.2 mmol,1 equiv.) w.r.t. the substrate was added as ^{1}H NMR internal standard and stirred for 20 mins. The water phase was then extracted with CH_2Cl_2 (3 x 5 mL). The combined organic phases were washed with brine and dried over anhydrous Na_2SO_4, filtered, and concentrated in *vacuo*.

The yield of Methyl phenyl sulfoxide product (1b) was determined by ^{1}H NMR with respect to 1,3,5-trimethoxybenzene as internal standard. No significant product derived from benzene disulphide were detected, it was recovered almost quantitatively.

Variation of scale of the developed reaction (XVII):

1) Reaction compatibility at low scale range:

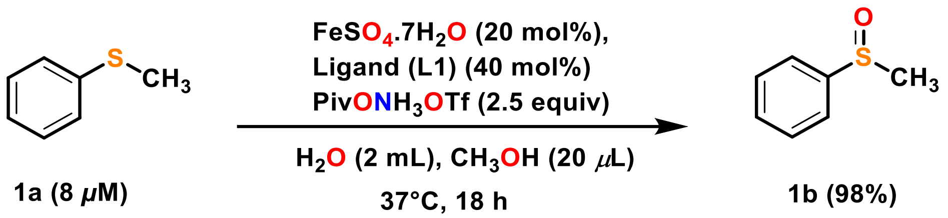

Scheme S36. Sulfide oxidation compatibility at low concentration range (8 μM of substrate) catalyzed by iron(II) and PivONH$_3$OTf (Ox$_1$) in water.

A clean screw cap vial was equipped with a magnetic bar in a stirrer. Then FeSO$_4$.7H$_2$O (0.448 mg, 1.6 μM, 20 mol %) and 1,10-phenanthroline (0.64 mg, 3.2 μM, 40 mol%) was added followed by immediate addition of solid oxidant PivONH$_3$OTf (Ox$_1$) (5.34 mg, 20 μM, 2.5 equiv.). 2.0 mL of H$_2$O and 20 μL CH$_3$OH was added via syringe followed by immediate addition of Methyl phenyl sulfide (0.97 μL, 8 μM) substrate. The resulting mixture was sealed and stirred for 18 h at 37℃. After 18 h, the reaction mixture was diluted with saturated NaHCO$_3$ (2 mL) and 1,3,5-trimethoxybenzene (1.35 mg, 8 μM,1 equiv.) w.r.t. the substrate was added as ^{1}H NMR internal standard and stirred for 20 mins. The water phase was then extracted with CH$_2$Cl$_2$ (3 x 5 mL). The combined organic phases were washed with brine and dried over anhydrous Na$_2$SO$_4$, filtered, and concentrated in *vacuo*. The yield of Methyl phenyl sulfoxide product (1b) was determined by ^{1}H NMR with respect to 1,3,5-trimethoxybenzene as internal standard.

2) Reaction compatibility at large scale range: Gram scale synthesis

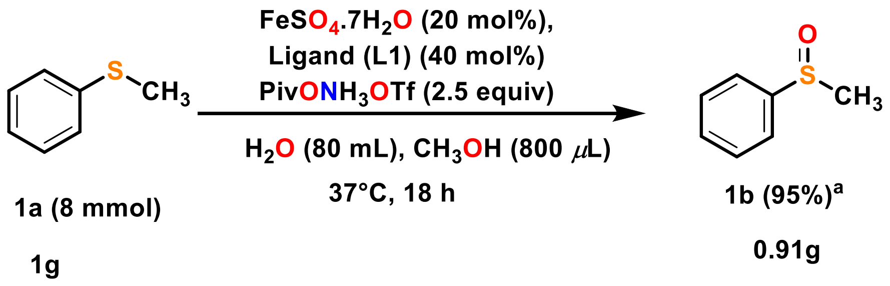

Scheme S37. Large scale reaction for sulfide oxidation catalyzed by iron (II) and PivONH$_3$OTf (Ox$_1$) in water. a) Value in the parenthesis denotes crude yield. The isolated yield in gram scale synthesis was ≥ 80%.

A clean round bottomed flask was equipped with a magnetic bar in a stirrer. Then $FeSO_4.7H_2O$ (0.44g, 1.6 mmol, 20 mol%) and 1,10-phenanthroline (L1) (0.576 g, 3.2 mmol, 40 mol%) was added followed by immediate addition of solid oxidant $PivONH_3OTf$ (Ox_1) (5.34 g, 20 mmol, 2.5 equiv.). 80 mL of H_2O and 800 μL CH_3OH was added via syringe followed by immediate addition of Methyl phenyl sulfide (0.94 mL, 8 mmol) substrate The resulting mixture was sealed and stirred for 18 h at 37℃. After 18 h, the reaction mixture was diluted with saturated $NaHCO_3$ (80 mL) and 1,3,5-trimethoxybenzene (1.34g, 8 mmol,1 equiv.) w.r.t. the substrate was added as ^{1}H NMR internal standard and stirred for 20 mins. The water phase was then extracted with CH_2Cl_2 (3 x 100 mL). The combined organic phases were washed with brine and dried over anhydrous Na_2SO_4, filtered, and concentrated in *vacuo*. The yield of Methyl phenyl sulfoxide product (1b) was determined by ^{1}H NMR with respect to 1,3,5-trimethoxybenzene as internal standard.

Reaction compatibilty in presence of bio-additives (XVIII):

A clean screw cap vial was equipped with a magnetic bar in a stirrer. Then $FeSO_4.7H_2O$ (11 mg, 0.04 mmol, 20 mol%) and 1,10-phenanthroline (15.8 mg, 0.08 mmol, 40 mol%) was added followed by immediate addition of solid oxidant $PivONH_3OTf$ (Ox_1) (5.34 mg, 20 μM, 2.5 equiv.). 2.0 mL of H_2O and 20 μL CH_3OH was added via syringe followed by immediate addition of Methyl phenyl sulfide (23.5 μL, 0.2 mmol) substrate and bio-additive molecule (0.04mmol,20 mol%). The resulting mixture was sealed and stirred for 18 h at 37℃. After 18 h, the reaction mixture was diluted with saturated $NaHCO_3$ (2 mL) and 1,3,5-trimethoxybenzene (33.6 mg, 0.2 mmol,1 equiv. w.r.t. the substrate was added as ^{1}H NMR internal standard and stirred for 20 mins. The water phase was then extracted with CH_2Cl_2 (3 x 5 mL). The combined organic phases were washed with brine and dried over anhydrous Na_2SO_4, filtered, and concentrated in *vacuo*.

The yield of Methyl phenyl sulfoxide product (1b) was determined by ^{1}H NMR with respect to 1,3,5-trimethoxybenzene as internal standard.

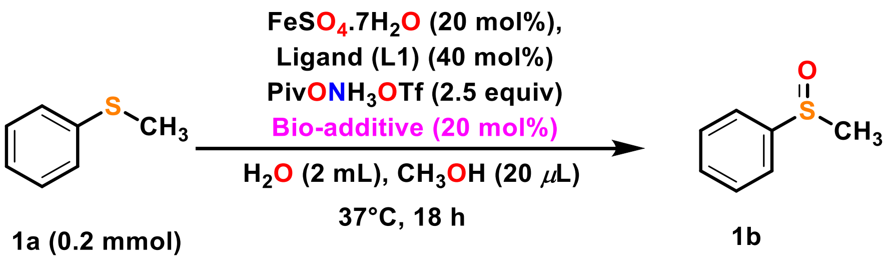

Scheme S38. Reaction of sulfide with $PivONH_3OTf$ (Ox_1) in the presence of bio-additive molecule.

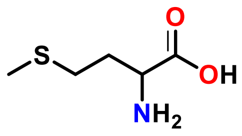

Methionine

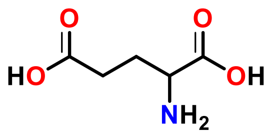

Glutamic Acid

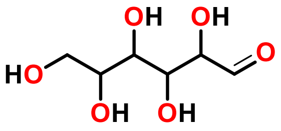

Glucose

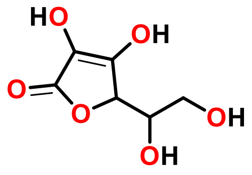

Ascorbic acid

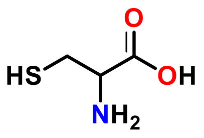

Cysteine

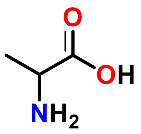

Alanine

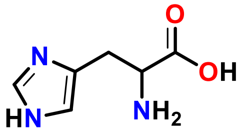

Histidine

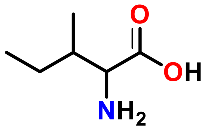

Isoleucine

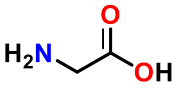

Glycine

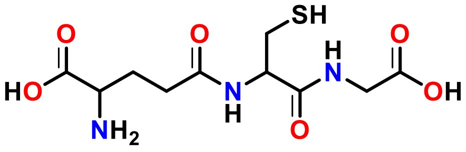

Glutathione

Scheme S39. Amino acids used as external additives under standard reaction conditions.

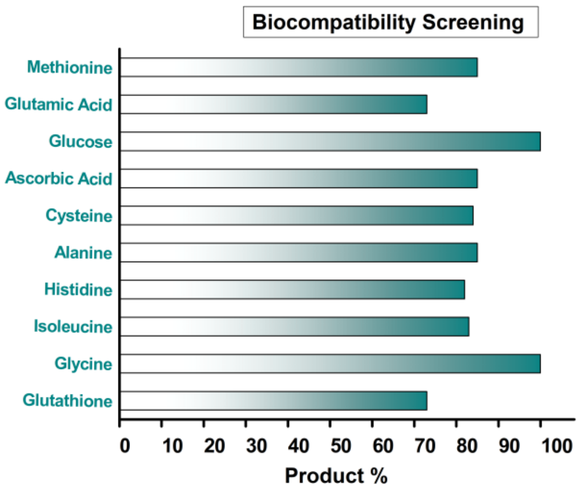

Figure S10. Reaction selectivity for sulfoxide product in the iron catalyzed reaction of sulfide with $PivONH_3OTf$ (Ox_1) in the presence of different bio-additive molecules.

Table S13. Screening of different amino acids under standard reaction condition.

**Entry**	**Amino-Acid**[a]	**Product (1b) %**
1	Methionine	85%
2	Glutamic Acid	73%
3	Glucose	100%
4	Ascorbic Acid	85%
5	Cysteine	82%
6	Alanine	85%
7	Histidine	82%
8	Isoleucine	83%
9	Glycine	100%
10	Glutathione	73%

a) $FeSO_4.7H_2O$ (11 mg, 0.04 mmol, 20 mol%) and 1,10-phenanthroline (L1) (15.8 mg, 0.08 mmol, 40 mol%) Methyl phenyl sulfide (1a) (23.5 μL, 0.20 mmol), $PivONH_3OTf$ (133.6 mg, 0.50 mmol and amino acid (0.04 mmol, 20 mol%).

General optimized procedure for aromatic substrate scope oxidation of sulfides (XIX):

A clean screw cap vial was equipped with a magnetic bar in a stirrer. Then $FeSO_4.7H_2O$ (11 mg, 0.04 mmol, 20 mol%) and 1,10-phenanthroline (L1) (15.8 mg, 0.08 mmol, 40 mol%) was added followed by immediate addition of solid oxidant $PivONH_3OTf$ (133.6 mg, 0.5 mmol, 2.5 equiv.). 2.0 mL of H_2O and 20 μL CH_3OH was added via syringe followed by immediate addition of respective sulfide (0.2 mmol) substrate The resulting mixture was sealed and stirred for 18 hr at 37℃. After 18 hr, the reaction mixture was diluted with saturated $NaHCO_3$ (2 mL) and 1,3,5-trimethoxybenzene (33.6 mg, 0.2 mmol,1 equiv.) w.r.t. the substrate was added as ^{1}H NMR internal standard and stirred for 20 mins. The water phase was then extracted with CH_2Cl_2 (3 x 5 mL). The combined organic phases were washed with brine and dried over anhydrous Na_2SO_4, filtered, and concentrated in *vacuo*. The yield of sulfoxide product (XX) was determined by ^{1}H NMR with respect to 1,3,5-trimethoxybenzene as internal standard.

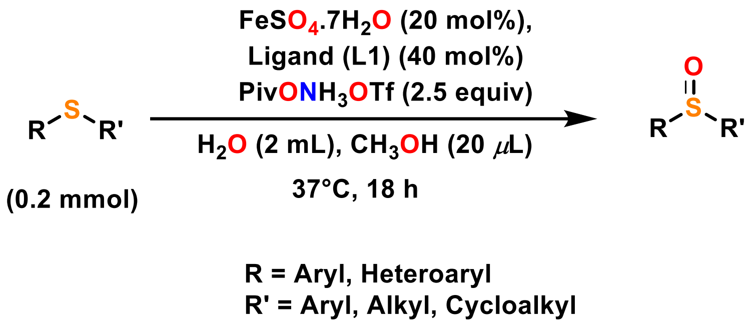

Scheme S40. General procedure of aromatic substrate for sulfide oxidation by oxidant $PivONH_3OTf$ under standard condition catalyzed by iron catalyst and Ligand L1.

General optimized procedure for aliphatic substrate scope of sulfide oxidation (XIX'):

A clean screw cap vial was equipped with a magnetic bar in a stirrer. Then anhydrous $FeCl_2$ (5 mg, 0.04 mmol, 20 mol%) and 2,2 bipyridine (L5) (12.49 mg, 0.08 mmol, 40 mol%) was added followed by immediate addition of solid oxidant $PivONH_3OTf$ (133.6 mg, 0.5 mmol, 2.5 equiv.). 2.0 mL of H_2O and 20 μL CH_3OH was added via syringe followed by immediate addition of respective sulfide (0.2 mmol) substrate The resulting mixture was sealed and stirred for 18 hr at 37℃. After 18 hr, the reaction mixture was diluted with saturated $NaHCO_3$ (2 mL) and 1,3,5-trimethoxybenzene (33.6 mg, 0.2 mmol,1 equiv.) w.r.t. the substrate was added as ^{1}H NMR internal standard and stirred for 20 mins. The water phase was then extracted with CH_2Cl_2 (3 x 5 mL). The combined organic phases were washed with brine and dried over anhydrous Na_2SO_4,

filtered, and concentrated in *vacuo*. The yield of sulfoxide product was determined by ^{1}H NMR with respect to 1,3,5-trimethoxybenzene as internal standard.

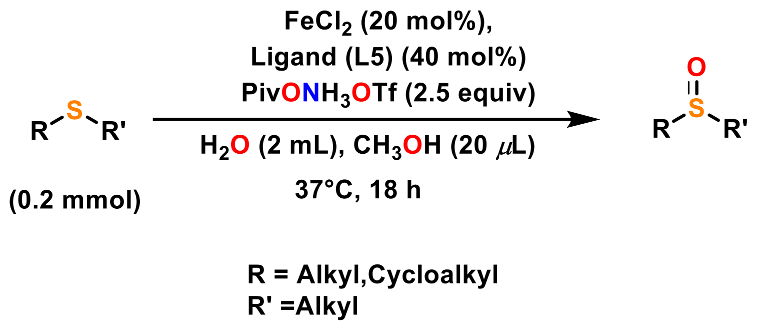

Scheme S41. General procedure of aliphatic substrate for sulfide oxidation by oxidant $PivONH_3OTf$ under standard condition catalyzed by iron catalyst and Ligand L5.

Tuning electronic factors to understand reaction mechanism (XX):

1) Effect of varying electron density of the oxidant (Ox) on the overall product profile:

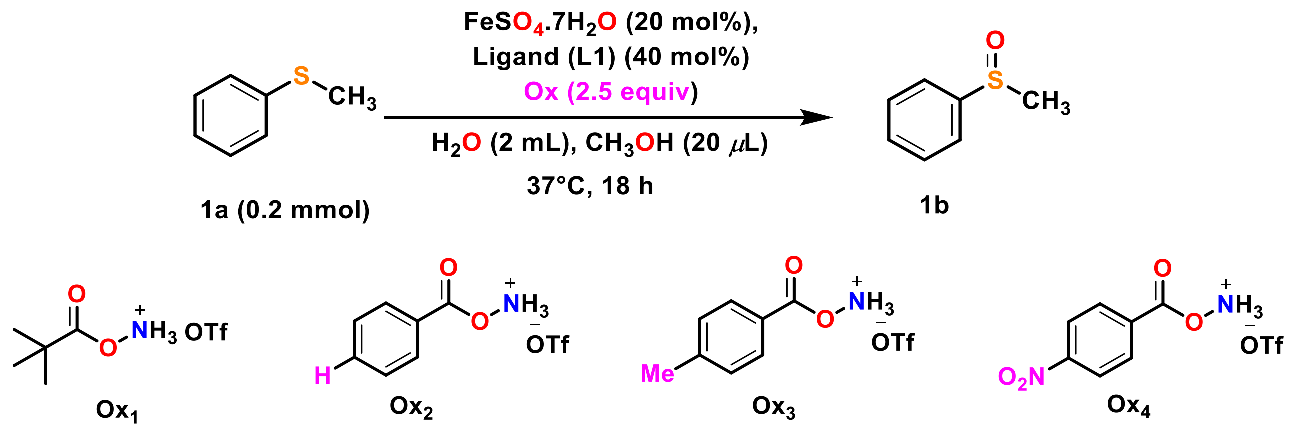

Scheme S42. Reaction of sulfide with varying electronic properties of oxidant (Ox) and its effect on the overall rate of reaction.

a) Using Methyl phenyl sulphide as substrate:

A clean screw cap vial was equipped with a magnetic bar in a stirrer. Then $FeSO_4.7H_2O$ (11 mg, 0.04 mmol, 20 mol%) and 1,10-phenanthroline (L1) (15.8 mg, 0.08 mmol, 40 mol%) was added followed by immediate addition of the respective solid oxidant (Ox) (2.5 equiv.). 2.0 mL of H_2O and 20 μL CH_3OH was added via syringe followed by immediate addition of Methyl phenyl sulfide (23.5 μL, 0.2 mmol) substrate. The resulting mixture was sealed and stirred for 18 h at 37 ℃. After 18 h, the reaction mixture was diluted with saturated $NaHCO_3$ (2 mL) and 1,3,5-

trimethoxybenzene (33.6 mg, 0.2 mmol,1 equiv. w.r.t. the substrate was added as ^{1}H NMR internal standard and stirred for 20 mins. The water phase was then extracted with CH_2Cl_2 (3 x 5 mL). The combined organic phases were washed with brine and dried over anhydrous Na_2SO_4, filtered, and concentrated in *vacuo*.

The yield of Methyl phenyl sulfoxide product (1b) was determined by ^{1}H NMR with respect to 1,3,5-trimethoxybenzene as internal standard.

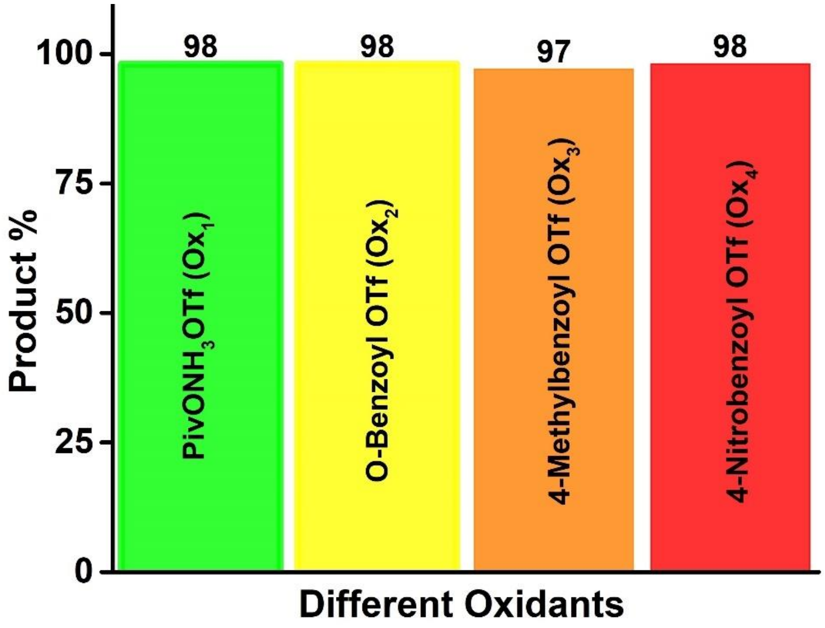

Figure S11. Formation of Methyl phenyl sulfoxide in iron catalyzed oxidative deamination reaction of sulfides in the presence of different oxidants.

b) Using 4-Methoxy-Methyl phenyl sulfoxide sulphide as substrate:

A clean screw cap vial was equipped with a magnetic bar in a stirrer. Then $FeSO_4.7H_2O$ (11 mg, 0.04 mmol, 20 mol%) and 1,10-phenanthroline (L1) (15.8 mg, 0.08 mmol, 40 mol%) was added followed by immediate addition of the respective solid oxidant (Ox) (2.5 equiv.). 2.0 mL of H_2O and 20 μL CH_3OH was added via syringe followed by immediate addition of 4-OMe-Methyl phenyl sulfide (27.8μL, 0.2 mmol) substrate. The resulting mixture was sealed and stirred for 18 h at 37℃. After 18 h, the reaction mixture was diluted with saturated $NaHCO_3$ (2 mL) and 1,3,5-trimethoxybenzene (33.6 mg, 0.2 mmol,1 equiv. w.r.t. the substrate was added as ^{1}H NMR internal standard and stirred for 20 mins. The water phase was then extracted with CH_2Cl_2 (3 x 5

mL). The combined organic phases were washed with brine and dried over anhydrous Na_2SO_4, filtered, and concentrated in *vacuo*.

The yield of 4-Methoxy-Methyl phenyl sulfoxide product (3b) was determined by ^{1}H NMR with respect to 1,3,5-trimethoxybenzene as an internal standard.

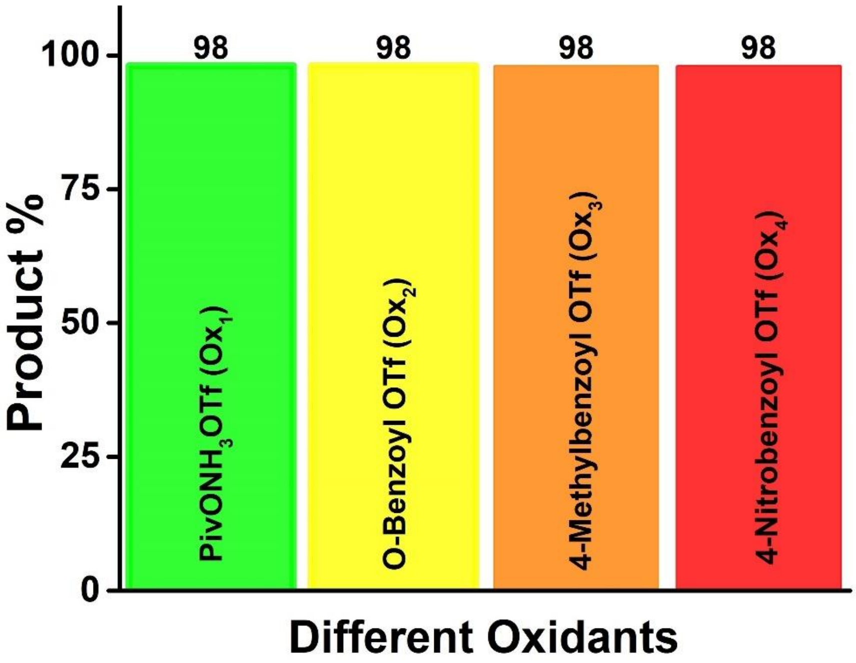

Figure S12. Formation of 4-Methoxy methyl phenyl sulfoxide in iron catalyzed oxidative deamination reaction of sulfides in the presence of different oxidants.

c) Using 4-Bromo methyl phenyl sulfide as substrate:

A clean screw cap vial was equipped with a magnetic bar in a stirrer. Then $FeSO_4.7H_2O$ (11 mg, 0.04 mmol, 20 mol%) and 1,10-phenanthroline (L1) (15.8 mg, 0.08 mmol, 40 mol%) was added followed by immediate addition of the respective solid oxidant (Ox) (2.5 equiv.). 2.0 mL of H_2O and 20 μL CH_3OH was added via syringe followed by immediate addition of 4-Bromo methyl phenyl sulfide 40.6 mg, 0.2 mmol) substrate. The resulting mixture was sealed and stirred for 18 h at 37℃. After 18 h, the reaction mixture was diluted with saturated $NaHCO_3$ (2 mL) and 1,3,5-trimethoxybenzene (33.6 mg, 0.2 mmol,1 equiv. w.r.t. the substrate was added as ^{1}H NMR internal standard and stirred for 20 mins. The water phase was then extracted with CH_2Cl_2 (3 x 5 mL). The combined organic phases were washed with brine and dried over anhydrous Na_2SO_4, filtered, and concentrated in *vacuo*.

The yield of 4-Bromo methyl phenyl sulfoxide product (9b) was determined by ^{1}H NMR with respect to 1,3,5-trimethoxybenzene as an internal standard

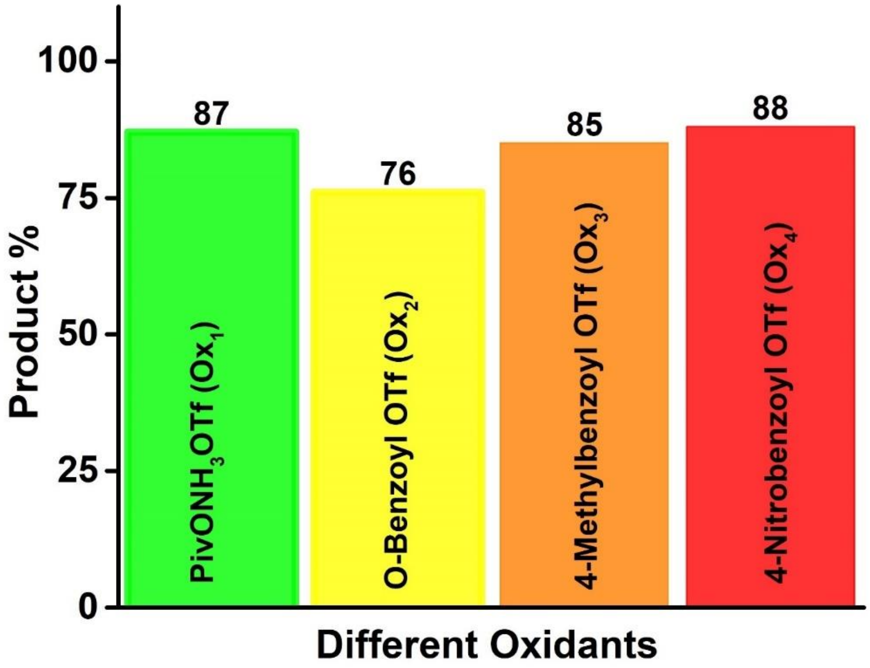

Figure S13. Formation of 4-Bromo methyl phenyl sulfoxide in iron catalyzed oxidative deamination reaction of sulfides in the presence of different oxidants.

d) Using 4-Nitro methyl phenyl sulphide as substrate:

A clean screw cap vial was equipped with a magnetic bar in a stirrer. Then $FeSO_4.7H_2O$ (11 mg, 0.04 mmol, 20 mol%) and 1,10-phenanthroline (L1) (15.8 mg, 0.08 mmol, 40 mol%) was added followed by immediate addition of the respective solid oxidant (Ox) (2.5 equiv.). 2.0 mL of H_2O and 20 μL CH_3OH was added via syringe followed by immediate addition of 4-Nitro methyl phenyl sulfide (33.8 mg, 0.2 mmol) substrate. The resulting mixture was sealed and stirred for 18 h at 37℃. After 18 h, the reaction mixture was diluted with saturated $NaHCO_3$ (2 mL) and 1,3,5-trimethoxybenzene (33.6 mg, 0.2 mmol,1 equiv. w.r.t. the substrate was added as ^{1}H NMR internal standard and stirred for 20 mins. The water phase was then extracted with CH_2Cl_2 (3 x 5 mL). The combined organic phases were washed with brine and dried over anhydrous Na_2SO_4, filtered, and concentrated in *vacuo*.

The yield of 4-Nitro methyl phenyl sulfoxide product (12b) was determined by ^{1}H NMR with respect to 1,3,5-trimethoxybenzene as an internal standard.

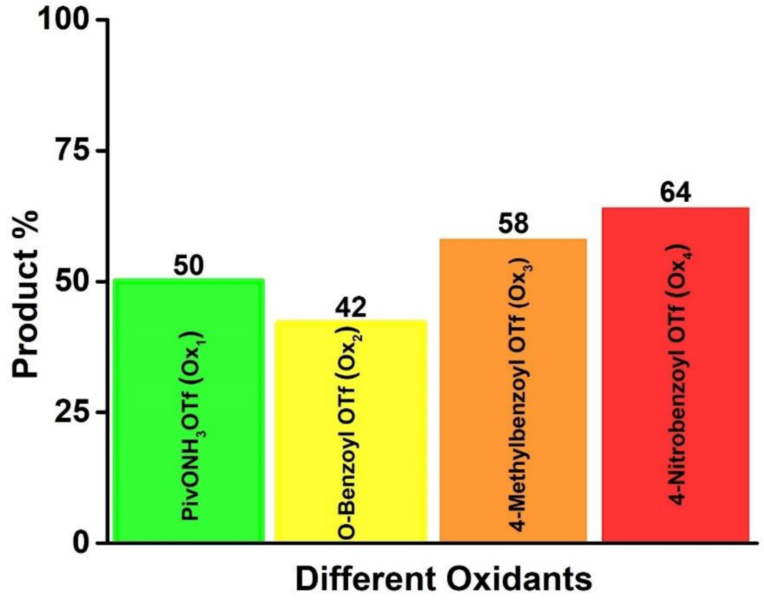

Figure S14. Formation of 4-Nitro methyl phenyl sulfoxide in iron catalyzed oxidative deamination reaction of sulfides in the presence of different oxidants.

2) Effect of varying electron density of the oxidant (Ox) at the initial stage of reaction:

A clean screw cap vial was equipped with a magnetic bar in a stirrer. Then $FeSO_4.7H_2O$ (11 mg, 0.04 mmol, 20 mol%) and 1,10-phenanthroline (L1) (15.8 mg, 0.08 mmol, 40 mol%) was added followed by immediate addition of the respective solid oxidant (Ox) (2.5 equiv.). 2.0 mL of H_2O and 20 μL CH_3OH was added via syringe followed by immediate addition of Methyl phenyl sulfide (23.5 μL, 0.2 mmol) substrate. The resulting mixture was sealed and stirred for 18 h at 37℃. After 15 min, the reaction mixture was diluted with saturated $NaHCO_3$ (2 mL) and 1,3,5-trimethoxybenzene (33.6 mg, 0.2 mmol,1 equiv. w.r.t. the substrate was added as ^{1}H NMR internal standard and stirred for 20 mins. The water phase was then extracted with CH_2Cl_2 (3 x 5 mL). The combined organic phases were washed with brine and dried over anhydrous Na_2SO_4, filtered, and concentrated in *vacuo*.

The yield of Methyl phenyl sulfoxide product (1b) was determined by ^{1}H NMR with respect to 1,3,5-trimethoxybenzene as internal standard.

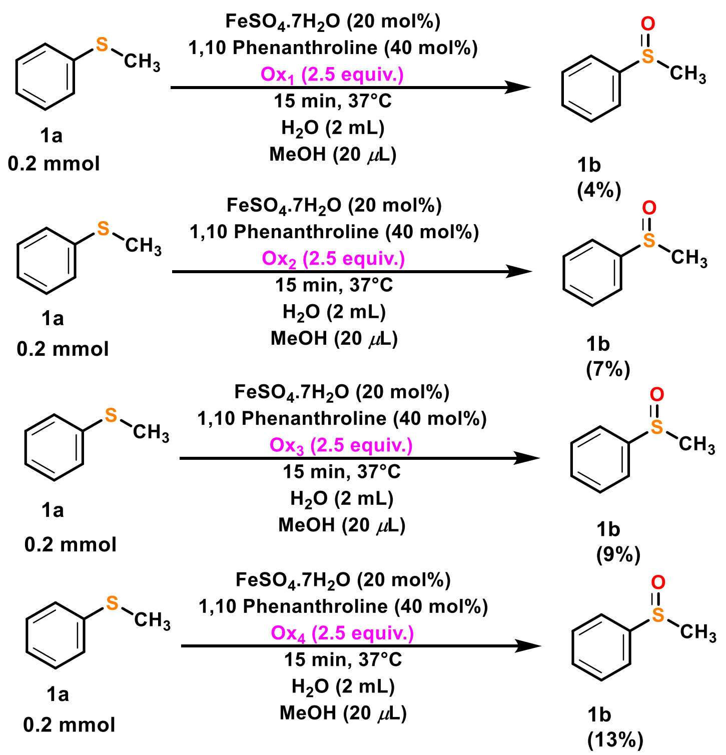

Scheme S43. Reaction of sulfide with varying electronic properties of oxidant (Ox) and its effect on the initial rate of reaction.

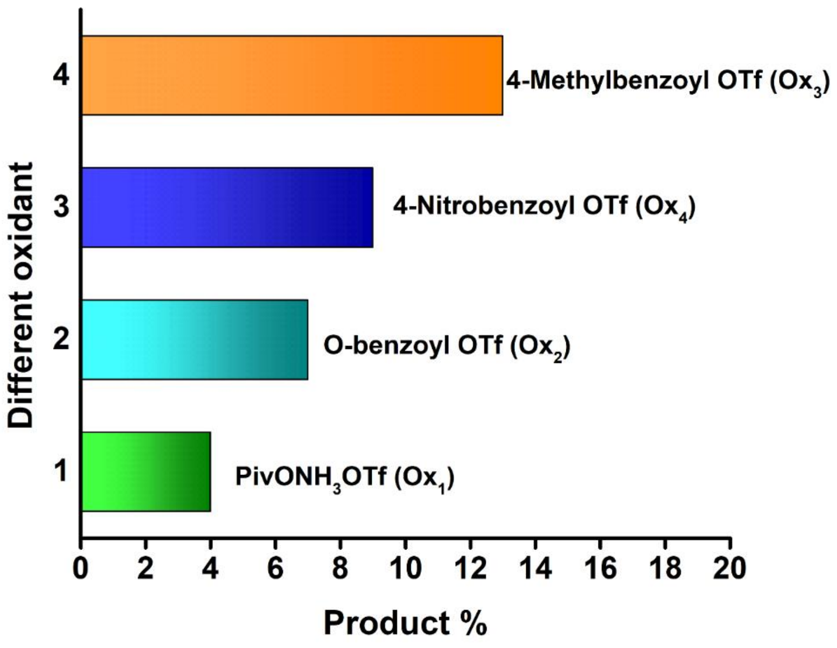

Figure S15. Conversion of sulfide to sulfoxide catalyzed by iron in the presence of different oxidants having varied electronic properties within 15 min of reaction.

3) **Effect of Tuning electronic properties of Ligand in the reaction yield:**

A clean screw cap vial was equipped with a magnetic bar in a stirrer. Then $FeSO_4.7H_2O$ (11 mg, 0.04 mmol, 20 mol%) and respective ligands (L) (40 mol%) (See Scheme XX) was added followed by immediate addition of the respective solid oxidant (Ox) (2.5 equiv.). 2.0 mL of H_2O and 20 μL CH_3OH was added via syringe followed by immediate addition of Methyl phenyl sulfide (23.5 μL, 0.2 mmol) substrate. The resulting mixture was sealed and stirred for 18 h at 37 ℃. After 18 h, the reaction mixture was diluted with saturated $NaHCO_3$ (2 mL) and 1,3,5-trimethoxybenzene (33.6 mg, 0.2 mmol,1 equiv. w.r.t. the substrate was added as ^{1}H NMR internal standard and stirred for 20 mins. The water phase was then extracted with CH_2Cl_2 (3 x 5 mL). The combined organic phases were washed with brine and dried over anhydrous Na_2SO_4, filtered, and concentrated in *vacuo*.

The yield of Methyl phenyl sulfoxide product (1b) was determined by ^{1}H NMR with respect to 1,3,5-trimethoxybenzene as internal standard.

(a)

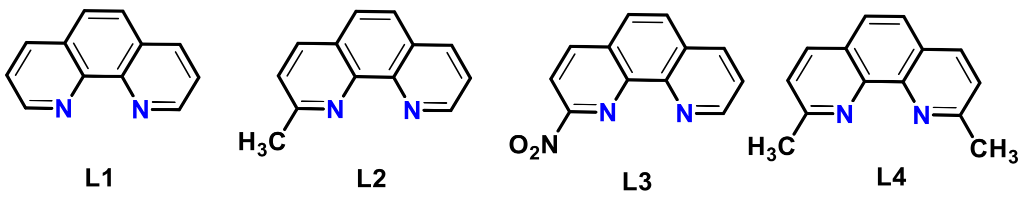

(b)

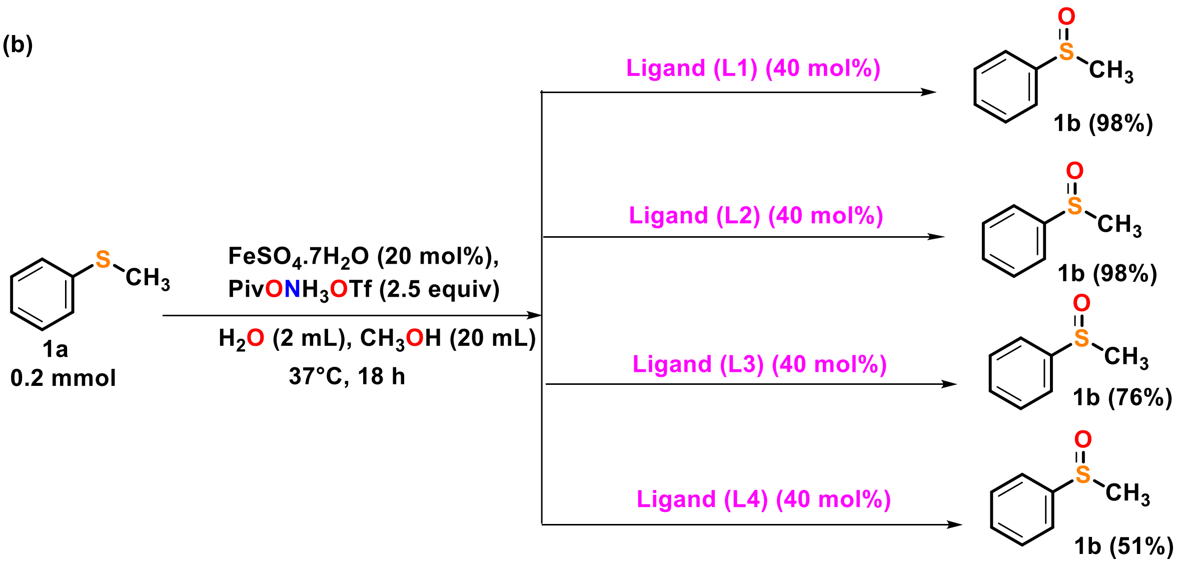

Scheme S44. a) Ligands with different stereoelectronic factors. b) Electronic effect of ligands (L) on sulfide oxidation reaction.

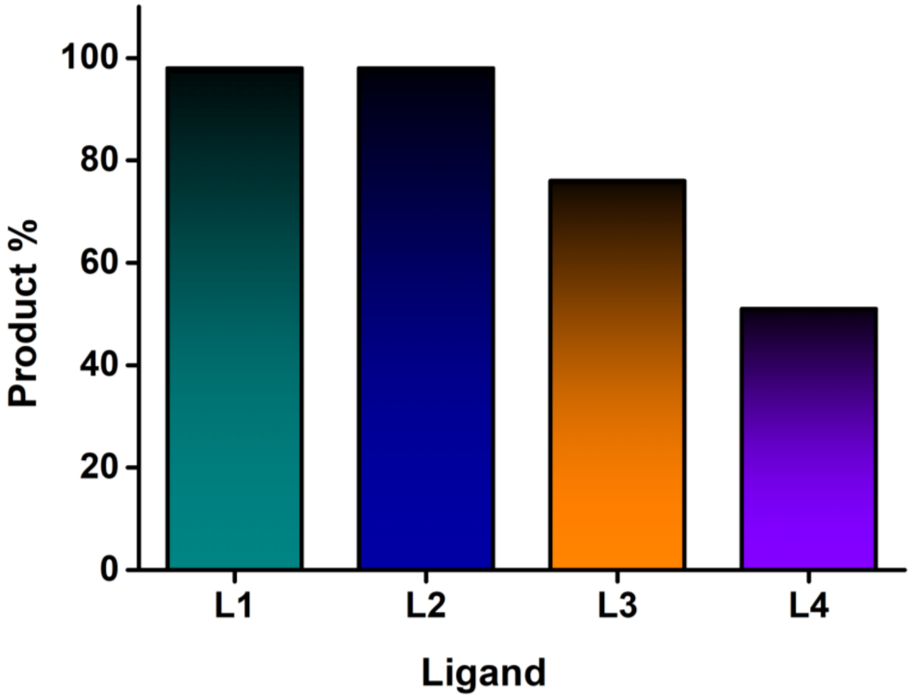

Figure S16. Electronic effect of ligand on standard reaction condition.

4) Electronic effect of substrate sulfide: Hammett analyses

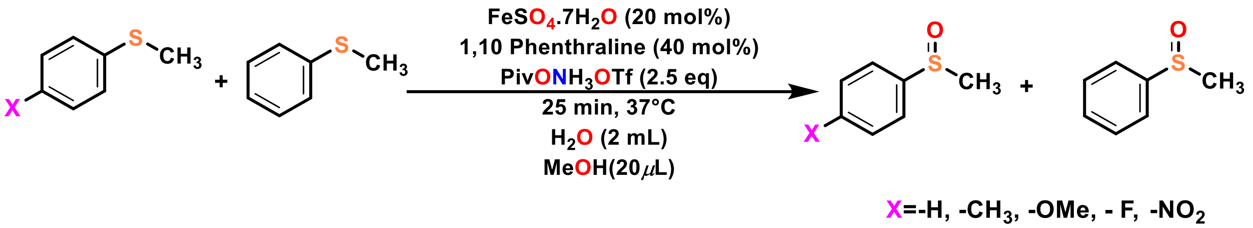

Scheme S45. Competitive reaction between Methyl phenyl sulfide and *p*-X-substituted Methyl phenyl sulfide in the iron catalyzed sulfide oxidation reaction

A clean screw cap vial was equipped with a magnetic bar in a stirrer. Then $FeSO_4.7H_2O$ (11 mg, 0.04 mmol, 20 mol%) and 1,10-phenanthroline (L1) (15.8 mg, 0.08 mmol, 40 mol%) was added followed by immediate addition of solid oxidant $PivONH_3OTf$ (Ox_1) (5.34 mg, 20 μM, 2.5 equiv.). 2.0 mL of H_2O and 20 μL CH_3OH was added via syringe followed by immediate addition of Methyl phenyl sulfide (23.5 μL, 0.2 mmol) substrate and *p*-X-substituted Methyl phenyl sulfide (0.2 mmol) substrates, simultaneously. The resulting mixture was sealed and stirred for 25 min at 37 ℃. After

25 min, the reaction mixture was diluted with saturated $NaHCO_3$ (2 mL) and 1,3,5-trimethoxybenzene (33.6 mg, 0.2 mmol,1 equiv. w.r.t. the substrate was added as ^{1}H NMR internal standard and stirred for 20 mins. The water phase was then extracted with CH_2Cl_2 (3 x 5 mL). The combined organic phases were washed with brine and dried over anhydrous Na_2SO_4, filtered, and concentrated in *vacuo*.

The relative yield of Methyl phenyl sulfoxide product and *p*-X-substituted Methyl phenyl sulfoxide was determined by ^{1}H NMR.

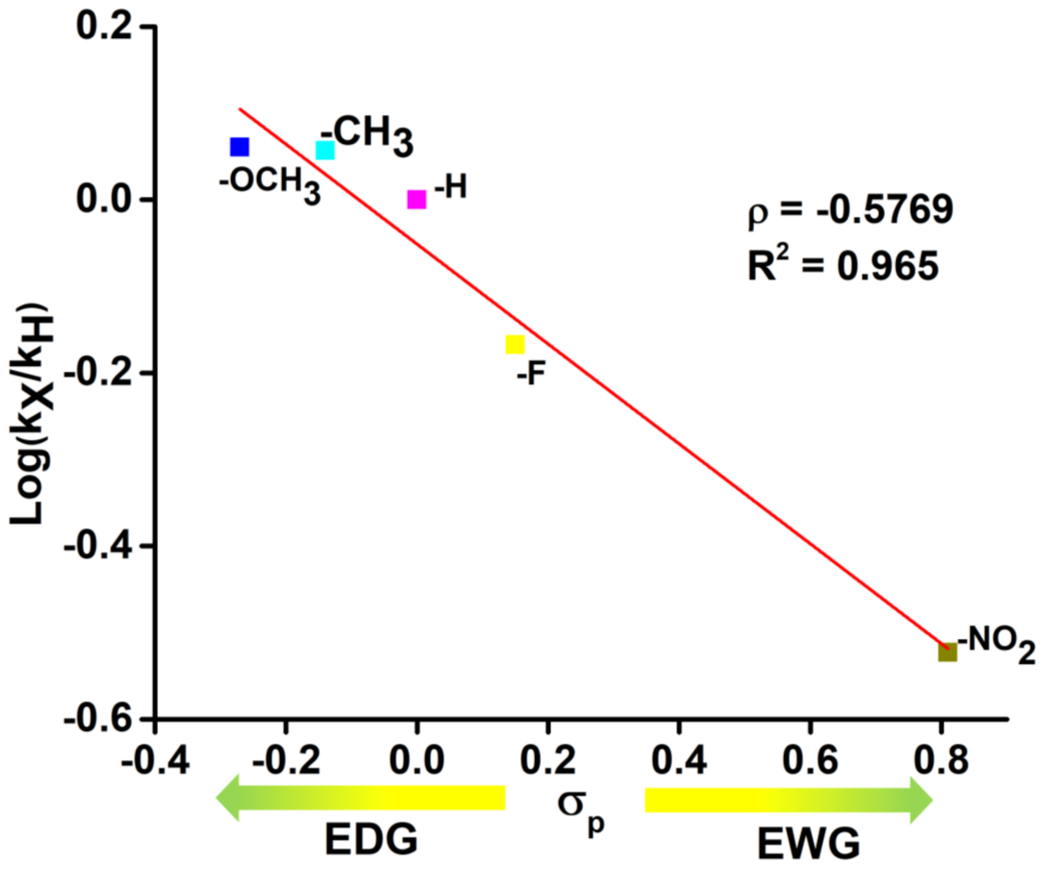

Figure S17. Hammett plot of log k_{rel} (k_{rel} = k_X/k_H) versus σ_p for *p*-$XPhSCH_3$. The k_{rel} value was calculated by dividing the concentration of the product from *p*-$XPhSCH_3$ by the concentration of the product from $PhSCH_3$.

Solvent free condition: Mechanochemical Reaction (XXI):

1) Mechanical grinding in mortar pestle

To a mortar, $FeSO_4.7H_2O$ (11 mg, 0.04 mmol, 20 mol%) and 1,10 phenanthroline (L1) (15.8 mg, 0.08 mmol, 40 mol%) was added followed by addition of solid oxidant $PivONH_3OTf$ (Ox_1) (133.6 mg, 0.5 mmol, 2.5 equiv.) and Methyl phenyl sulfide (23.5 μL, 0.2 mmol) substrate one after the other respectively. Now continuous grinding by pestle for N hours were performed. After n hr the reaction mixture was quenched by diluting with saturated $NaHCO_3$ (2 mL) and then the resulting mixture was transferred to a separatory funnel and the water phase was extracted with CH_2Cl_2 (3 x 5 ml). The combined organic phases were washed with brine and dried over Na_2SO_4, filtered, now 1,3,5-trimethoxybenzene (33.6 mg, 0.2mmol,1equiv.) (^{1}H NMR internal standard) was added to it and mixture was concentrated in *vacuo*. The yield of Methyl phenyl sulfoxide product (1b) was determined by ^{1}H NMR with respect to 1,3,5-trimethoxybenzene as internal standard.

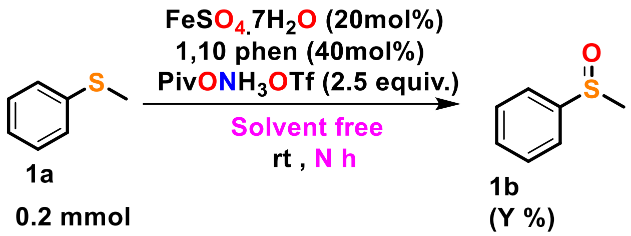

Scheme S46. Reaction of iron catalyzed sulfide oxidation under solvent free condition using mechanochemical process.

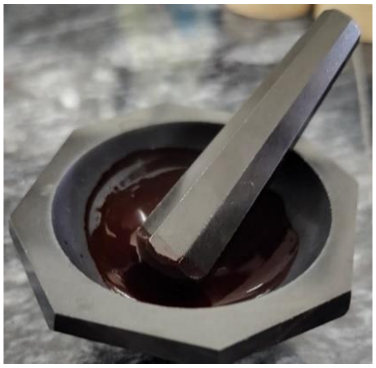

Figure S18. Set up of mortar pestle for mechanochemical grinding.

Table S14. Reaction of iron catalyzed sulfide oxidation under solvent free condition using mechanochemical process.

Entry	Time N (h)[a]	Product (1b) (Y) %
1.	2	13 %
3.	4	26 %
4.	6	58 %
5.	6	72% [b]

a) $FeSO_4.7H_2O$ (11 mg, 0.04 mmol, 20 mol%) and 1,10-phenanthroline (L1) (15.8 mg, 0.08 mmol, 40 mol%) Methyl phenyl sulfide (1a) (23.5 μL, 0.20 mmol) and $PivONH_3OTf$ (133.6 mg, 0.50 mmol) under solvent free condition. b) 1 equiV. H_2O (1 μL) was used as a reactant under standard reaction condition.

2) Mechanical grinding in Ball Milling:

To a cleaned 10 mL Retsch stainless steel milling jar, was charge with $FeSO_4.7H_2O$ (11 mg, 0.04 mmol, 20 mol%) and 1,10 phenanthroline (15.8 mg, 0.08 mmol, 40 mol%) was added followed by immediate addition of solid oxidant $PivONH_3OTf$ (133.6 mg, 0.5 mmol, 2.5 equiv.) and Methyl phenyl sulfide (23.5 μL, 0.2 mmol) substrate one after the other respectively and stainless steel grinding balls.The jar was closed and mounted to the ball mill and milled at 30 Hz for 2 h. After the completion of the reaction, the jar was removed from the mixer mill and the reaction mixture was quenched by diluting with saturated $NaHCO_3$ (2 mL) and then the resulting mixture was transferred to a separatory funnel and the water phase was extracted with CH_2Cl_2 (3 x 5 ml). The combined organic phases were washed with brine and dried over Na_2SO_4, filtered, now 1,3,5-trimethoxybenzene (33.6 mg, 0.2mmol,1equiv.) (^{1}H NMR internal standard) was added to it and mixture was concentrated in *vacuo*. The yield of Methyl phenyl sulfoxide product (1b) was determined by ^{1}H NMR with respect to 1,3,5-trimethoxybenzene as internal standard.

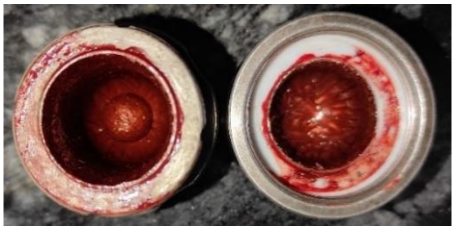

Figure S19. Ball mill used for the iron catalyzed sulfide oxidation reaction.

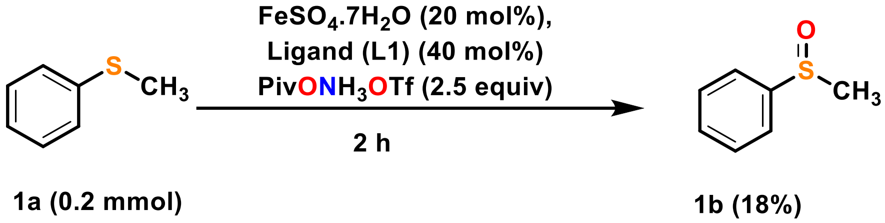

Scheme S47. Reaction of iron catalyzed sulfide oxidation under solvent free condition using ball milling method.

Procedure for cell viability analysis (XXII):

The compatibility of the synthesized molecules was assessed over HEK-293 cells with the help of 3-(4,5-dimethylthiazolyl-2)-2,5-diphenyltetrazolium bromide (MTT) assay. Briefly, 10,000 cells per well were seeded in 96 well culture plates and allowed to grow for 24 h. Different concentrations of all the synthesized molecules were prepared in DMSO (0.5% v/v) and further diluted with a cell culture medium to get the working concentrations. All the prepared solutions were added to the cells and incubated for 48 h. Cells were washed twice in PBS and further incubated with 20 µL (5 mg/mL) MTT in fresh medium for 4 h at 37 °C followed by solubilization of formazan crystals in DMSO. The absorbance was measured at 570 nm using a microplate reader. Cell viability was calculated by comparing the absorbance of treated cells relative to the untreated cells (control). DMSO (0.5%) was taken as control.

From the obtained results, all the modified precursors were found to be highly biocompatible in nature, where the calculated IC50 was observed > 100 μM.

Data represented as mean ± SD (n = 3). Statistical significance was evaluated using 1 sample t-test. $^{***}p < 0.001$.

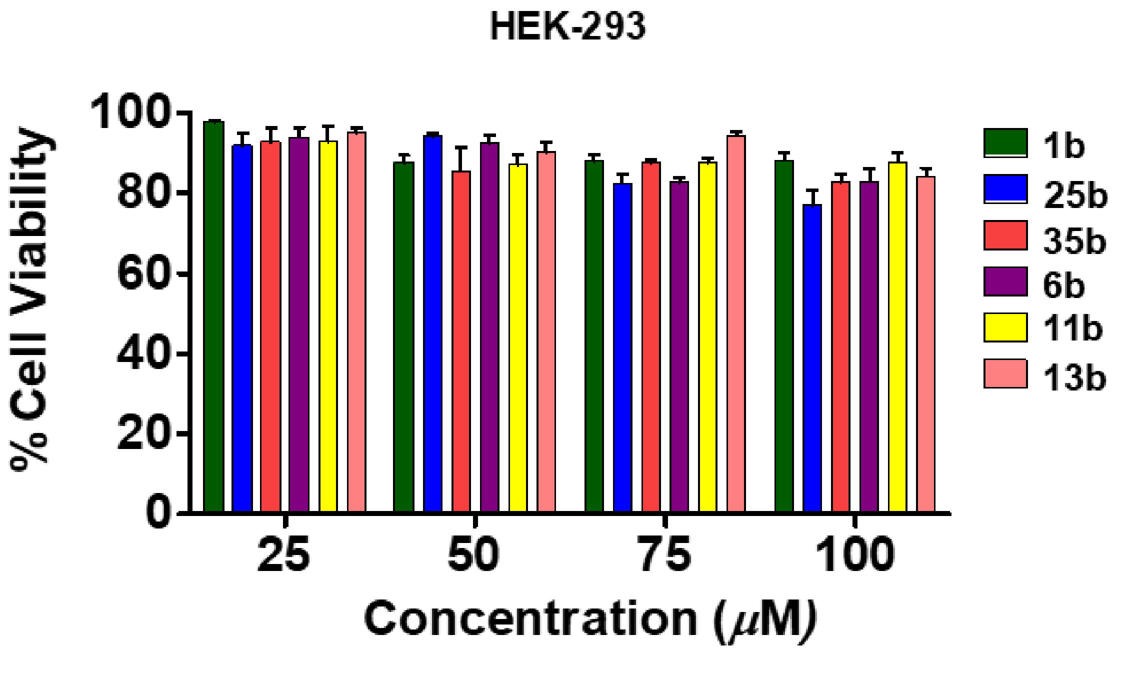

Figure S20. Plot of % cell viability vs concentration of the product derived from iron catalysed oxidation of sulphide with $PivONH_3OTf$. (All isolated purified products were used to study their biocompatibility over a wide range of concentration in HEK cell line.

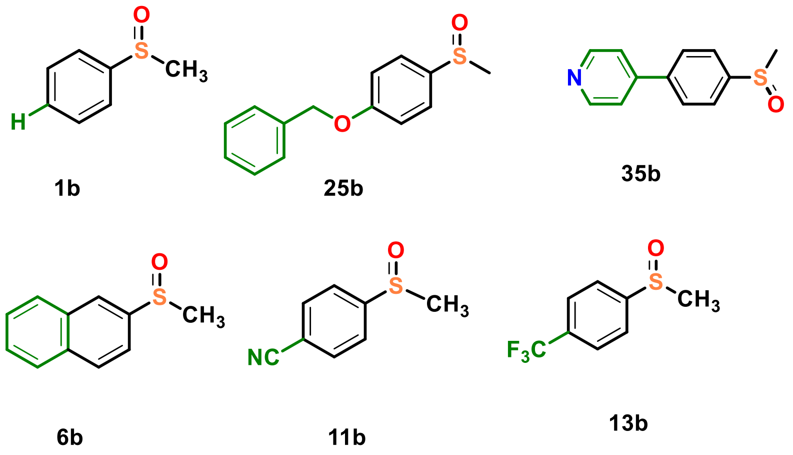

Scheme S48. Substrates used for biocompatibility study in HEK cell line.

Antioxidant Study of Selected Sulfoxides: Using FRAP Assay (XXIII):

Antioxidant properties of the synthesized sulfoxides (1b, 3b, 6b, 10b, 11b, 12b, 14b, 23b, 25b, 29b) is measured by the ferric-reducing/ antioxidant power (FRAP) assay. While the conventional FRAP assay uses tripyridyltriazine (TPTZ) complexation with iron, ferricyanide which is more recently used as a FRAP reagent was chosen and subsequent formation of the Prussian-blue complex $[Fe^{III}_4(Fe^{II}(CN)_6)_3]$ was monitored.

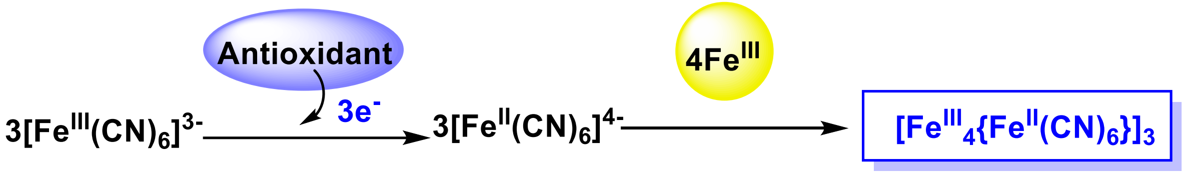

Scheme S49. Spectrophotometric analyses of antioxidant property measurement principle using FRAP assay.

Procedure: Stock solutions of 0.2M phosphate buffer saline (PBS) solution (pH=6.6), 1% potassium ferricyanide solution $[K_3Fe(CN)_6]$, 10% trichloroacetic acid solution and 1% ferric chloride solution $[FeCl_3]$ were prepared. To 2mM concentration of each isolated sulfoxides used as potent antioxidant (Scheme SXX), 50 μL of PBS, 50 μL of the prepared stock $K_3Fe(CN)_6$ solution, 50 μL of trichloroacetic acid(TCA) solution and 10 μL of $FeCl_3$ solution were added sequentially. Each of PBS, $K_3Fe(CN)_6$, TCA and $FeCl_3$ were diluted 500 times during addition making the total volume of the mixture upto 8 mL. The concentration of the antioxidant was chosen

such that it is ten times excess the concentration of the ferricyanide ions. Antioxidants were dissolved in 5 μL of CH_3OH or CH_2Cl_2 and incubated over a period of 24 hours, 48 hours and 72 hours. Corresponding UV-Vis spectra was recorded. Peak was observed near 720 nm, which matched with the reported peak for Prussian blue. Control studies in absence of any sulfoxides were also recorded as standard blanks. Quantification of the generated Prussian blue and subsequent reducing power of the sulfoxide were analysed spectrophotometrically to probe their potential antioxidant property. The following synthesized compounds were analysed for their antioxidant property:

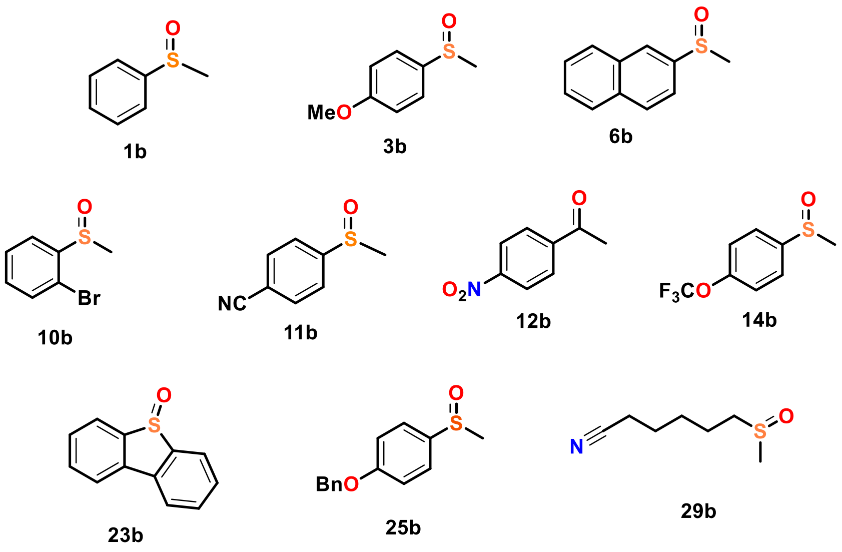

Scheme S50. Sulfoxides used for antioxidant study.

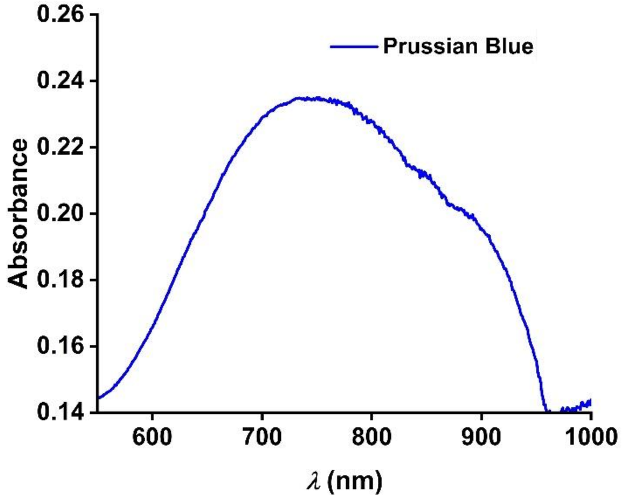

Figure S21. Generation of characteristic Prussian blue peak at 720 nm using different sulfoxides and FRAP assay.

Polarimeter data (XXIV):

A clean screw cap vial was equipped with a magnetic bar in a stirrer. Then $FeSO_4.7H_2O$ (11 mg, 0.04 mmol, 20 mol%) and chiral ligand (**L12**) (24.09 mg, 0.08 mmol, 40 mol%) was added followed by immediate addition of solid oxidant $PivONH_3OTf$ (133.6 mg, 0.5 mmol, 2.5 equiv.). 2.0 mL of H_2O and 20 μL CH_3OH was added via syringe followed by immediate addition of respective sulfide (0.2 mmol) substrate. The resulting mixture was sealed and stirred for 18 h at 37℃. After 18 hr, the reaction mixture was diluted with saturated $NaHCO_3$ (2 mL) and 1,3,5-trimethoxybenzene (33.6 mg, 0.2 mmol,1 equiv.) w.r.t. the substrate was added as ^1H NMR internal standard and stirred for 20 mins. The water phase was then extracted with CH_2Cl_2 (3 x 5 mL). The combined organic phases were washed with brine and dried over anhydrous Na_2SO_4, filtered, and concentrated in *vacuo*. The sulfoxide product (1b) thus formed was analyzed by polarimeter.

Similar procedure was followed with the achiral ligand (**L1**) for comparison.

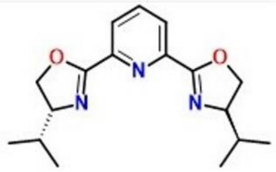

L12

This sample was measured on an Autopol V Plus, Serial #85232
Manufactured by Rudolph Research Analytical, Hackettstown, NJ, USA.

Set Temperature : 25.0
Temperature Correction : Sucrose

n	Average	Std.Dev.	Maximum	Minimum
5	5.761	0.3568	6.259	6.259

S.No	Sample ID	Time	Result	Scale	OR °Arc	WLG	Conc.	Temp.
1	as-376	02:56:52 PM	5.809	SR	0.058	589	1.000	25.0
2	as-376	02:57:02 PM	6.259	SR	0.063	589	1.000	25.0
3	as-376	02:57:12 PM	5.262	SR	0.053	589	1.000	25.0
4	as-376	02:57:22 PM	5.671	SR	0.057	589	1.000	25.0
5	as-376	02:57:32 PM	5.803	SR	0.058	589	1.000	25.0

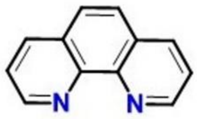

L1

This sample was measured on an Autopol V Plus, Serial #85232
Manufactured by Rudolph Research Analytical, Hackettstown, NJ, USA.

Set Temperature : 25.0
Temperature Correction : Sucrose

n	Average	Std.Dev.	Maximum	Minimum
5	3.809	0.1808	4.033	4.033

S.No	Sample ID	Time	Result	Scale	OR °Arc	WLG	Conc.	Temp.
1	AS-244	03:51:12 PM	4.033	SR	0.040	589	1.000	25.0
2	AS-244	03:51:22 PM	3.555	SR	0.036	589	1.000	25.0
3	AS-244	03:51:32 PM	3.869	SR	0.039	589	1.000	25.0
4	AS-244	03:51:43 PM	3.716	SR	0.037	589	1.000	25.0
5	AS-244	03:51:53 PM	3.871	SR	0.039	589	1.000	25.0

Application of the developed methodology (XXV):

Molecules **33b** and **36b** were synthesized using general procedure H. To further demonstrate the utility and effectiveness of this developed methodology we further explored its applications for the synthesis of 2,2'-Bipyridine (L5) and terpyridine (L6). These are important ligands in coordination chemistry having ability to form stable complexes with various metal ions.

General procedure for the coupling reaction of pyridyllithium and heteroaromatic sulfoxide:

To a stirred solution of bromopyridine (1 equiv.) in a mixture of ether, hexane, and THF (12:6:6, 24 mL) was added dropwise n-BuLi (1.66 M in hexane solution, 1.1 equiv.) at -78 °C during 5-10 min. To the resulting yellow solution was added pyridine-2- sulfoxide (1 equiv.) (**33b)** (Scheme S51) or bipyridine sulfoxide **(36b)** (Scheme S52) (isolated following the standard procedure) in THF (2 mL) drop-by-drop at the same temperature. After being stirred overnight, the reaction mixture was quenched with water (5 mL) and extracted with EtOAc (150 mL). The organic layer was washed with water and brine and dried over Na_2SO_4. Solvent was evaporated under reduced pressure to form the product **L5** from sulfoxide **33b** and **L6** from **36b**.[5]

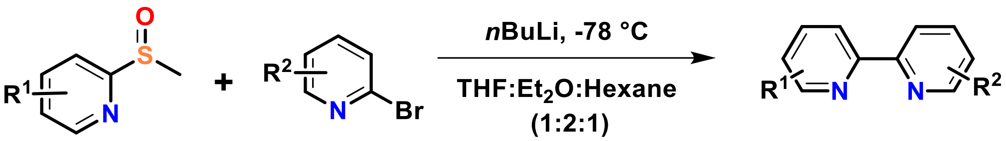

Scheme S51. General procedure for the coupling reaction of pyridyllithium and heteroaromatic sulfoxide.

1.Synthesis of 2,2'-Bipyridine

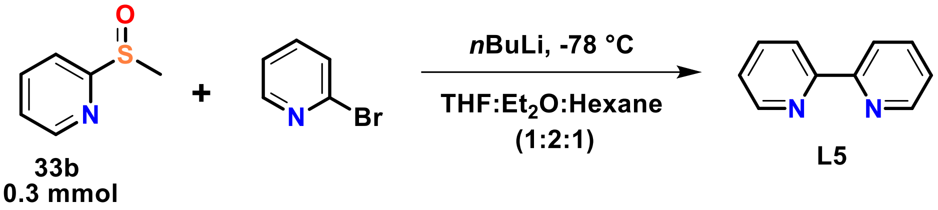

Scheme S52. Synthesis procedure for bipyridine.

2. Synthesis of terpyridine

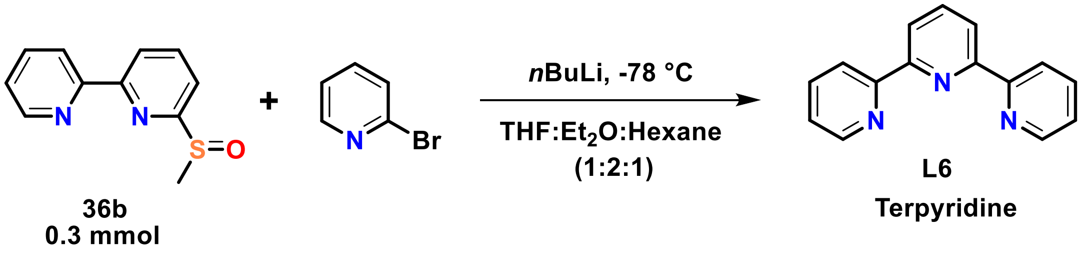

Scheme S53. Synthesis procedure for terpyridine.

3(a). Transition-metal-free formal cross-coupling of aryl methyl sulfoxides and alcohols via nucleophilic activation of C-S bond.

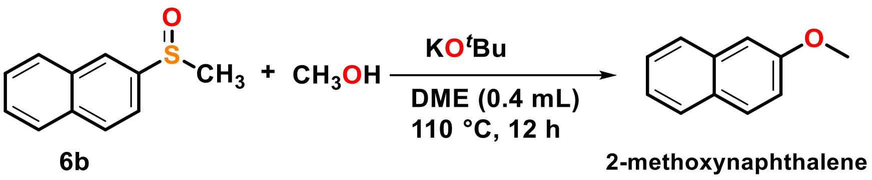

Scheme S54. Transition-metal free formal cross-coupling of methyl 2-naphthyl sulfoxide and methanol.

Methyl 2-naphthyl sulfoxide (**6b**) isolated and purified using standard reaction (See section XXX) was taken (19.0 mg, 0.1 mmol, 1 equiv.) under inert atmosphere in an oven-dried vial equipped

with a stir bar. To it was added KO*t*Bu (22.5 mg, 0.2 mmol) under inert atmosphere. DME (0.4 mL) was added to the vial by syringe. Then, methanol (8.1 μL, 0.2 mmol, 2 equiv.) was added by syringe under nitrogen atmosphere. The reaction mixture was heated to 110 °C in an oil bath and stirred for 12 h. Upon completion of the reaction, the sealed vial was cooled to room temperature and opened to air. The reaction mixture was passed through a short pad of silica gel. The pad was then rinsed with 10:1 dichloromethane: methanol. The resulting solution was subjected to reduced pressure to remove the volatile materials and yielded a viscous oil. The residue was purified by flash chromatography to isolate white solid whose NMR matched with literature reported 2-methoxynaphthalene.[6]

3(b). Transition-metal free formal cross-coupling of methylsulfinyl benzene (1b) and 2-cyclohexyl ethanol.

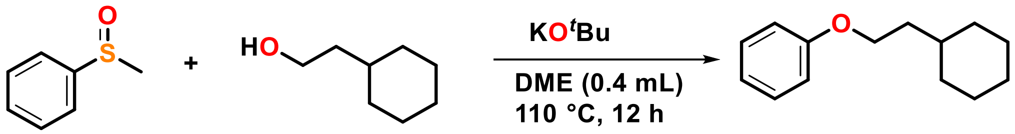

(2-cyclohexylethoxy)benzene

1b

Scheme S55. transition-metal free formal cross-coupling of methylsulfinyl benzene and 2-cyclohexyl ethanol.

Methyl phenyl sulfoxide (**1b**) was isolated and purified following the standard procedure. To an oven-dried vial equipped with a stir bar was added KO*t*Bu (67.3 mg, 0.6 mmol, 3 equiv.), and Methyl phenyl sulfoxide (**1b**) (28.0 mg, 0.2 mmol, 1 equiv.) under inert atmosphere. DME (0.4 mL) was added to the vial by syringe. Then, 2-cyclohexyl ethanol (84.5μL, 0.6 mmol, 3 equiv.) was added by syringe under nitrogen atmosphere. Note that solid and viscous oil alcohols were added to the reaction vial prior to KO*t*Bu. The reaction mixture was heated to 110 °C in an oil bath and stirred for 12 h. Upon completion of the reaction, the sealed vial was cooled to room temperature and opened to air. The reaction mixture was passed through a short pad of silica gel. The pad was then rinsed with 10:1 dichloromethane: methanol. The resulting solution was subjected to reduced pressure to remove the volatile materials and yielded a viscous oil. The residue was purified by flash chromatography to isolate solid product whose NMR matched with literature reported (2-cyclohexylethoxy)benzene.[6]

3(c). Synthesis of biologically relevant molecules:

Step-1. Transition-metal free formal cross-coupling of methylsulfinyl benzene and 2-aminoethanol.

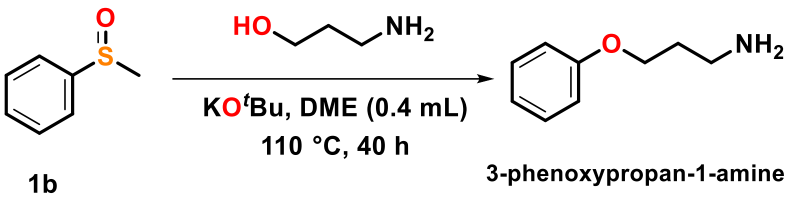

Scheme S56. Transition-metal free formal cross-coupling of methylsulfinyl benzene and 2-aminoethanol.

To an oven-dried vial equipped with a stir bar was added KO*t*Bu (169.83 mg, 1.5 mmol, 3 equiv.), and methanesulfinyl-benzene (70.1 mg, 0.5 mmol, 1 equiv.) under inert atmosphere. DME (2 mL) was added to the vial by syringe. Then, 2-aminoethanol (114.7 μL, 1.5 mmol, 3 equiv.) was added by syringe under nitrogen atmosphere. Note that solid and viscous oil alcohols were added to the reaction vial prior to KO*t*Bu. The reaction mixture was heated to 110 °C in an oil bath and stirred for 40 h. Upon completion of the reaction, the sealed vial was cooled to room temperature and opened to air. The reaction mixture was passed through a short pad of silica gel. The pad was then rinsed with 10:1 dichloromethane: methanol. The resulting solution was subjected to reduced pressure to remove the volatile materials and yielded a viscous oil. The residue was purified by flash chromatography to isolate yellow oil whose NMR matched with 3-phenoxy propane-1 amine.[6]

Step-2. Synthesis of (5-Nitro-pyridin-2-yl)-(3-phenoxy-propyl)-amine

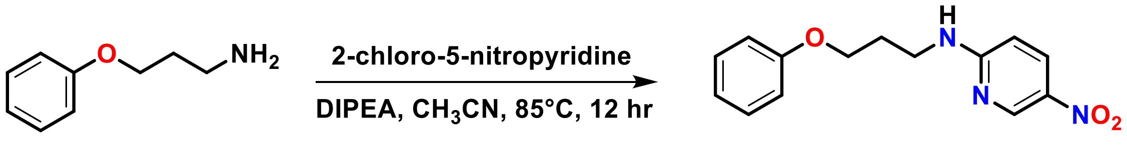

Scheme S57. Synthesis of (5-Nitro-pyridin-2-yl)-(3-phenoxy-propyl)-amine

In a Schlenk tube charged with a stir bar was added 3-phenoxy-propylamine (0.2 mmol, 31.2 mg), 2-Chloro-5-nitropyridine (0.24 mmol, 38.0 mg), DIPEA (0.4 mmol, 51.7 mg) and CH_3CN (2.0 mL, 0.1 M). The mixture was then heated to 85°C and kept stirring for 12 h. The solvent was removed

under vacuum. The crude product was purified by flash chromatography on silica gel (eluted with DCM) to give the product as a yellow-green solid whose NMR matched with the literature reported.[6] The compound (5-Nitro-pyridin-2-yl)-(3-phenoxy-propyl)-amine can further be transformed to the biological probe N^2-(3-Phenoxy-propyl)-pyridine-2,5-diamine using a standard amine nitro group reduction (Scheme S58).

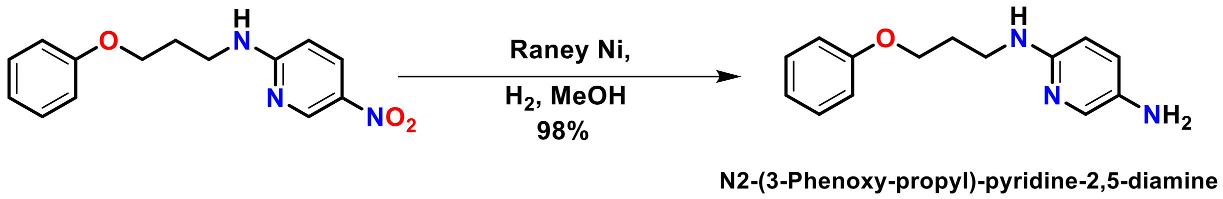

Scheme S58. Synthesis of N^2-(3-Phenoxy-propyl)-pyridine-2,5-diamine

EPR Experiments (XXVI):

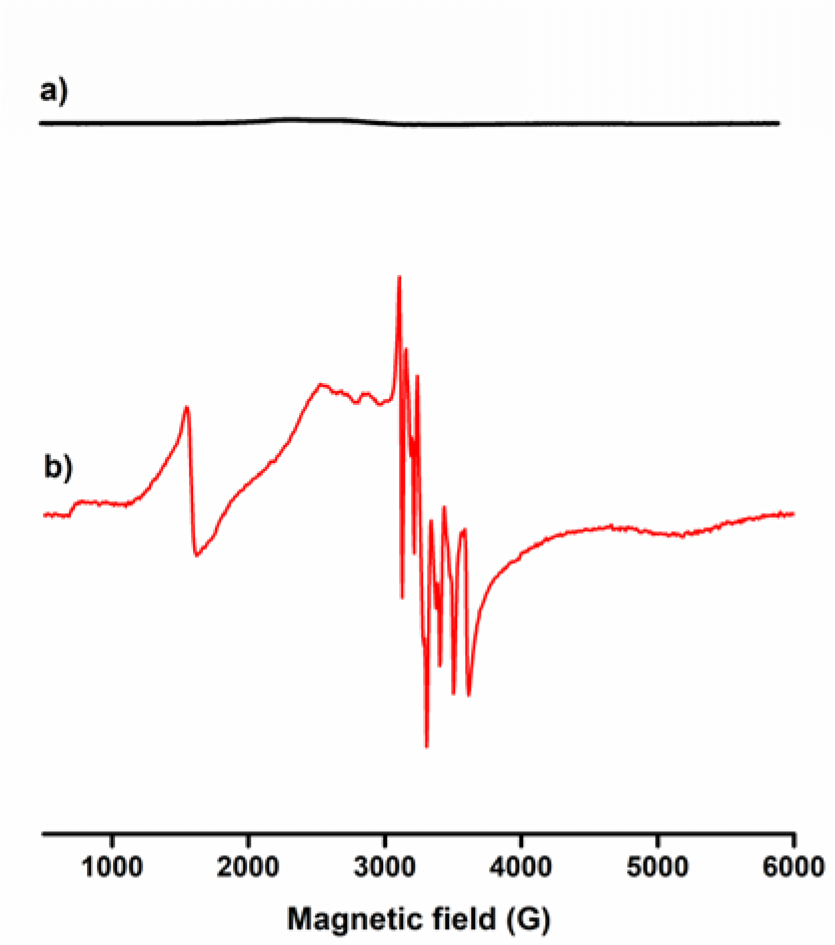

Figure S22. X-band EPR spectra (T=100 K) of (**a**) $FeSO_4.7H_2O$ (11 mg, 0.04 mmol) and ligand L1 (15.8 mg, 0.08 mmol) dissolved in a mixture of DCM and MeOH. (**b**) $FeSO_4.7H_2O$ (11 mg, 0.04 mmol), ligand L1 (15.8 mg, 0.08 mmol), and $PivONH_3OTf$ (133.6 mg, 0.5 mmol) and Methyl phenyl sulfide (1a) (23.5 μL, 0.20 mmol) added one after the other followed by 0.8 mL of H_2O.

GC-MS spectra (XXVII):

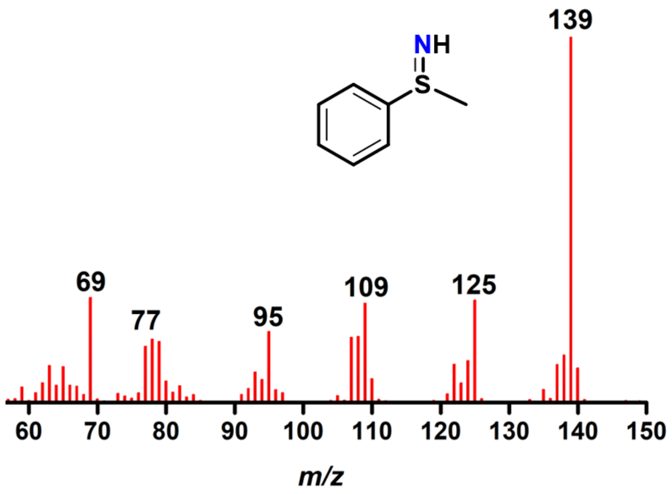

Figure S23. GC-mass spectrum of the Sulfilimine product derived after mixing $FeSO_4.7H_2O$+L1+$PivONH_3$ (2.5 equiv.+ Methyl phenyl sulfide in H_2O (2 mL): MeOH (20 μL).

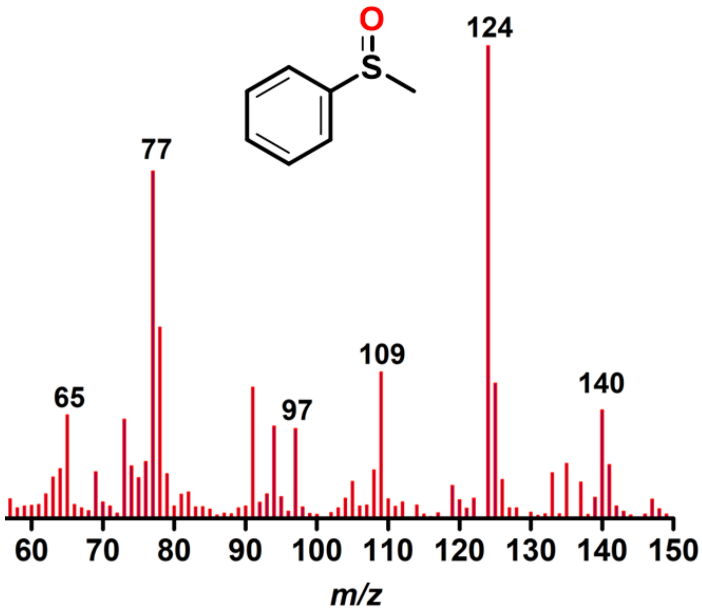

Figure S24. GC-mass spectrum of the sulfoxide product derived after mixing $FeSO_4.7H_2O$+L1+$PivONH_3$ (2.5 equiv + Methyl phenyl sulfide in H_2O (2 mL): MeOH (20 μL) in 18 h.

Unsuccessful substrates (XXVIII):

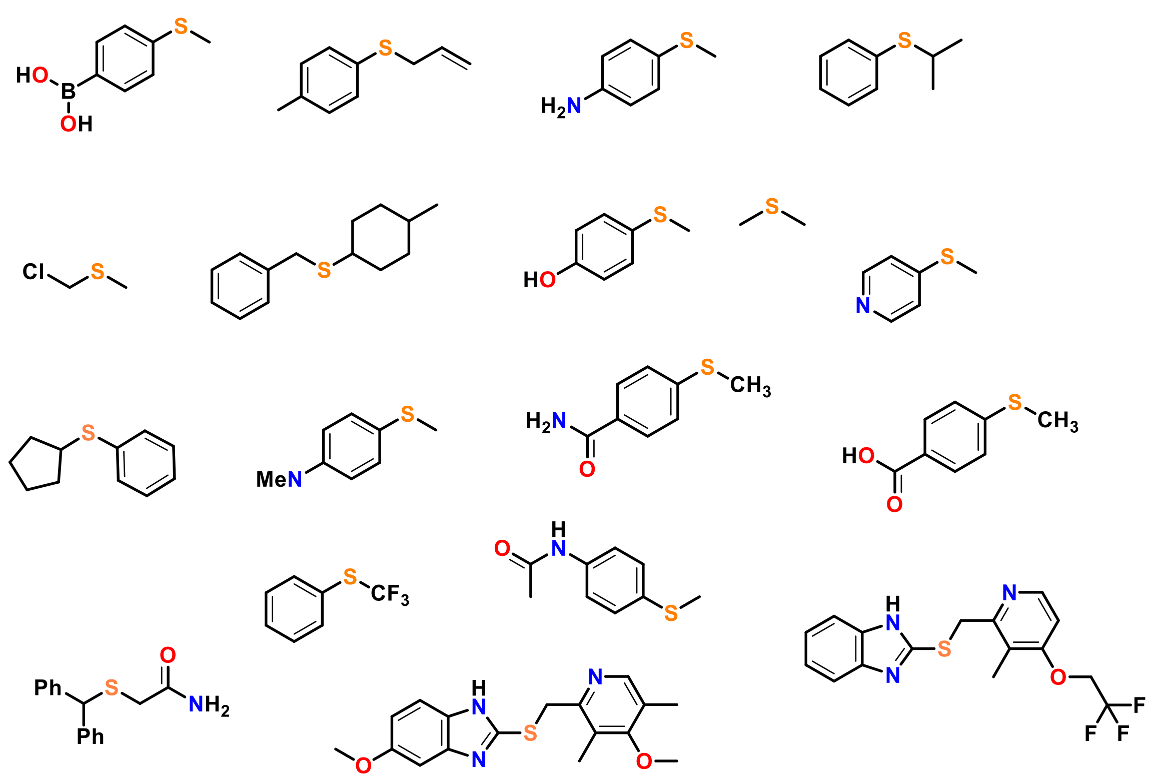

Scheme S59: Unsuccessful substrates for iron catalyzed sulfide oxidation.

Crystallographic data (XXIX):

1. **Dibenzothiophene sulfoxide (23b)**

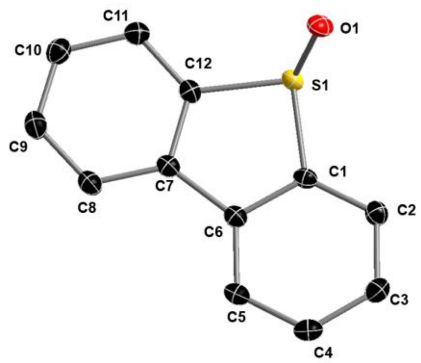

Table S15. Crystallographic parameters for (23b)

Empirical formula	$C_{12}H_8OS$
Formula weight	200.24 g·mol^{-1}
Color	Red
Temperature	100 K
Wavelength	0.71073 Å
Crystal system	Triclinic
Space group	P-1
a	8.4786(2) Å
b	9.4229(2) Å
c	12.0453(2) Å
α	97.8380°
β	106.2490°
γ	96.4380°
Volume	903.76(3)Å^3
Z	4
Density (calculated)	1.472 g/cm^{-3}
Absorption coefficient	0.313 mm^{-1}
F(000)	416.0
Crystal size	0.64 × 0.172 × 0.11 mm^3
range for data collection	4.18 to 46.72°
Completeness	81.2 %
Index ranges	-10 ≤ h ≤ 10, -13 ≤ k ≤ 13, -15 ≤ l
Reflections collected	34091
Independent reflections	4259 [R_{int} = 0.0478, R_{sigma} = 0.0290]
Data / restraints / parameters	4259/0/253
Goodness-of-fit on F^2	1.127
Final R indices [I>2σ(I)]	R_1 = 0.0359, wR_2 = 0.1054
R indices (all data)	R_1 = 0.0392, wR_2 = 0.1081
Largest diff. peak and hole	e Å^{-3} 0.62/-0.43
Extinction coefficient	n/a
CCDC	2405157

2. **Naphthalene Sulfoxide (6b)**

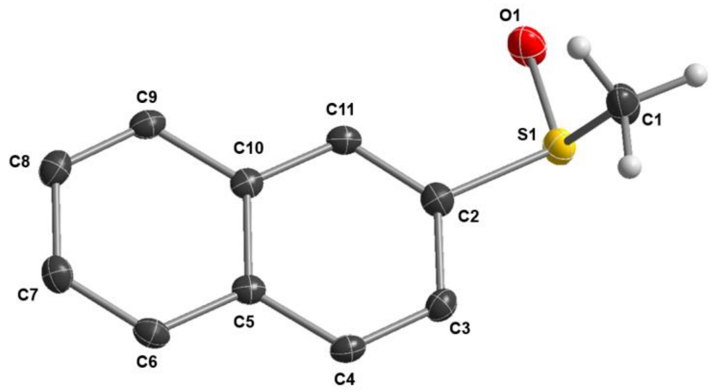

Table S16. Crystallographic parameters for (6b)

Empirical formula	$C_{11}H_{10}OS$
Formula weight	190.267 g·mol^{-1}
Color	Red
Temperature	100 K
Wavelength	0.71073 Å
Crystal system	orthorhombic
Space group	Pbca
a	5.8016(2) Å
b	8.0220(3) Å
c	38.9098(13) Å
α	90°
β	90°
γ	90°
Volume	1810.88(11) Å^3
Z	8
Density (calculated)	1.396 g/cm^{-3}
Absorption coefficient	0.308 mm^{-1}
F(000)	801.393
Crystal size	0.168 x 0.105 x 0.05
range for data collection	4.18 to 46.72°
Completeness	99.9 %
Index ranges	-6 ≤ h ≤ 6, -8 ≤ k ≤ 7, -43 ≤ l ≤ 43
Reflections collected	29632
Independent reflections	1323 [R_{int} = 0.0691, R_{sigma} = 0.0212]

Data / restraints / parameters	1323/0/119
Goodness-of-fit on F^2	1.051
Final R indices [I>2σ(I)]	R_1 = 0.0318, wR_2 = 0.0819
R indices (all data)	R_1 = 0.0345, wR_2 = 0.0836
Largest diff. peak and hole	e·$Å^{-3}$ 0.35/-0.27
Extinction coefficient	n/a
CCDC	2405155

3. 4-nitrilephenylmethyl sulfoxide (11b)

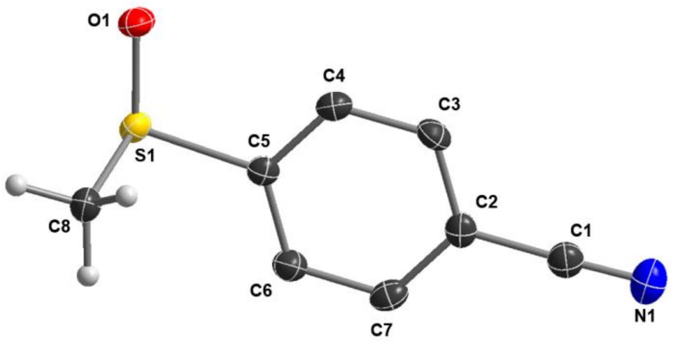

Table S17. Crystallographic parameters for (11b)

Empirical formula	$C_8 H_7$ NOS
Formula weight	165.21 g·mol^{-1}
Color	Red
Temperature	100 K
Wavelength	0.71073 Å
Crystal system	triclinic
Space group	*P-1*
a	6.429 Å
b	7.427 Å
c	8.386 Å
α	98.02°.
β	97.00°.
γ	99.55°.

Volume	386.7 Å^3
Z	2
Density (calculated)	1.419 g/cm^3
Absorption coefficient	0.352 mm^{-1}
F(000)	172.0
Crystal size	0.153 × 0.1 × 0.05 mm^3
range for data collection	4.96 to 50.218°
Completeness	58 %
Index ranges	-6 ≤ h ≤ 6, -7 ≤ k ≤ 8, -9 ≤ l ≤ 9
Reflections collected	1343
Independent reflections	804 [R_{int} = 0.0249, R_{sigma} = 0.0384]
Data / restraints / parameters	804/0/101
Goodness-of-fit on F^2	1.089
Final R indices [I>2σ(I)]	R_1 = 0.0346, wR^2 = 0.0887
R indices (all data)	R_1 = 0.0387, wR^2 = 0.0937
Largest diff. peak and hole	0.24/-0.27 e·Å^{-3}
Extinction coefficient	n/a
CCDC	2405154

4. 4-Chloro-phenylmethyl sulfoxide (8b)

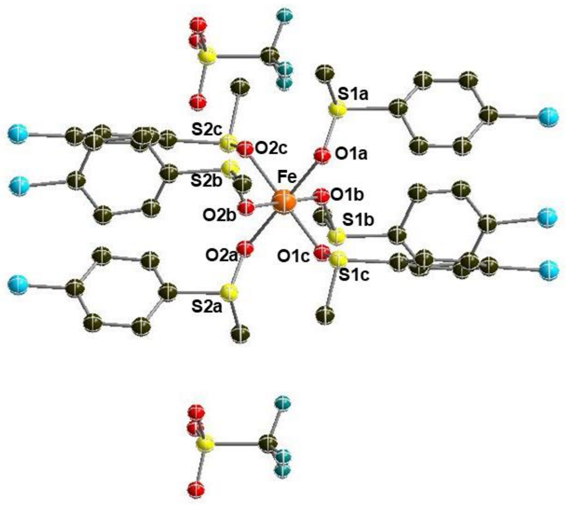

Table S18. Crystallographic parameters for (8b)

Empirical formula	$C_{48}H_{48}Cl_{48}FFeO_{48}S_{48}$
Formula weight	4708.19 g·mol^{-1}
Color	White
Temperature	100 K
Wavelength	0.71073 Å
Crystal system	trigonal
Space group	R3
a	12.504(3)Å
b	12.504(3)Å
c	31.760(6)Å
α	90°
β	90°
γ	120°
Volume	4301(2)Å^3
Z	1
Density (calculated)	1.818 g/cm^3
Absorption coefficient	1.483 mm^{-1}
F(000)	2339.0
Crystal size	0.16 × 0.11 × 0.94 mm^3
range for data collection	3.848 to 54.97
Completeness	92 %
Index ranges	-16 ≤ h ≤ 15, -16 ≤ k ≤ 13, -
Reflections collected	30429
Independent reflections	4058 [R_{int} = 0.1060, R_{sigma} = 0.0503]
Data / restraints / parameters	4058/1/234
Goodness-of-fit on F^2	1.933
Final R indices [I>2σ(I)]	R_1 = 0.1405, wR_2 = 0.4076
R indices (all data)	R_1 = 0.1618, wR_2 = 0.4368
Largest diff. peak and hole	e·Å^{-3} 6.67/-4.51
Extinction coefficient	n/a
CCDC	2405156

Table S19. Bond angle of $[Fe(S)_6(OTf)_2]$ (S = 8b)

Atoms	Bond Angles/°
O2a-Fe-O1a	179.8(9)
O2b-Fe-O1b	179.8(9)
O2c-Fe-O1c	179.8(9)
O1a-Fe-O1b	92.2(7)
O1b-Fe-O1c	92.2(7)
O1c-Fe-O1a	92.2(7)
O2a-Fe-O2b	92.0
O2b-Fe-O2c	92.0
O2c-Fe-O2a	92.0
O1a-Fe-O2c	88.0(6)
O1a-Fe-O2b	87.7(6)
O1b-Fe-O2c	88.0(6)
O1b-Fe-O2a	87.7(6)
O1c-Fe-O2a	88.0(6)
O1c-Fe-O2b	87.7(6)

Table S20. Bond length of $[Fe(S)_6(OTf)_2]$ (S = 8b)

Atoms	Bond Lengths/Å
Fe-O1a	2.061(17)
Fe-O1b	2.061(17)
Fe-O1c	2.061(17)
Fe-O2a	2.014(13)
Fe-O2b	2.014(13)
Fe-O2c	2.014(13)

5. **4-Bromo-phenylmethyl sulfoxide (9b)**

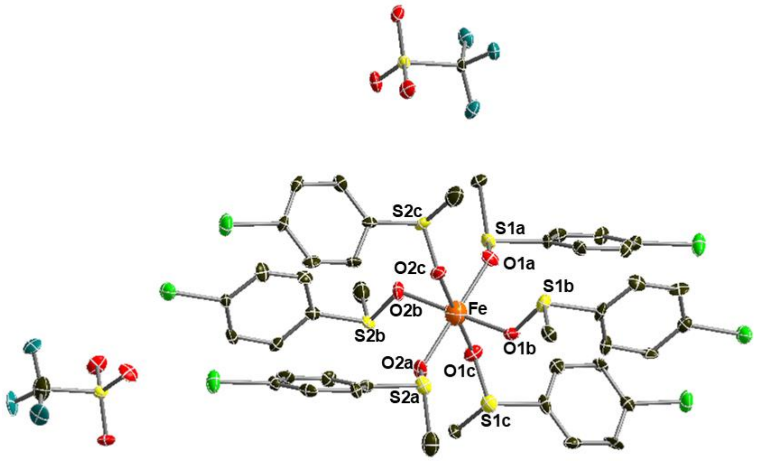

Table S21. Crystallographic parameters for 9b

Empirical formula	$C_{44}H_{42}Br_6F_6FeO_{12}S_8$
Formula weight	1668.56 g·mol^{-1}
Color	White
Temperature	100 K
Wavelength	0.71073 Å
Crystal system	trigonal
Space group	R3
a	12.5796 Å
b	12.5796 Å
c	32.0770 Å
α	90°
β	90°
γ	120°
Volume	4396.00 Å^3
Z	3
Density (calculated)	1.891 g/cm^3
Absorption coefficient	4.707 mm^{-1}
F(000)	2460.0
Crystal size	0.16 × 0.11 × 0.08 mm^3
range for data collection	3.81 to 60.49°

Completeness	96 %
Index ranges	-17 ≤ h ≤ 17, -17 ≤ k ≤ 17, -43 ≤ l ≤ 44
Reflections collected	27119
Independent reflections	5615 [Rint = 0.0298, Rsigma = 0.0246]
Data / restraints / parameters	5615/1/232
Goodness-of-fit on F^2	1.081
Final R indices [I>2σ(I)]	R1 = 0.0745, wR2 = 0.2376
R indices (all data)	R1 = 0.0810, wR2 = 0.2445
Largest diff. peak and hole	4.00/-6.38 e·$Å^{-3}$
Extinction coefficient	n/a
CCDC	2405153

Table S22. Bond angle table of $[Fe(S)_6(OTf)_2]$ (S = 9b)

Atoms	Bond Angle/°
O2a-Fe-O1a	179.3(7)
O2b-Fe-O1b	179.3(7)
O2c-Fe-O1c	179.3(7)
O1a-Fe-O1b	92.2(6)
O1b-Fe-O1c	92.2(6)
O1c-Fe-O1a	92.2(6)
O2a-Fe-O2b	91.6(7)
O2b-Fe-O2c	91.6(7)
O2c-Fe-O2a	91.6(7)
O1a-Fe-O2c	88.6(4)
O1a-Fe-O2b	87.7(5)
O1b-Fe-O2c	88.6(4)
O1b-Fe-O2a	87.7(5)
O1c-Fe-O2a	88.6(4)
O1c-Fe-O2b	87.7(5)

Table S23. Bond length of $[Fe(S)_6(OTf)_2]$ (S = 9b)

Atoms	Bond Lengths /Å
Fe-O1a	2.073(13)
Fe-O1b	2.073(13)
Fe-O1c	2.073(13)
Fe-O2a	2.032(13)
Fe-O2b	2.032(13)
Fe-O2c	2.032(13)

Synthesis and characterization of sulfoxides (XXX):

1. Methyl phenyl sulfoxide (1b)

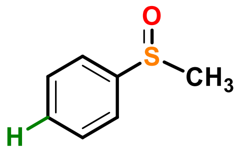

Was prepared from commercially available methyl phenyl sulfide (0.2 mmol) according to general procedure (XIX): Purification by column chromatography (SiO_2, ethyl acetate: hexane = 4:1) afforded the title compound Methyl phenyl sulfoxide (25.0 mg, 0.17 mmol, 90%) as brown liquid.

^{1}H NMR (500 MHz, $CDCl_3$) *δ* 7.63 (dd, *J* = 8.1, 1.5 Hz, 2H), 7.55 – 7.43 (m, 3H), 2.70 (s, 3H).
^{13}C NMR (126 MHz, $CDCl_3$) *δ* 145.74, 131.13, 129.45, 123.58, 44.01.
HR-MS(ESI$^+$) calc. for C_7H_8OS $[M+H]^+$ 141.0374, found 141.0380.

2. 4-Methylphenyl methyl sulfoxide (2b)

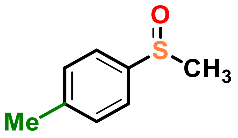

Was prepared from 4-Methylthioanisole (0.2 mmol) according to general procedure (XIX): Purification by column chromatography (SiO_2, ethyl acetate:hexane = 4:1) afforded the title compound 4-Methylphenyl methyl sulfoxide (24.48 mg, 0.16 mmol, 80%).

^{1}H NMR (500 MHz, $CDCl_3$) *δ* 7.54 (d, *J* = 8.3 Hz, 2H), 7.33 (d, *J* = 7.8 Hz, 2H), 2.70 (s, 3H), 2.42 (s, 3H).
^{13}C NMR (126 MHz, $CDCl_3$) *δ* 142.60, 141.67, 130.18, 123.68, 44.13, 21.54.
HR-MS(ESI$^+$) calc. for $C_8H_{10}OS$ $[M+H]^+$ 155.0531, found 155.0553.

3. 4-Methoxyphenyl methyl sulfoxide (3b)

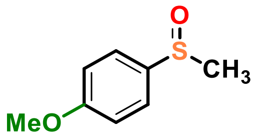

Was prepared from commercially available 4-Methoxythioanisole (0.2 mmol) according to general procedure (XIX): Purification by column chromatography (SiO_2, ethyl acetate:hexane = 4:1) afforded the title compound 4-Methoxyphenyl methyl sulfoxide (30.42 mg, 0.18 mmol, 90%).

^{1}H NMR (500 MHz, $CDCl_3$) *δ* 7.23 (t, *J* = 7.6 Hz, 1H), 7.05 (d, *J* = 7.5 Hz, 2H), 2.87 (s, 3H), 2.60 (s, 6H).

^{13}C NMR (126 MHz, $CDCl_3$) *δ* 139.21, 138.03, 130.96, 130.38,38.37, 19.18.

HR-MS(ESI$^+$) calc. for $C_8H_{11}O_2S$ [M+H]$^+$ 171.0480, found 171.0554.

4. (4-*tert*-butyl)phenylmethyl sulfoxide (4b)

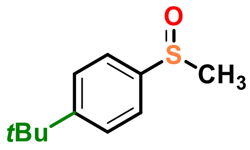

Was prepared from 4-*tert*butyl-thioanisole (0.2 mmol) according to general procedure (XIX): Purification by column chromatography (SiO_2, ethyl acetate:hexane = 4:1) afforded the title compound (4-*tert*-butyl)phenylmethyl sulfoxide (28.1mg, 0.14 mmol, 71%) as a yellow solid.

^{1}H NMR (500 MHz, $CDCl_3$) *δ* 7.59 – 7.49 (m, 4H), 2.71 (s, 3H), 1.32 (s, 9H).

^{13}C NMR (126 MHz, $CDCl_3$) *δ* 154.81, 142.45, 126.53, 123.53, 43.97, 35.11, 31.34.

HR-MS(ESI$^+$) calc. for $C_{11}H_{16}OS$ [M+H]$^+$ 197.1000, found 197.1007.

5. 2,6-Dimethylbenzenethiosulfoxide (5b)

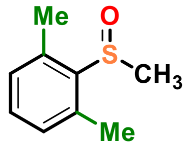

Was prepared from 2,6-Dimethylbenzenethiosulfane (0.2 mmol) according to general procedure (XIX): Purification by column chromatography (SiO_2, EtOAc:Hexane = 4:1) afforded the 2,6-Dimethylbenzenethiosulfoxide (14.9mg, 0.08mmol, 44%) as brown solid.

^{1}H NMR (500 MHz, $CDCl_3$) δ 7.23 (s, 1H), 7.05 (s, 2H), 2.87 (s, 3H), 2.60 (s, 6H).
^{13}C NMR (126 MHz, $CDCl_3$) δ 139.32, 138.04, 130.96, 130.40, 38.43, 29.84.

HR-MS(ESI$^+$) calc. for $C_9H_{12}OS$ [M+H]$^+$ 169.0687, found 169.7924.

6. (methylsulfinyl)naphthalene (6b)

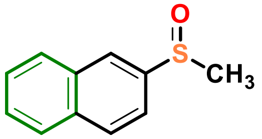

was prepared from commercially available Biphenyl sulfide (0.2 mmol) according to general procedure (XIX) at 50℃: Purification by column chromatography (SiO_2, EtOAc:Hexane = 4:1) afforded the (methylsulfinyl)naphthalene sulfoxide (24.2 mg, 0.128mmol, 62%) as a reddish brown solid.

^{1}H NMR (500 MHz, $CDCl_3$) δ 8.19 (s, 1H), 8.06 – 7.75 (m, 3H), 7.65 – 7.48 (m, 3H), 2.79 (s, 3H).
^{13}C NMR (126 MHz, $CDCl_3$) δ 142.01, 134.51, 132.93, 129.74, 128.66, 128.12, 127.95, 127.46, 124.37, 119.55, 43.49.
HR-MS(ESI$^+$) calc. for $C_{11}H_{10}OS$ [M+H]$^+$ 191.0531, found 191.0525.

7. 4-Fluorophenyl methyl sulfoxide (7b)

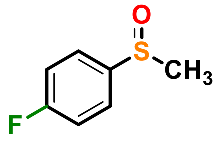

Was prepared from commercially available 4-Fluorothioanisole (0.2 mmol) according to general procedure (XIX): Purification by column chromatography (SiO_2, ethyl acetate: hexane = 4:1) afforded the title compound 4-Fluorophenyl methyl sulfoxide (25.43 mg, 0.162 mmol, 81%) as a brown solid.

^{1}H NMR (500 MHz, $CDCl_3$) *δ* 7.66 – 7.59 (m, 2H), 7.25 – 7.14 (m, 2H), 2.69 (s, 3H).
^{13}C NMR (126 MHz, $CDCl_3$) *δ* 165.35, 163.35, 141.14, 125.90, 116.76 ,44.20.
^{19}F NMR (471 MHz, $CDCl_3$) *δ* -108.40.

HR-MS(ESI$^+$) calc. for C_7H_7FOS $[M+H]^+$ 159.0280, found 159.0393.

8. 4-Chlorophenyl methyl sulfoxide (8b)

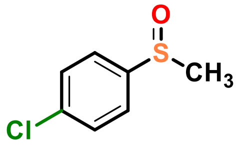

Was prepared from commercially available 4-Chlorothioanisole (0.2 mmol) according to general procedure (XIX): Purification by column chromatography (SiO_2, ethyl acetate: hexane = 4:1) afforded the title compound 4-Chlorophenyl methyl sulfoxide (27.60 mg, 0.16 mmol, 79%).

^{1}H NMR (500 MHz, $CDCl_3$) *δ* 7.58 (d, *J* = 8.7 Hz, 2H), 7.49 (d, *J* = 8.6 Hz, 2H), 2.70 (s, 3H).
^{13}C NMR (126 MHz, $CDCl_3$) *δ* 144.22, 137.34, 129.75, 125.07, 44.11.

HR-MS(ESI$^+$) calc. for C_7H_7ClOS $[M+H]^+$ 174.9984, found 175.0064.

9. **4-Bromophenyl methyl sulfoxide (9b)**

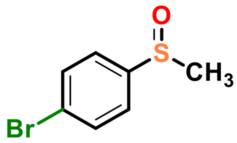

Was prepared from commercially available 4-Bromothioanisole (0.2 mmol) according to general procedure (XIX): Purification by column chromatography (SiO_2, ethyl acetate:hexane = 4:1) afforded the title compound 4-Bromophenyl methyl sulfoxide (34.88mg, 0.16 mmol, 80%).

^{1}H NMR (500 MHz, $CDCl_3$) δ 7.67 (d, *J* = 8.6 Hz, 2H), 7.52 (d, *J* = 8.7 Hz, 2H), 2.71 (s, 3H).

^{13}C NMR (126 MHz, $CDCl_3$) δ 145.04, 132.74, 125.62, 125.30, 44.14.

HR-MS(ESI$^+$) calc. for C_7H_7BrOS $[M+H]^+$ 218.9479, found 218.9448.

10. **2- Bromophenylsulfoxide (10b)**

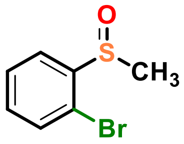

Was prepared from commercially available 2-Bromothioanisole (0.2 mmol) according to general procedure (XIX): Purification by column chromatography (SiO_2, EtOAc:Hexane = 1:1) afforded the 2- Bromophenylsulfoxide(29.1mg, 0.13mmol, 66%) as a reddish brown solid.

^{1}H NMR (500 MHz, $CDCl_3$) δ 7.94 (dd, *J* = 7.8, 1.7 Hz, 1H), 7.60 – 7.54 (m, 2H), 7.37 (td, *J* = 7.7, 1.7 Hz, 1H), 2.81 (s, 3H).
^{13}C NMR (126 MHz, $CDCl_3$) δ 145.41, 133.03, 132.40, 128.86, 125.79, 118.52, 41.98, 29.80.

HR-MS(ESI$^+$) calc. for C_7H_7BrOS $[M+H]^+$ 218.9479, found 218.9496.

11) 4-Nitrilephenyl sulfoxide (11b)

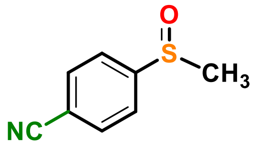

Was prepared from commercially available 4-Nitrilethioanisole (0.2 mmol) according to general procedure (XIX) at 50°C: Purification by column chromatography (SiO_2, EtOAc:Hexane = 4:1) afforded the 4-Nitrilephenylsulfoxide(19.2mg, 0.11mmol, 58%) as a white solid.

^{1}H NMR (500 MHz, $CDCl_3$) *δ* 7.83 (d, *J* = 8.6 Hz, 1H), 7.76 (d, *J* = 8.6 Hz, 1H), 2.76 (s, 2H).
^{13}C NMR (126 MHz, $CDCl_3$) *δ* 151.30, 132.88, 124.17, 117.59, 114.67, 43.68.

HR-MS(ESI$^+$) calc. for C_8H_7NOS $[M+H]^+$ 166.0327, found 166.0322.

12) 4-Nitrophenyl methyl sulfoxide (12b)

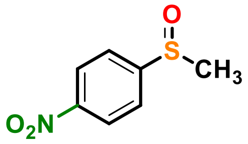

Was prepared from commercially available 4-Nitrothioanisole (0.2 mmol) according to general procedure (XIX) at 50°C: Purification by column chromatography (SiO_2, ethyl acetate: hexane = 4:1) afforded the title compound 4-Nitrophenyl methyl sulfoxide (16.5 mg, 0.09 mmol, 45%).

^{1}H NMR (500 MHz, $CDCl_3$) *δ* 8.39 (d, *J* = 9.0 Hz, 2H), 7.84 (d, *J* = 8.9 Hz, 2H), 2.79 (s, 3H).
^{13}C NMR (126 MHz, $CDCl_3$) *δ* 153.30, 149.62, 124.76, 124.60, 43.95.

HR-MS(ESI$^+$) calc. for $C_7H_7NO_3S$ $[M+H]^+$ 186.0225, found 186.0218.

13) Methyl(4-(trifluoromethyl)phenyl) sulfoxide (13b)

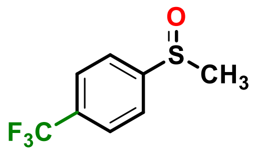

Was prepared from Methyl(4-(trifluoromethyl)phenyl) sulfane(0.2 mmol) according to general procedure (XIX): Purification by column chromatography (SiO_2,EtOAc : Hexane = 4:1) afforded the Methyl(4-(trifluoromethyl)phenyl) sulfoxide(26.7mg, 0.13mmol, 64%) as pale yellow solid.

^{1}H NMR (500 MHz, $CDCl_3$) δ 7.87 – 7.71 (m, 4H), 2.76 (s, 3H).
^{13}C NMR (126 MHz, $CDCl_3$) δ 150.27, 133.13, 126.56, 126.53, 124.17,4.
^{19}F NMR (471 MHz, $CDCl_3$) δ -62.75.

HR-MS(ESI$^+$) calc. for $C_8H_7F_3OS$ [M+H]$^+$ 209.0248, found 209.0244.

14. (methylsulfinyl)-4-(trifluoromethoxy)benzene (14b)

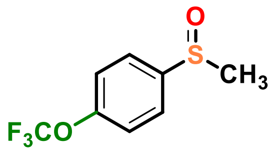

Was prepared from methyl(4-(trifluoromethoxy)phenyl)sulfane (0.2 mmol) according to general procedure (XIX): Purification by column chromatography (SiO_2, ethyl acetate: hexane = 4:1) afforded the title compound (methylsulfinyl)-4-(trifluoromethoxy)benzene (31.2 mg, 0.13 mmol, 69%).

^{1}H NMR (500 MHz, $CDCl_3$) δ 7.69 (d, J = 8.7 Hz, 2H), 7.37 (d, J = 7.8 Hz, 2H), 2.73 (s, 3H).
^{13}C NMR (126 MHz, $CDCl_3$) δ 151.20, 144.19, 125.54, 121.87,119.39, 44.15.

^{19}F NMR (471 MHz, $CDCl_3$) δ -57.65.

HR-MS(ESI$^+$) calc. for $C_8H_7F_3O_2S$ [M+H]$^+$ 225.0197, found 225.0284.

15. N-[4-(methylthiosulfoxide)phenyl]pivalamide (15b)

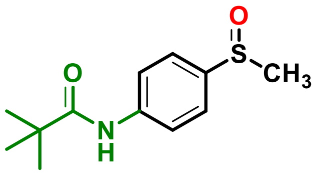

Was prepared from N-[4-(methylthio)phenyl]pivalamide (0.2 mmol) according to general procedure (XIX): Purification by column chromatography (SiO_2, ethyl acetate: hexane = 4:1)afforded the title compound N-(4-(methylthiosulfoxide)phenyl)pivalamide (28.56 mg, 0.12 mmol, 60%).

^{1}H NMR (500 MHz, $CDCl_3$) *δ* 7.72 (d, *J* = 8.7 Hz, 1H), 7.60 (d, *J* = 8.7 Hz, 1H), 2.70 (s, 2H), 1.33 (s, 1H).
^{13}C NMR (126 MHz, $CDCl_3$) *δ* 177.34, 141.24, 139.64, 124.63, 120.80, 43.90, 39.87, 27.58.
HR-MS(ESI$^+$) calc. for $C_{12}H_{17}NO_2S$ $[M+H]^+$ 240.1058, found 240.1056.

16. 4-(methylsulfinyl)benzoate (16b)

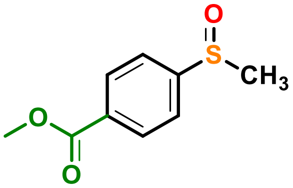

Was prepared from methyl 4-(methylthio)benzoate (0.2 mmol) according to general procedure (XIX) at 50℃: Purification by column chromatography (SiO_2, EtOAc: Hexane = 4:1) afforded the methyl 4-(methylsulfinyl)benzoate (25.6 mg, 0.13 mmol, 64%) as brown solid.

^{1}H NMR (500 MHz, $CDCl_3$) *δ* 8.16 (d, *J* = 8.7 Hz, 2H), 7.69 (d, *J* = 8.0 Hz, 2H), 3.92 (s, 3H), 2.73 (s, 3H).
^{13}C NMR (126 MHz, $CDCl_3$) *δ* 166.09, 150.88, 132.66, 130.55, 123.58, 52.58, 43.89.
HR-MS(ESI$^+$) calc. for $C_9H_{11}NO_2S$ $[M+H]^+$ 199.0429, found 199.0443.

17. 4-(methylsulfinyl)benzaldehyde (17b)

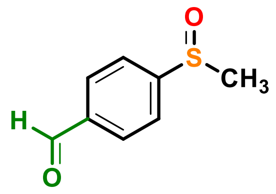

Was prepared from commercially available 4-methylthiobenzaldehyde (0.2 mmol) according to general procedure (XIX) at 50 ℃: Purification by column chromatography (SiO_2, EtOAc: Hexane = 4:1) afforded the 4-(methylsulfinyl)benzaldehyde (mg, mmol, %) as brown solid.

^{1}H NMR (500 MHz, $CDCl_3$) δ 7.81 (d, J = 8.7 Hz, 2H), 7.75 (d, J = 7.6 Hz, 2H), 2.75 (s, 3H).
^{13}C NMR (126 MHz, $CDCl_3$) δ 151.46, 133.05, 124.37, 117.77, 114.83, 43.82.
HR-MS(ESI$^+$) calc. for $C_8H_8O_2S$ $[M+H]^+$ 169.0323, found 169.0861.

18. 4,4,5,5-tetramethyl-2-(4-(methylsulfinyl)phenyl)-1,3,2-dioxaborolane (18b)

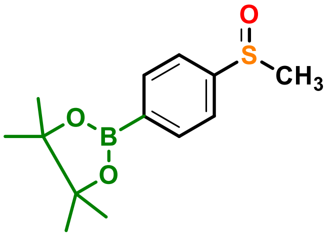

Was prepared from 4,4,5,5-tetramethyl-2-(4-(methylthio)phenyl)-1,3,2-dioxaborolane (0.2 mmol) according to general procedure (XIX) at 50 ℃: afforded the 4,4,5,5-tetramethyl-2-(4-(methylsulfinyl)phenyl)-1,3,2-dioxaborolane (6.9 mg, 0.025 mmol, 13%) as crude product.

^{1}H NMR (500 MHz, $CDCl_3$) δ 7.96 (d, J = 8.4 Hz, 2H), 7.63 (d, J = 8.3 Hz, 2H), 2.71 (s, 3H), 1.35 (s, 12H).
^{13}C NMR (126 MHz, $CDCl_3$) δ 148.78, 142.67, 135.65, 122.64, 84.33, 43.95, 29.79.

HR-MS(ESI$^+$) calc. for $C_{13}H_{19}BO_3S$ $[M+H]^+$ 267.1226, found 267.1227.

19. Benzyl methyl sulfoxide (19b)

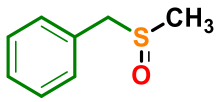

Was prepared from commercially available Benzyl methyl sulfane (0.2 mmol) according to general procedure (XIX) at 50℃. Purification by column chromatography (SiO_2, EtOAc:Hexane = 4:1) afforded the Benzylmethylsulfoxide (18.1 mg, 0.117 mmol, 58.6%) as a reddish brown solid.

^{1}H NMR (500 MHz, $CDCl_3$) δ 7.40 – 7.33 (m, 2H), 7.32 – 7.25 (m, 2H), 4.08 (d, 1H), 3.93 (d,1H), 2.45 (s,3H).

^{13}C NMR (126 MHz, $CDCl_3$) δ 129.90, 129.51, 128.88, 128.34, 60.17, 37.12.

HR-MS(ESI$^+$) calc. for $C_8H_{10}OS$ $[M+H]^+$ 155.0531, found 155.0526.

20. Diphenylsulfoxide (20b)

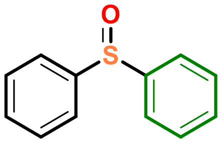

Was prepared from commercially available Diphenyl sulfane (0.2 mmol) according to general procedure (XIX): Purification by column chromatography (SiO_2, EtOAc:Hexane = 4:1) afforded the Diphenylsulfoxide(6 mg, 0.029 mmol, 15%) as a reddish brown solid.

^{1}H NMR (500 MHz, $CDCl_3$) δ 7.80 – 7.58 (m, 2H), 7.55 – 7.41 (m, 3H).

^{13}C NMR (126 MHz, $CDCl_3$) δ 145.71, 131.21, 129.48, 124.93.

HR-MS(ESI$^+$) calc. for $C_{12}H_{10}NOS$ $[M+H]^+$ 203.0531, found 203.0596.

21. (Cyclopropylsulfinyl)benzene (21b)

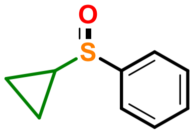

Was prepared from commercially available cyclopropyl(phenyl)sulfane (0.2 mmol) according to general procedure (XIX'): Purification by column chromatography (SiO_2, EtOAc:Hexane = 4:1) afforded the (cyclopropylsulfinyl)benzene (5.33 mg, 0.03 mmol, 16%) as yellow liquid.

^{1}H NMR (500 MHz, $CDCl_3$) *δ* 7.90 – 7.62 (m, 2H), 7.62 – 7.45 (m, 3H), 2.27 (tt, J = 7.8, 4.8 Hz, 1H), 1.07 – 1.01 (m, 1H), 1.00 – 0.91 (m, 2H), 0.88 (t, J = 7.0 Hz, 1H).

^{13}C NMR (126 MHz, $CDCl_3$) *δ* 144.98, 131.09, 129.32, 124.17, 33.98, 3.57.

HR-MS(ESI$^+$) calc. for $C_9H_{10}OS$ [M+H]$^+$ 167.0531, found 167.1062.

22. Ethylphenyl Sulfoxide (22b)

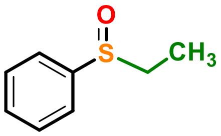

Was prepared from commercially available ethylphenyl sulfane (0.2 mmol) according to general procedure (XIX): Purification by column chromatography (SiO_2, EtOAc:Hexane = 4:1) afforded the ethylphenylsulfoxide (22.8 mg, 0.16 mmol, 80%) as pale yellow solid.

^{1}H NMR (500 MHz, $CDCl_3$) *δ* 7.64 – 7.58 (m, 2H), 7.56 – 7.48 (m, 3H), 3.00 – 2.84 (m, 1H), 2.84 – 2.72 (m, 1H), 1.20 (t, *J* = 7.5 Hz, 3H).

^{13}C NMR (126 MHz, $CDCl_3$) *δ* 143.39, 131.08, 129.29, 124.32, 50.43, 6.12.

HR-MS(ESI$^+$) calc. for $C_8H_{10}OS$ [M+H]$^+$ 155.0531, found 155.0534.

23. Dibenzothiophene sulfoxide (23b)

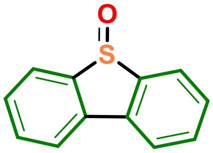

Was prepared from commercially available Dibenzothiophene (0.2 mmol) according to general procedure (XIX) by adding solvent 0.5mL DCM and 1.5 mL MeOH at 50 ℃: Purification by column chromatography (SiO_2, ethyl acetate:hexane = 4:1) afforded the title compound dibenzothiophene oxide (21.89 mg, .11 mmol, 55%) as a white solid.

^{1}H NMR (500 MHz, $CDCl_3$) *δ* 7.99 (d, *J* = 7.6 Hz, 2H), 7.80 (d, *J* = 7.6 Hz, 2H), 7.59 (td, *J* = 7.5, 1.1 Hz, 2H), 7.50 (td, *J* = 7.6, 1.2 Hz, 2H).

^{13}C NMR (126 MHz, $CDCl_3$) *δ* 145.25, 137.22, 132.68, 129.68, 127.66, 122.04.

HR-MS(ESI$^+$) calc. for $C_{12}H_8OS$ $[M+H]^+$ 201.0374, found 201.0442.

24.Thianaphthalene sulfoxide (24b)

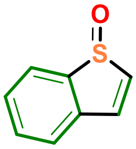

Was prepared from commercially available Thianaphthalene sulfide (0.2 mmol) according to general procedure (XIX) at 50 ℃: Purification by column chromatography (SiO_2, EtOAc:Hexane = 4:1) afforded the Thianaphthalene sulfoxide (13.5 mg, 0.08 mmol, 45%) as brown solid.

^{1}H NMR (500 MHz, $CDCl_3$) *δ* 7.93 (d, *J* = 8.4 Hz, 1H), 7.54 – 7.39 (m, 3H), 7.23 (d, *J* = 6.0 Hz, 1H), 7.09 (d, *J* = 6.3 Hz, 1H).

^{13}C NMR (126 MHz, $CDCl_3$) *δ* 139.78, 139.66, 126.41, 124.29, 124.24, 123.94, 123.71, 122.58.

HR-MS(ESI$^+$) calc. for C_8H_6OS $[M+H]^+$ 151.0218, found 150.9427

25. (4-(benzyloxy)phenyl)(methyl)sulfoxide (25b)

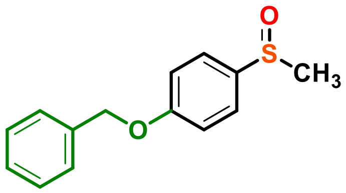

Was prepared from (4-(benzyloxy)phenyl)(methyl)sulfane(0.2 mmol) according to general procedure (XIX) at 50°C by adding solvent 0.5mL DCM and 1.5 mL H_2O. Purification by column chromatography (SiO_2, EtOAc:Hexane = 4:1) afforded the (4-(benzyloxy)phenyl)(methyl)sulfoxide (33.7mg, 0.13mmol, 68%) as brown solid.

^{1}H NMR (500 MHz, $CDCl_3$) *δ* 7.59 (d, *J* = 8.9 Hz, 2H), 7.45 – 7.32 (m, 5H), 7.10 (d, *J* = 8.9 Hz, 2H), 5.11 (s, 2H), 2.70 (s, 3H).
^{13}C NMR (126 MHz, $CDCl_3$) *δ* 161.20, 136.90, 136.26, 128.81, 128.37, 127.59, 125.61, 115.83, 70.34, 44.06.

HR-MS(ESI$^+$) calc. for $C_{14}H_{14}O_2S$ $[M+H]^+$ 247.0793, found 247.0798.

26. Dibutylsulfoxide (26b)

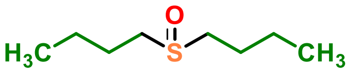

Was prepared from commercially available dibutylsulfane (0.2 mmol) according to general procedure (XIX'): Purification by column chromatography (SiO_2, EtOAc:Hexane = 4:1) afforded the dibutylsulfoxide (19.7mg, 0.122mmol, 61%) as a reddish brown solid.

^{1}H NMR (500 MHz, $CDCl_3$) *δ* 2.75 – 2.56 (m, 4H), 1.81 – 1.66 (m, 4H), 1.57 – 1.37 (m, 4H), 0.95 (t, *J* = 7.3 Hz, 6H).
^{13}C NMR (126 MHz, $CDCl_3$) *δ* 52.32, 24.77, 22.25, 13.83.
HR-MS(ESI$^+$) calc. for C_8H_8OS $[M+H]^+$ 163.1157, found 163.1157.

27. Hexylmethyl Sulfoxide (27b)

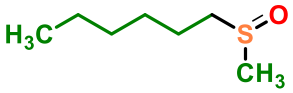

Was prepared from hexylmethyl sulfane (0.2 mmol) according to general procedure (XIX'): Purification by column chromatography (SiO_2, EtOAc:Hexane = 4:1) afforded hexylmethyl sulfoxide (16.7mg, 0.105mmol, 58.5%) as a reddish brown solid.

^{1}H NMR (500 MHz, $CDCl_3$) δ 2.82 – 2.71 (m, 1H), 2.69 – 2.60 (m, 1H), 2.57 (s, 3H), 1.88 – 1.67 (m, 2H), 1.51 – 1.38 (m, 2H), 1.36 – 1.29 (m, 4H), 0.97 – 0.81 (m, 3H).
^{13}C NMR (126 MHz, $CDCl_3$) δ 54.78, 38.53, 31.45, 28.55, 22.55, 14.07.

HR-MS(ESI$^+$) calc. for C_7H1_6OS $[M+H]^+$ 149.1000, found 149.0996.

28. Tertbutyl methyl sulfoxide (28b)

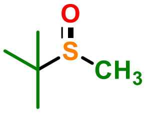

Was prepared from commercially available tertbutyl methyl sulfide (0.2 mmol) according to general procedure (XIX') at 50 ℃: Purification by column chromatography (SiO_2, EtOAc:Hexane = 4:1) afforded the Tertbutyl methyl sulfoxide (8.5 mg, 0.07 mmol, 35 %) as brown solid.

^{1}H NMR (500 MHz, $CDCl_3$) δ 2.38 (s, 3H), 1.24 (s, 9H).
^{13}C NMR (126 MHz, $CDCl_3$) δ 52.72, 31.66, 22.62.
HR-MS(ESI$^+$) calc. for $C_5H_{12}OS$ $[M+H]^+$ 121.0687, found 121.0681.

29. 6-(methylsulfinyl)hexanenitrile (29b)

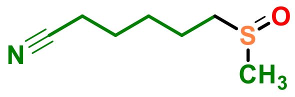

Was prepared from 6-(methylthio)hexanenitrile (0.2 mmol) according to general procedure (XIX') at 50℃: Purification by column chromatography (SiO_2, EtOAc:Hexane = 4:1) afforded the 6-(methylsulfinyl)hexanenitrile (15.3 mg, 0.09 mmol, 48%) as yellow liquid.

^{1}H NMR (500 MHz, $CDCl_3$) *δ* 2.79 – 2.69 (m, 1H), 2.65 (dd, J = 12.8, 7.9 Hz, 1H), 2.57 (s, 3H), 2.37 (t, J = 7.0 Hz, 2H), 1.84 (dd, J = 16.3, 6.6 Hz, 2H), 1.76 – 1.68 (m, 2H), 1.67 – 1.56 (m, 2H).
^{13}C NMR (126 MHz, $CDCl_3$) *δ* 119.45, 54.12, 38.72, 27.86, 25.12, 22.05, 17.10.
HR-MS(ESI$^+$) calc. for $C_7H_{13}NOS$ [M+H]$^+$ 160.0796, found 160.0792.

30. Cyclopentyl methyl sulfoxide (30b)

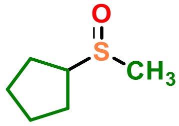

Was prepared from cyclopentyl methyl sulfide (0.2 mmol) according to general procedure (XIX'): Purification by column chromatography (SiO_2, EtOAc:Hexane = 4:1) afforded the cyclopentyl methyl sulfoxide (10.2 mg, 0.077 mmol, 39%) as yellow liquid.

^{1}H NMR (500 MHz, $CDCl_3$) *δ* 3.00 (ddd, J = 15.0, 8.7, 6.6 Hz, 1H), 2.50 (s, 3H), 2.21 – 2.09 (m, 2H), 2.01 – 1.89 (m, 2H), 1.79 – 1.61 (m, 4H).
^{13}C NMR (126 MHz, $CDCl_3$) *δ* 62.11, 37.20, 27.49, 25.85.
HR-MS(ESI+) calc. for $C_6H_{12}OS$ [M+H]+ 133.0687, found 133.0681.

31. Cyclohexylmethyl sulfoxide (31b)

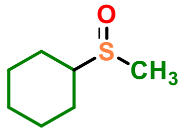

Was prepared from cyclohexyl(methyl)sulfane (0.2 mmol) according to general procedure (XIX'): Purification by column chromatography (SiO_2, EtOAc:Hexane = 4:1) afforded cyclohexylmethyl sulfoxide (17.2mg, 0.105mmol, 52.8%) as a reddish brown solid.

^{1}H NMR (500 MHz, $CDCl_3$) *δ* 2.51 (s, 3H), 2.14 (d, *J* = 14.5 Hz, 1H), 1.95 – 1.83 (m, 3H), 1.72 (d, *J* = 11.0 Hz, 1H), 1.50 – 1.18 (m, 5H), 0.90 – 0.80 (m, 1H).
^{13}C NMR (126 MHz, $CDCl_3$) *δ δ* 60.90, 35.16, 26.09, 25.59, 25.45, 25.21, 24.92.

HR-MS(ESI$^+$) calc. for $C_7H_{14}OS$ [M+H]$^+$ 147.0844, found 147.0843.

32. (Methylsulfinyl)adamantane (32b)

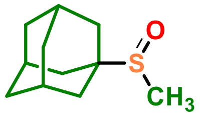

Was prepared from (0.2 mmol) according to general procedure (XIX') at 50℃: Purification by column chromatography (SiO_2, EtOAc:Hexane = 4:1) afforded the (methylsulfinyl)adamantane (13.1 mg, 0.06 mmol, 33%) as yellow liquid.

^{1}H NMR (500 MHz, $CDCl_3$) *δ* 2.38 (s, 3H), 2.19 (p, *J* = 3.2 Hz, 3H), 2.03 (d, *J* = 3.1 Hz, 1H), 1.93 – 1.80 (m, 3H), 1.78 (d, *J* = 10.5 Hz, 6H), 1.71 (d, *J* = 14.5 Hz, 2H).

^{13}C NMR (126 MHz, $CDCl_3$) *δ* 36.41, 35.83, 34.79, 29.79, 28.65.

HR-MS(ESI$^+$) calc. for $C_{11}H_{18}OS$ [M+H]$^+$ 199.1157, found 199.1156.

33.(2-Methylthiosulfoxide) pyridine (33b)

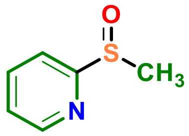

Was prepared from commercially available 2-Methylthiopyridine (0.2 mmol) according to general procedure (XIX): Purification by column chromatography (SiO_2, EtOAc:Hexane = 4:1) afforded the (2-Methylthiosulfoxide) pyridine (22.8mg, 0.16 mmol, 80%) as a yellowish solid.

^{1}H NMR (500 MHz, $CDCl_3$) *δ* 8.62 (ddd, *J* = 4.7, 1.8, 0.9 Hz, 1H), 8.03 (dt, *J* = 7.8, 1.1 Hz, 1H), 7.95 (td, *J* = 7.7, 1.8 Hz, 1H), 7.38 (ddd, *J* = 7.6, 4.7, 1.2 Hz, 1H), 2.85 (s, 3H).
^{13}C NMR (126 MHz, $CDCl_3$) *δ* 166.05, 149.70, 138.29, 124.76, 119.43, 41.41.

HR-MS(ESI$^+$) calc. for C_6H_7NOS [M+H]$^+$ 142.0237, found 142.0318.

34. 2-(methylthiosulfoxide)pyrazine (34b)

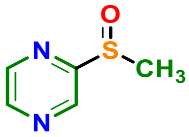

Was prepared from commercially available 2-Methylthiopyrazine (0.2 mmol) according to general procedure (XIX): Purification by column chromatography (SiO_2, EtOAc:Hexane = 4:1) afforded the (2-Methylthiosulfoxide) pyrazine(16.7mg, 0.12mmol, 59%) as yellowish solid.

^{1}H NMR (500 MHz, $CDCl_3$) *δ* 9.23 (d, *J* = 1.5 Hz, 1H), 8.73 (d, *J* = 2.3 Hz, 1H), 8.60 (dd, *J* = 2.4, 1.5 Hz, 1H), 2.93 (s, 3H).
^{13}C NMR (126 MHz, $CDCl_3$) *δ* 161.67, 146.00, 143.89, 142.04, 41.16.

HR-MS(ESI$^+$) calc. for $C_5H_6N_2OS$ [M+H]$^+$ 143.0279, found 143.0013.

35. 4-(4-(methylthiosulfoxide)phenyl)pyridine (35b)

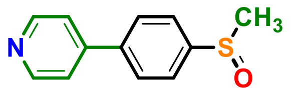

Was prepared from 4-(4-(methylthio)phenyl)pyridine (0.2 mmol) according to general procedure (XIX) at 50°C by adding solvent 0.5mL DCM and 1.5 mL H_2O. Purification by column chromatography (SiO_2, EtOAc:Hexane = 4:1) afforded the 4-(4-(methylthiosulfoxide)phenyl)pyridine (27.3mg, 0.12mmol, 62%) as brown solid.

^{1}H NMR (500 MHz, $CDCl_3$) *δ* 8.70 (d, *J* = 5.7 Hz, 2H), 7.81 – 7.73 (m, 4H), 7.52 (d, *J* = 6.6 Hz, 2H), 2.77 (s, 3H).
^{13}C NMR (126 MHz, $CDCl_3$ *δ* 150.51, 147.22, 146.76, 141.10, 128.16, 124.49, 121.87, 44.09.
HR-MS(ESI$^+$) calc. for $C_{12}H_{11}NOS$ $[M+H]^+$ 218.0670, found 218.0645.

36. 6-(methylsulfinyl)-2,2'-bipyridine (36b)

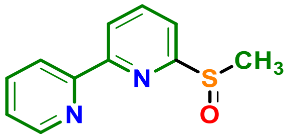

Was prepared from 6-(methylthio)-2,2'-bipyridine (0.2 mmol) according to general procedure (XIX) at 50℃: Purification by column chromatography (SiO_2, EtOAc:Hexane = 4:1) afforded the 6-(methylsulfinyl)-2,2'-bipyridine (15.4 mg, 0.07 mmol, 35%) as brown solid.

^{1}H NMR (500 MHz, $CDCl_3$) *δ* 8.70 (ddd, J = 4.7, 1.8, 0.9 Hz, 1H), 8.51 (dd, J = 7.6, 1.3 Hz, 1H), 8.37 (dt, J = 7.9, 1.1 Hz, 1H), 8.12 – 8.01 (m, 2H), 7.84 (td, J = 7.7, 1.8 Hz, 1H), 7.36 (ddd, J = 7.5, 4.7, 1.2 Hz, 1H), 2.91 (s, 3H).
^{13}C NMR (126 MHz, $CDCl_3$) *δ* 149.53, 139.17, 137.18, 124.59, 122.05, 121.39, 119.27, 41.52.
HR-MS(ESI$^+$) calc. for $C_{11}H_{10}N_2OS$ $[M+H]^+$ 219.0592, found 219.0589.

37. Thieno[2,3-b]pyridine 1-oxide (37b)

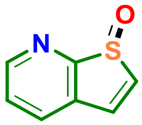

Was prepared from commercially available Thieno[2,3-b]pyridine (0.2 mmol) according to general procedure (XIX) at 50 ℃: afforded the Thieno[2,3-b]pyridine 1-oxide (14.9 mg, 0.1 mmol, 49%) as crude product.

^{1}H NMR (500 MHz, $CDCl_3$): *δ* 8.57 (dd, *J* = 4.7, 1.6 Hz, 1H), 8.07 (dd, *J* = 8.0, 1.6 Hz, 1H), 7.52 (d, *J* = 6.0 Hz, 1H), 7.37 – 7.20 (m, 2H).
^{13}C NMR (126 MHz, $CDCl_3$): *δ* 161.95, 146.69, 132.63, 131.18, 127.12, 121.61, 119.47.
HR-MS(ESI$^+$) calc. for C_7H_5NOS $[M+H]^+$ 152.0170 found 152.0326.

38. 4-(4-(4-fluorophenyl)-2-(4-(methylsulfinyl)phenyl)-1H-imidazol-5-yl)pyridine (38b)

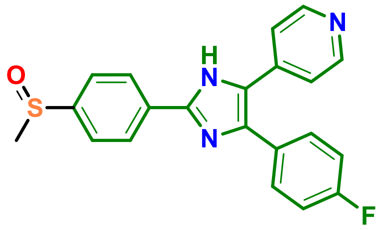

Was prepared from 4-(4-(4-fluorophenyl)-2-(4-(methylthio)phenyl)-1H-imidazol-5-yl)pyridine (0.2 mmol) according to general procedure (XIX) at 50 ℃: Purification by column chromatography (SiO_2, EtOAc:Hexane = 4:1) afforded the 4-(4-(4-fluorophenyl)-2-(4-(methylsulfinyl)phenyl)-1H-imidazol-5-yl)pyridine (31.9 mg, 0.08 mmol, 42%) as white solid.

^{1}H NMR (500 MHz, DMSO-*d*$_6$): *δ* 8.47 (d, *J* = 6.1 Hz, 2H), 8.26 (d, *J* = 6.7 Hz, 2H), 7.81 (d, *J* = 10.7 Hz, 2H), 7.64 – 7.59 (m, 2H), 7.50 (d, *J* = 4.6 Hz, 2H), 7.38 (t, *J* = 8.9 Hz, 2H), 2.80 (s, 3H).
^{13}C NMR (126 MHz, DMSO-*d*$_6$): δ 150.11, 149.79, 146.34, 145.14, 141.87, 134.58, 131.28, 129.90, 125.89, 124.32, 121.87, 120.78, 116.13, 115.96, 43.08.
^{19}F NMR (471 MHz, DMSO_*d*$_6$): δ -112.39.
HR-MS(ESI$^+$) calc. for $C_{21}H_{16}FN_3OS$ $[M+H]^+$ 378.1076 found 378.1168

Spectral data for the substrates synthesized (XXXI):

1.Synthesis of 2,6-Dimethylbenzenethioanisole (5a)

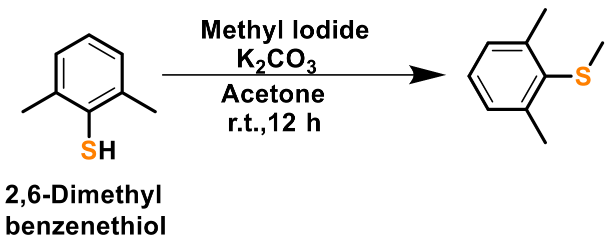

Potassium carbonate (10 mmol, 2.0 equiv.) was added to a solution of,6-Dimethylbenzenethiol (5 mmol,1.0 equiv.) in acetone (30 mL) followed by dropwise addition of iodomethane (2.1g, 15.0mmol, 3.0 equiv.). The reaction mixture was heated for 12 h. Then reaction mixture was allowed to cool to room temperature followed by filteration and removal of solvent. The crude reaction mixture was dissolved in ethyl acetate, washed with brine and dried over Na_2SO_4. Finally the combined organic phases were concentrated and purified by column chromatography (4:1, EtOAc:Hexane) to obtain 2,6-Dimethylbenzenethioanisole as a yellow liquid (0.624g, 4.1 mmol, 82 % yield) whose NMR matches with the literature report.[7]

^{1}H NMR (500 MHz, $CDCl_3$) *δ* 7.15 – 7.08 (m, 3H), 2.57 (s, 6H), 2.24 (s, 3H).
^{13}C NMR (125 MHz, $CDCl_3$) *δ* 142.84, 135.24, 128.22, 128.17, 21.88, 18.36.
HR-MS(ESI$^+$) calc. for $C_9H_{12}S$ $[M+H]^+$ 153.0738, found 153.0907.

2. Synthesis of 4-MethylBenzeneThioanisole (2a)

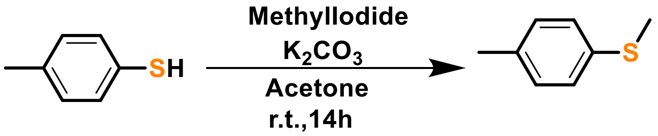

Potassium carbonate (6.91g, 50 mmol) was added to a solution of 4-methylbenzenethiol (5g, 40mmol) in acetone (50 mL) followed by dropwise addition of iodomethane (2.80 mL ,45 mmol). The reaction mixture was stirred at room temperature for 14 h. After 14 h the reaction mixture was filtered through celite and the filterate was concentrated under reduced pressure. The crude reaction mixture was dissolved in diethyl ether and washed with 5% NaOH (7.5g), then extracted the combined aqueous layer with diethyl ether and washed the organic layer with brine, dried over

Na_2SO_4. Finally the combined organic phases were concentrated and purified by column chromatography (4:1, EtOAc:Hexane) to obtain 4-methylbenzenesulfide as a yellow liquid (5.418 g, 39 mmol, 92%) whose NMR matches with the literature report. [8]

^{1}H NMR (500 MHz, $CDCl_3$) *δ* 7.19 (d, J = 8.4 Hz, 2H), 7.11 (d, J = 7.9 Hz, 2H), 2.47 (s, 3H), 2.32 (s, 3H).
^{13}C NMR (125 MHz, $CDCl_3$) *δ* 135.16, 134.78, 129.73, 127.35, 21.05, 16.63.
HR-MS(ESI$^+$) calc. for $C_8H_{10}S$ $[M+H]^+$ 139.0581, found 139.0576.

3. Synthesis of 4-*tert*butylbenzenethioanisole (4a)

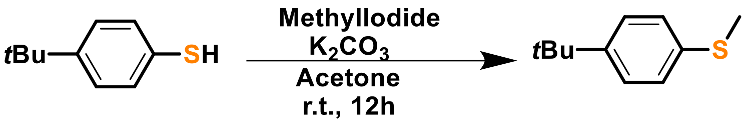

Potassium carbonate (3.327gm, 24.08mmol), was added to a solution of 4-*tert*-butylbenzene thiol (12.04 mmol,1 equiv.) in acetone (30 mL) followed by dropwise addition of iodomethane (2.307 mL, 36.12 mmol), The reaction mixture was heated for 12 h. Then reaction mixture was allowed to cool to room temperature followed by filteration and removal of solvent. The crude reaction mixture was dissolved in ethyl acetate, washed with brine and dried over Na_2SO_4. Finally the combined organic phases were concentrated and purified by column chromatography (4:1, EtOAc:Hexane) to obtain 4-*tert*butylbenzenethioanisole as a yellow liquid (1.78 g, 9.87 mmol,82% yield) whose NMR matches with the literature report.[7]

^{1}H NMR (500 MHz, $CDCl_3$) *δ* 7.40 – 7.34 (m, 2H), 7.30 – 7.24 (m, 2H), 2.51 (s, 3H), 1.36 (s, 9H).
^{13}C NMR (125 MHz, $CDCl_3$) *δ* 142.86, 135.31, 128.23, 128.20, 21.89, 18.39.
HR-MS(ESI$^+$) calc. for $C_{11}H_{16}S$ $[M+H]^+$ 181.1051, found 181.1042.

4.Synthesis of Cyclohexylmethylsulfide (31a)

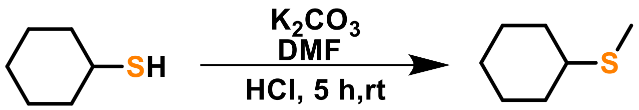

A solution of Iodomethane (3.22 mL,0.5 mol) in DMF (5 mL) was added dropwise to a solution of cyclohexane thiol (5.25 mL,0.42 mol) in DMF (30mL) while stirring at room temperature, followed by addition of potassium carbonate (8.5 g,0.5 mol). The reaction mixture was stirred for 5 h at room temperature. Then, the reaction mixture was quenched with aqueous hydrochloric acid followed by addition of DCM. The combined organic layer was washed with aqueous hydrochloric acid, brine, dried over Na_2SO_4. Finally the combined organic phases were concentrated and purified by column chromatography (1:1, EtOAc:Hexane) to obtain Cyclohexyl methyl sulfide as a light yellow oil (1.934g, 14.84 mmol,) whose NMR matches with the literature report. [9]

^{1}H NMR (500 MHz, $CDCl_3$) δ 2.60 – 2.47 (m, 1H), 2.08 (s, 3H), 2.03 – 1.91 (m, 2H), 1.83 – 1.70 (m, 2H), 1.67 – 1.59 (m, 1H), 1.37 – 1.16 (m, 5H).
^{13}C NMR (125 MHz, $CDCl_3$) δ 44.99, 33.23, 26.27, 25.99, 13.41.
HR-MS(ESI$^+$) calc. for $C_7H_{14}S$ $[M+H]^+$ 131.0894, found 131.0280.

5. Synthesis of Hexylmethylsulfide (27a)

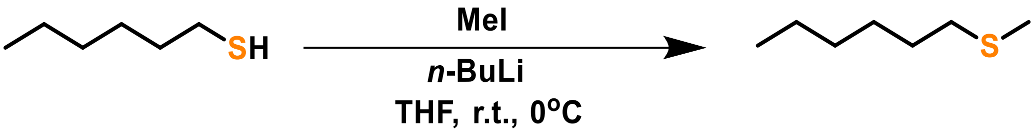

n-butyllithium (1.6M in hexane,6.25mL,10mmol) was added to a solution of the hexylmethyl thiol (1.13mL, 8mmol) in anhydrous tetrahydrofuran at 0˚C. Then, the reaction mixture was allowed to warm to room temperature, and stirred for 10 minutes followed by addition of methyl iodide (0.498mL ,8mmol) slowly at 0˚C. The reaction mixture was stirred for 20 minutes at ambient temperature. Then the reaction mixture was quenched with H_2O and extracted with hexane. The combined organic layer was washed with H_2O and dried over Na_2SO_4. Finally the combined organic phases were concentrated and purified by column chromatography (1:1, EtOAc:Hexane)

to obtain Hexyl methyl sulfide as a light yellow liquid (0.793g, 6 mmol, 75%) whose NMR matches with the literature report.[10]

^{1}H NMR (500 MHz, $CDCl_3$) δ 2.56 – 2.45 (m, 2H), 2.09 (s, 3H), 1.64 – 1.52 (m, 2H), 1.44 – 1.34 (m, 2H), 1.33 – 1.24 (m, 4H), 0.88 (t, J = 7.0 Hz, 3H).
^{13}C NMR (125 MHz, $CDCl_3$) δ 34.43, 31.60, 29.28, 28.66, 22.71, 15.67, 14.19.
HR-MS(ESI$^+$) calc. for $C_7H_{16}S$ [M+H]$^+$ 133.1051, found 133.0440.

6. Synthesis of 4-[4-(Methylthio)phenyl]pyridine (35a)

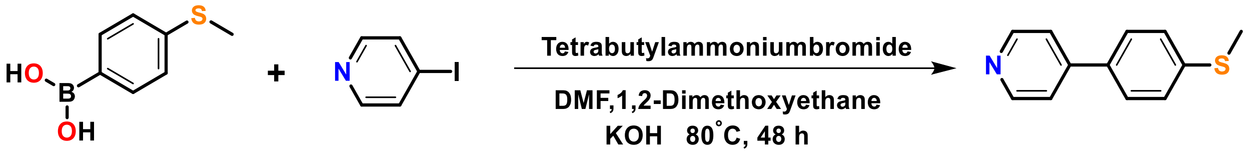

4-Methylthiophenylboronic acid (0.505g,3mmol) and 4-Iodopyridine (0.615g,3mmol) was dissolved in a mixture of Dimethylformamide:Dimethoxyethane (40:32) in a round bottom flask. To this was added Potassium hydroxide (0.86g,15.3mmol) and $(Bu)_4NBr$ (0.188g,0.39mmol). The round bottom flask was placed in a Schlenk line and added $Pd(PPh_3)_4$ (0.1271g,0.11mmol). the vessel was sealed, heated to 80°C and stirred for 48 h. The reaction mixture was then cooled and poured into CH_2Cl_2. The crude organic mixture was washed with water and brine. The organic layer was then dried over anhydrous Na_2SO_4, filtered, and the solvent was removed via rotary evaporation. Column chromatography (SiO2, EtOAc : Hexanes = 4:1) was performed to obtain 4-[4-(Methylthio)phenyl]pyridine was obtained as a white crystalline solid (0.483g,2.4 mmol, 80%) whose NMR matches with the literature report.[11]

^{1}H NMR (500 MHz, $CDCl_3$) δ 8.63 (d, J = 6.3 Hz, 2H), 7.55 (d, J = 9.5 Hz, 2H), 7.46 (d, J = 5.0 Hz, 2H), 7.33 (d, J = 7.8 Hz, 2H), 2.51 (s, 3H).
^{13}C NMR (125 MHz, $CDCl_3$) δ 150.40, 147.63, 140.41, 134.54, 127.31, 126.68, 121.23, 15.50.
HR-MS(ESI$^+$) calc. for $C_{12}H_{11}NS$ [M+H]$^+$ 202.0694, found 202.0775.

7. (4-(benzyloxy)phenyl)(methyl)sulfane (25a)

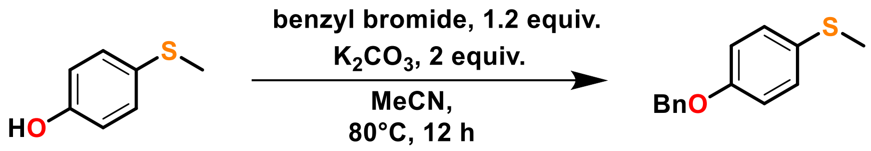

4-(methylthio)phenol

To a solution of 4-(methylthio) phenol (1.0 g, 7.1 mmol) and K_2CO_3 (2.0 g, 14.3 mmol) in dry MeCN (36 mL), was added benzyl bromide (1.0 mL, 8.6 mmol). The reaction mixture was stirred at 80 °C for 12 hours. After 12 h the reaction mixture was allowed to cooled to room temperature and concentrated to dryness. The crude reaction mixture was partitioned between CH_2Cl_2 and water. The layers were separated, and the aqueous phase was extracted three times with CH_2Cl_2. The combined organic layers were dried over anhydrous Na_2SO_4 and concentrated to dryness. The residue was purified by column chromatography (SiO_2, hexane:EtOAc 20:1) to afford product (4-(benzyloxy)phenyl)(methyl)sulfane (1.49 g,6.5 mmol 91%) as a white solid whose NMR matches with the literature report. [12]

^{1}H NMR (500 MHz, $CDCl_3$) δ 7.46 – 7.31 (m, 5H), 7.27 (d, J = 8.9 Hz, 2H), 6.93 (d, J = 8.9 Hz, 2H), 5.05 (s, 2H), 2.45 (s, 3H).

^{13}C NMR (125 MHz, $CDCl_3$) δ 157.44, 136.97, 130.13, 129.24, 128.75, 128.16, 127.61, 115.70, 70.26, 18.06.

HR-MS(ESI$^+$) calc. for $C_{14}H_{14}OS$ $[M+H]^+$231.0844, found 231.0837.

8. 4,4,5,5-tetramethyl-2-(4-(methylthio)phenyl)-1,3,2-dioxaborolane (18a)

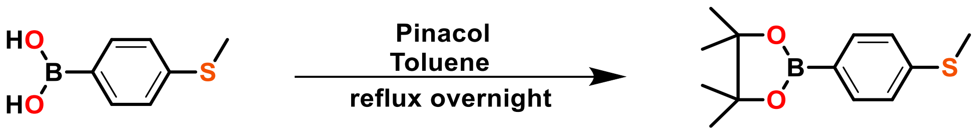

A solution of 4-(methylthio) phenylboronic acid (2 g, 11.84 mmol) and pinacol (1.40 g, 5.95 mmol) in toluene (50 mL) was refluxed overnight. Then, molecular sieves (3 Å, 50 mg) were added, and

the reaction mixture was stirred for an additional 2 h. After 2 h the reaction mixture was allowed to cooled to room temperature, filtered, and the solvent was evaporated to give a pale yellow solid. The residue was purified by column chromatography (SiO_2; *n*-hexane/EtOAc, 2:1) to give the product as a white solid (0.705g, 2.82 mmol,94%) whose NMR matches with the literature report.[13]

^{1}H NMR (500 MHz, $CDCl_3$) δ 7.71 (d, J = 8.4 Hz, 2H), 7.22 (d, J = 8.4 Hz, 2H), 2.49 (s, 3H), 1.34 (s, 12H).

^{13}C NMR (125 MHz, $CDCl_3$) δ 142.70, 135.20, 125.09, 83.86, 24.98, 15.16.

HR-MS(ESI$^+$) calc. for $C_{13}H_{13}BO_2S$ [M+H]$^+$ 251.1277, found 251.1273.

9. Synthesis of methyl 4-(methylthio)benzoate (16a)

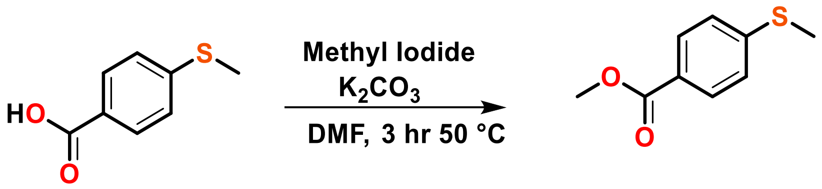

Methyl iodide (0.56 mL, 9 mmol, 3 equiv) was added to a solution of 4-(methylthio)benzoic acid (504.6 mg, 3 mmol, 1 equiv) and potassium carbonate (1.26 g, 9 mmol, 3.0 equiv) in N,N-dimethylformamide (20 mL) at room temperature. Then the reaction mixture was stirred for 3 h at 50°C. Then, reaction mixture was allowed to cooled to room temperature. The reaction mixture was quenched with H_2O and extracted Et_2O (5 mL × 3). The combined organic layer was washed with brine and dried over Na_2SO_4.Finally the combined organic phases were concentrated and purified by column chromatography (n-hexane/EtOAc = 4/1) to give product as a colorless solid whose NMR matches with the literature report.[14]

^{1}H NMR (500 MHz, $CDCl_3$) δ 7.93 (d, J = 8.7 Hz, 2H), 7.24 (d, J = 8.9 Hz, 2H), 3.89 (s, 3H), 2.51 (s, 3H).

^{13}C NMR (125 MHz, $CDCl_3$) δ 167.01, 145.57, 130.01, 126.42, 125.08, 52.14, 14.96.

HR-MS(ESI$^+$) calc. for $C_9H_{10}O_2S$ [M+H]$^+$ 183.0480, found 183.0839.

10. 6-(methylthio)hexanenitrile (29a)

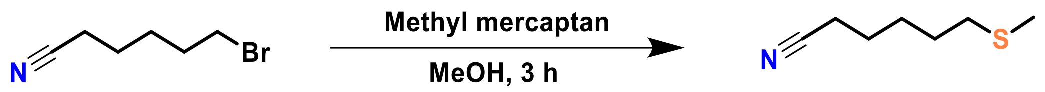

15% Methyl mercaptan sodium solution (3.25 g) was added to MeOH (2mL). To this, 6-bromohexanenitrile (1 g, 5.68 mmol) was slowly added dropwise at 0°C. After the dropwise addition, the reaction mixture was stirred for 2 minutes under ice-cooling and further stirred for 3 h at room temperature. Then, methanol was completely distilled off by rotary evaporator, water (20mL) was added to the obtained residue and extracted three times with diethyl ether (20ml). The organic layer was washed sequentially with 5% hydrochloric acid, water and brine and dried over Na_2SO_4.Finally the combined organic phases were concentrated to give 6-Methylthio hexanenitrile as colourless oil (0.6 g, 4.2 mmol,74%).

^{1}H NMR (500 MHz, $CDCl_3$) δ 2.50 (t, J = 7.1 Hz, 2H), 2.35 (t, J = 7.1 Hz, 2H), 2.09 (s, 3H), 1.74 – 1.50 (m, 6H).

^{13}C NMR (125 MHz, $CDCl_3$) δ 119.74, 33.92, 28.36, 27.87, 25.16, 17.23, 15.66.

HR-MS(ESI$^+$) calc. for $C_7H_{13}NS$ $[M+H]^+$ 144.0847, found 144.0842.

11. Sythesis of methyl(4-(trifluoromethyl)phenyl) sulfane (13a)

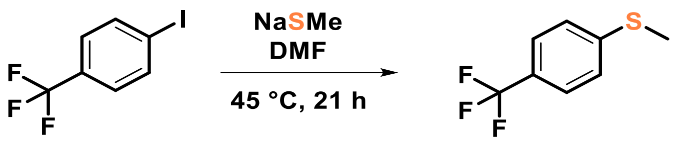

4-Iodobenzo-trifluoride (2.16 mL, 14.70 mmol) was added to a solution of sodium thiomethoxide (1.14 g, 16.16 mmol) in DMF (36 mL) and the resulting solution was heated at 45 ºC for 21 h.Then, the reaction mixture was quenched with sat. aq. NaCl (20 mL) and extracted with Et_2O (6 × 20 mL). The combined organic layer was washed with brine and dried over Na_2SO_4. Finally the combined organic phases were concentrated and purified by column chromatography (100%

hexane) to mobtain methyl(4-(trifluoromethyl)phenyl)sulfane as a white solid (1.3 g, 6.7 mmol,46%) whose NMR matches with the literature report.[15]

^{1}H NMR (500 MHz, $CDCl_3$) *δ* 7.52 (d, *J* = 8.3 Hz, 2H), 7.30 (d, *J* = 9.0 Hz, 2H), 2.51 (s, 3H).

^{13}C NMR (125 MHz, $CDCl_3$) *δ* 143.98, 127.01, 126.75, 125.66, 123.31, 15.09.

^{19}F NMR (471 MHz, $CDCl_3$) *δ* -62.19.

HR-MS(ESI$^+$) calc. for $C_8H_7F_3S$ $[M+H]^+$ 193.0299, found 193.0109.

12. Cyclopentylmethyl sulfide (30a)

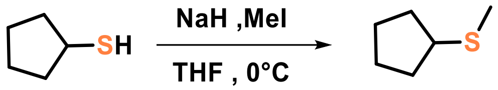

NaH (1.1 equiv., 0.25g, 10.35 mmol) was added to a solution of Cyclopentane thiol (1.0 equiv., 1mL, 9.4 mmol) in dry THF at 0℃. The reaction mixture was stirred for 30 min followed by dropwise addition of iodomethane (1.5 equiv., 0.87ml, 14.1 mmol). The reaction mixture was stirred for 2-6 h at rt. Then, the reaction was quenched by the addition of ice-cold water extracted with DCM, and the combined organic layers were washed with brine and dried over anhydrous Na_2SO_4. The solvent was removed under reduced pressure to give Cyclopentylmethyl sulfide as yellow oil (870 mg, 7.5 mmol, 79%) whose NMR matches with the literature report. [16]

^{1}H NMR (500 MHz, $CDCl_3$) *δ* 3.02 (p, *J* = 7.0 Hz, 1H), 2.09 (s, 3H), 2.05 – 1.91 (m, 2H), 1.81 – 1.68 (m, 2H), 1.60 – 1.48 (m, 4H).
^{13}C NMR (126 MHz, $CDCl_3$) *δ* 45.60, 33.36, 24.98, 15.09.
HR-MS(ESI$^+$) calc. for $C_6H_{12}S$ $[M+H]^+$ 117.0738, found 117.0911.

13. Adamantan-1-yl)(methyl)sulfane (32a)

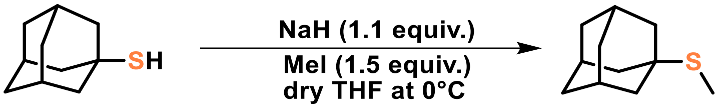

NaH (1.1 equiv., 87.64 mg) was added to a solution of substituted thiol (1 equiv., 336.6 mg, 2 mmol) in dry THF at 0°C. The reaction mixture was stirred for 30 min followed by dropwise addition of iodomethane (1.5 equiv.,0.186 mL). The reaction mixture was stirred for 2-6 h at rt. Then, the reaction was quenched by the addition of ice-cold water extracted with DCM, and the combined organic layers were washed with brine and dried over anhydrous Na_2SO_4. The solvent was removed under reduced pressure to give Adamantan-1-yl)(methyl)sulfane as colourless whose NMR matches with the literature report.[17]

^{1}H NMR (500 MHz, $CDCl_3$) *δ* 2.03 (dd, *J* = 6.6, 3.1 Hz, 2H), 2.00 (s, 3H), 1.83 (d, *J* = 2.9 Hz, 6H), 1.74 – 1.60 (m, 6H).

^{13}C NMR (126 MHz, CDCl3) *δ* 42.90, 42.83, 36.51, 29.72,8.92.

HR-MS(ESI$^+$) calc. for $C_{11}H_{18}S$ [M+H]$^+$ 183.1207, found 183.0808.

14. Synthesis of N-[4-(methyl Thio)phenyl] Pivalamide (15a)

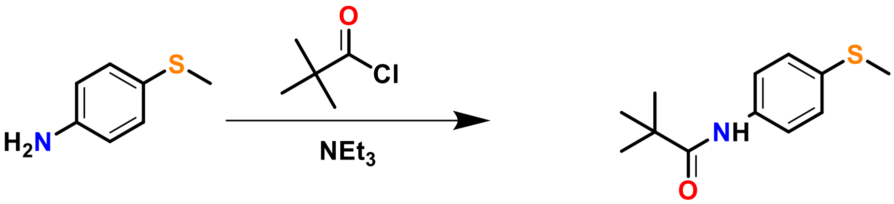

Pivolyl chloride (0.529mL, 4.3 mmol ,1.2 equiv.) was added to a solution of 4-(methyl-thio) aniline (0.44 mL, 3.59 mmol,) and triethylamine (0.649 mL,4.667mmol) in CH_2Cl_2 (30 mL) at 0 °C. The reaction mixture was warmed to room temperature and stirred for 1 h. Then the reaction mixture was diluted with a saturated aqueous ammonium chloride solution (15 mL) and extracted with CH_2Cl_2 (ca. 10 mL × 3). Finally, the combined organic layer with brine (20 mL) and dried over Na_2SO_4, filtered, concentrated under reduced pressure. The crude residue was triturated with Et_2O (2 mL) and n-hexane (10 mL) to obtain N-[4-(methylThio)phenyl] Pivalamide as colorless solid (623 mg, 2.7 mmol,77%) whose NMR matches with the literature report. [14]

^{1}H NMR (500 MHz, $CDCl_3$) *δ* 7.47 (d, *J* = 8.7 Hz, 2H), 7.24 (d, *J* = 8.9 Hz, 2H), 2.46 (s, 3H), 1.31 (s, 9H).

^{13}C NMR (125 MHz, $CDCl_3$) *δ* 176.67, 135.79, 133.52, 128.10, 120.68, 39.73, 27.76, 16.88.

HR-MS(ESI$^+$) calc. for $C_{12}H_{17}NOS$ $[M+H]^+$ 224.1109 , found 224.1197.

Spectral Data (XXXII)

1. ^{1}H NMR (500 MHz, $CDCl_3$)- Methyl phenyl sulfoxide (1b)

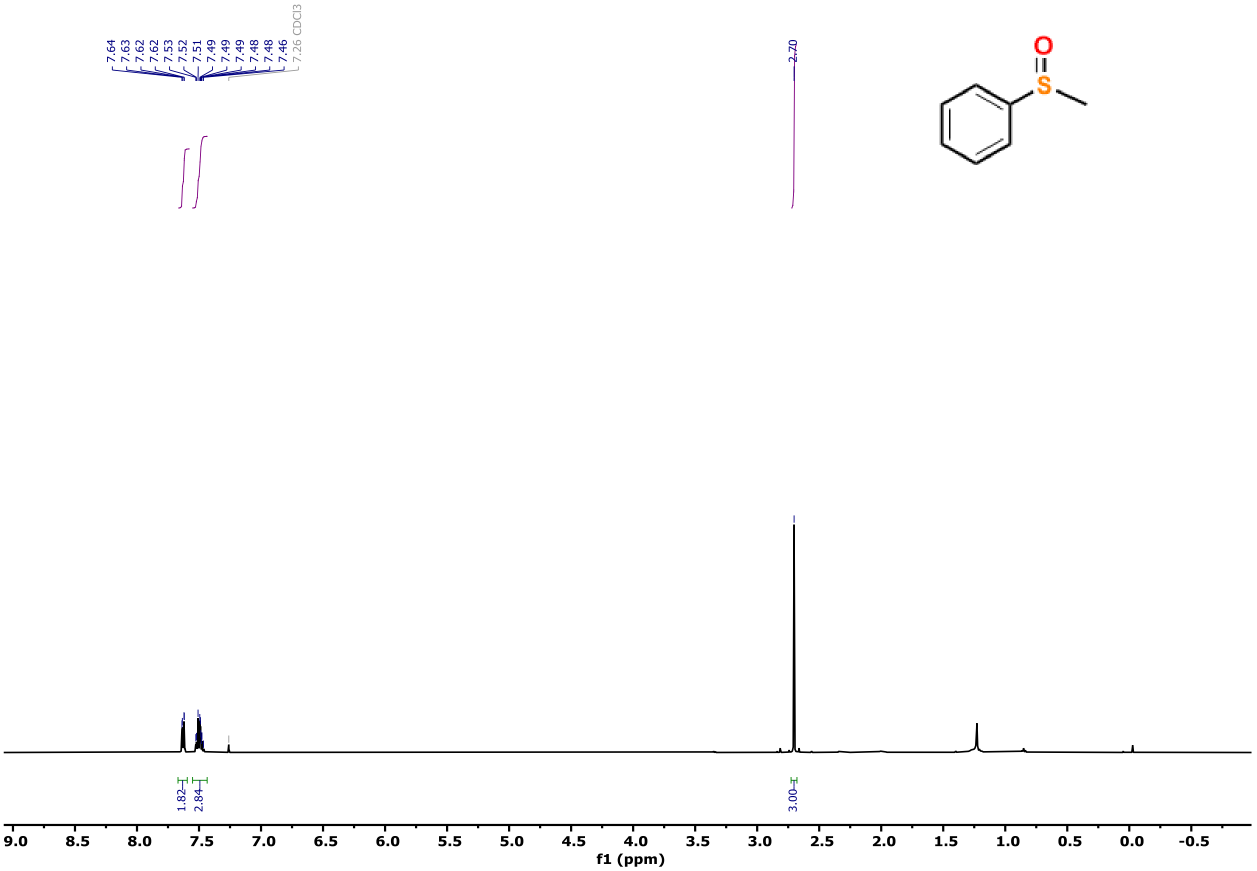

^{13}C NMR (126 MHz, $CDCl_3$)- Methyl phenyl sulfoxide (1b)

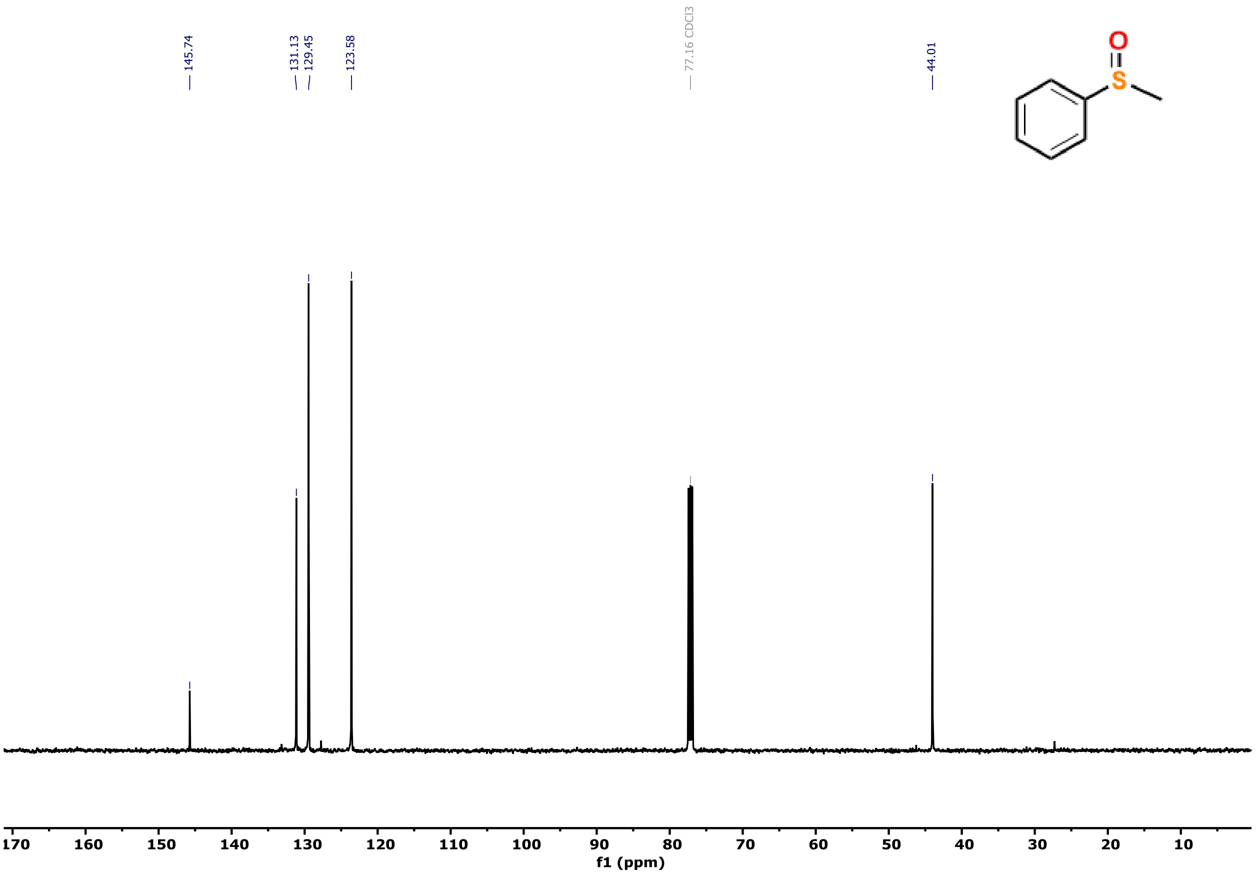

2. ^{1}H NMR (500 MHz, $CDCl_3$)- 4-Methylphenyl methyl sulfoxide (2b)

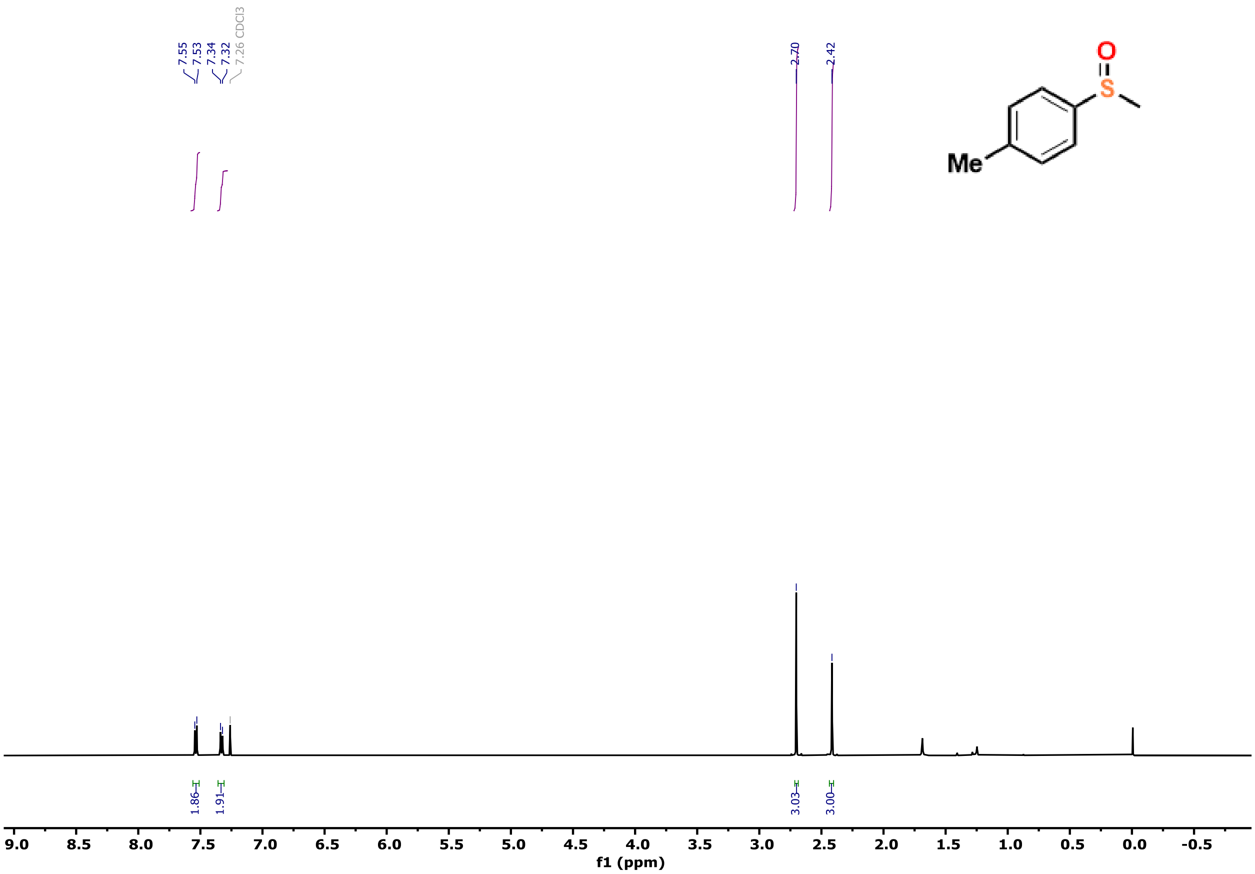

^{13}C NMR (126 MHz, $CDCl_3$)- 4-Methylphenyl methyl sulfoxide (2b)

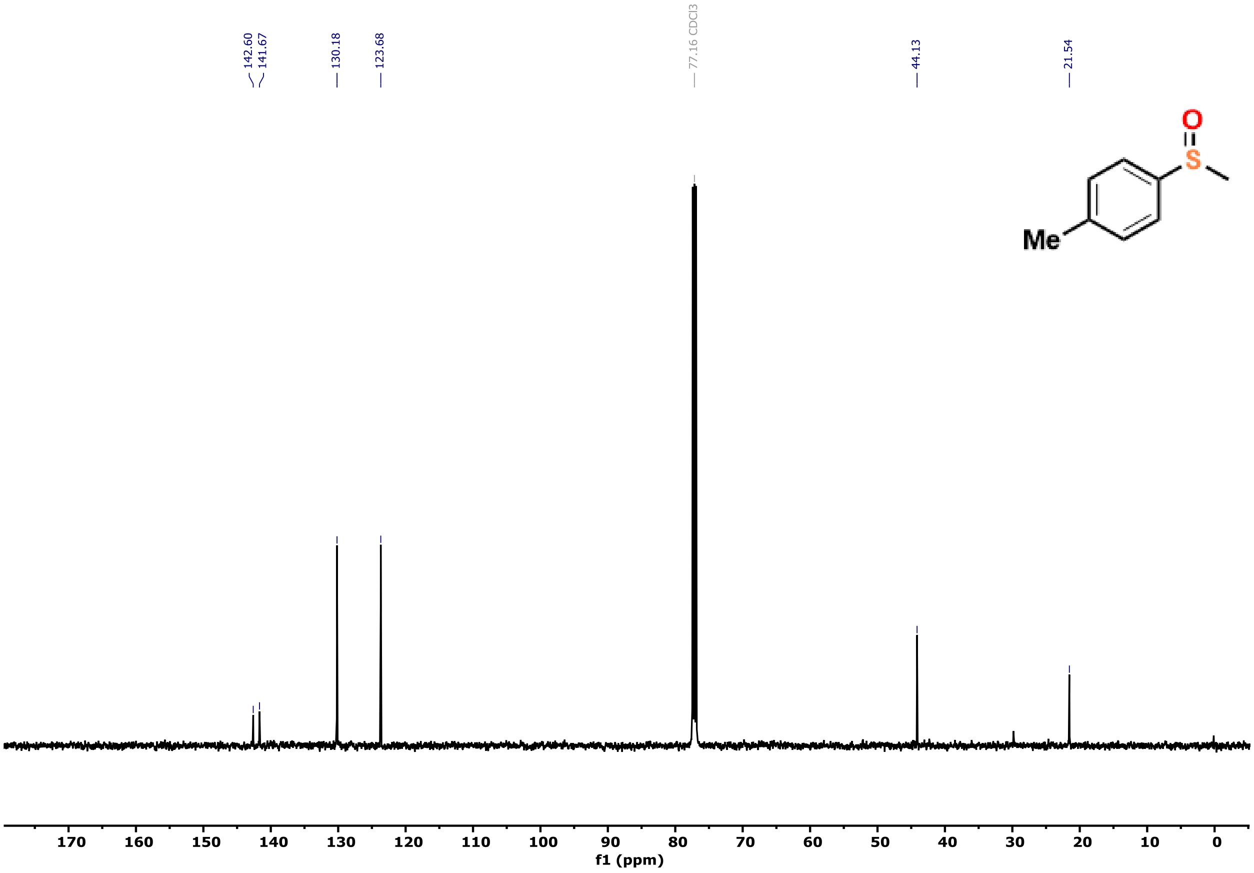

3. ^{1}H NMR (500 MHz, $CDCl_3$)- 4-Methoxyphenyl methyl sulfoxide (3b)

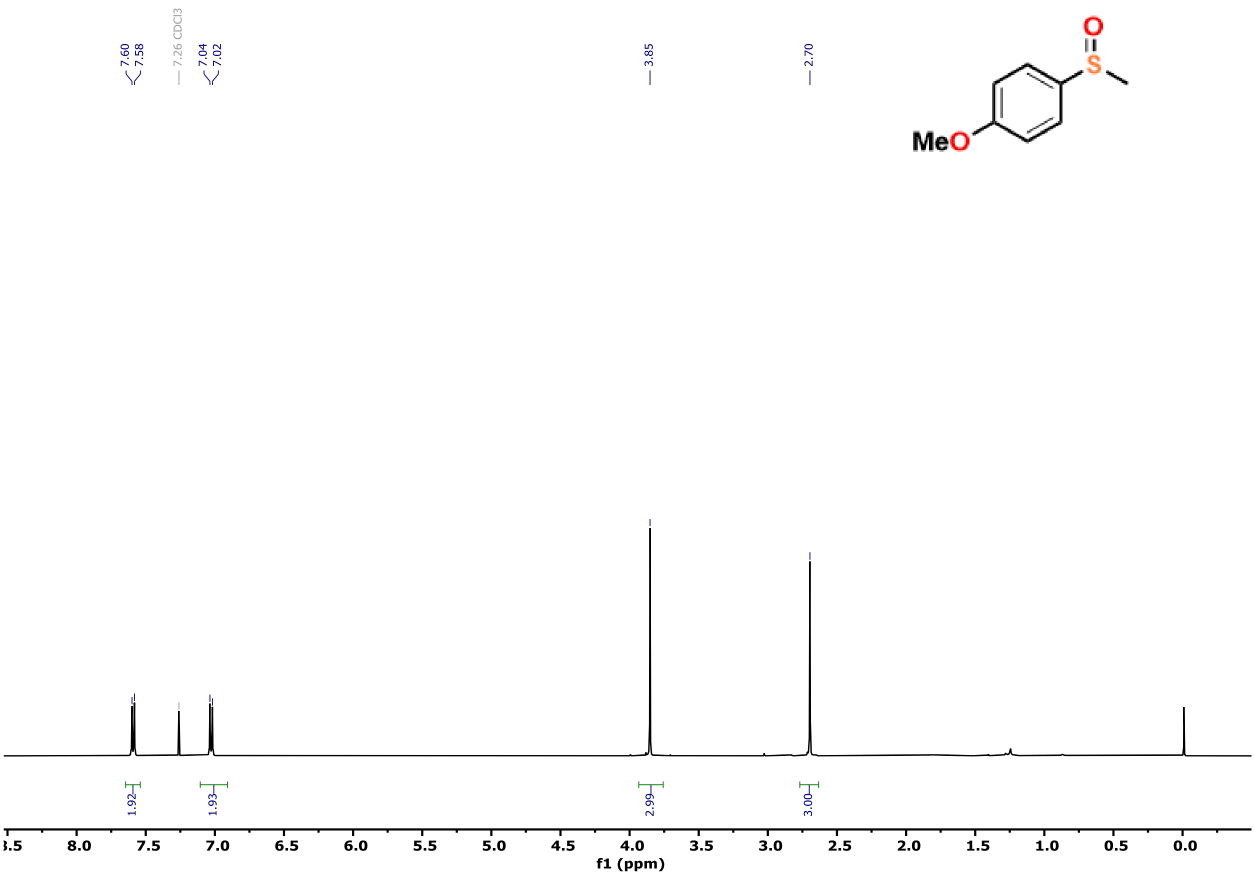

^{13}C NMR (126 MHz, $CDCl_3$)- 4-Methoxyphenyl methyl sulfoxide (3b)

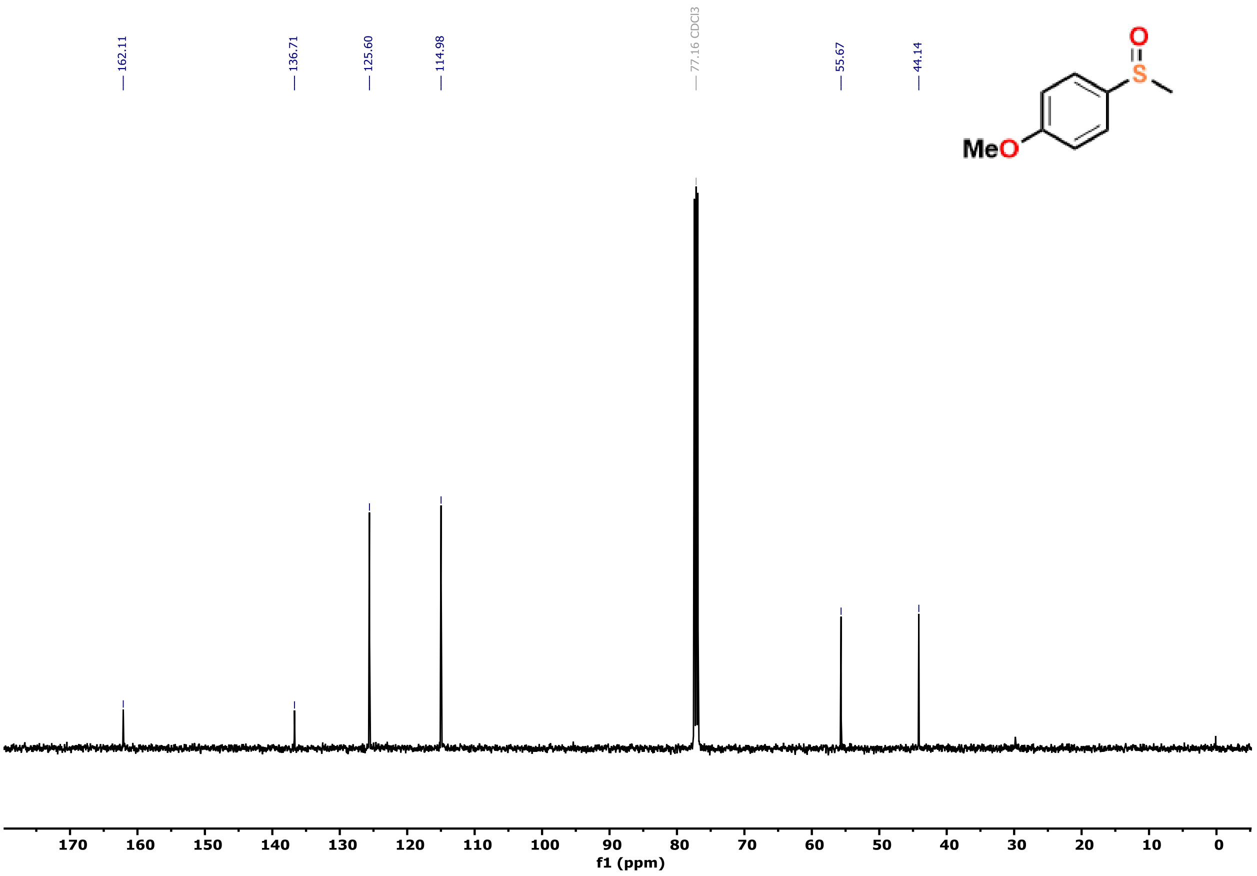

4. ^{1}H NMR (500 MHz, $CDCl_3$)- (4-tert-butyl) phenylmethyl sulfoxide (4b)

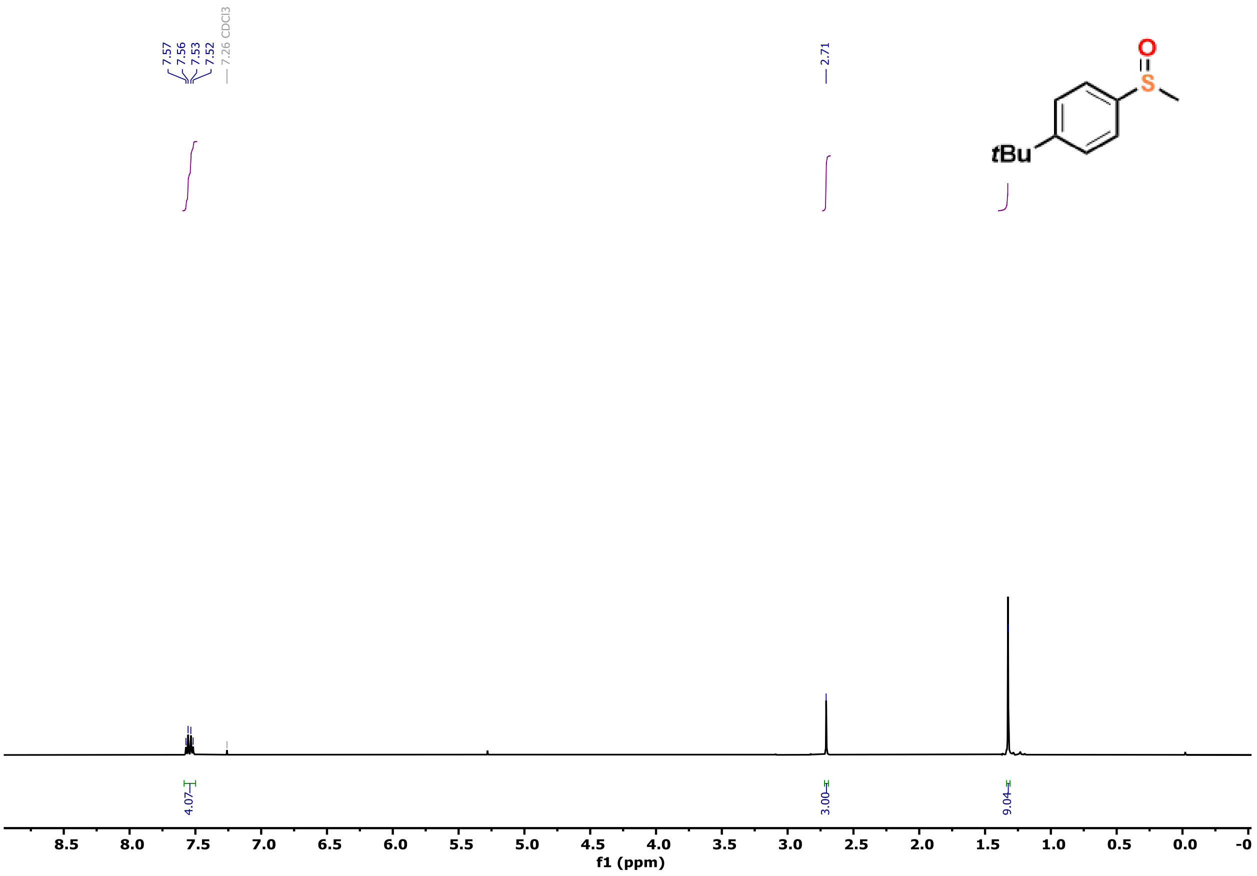

^{13}C NMR (126 MHz, $CDCl_3$)- (4-tert-butyl)phenylmethyl sulfoxide (4b)

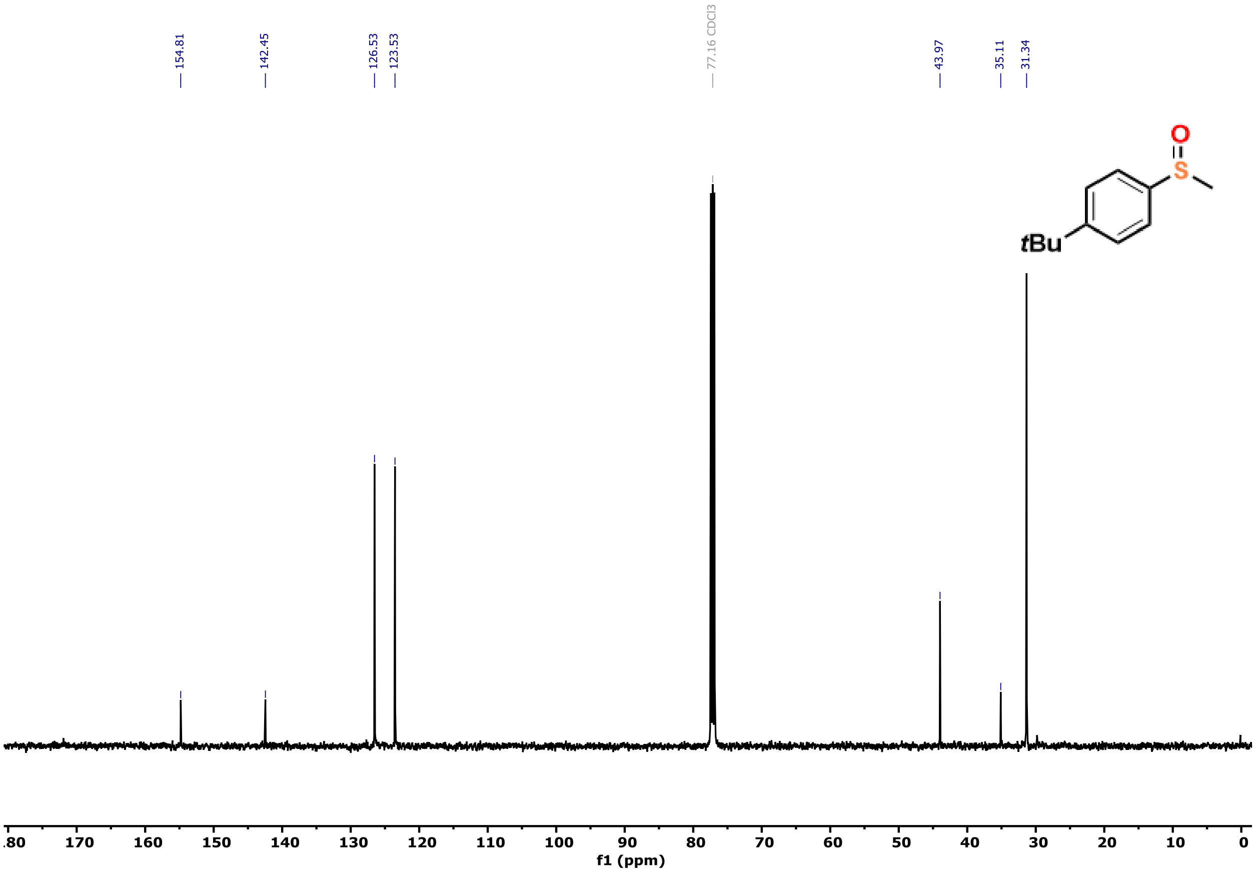

5) ^1H NMR (500 MHz, $CDCl_3$)- 2,6-Dimethylbenzenethiosulfoxide (5b)

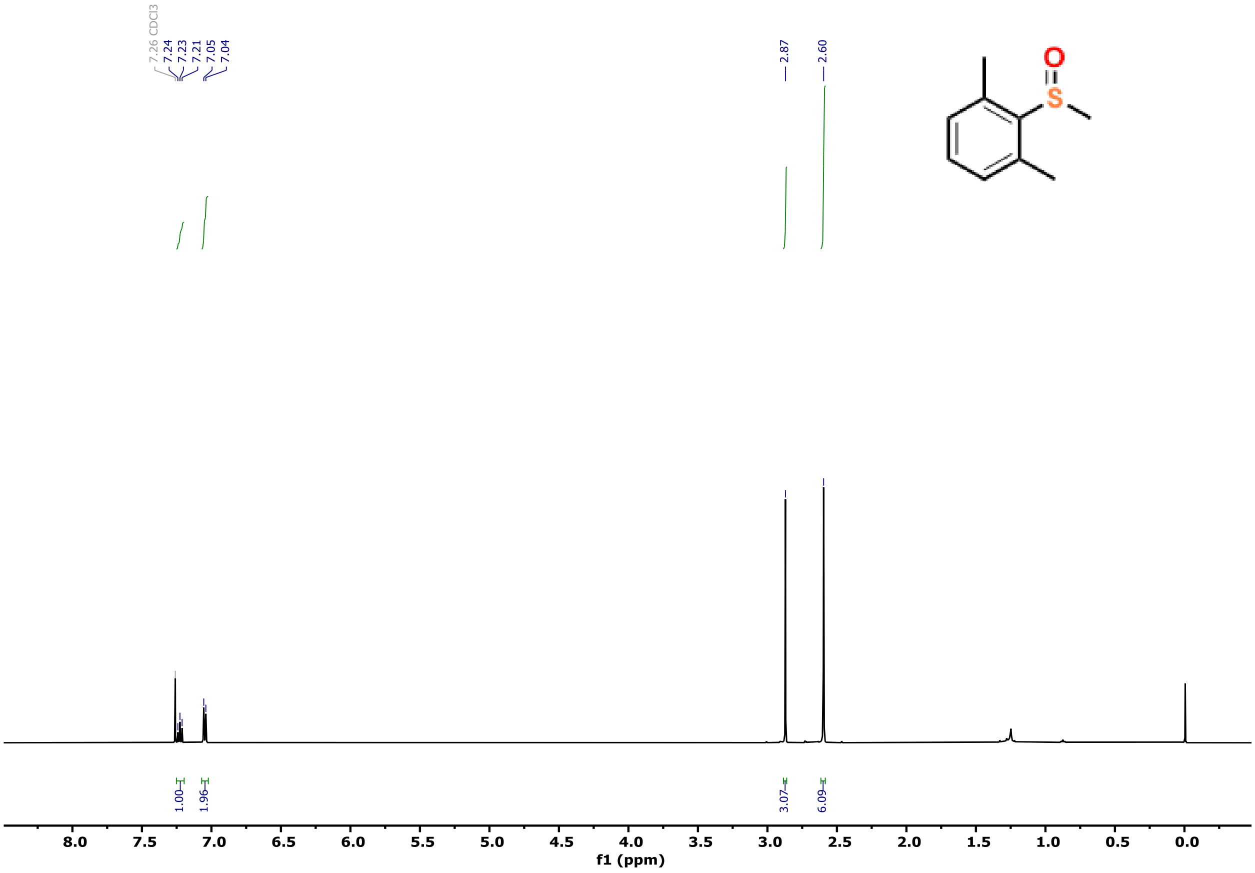

^{13}C NMR (126 MHz, $CDCl_3$)- 2,6-Dimethylbenzenethiosulfoxide (5b)

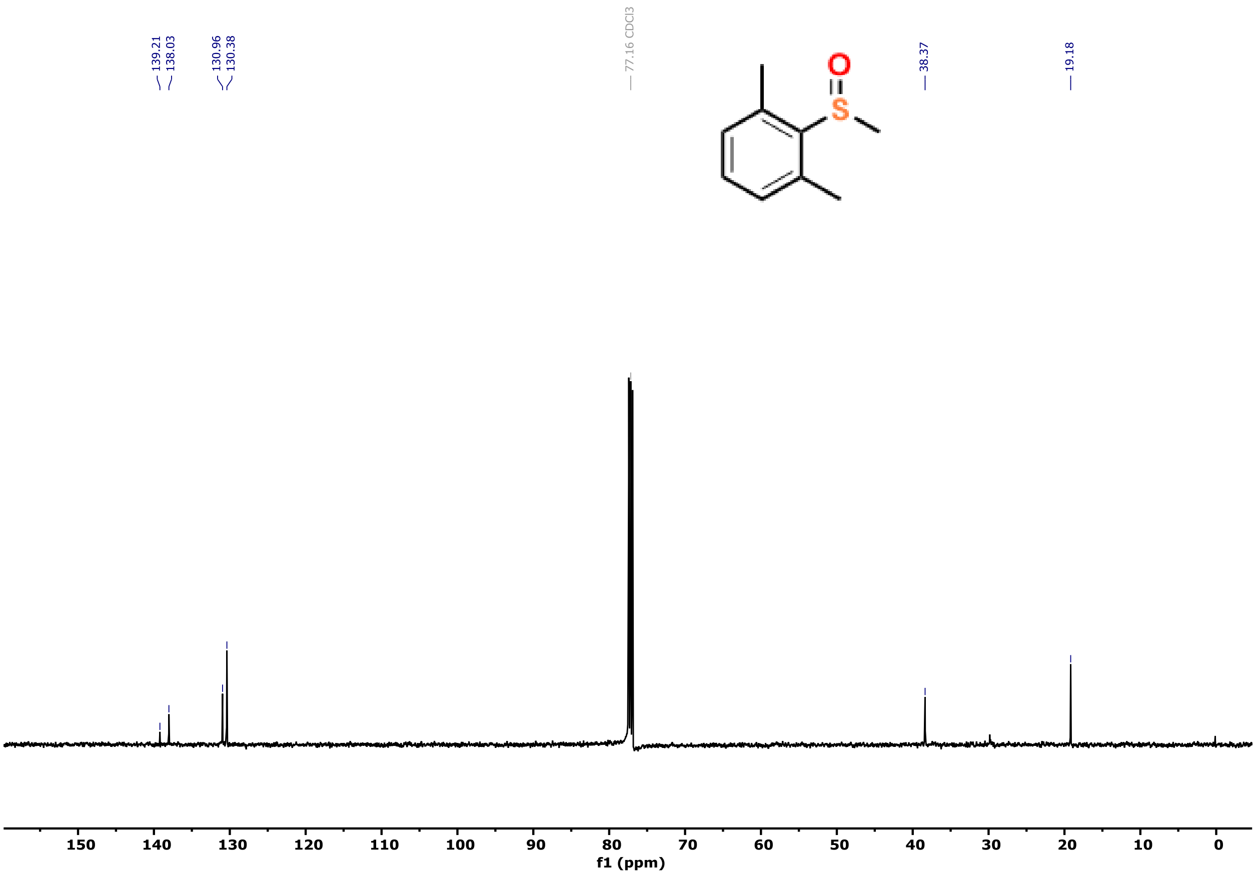

6. ^{1}H NMR (500 MHz, $CDCl_3$)- (methylsulfinyl)naphthalene sulfoxide (6b)

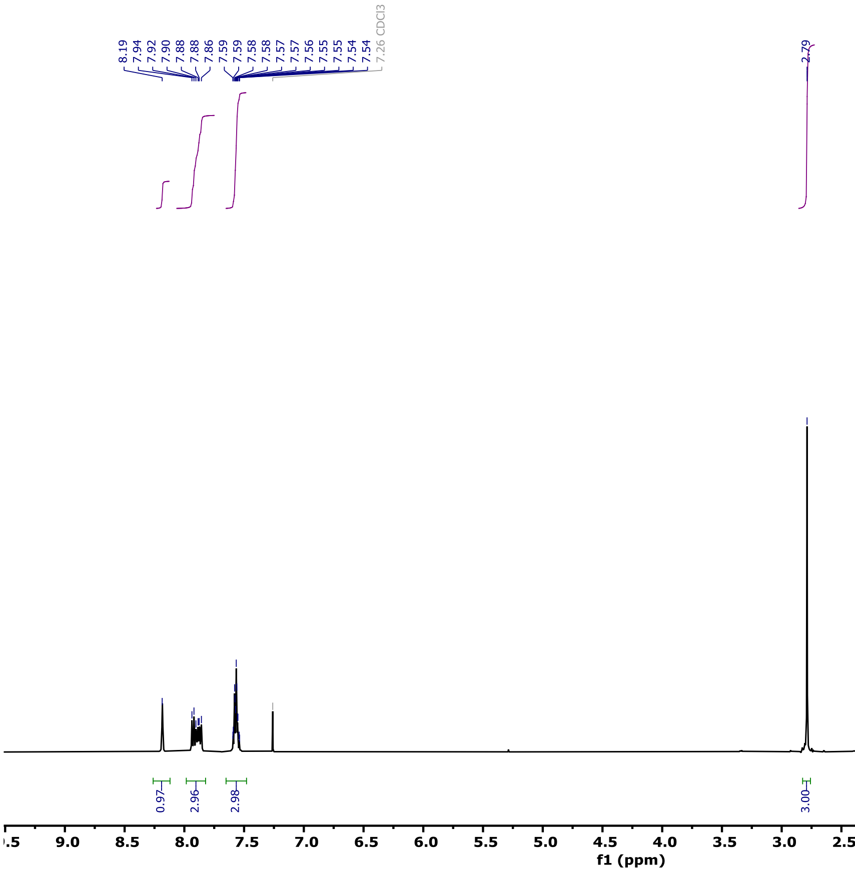

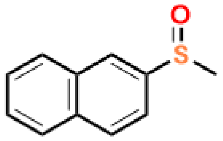

^{13}C NMR (126 MHz, $CDCl_3$)- (methylsulfinyl)naphthalene sulfoxide (6b)

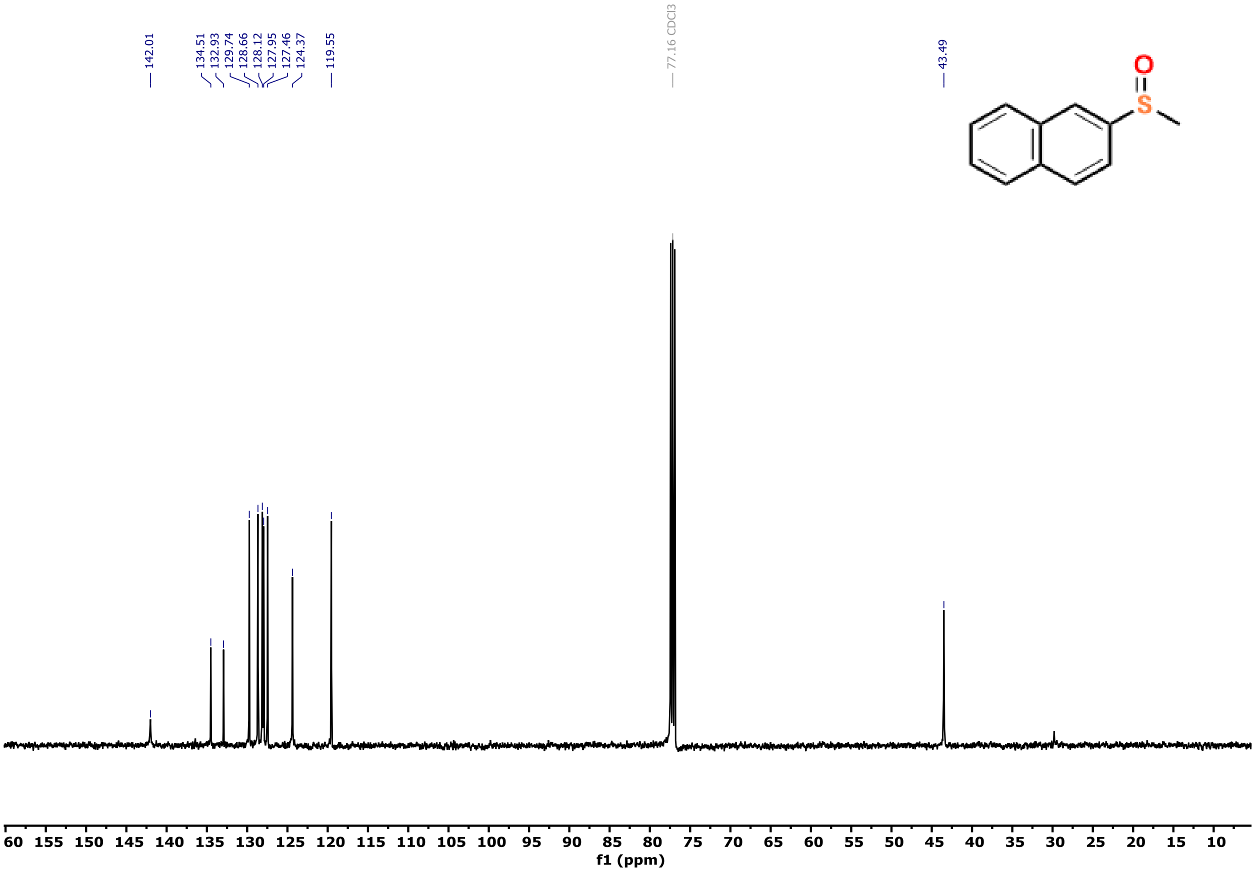

7. ^{1}H NMR (500 MHz, $CDCl_3$)- 4-Fluorophenyl methyl sulfoxide (7b)

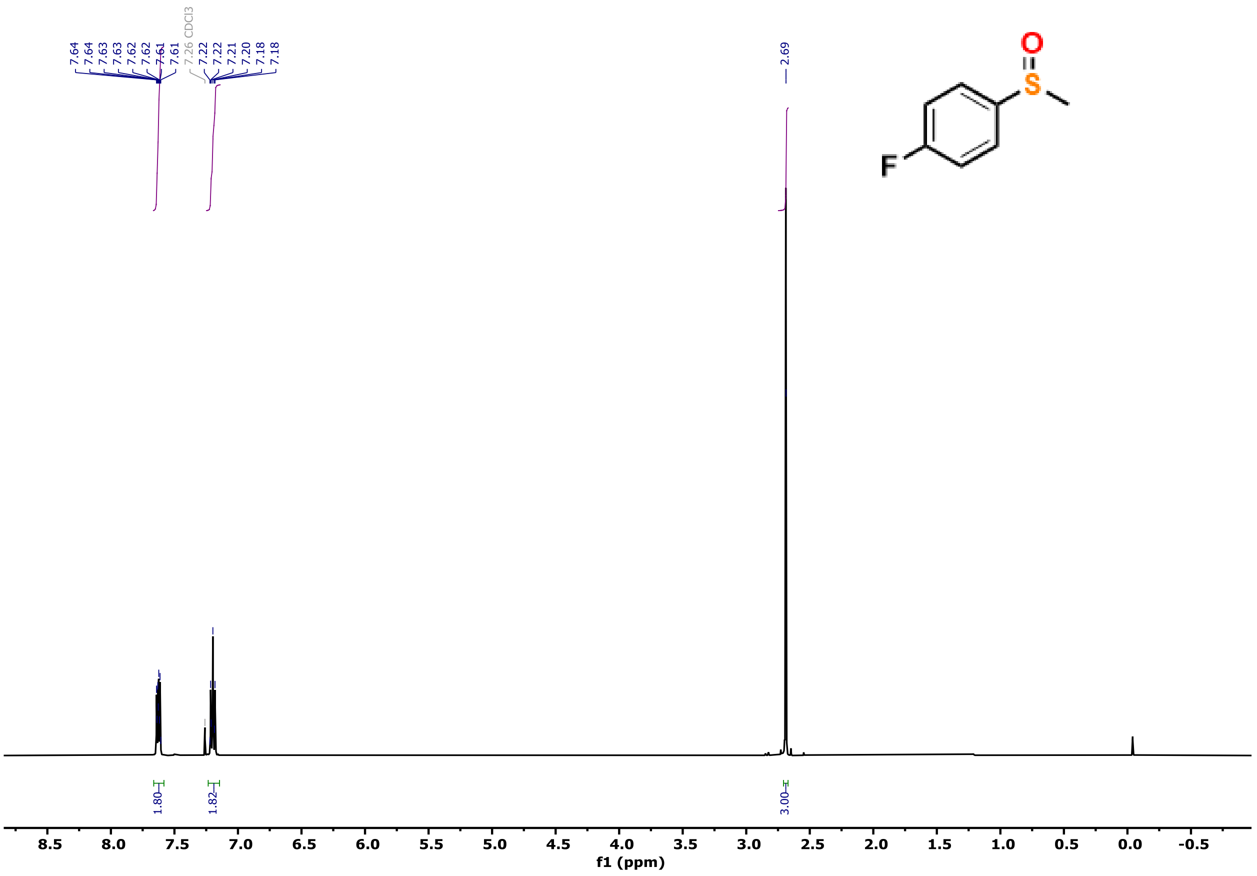

^{13}C NMR (126 MHz, $CDCl_3$)- 4-Fluorophenyl methyl sulfoxide (7b)

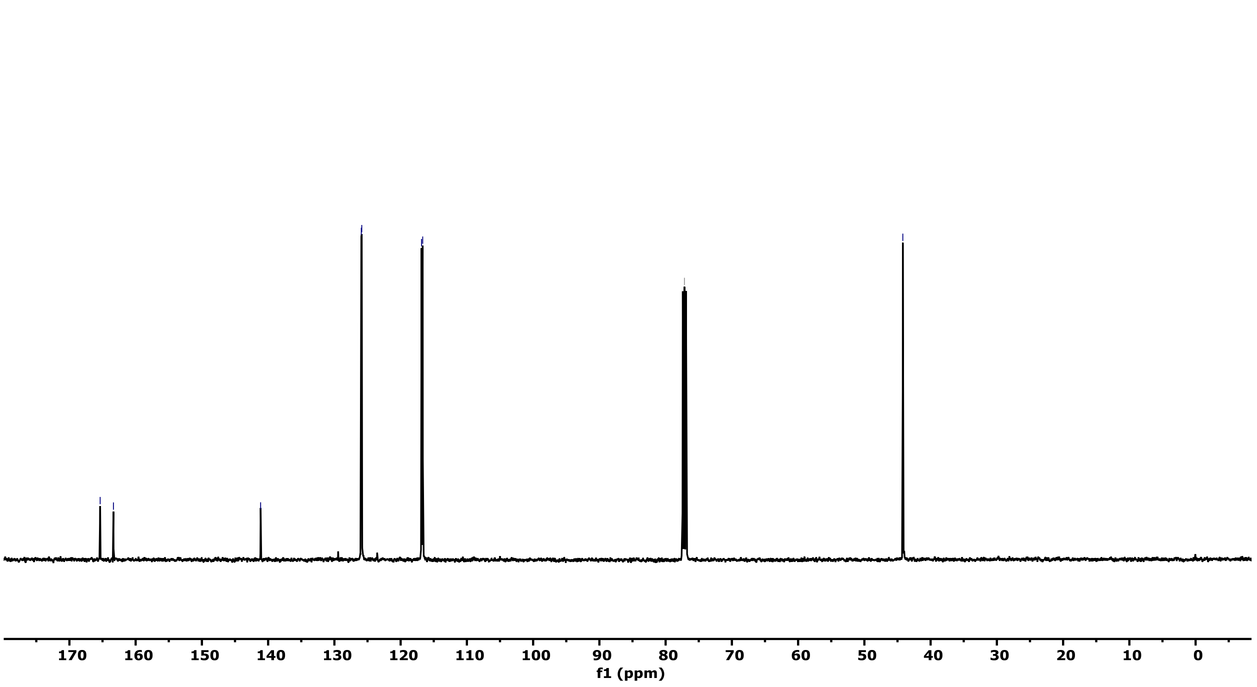

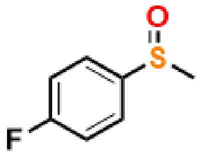

^{19}F NMR (471 MHz, $CDCl_3$)- 4-Fluorophenyl methyl sulfoxide (7b)

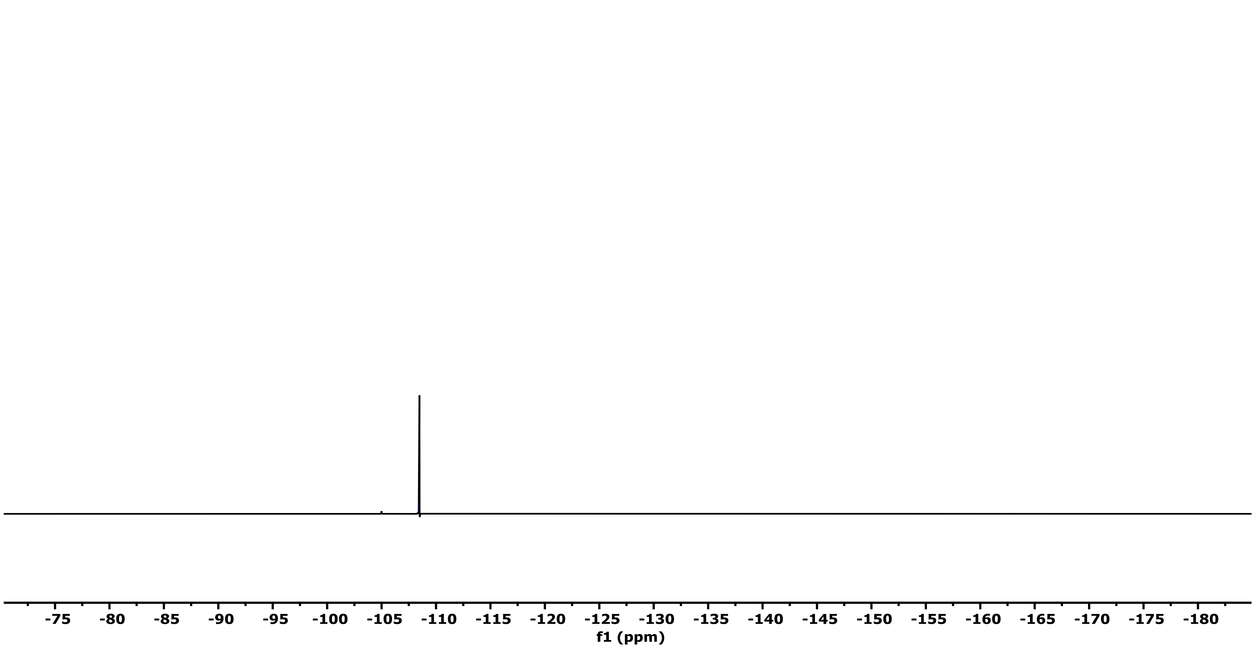

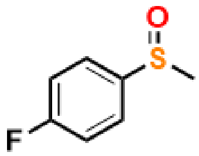

8. ^{1}H NMR (500 MHz, $CDCl_3$)- 4-Chlorophenyl methyl sulfoxide (8b)

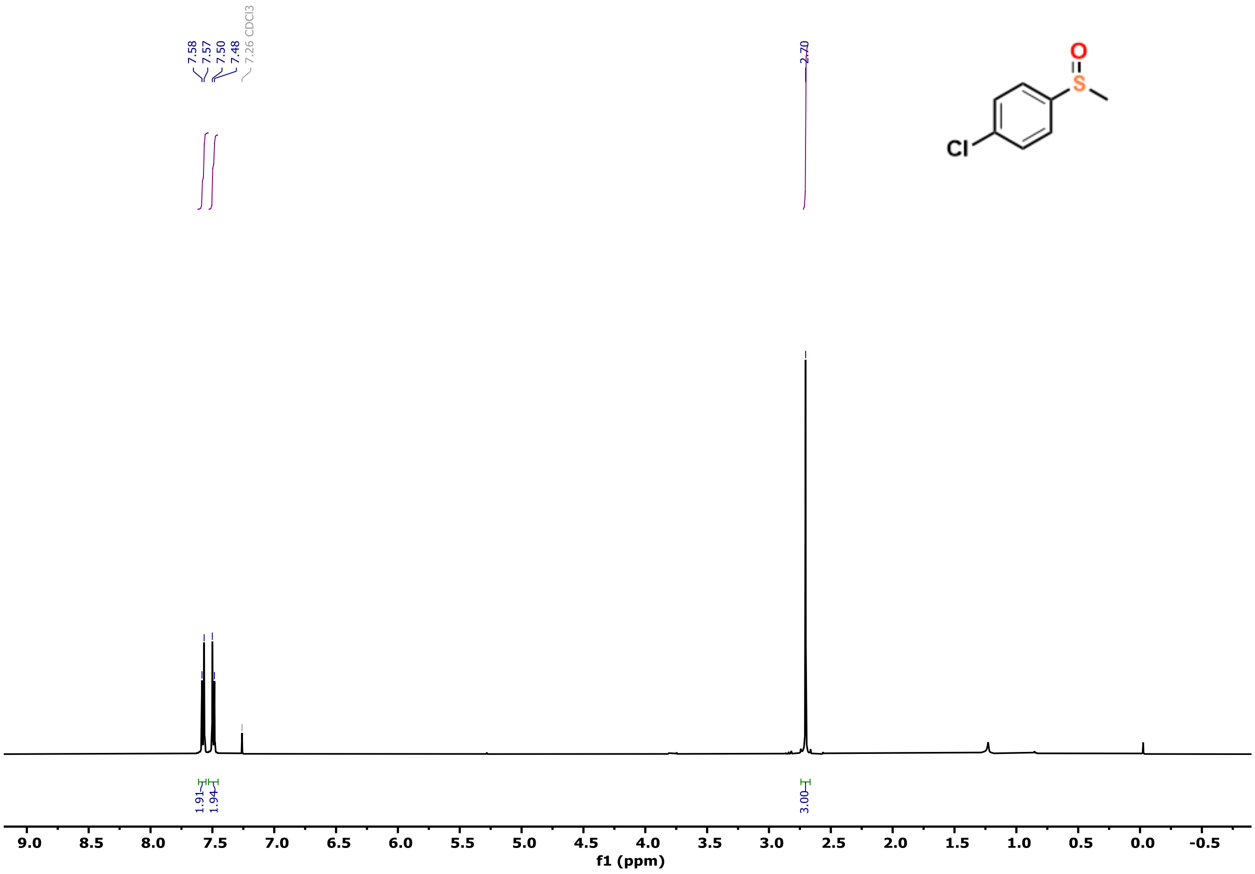

^{13}C NMR (126 MHz, $CDCl_3$)- 4-Chlorophenyl methyl sulfoxide (8b)

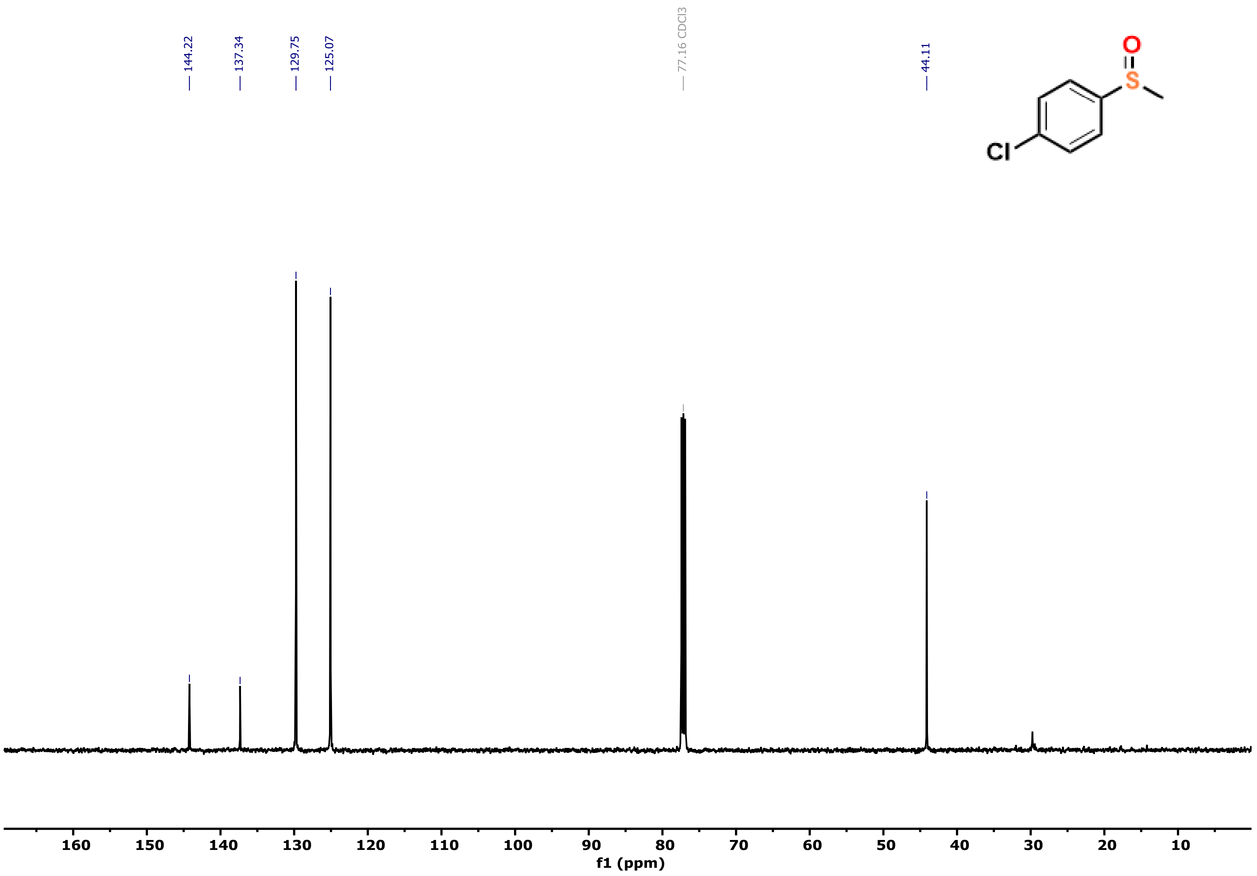

9.^{1}H NMR (500 MHz, $CDCl_3$)- 4-Bromophenyl methyl sulfoxide (9b)

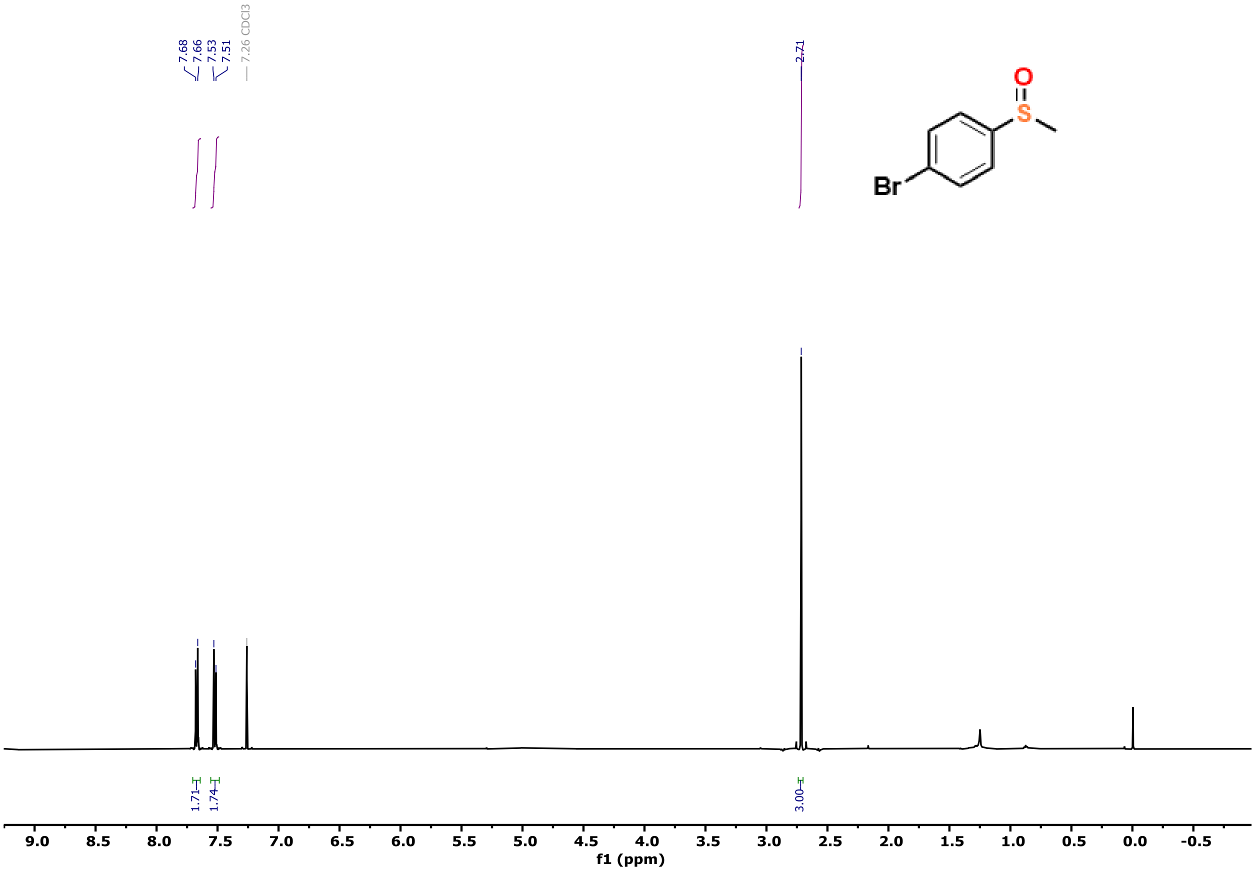

^{13}C NMR (126 MHz, $CDCl_3$)- 4-Bromophenyl methyl sulfoxide (9b)

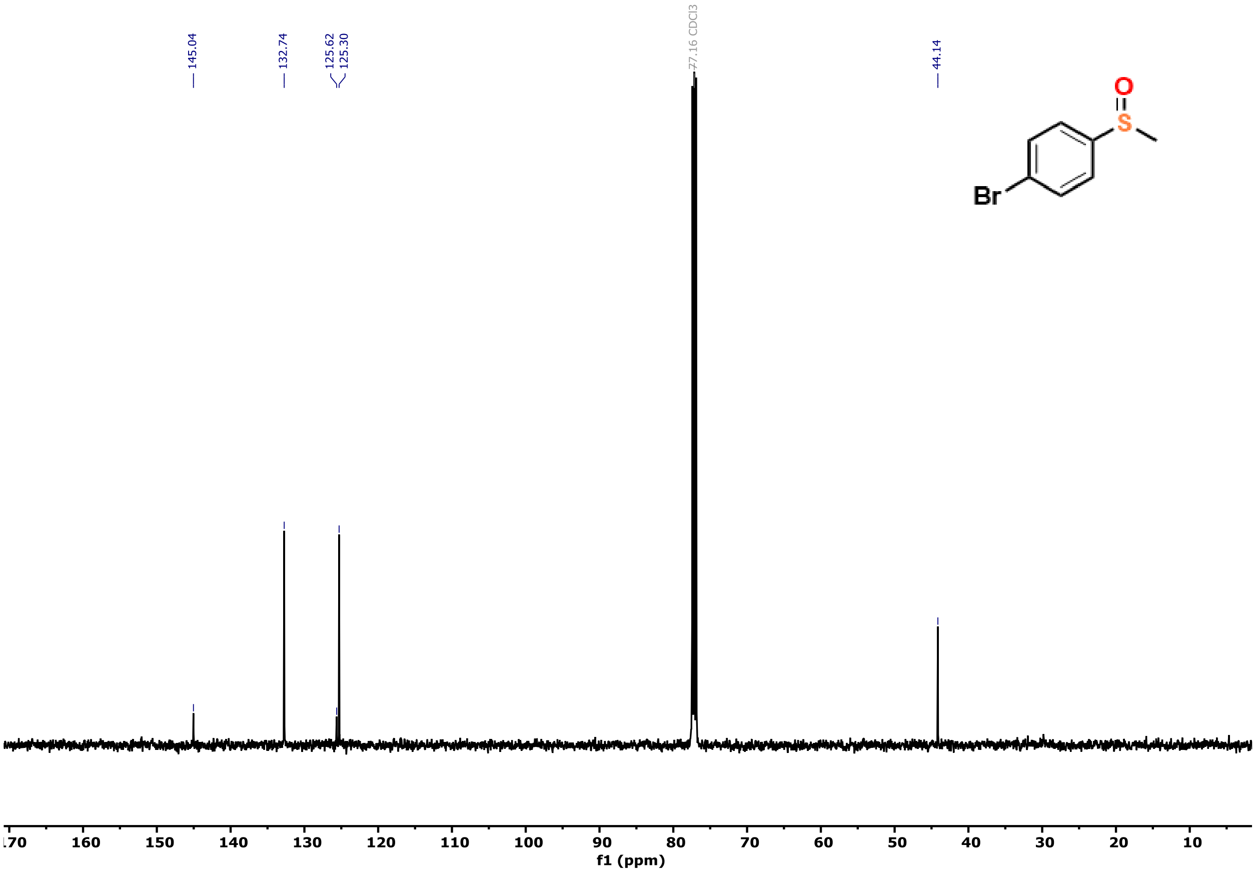

10. ^{1}H NMR (500 MHz, $CDCl_3$)- 2-Bromophenylsulfoxide (10b)

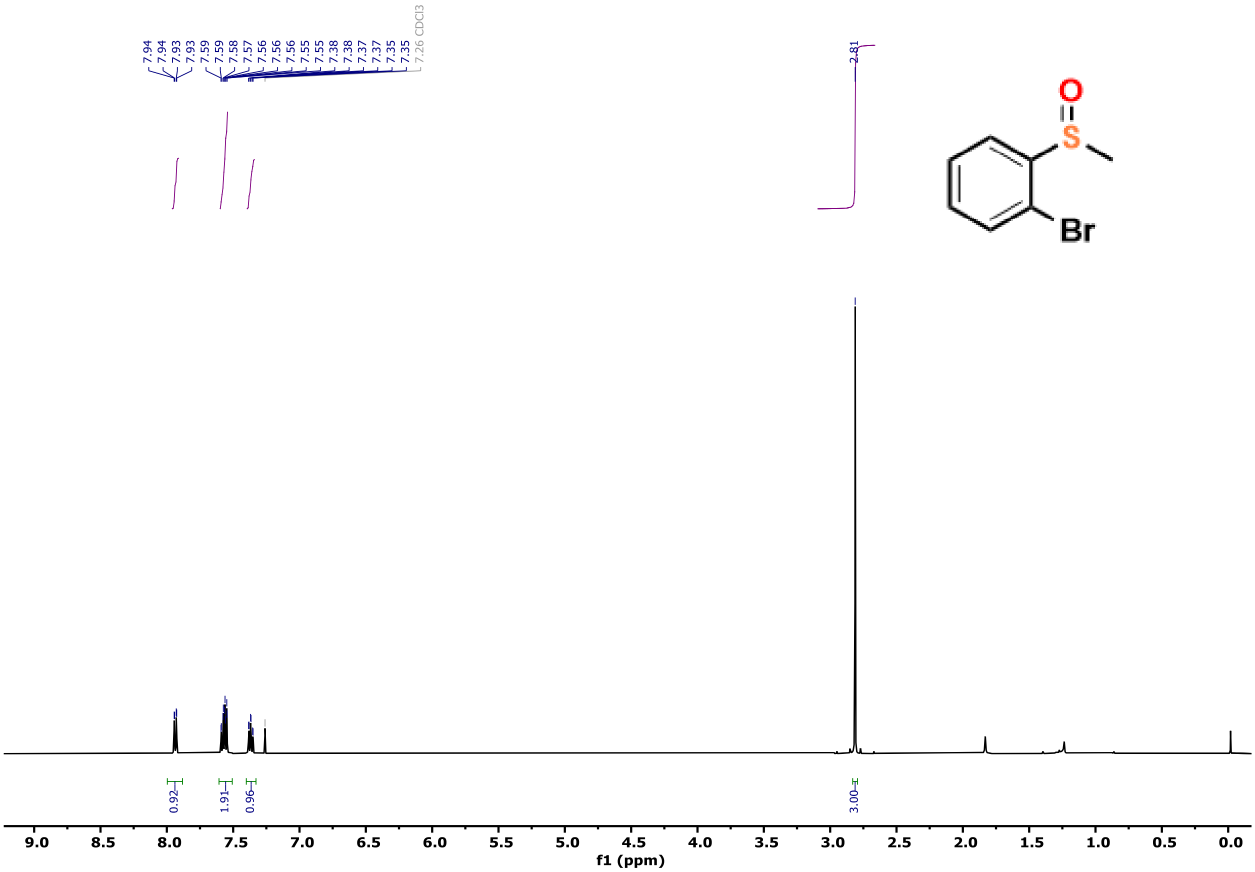

^{13}C NMR (126 MHz, $CDCl_3$)- 2-Bromophenylsulfoxide (10b)

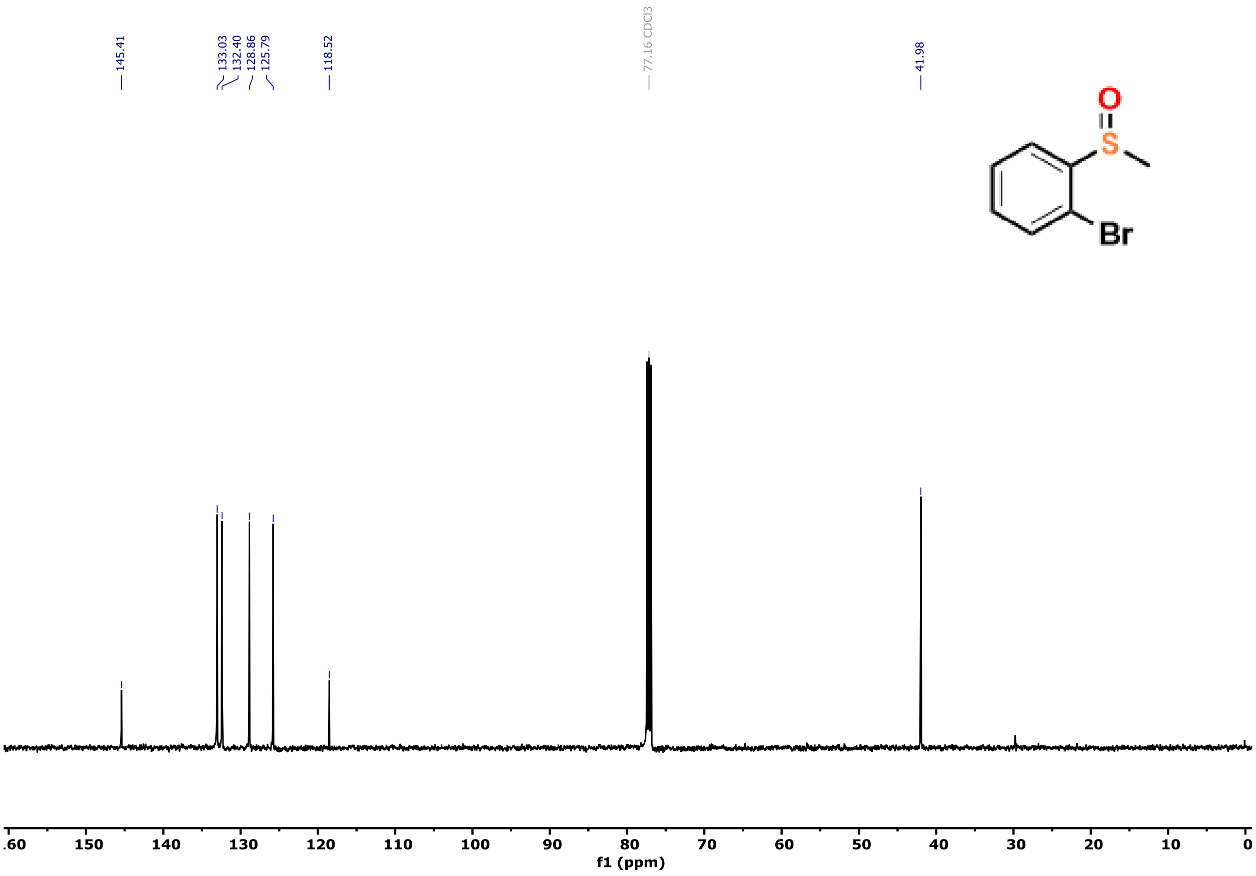

11. ^{1}H NMR (500 MHz, $CDCl_3$)- 4-Nitrilephenyl sulfoxide (11b)

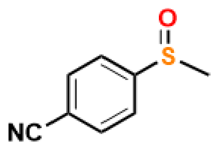

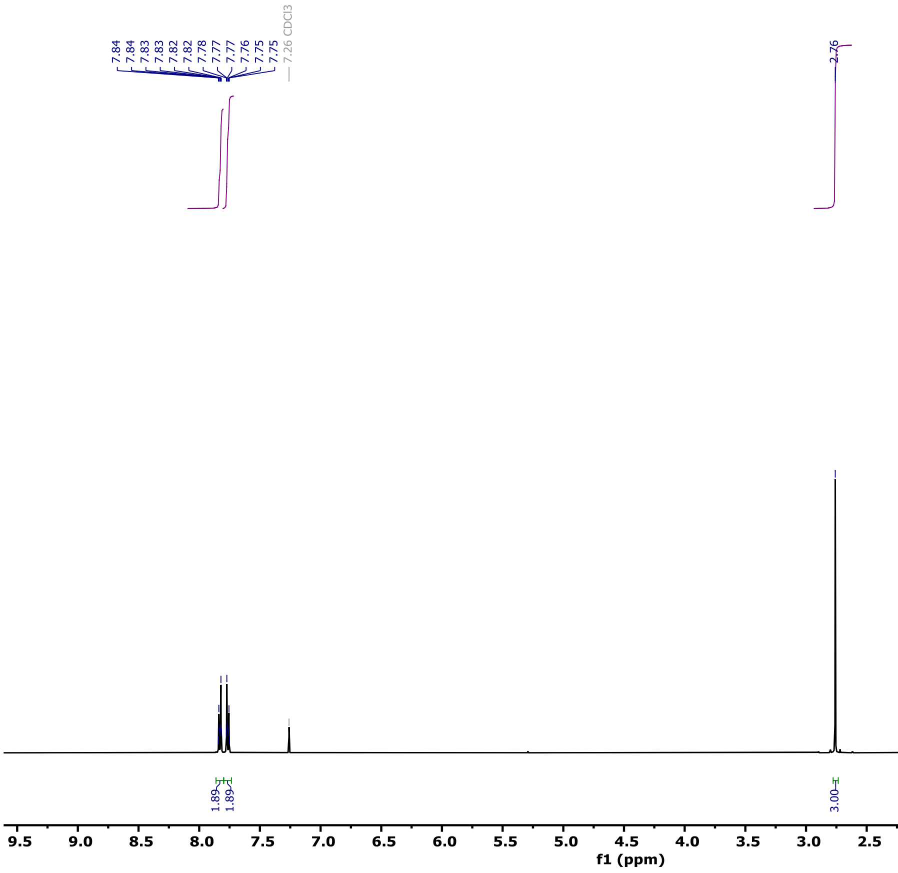

^{13}C NMR (126 MHz, $CDCl_3$)- 4-Nitrilephenyl sulfoxide (11b)

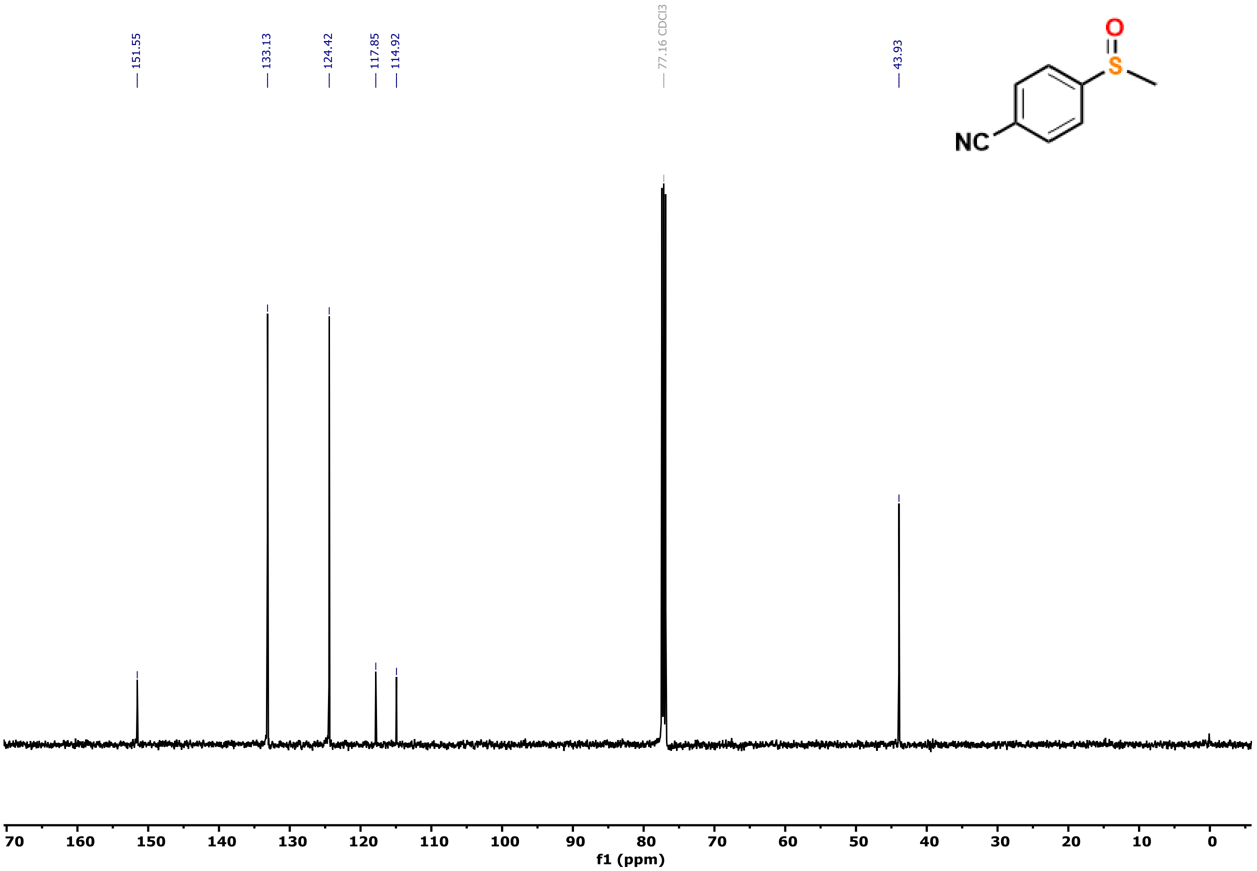

12. ^{1}H NMR (500 MHz, $CDCl_3$)- 4-Nitrophenyl methyl sulfoxide (12b)

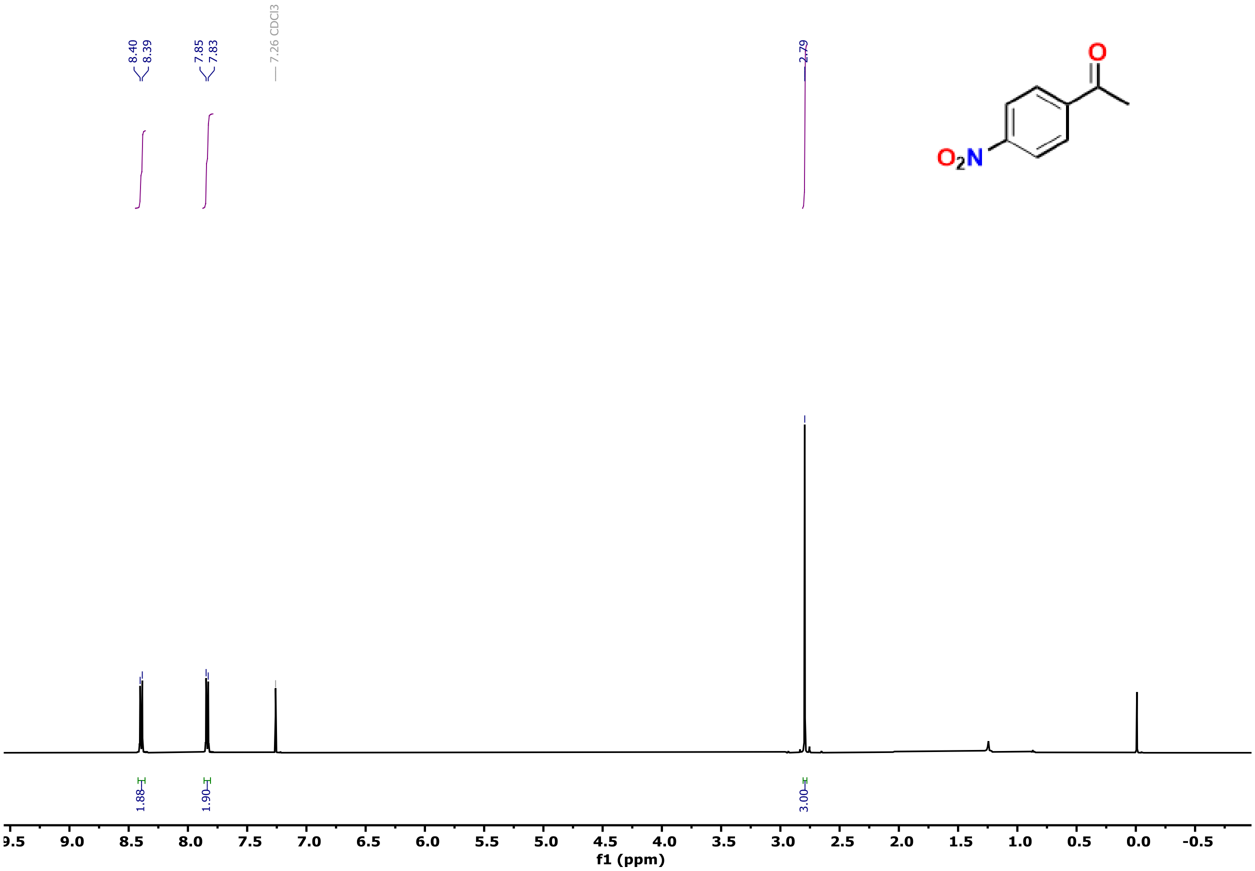

^{13}C NMR (126 MHz, $CDCl_3$)- 4-Nitrophenyl methyl sulfoxide (12b)

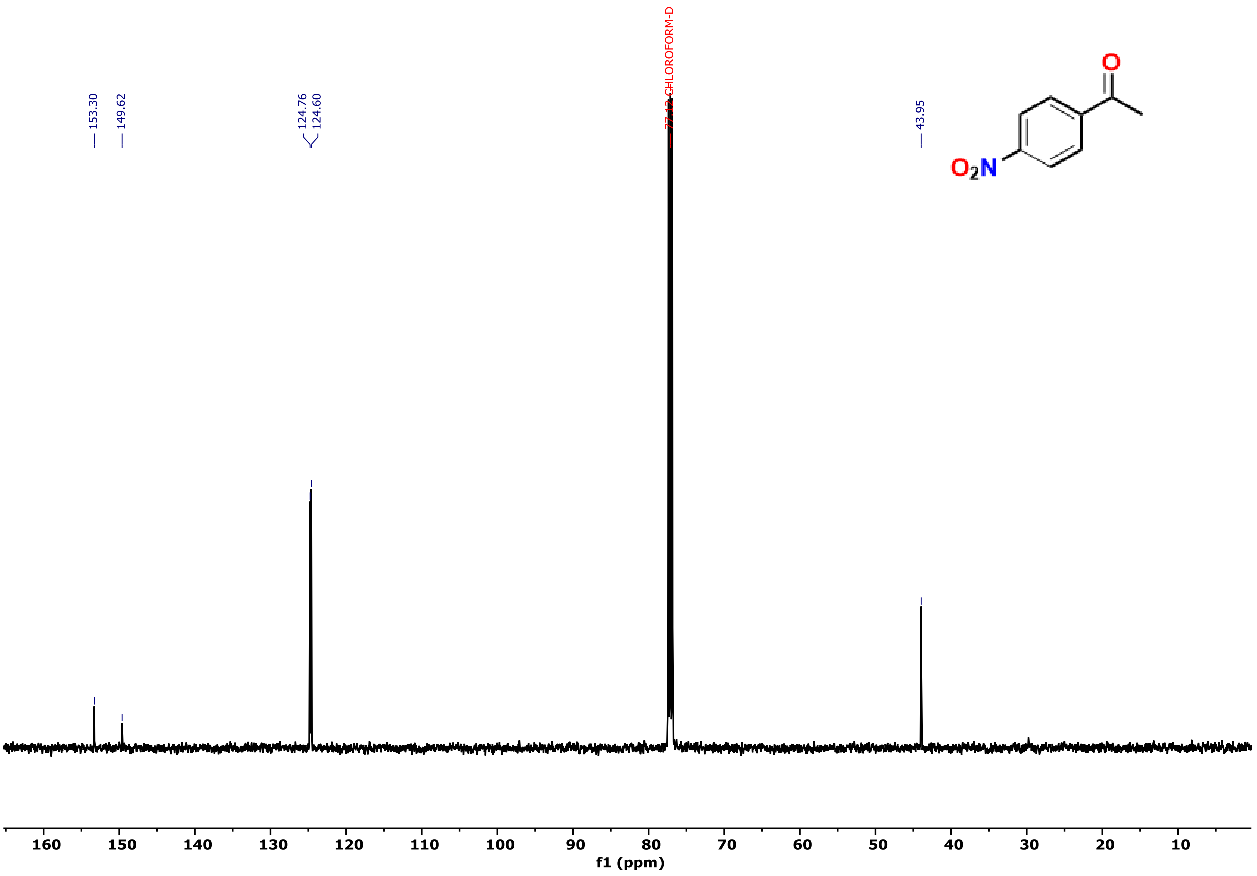

13. ^{1}H NMR (500 MHz, $CDCl_3$)- Methyl(4-(trifluoromethyl)phenyl) sulfoxide (13b)

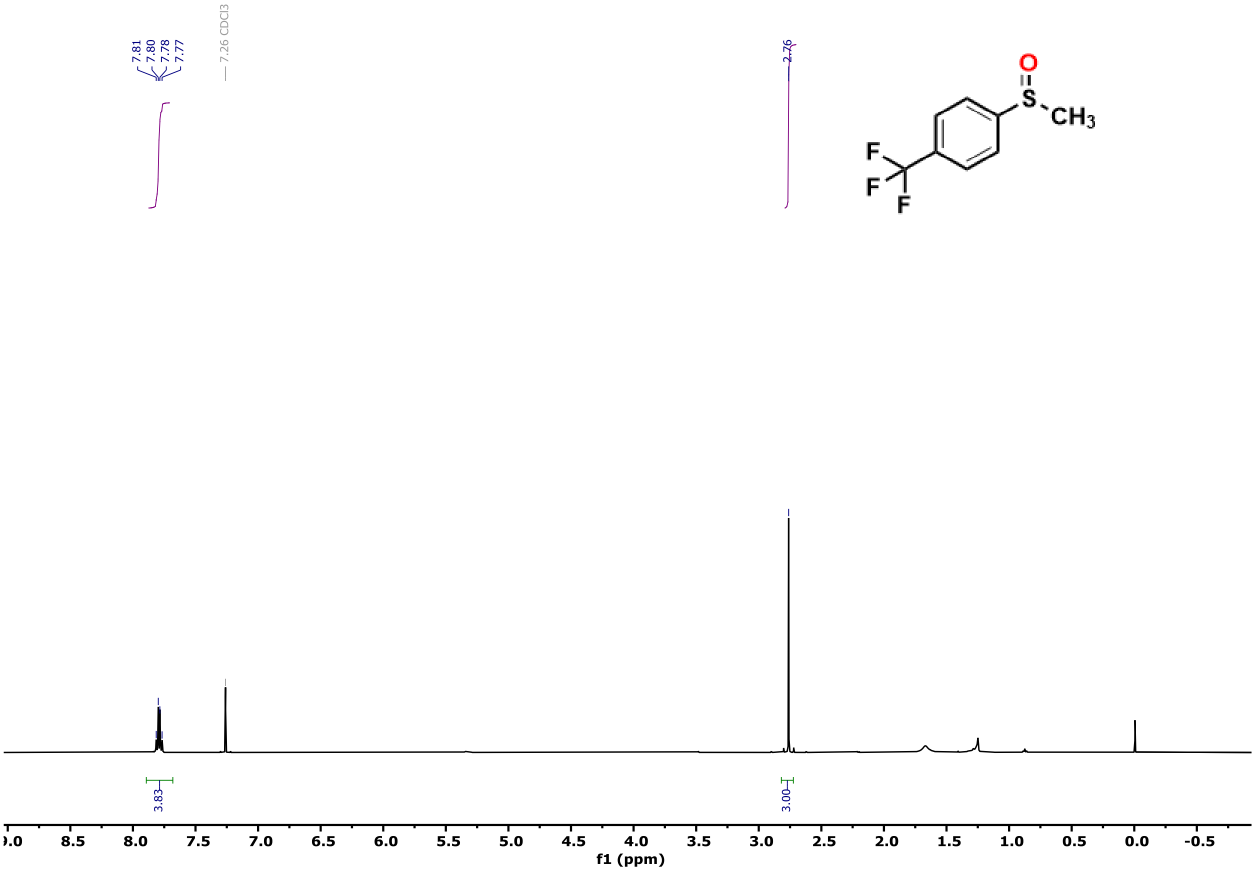

^{13}C NMR (126 MHz, $CDCl_3$)- Methyl(4-(trifluoromethyl)phenyl) sulfoxide (13b)

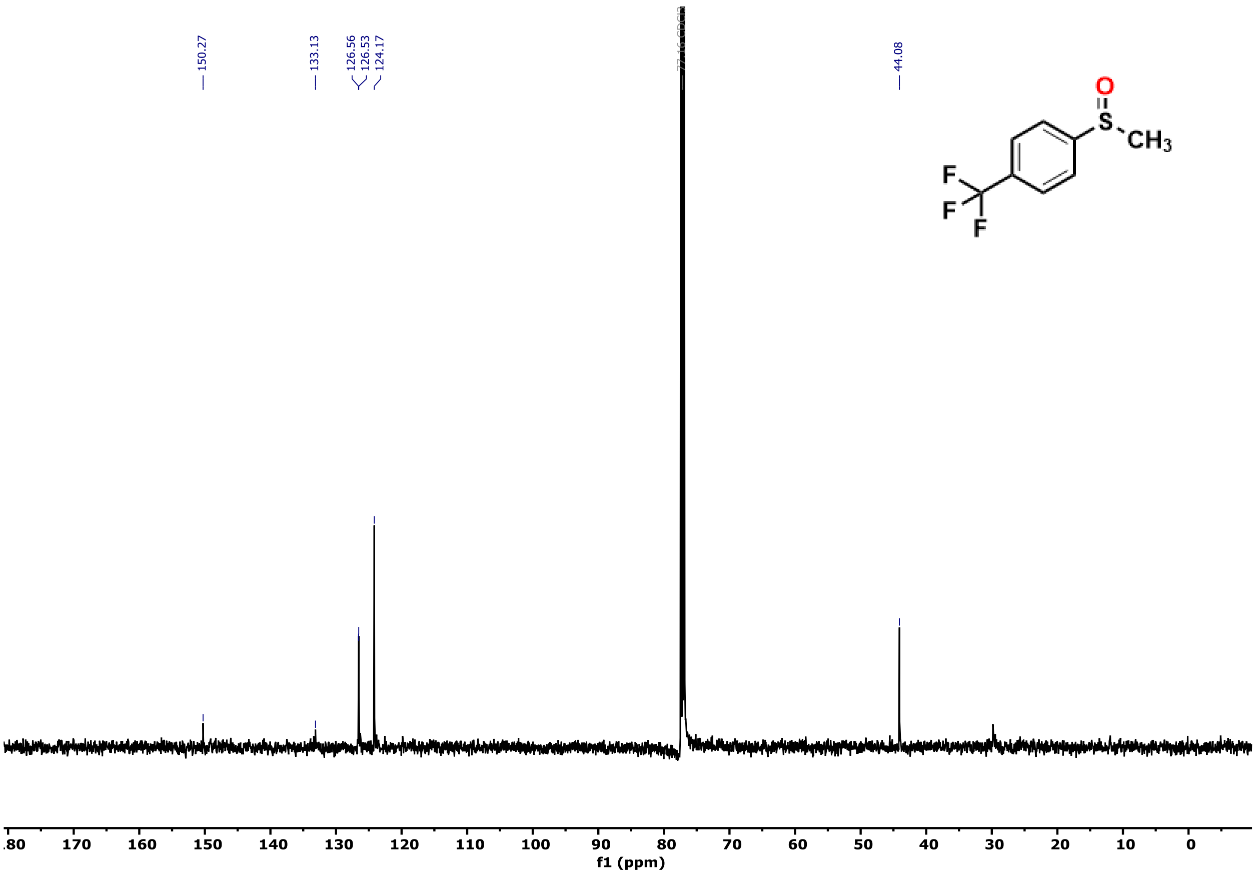

^{19}F NMR (471 MHz, $CDCl_3$)- Methyl(4-(trifluoromethyl)phenyl) sulfoxide (13b)

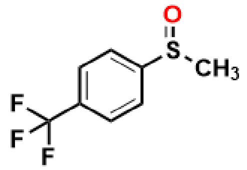

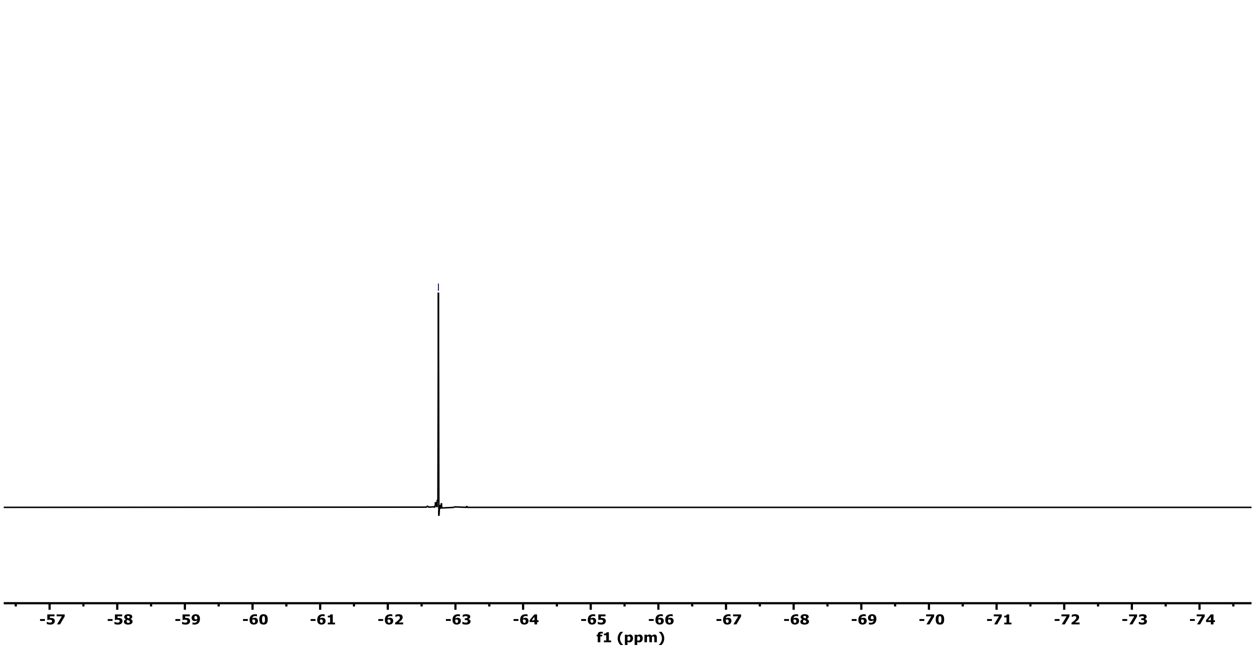

14. ^{1}H NMR (500 MHz, $CDCl_3$)-Benzene,1-(methylsulfinyl)-4-(trifluoromethoxy) (14b)

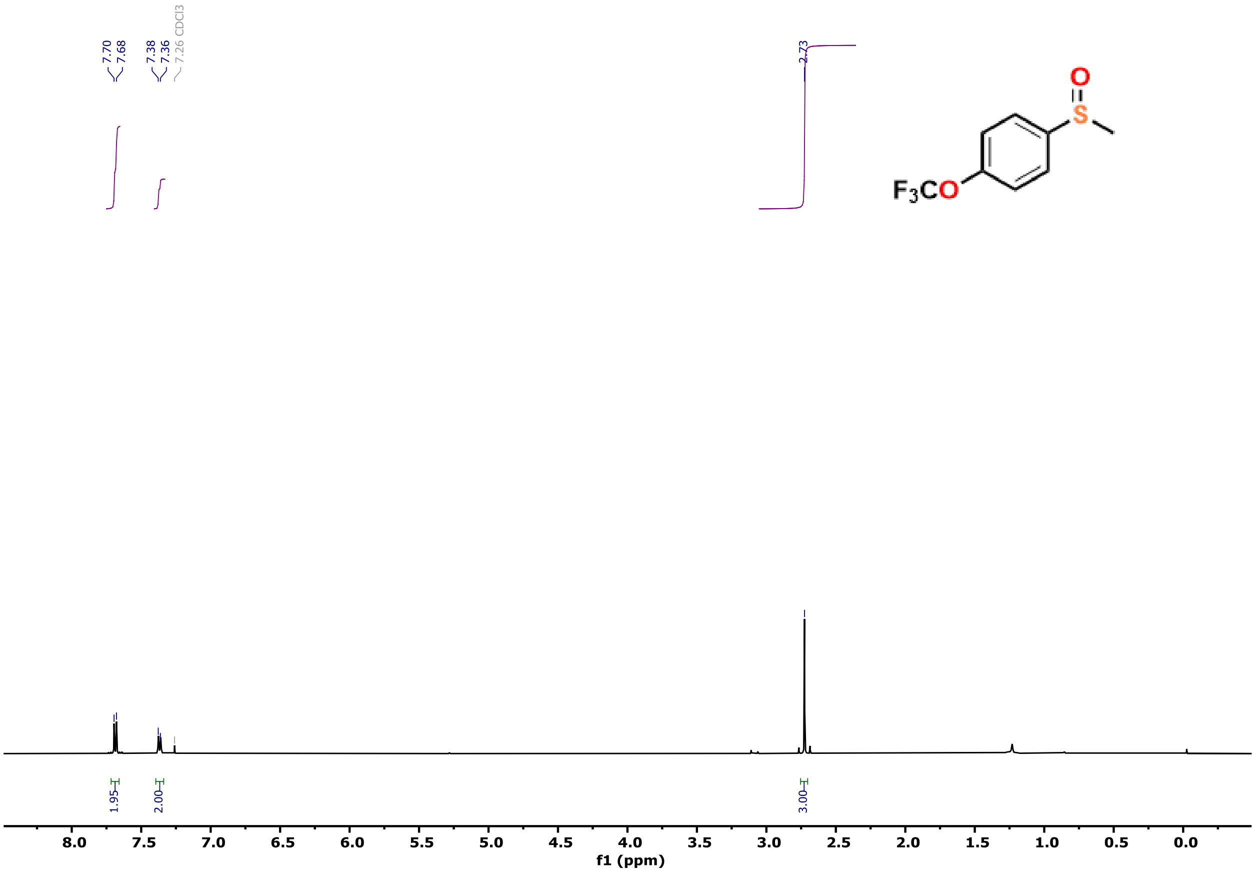

^{13}C NMR (126 MHz, $CDCl_3$)-Benzene,1-(methylsulfinyl)-4-(trifluoromethoxy) (14b)

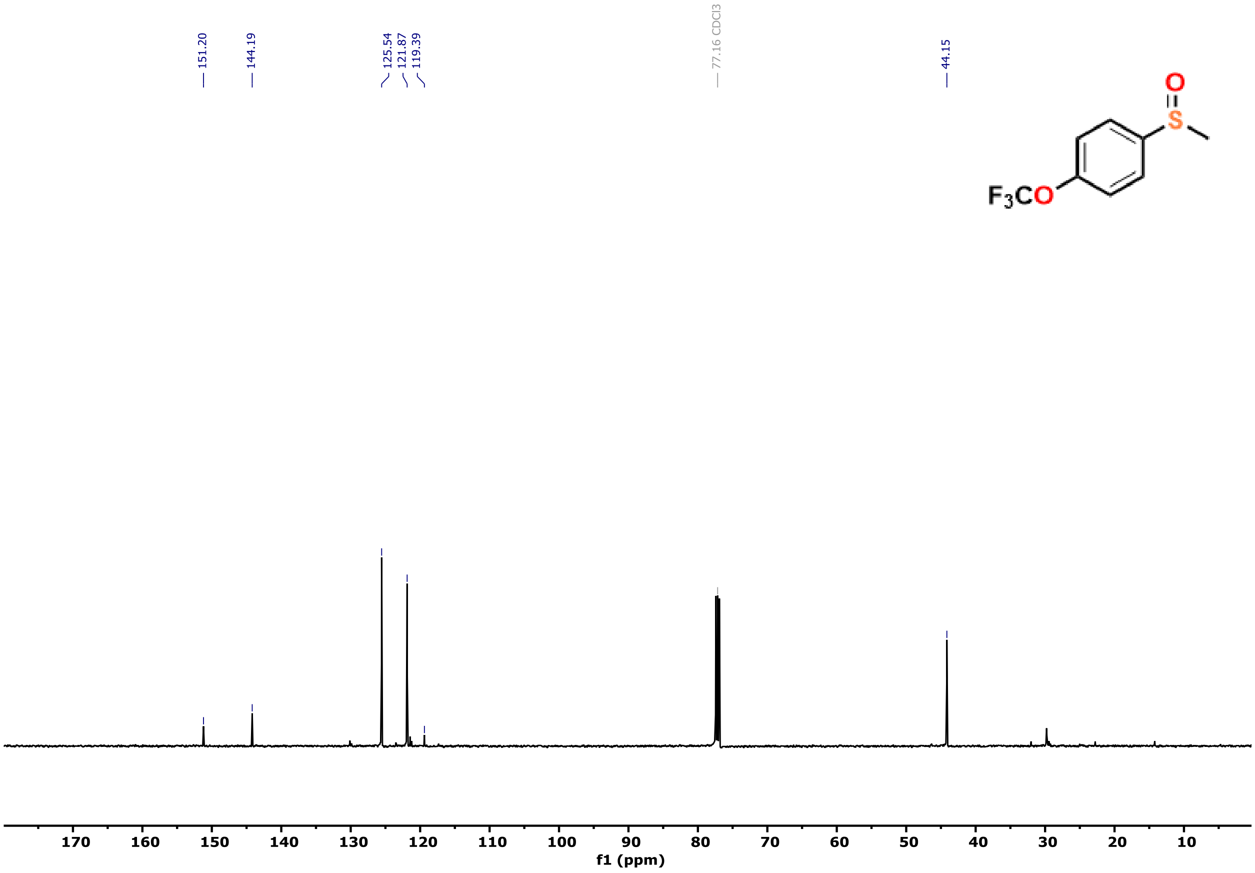

^{19}F NMR (471 MHz, $CDCl_3$)-Benzene,1-(methylsulfinyl)-4-(trifluoromethoxy) (14b)

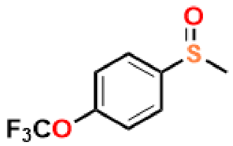

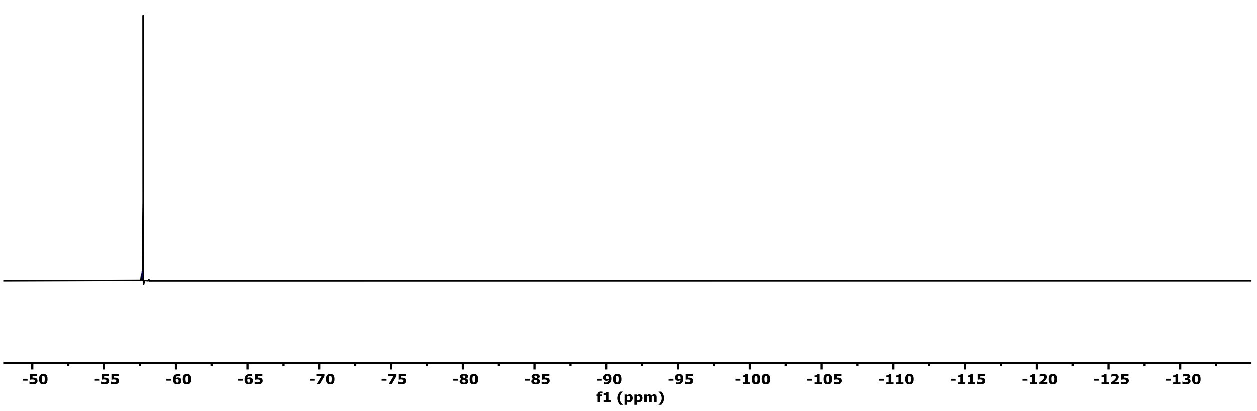

15.^{1}H NMR (500 MHz, $CDCl_3$)- N-[4-(methylthiosulfoxide)phenyl]pivalamide (15b)

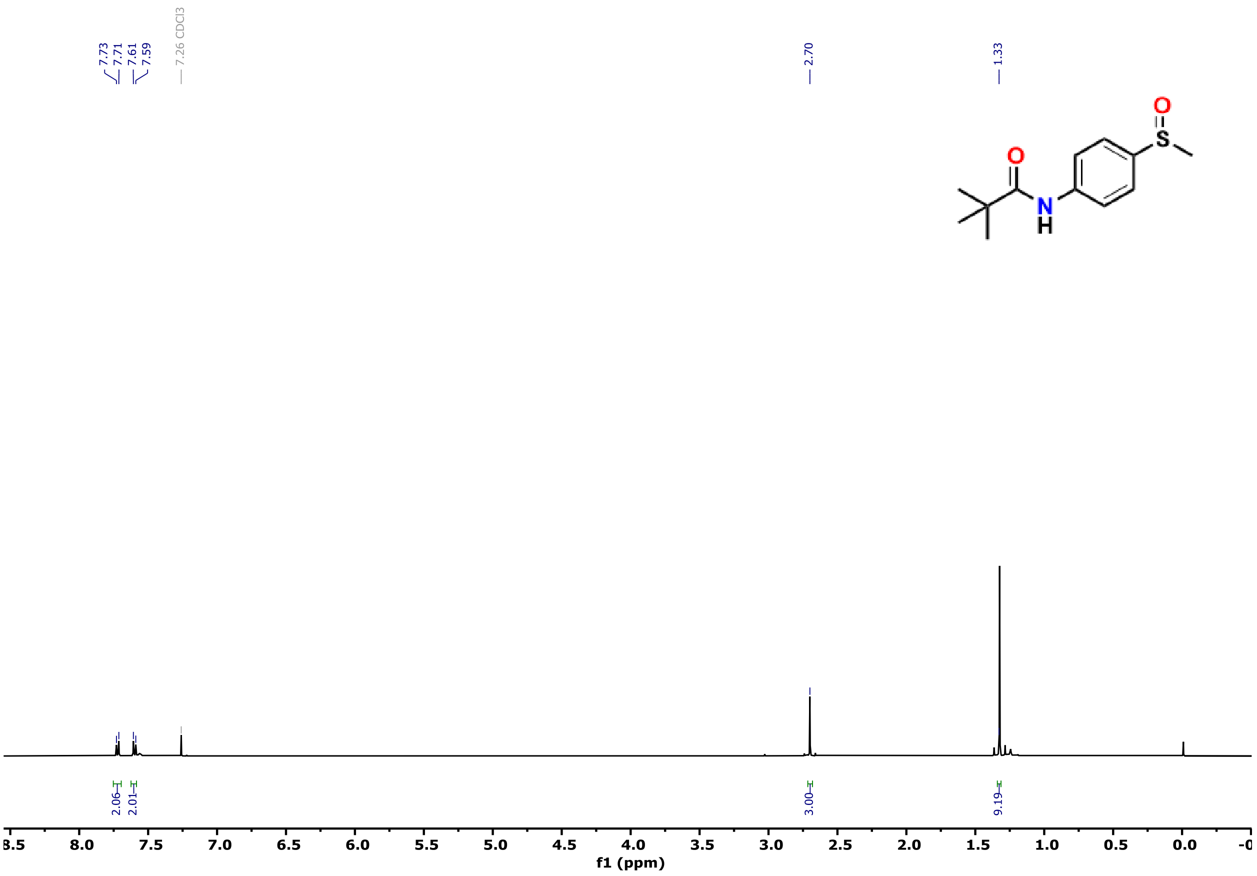

^{13}C NMR (126 MHz, $CDCl_3$)- N-[4-(methylthiosulfoxide)phenyl]pivalamide (15b)

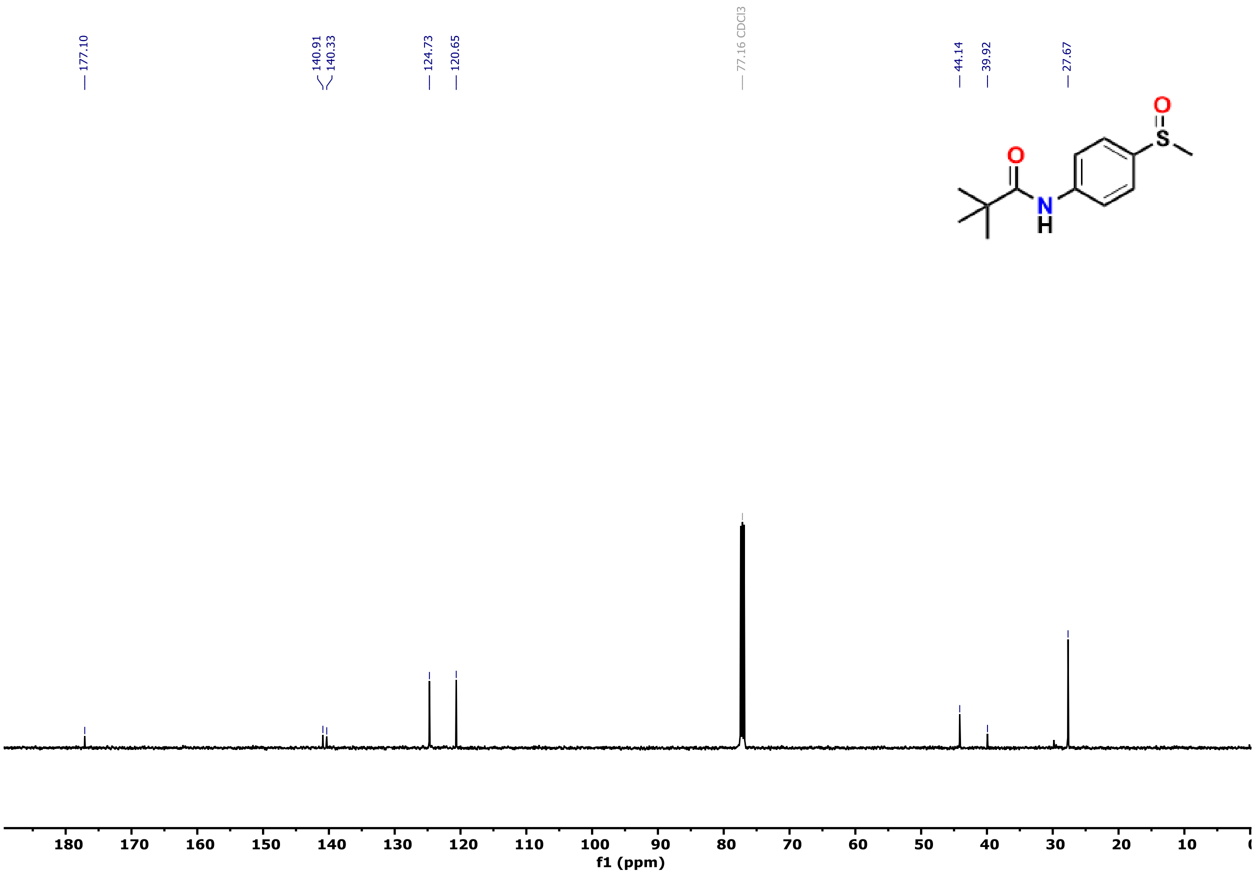

16. ^{1}H NMR (500 MHz, $CDCl_3$)- 4-(methylsulfinyl)benzoate (16b)

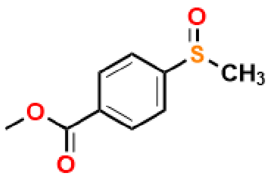

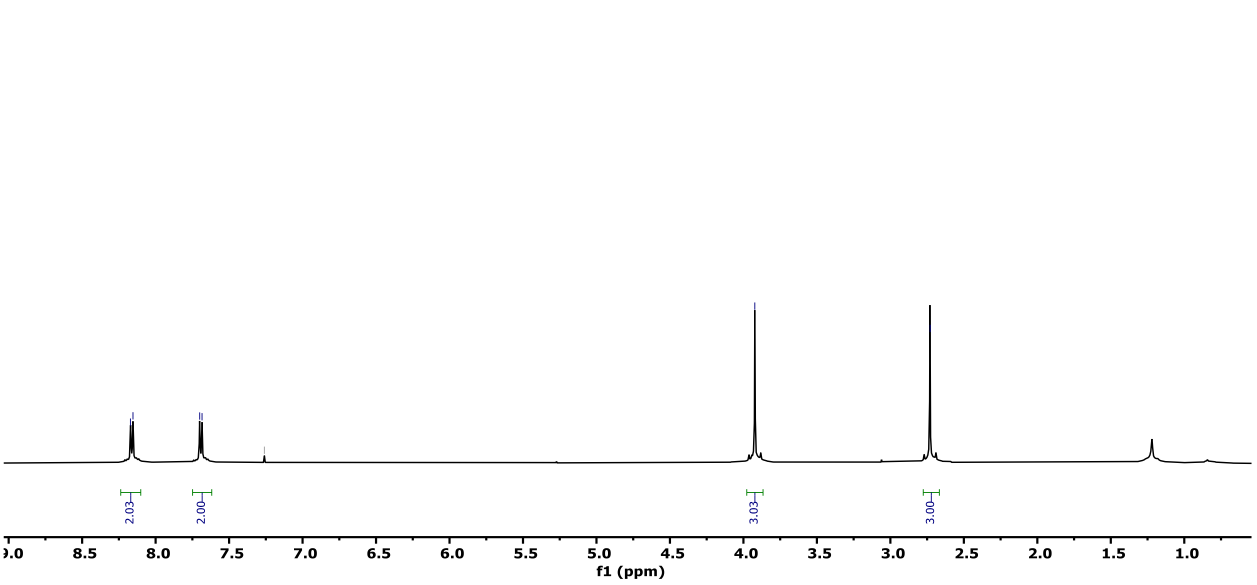

^{13}C NMR (126 MHz, $CDCl_3$)- 4-(methylsulfinyl)benzoate (16b)

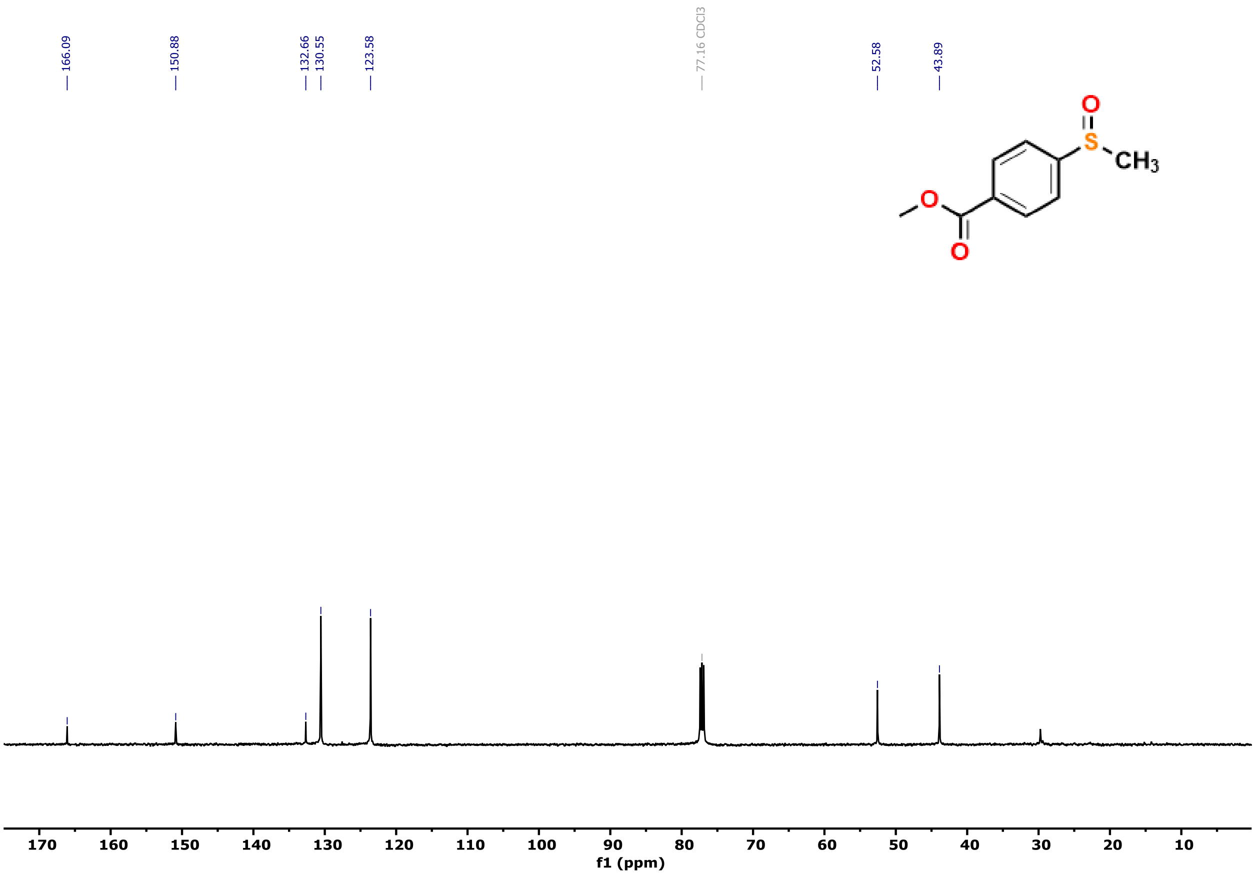

17. ^{1}H NMR (500 MHz, $CDCl_3$)- 4-(methylsulfinyl)benzaldehyde (17b)

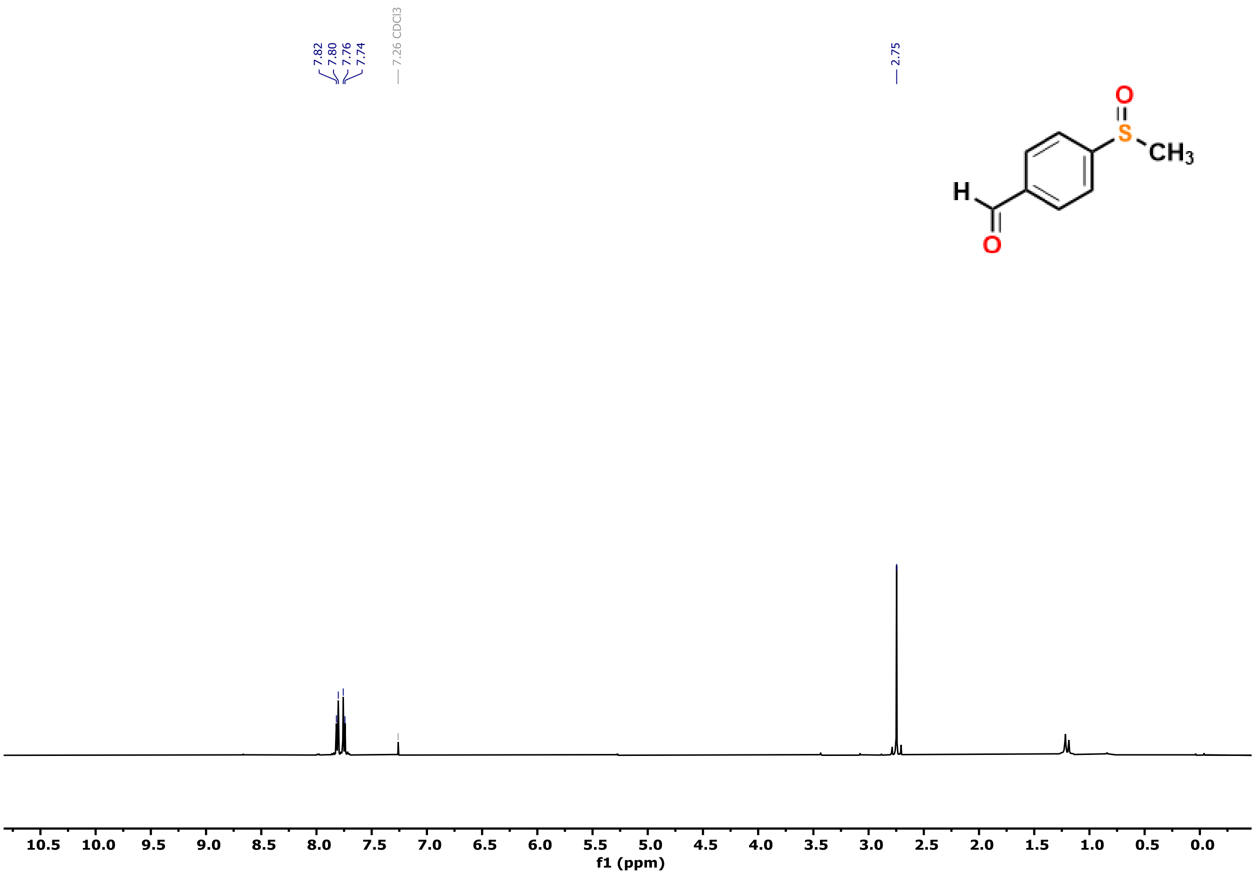

^{13}C NMR (126 MHz, $CDCl_3$)- 4-(methylsulfinyl)benzaldehyde (17b)

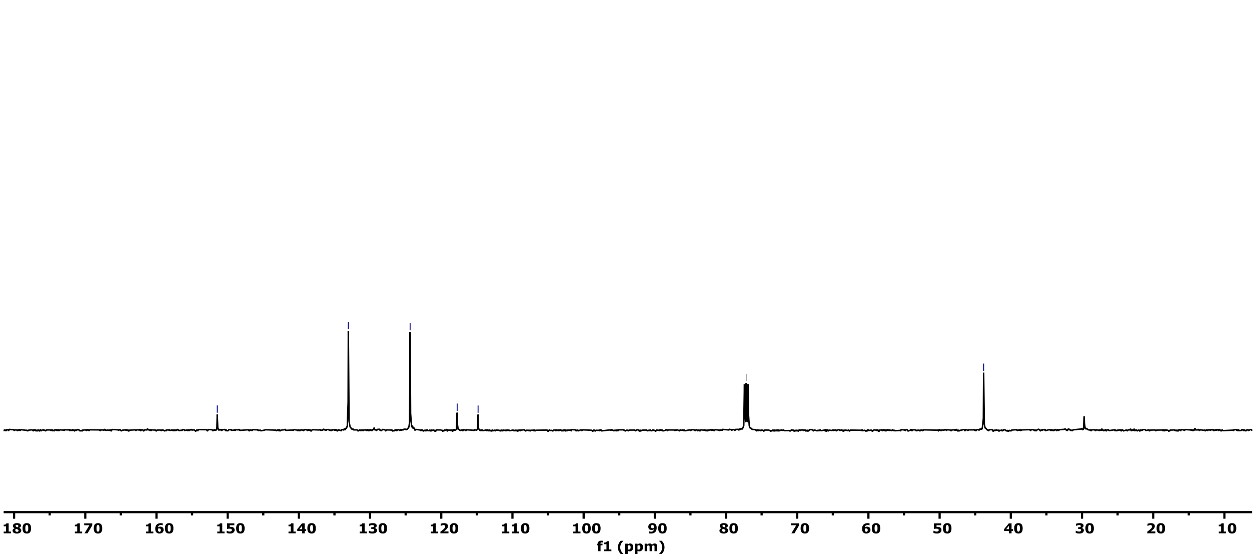

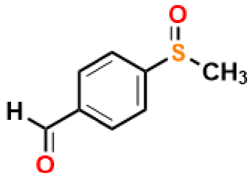

18) ^{1}H NMR (500 MHz, $CDCl_3$)- 4,4,5,5-tetramethyl-2-(4-(methylsulfinyl)phenyl)-1,3,2-dioxaborolane (18b)

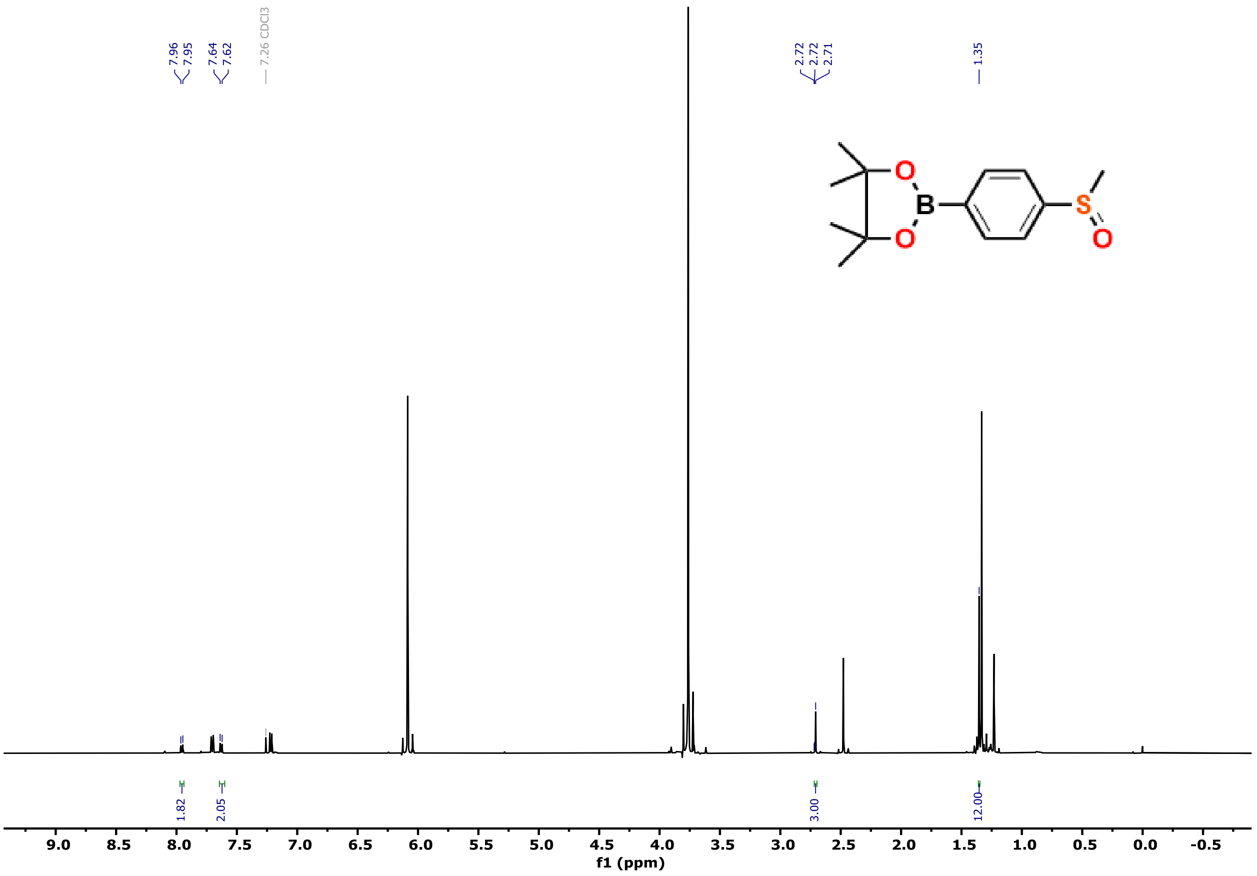

^{13}C NMR (126 MHz, $CDCl_3$)- 4,4,5,5-tetramethyl-2-(4-(methylsulfinyl)phenyl)-1,3,2-dioxaborolane (18b)

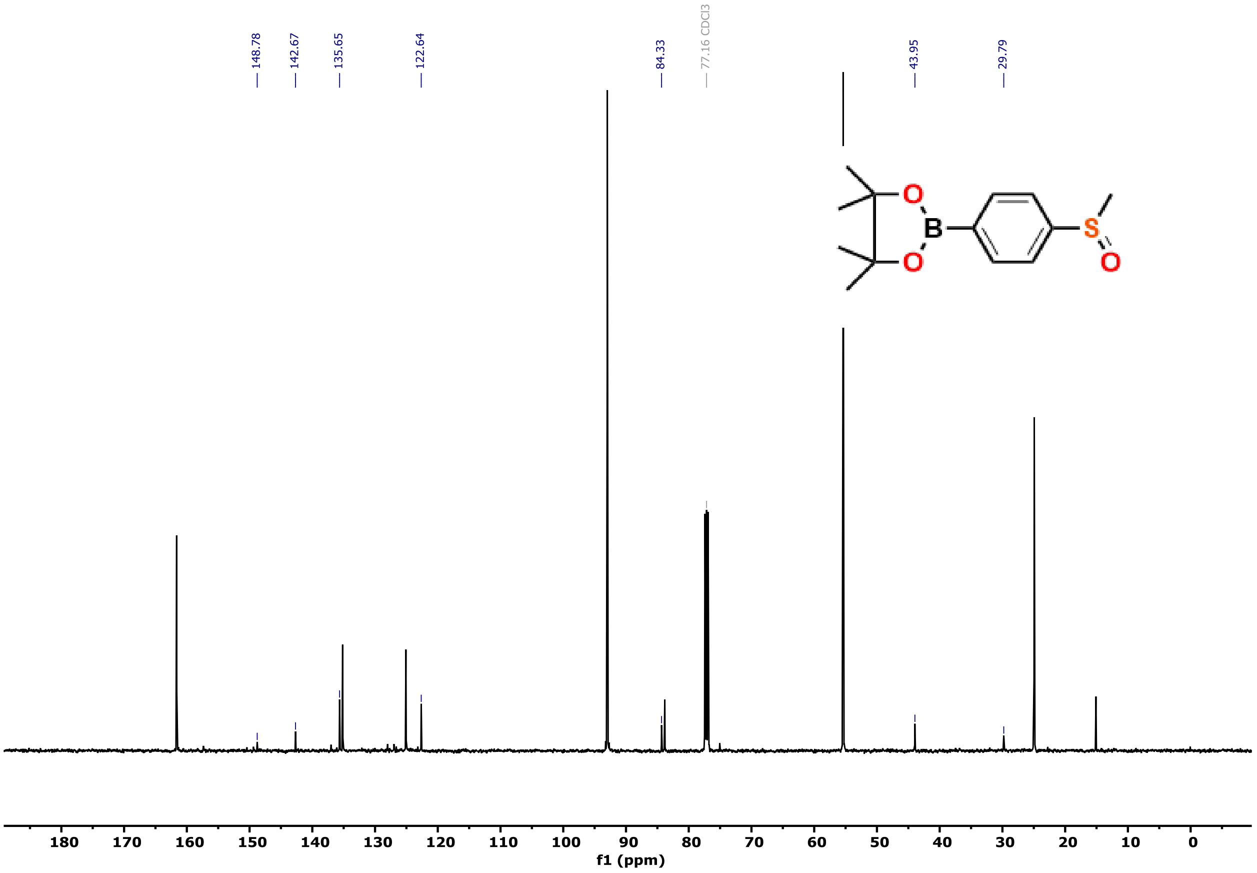

19. ^{1}H NMR (500 MHz, $CDCl_3$)- Benzyl methyl sulfoxide (19b)

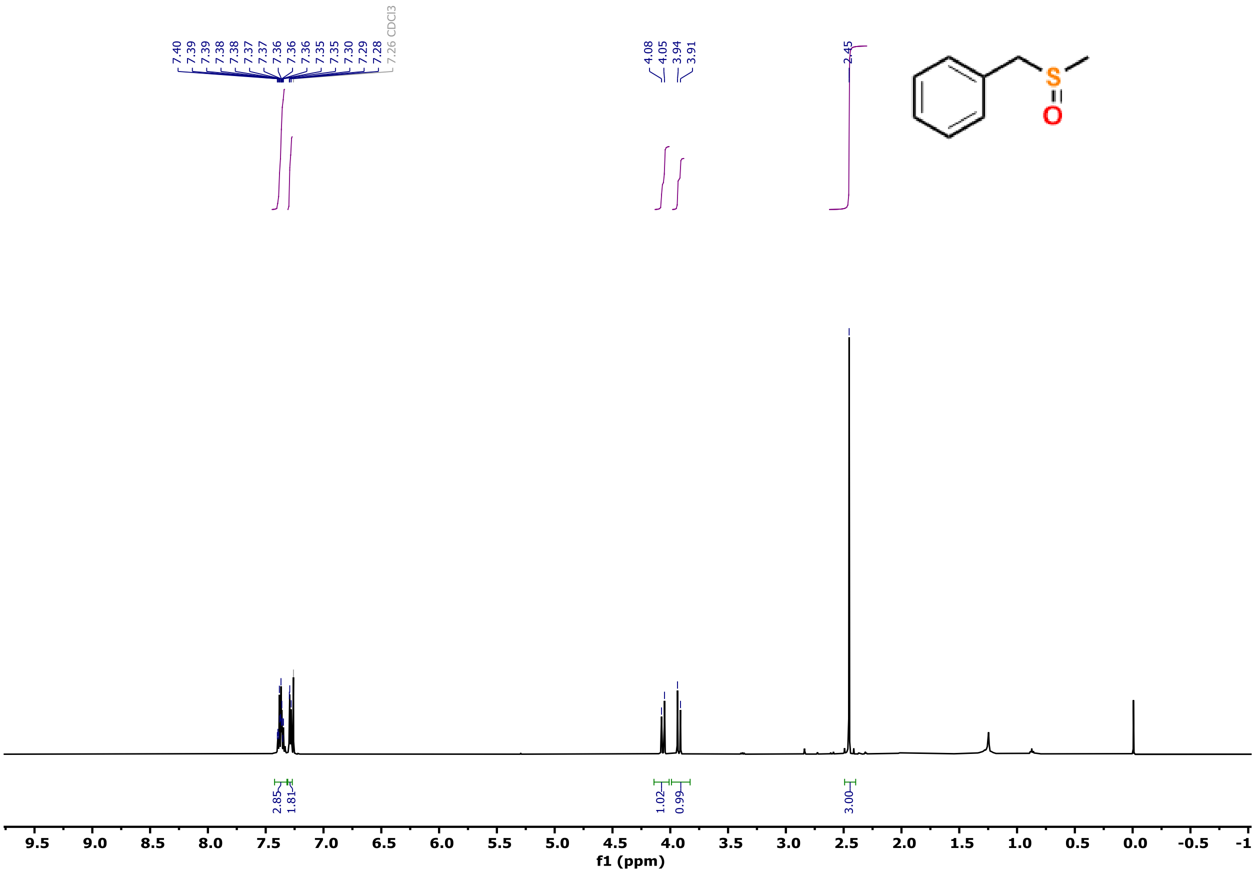

^{13}C NMR (126 MHz, $CDCl_3$)- Benzyl methyl sulfoxide (19b)

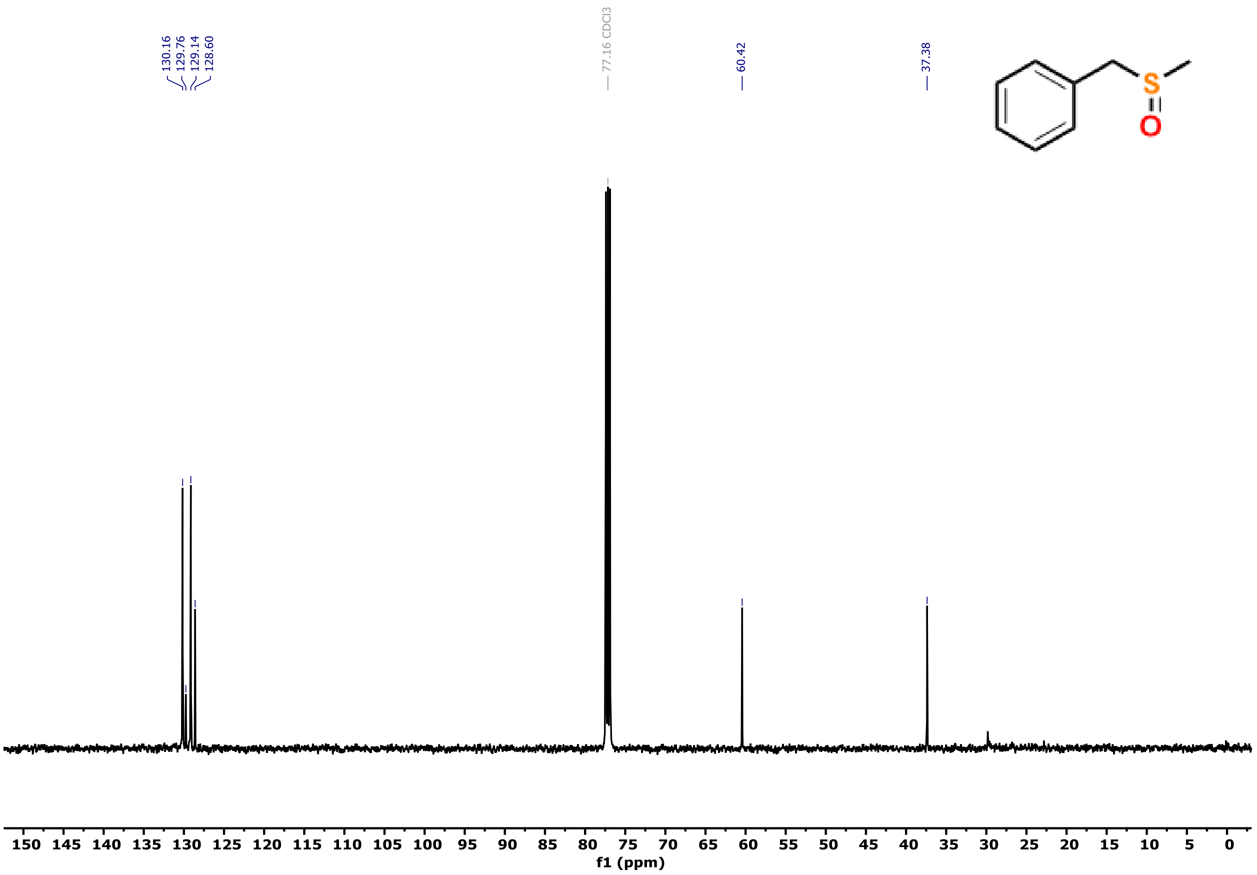

20. ^{1}H NMR (500 MHz, $CDCl_3$)- Diphenyl sulfoxide (20b)

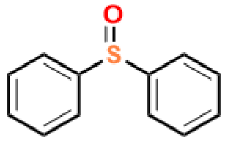

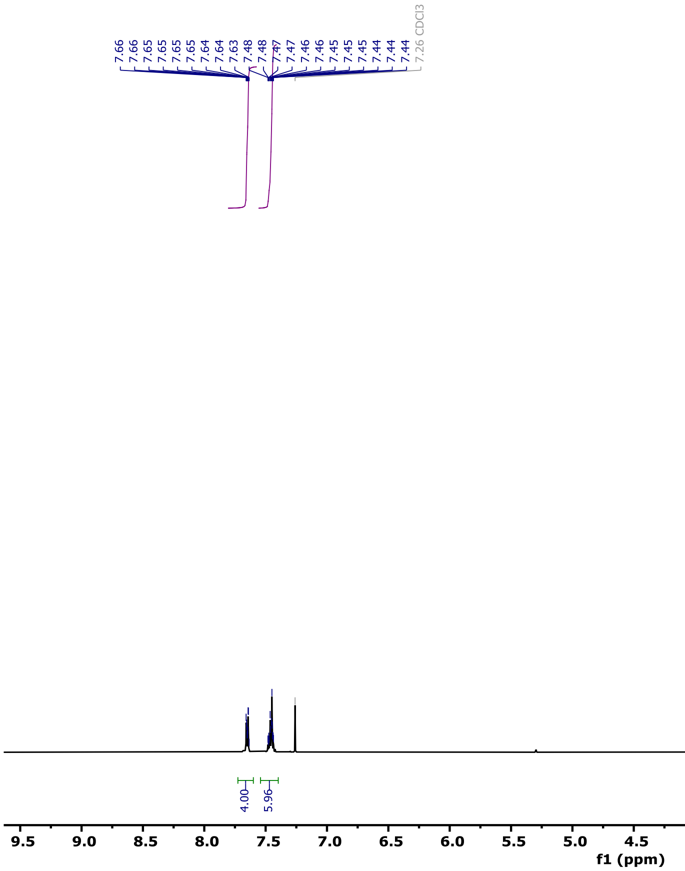

^{13}C NMR (126 MHz, $CDCl_3$)- Diphenyl sulfoxide (20b)

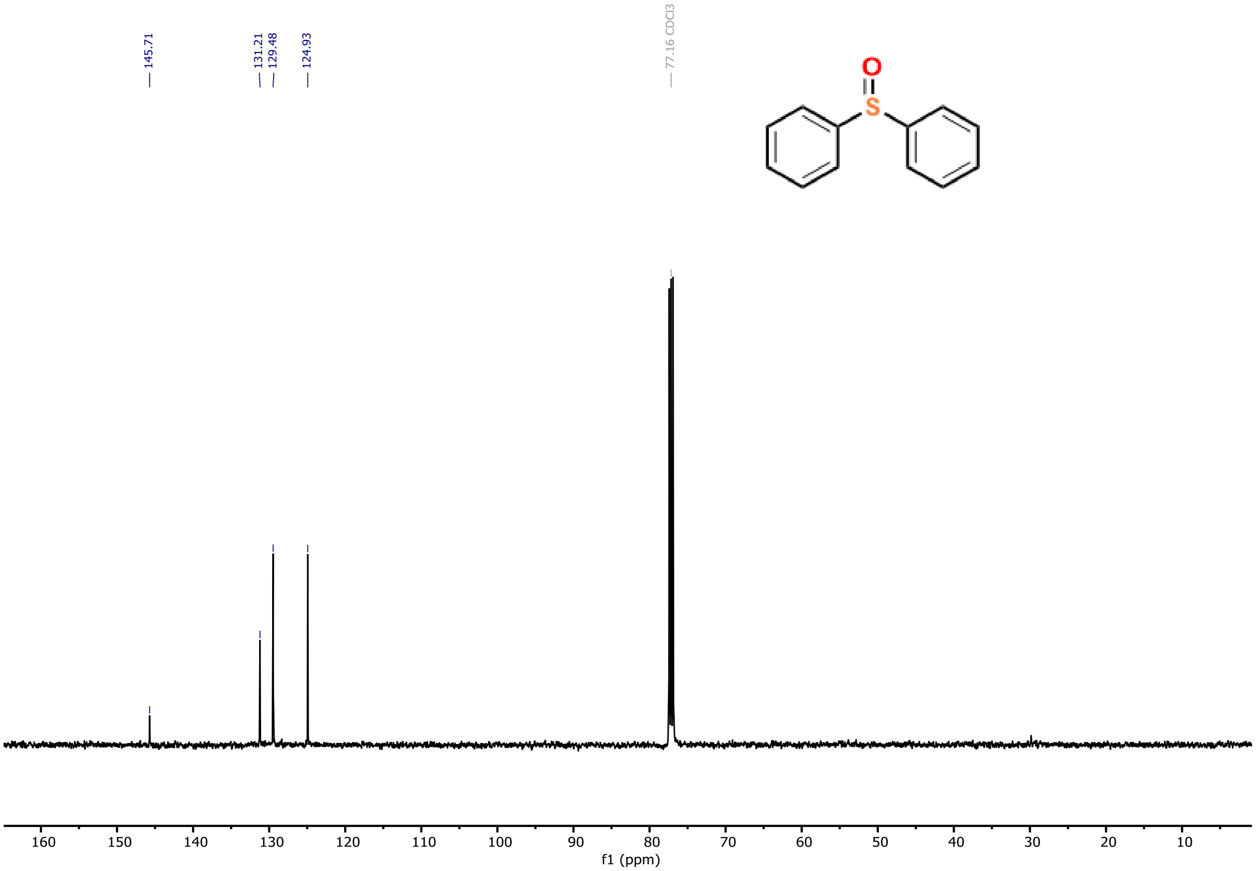

21. ¹H NMR (500 MHz, $CDCl_3$)- (methylsulfinyl)cyclopropane (21b)

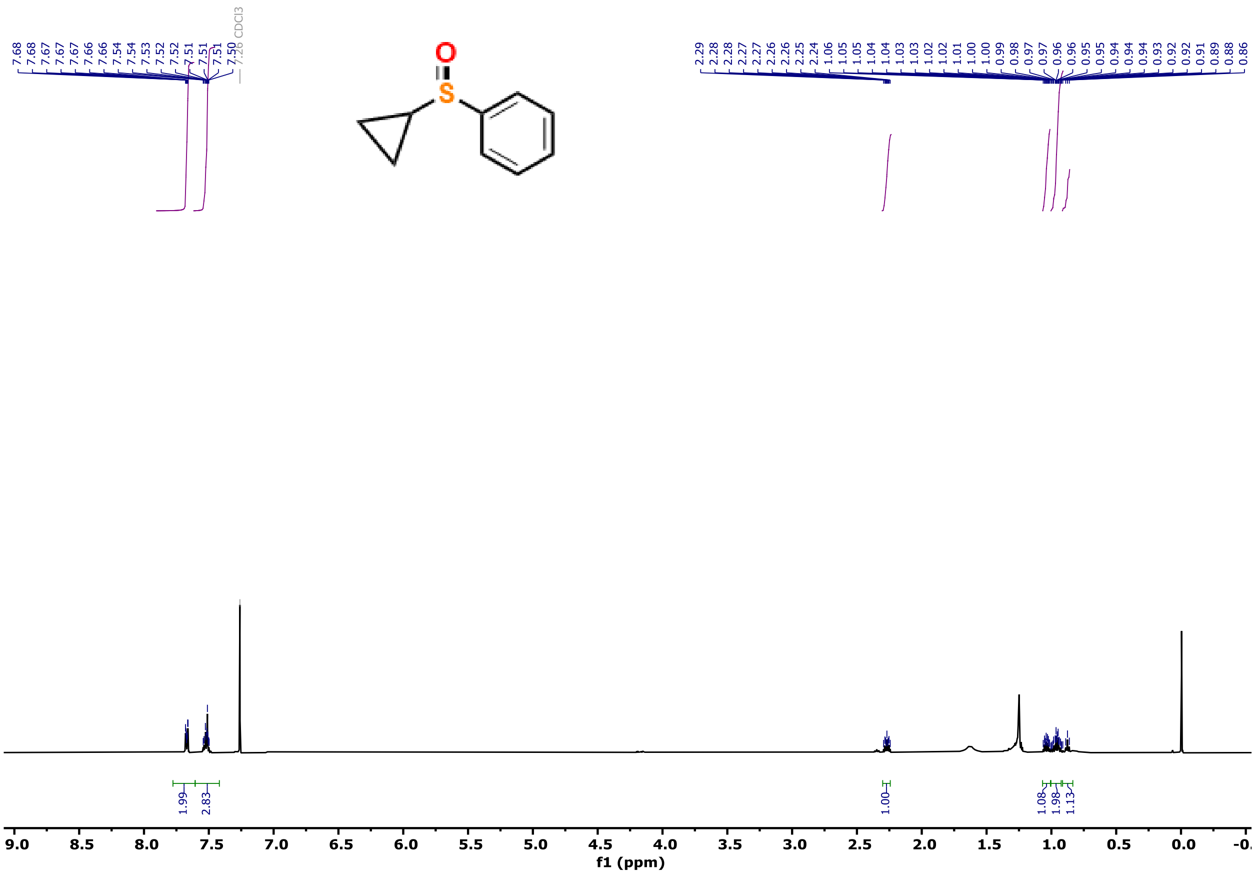

^{13}C NMR (126 MHz, $CDCl_3$)- (methylsulfinyl)cyclopropane (21b)

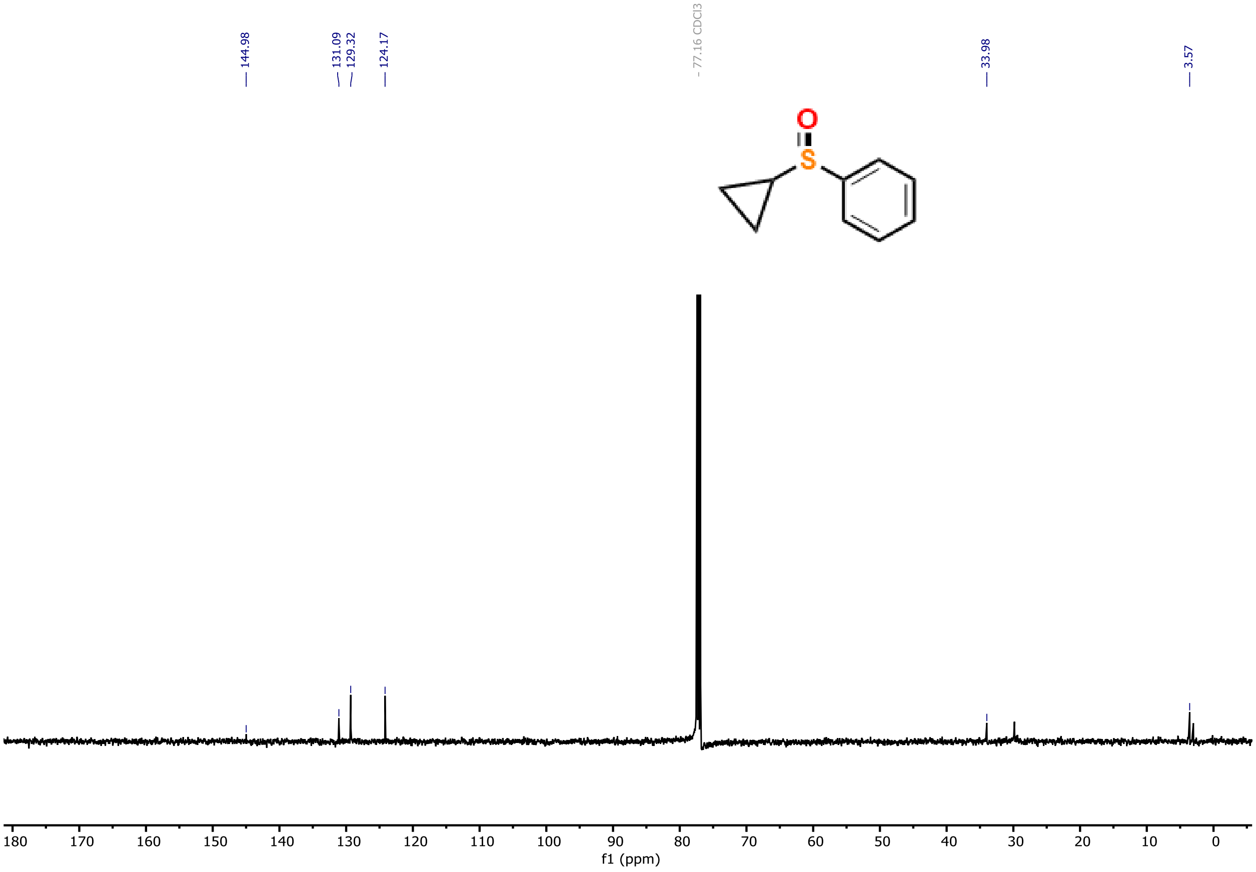

22. 1H NMR (500 MHz, CDCl3)- Ethylphenyl Sulfoxide (22b)

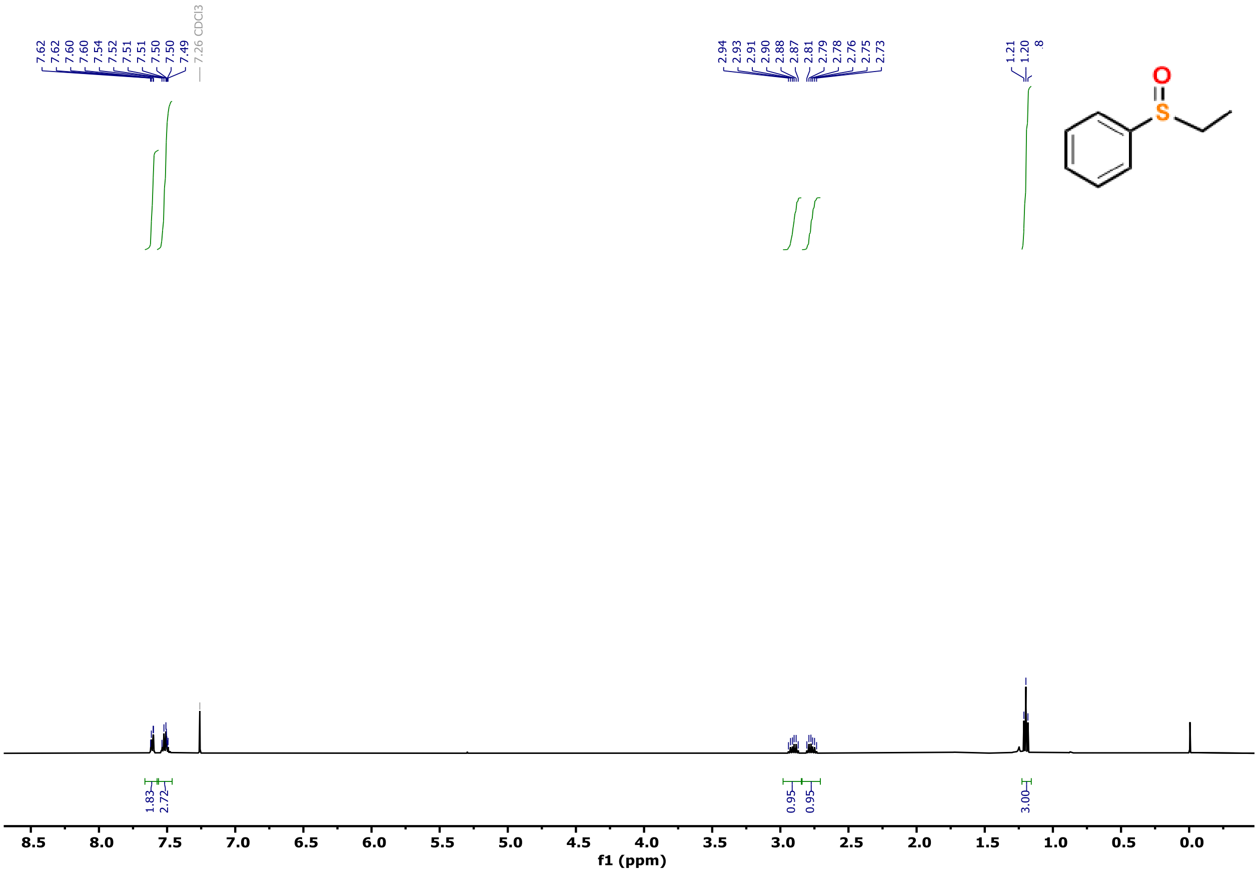

^{13}C NMR (126 MHz, $CDCl_3$)- Ethylphenyl Sulfoxide (22b)

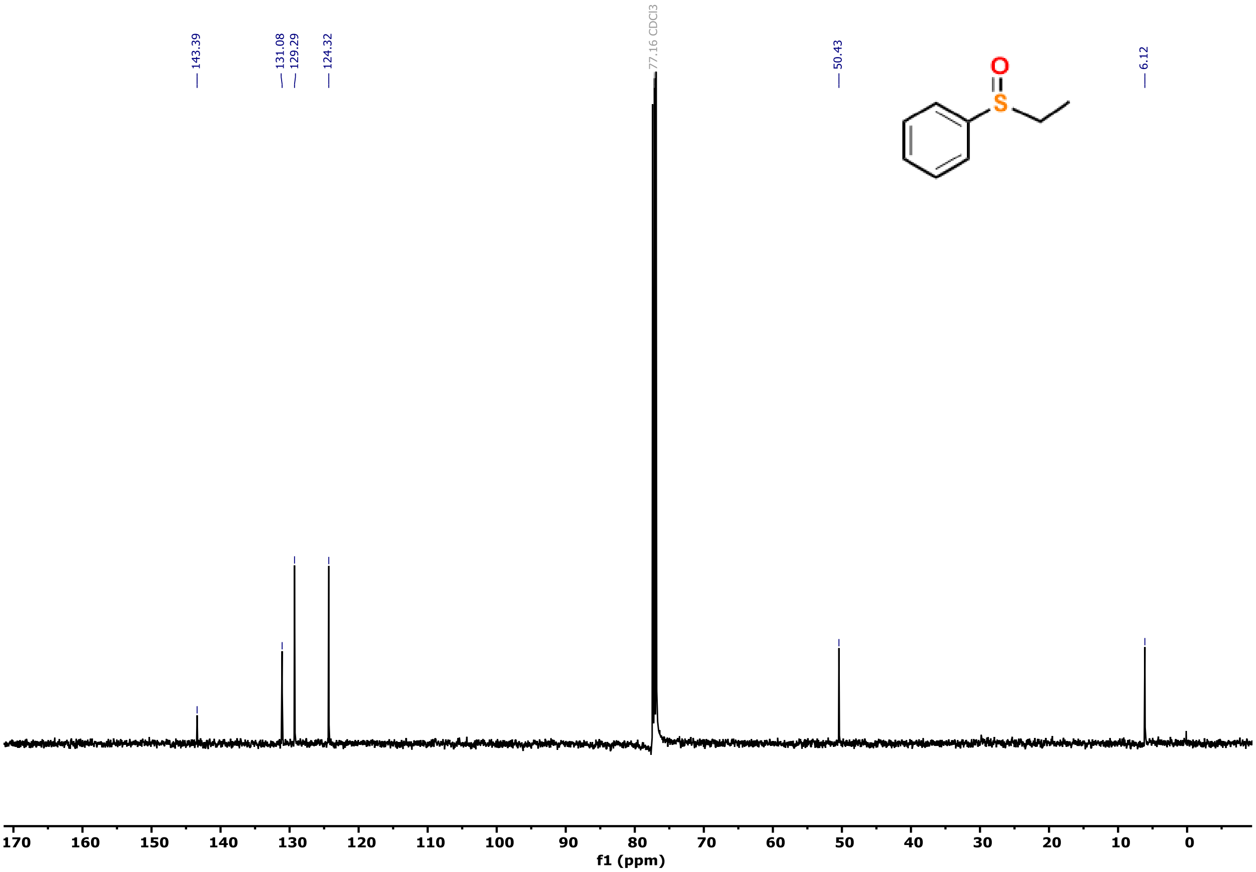

23. ^{1}H NMR (500 MHz, $CDCl_3$)- Dibenzothiophene sulfoxide (23b)

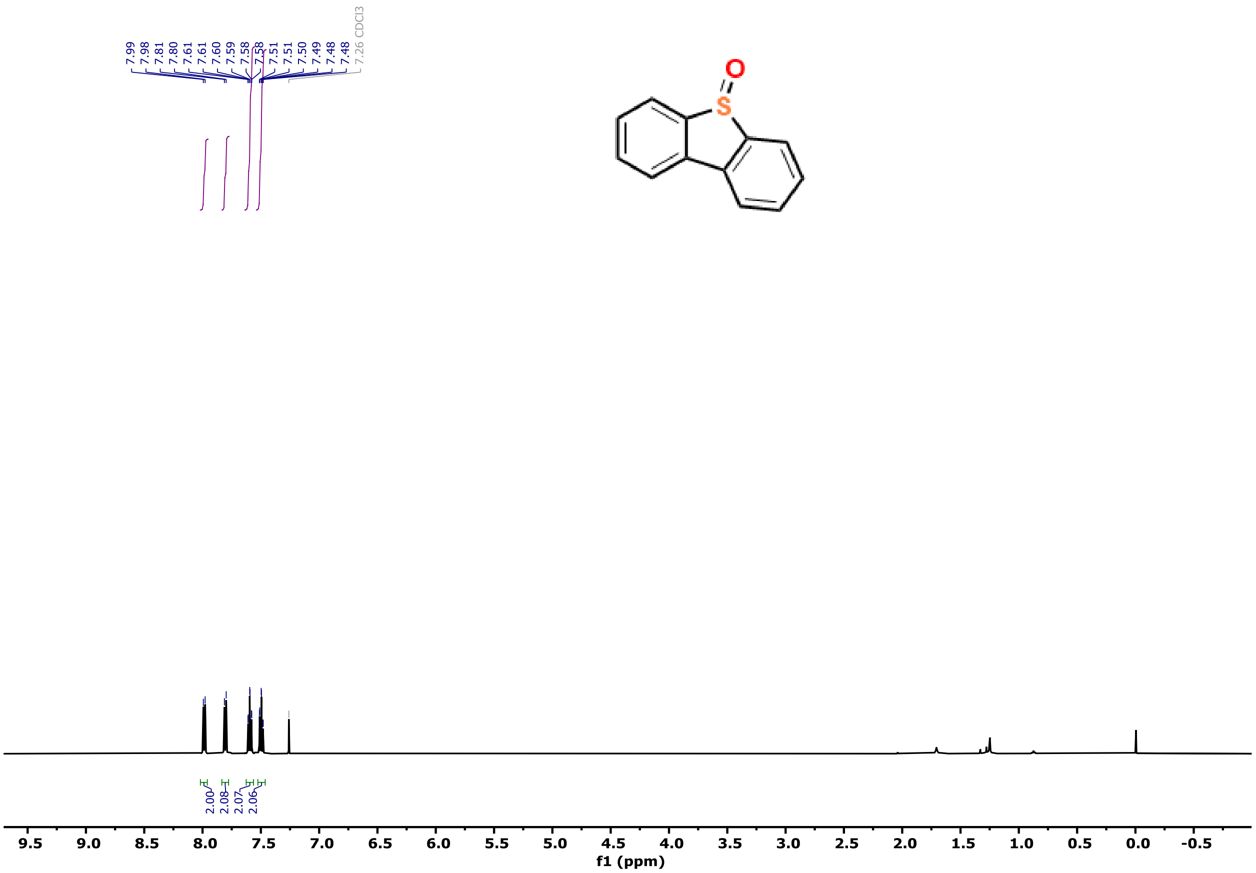

^{13}C NMR (126 MHz, $CDCl_3$)- Dibenzothiophene sulfoxide (23b)

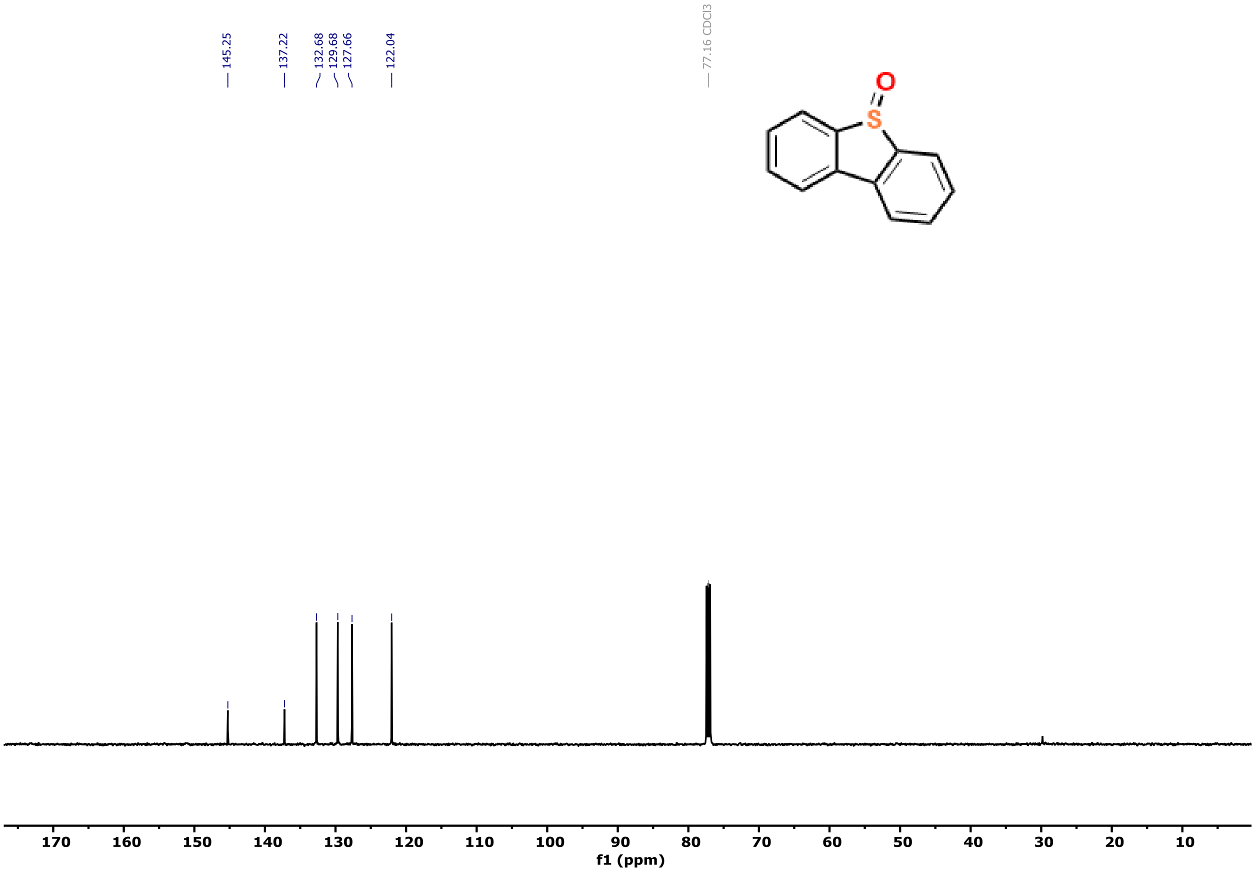

24. ^{1}H NMR (500 MHz, $CDCl_3$)- Thianaphthalene sulfoxide (24b)

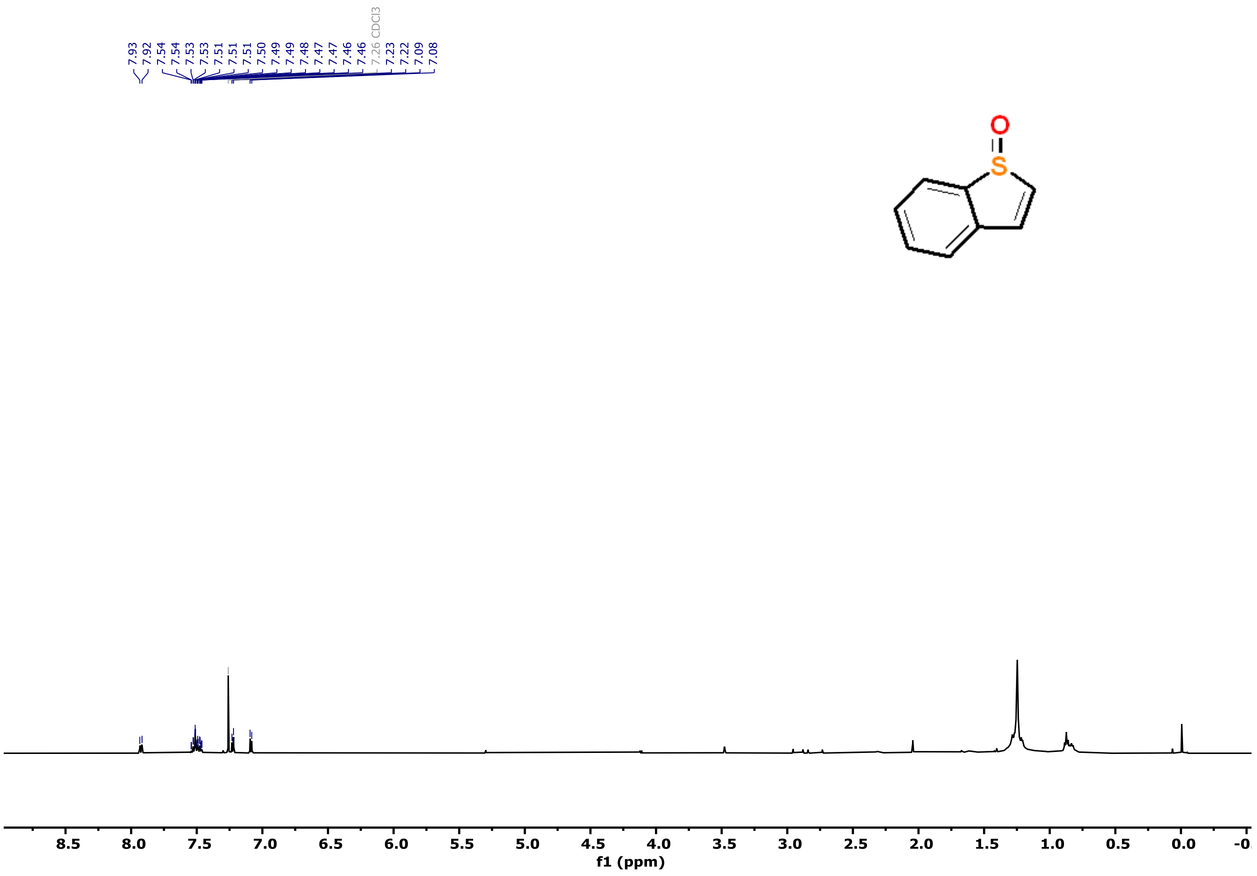

^{13}C NMR (126 MHz, $CDCl_3$)- Thianaphthalene sulfoxide (24b)

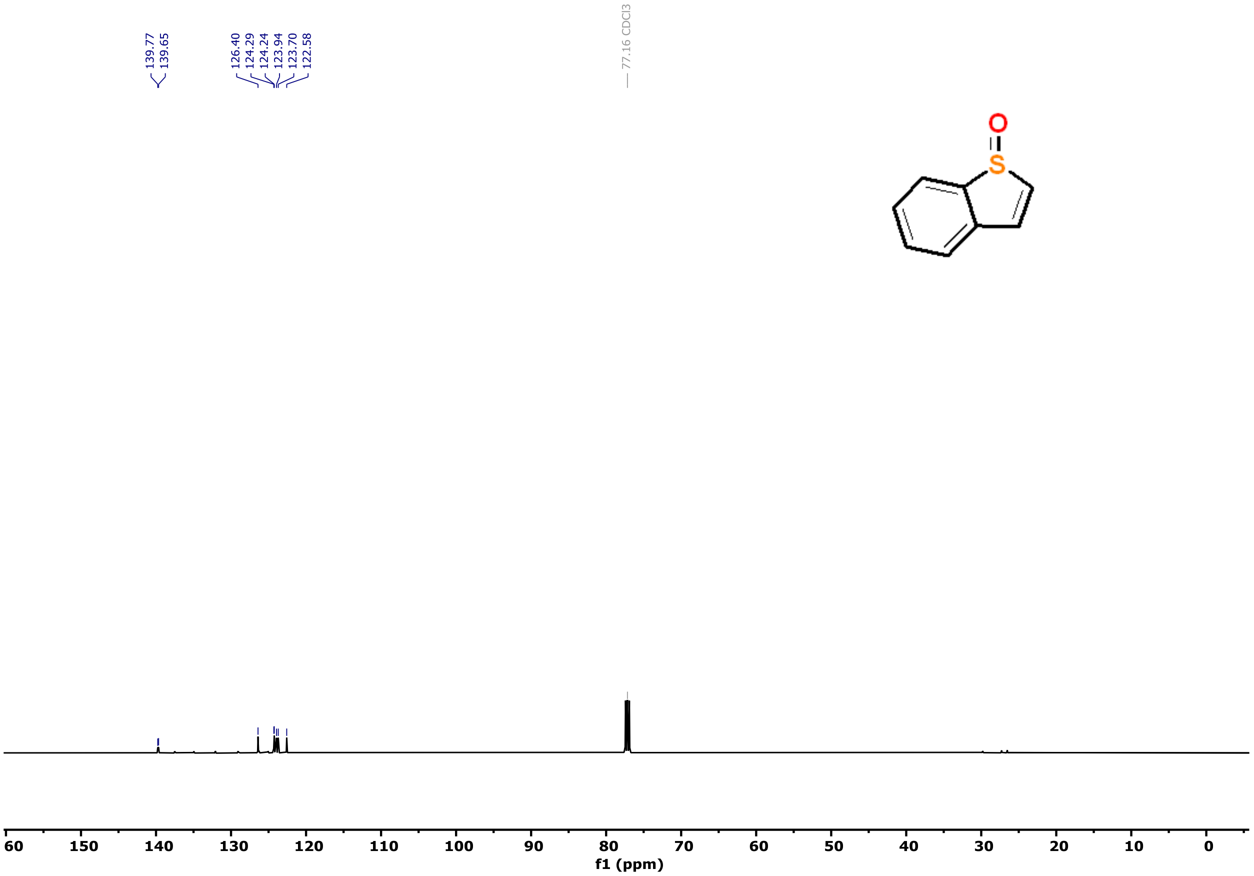

25. 1H NMR (500 MHz, CDCl3)- (4-(benzyloxy)phenyl) (methyl)sulfoxide (25b)

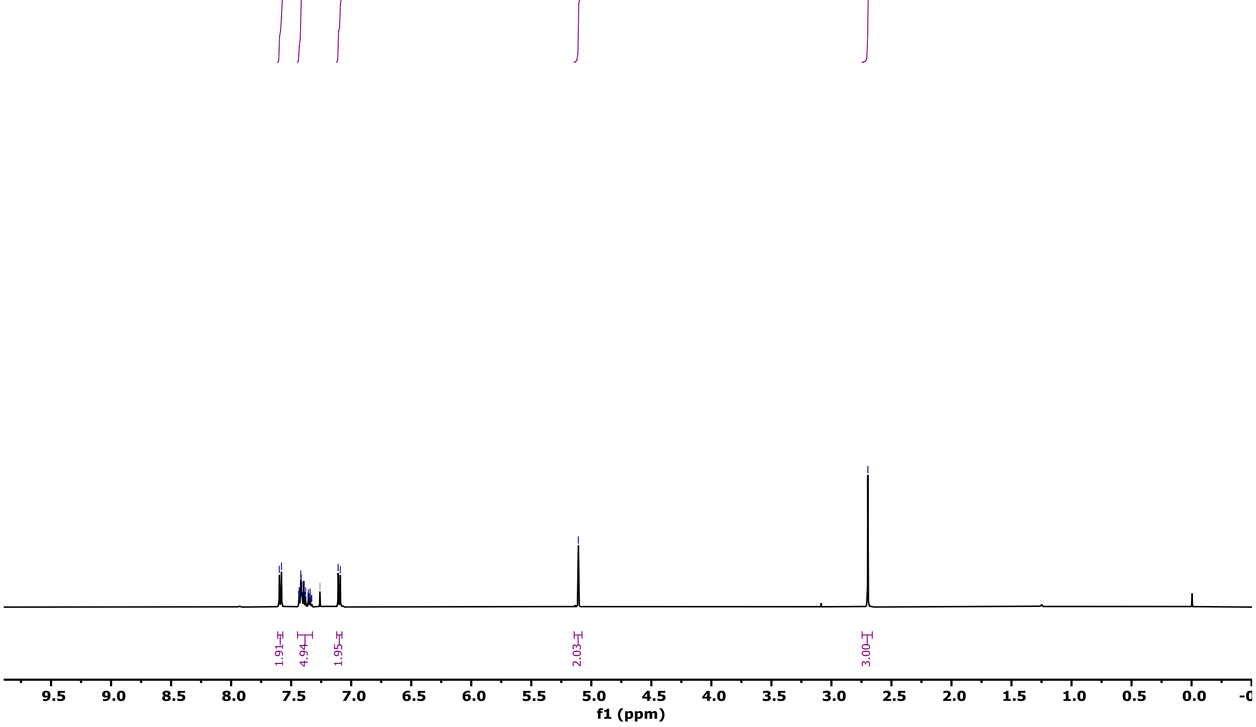

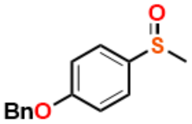

^{13}C NMR (126 MHz, $CDCl_3$)- (4-(benzyloxy)phenyl) (methyl)sulfoxide (25b)

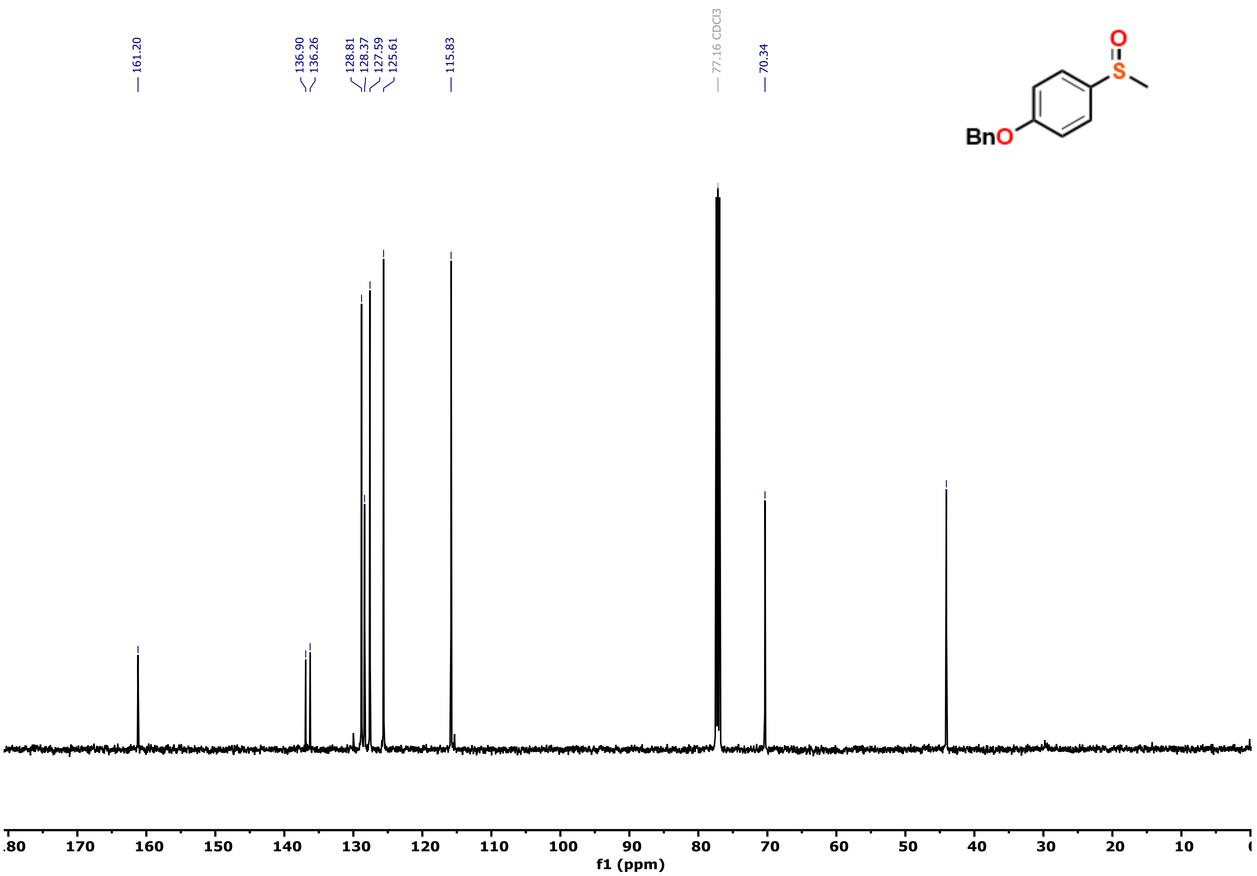

26.¹H NMR (500 MHz, $CDCl_3$)- Dibutylsulfoxide (26b)

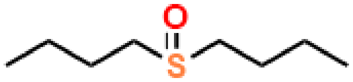

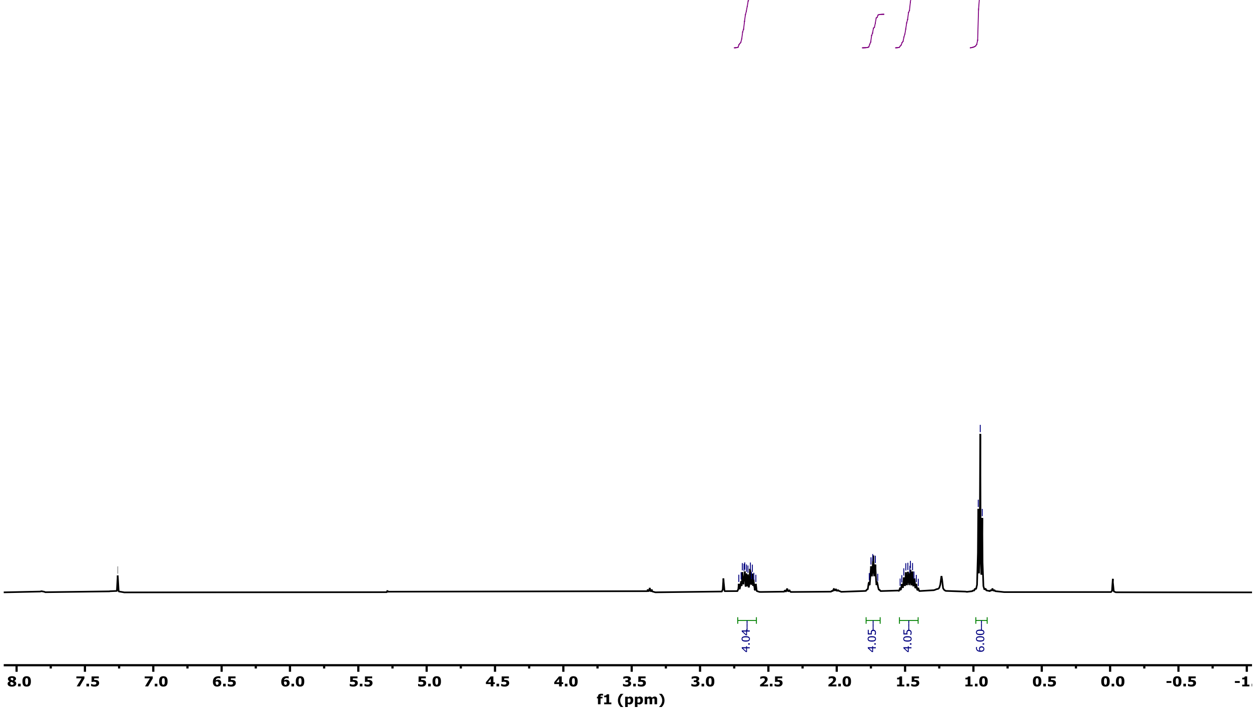

^{13}C NMR (126 MHz, $CDCl_3$)-Dibutylsulfoxide (26b)

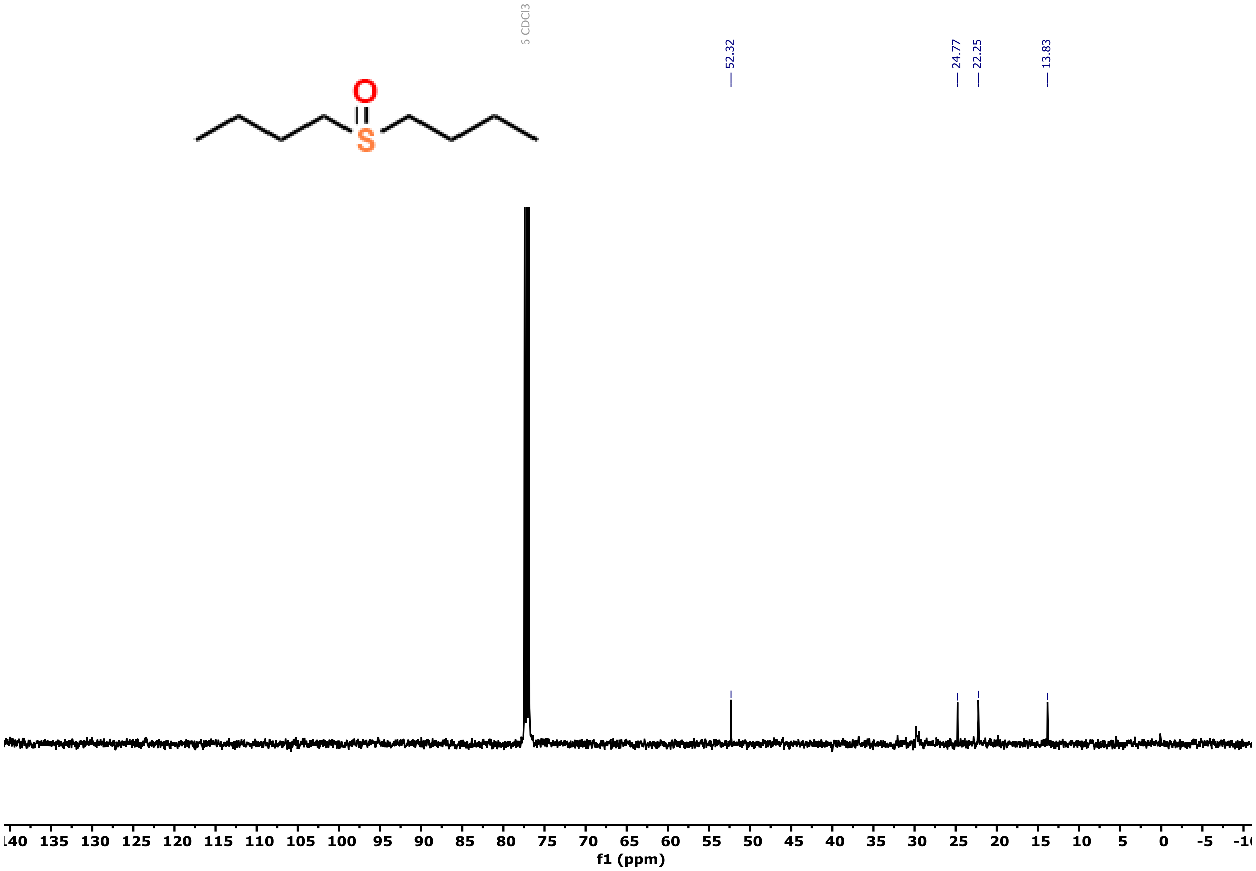

27. ^{1}H NMR (500 MHz, $CDCl_3$)-Hexylmethyl Sulfoxide (27b)

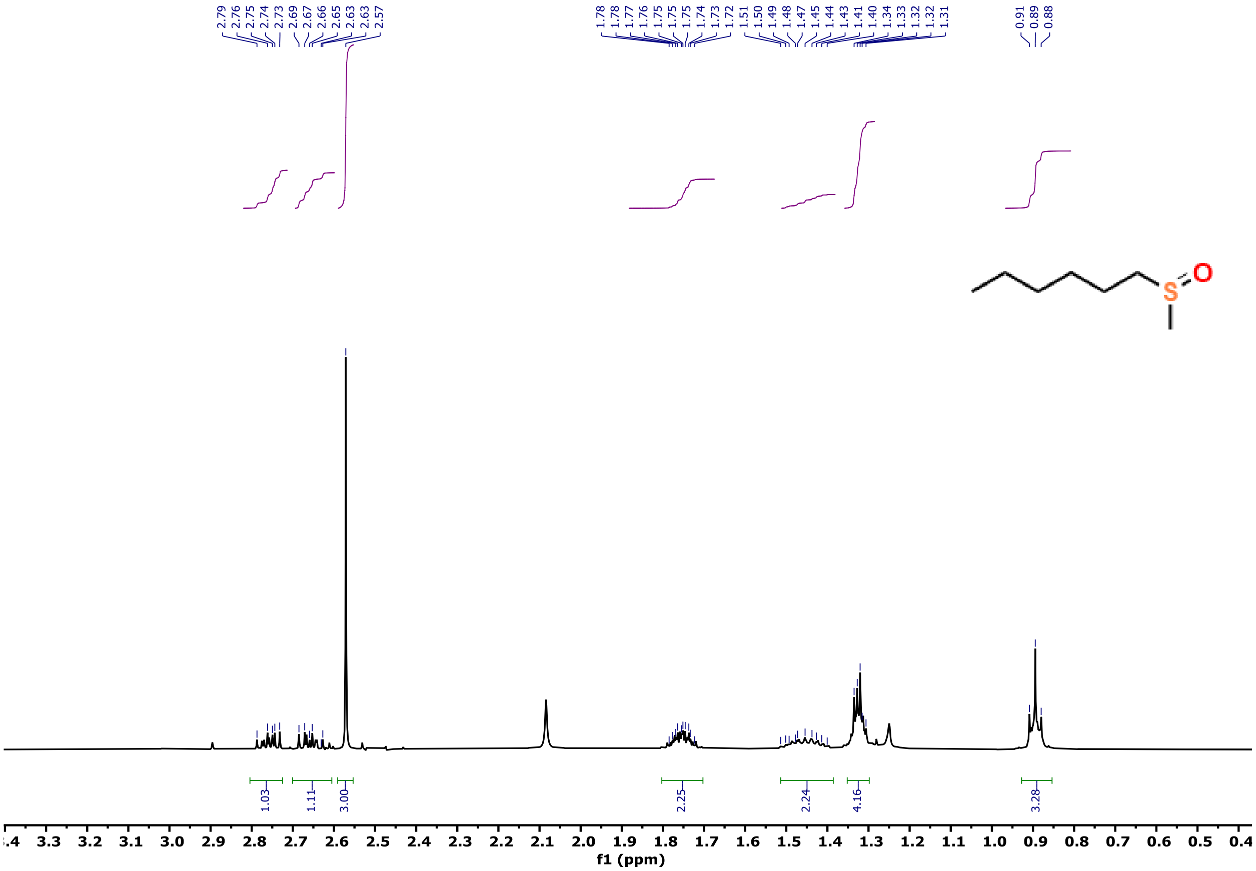

^{13}C NMR (126 MHz, $CDCl_3$)- Hexylmethyl sulfoxide (27b)

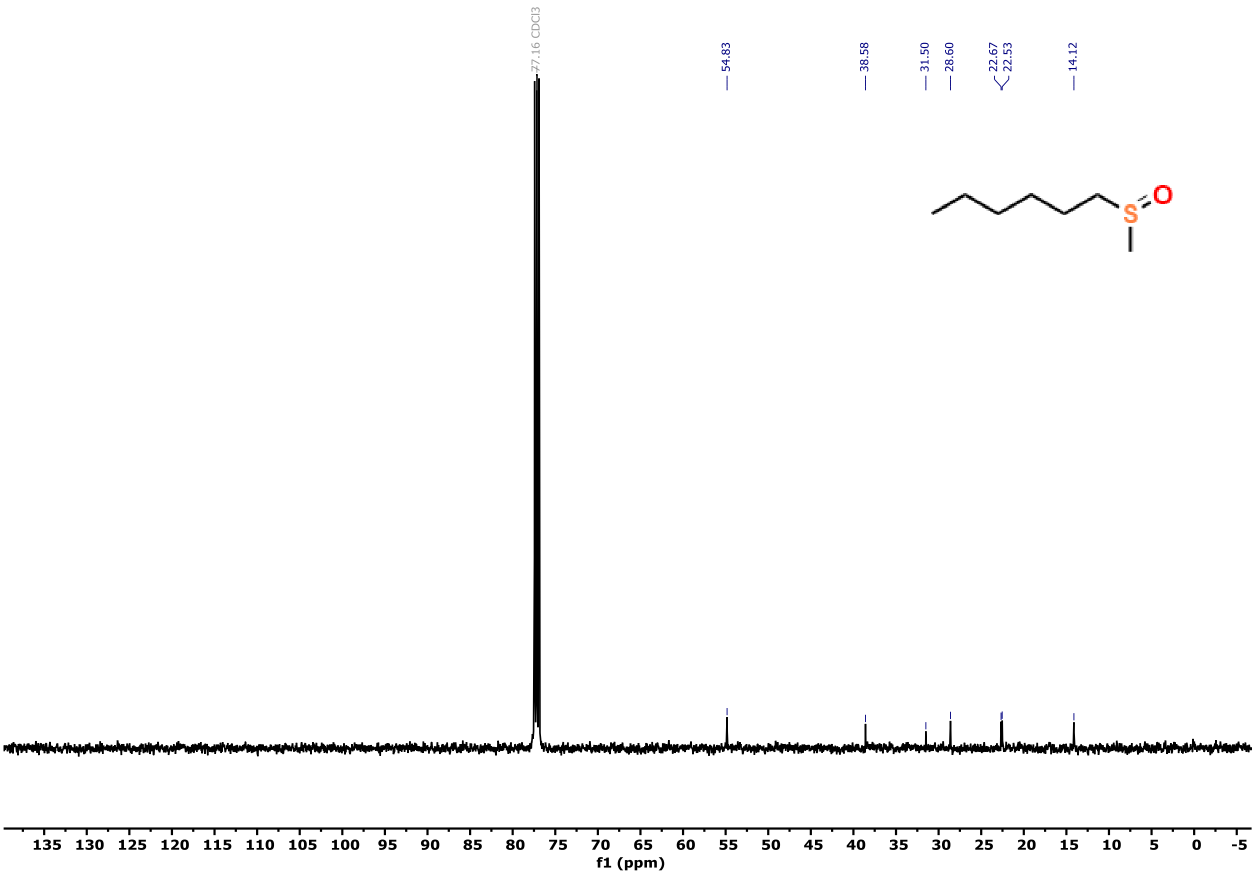

28. ^{1}H NMR (500 MHz, $CDCl_3$)- Tertbutyl methyl sulfoxide (28b)

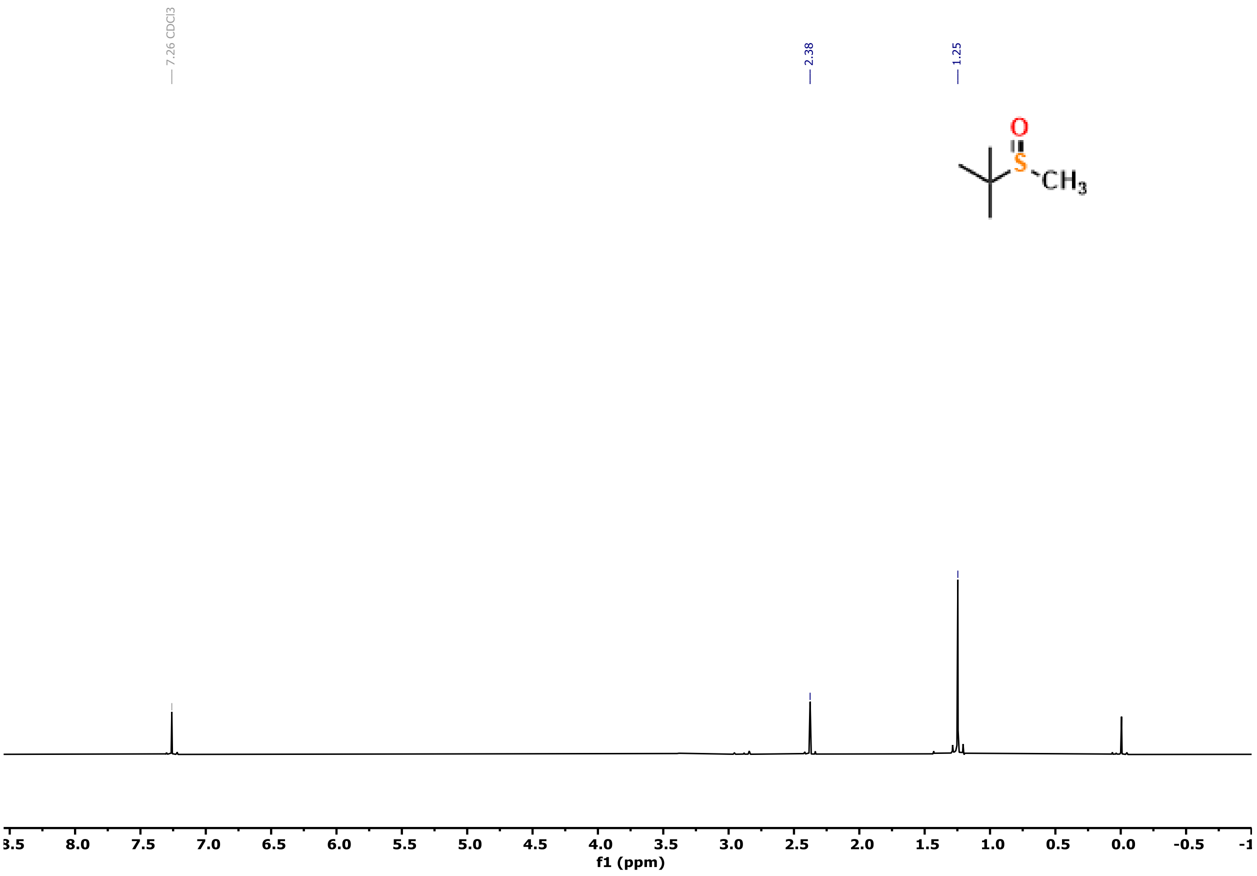

^{13}C NMR (126 MHz, $CDCl_3$)- *tert*-Butyl methyl sulfoxide (28b)

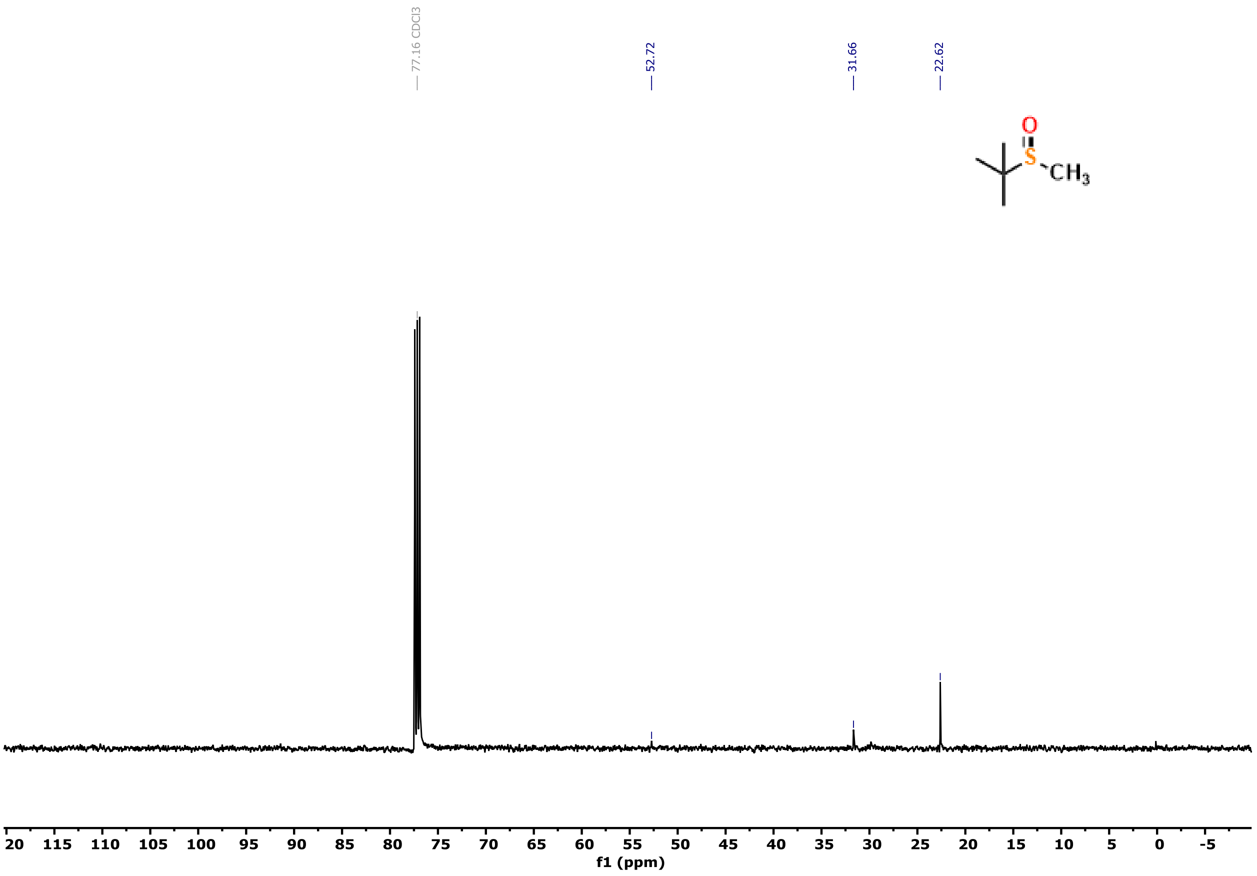

29. ^{1}H NMR (500 MHz, $CDCl_3$)- 6-(methylsulfinyl)hexanenitrile (29b)

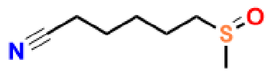

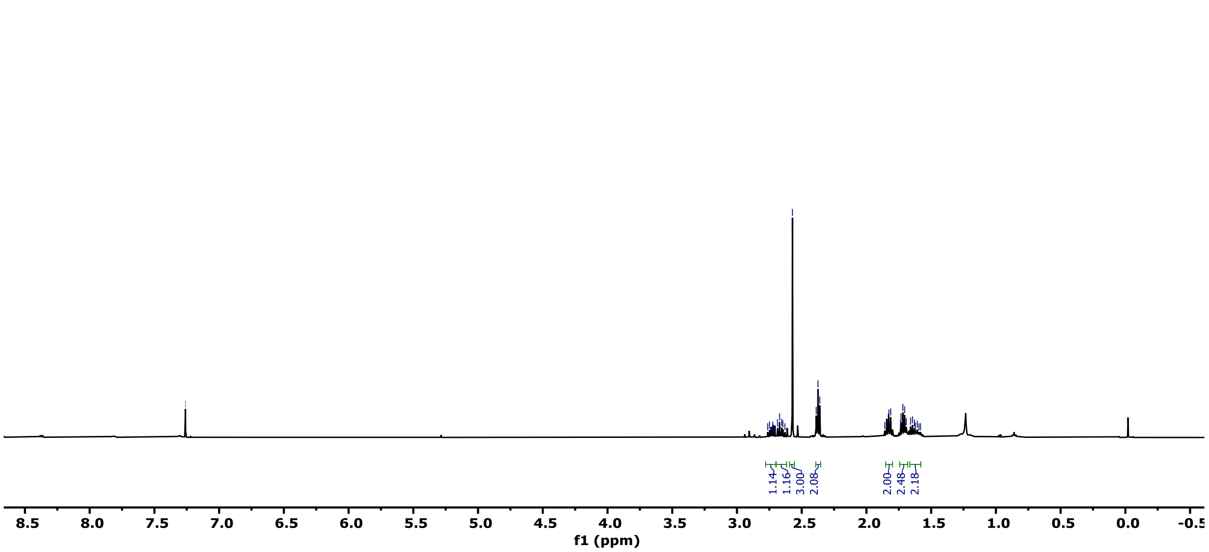

^{13}C NMR (126 MHz, $CDCl_3$)- 6-(methylsulfinyl)hexanenitrile (29b)

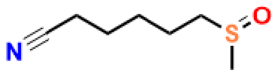

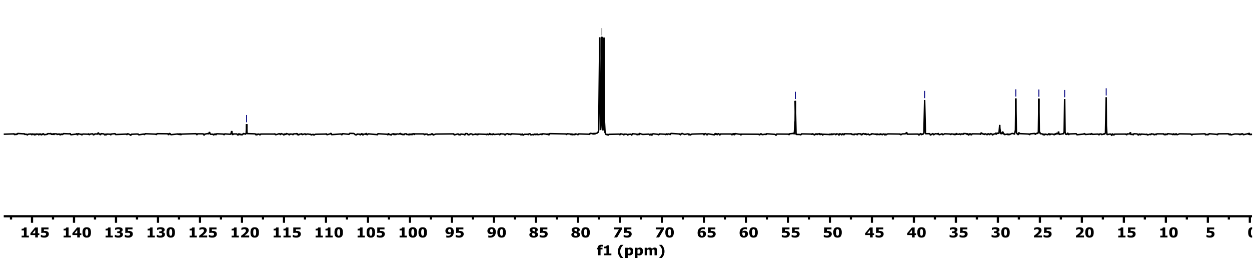

30. ^{1}H NMR (500 MHz, $CDCl_3$)- Cyclopentyl methyl sulfoxide (30b)

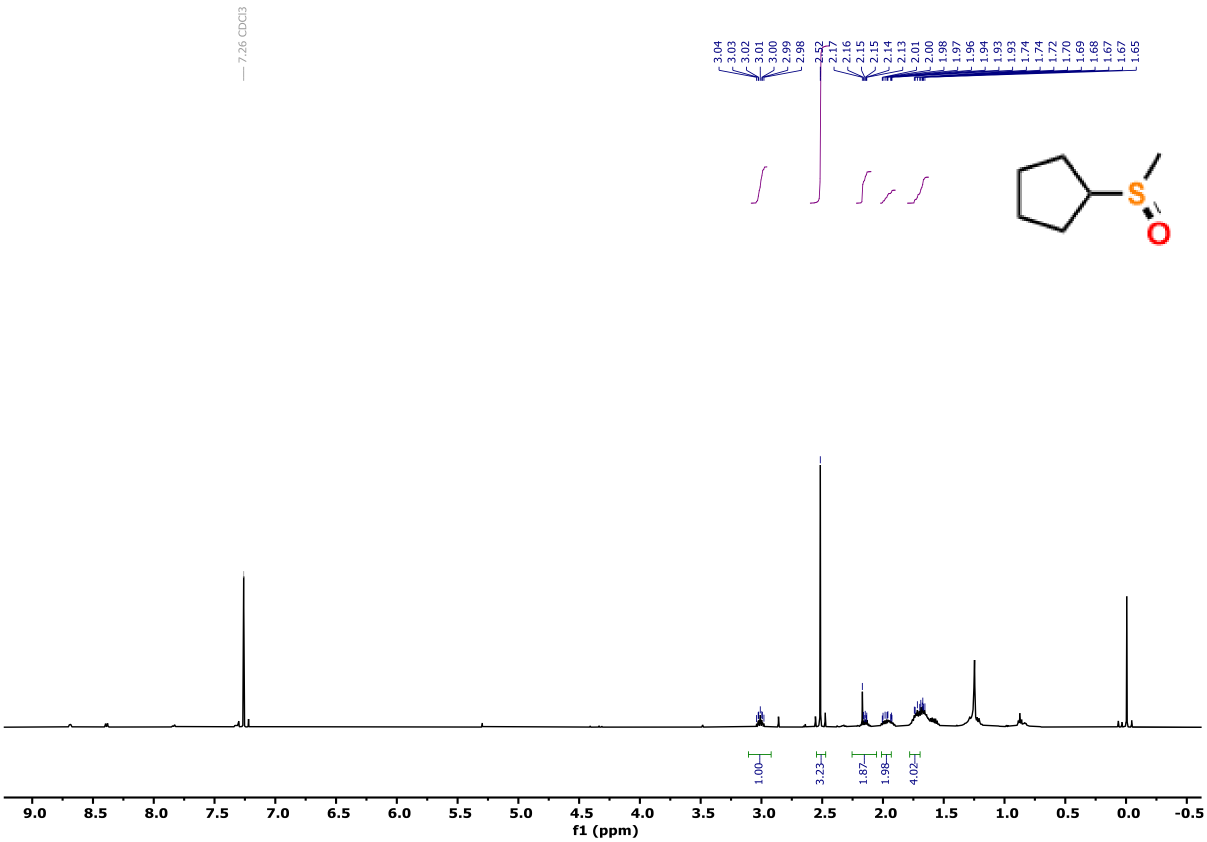

^{13}C NMR (126 MHz, $CDCl_3$)- Cyclopentyl methyl sulfoxide (30b)

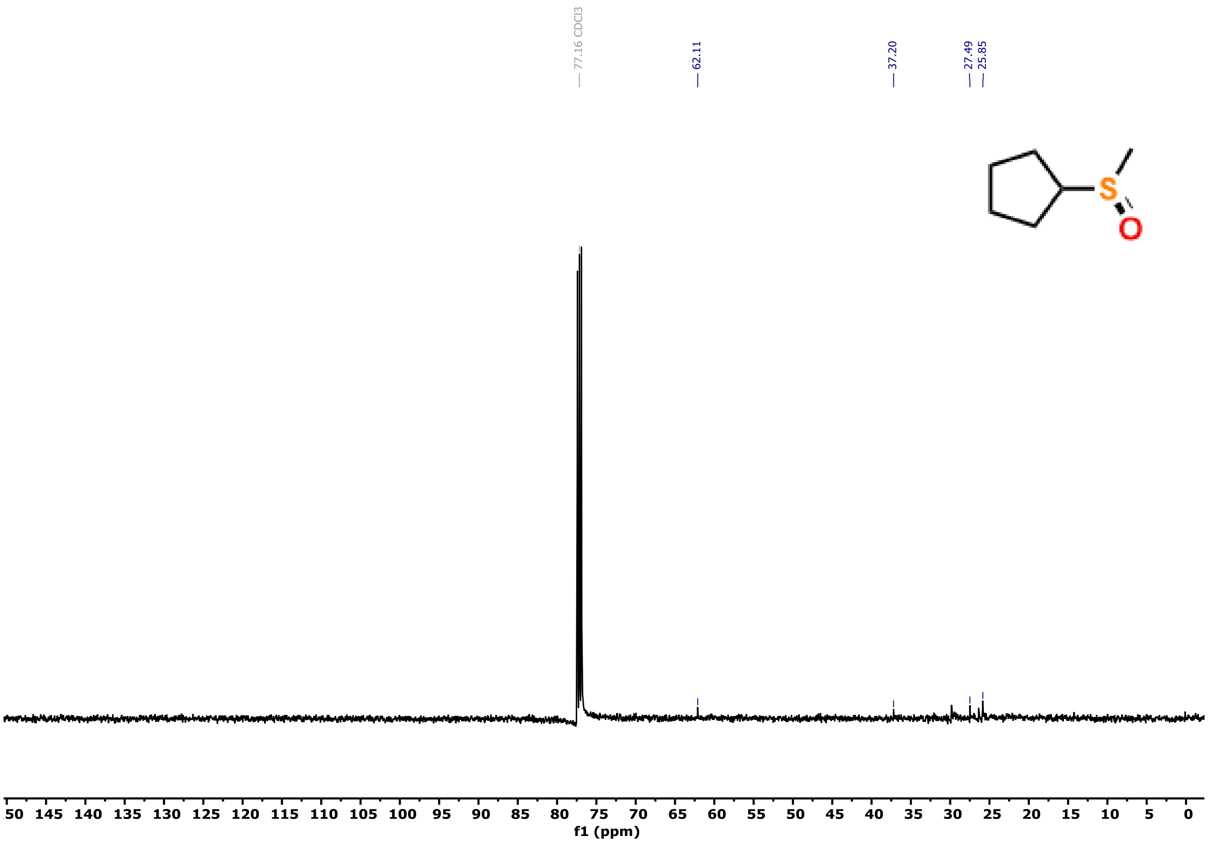

31.¹H NMR (500 MHz, $CDCl_3$)- Cyclohexyl methyl sulfoxide (31b)

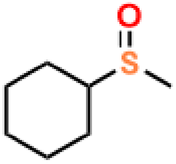

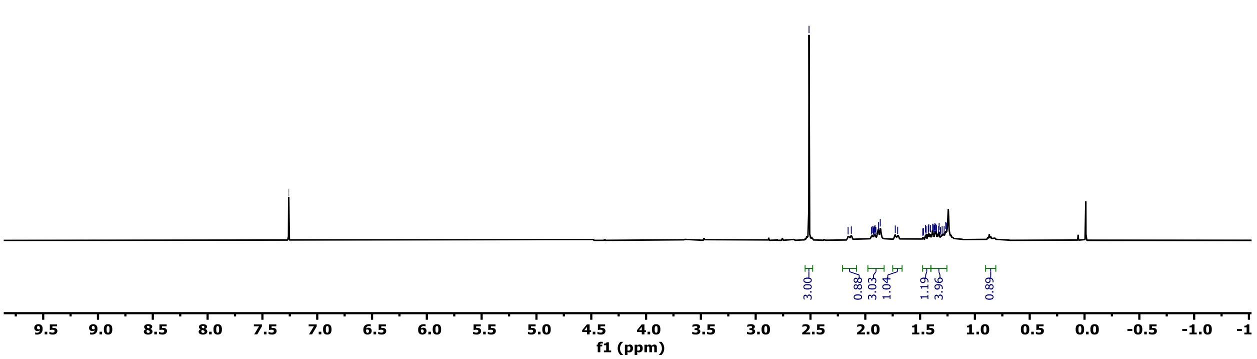

^{13}C NMR (126 MHz, $CDCl_3$)- Cyclohexyl methyl sulfoxide (31b)

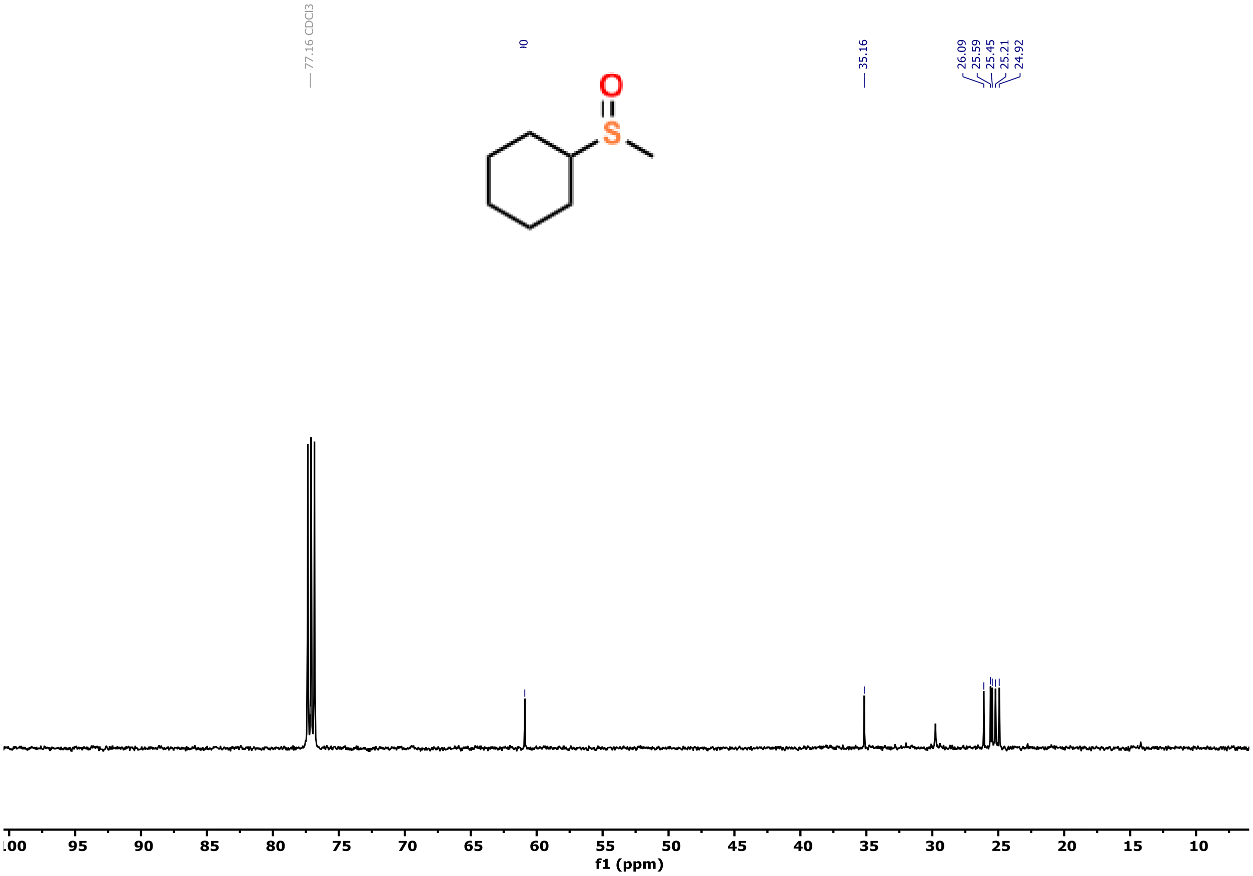

32. ^{1}H NMR (500 MHz, $CDCl_3$)- (Methylsulfinyl)adamantane (32b)

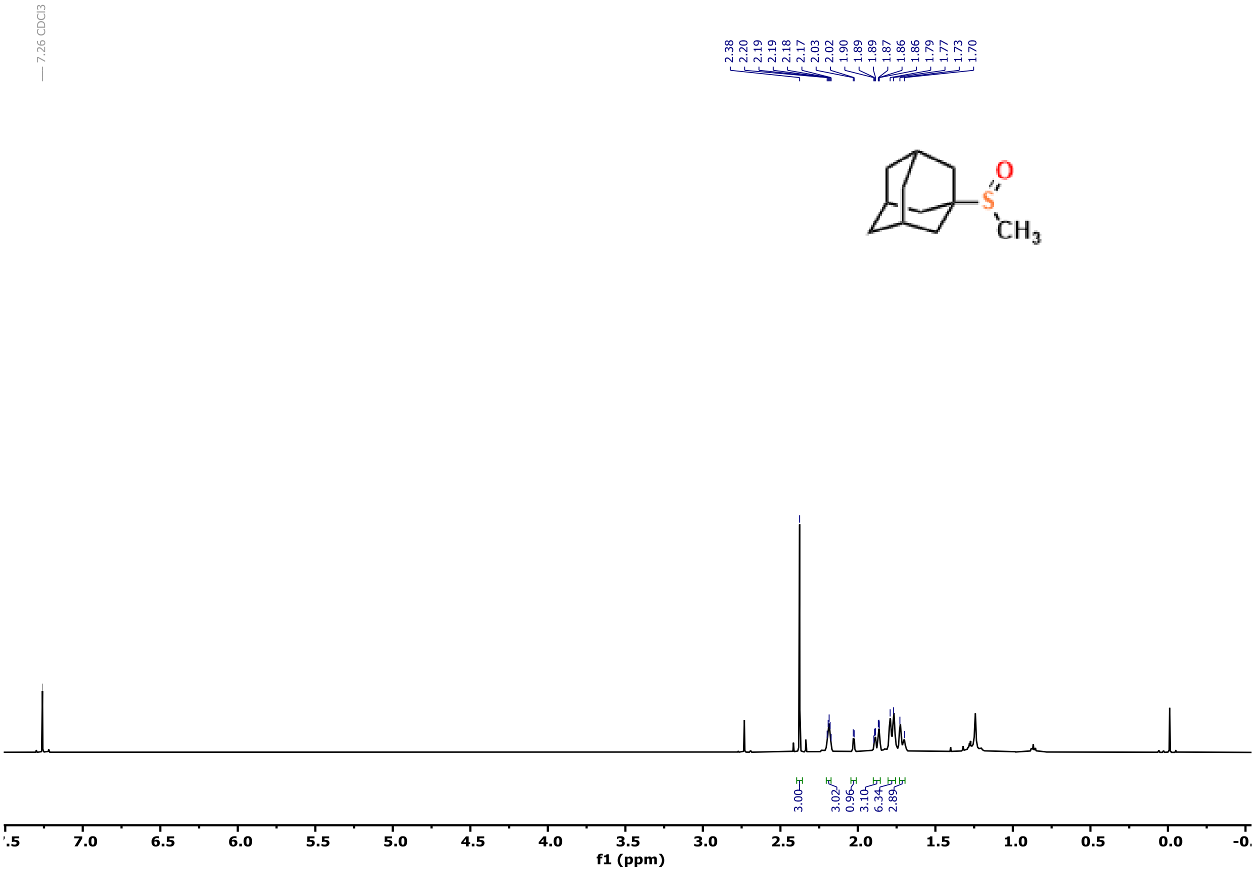

^{13}C NMR (126 MHz, $CDCl_3$)- (Methylsulfinyl)adamantane (32b)

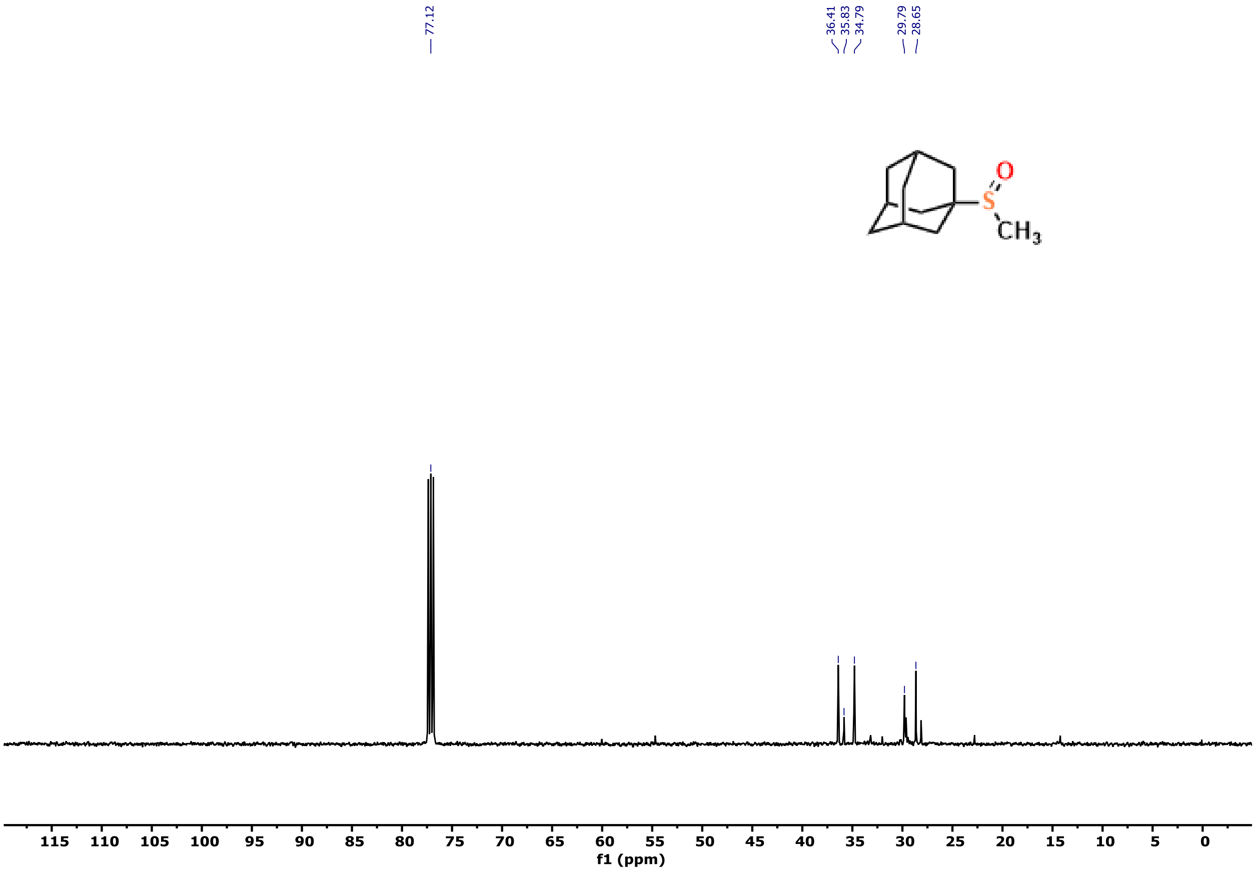

33. ^{1}H NMR (500 MHz, $CDCl_3$)- (2-Methylthiosulfoxide) pyridine (33b)

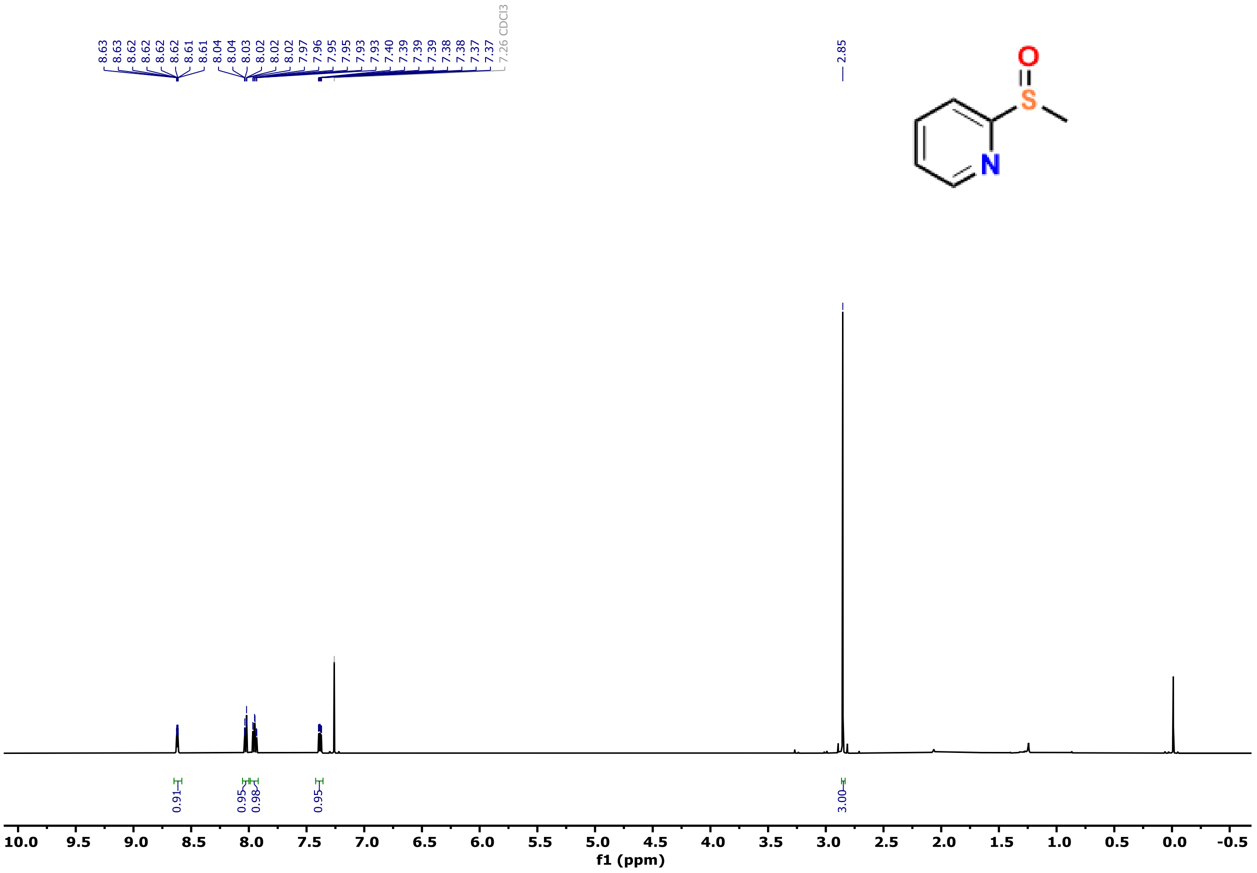

^{13}C NMR (126 MHz, $CDCl_3$)- (2-Methylthiosulfoxide) pyridine (33b)

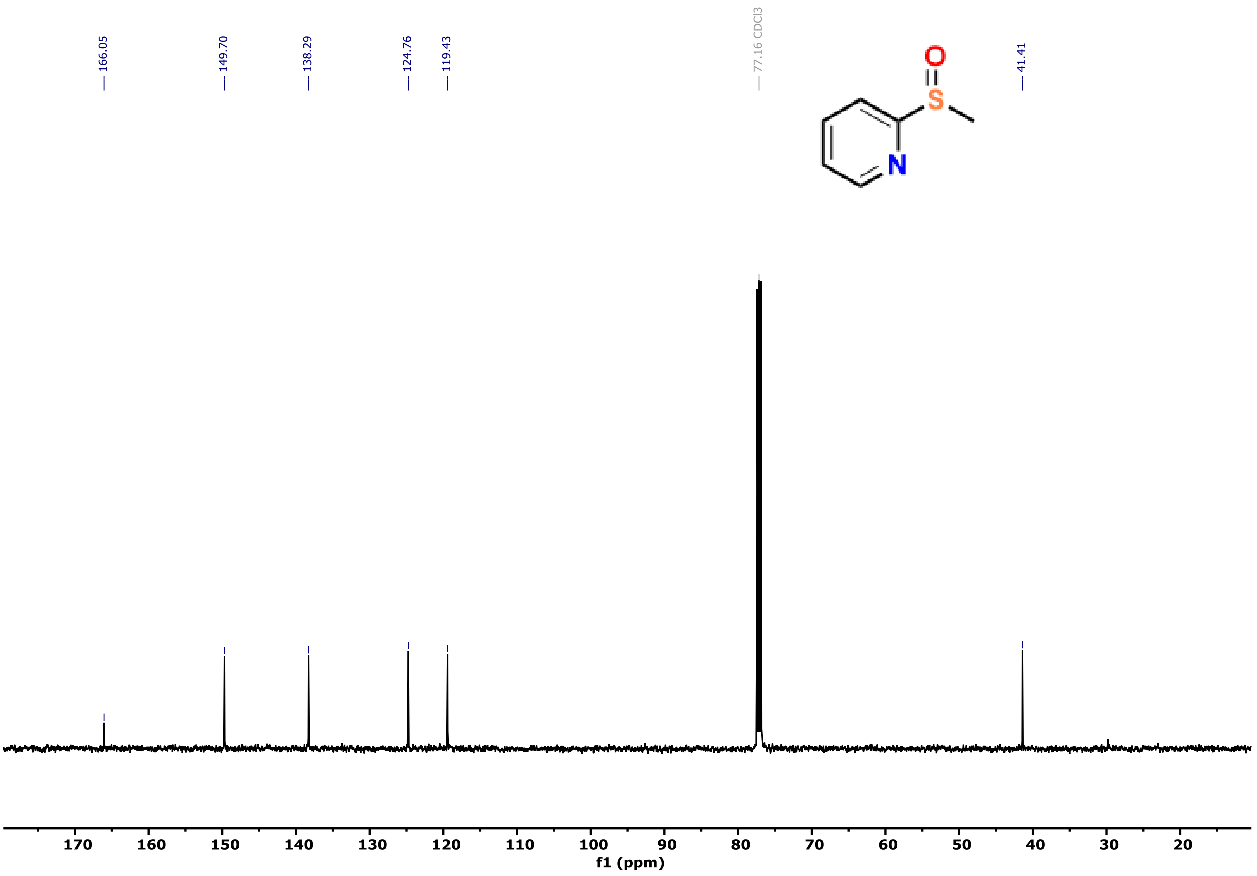

34. ^{1}H NMR (500 MHz, $CDCl_3$)-2-(methylthiosulfoxide) pyrazine (34b)

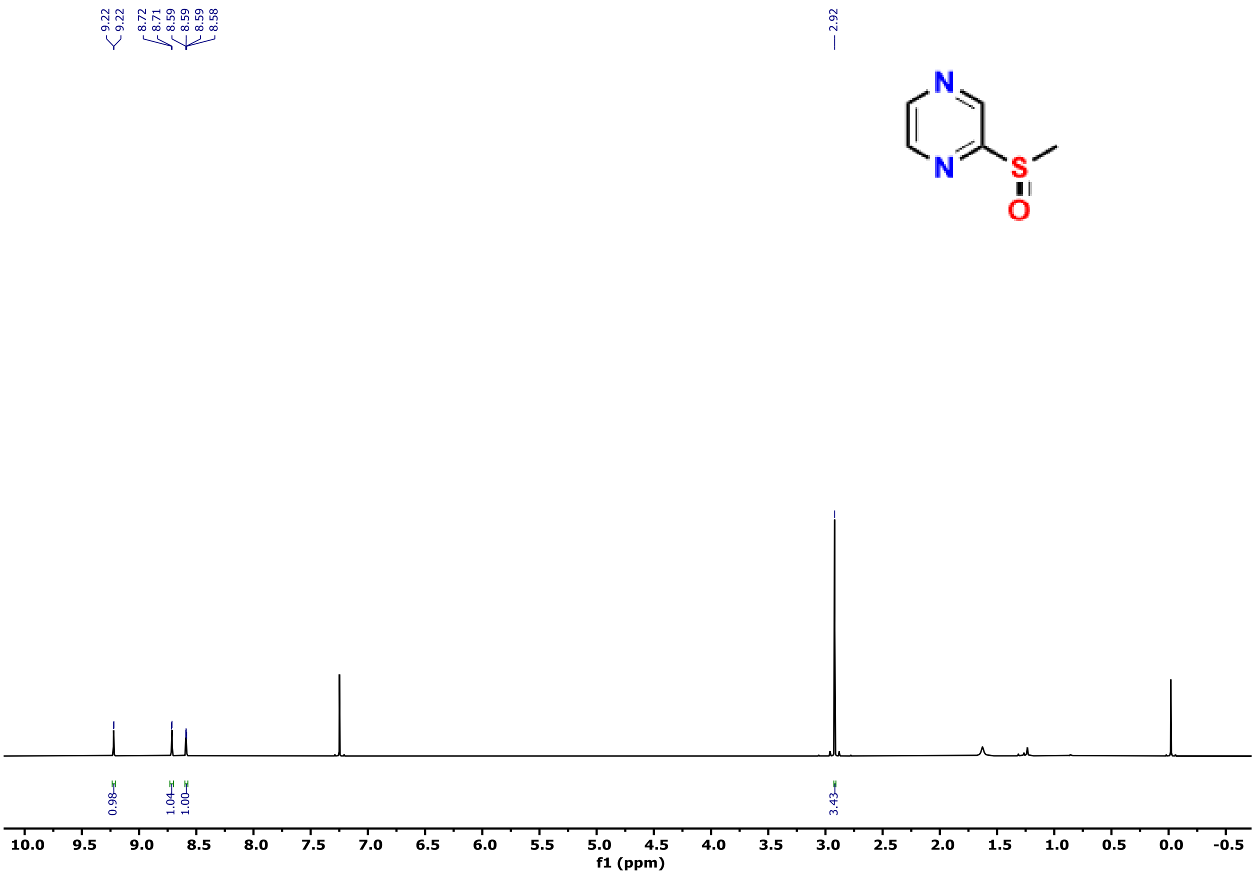

^{13}C NMR (126 MHz, $CDCl_3$)-2-(methylthiosulfoxide)pyrazine (34b)

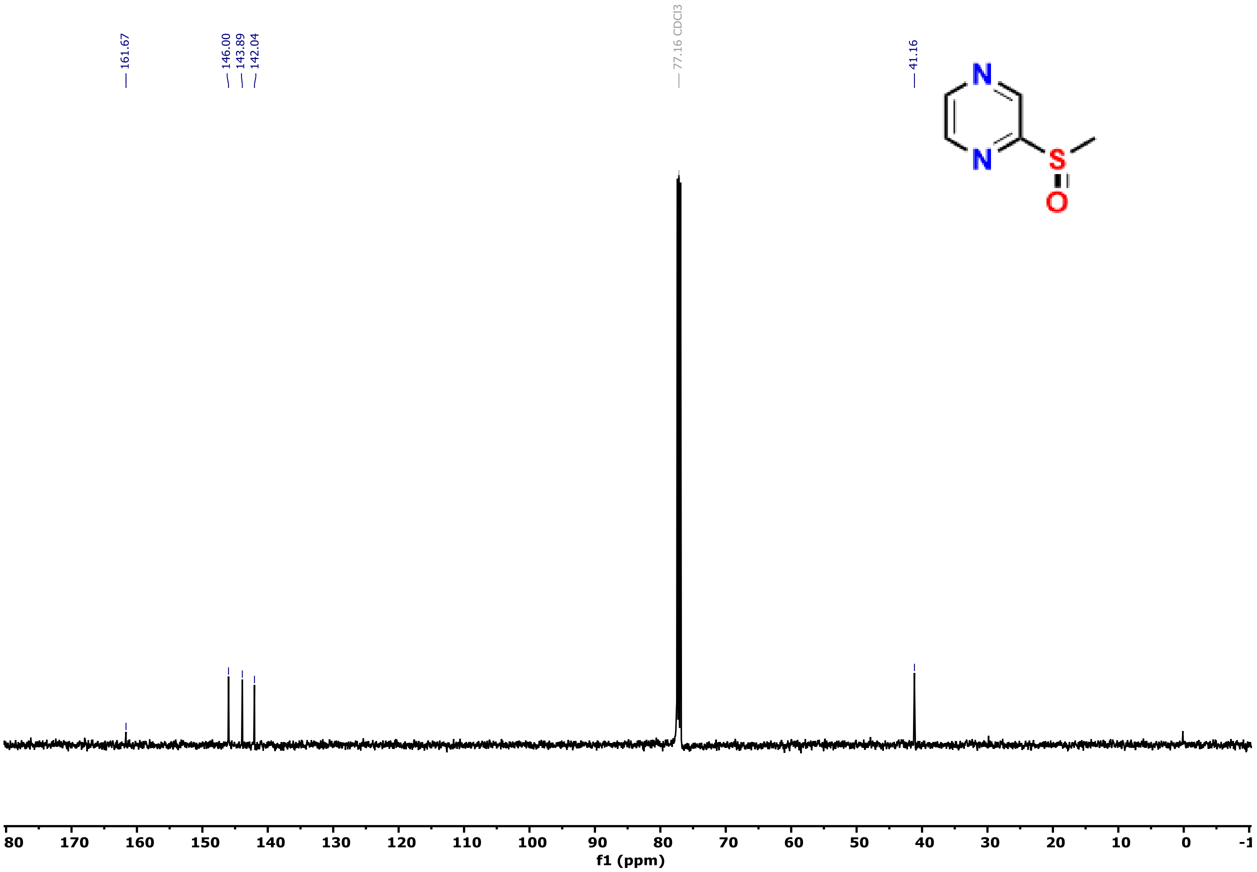

35.^{1}H NMR (500 MHz, CDCl₃)-4-(4-(methylthiosulfoxide)phenyl)pyridine (35b)

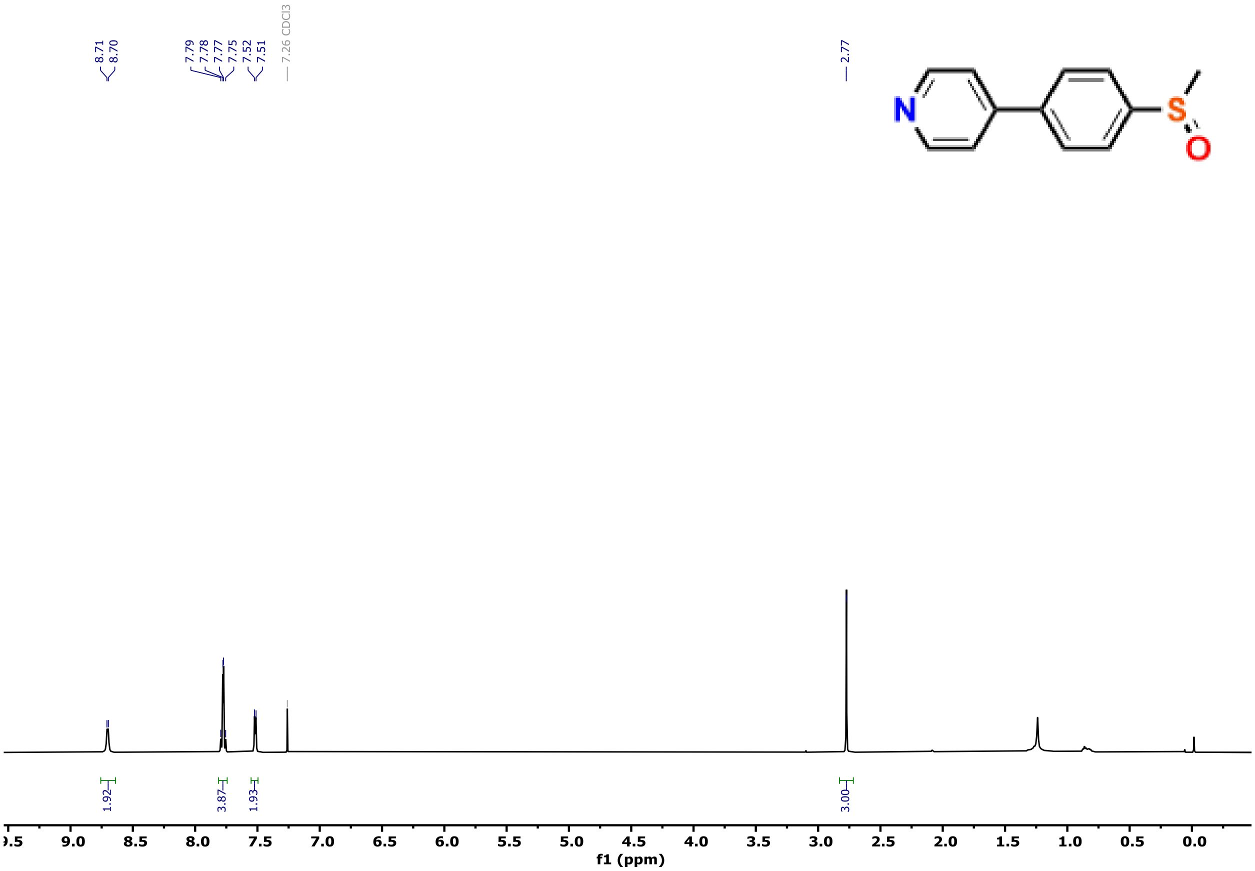

^{13}C NMR (126 MHz, $CDCl_3$)- 4-(4-(methylthiosulfoxide)phenyl)pyridine (35b)

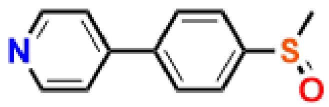

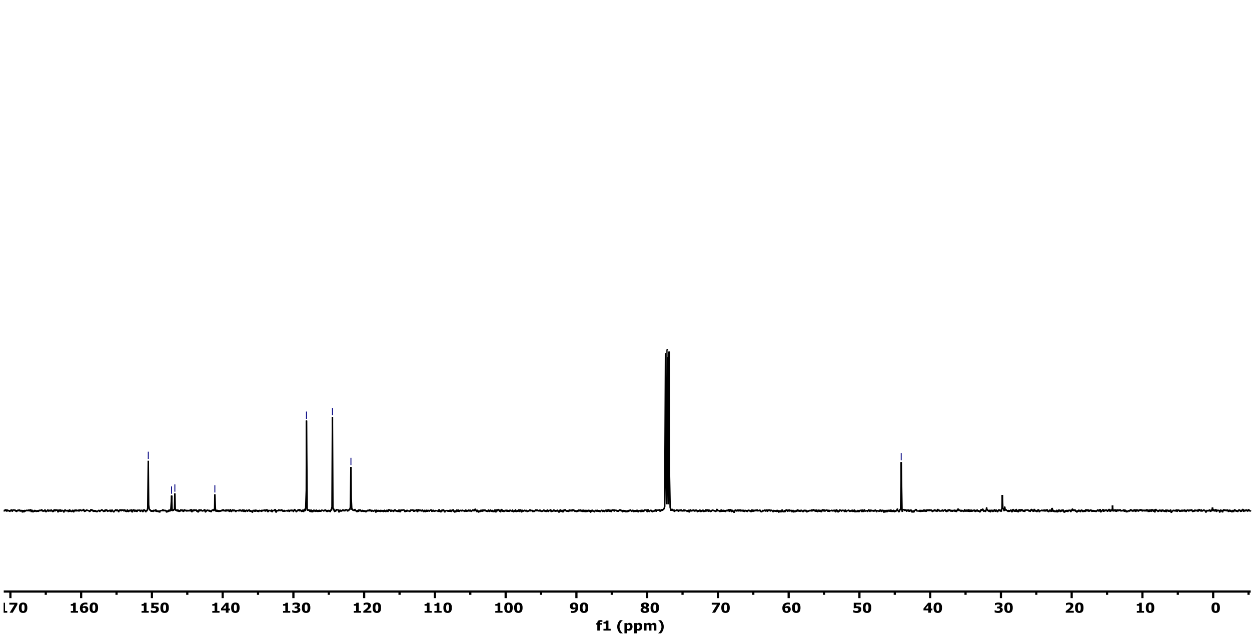

36. ^{1}H NMR (500 MHz, $CDCl_3$)-)- 6-(methylsulfinyl)-2,2'-bipyridine (36b)

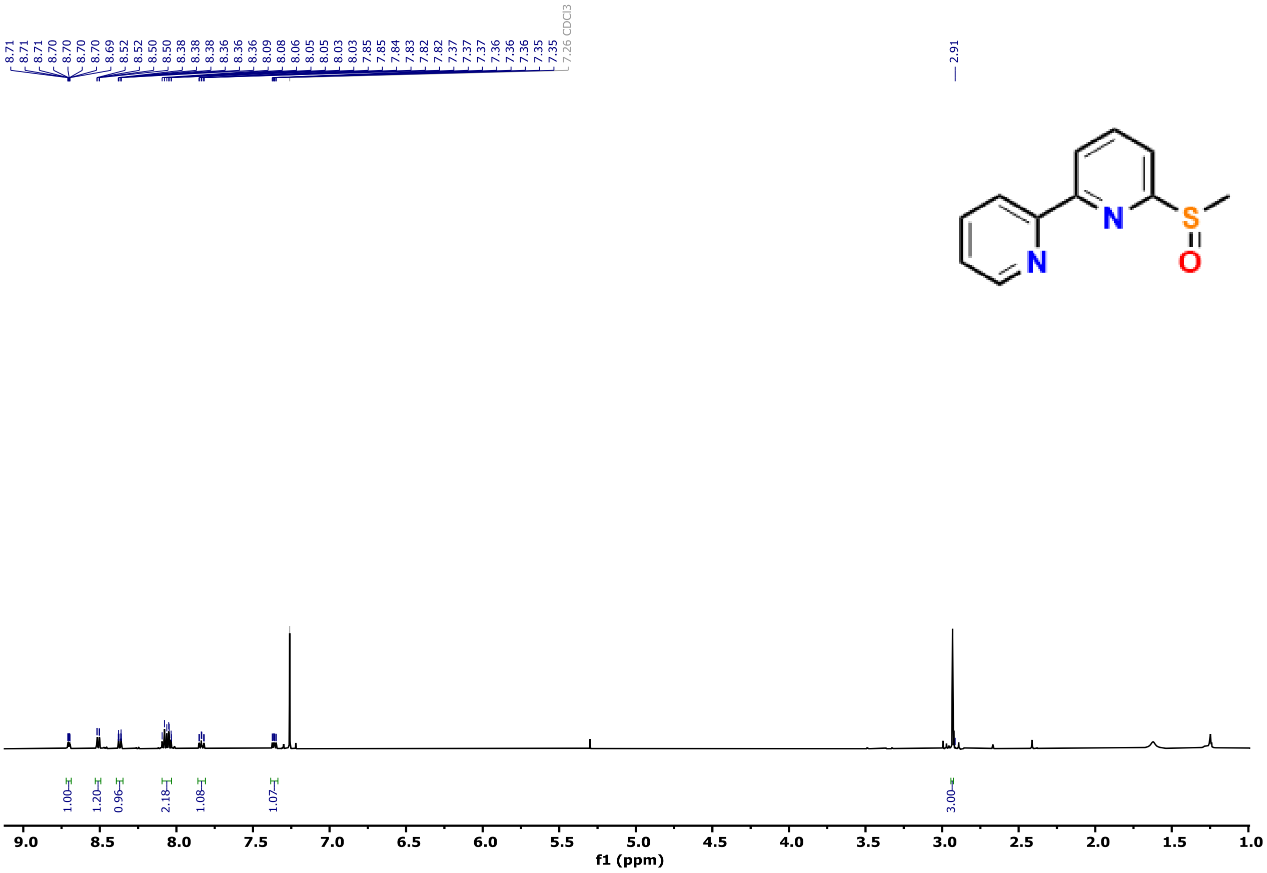

^{13}C NMR (126 MHz, $CDCl_3$)- 6-(methylsulfinyl)-2,2'-bipyridine (36b)

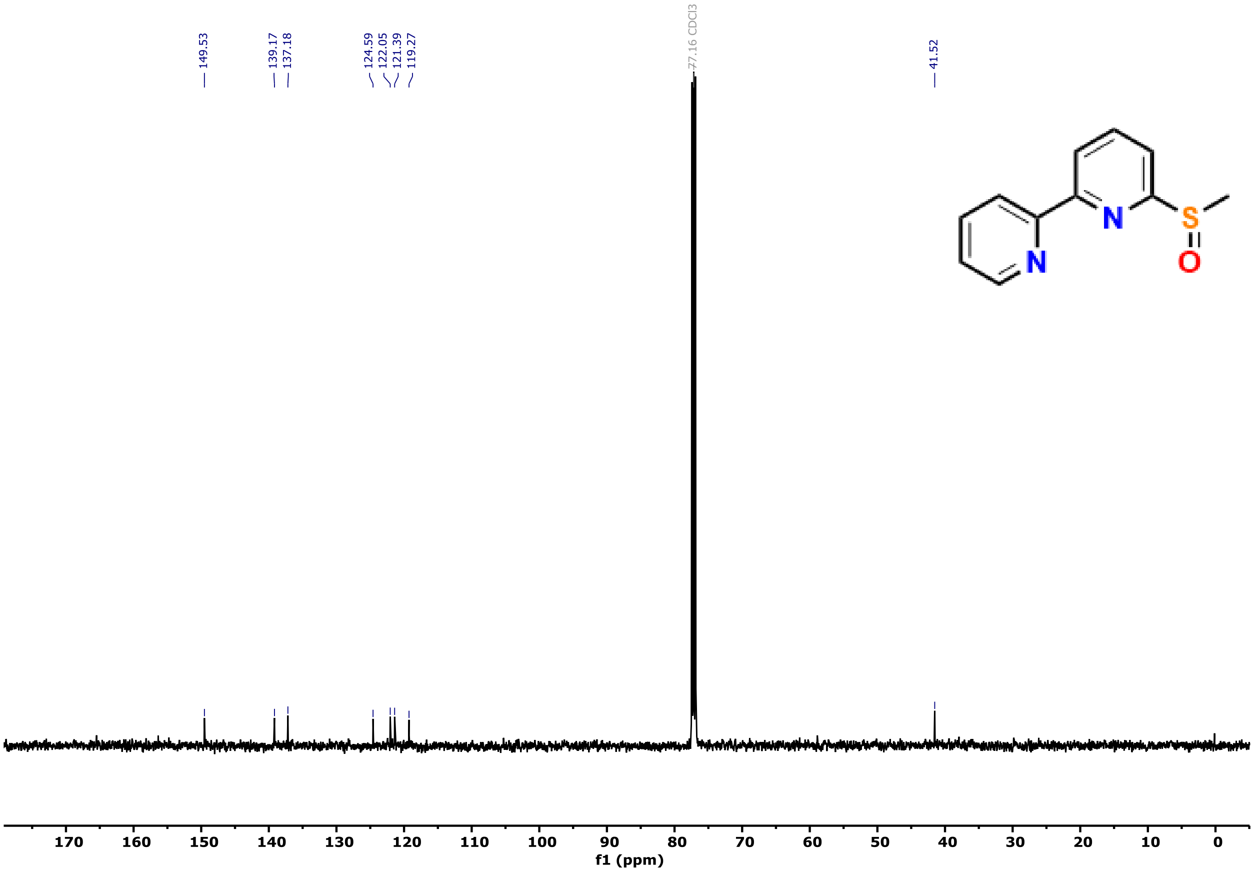

37. ¹H NMR (500 MHz, $CDCl_3$)- Thieno[2,3-b]pyridine 1-oxide (37b)

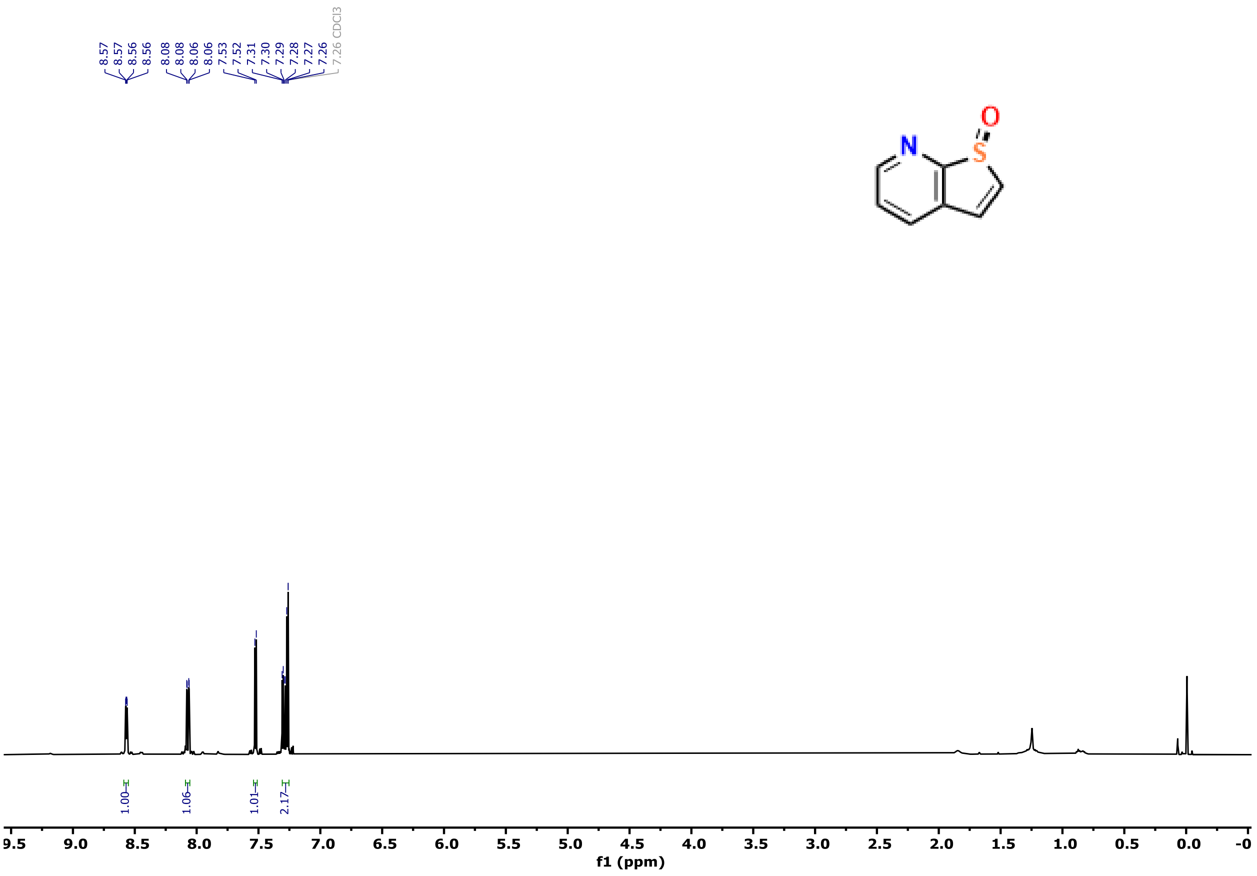

^{13}C NMR (126 MHz, $CDCl_3$)- Thieno[2,3-b]pyridine 1-oxide (37b)

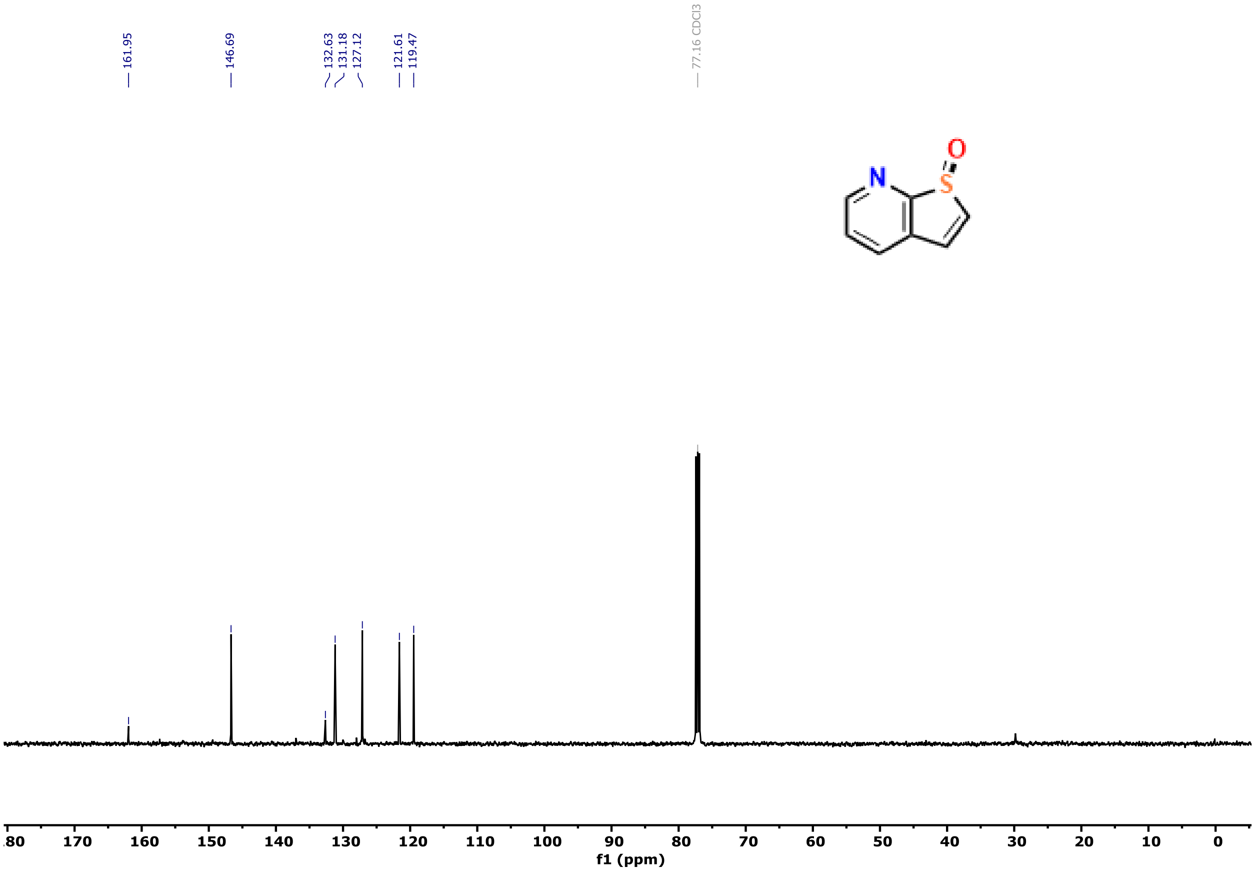

38. ^{1}H NMR (500 MHz, DMSO-*d*$_6$)- 4-(4-(4-fluorophenyl)-2-(4-(methylsulfinyl)phenyl)-1H-imidazol-5-yl)pyridine (38b)

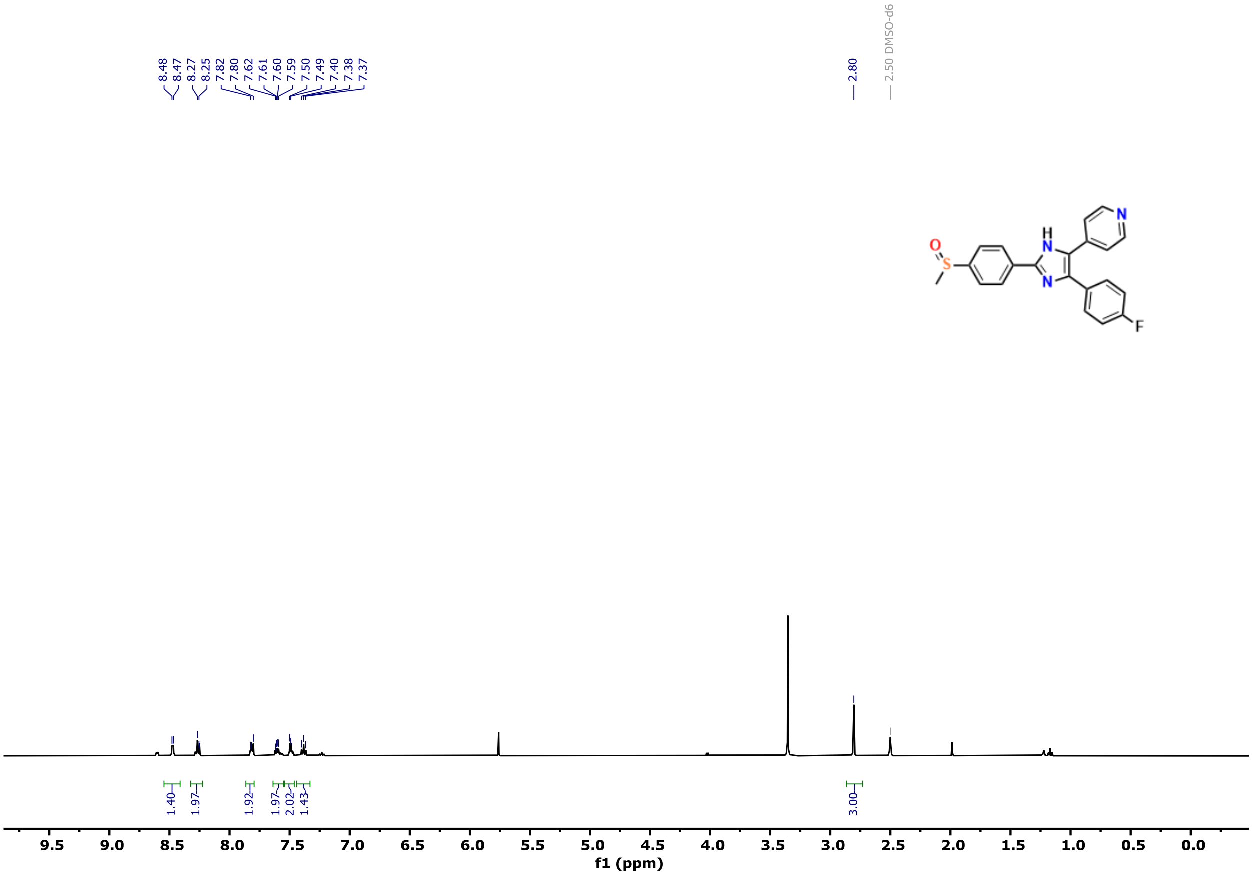

^{13}C NMR (126 MHz, DMSO-d_6)- 4-(4-(4-fluorophenyl)-2-(4-(methylsulfinyl)phenyl)-1H-imidazol-5-yl)pyridine (38b)

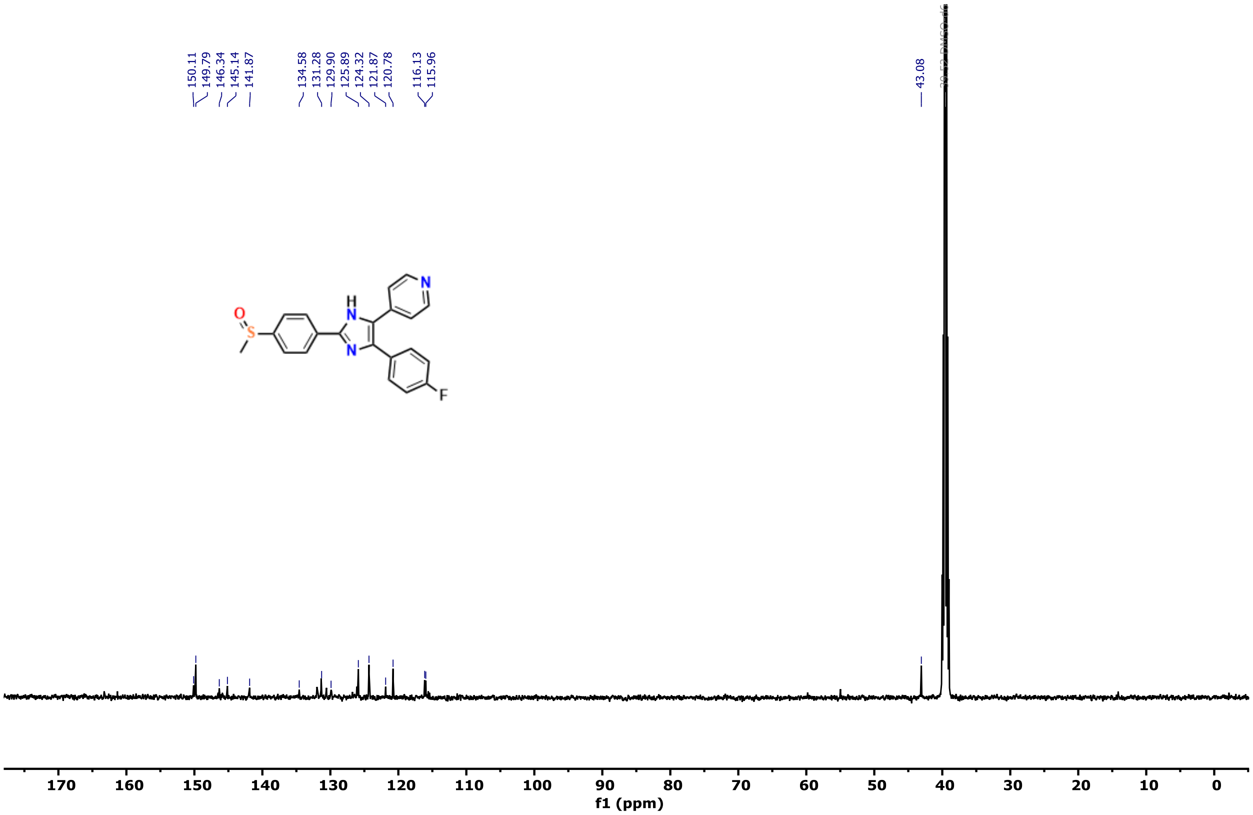

^{19}F NMR (471 MHz, DMSO-d_6)- 4-(4-(4-fluorophenyl)-2-(4-(methylsulfinyl)phenyl)-1H-imidazol-5-yl)pyridine (38b)

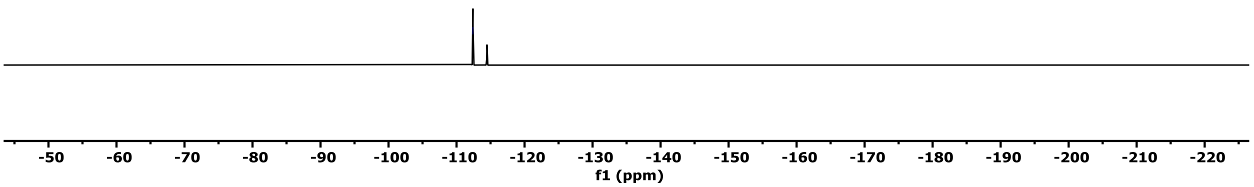

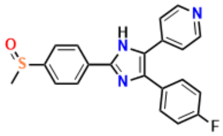

Spectral data of Synthesized substrates:

1. ^{1}H NMR (500 MHz, $CDCl_3$)- 2,6-Dimethylbenzenethioanisole (5a)

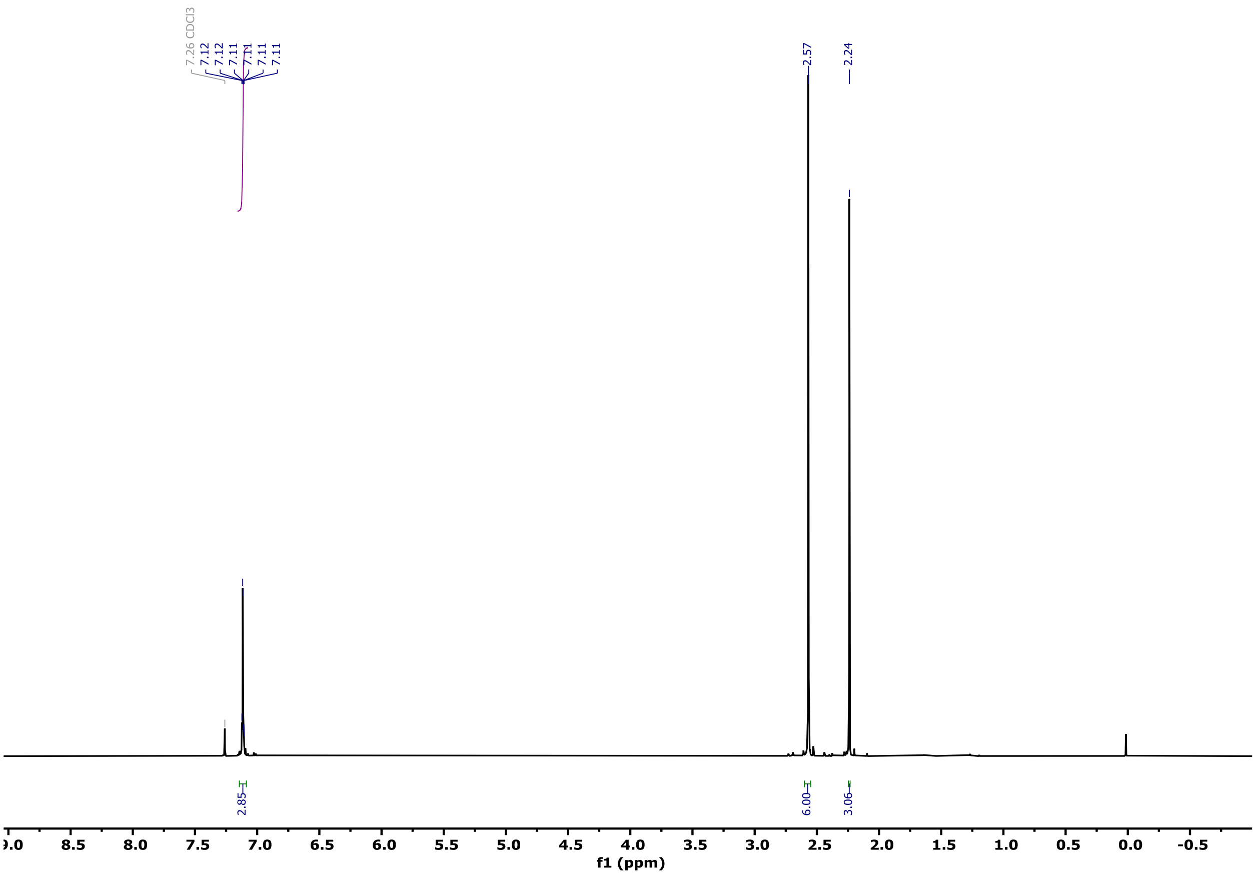

^{13}C NMR (126 MHz, $CDCl_3$)- 2,6-Dimethylbenzenethioanisole (5a)

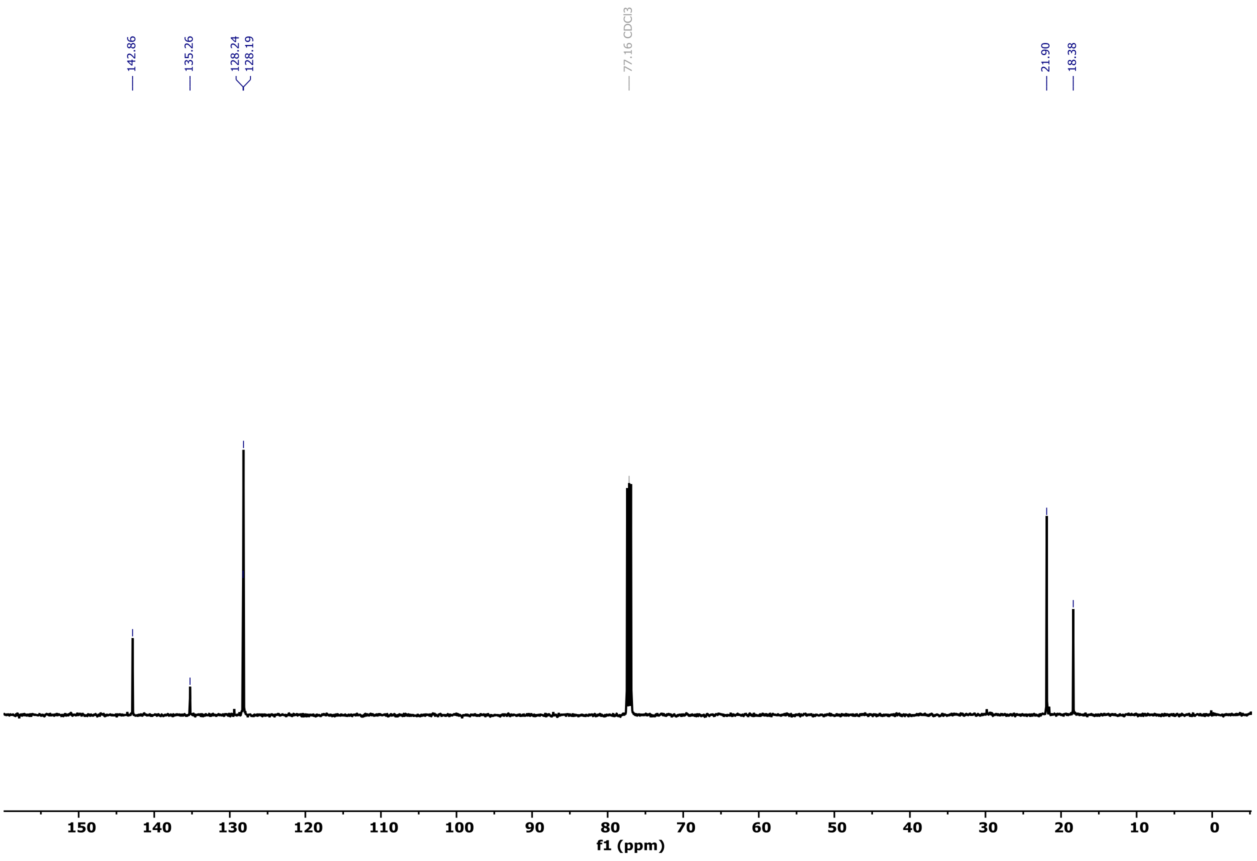

2. ^{1}H NMR (500 MHz, $CDCl_3$)- 4-MethylBenzeneThioanisole (2a)

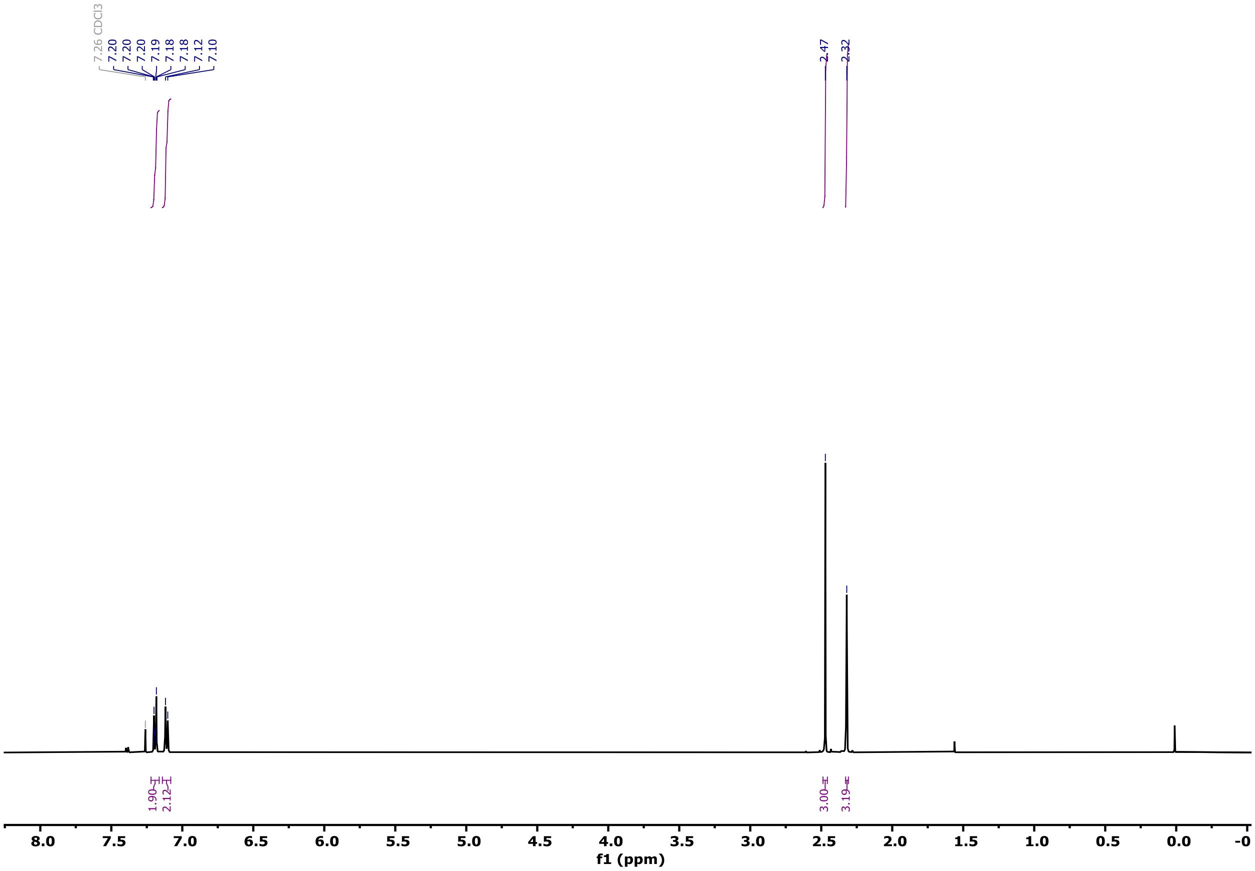

^{13}C NMR (126 MHz, $CDCl_3$)- 4-MethylBenzeneThioanisole (2a)

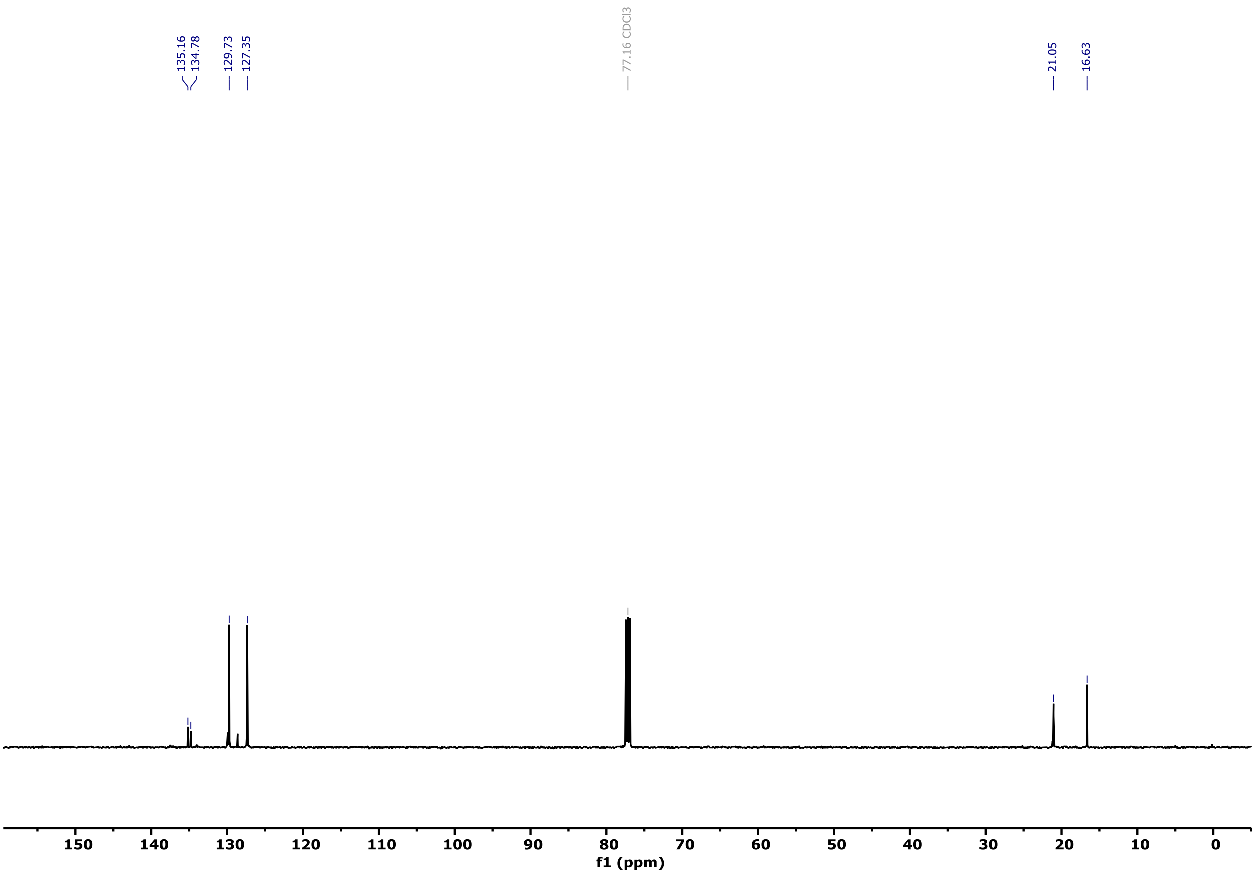

3. ^{1}H NMR (500 MHz, $CDCl_3$)- 4-tertbutylbenzenethioanisole (4a)

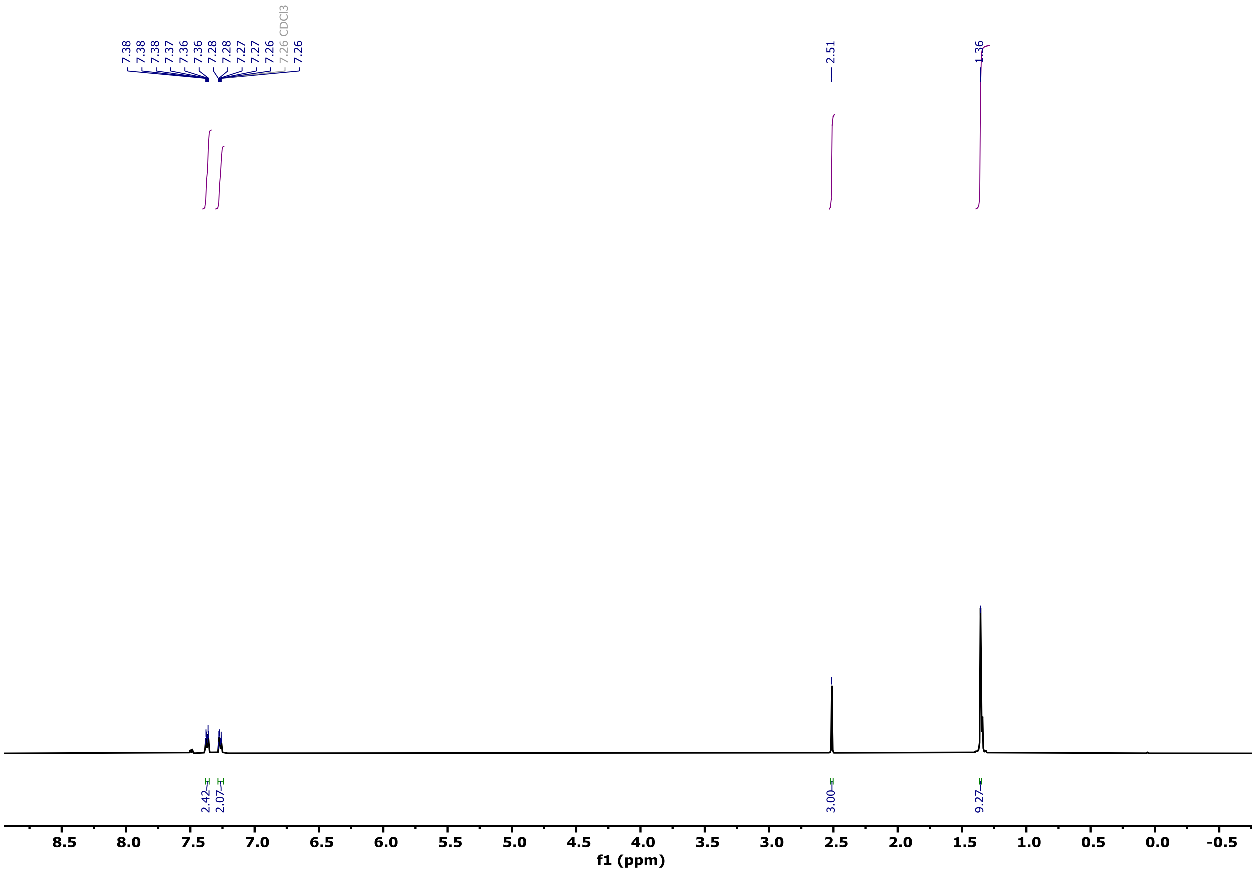

^{13}C NMR (126 MHz, $CDCl_3$)- 4-tertbutylbenzenethioanisole (4a)

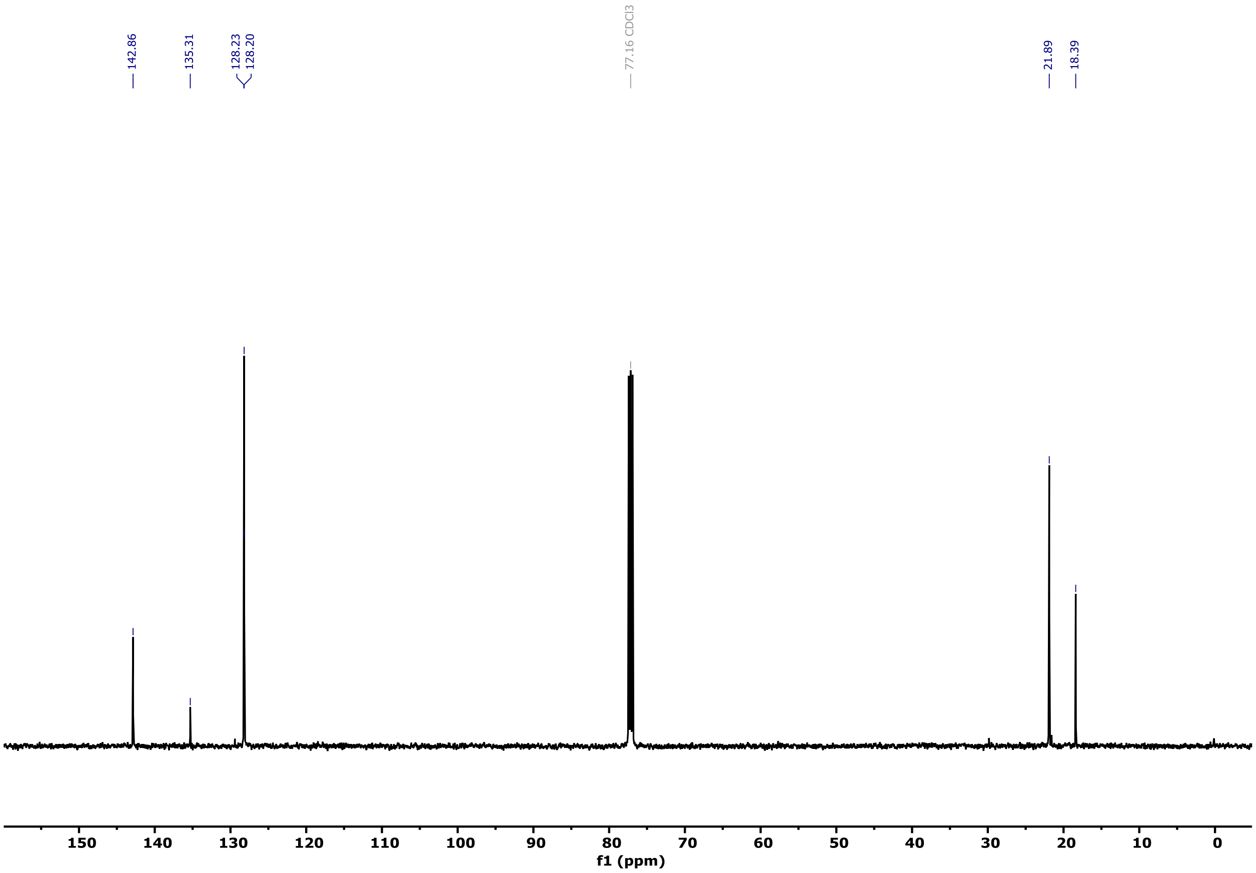

4. ^1H NMR (500 MHz, $CDCl_3$)- Cyclohexylmethylsulfide (31a)

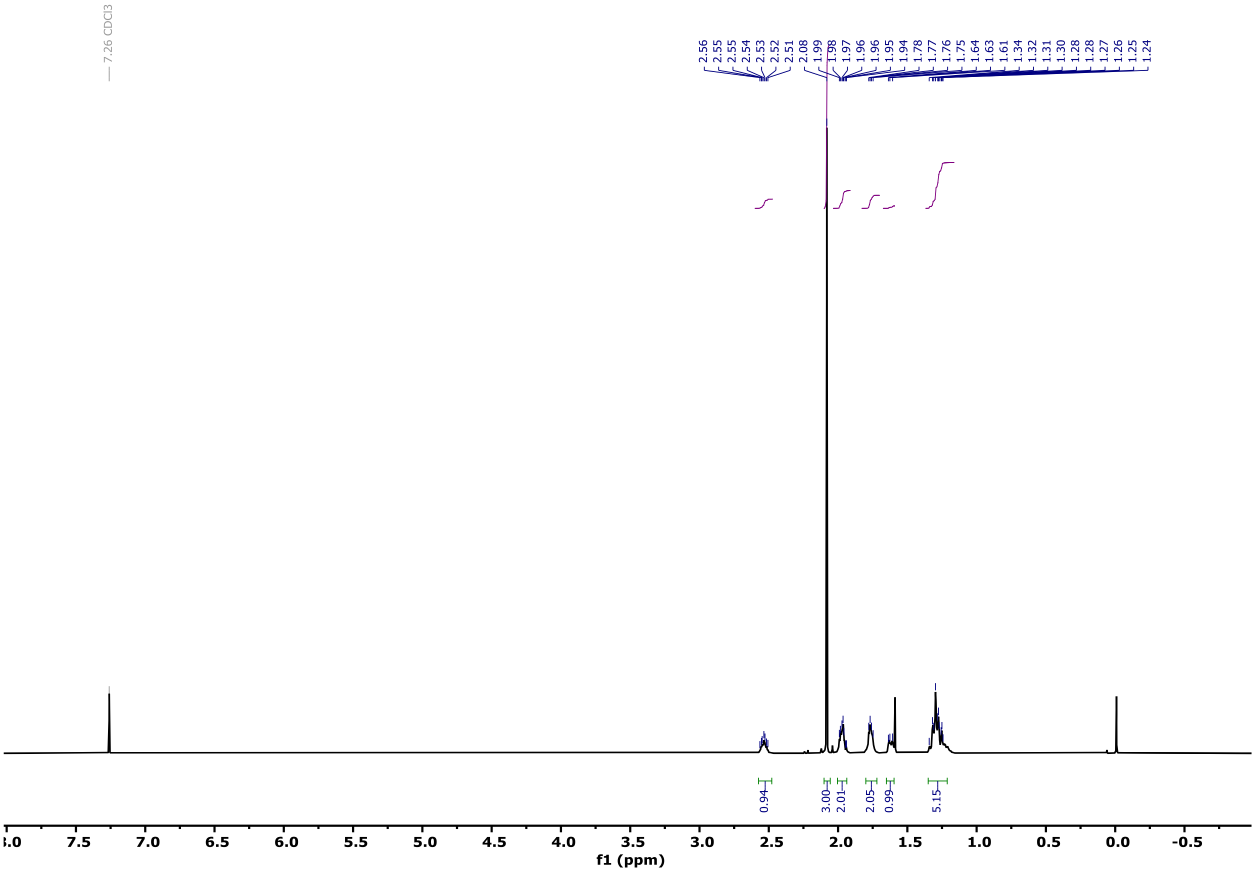

^{13}C NMR (126 MHz, CDCl$_3$)- Cyclohexylmethylsulfide (31a)

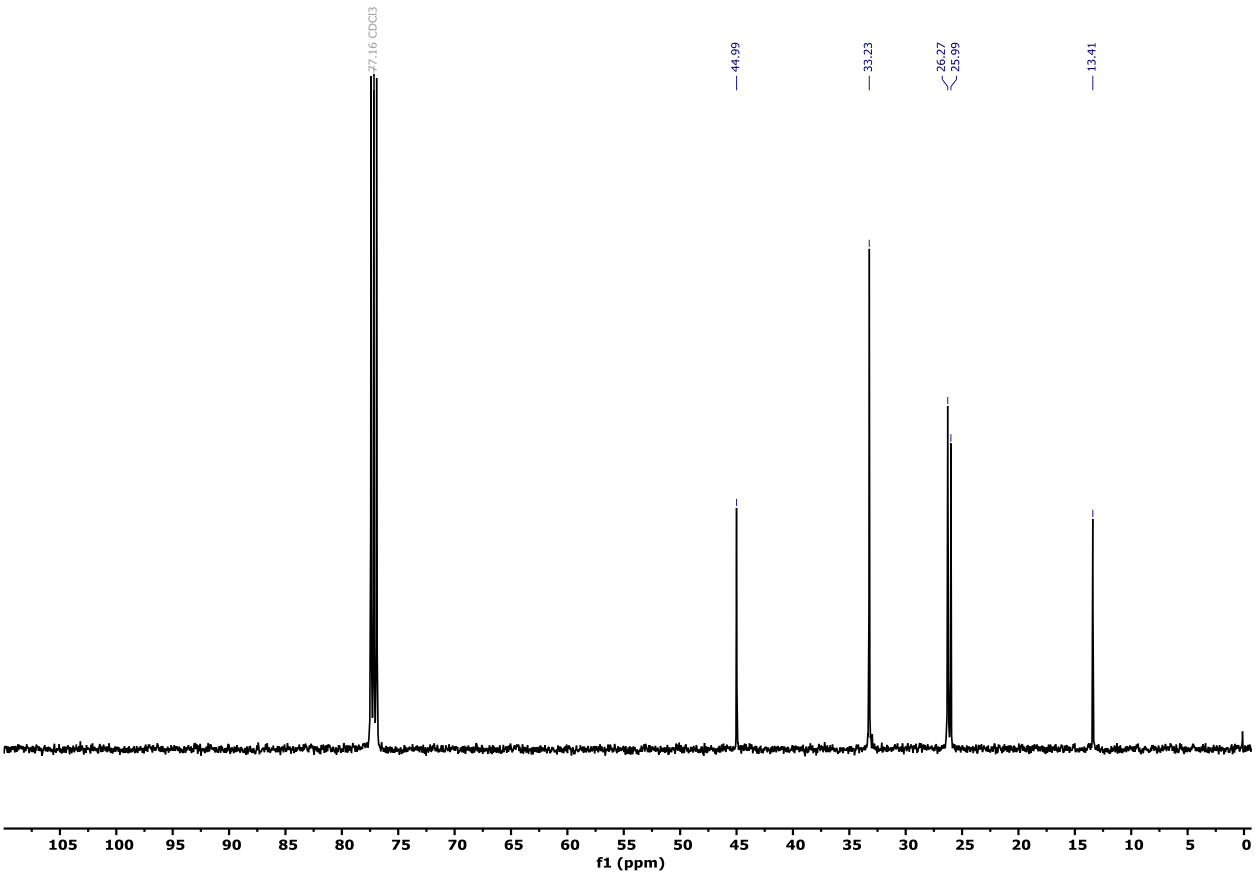

5. ^{1}H NMR (500 MHz, $CDCl_3$)- Hexylmethylsulfide (21a)

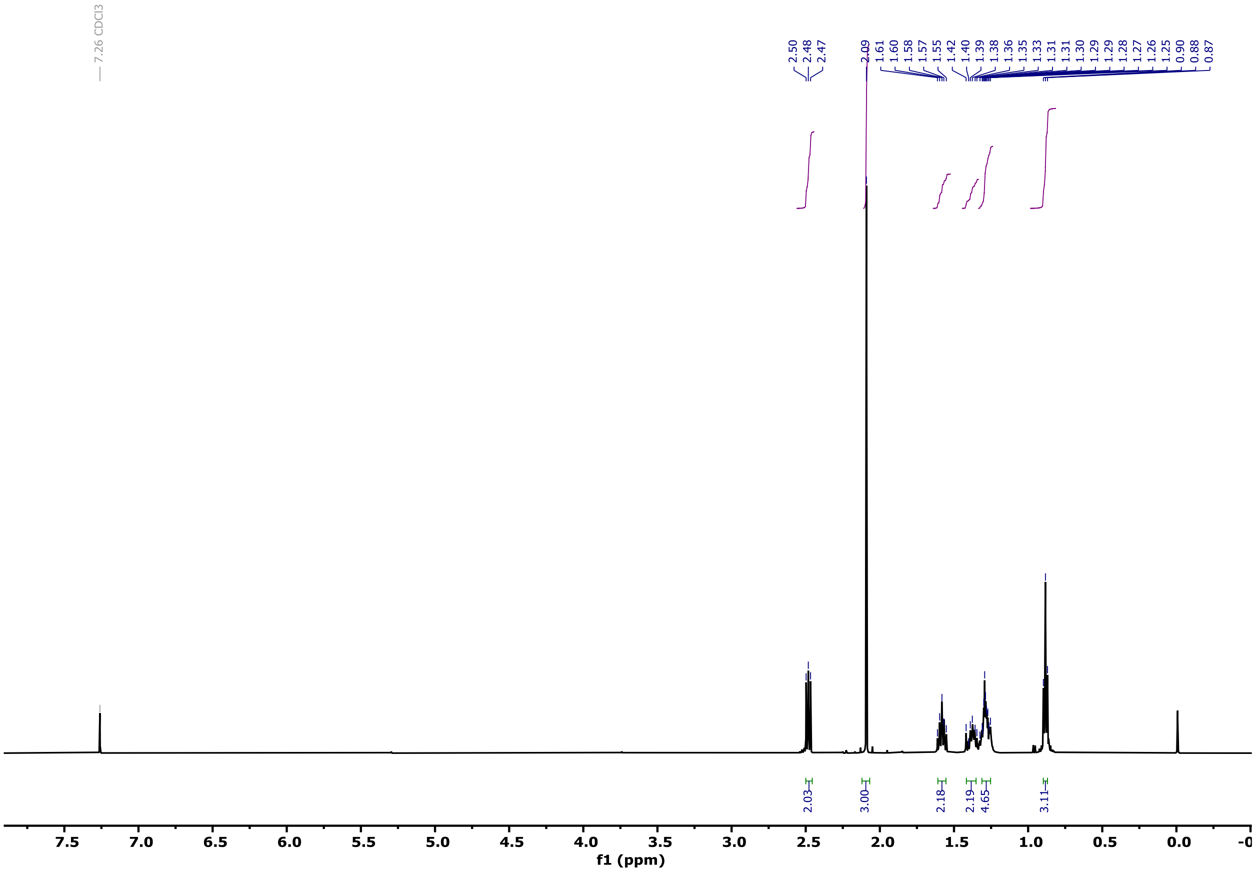

^{13}C NMR (126 MHz, $CDCl_3$)- Hexylmethylsulfide (21a)

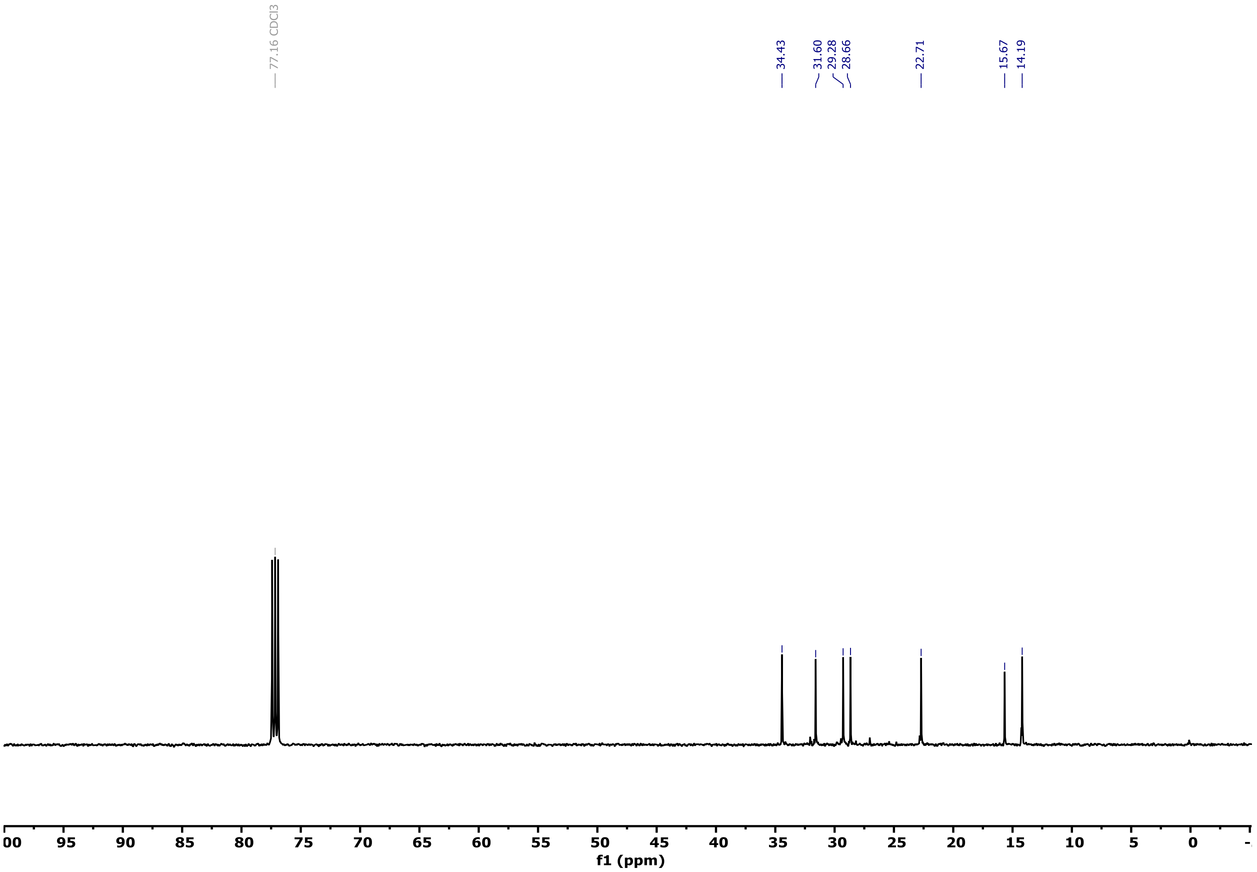

6. ^{1}H NMR (500 MHz, $CDCl_3$)- 4-[4-(Methylthio)phenyl]pyridine (35a)

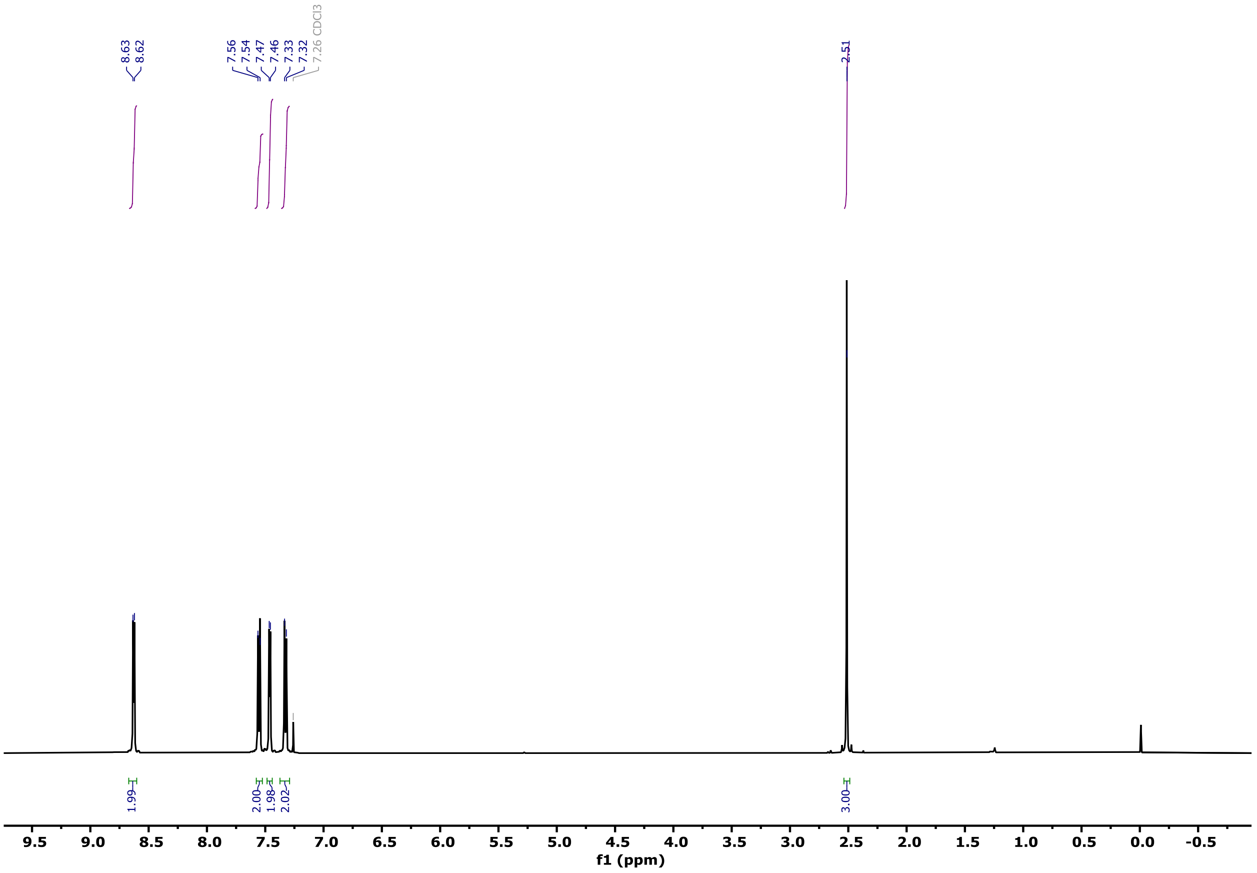

^{13}C NMR (126 MHz, $CDCl_3$)- 4-[4-(Methylthio)phenyl]pyridine (35a)

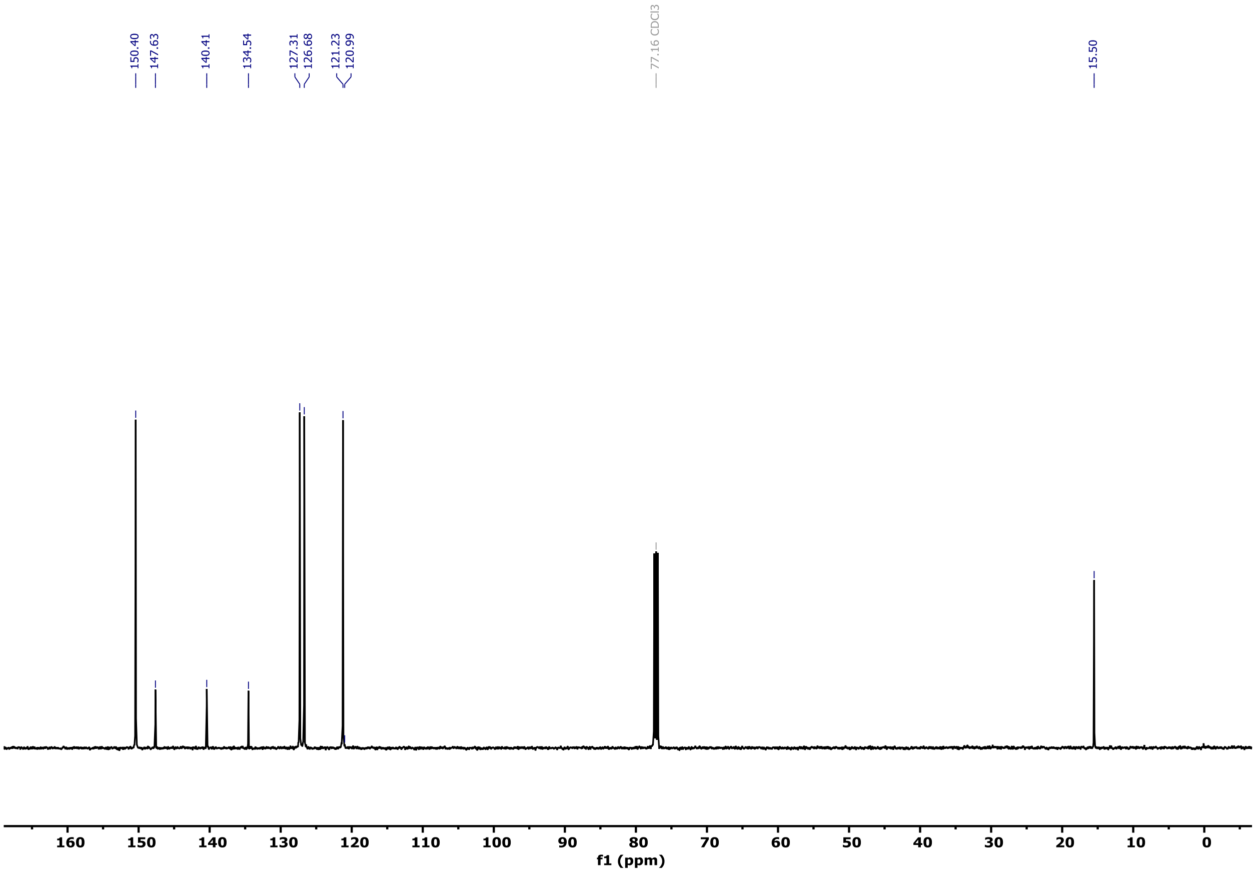

7. ^{1}H NMR (500 MHz, $CDCl_3$)- 4-(Benzyloxy)phenylmethyl Sulfane (25a)

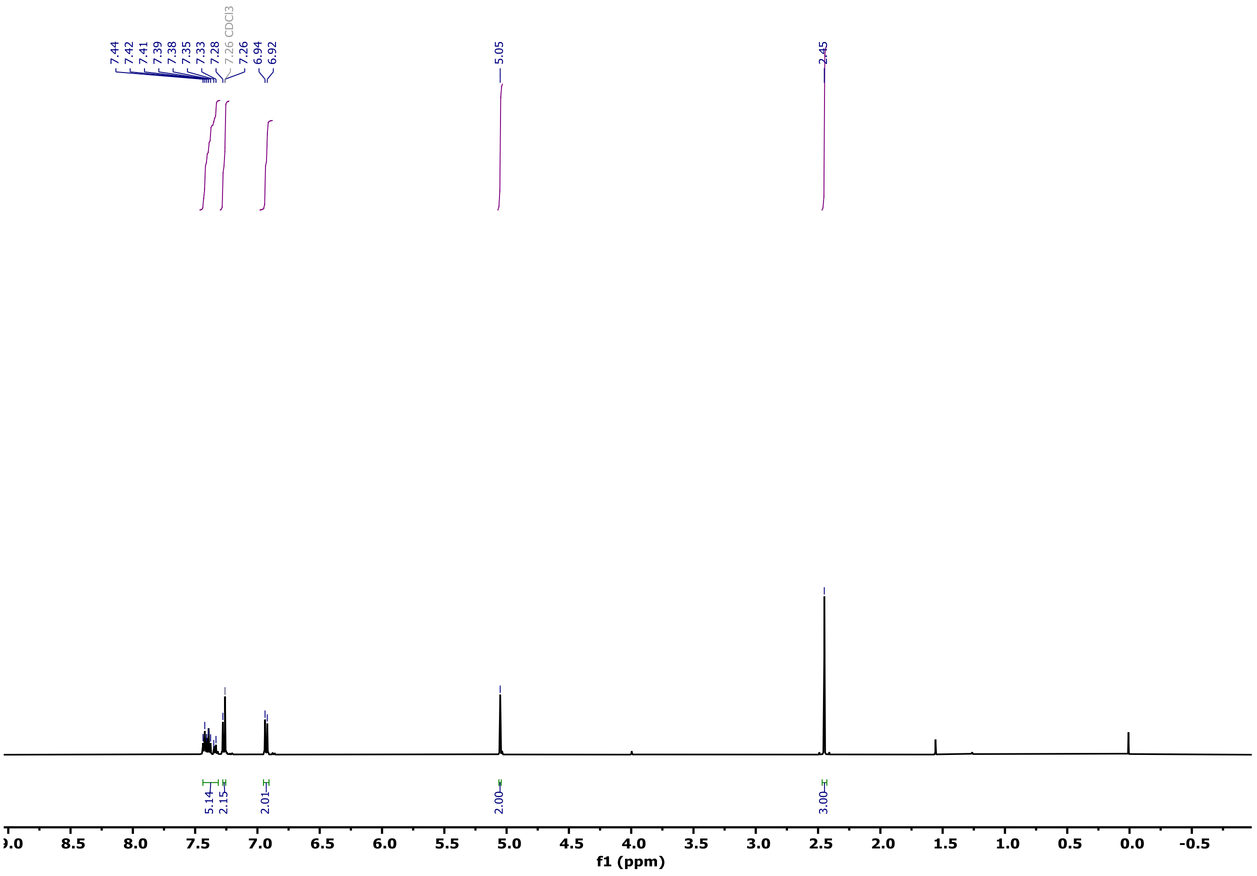

^{13}C NMR (126 MHz, $CDCl_3$)- 4-(Benzyloxy)phenylmethyl Sulfane (25a)

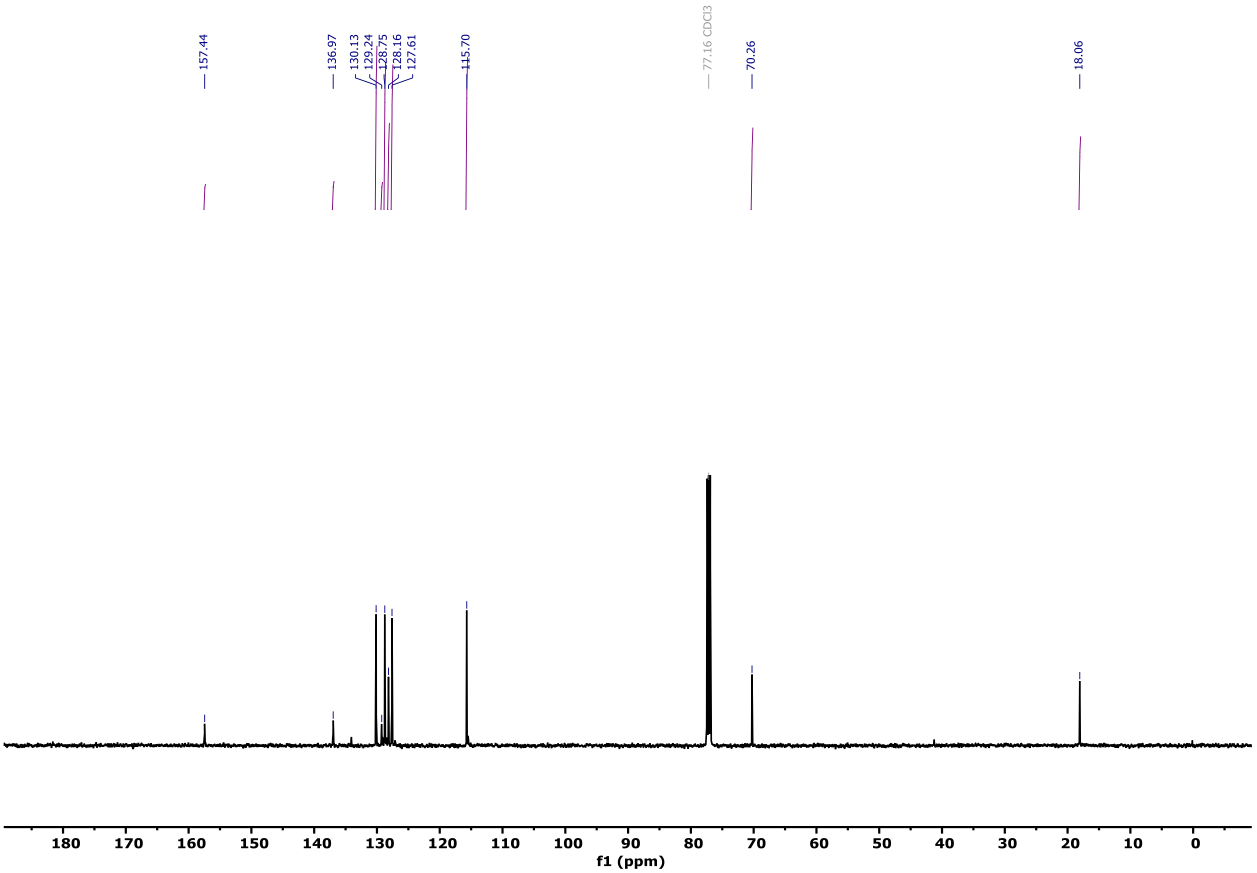

8. ^{1}H NMR (500 MHz, $CDCl_3$)- 4,4,5,5-tetramethyl-2-(4-(methylthio)phenyl)-1,3,2-dioxaborolane (18a)

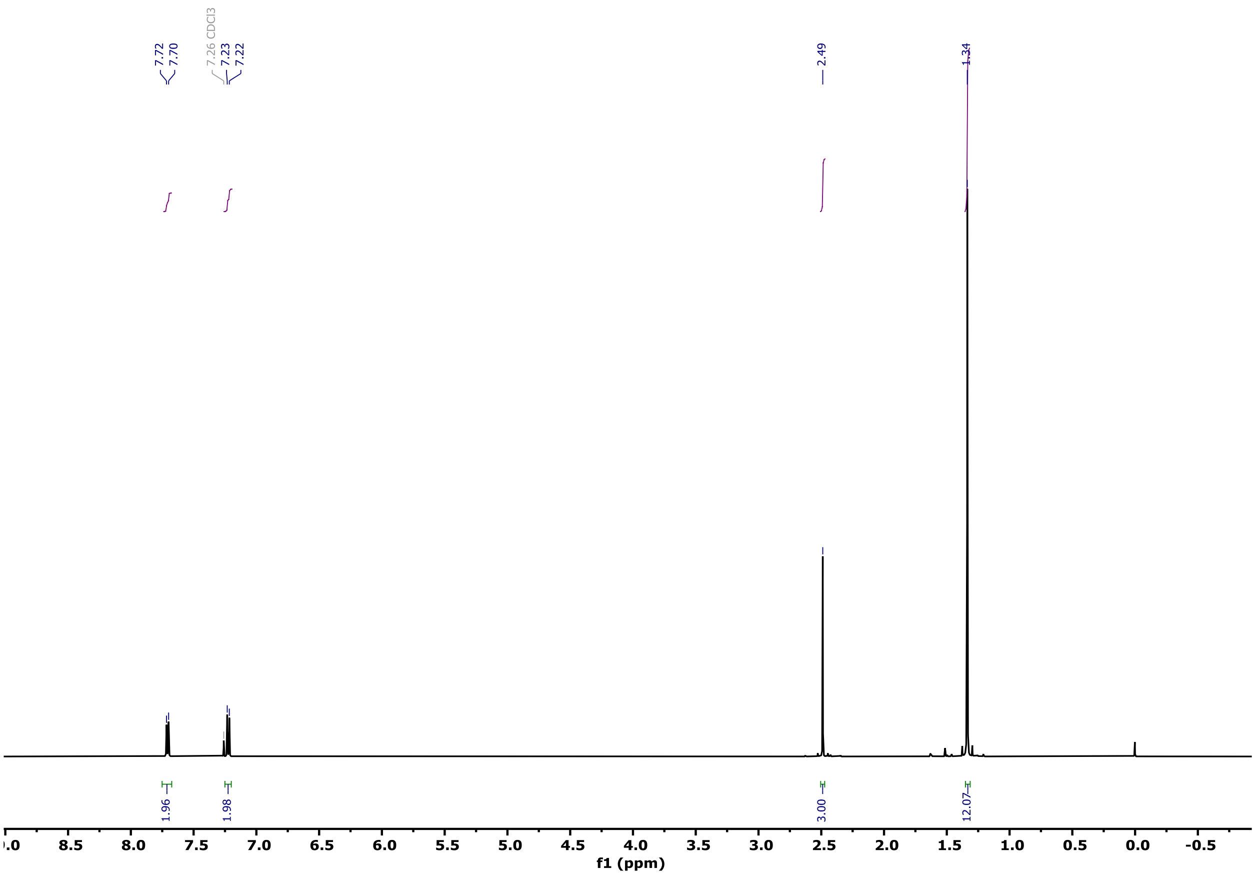

^{13}C NMR (126 MHz, $CDCl_3$)- 4,4,5,5-tetramethyl-2-(4-(methylthio)phenyl)-1,3,2-dioxaborolane (18a)

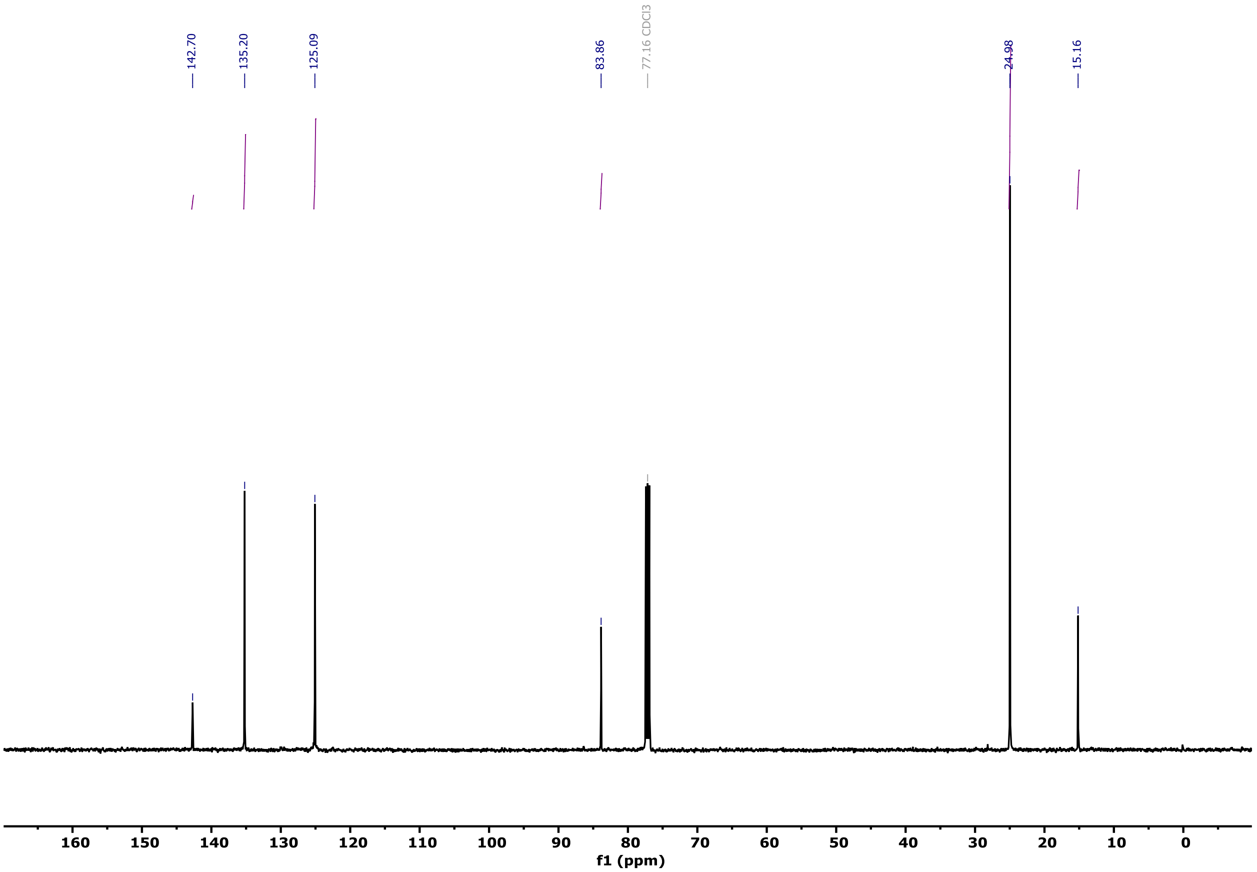

9. ^{1}H NMR (500 MHz, $CDCl_3$)- Methyl 4-(methylthio)benzoate (16a)

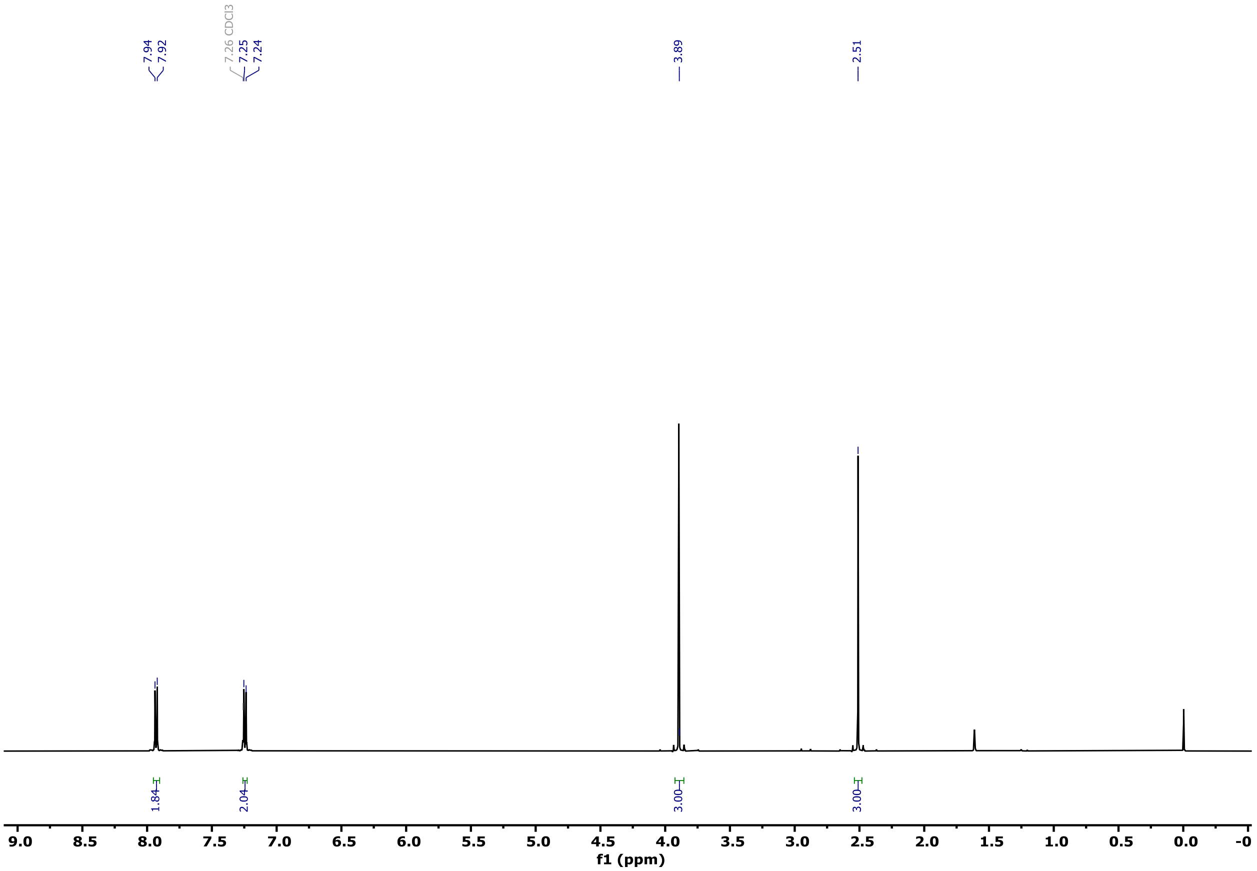

^{13}C NMR (126 MHz, $CDCl_3$)- Methyl 4-(methylthio)benzoate (16a)

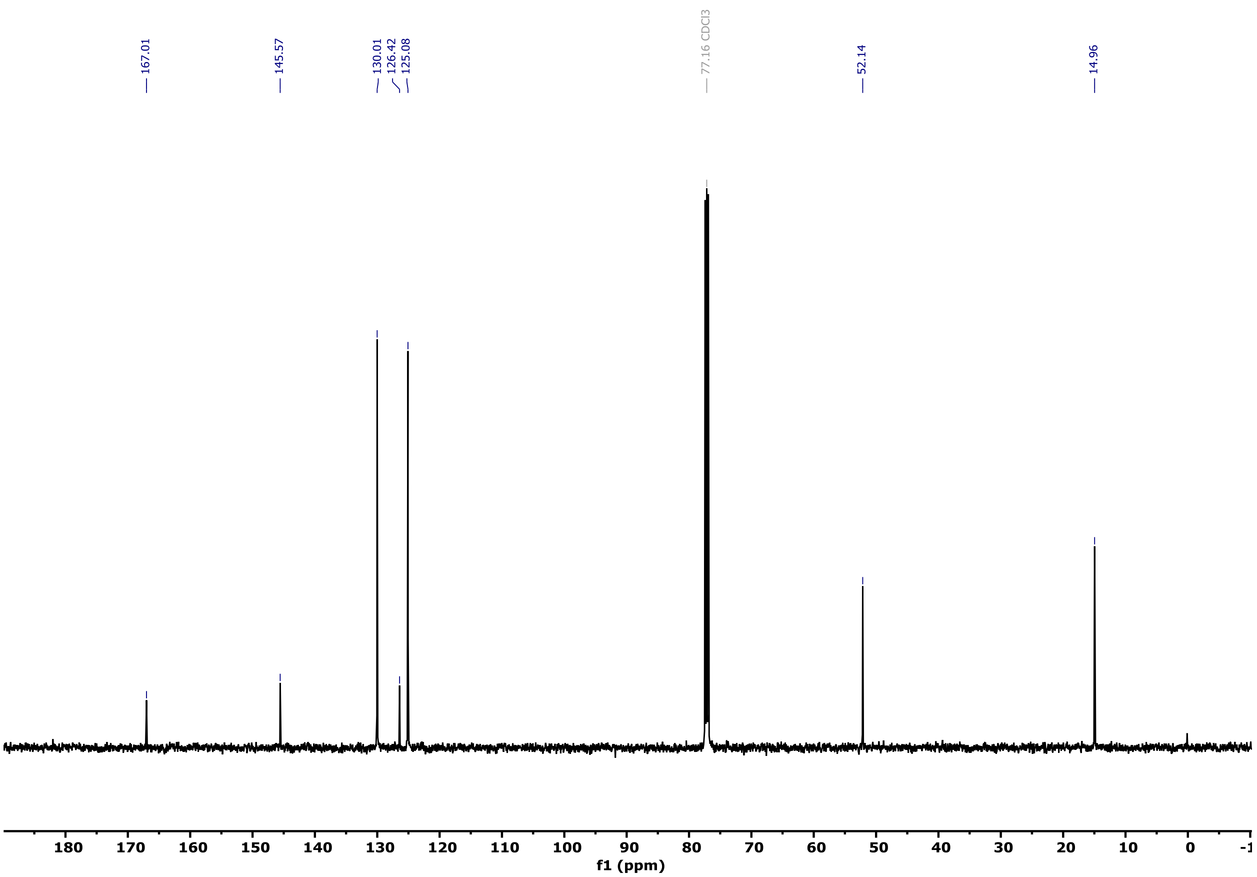

10. ^{1}H NMR (500 MHz, CDCl$_3$)- 6-(methylthio)hexanenitrile (29a)

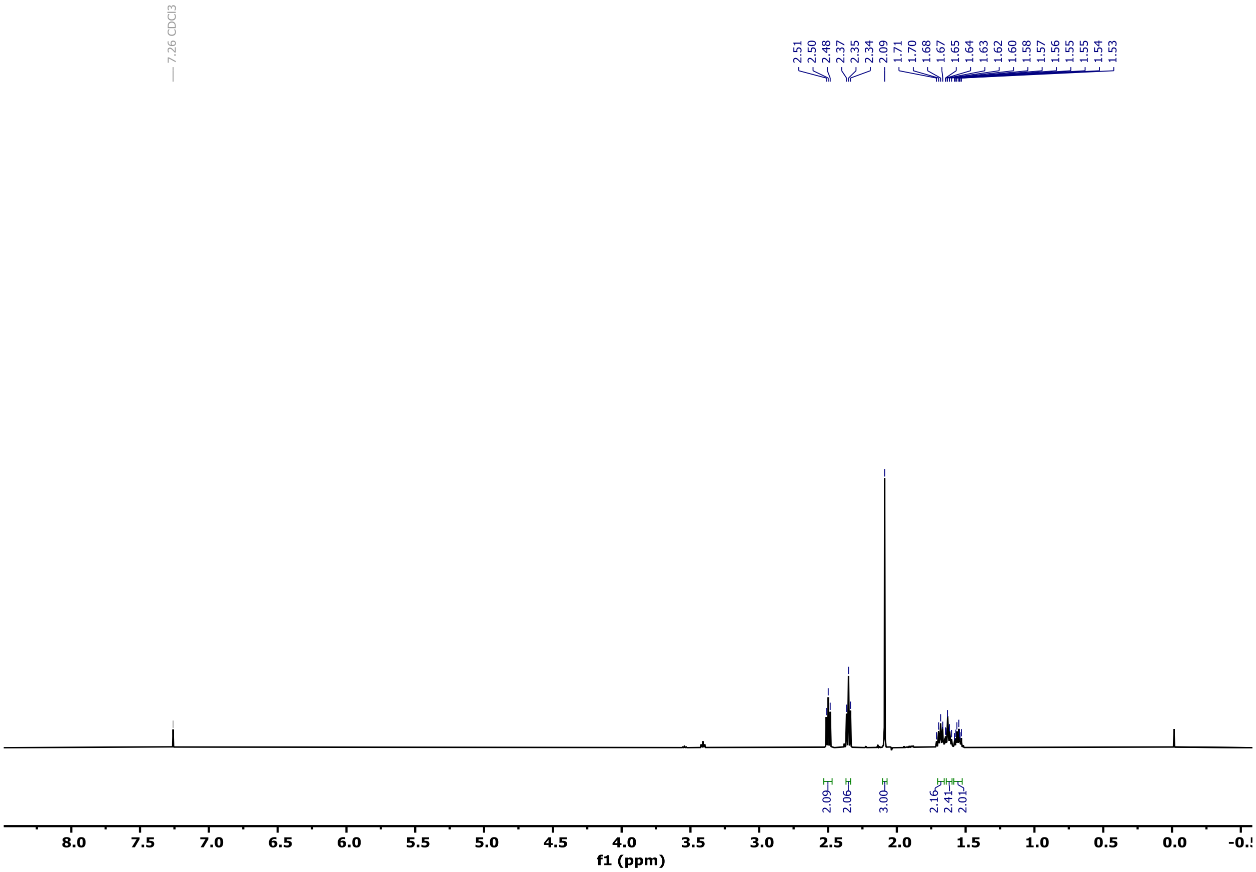

^{13}C NMR (126 MHz, $CDCl_3$)- 6-(methylthio)hexanenitrile (29a)

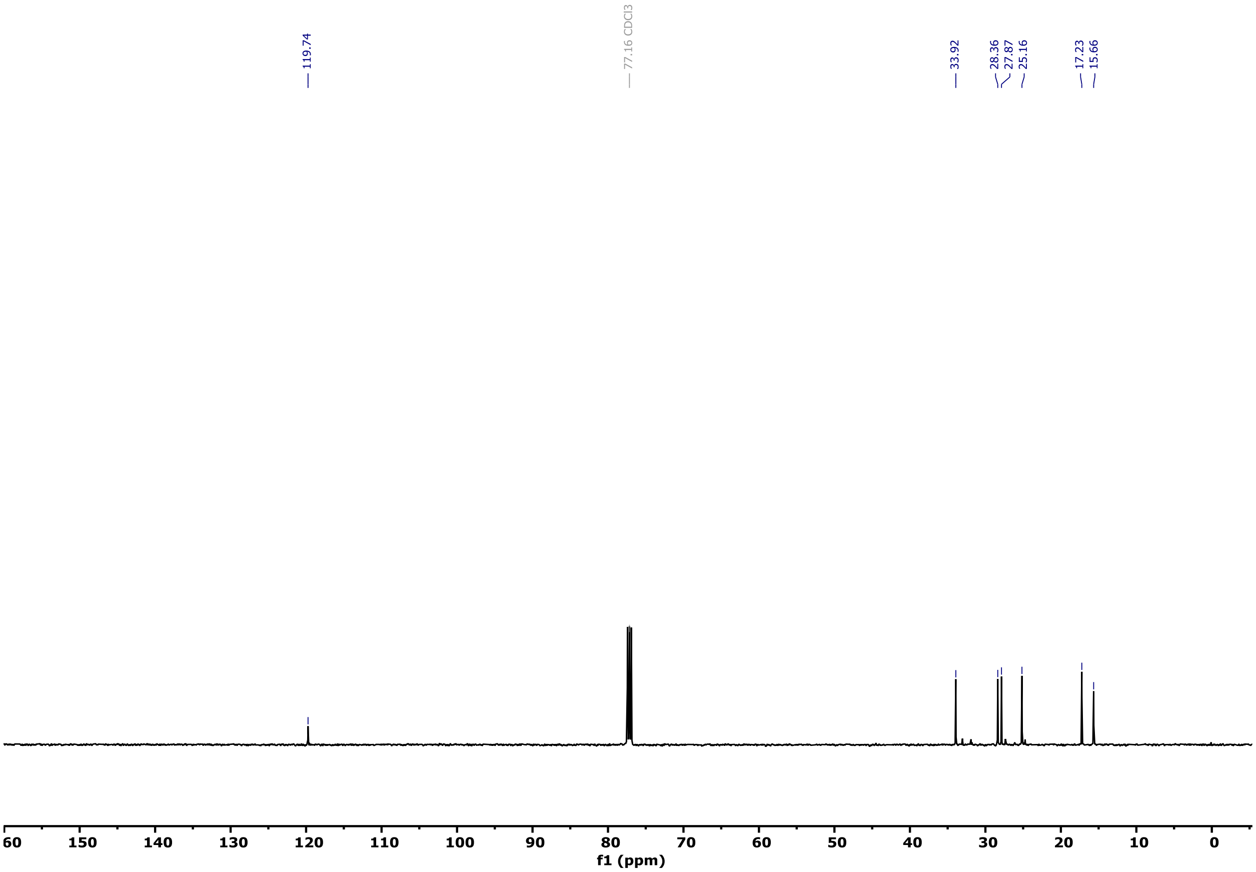

11. ^{1}H NMR (500 MHz, $CDCl_3$)- methyl(4-(trifluoromethyl)phenyl) sulfane (13a)

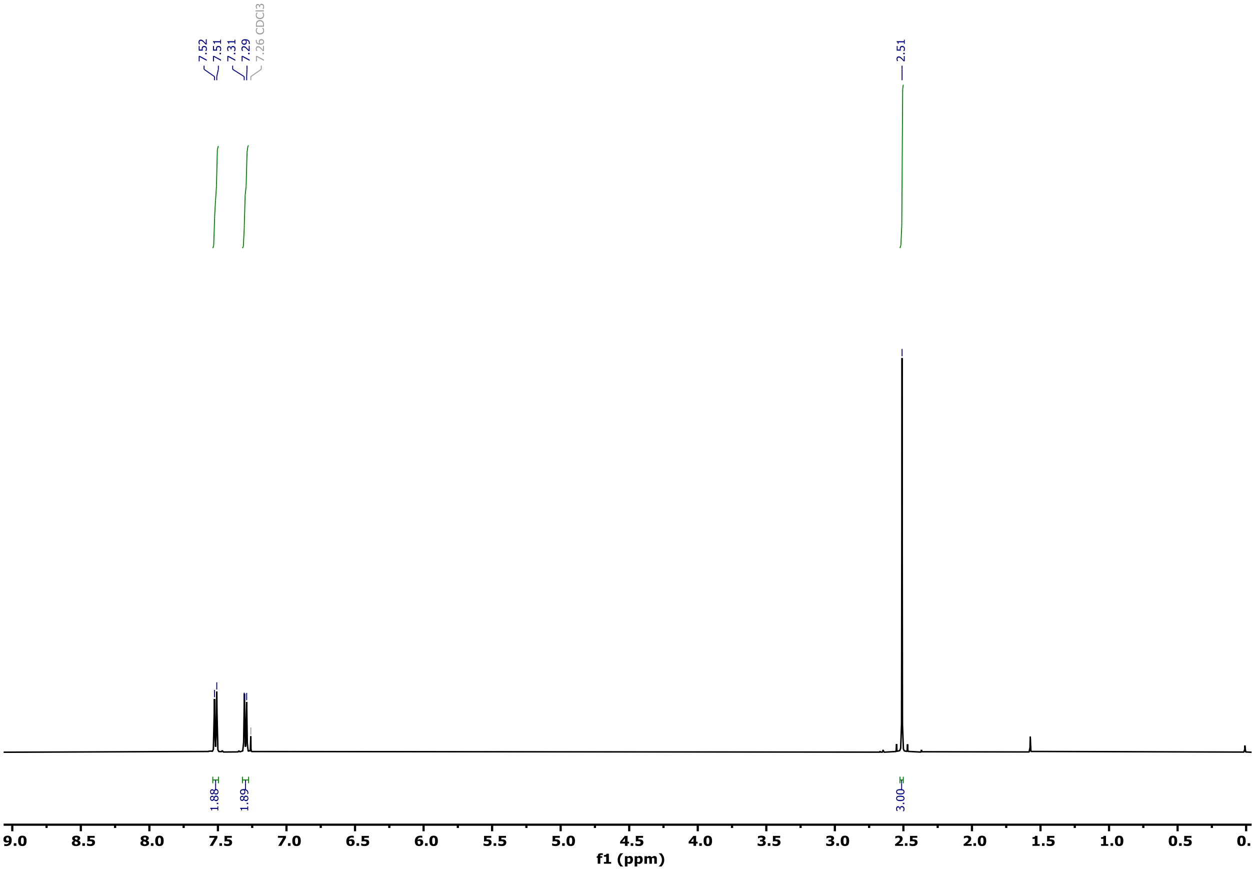

^{13}C NMR (126 MHz, $CDCl_3$)- methyl(4-(trifluoromethyl)phenyl) sulfane (13a)

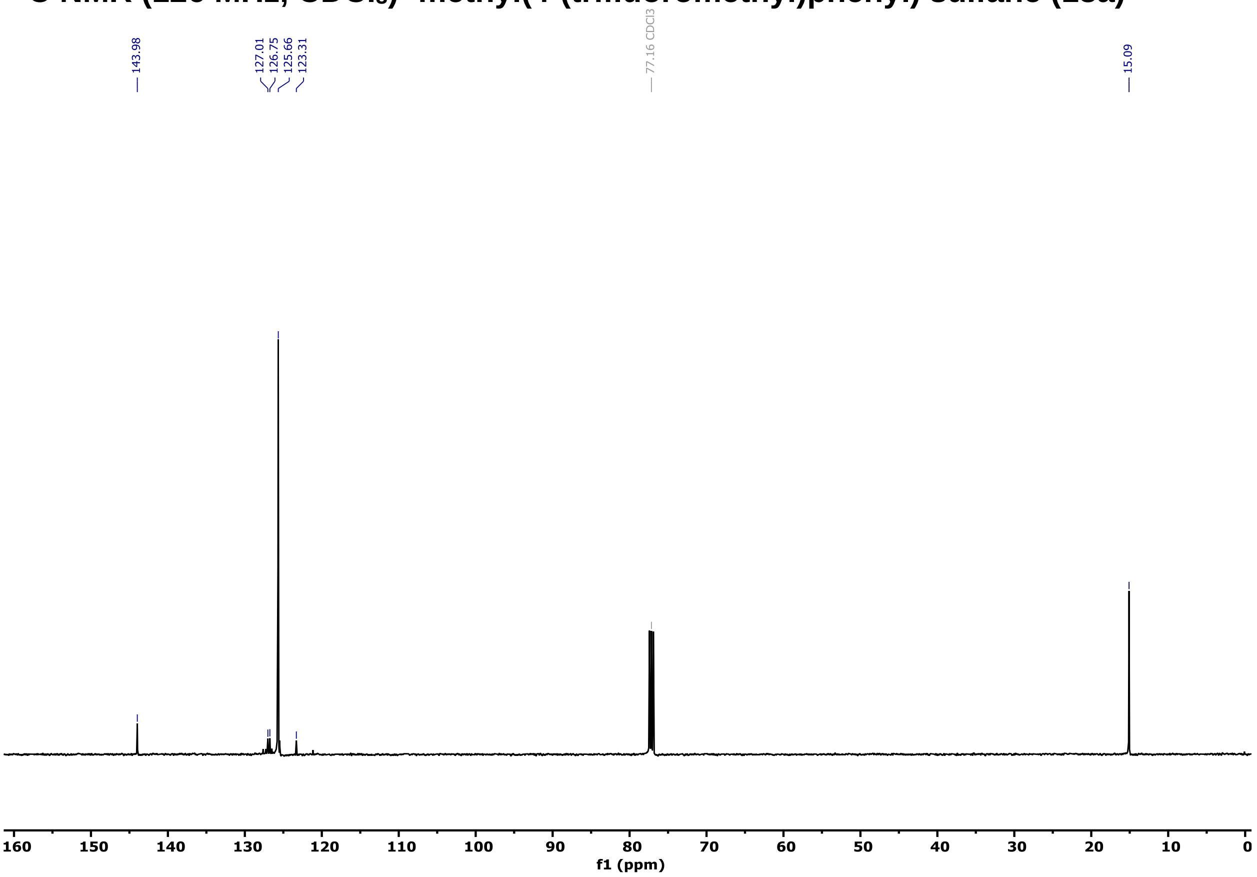

^{19}F NMR (471 MHz, $CDCl_3$)-(1methyl(4-(trifluoromethyl)phenyl) sulfane (13a)

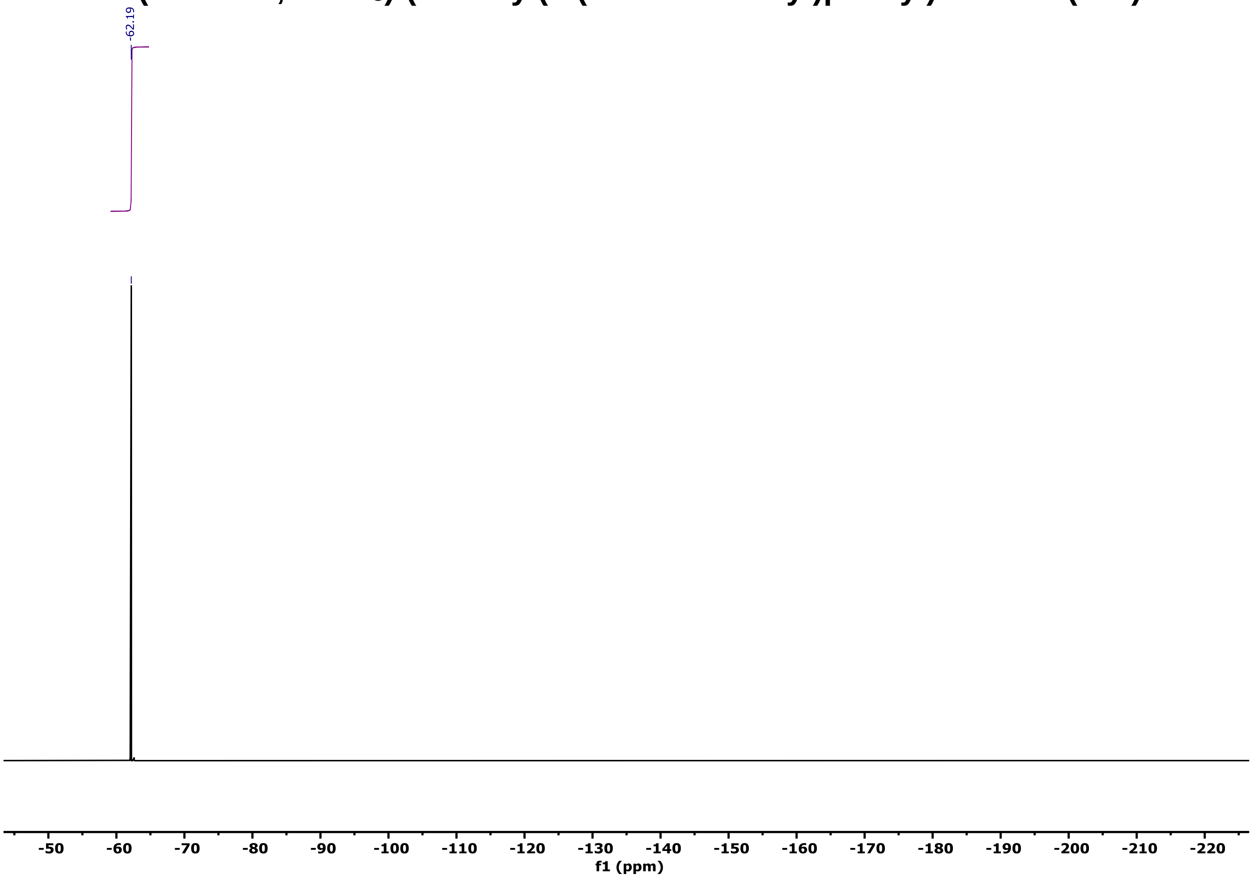

12. ^{1}H NMR (500 MHz, $CDCl_3$)- Cyclopentylmethyl sulfide (30a)

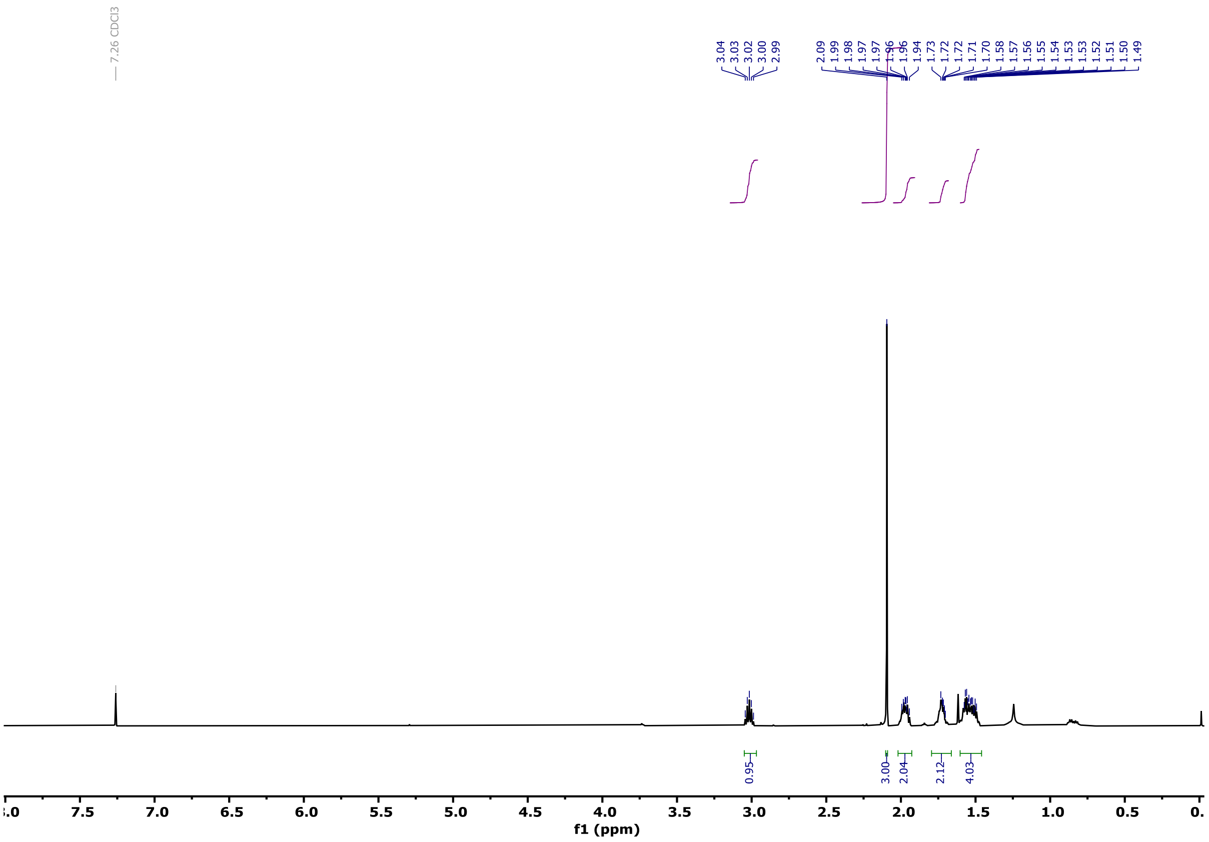

^{13}C NMR (126 MHz, $CDCl_3$)- Cyclopentylmethyl sulfide (30a)

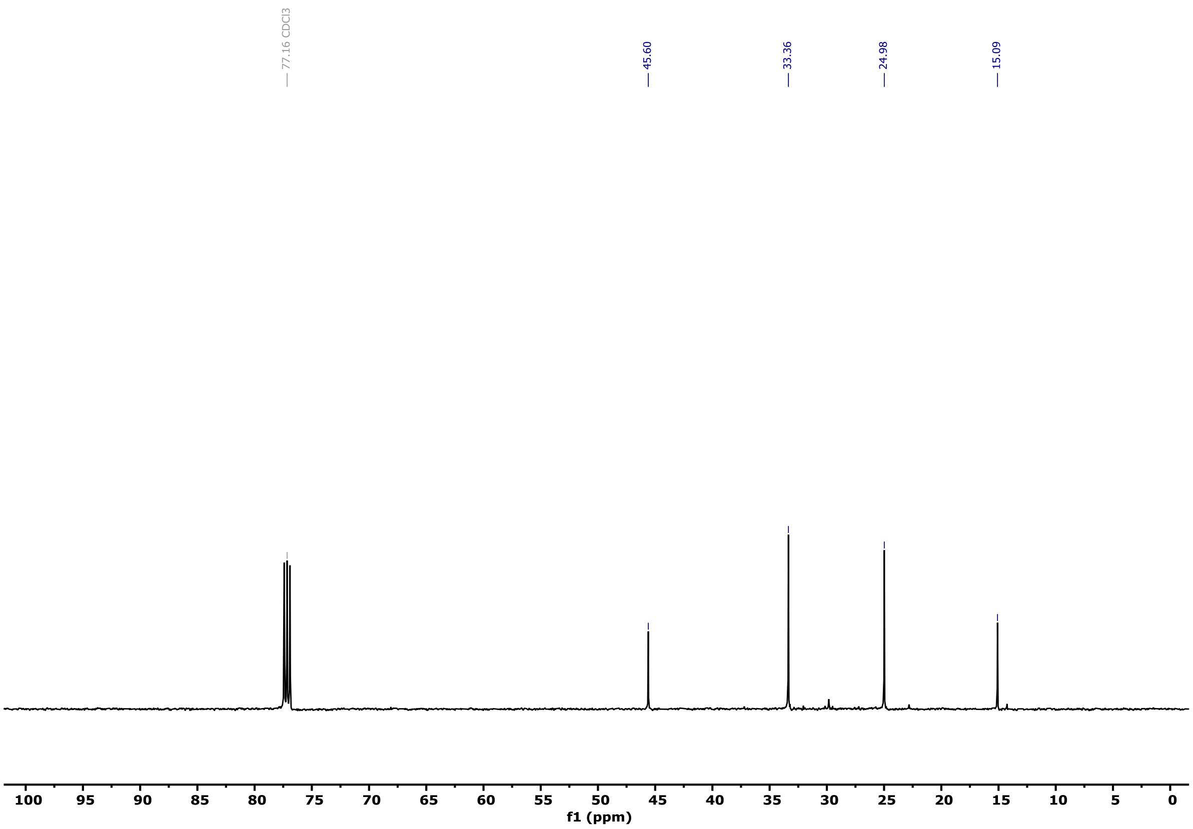

13. ^{1}H NMR (500 MHz, $CDCl_3$)- adamantan-1-yl)(methyl)sulfane (32a)

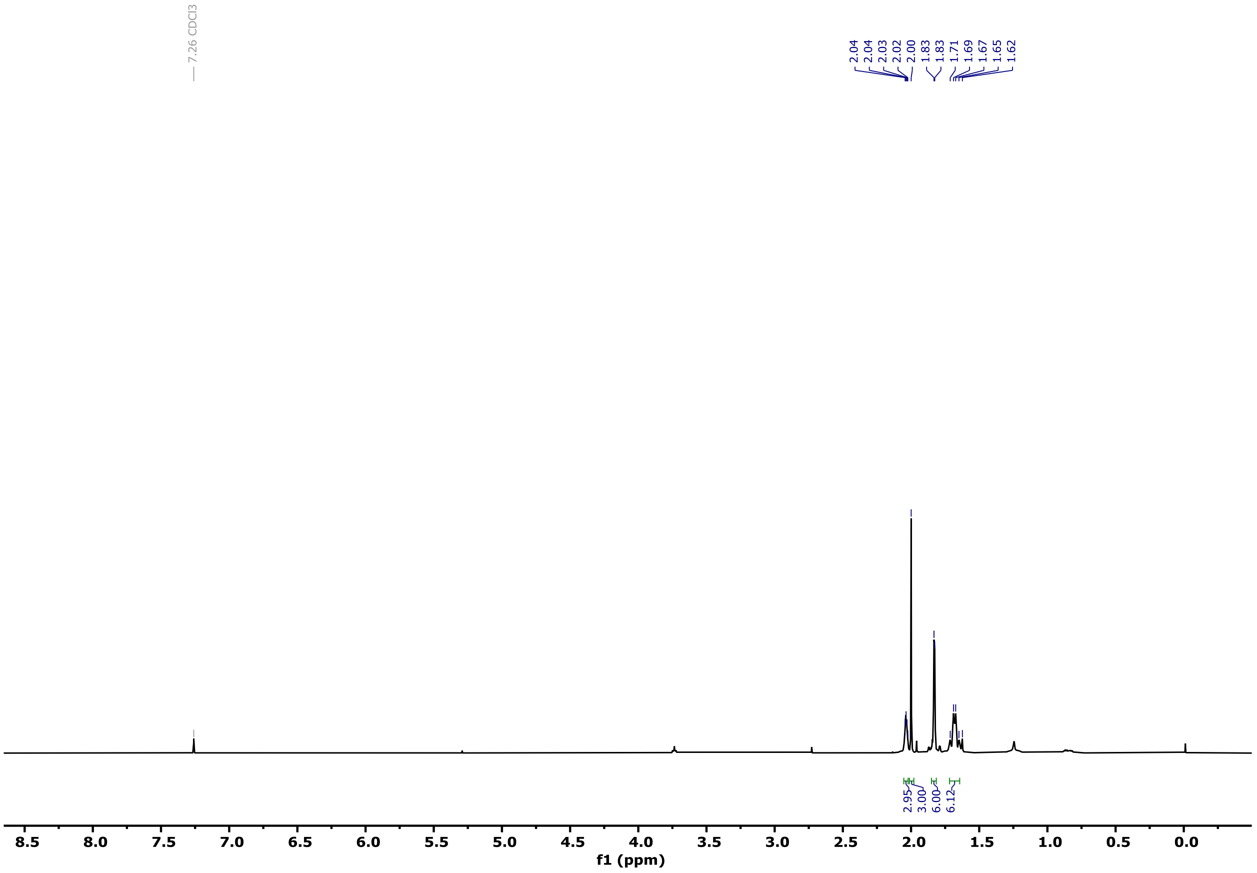

^{13}C NMR (126 MHz, $CDCl_3$)- adamantan-1-yl)(methyl)sulfane (32a)

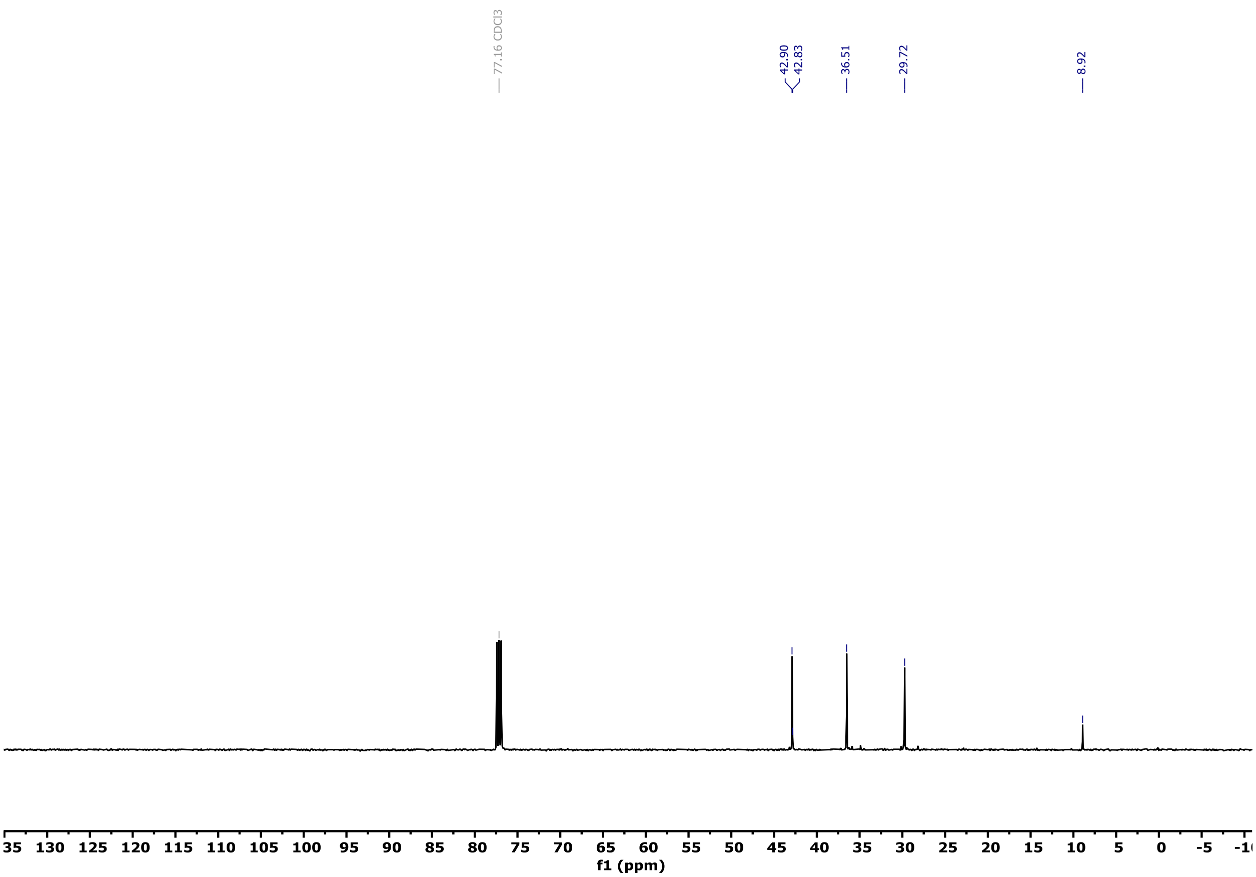

14. ^{1}H NMR (500 MHz, $CDCl_3$)- N-[4-(methylthio)phenyl]pivalamide (15a)

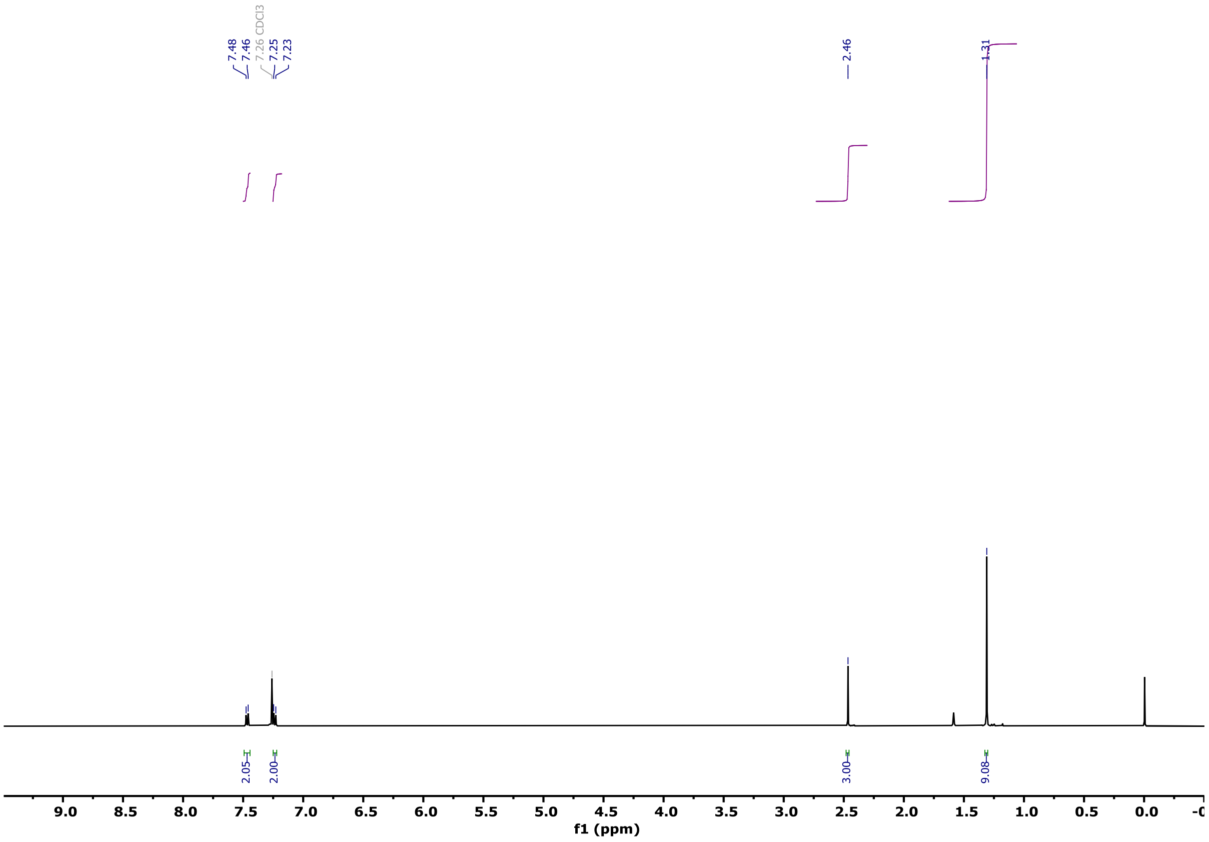

^{13}C NMR (126 MHz, $CDCl_3$)- N-[4-(methylthio)phenyl]pivalamide (15a)

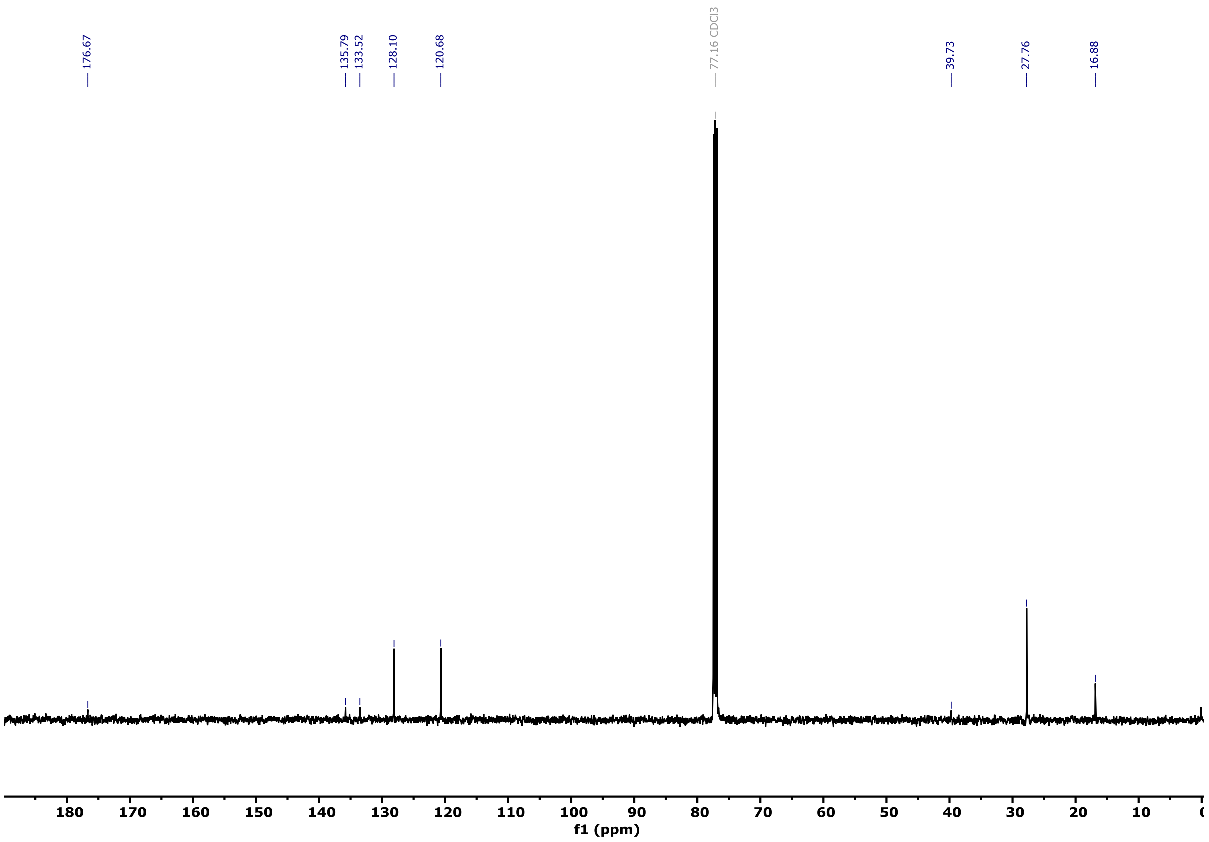

Computational methodology (XXXIII):

All density functional theory (DFT) calculations were conducted using the ORCA version 5.0.3 quantum chemical software package.[18-19] B3LYP density functional Becke, A. D., Density-functional thermochemistry. III. The role of exact exchange [20-23] with Grimme's D3 dispersion correction[24] consistent and accurate ab initio parametrization of density functional dispersion correction (DFT-D) for the 94 elements HPu. 94 with Becke-Johnson damping[25] was utilized to perform the calculations. The Ahlrichs def2-SVP[26] basis set was employed for geometry optimization. In addition, the RIJCOSX[27] approximation an improvement of the resolution of the identity approximation for the formation of the Coulomb matrix. variant was employed to expedite the calculations. To verify the characteristics of the stationary points and obtain zero-point energy (ZPE) and thermal energy corrections at 298.15K and at 1atm, harmonic vibrational frequency analysis was performed. Optimized geometries were identified as true minima on the potential energy surface (PES) with all positive vibrational frequencies and transition states were identified as first-order saddle points on the PES with one single imaginary vibrational frequency. Conductor-like polarizable continuum model (C-PCM) continuum solvation model[28] was incorporated for geometry optimization to account for the influence of the implicit solvent (water), on the computed Gibbs free energy profile. To improve the accuracy, single-point energy calculations of the optimized geometries were conducted with a larger split valence basis set def2-TZVP[26] in the present CPCM solvation model. All the reported relative Gibbs free energies in the mechanistic calculations were obtained using CPCM(H_2O)-B3LYP-D3BJ/def2-TZVP//B3LYP-D3BJ/def2-SVP.

1. **Stability of *cis* and *trans* conformer of Catalyst (A) $[Fe^{II}(L1)_2(H_2O)_2]^{2+}$ (L1 = phenanthroline):**

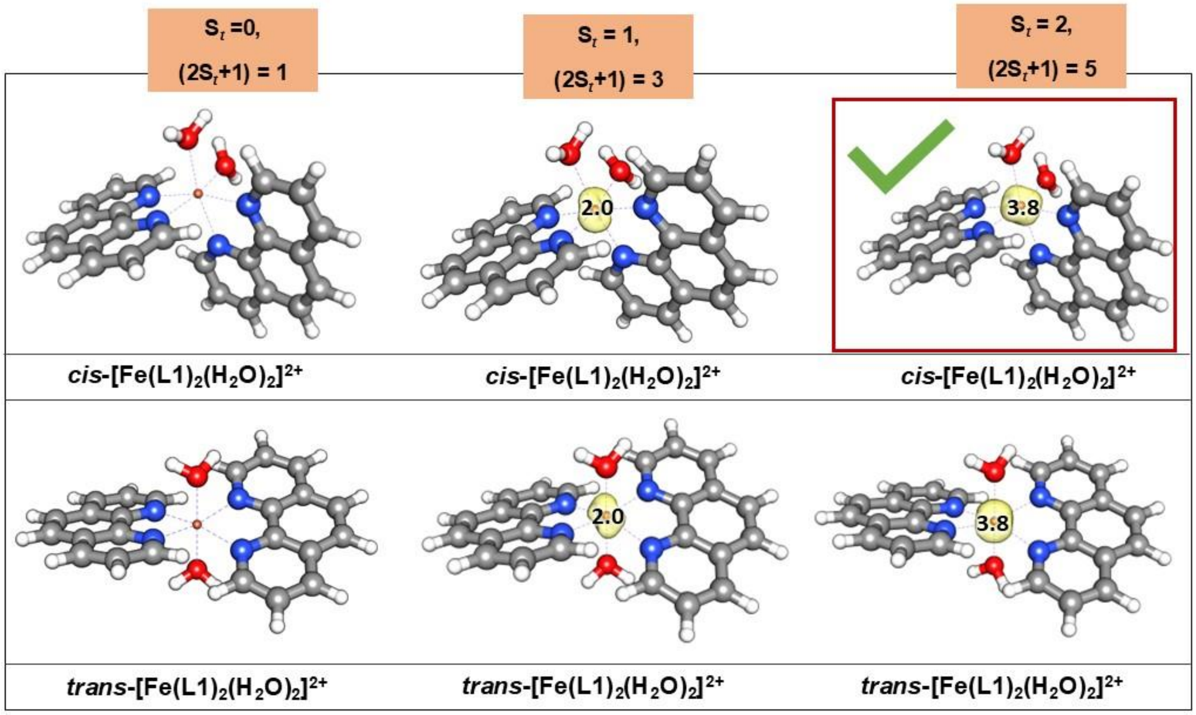

Figure S25. Optimized geometry and spin density plots of *cis* and *trans* $[Fe^{II}(L1)_2(H_2O)]^{2+}$ (**A**)

Table S24. Comparative free energy for the *cis* and *trans* conformer of precursor Fe(II) catalyst (A):

Spin state and multiplicity	Molecule	E_{tot} [au]	$G_{298.15}$ [au]	ΔG (kcal mol^{-1})
S_t =0, (2S_t+1) = 1	*cis*-$[Fe(L1)_2(H_2O)_2]^{2+}$	−2559.470322	−2559.116799	0
S_t =1, (2S_t+1) = 3		−2559.461293	−2559.116002	0.5
S_t =2, (2S_t+1) = 5		−2559.479902	−2559.137526	**−13.0**
S_t =0, (2S_t+1) = 1	*trans*-$[Fe(L1)_2(H_2O)_2]^{2+}$	−2559.450798	−2559.099577	10.8
S_t =1, (2S_t+1) = 3		−2559.454981	−2559.110512	3.9
S_t =2, (2S_t+1) = 5		−2559.471607	−2559.128857	−7.6

***cis*-$[Fe(L1)_2(H_2O)_2]^{2+}$** complex with high spin Fe^{II} (S_t = 2) having **quintet spin multiplicity** is the most thermodynamically favourable form.

2. Stability of possible [$Fe^{III}(L1)_2$(NH−*t*BuCOO)]$^{2+}$ (Int 1) species:

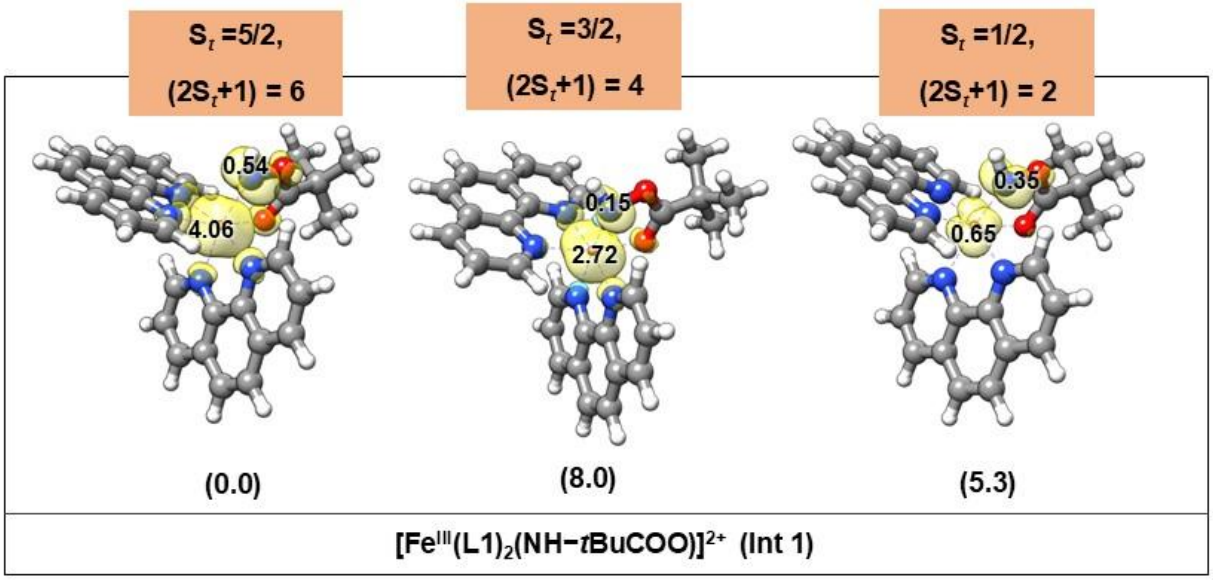

Figure S26. Optimized geometry and spin density plots [$Fe^{III}(L1)_2$(NH−*t*BuCOO)]$^{2+}$ (Int 1) species.

Table S25. Comparative free energy for the (Int 1) [$Fe^{III}(L1)_2$(NH−*t*BuCOO)]$^{2+}$ species:

Spin state and spin multiplicity	Molecule	E_{tot} [au]	$G_{298.15}$ [au]	∆G (kcal mol^{-1})
S_t =5/2, (2S_t+1) = 6	[$Fe^{III}(L1)_2$(NH−*t*BuCOO)]$^{2+}$	−2808.255785	−2807.814291	0
S_t =3/2, (2S_t+1) = 4		−2808.244820	−2807.801317	8.1
S_t =1/2, (2S_t+1) = 2		−2808.253788	−2807.805766	5.3

3. Optimized geometry and spin density plot of $[Fe^{III}(L1)NH\bullet]^{2+}$ (Int 5) species for all possible spin states:

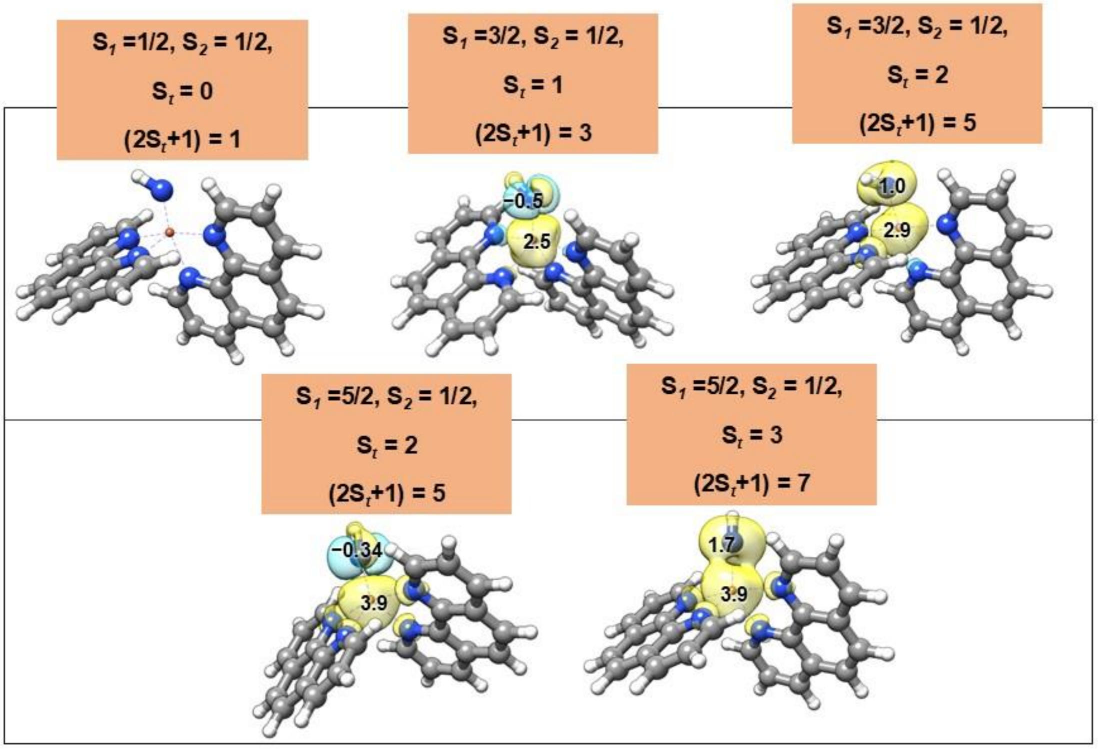

$[Fe^{III}(L1)NH\bullet]^{2+}$ (Int 5)

Figure S27. Optimized geometry and spin density plots $[Fe^{III}(L1)_2(NH)\bullet]^{2+}$ (Int 5) species.

Table S26. Comparative free energy for the $[Fe^{III}(L1)NH\bullet]^{2+}$ (Int 5):

Spin state	Molecule	E_{tot} [au]	$G_{298.15}$ [au]	ΔG (kcal mol⁻¹)
S_1 =1/2, S_2 = 1/2, S_t = 0 $(2S_t+1)$ = 1		−2461.81839	−2461.501391	0
S_1 =3/2, S_2 = 1/2, S_t = 1 $(2S_t+1)$ = 3		−2461.83459	−2461.521949	**−12.9**
S_1 =3/2, S_2 = 1/2, S_t = 2 $(2S_t+1)$ = 5	**$[Fe^{III}(L1)NH\bullet]^{2+}$**	−2461.82848	−2461.517643	−10.2
S_1 =5/2, S_2 = 1/2, S_t = 2 $(2S_t+1)$ = 5		−2461.81885	−2461.510414	−5.6
S_1 =5/2, S_2 = 1/2, S_t = 3 $(2S_t+1)$ = 7		−2461.80925	−2461.50079	0.38

Based on DFT calculation, it can be inferred that **$[Fe^{III}(L1)NH\bullet]^{2+}$ (Int 5)** species with an overall triplet spin multiplicity have the lowest relative free energy as compared to all spin states. However, we are unable to locate the transition state for −*NH* transfer on the triplet spin multiplicity and so for NH transfer reaction an overall quintet spin multiplicity was considered.

Table S27. Mulliken atomic charges, Mulliken and Lowedin spin population for all possible spin states for $[Fe(L1)NH\bullet]^{2+}$ species are mentioned below:

	Properties	Fe	N(NH)
($2S_t+1=1$) $[Fe^{III}(L1)NH\bullet]^{2+}$ (Int 5)	Mulliken spin populations	0.0	0.0
	Loewdin spin populations	0.0	0.0
	Mulliken atomic charges	0.588	−0.218
($2S_t+1=3$) $[Fe^{III}(L1)NH\bullet]^{2+}$ (Int 5)	Mulliken spin populations	2.515	−0.558
	Loewdin spin populations	2.403	−0.470
	Mulliken atomic charges	0.683	−0.253
($2S_t+1_a=5$) $[Fe^{III}(L1)NH\bullet]^{2+}$ (Int 5)	Mulliken spin populations	2.94	1.007
	Loewdin spin populations	2.90	0.97
	Mulliken atomic charges	0.319	−0.439
($2S_t+1_b=5$) $[Fe^{III}(L1)NH\bullet]^{2+}$ (Int 5)	Mulliken spin populations	3.983	−0.340
	Loewdin spin populations	3.987	−0.285
	Mulliken atomic charges	0.907	−0.266
($2S_t+1=7$) $[Fe^{III}(L1)NH\bullet]^{2+}$ (Int 5)	Mulliken spin populations	3.997	1.760
	Loewdin spin populations	3.957	1.698
	Mulliken atomic charges	0.913	−0.175

Table S28. Comparison of $[Fe^{III}(L1)_2(NH-tBuCOO)]^{2+}$ (Int 1) and $[Fe^{III}(L1)NH•]^{2+}$ (Int 5):

Complex	Properties	Fe	−N(NH)	Fe−N^{NH} (in Å)
**$[Fe^{III}(L1)_2(NH-tBuCOO)]^{2+}$** **(Int 1)** Lowest energy Fe(III) High spin (S=5/2)	Mulliken spin populations	4.066	0.546	1.97
	Loewdin spin populations	4.066	0.546	
	Mulliken atomic charges	**0.836**	**−0.127**	
**$[FeNH•(L1)_2]^{2+}$** **(Int 5)** Lowest energy Fe(III) Intermediate spin (S=3/2)	Mulliken spin populations	2.94	1.007	1.71
	Loewdin spin populations	2.90	0.97	
	Mulliken atomic charges	**0.319**	**−0.439**	

4. Electronic energies and Gibbs free energies

Total electronic energies and Gibbs free energies (298.15 K) of the optimized structures at CPCM(H_2O)-B3LYP/def2-TZVP//B3LYP/def2-SVP are given below.

**Molecule**	**E_{tot} [au]**	**$G_{298.15}$ [au]**	**Imaginary frequency**
(S=1) cis-$[Fe(Phen)_2(H_2O)_2]^{2+}$	−2559.470322	−2559.116799	
(S=3) cis-$[Fe(Phen)_2(H_2O)_2]^{2+}$	−2559.461293	−2559.116002	
(S=5) cis-$[Fe(Phen)_2(H_2O)_2]^{2+}$	−2559.479902	−2559.137526	
(S=1) trans-$[Fe(Phen)_2(H_2O)_2]^{2+}$	−2559.450798	−2559.099577	
(S=3) trans-$[Fe(Phen)_2(H_2O)_2]^{2+}$	−2559.454981	−2559.110512	
(S=5) trans-$[Fe(Phen)_2(H_2O)_2]^{2+}$	−2559.471607	−2559.128857	
$PhSCH_3NH$	−724.9735627	−724.8635621	
$[Fe(Phen)_2]^{2+}$	−2406.557655	−2406.258819	
$PhSCH_3$	−669.6754904	−669.5785501	
H_2O	−76.43721041	−76.43384099	
t-$BuCOONH_2$ HOTf	−1364.397195	−1364.240247	
TfOH	−962.0934426	−962.087354	
NH_4OTf	−1018.707687	−1018.665869	
CO_2	−347.0000841	−346.8861509	
Isoprene	−188.5919284	−188.5983725	
Int 0	−3923.877097	−5287.617646	
Int 1	−3154.599013	−3154.042644	
Int 1(S=6)	−2808.255785	−2807.814291	
Int 1(S=4)	−2808.244820	−2807.801317	
Int 1(S=2)	−2808.253788	−2807.805766	
$TS_{1\rightarrow2}$	−3154.569039	−3154.00988	*i975.65*
Int 2	−3154.578407	−3154.014422	

Int 3	−2807.574491	−2807.146644	
$TS_{3\rightarrow4}$	−2807.544129	−2807.119898	*i496.29*
Int 4	−2807.627859	−2807.207583	
Int 5 (S=1)	−2461.81839	−2461.501391	
Int 5 (S=3)	−2461.83459	−2461.521949	
Int 5 (S_a=5)	−2461.82848	−2461.517643	
Int 5 (S_b=5)	−2461.81885	−2461.510414	
Int 5(S=7)	−2461.80925	−2461.50079	
Int 6	−3131.53952	−3131.104558	
$TS_{6\rightarrow7}$	−3131.524325	−3131.091309	*i109.35*
Int 7	−3131.594501	−3131.158227	
Int 8	−724.9735627	−724.8635621	
Int 9	−1763.575091	−1763.412919	
Product	−1763.604142	−1763.439791	

5. Cartesian Coordinates of the Optimized Structures

DFT optimization method: CPCM(H_2O)-B3LYP-D3BJ/def2-TZVP//B3LYP-D3BJ/def2-SVP

(S=1) *cis*-$[Fe(Phen)_2(H_2O)_2]^{2+}$

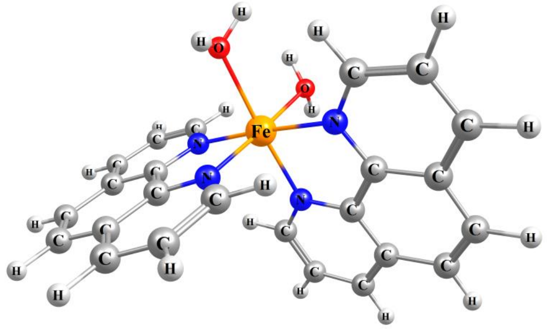

Fe	-0.109532000	-0.401651000	-0.022678000
N	1.257442000	-1.060817000	1.314734000
N	-0.498573000	1.250126000	1.077857000
N	1.552614000	0.347180000	-0.918884000
N	-1.719577000	-1.109469000	0.994342000

C	2.687448000	0.057331000	-0.224599000
C	2.527248000	-0.692742000	0.985487000
C	-1.565740000	1.081476000	1.908031000
C	-2.227633000	-0.188272000	1.858382000
C	-2.308240000	-2.298701000	0.909742000
C	-3.431661000	-2.638447000	1.688633000
C	-3.952248000	-1.714586000	2.578436000
C	-3.347747000	-0.439246000	2.684256000
C	0.144691000	2.414958000	1.094212000
C	-0.241338000	3.474672000	1.938150000
C	-1.325061000	3.316645000	2.784834000
C	-2.027186000	2.088264000	2.787676000
C	1.656073000	1.029244000	-2.055435000
C	1.064227000	-1.758539000	2.431113000
C	2.896446000	1.468388000	-2.558144000
C	4.056770000	1.190645000	-1.855937000
C	3.975343000	0.462997000	-0.644804000
C	2.126739000	-2.129909000	3.278034000
C	3.422030000	-1.765283000	2.953029000
C	3.655745000	-1.022187000	1.771526000
C	-3.166535000	1.810141000	3.620551000
C	-3.800179000	0.599018000	3.570612000
C	5.108908000	0.117223000	0.170054000
C	4.955284000	-0.592440000	1.329135000
H	0.728839000	1.244610000	-2.589044000
H	2.921982000	2.025920000	-3.495737000
H	5.030647000	1.524287000	-2.222588000
H	6.100157000	0.436726000	-0.159666000
H	5.821387000	-0.849882000	1.942905000
H	4.262524000	-2.042520000	3.593466000
H	1.909928000	-2.703049000	4.181070000
H	0.037302000	-2.038520000	2.671359000
H	-1.644373000	4.123518000	3.448332000
H	-3.515326000	2.593343000	4.297072000
H	-4.665074000	0.396654000	4.206430000
H	0.323185000	4.408078000	1.909972000

H	0.995909000	2.523875000	0.419853000
H	-4.819558000	-1.955347000	3.197936000
H	-3.872854000	-3.630368000	1.580210000
H	-1.878749000	-3.015787000	0.208417000
O	0.181796000	-2.194133000	-1.199631000
H	0.438717000	-2.971597000	-0.676720000
H	-0.559295000	-2.493762000	-1.751389000
O	-1.488129000	0.317580000	-1.531381000
H	-1.526290000	1.284778000	-1.611408000
H	-1.369391000	-0.008068000	-2.438802000

(S=3) *cis*-$[Fe(Phen)_2(H_2O)_2]^{2+}$

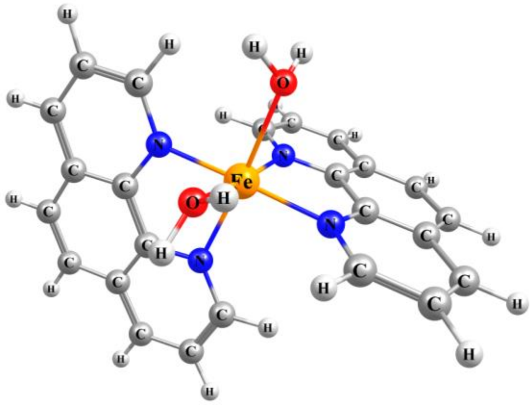

Fe	-0.090036000	-0.404999000	0.022986000
N	1.335499000	-1.190419000	1.371759000
N	-0.527841000	1.366390000	1.091291000
N	1.555507000	0.297579000	-0.837160000
N	-1.674692000	-1.049649000	1.030548000
C	2.707088000	-0.016110000	-0.180673000
C	2.586812000	-0.808216000	1.008738000
C	-1.614313000	1.179158000	1.883678000
C	-2.231995000	-0.115214000	1.849777000
C	-2.209346000	-2.267867000	0.986791000
C	-3.335515000	-2.621990000	1.752815000
C	-3.917139000	-1.682050000	2.586159000
C	-3.364229000	-0.381461000	2.655945000
C	0.080915000	2.546493000	1.097036000
C	-0.365742000	3.616946000	1.896685000
C	-1.474381000	3.442178000	2.708287000

C	-2.137983000	2.191999000	2.722339000
C	1.627054000	1.032541000	-1.944465000
C	1.176073000	-1.916467000	2.472258000
C	2.851355000	1.497375000	-2.459826000
C	4.030129000	1.191195000	-1.801441000
C	3.981760000	0.412624000	-0.620857000
C	2.264369000	-2.311114000	3.275017000
C	3.547082000	-1.932587000	2.915184000
C	3.742630000	-1.155430000	1.748658000
C	-3.293078000	1.900360000	3.527520000
C	-3.880753000	0.666114000	3.495221000
C	5.141448000	0.042741000	0.145564000
C	5.027205000	-0.707657000	1.282954000
H	0.682467000	1.271452000	-2.435115000
H	2.850397000	2.097061000	-3.371377000
H	4.993580000	1.542984000	-2.178067000
H	6.120080000	0.379740000	-0.203670000
H	5.912618000	-0.982235000	1.860761000
H	4.409291000	-2.224526000	3.519739000
H	2.080982000	-2.909093000	4.169201000
H	0.154681000	-2.200720000	2.736695000
H	-1.842938000	4.255194000	3.338575000
H	-3.689970000	2.690093000	4.169369000
H	-4.756212000	0.449993000	4.111619000
H	0.167918000	4.568199000	1.865123000
H	0.953536000	2.658330000	0.448792000
H	-4.791252000	-1.931030000	3.192567000
H	-3.731265000	-3.636107000	1.678769000
H	-1.726212000	-2.993287000	0.330337000
O	0.052352000	-2.126599000	-1.289487000
H	0.625996000	-2.855688000	-1.002258000
H	-0.733423000	-2.552697000	-1.667302000
O	-1.462852000	0.261806000	-1.544893000
H	-1.763299000	1.184172000	-1.501445000
H	-1.265266000	0.102851000	-2.481722000

(S=5) *cis*-$[Fe(Phen)_2(H_2O)_2]^{2+}$

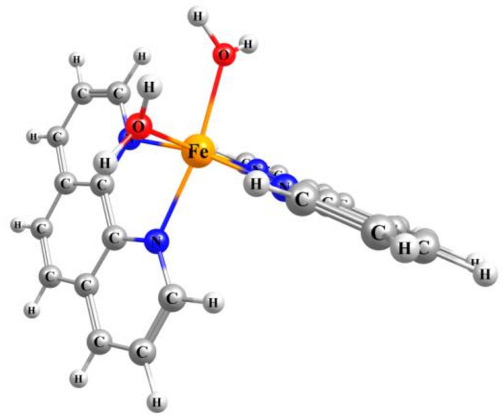

Fe	-0.179159000	-0.468485000	-0.188134000
N	1.325411000	-1.183872000	1.242261000
N	-0.626201000	1.315740000	1.010896000
N	1.673145000	0.358629000	-0.975619000
N	-1.841209000	-1.121338000	1.051658000
C	2.764684000	0.056649000	-0.227447000
C	2.580100000	-0.754987000	0.951035000
C	-1.642773000	1.121801000	1.889620000
C	-2.288252000	-0.168489000	1.908282000
C	-2.415256000	-2.320024000	1.046767000
C	-3.481618000	-2.648131000	1.904054000
C	-3.950794000	-1.691811000	2.787643000
C	-3.355153000	-0.408404000	2.811530000
C	-0.016841000	2.495176000	0.978406000
C	-0.385214000	3.562619000	1.820184000
C	-1.419497000	3.383470000	2.721283000
C	-2.084016000	2.135291000	2.778523000
C	1.813850000	1.102556000	-2.068079000
C	1.133204000	-1.931852000	2.322575000
C	3.062378000	1.597598000	-2.487550000
C	4.187338000	1.305146000	-1.736209000
C	4.062780000	0.515977000	-0.568376000
C	2.182976000	-2.304915000	3.183981000
C	3.468446000	-1.878054000	2.902577000
C	3.701005000	-1.079070000	1.758047000
C	-3.167717000	1.861401000	3.682159000
C	-3.778763000	0.639856000	3.698239000

C	5.177344000	0.168651000	0.269363000
C	5.002831000	-0.595945000	1.387548000
H	0.903013000	1.321915000	-2.629763000
H	3.124171000	2.204222000	-3.392429000
H	5.172237000	1.675825000	-2.030969000
H	6.168735000	0.532293000	-0.010300000
H	5.851850000	-0.857592000	2.023162000
H	4.306529000	-2.148172000	3.549641000
H	1.967575000	-2.922345000	4.057625000
H	0.108878000	-2.254547000	2.524655000
H	-1.730842000	4.189517000	3.390191000
H	-3.491056000	2.656532000	4.357817000
H	-4.602005000	0.435098000	4.386364000
H	0.149235000	4.511447000	1.750105000
H	0.799117000	2.611483000	0.260556000
H	-4.775010000	-1.913235000	3.470207000
H	-3.918594000	-3.647072000	1.862788000
H	-2.010941000	-3.051410000	0.343230000
O	-0.104367000	-2.316541000	-1.248872000
H	0.410661000	-3.075641000	-0.931214000
H	-0.815037000	-2.682796000	-1.799518000
O	-1.336495000	0.292622000	-1.808359000
H	-1.677383000	1.201700000	-1.810995000
H	-1.224207000	0.043872000	-2.740136000

(S=1) *trans*-$[Fe(Phen)_2(H_2O)_2]^{2+}$

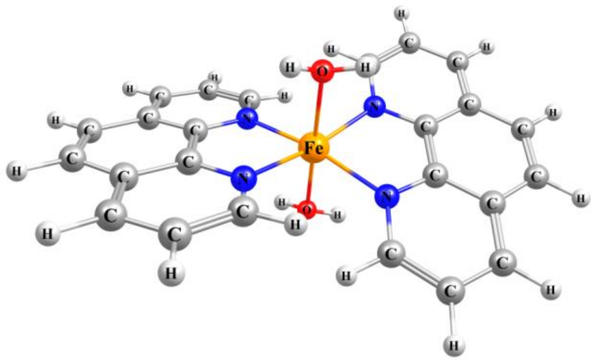

Fe	0.034489000	-0.307845000	-0.034710000
N	1.593464000	-1.517206000	0.597949000
N	-1.575143000	0.557358000	-1.015695000
N	1.632548000	0.702129000	-0.890826000
N	-1.508082000	-0.969908000	1.171015000
C	2.833596000	0.123689000	-0.608510000
C	2.809843000	-1.083448000	0.164016000
C	-2.769751000	0.262719000	-0.431545000
C	-2.730666000	-0.528018000	0.763147000
C	-1.428016000	-1.596556000	2.342324000
C	-2.558205000	-1.892959000	3.127176000
C	-3.813385000	-1.517658000	2.683226000
C	-3.927337000	-0.801325000	1.469251000
C	-1.590677000	1.172996000	-2.195120000
C	-2.779208000	1.603570000	-2.814690000
C	-3.993090000	1.379683000	-2.190324000
C	-4.015177000	0.678371000	-0.962407000
C	1.635617000	1.884192000	-1.503398000
C	1.531295000	-2.696733000	1.211902000
C	2.816300000	2.516443000	-1.936786000
C	4.036213000	1.902416000	-1.718800000
C	4.071960000	0.670241000	-1.025308000
C	2.672751000	-3.467979000	1.500603000
C	3.920597000	-3.001003000	1.129662000
C	4.016348000	-1.776891000	0.427776000
C	-5.219448000	0.361640000	-0.244285000
C	-5.176208000	-0.337247000	0.929540000
C	5.285510000	-0.036522000	-0.719322000

C	5.257909000	-1.217112000	-0.031459000
H	0.672760000	2.371998000	-1.653649000
H	2.746885000	3.480474000	-2.442970000
H	4.968315000	2.360296000	-2.058405000
H	6.231990000	0.392055000	-1.056391000
H	6.181327000	-1.757372000	0.188813000
H	4.826685000	-3.568510000	1.354922000
H	2.552695000	-4.420880000	2.018316000
H	0.542876000	-3.066788000	1.482692000
H	-4.930152000	1.719593000	-2.637751000
H	-6.172144000	0.699411000	-0.658349000
H	-6.092840000	-0.564363000	1.478466000
H	-2.721108000	2.114407000	-3.777077000
H	-0.633143000	1.331951000	-2.690048000
H	-4.710679000	-1.750840000	3.261397000
H	-2.424072000	-2.421506000	4.072236000
H	-0.435176000	-1.875287000	2.693302000
O	-0.181850000	-1.740578000	-1.564137000
H	0.488113000	-2.443996000	-1.554795000
H	-1.031000000	-2.211325000	-1.522773000
O	0.247138000	1.118626000	1.498461000
H	0.887964000	1.822183000	1.302704000
H	-0.576886000	1.588900000	1.709111000

(S=3) *trans*-$[Fe(Phen)_2(H_2O)_2]^{2+}$

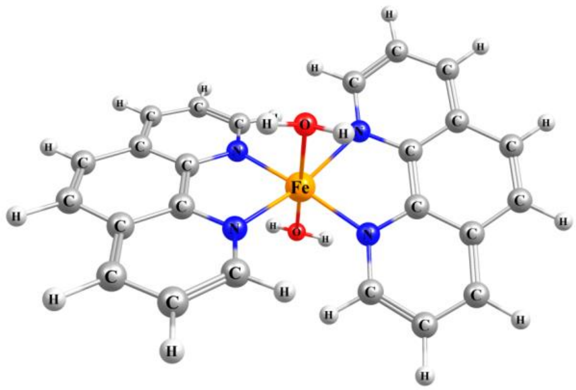

Fe	0.053210000	-0.126136000	0.141015000
N	1.556611000	-1.483552000	0.661322000
N	-1.530654000	0.606048000	-1.003622000
N	1.602264000	0.791285000	-0.721809000
N	-1.461340000	-0.798792000	1.251784000
C	2.789653000	0.142909000	-0.561142000
C	2.758267000	-1.094937000	0.156052000
C	-2.727792000	0.244739000	-0.468936000
C	-2.692346000	-0.479982000	0.763603000
C	-1.383957000	-1.371721000	2.452347000
C	-2.525725000	-1.722947000	3.195322000
C	-3.784236000	-1.467312000	2.677555000
C	-3.896265000	-0.817199000	1.426435000
C	-1.524038000	1.191634000	-2.197238000
C	-2.706447000	1.506220000	-2.893581000
C	-3.932775000	1.201130000	-2.328378000
C	-3.971052000	0.543322000	-1.077044000
C	1.609825000	1.996479000	-1.290488000
C	1.471059000	-2.675440000	1.245056000
C	2.784145000	2.588391000	-1.789969000
C	3.986860000	1.909772000	-1.691532000
C	4.016794000	0.648055000	-1.052857000
C	2.586294000	-3.517794000	1.412835000
C	3.826618000	-3.105943000	0.956453000
C	3.941175000	-1.860489000	0.296332000
C	-5.183105000	0.163903000	-0.402880000
C	-5.147378000	-0.477657000	0.804161000

C	5.210506000	-0.133351000	-0.872912000
C	5.172175000	-1.342404000	-0.235738000
H	0.657990000	2.525850000	-1.347376000
H	2.725144000	3.574277000	-2.253542000
H	4.910815000	2.339309000	-2.085870000
H	6.150653000	0.261353000	-1.264556000
H	6.079954000	-1.937333000	-0.112699000
H	4.712055000	-3.732637000	1.087952000
H	2.455585000	-4.481271000	1.907971000
H	0.487832000	-2.994670000	1.592880000
H	-4.866439000	1.450679000	-2.838213000
H	-6.136565000	0.408630000	-0.876327000
H	-6.071495000	-0.751174000	1.318244000
H	-2.636411000	1.995180000	-3.866473000
H	-0.553972000	1.424029000	-2.637789000
H	-4.686898000	-1.746080000	3.226354000
H	-2.399055000	-2.199438000	4.168631000
H	-0.386511000	-1.554141000	2.853737000
O	-0.182095000	-1.754132000	-1.547940000
H	0.571353000	-2.348280000	-1.690023000
H	-0.925873000	-2.354196000	-1.378249000
O	0.269564000	1.319700000	1.716791000
H	0.943755000	2.017415000	1.694357000
H	-0.515984000	1.724187000	2.118471000

(S=5) *trans*-$[Fe(Phen)_2(H_2O)_2]^{2+}$

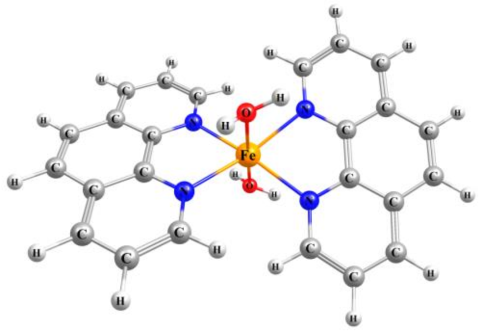

Fe	0.053988000	-0.338870000	0.012340000
N	1.768478000	-1.586032000	0.598157000
N	-1.687229000	0.443885000	-1.084995000
N	1.777521000	0.771814000	-0.784991000
N	-1.648101000	-0.947421000	1.267127000
C	2.961607000	0.143392000	-0.577206000
C	2.956267000	-1.105969000	0.149934000
C	-2.866173000	0.212910000	-0.454705000
C	-2.844899000	-0.517415000	0.792457000
C	-1.603555000	-1.586048000	2.430560000
C	-2.756412000	-1.863836000	3.189379000
C	-3.987272000	-1.451929000	2.711749000
C	-4.061707000	-0.753846000	1.482878000
C	-1.688443000	1.070685000	-2.255811000
C	-2.868453000	1.539154000	-2.864570000
C	-4.077419000	1.338322000	-2.223296000
C	-4.106260000	0.654417000	-0.984416000
C	1.764391000	1.941442000	-1.414384000
C	1.739836000	-2.747787000	1.241406000
C	2.933703000	2.552536000	-1.905672000
C	4.147760000	1.914820000	-1.725335000
C	4.192067000	0.676585000	-1.041031000
C	2.900157000	-3.501135000	1.501978000
C	4.121804000	-3.019645000	1.067759000
C	4.179551000	-1.791419000	0.367005000
C	-5.320518000	0.392338000	-0.262585000
C	-5.298667000	-0.281707000	0.924819000
C	5.412828000	-0.041661000	-0.800334000

C	5.406387000	-1.228939000	-0.125847000
H	0.793075000	2.428578000	-1.532027000
H	2.865432000	3.512921000	-2.419132000
H	5.075840000	2.355521000	-2.097857000
H	6.346884000	0.386028000	-1.171717000
H	6.335145000	-1.774937000	0.054481000
H	5.044025000	-3.576125000	1.252487000
H	2.818917000	-4.448327000	2.037488000
H	0.760517000	-3.114146000	1.559106000
H	-5.013122000	1.694178000	-2.661666000
H	-6.262142000	0.747593000	-0.687393000
H	-6.222285000	-0.477609000	1.474194000
H	-2.812258000	2.052427000	-3.825858000
H	-0.721025000	1.207752000	-2.745275000
H	-4.904082000	-1.651585000	3.272022000
H	-2.662449000	-2.395714000	4.137470000
H	-0.618063000	-1.888453000	2.792946000
O	-0.077377000	-1.835499000	-1.537821000
H	0.370053000	-2.690644000	-1.431546000
H	-0.948954000	-2.036745000	-1.915853000
O	0.173269000	1.179994000	1.544196000
H	0.700158000	1.982048000	1.397429000
H	-0.629031000	1.470775000	2.007720000

$PHSCH_3$

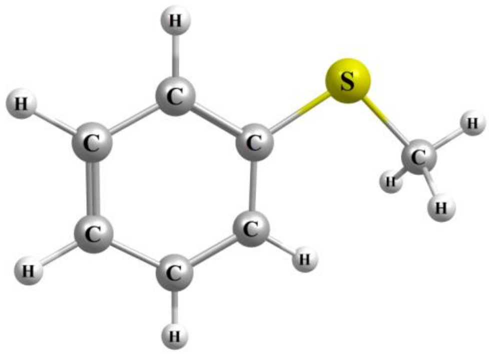

C	-3.996735000	-2.498265000	-2.958745000
C	-3.137889000	-2.166814000	-4.025542000
C	-3.876513000	-3.757351000	-2.348103000
C	-2.180164000	-3.079264000	-4.468238000
C	-2.911555000	-4.664798000	-2.801331000
C	-2.059936000	-4.335163000	-3.858805000
H	-3.224710000	-1.188994000	-4.507718000
H	-4.526700000	-4.042354000	-1.520717000
H	-1.521714000	-2.806216000	-5.297332000
H	-2.830179000	-5.640956000	-2.315327000
H	-1.308707000	-5.048274000	-4.206772000
S	-5.178130000	-1.262308000	-2.474090000
C	-6.090280000	-2.050744000	-1.115475000
H	-6.838498000	-1.310981000	-0.797706000
H	-6.603596000	-2.962192000	-1.454509000
H	-5.427979000	-2.282640000	-0.268864000

$[Fe(Phen)_2]^{2+}$

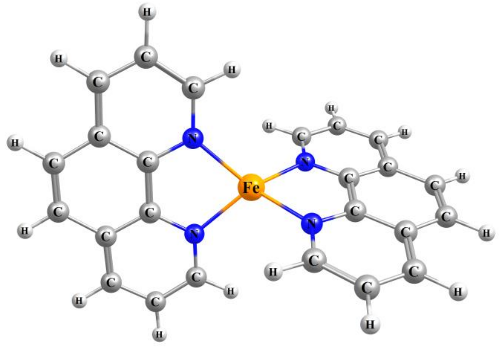

Fe	-0.467409000	0.761877000	2.679608000
N	0.214350000	-0.981902000	1.791588000
N	0.353018000	2.136714000	3.996837000

N	-0.459938000	1.444101000	0.721191000
N	-1.927469000	0.632901000	4.146411000
C	-0.029737000	0.463283000	-0.121385000
C	0.319289000	-0.817629000	0.442048000
C	-0.438861000	2.272083000	5.097346000
C	-1.638581000	1.475666000	5.178572000
C	-3.028107000	-0.114764000	4.192420000
C	-3.917087000	-0.065559000	5.281169000
C	-3.644958000	0.786277000	6.337478000
C	-2.480336000	1.591829000	6.310750000
C	1.464388000	2.861275000	3.887141000
C	1.859308000	3.773149000	4.882187000
C	1.071930000	3.923557000	6.010626000
C	-0.115691000	3.164005000	6.147927000
C	-0.785754000	2.639292000	0.233738000
C	0.528204000	-2.153833000	2.340128000
C	-0.703715000	2.930437000	-1.139754000
C	-0.273405000	1.947381000	-2.013844000
C	0.079062000	0.668450000	-1.517788000
C	0.969912000	-3.243691000	1.568672000
C	1.087799000	-3.096834000	0.197644000
C	0.761640000	-1.859112000	-0.408450000
C	-0.989766000	3.260038000	7.284311000
C	-2.125270000	2.504346000	7.362577000
C	0.532974000	-0.407194000	-2.355252000
C	0.859173000	-1.622094000	-1.822314000
H	-1.118779000	3.392479000	0.952656000
H	-0.980299000	3.924410000	-1.494294000
H	-0.200794000	2.144006000	-3.086237000
H	0.609607000	-0.229128000	-3.429988000
H	1.201974000	-2.438897000	-2.461104000
H	1.430288000	-3.924935000	-0.427715000
H	1.214090000	-4.185530000	2.062090000
H	0.426258000	-2.236097000	3.425377000
H	1.353011000	4.623919000	6.800877000
H	-0.723127000	3.954114000	8.084166000

H	-2.788297000	2.579955000	8.227074000
H	2.778041000	4.346409000	4.750045000
H	2.060921000	2.718053000	2.982408000
H	-4.318435000	0.847885000	7.195825000
H	-4.806810000	-0.696852000	5.275305000
H	-3.214068000	-0.775834000	3.341863000

H_2O

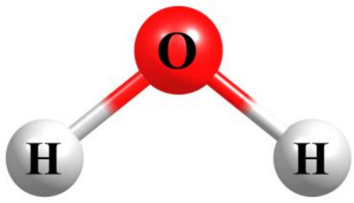

O	-4.448209000	-2.293275000	1.578691000
H	-4.448209000	-1.538362000	2.186193000
H	-4.448209000	-3.048189000	2.186193000

t-BuCOONH$_2$ HOTf

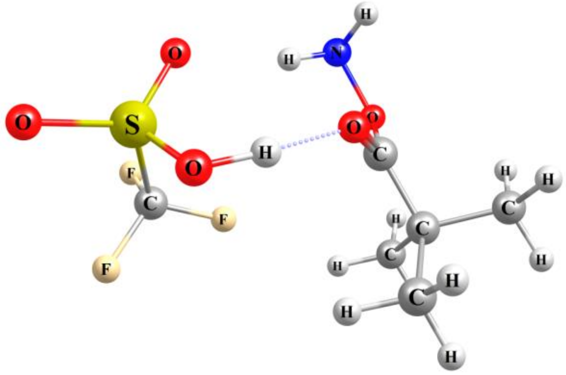

O	0.094303000	-1.729312000	-1.459111000
C	-0.943011000	-2.477376000	-1.836618000
O	-2.090609000	-2.066047000	-1.741670000
C	-0.539123000	-3.859239000	-2.330659000
C	-1.738554000	-4.492434000	-3.049182000
C	0.668888000	-3.762869000	-3.280673000
C	-0.163793000	-4.686797000	-1.078622000
H	-2.599526000	-4.596597000	-2.372972000
H	-2.049791000	-3.882330000	-3.911157000
H	-1.458601000	-5.492112000	-3.413872000
H	1.540521000	-3.319544000	-2.779463000

H	0.939855000	-4.774862000	-3.618540000
H	0.430144000	-3.156619000	-4.167483000
H	0.091841000	-5.711872000	-1.388625000
H	0.703989000	-4.251455000	-0.561322000
H	-1.006462000	-4.739160000	-0.371585000
N	-0.199592000	-0.409142000	-0.970909000
H	-0.844150000	-0.002016000	-1.661405000
H	-0.782696000	-0.562076000	-0.139762000
H	-3.128119000	-1.582552000	-2.773707000
C	-1.855326000	-0.149711000	-5.137856000
S	-3.172956000	0.290775000	-3.872654000
F	-1.104769000	0.919406000	-5.358126000
F	-1.091726000	-1.133486000	-4.660984000
F	-2.421905000	-0.540897000	-6.267478000
O	-3.709654000	-1.147935000	-3.486071000
O	-4.229150000	0.983801000	-4.587709000
O	-2.464333000	0.920128000	-2.760653000

TfOH

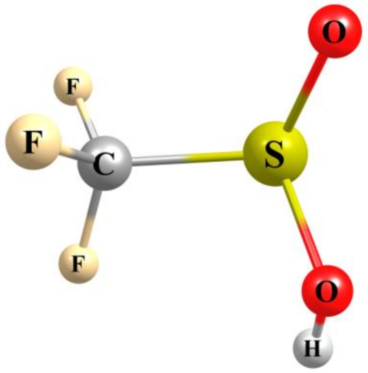

H	-3.181343000	-1.582027000	-2.811366000
C	-1.845529000	-0.149012000	-5.135167000
S	-3.150610000	0.308549000	-3.863570000
F	-1.138089000	0.935125000	-5.420073000
F	-1.047951000	-1.083662000	-4.635079000
F	-2.433567000	-0.601749000	-6.231134000
O	-3.718585000	-1.154623000	-3.511246000
O	-4.212507000	0.999895000	-4.569527000
O	-2.449758000	0.887033000	-2.728077000

NH_4OTf

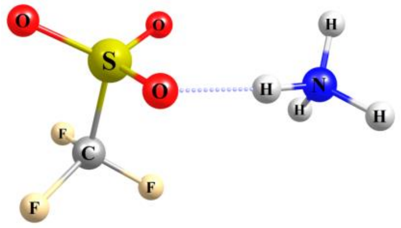

N	-1.060711000	-0.697083000	-1.217373000
H	-1.346997000	0.039842000	-0.564344000
H	-0.805105000	-1.541022000	-0.694324000
H	-1.856463000	-0.894200000	-1.890996000
C	-1.835993000	-0.160017000	-5.257998000
S	-2.958828000	0.315245000	-3.843245000
F	-1.597184000	0.893584000	-6.044528000
F	-0.664495000	-0.609945000	-4.792242000
F	-2.391450000	-1.123816000	-5.997438000
O	-3.030751000	-0.961999000	-3.059077000
O	-4.221437000	0.699344000	-4.493870000
O	-2.209682000	1.384464000	-3.149758000
H	-0.251788000	-0.368336000	-1.755599000

CO_2

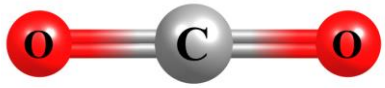

O	-4.073089000	-3.555042000	-5.266502000
C	-3.714501000	-3.190697000	-4.221748000
O	-3.354458000	-2.826242000	-3.177464000

Isoprene

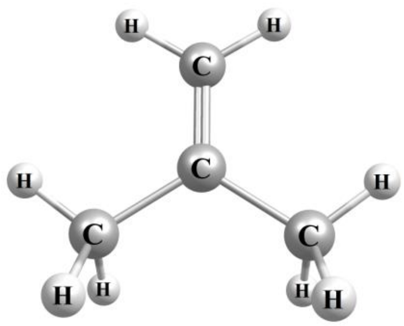

C	-0.952314000	-5.875887000	-3.490448000
C	-2.057450000	-5.981779000	-2.736957000
C	-0.912479000	-6.329502000	-4.927072000
C	0.332064000	-5.295449000	-2.956654000
H	-2.069916000	-5.648337000	-1.694151000
H	-2.983544000	-6.405021000	-3.139395000
H	-0.637898000	-5.492060000	-5.593176000
H	-0.138329000	-7.104582000	-5.070053000
H	-1.878965000	-6.737066000	-5.259286000
H	1.154979000	-6.027940000	-3.038496000
H	0.639869000	-4.417147000	-3.551979000
H	0.241490000	-4.988277000	-1.903885000

Int 1

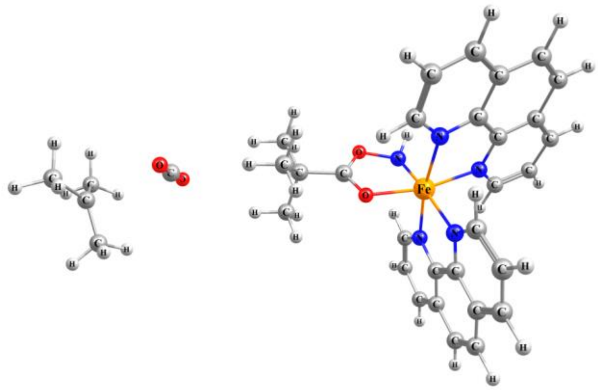

Fe	-0.357768000	-0.562681000	0.321746000
N	1.110016000	-1.089638000	1.660274000
N	-0.492656000	1.351804000	1.155552000
N	1.387698000	-0.195848000	-0.849100000
N	-1.902450000	-0.863226000	1.811391000
C	2.525037000	-0.473784000	-0.167404000
C	2.376444000	-0.950791000	1.175456000
C	-1.428510000	1.460034000	2.131558000
C	-2.186688000	0.285644000	2.471716000
C	-2.581249000	-1.970248000	2.093278000
C	-3.595453000	-1.993125000	3.069612000
C	-3.895976000	-0.832311000	3.761340000
C	-3.184157000	0.357050000	3.474713000
C	0.229325000	2.413395000	0.813892000

C	0.056944000	3.662724000	1.438960000
C	-0.894954000	3.797512000	2.434291000
C	-1.676207000	2.678642000	2.809400000
C	1.458285000	0.245286000	-2.099766000
C	0.943353000	-1.534086000	2.903338000
C	2.694424000	0.441446000	-2.745699000
C	3.868648000	0.166912000	-2.063976000
C	3.811585000	-0.308349000	-0.731103000
C	2.029758000	-1.867112000	3.733962000
C	3.321085000	-1.731350000	3.257642000
C	3.526738000	-1.259983000	1.939566000
C	-2.691713000	2.720834000	3.825586000
C	-3.417856000	1.607403000	4.143176000
C	4.963975000	-0.626040000	0.066144000
C	4.825320000	-1.083332000	1.347330000
H	0.508056000	0.452135000	-2.598897000
H	2.709685000	0.806789000	-3.773660000
H	4.840547000	0.310875000	-2.542144000
H	5.954199000	-0.494058000	-0.375361000
H	5.702608000	-1.325520000	1.951156000
H	4.181355000	-1.982514000	3.882591000
H	1.830443000	-2.227914000	4.744045000
H	-0.078553000	-1.637414000	3.267337000
H	-1.052387000	4.756110000	2.934386000
H	-2.870008000	3.668756000	4.338096000
H	-4.189957000	1.646157000	4.914665000
H	0.675719000	4.505034000	1.126073000
H	0.969445000	2.280768000	0.021917000
H	-4.674882000	-0.821102000	4.527585000
H	-4.125292000	-2.925822000	3.268766000
H	-2.312378000	-2.873464000	1.540952000
O	-2.124907000	-1.268347000	-1.646327000
C	-1.536425000	-2.402966000	-1.346848000
O	-0.671420000	-2.432702000	-0.452501000
C	-1.956177000	-3.617940000	-2.143440000
C	-2.286161000	-4.738993000	-1.137191000

C	-3.166764000	-3.314291000	-3.037077000
C	-0.735232000	-4.012812000	-3.008038000
H	-1.418591000	-4.970516000	-0.503002000
H	-3.128211000	-4.450039000	-0.489046000
H	-2.570992000	-5.648006000	-1.685463000
H	-2.942877000	-2.515242000	-3.758525000
H	-3.428630000	-4.221818000	-3.599604000
H	-4.039838000	-3.011158000	-2.439861000
H	-0.998742000	-4.895453000	-3.609203000
H	-0.456893000	-3.197440000	-3.693762000
H	0.133004000	-4.258498000	-2.379406000
N	-1.549585000	-0.154903000	-0.975745000
H	-2.293789000	0.547226000	-0.955400000
O	-3.242903000	-7.905549000	-3.137336000
C	-3.006100000	-7.280387000	-4.090195000
O	-2.766714000	-6.603237000	-5.007011000
C	-2.846351000	-9.732624000	-5.767103000
C	-3.670800000	-10.669722000	-4.943814000
C	-3.425978000	-9.181664000	-7.030895000
C	-1.358069000	-9.761869000	-5.615014000
H	-3.289366000	-10.751471000	-3.912518000
H	-4.727564000	-10.356360000	-4.901951000
H	-3.662237000	-11.698178000	-5.368743000
H	-2.886516000	-8.281303000	-7.368558000
H	-3.364216000	-9.919779000	-7.861466000
H	-4.493259000	-8.927452000	-6.916308000
H	-0.907212000	-10.606424000	-6.182320000
H	-0.889321000	-8.840703000	-6.000543000
H	-1.056635000	-9.894792000	-4.562156000

Int 1 (S=2)

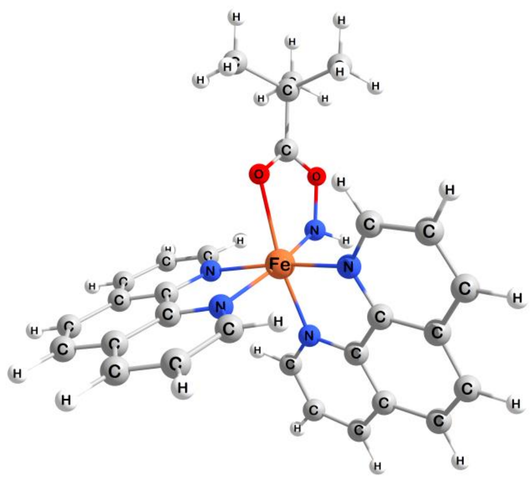

Fe	-0.321279000	-0.490210000	0.344794000
N	1.280038000	-0.968805000	1.777783000
N	-0.575843000	1.315257000	1.196414000
N	1.390360000	0.016629000	-0.737587000
N	-1.737899000	-1.013571000	1.697100000
C	2.566178000	-0.193540000	-0.084808000
C	2.507141000	-0.713742000	1.252622000
C	-1.501287000	1.295445000	2.197630000
C	-2.130802000	0.040519000	2.464461000
C	-2.284339000	-2.210262000	1.897807000
C	-3.261340000	-2.414280000	2.891232000
C	-3.671079000	-1.353705000	3.682903000
C	-3.101182000	-0.072988000	3.483616000
C	0.038227000	2.459092000	0.906375000
C	-0.241165000	3.647900000	1.608612000
C	-1.177845000	3.643559000	2.628045000
C	-1.845061000	2.438085000	2.952486000
C	1.402403000	0.494947000	-1.978589000
C	1.183813000	-1.445791000	3.012570000
C	2.602830000	0.800788000	-2.645982000
C	3.809744000	0.600602000	-1.999331000
C	3.819292000	0.088353000	-0.680086000
C	2.320194000	-1.705691000	3.803627000
C	3.578988000	-1.457106000	3.286188000
C	3.704202000	-0.944062000	1.972805000

C	-2.836008000	2.304408000	3.986774000
C	-3.438617000	1.102283000	4.241038000
C	5.019062000	-0.155128000	0.073202000
C	4.963869000	-0.650932000	1.345604000
H	0.429409000	0.642939000	-2.452339000
H	2.561745000	1.192941000	-3.663206000
H	4.756651000	0.832016000	-2.492939000
H	5.978855000	0.067121000	-0.398257000
H	5.878761000	-0.834419000	1.913378000
H	4.477733000	-1.650184000	3.876900000
H	2.188589000	-2.099820000	4.812466000
H	0.181177000	-1.635487000	3.400773000
H	-1.407743000	4.555793000	3.183612000
H	-3.097970000	3.190792000	4.568582000
H	-4.189383000	1.011772000	5.029122000
H	0.290640000	4.559775000	1.333114000
H	0.772534000	2.443401000	0.099655000
H	-4.427455000	-1.494153000	4.458745000
H	-3.681901000	-3.412418000	3.021388000
H	-1.940084000	-3.028552000	1.262322000
O	-1.929685000	-1.249313000	-1.792170000
C	-1.256068000	-2.379894000	-1.611486000
O	-0.394248000	-2.469522000	-0.732744000
C	-1.694449000	-3.521449000	-2.507755000
C	-2.941602000	-4.143942000	-1.833301000
C	-2.049611000	-3.002556000	-3.911956000
C	-0.562339000	-4.556357000	-2.583133000
H	-2.703342000	-4.507156000	-0.821618000
H	-3.763330000	-3.415553000	-1.763145000
H	-3.281838000	-4.998013000	-2.438584000
H	-1.182639000	-2.515954000	-4.385355000
H	-2.353400000	-3.853404000	-4.540013000
H	-2.879878000	-2.283135000	-3.878830000
H	-0.891025000	-5.401369000	-3.206087000
H	0.342144000	-4.121572000	-3.035848000
H	-0.303524000	-4.939151000	-1.585675000

N	-1.511647000	-0.176159000	-0.972209000
H	-2.302427000	0.473727000	-0.990256000

Int 1 (S=4)

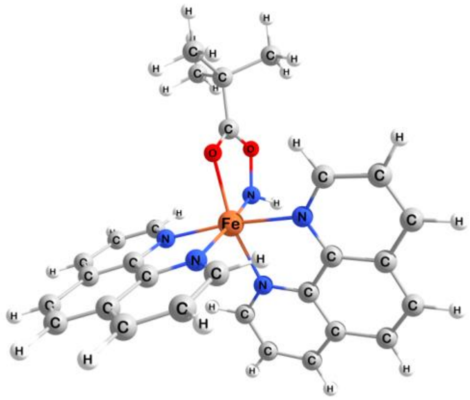

Fe	-0.259007000	-0.520079000	0.276034000
N	1.279797000	-0.981988000	1.555205000
N	-0.565433000	1.297531000	1.261833000
N	1.415458000	0.090808000	-0.892994000
N	-1.745025000	-1.089112000	1.747673000
C	2.589168000	-0.114904000	-0.248152000
C	2.516176000	-0.686547000	1.063494000
C	-1.492299000	1.236750000	2.250264000
C	-2.130416000	-0.028409000	2.498751000
C	-2.316625000	-2.273398000	1.938878000
C	-3.315972000	-2.469573000	2.911134000
C	-3.715369000	-1.402095000	3.696896000
C	-3.119348000	-0.132262000	3.507227000
C	0.048142000	2.446978000	1.002786000
C	-0.232360000	3.620065000	1.728511000
C	-1.178638000	3.582991000	2.737618000
C	-1.844893000	2.368129000	3.025744000
C	1.415453000	0.614018000	-2.113695000
C	1.182301000	-1.514325000	2.770883000
C	2.612481000	0.972155000	-2.763963000
C	3.822336000	0.774024000	-2.118850000
C	3.840835000	0.212977000	-0.818694000
C	2.312605000	-1.785687000	3.564507000
C	3.574560000	-1.492720000	3.079789000

C	3.706332000	-0.924339000	1.790817000
C	-2.845953000	2.231627000	4.047817000
C	-3.460882000	1.032655000	4.276357000
C	5.035286000	-0.036895000	-0.059796000
C	4.968758000	-0.583075000	1.192062000
H	0.438881000	0.754591000	-2.584029000
H	2.569919000	1.401615000	-3.766029000
H	4.764786000	1.045455000	-2.600754000
H	5.998393000	0.220172000	-0.506174000
H	5.878104000	-0.772907000	1.766530000
H	4.467687000	-1.693707000	3.676221000
H	2.170900000	-2.225034000	4.552945000
H	0.182505000	-1.741675000	3.140493000
H	-1.419829000	4.478612000	3.315200000
H	-3.106237000	3.113231000	4.637650000
H	-4.223487000	0.935119000	5.052250000
H	0.298862000	4.540220000	1.480594000
H	0.785259000	2.448872000	0.196889000
H	-4.484619000	-1.526154000	4.462912000
H	-3.756277000	-3.460335000	3.032584000
H	-1.971670000	-3.099427000	1.312509000
O	-2.011362000	-1.264040000	-1.688742000
C	-1.308120000	-2.357890000	-1.485067000
O	-0.409187000	-2.361505000	-0.627832000
C	-1.697041000	-3.542670000	-2.341757000
C	-3.190917000	-3.844144000	-2.089807000
C	-1.466784000	-3.159969000	-3.820510000
C	-0.832364000	-4.749170000	-1.955264000
H	-3.373529000	-4.071770000	-1.027972000
H	-3.825825000	-2.995227000	-2.381541000
H	-3.481701000	-4.720802000	-2.687995000
H	-0.412405000	-2.898793000	-4.001308000
H	-1.723695000	-4.021603000	-4.455011000
H	-2.095992000	-2.307672000	-4.115506000
H	-1.120222000	-5.608558000	-2.578496000
H	0.235833000	-4.543087000	-2.118033000

H	-0.976580000	-5.021399000	-0.899030000
N	-1.512356000	-0.144444000	-0.971399000
H	-2.313161000	0.487592000	-0.892194000

Int 1 (S=6)

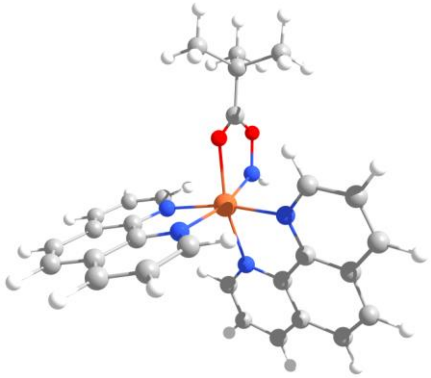

Fe	-0.333856000	-0.447269000	0.259464000
N	1.269664000	-1.120984000	1.582722000
N	-0.631842000	1.428108000	1.242845000
N	1.441284000	0.136347000	-0.810739000
N	-1.821791000	-0.957139000	1.769247000
C	2.597443000	-0.138487000	-0.156374000
C	2.505654000	-0.798355000	1.119780000
C	-1.538347000	1.372363000	2.254546000
C	-2.176972000	0.111252000	2.526459000
C	-2.403383000	-2.132791000	1.980589000
C	-3.377537000	-2.317076000	2.980653000
C	-3.745048000	-1.242818000	3.771855000
C	-3.142769000	0.020577000	3.559091000
C	-0.019801000	2.576948000	0.968971000
C	-0.281722000	3.753840000	1.693270000
C	-1.206968000	3.721286000	2.722189000
C	-1.869370000	2.509344000	3.031656000
C	1.483421000	0.720670000	-2.004834000
C	1.157018000	-1.729293000	2.758654000
C	2.697869000	1.083112000	-2.616020000
C	3.887954000	0.824478000	-1.957902000
C	3.866177000	0.194735000	-0.690133000

C	2.275636000	-2.059863000	3.546815000
C	3.542003000	-1.743894000	3.088177000
C	3.689499000	-1.094102000	1.839283000
C	-2.846050000	2.385673000	4.078507000
C	-3.460303000	1.191254000	4.329534000
C	5.049211000	-0.117236000	0.063227000
C	4.963460000	-0.733974000	1.279535000
H	0.524134000	0.912654000	-2.491585000
H	2.683236000	1.563370000	-3.595521000
H	4.847006000	1.098134000	-2.404522000
H	6.019253000	0.151188000	-0.360985000
H	5.863860000	-0.970686000	1.850791000
H	4.430381000	-1.989269000	3.675280000
H	2.126475000	-2.561067000	4.504389000
H	0.148581000	-1.969965000	3.099475000
H	-1.434422000	4.619699000	3.301194000
H	-3.087672000	3.272815000	4.667993000
H	-4.204795000	1.101920000	5.123843000
H	0.245699000	4.672274000	1.431546000
H	0.702611000	2.572594000	0.149694000
H	-4.494471000	-1.355223000	4.559075000
H	-3.824301000	-3.302940000	3.117768000
H	-2.086512000	-2.962596000	1.343975000
O	-1.907993000	-1.260034000	-2.032780000
C	-1.289091000	-2.376207000	-1.650714000
O	-0.526884000	-2.353104000	-0.681271000
C	-1.651722000	-3.614326000	-2.438280000
C	-2.882554000	-4.228556000	-1.724056000
C	-2.004740000	-3.259517000	-3.892367000
C	-0.468184000	-4.593373000	-2.385949000
H	-2.645406000	-4.493776000	-0.682475000
H	-3.734409000	-3.531798000	-1.727441000
H	-3.176910000	-5.143968000	-2.259814000
H	-1.157585000	-2.773233000	-4.400322000
H	-2.246532000	-4.186248000	-4.433876000
H	-2.876008000	-2.591556000	-3.946548000

H	-0.742669000	-5.516206000	-2.918039000
H	0.422154000	-4.163621000	-2.870628000
H	-0.210951000	-4.851544000	-1.348874000
N	-1.607566000	-0.171972000	-1.223064000
H	-2.147309000	0.598229000	-1.626522000

$TS_{1\rightarrow2}$

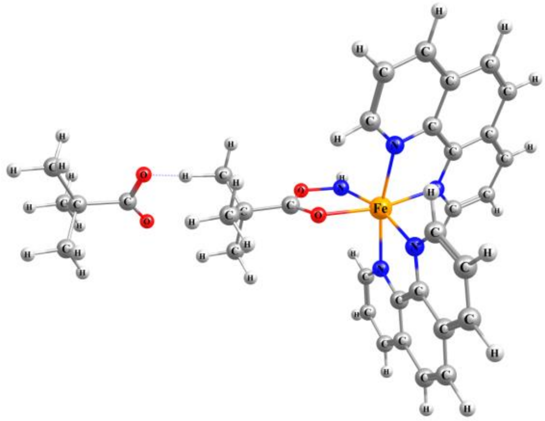

Fe	-0.524003000	-0.706156000	0.256097000
N	1.186097000	-1.231414000	1.506667000
N	-0.824144000	1.238209000	1.082023000
N	1.155228000	-0.182271000	-0.990037000
N	-1.893068000	-1.123045000	1.890624000
C	2.362375000	-0.398974000	-0.409857000
C	2.378433000	-0.950878000	0.919287000
C	-1.664725000	1.247045000	2.151050000
C	-2.241457000	-0.002824000	2.571771000
C	-2.417080000	-2.293111000	2.239154000
C	-3.323075000	-2.416902000	3.309879000
C	-3.682236000	-1.287941000	4.025288000
C	-3.140387000	-0.030230000	3.666028000
C	-0.264441000	2.374337000	0.674493000
C	-0.517709000	3.601669000	1.312810000
C	-1.379498000	3.634884000	2.395501000
C	-1.985088000	2.437793000	2.847186000
C	1.097916000	0.302983000	-2.227302000
C	1.171500000	-1.740627000	2.734019000

C	2.256677000	0.621169000	-2.959230000
C	3.497637000	0.419046000	-2.379899000
C	3.581894000	-0.108762000	-1.069014000
C	2.351939000	-2.007273000	3.453061000
C	3.575368000	-1.733596000	2.867956000
C	3.618076000	-1.189233000	1.562086000
C	-2.894709000	2.379320000	3.958325000
C	-3.452383000	1.194681000	4.349395000
C	4.823702000	-0.360731000	-0.391413000
C	4.840451000	-0.878535000	0.872943000
H	0.101545000	0.447810000	-2.651680000
H	2.160154000	1.021135000	-3.969683000
H	4.415628000	0.657962000	-2.922490000
H	5.754814000	-0.128730000	-0.913154000
H	5.785670000	-1.070107000	1.385657000
H	4.509815000	-1.931973000	3.398564000
H	2.283546000	-2.426977000	4.457803000
H	0.194990000	-1.950806000	3.174024000
H	-1.599794000	4.574341000	2.908245000
H	-3.130843000	3.308275000	4.481997000
H	-4.145919000	1.155168000	5.192224000
H	-0.034590000	4.507127000	0.942821000
H	0.407177000	2.318030000	-0.185180000
H	-4.378750000	-1.353055000	4.864679000
H	-3.724015000	-3.399981000	3.561237000
H	-2.106627000	-3.166462000	1.659969000
O	-2.080822000	-1.874914000	-1.893107000
C	-1.359932000	-2.890984000	-1.422291000
O	-0.621887000	-2.728120000	-0.452353000
C	-1.496661000	-4.184904000	-2.205904000
C	-2.954556000	-4.353096000	-2.619356000
C	-0.570749000	-4.038043000	-3.437721000
C	-1.035978000	-5.348932000	-1.312851000
H	-3.666830000	-4.350613000	-1.781232000
H	-3.283669000	-3.731707000	-3.461174000
H	-3.097150000	-5.520133000	-3.025351000

H	0.474637000	-3.908892000	-3.119996000
H	-0.655790000	-4.943077000	-4.054446000
H	-0.865646000	-3.173991000	-4.051715000
H	-1.082812000	-6.283203000	-1.888910000
H	-0.002658000	-5.193815000	-0.973682000
H	-1.685004000	-5.449796000	-0.429642000
N	-1.870408000	-0.694717000	-1.188728000
H	-2.482710000	-0.015818000	-1.648731000
O	-3.278738000	-6.789566000	-3.367627000
C	-2.881446000	-6.940840000	-4.604763000
O	-2.430621000	-6.068112000	-5.323377000
C	-3.002948000	-8.424498000	-5.080729000
C	-3.843741000	-9.289452000	-4.133628000
C	-3.630615000	-8.399394000	-6.483179000
C	-1.556878000	-8.953863000	-5.144819000
H	-3.399520000	-9.337498000	-3.128848000
H	-4.869248000	-8.901122000	-4.039830000
H	-3.899828000	-10.313977000	-4.534233000
H	-3.039667000	-7.774795000	-7.167517000
H	-3.667635000	-9.425577000	-6.882412000
H	-4.659437000	-8.006916000	-6.451429000
H	-1.567551000	-9.994647000	-5.506503000
H	-0.945395000	-8.350462000	-5.831800000
H	-1.084632000	-8.941305000	-4.149724000

Int 2

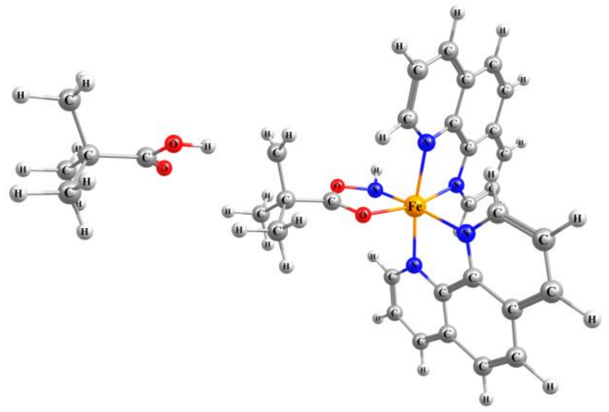

Fe	-0.172616000	-0.672945000	0.140694000
N	1.315504000	-1.020436000	1.512764000
N	-0.656913000	1.137545000	1.072720000
N	1.524135000	-0.004937000	-0.954463000
N	-1.692975000	-1.319333000	1.533770000
C	2.672954000	-0.143572000	-0.249351000
C	2.560744000	-0.683872000	1.072699000
C	-1.639492000	1.027123000	2.001297000
C	-2.203050000	-0.275998000	2.233505000
C	-2.184942000	-2.539797000	1.719980000
C	-3.230190000	-2.790862000	2.628921000
C	-3.759815000	-1.740692000	3.358959000
C	-3.245727000	-0.434063000	3.178797000
C	-0.105292000	2.321761000	0.832795000
C	-0.509612000	3.482559000	1.519138000
C	-1.515073000	3.395061000	2.466087000
C	-2.117007000	2.142067000	2.732407000
C	1.560846000	0.488350000	-2.187028000
C	1.180907000	-1.519551000	2.739146000
C	2.771758000	0.881509000	-2.789266000
C	3.956467000	0.750946000	-2.083210000
C	3.935718000	0.224050000	-0.768818000
C	2.281185000	-1.716526000	3.594176000
C	3.552020000	-1.383539000	3.161374000
C	3.722929000	-0.847908000	1.862982000
C	-3.172457000	1.951273000	3.689165000
C	-3.717846000	0.715842000	3.899856000

C	5.101395000	0.046344000	0.052583000
C	4.997318000	-0.468089000	1.315271000
H	0.602942000	0.574792000	-2.706286000
H	2.759120000	1.283549000	-3.803463000
H	4.909087000	1.050052000	-2.527201000
H	6.073178000	0.333192000	-0.355111000
H	5.884647000	-0.602035000	1.937871000
H	4.422457000	-1.527716000	3.805829000
H	2.109270000	-2.131133000	4.588510000
H	0.175248000	-1.780045000	3.068621000
H	-1.852777000	4.280009000	3.010776000
H	-3.531633000	2.820996000	4.243590000
H	-4.522255000	0.576504000	4.625486000
H	-0.026163000	4.433361000	1.289823000
H	0.680628000	2.362348000	0.075260000
H	-4.567783000	-1.906561000	4.075657000
H	-3.602312000	-3.809636000	2.746864000
H	-1.734270000	-3.350708000	1.143868000
O	-1.763720000	-1.556468000	-1.909717000
C	-1.017375000	-2.605846000	-1.638911000
O	-0.163619000	-2.546903000	-0.740702000
C	-1.341171000	-3.858705000	-2.447323000
C	-2.642846000	-4.359308000	-1.870103000
C	-1.495451000	-3.495875000	-3.937279000
C	-0.217450000	-4.888321000	-2.237513000
H	-2.641716000	-4.839837000	-0.886381000
H	-3.592403000	-4.012945000	-2.287591000
H	-2.927198000	-6.379072000	-2.616865000
H	-0.547767000	-3.107624000	-4.340200000
H	-1.783623000	-4.399063000	-4.491973000
H	-2.278137000	-2.737714000	-4.081851000
H	-0.461180000	-5.802571000	-2.797022000
H	0.741943000	-4.494909000	-2.605227000
H	-0.107888000	-5.146760000	-1.174737000
N	-1.372445000	-0.396929000	-1.186763000
H	-2.216739000	0.180912000	-1.158120000

O	-3.020707000	-7.306389000	-2.926474000
C	-3.273177000	-7.294101000	-4.243364000
O	-3.352921000	-6.262133000	-4.877588000
C	-3.439877000	-8.699578000	-4.821882000
C	-4.622920000	-9.387692000	-4.110199000
C	-3.712393000	-8.598097000	-6.326776000
C	-2.140966000	-9.492506000	-4.568322000
H	-4.437942000	-9.482771000	-3.030417000
H	-5.556499000	-8.820713000	-4.255173000
H	-4.766781000	-10.396178000	-4.528935000
H	-2.881730000	-8.099191000	-6.848518000
H	-3.831592000	-9.607395000	-6.749859000
H	-4.631485000	-8.027279000	-6.527980000
H	-2.237082000	-10.501183000	-5.000040000
H	-1.277080000	-8.998476000	-5.041372000
H	-1.937503000	-9.592999000	-3.492469000

Int 3

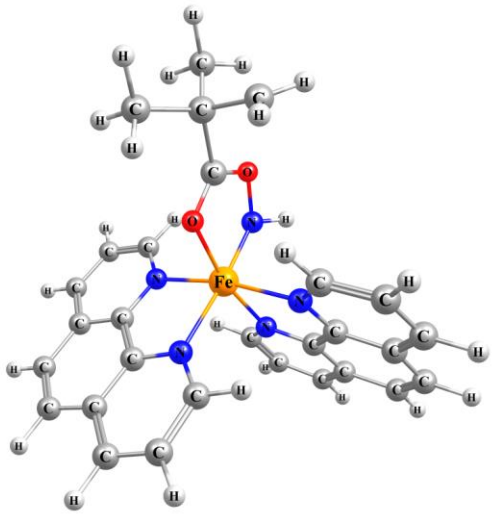

Fe	-0.376541000	-0.709239000	0.207907000
N	1.270650000	-1.119205000	1.367140000
N	-0.626978000	1.141469000	1.151176000
N	1.188291000	-0.107871000	-1.110710000
N	-1.721166000	-1.242926000	1.815874000
C	2.413680000	-0.277676000	-0.557558000
C	2.457379000	-0.816973000	0.768979000

C	-1.474756000	1.104480000	2.209506000
C	-2.066555000	-0.160619000	2.555148000
C	-2.245321000	-2.430275000	2.101478000
C	-3.155648000	-2.606831000	3.160686000
C	-3.516143000	-1.515723000	3.932557000
C	-2.968399000	-0.242697000	3.643970000
C	-0.056200000	2.289856000	0.803904000
C	-0.302694000	3.485217000	1.505281000
C	-1.169508000	3.472436000	2.583834000
C	-1.788811000	2.259003000	2.967265000
C	1.081843000	0.384728000	-2.339394000
C	1.281321000	-1.620708000	2.599659000
C	2.216259000	0.746986000	-3.091718000
C	3.477299000	0.585556000	-2.541439000
C	3.609622000	0.056688000	-1.234331000
C	2.476546000	-1.852950000	3.305535000
C	3.689469000	-1.552579000	2.712527000
C	3.706522000	-1.016727000	1.403352000
C	-2.704627000	2.145683000	4.069040000
C	-3.273375000	0.945698000	4.392196000
C	4.865576000	-0.154823000	-0.568742000
C	4.910370000	-0.670743000	0.696745000
H	0.068674000	0.498137000	-2.733470000
H	2.085559000	1.150869000	-4.096814000
H	4.373079000	0.860860000	-3.103486000
H	5.784562000	0.106472000	-1.098077000
H	5.866344000	-0.831232000	1.199940000
H	4.631670000	-1.721911000	3.239116000
H	2.423221000	-2.267228000	4.313315000
H	0.318891000	-1.852271000	3.055587000
H	-1.383342000	4.385759000	3.144144000
H	-2.935665000	3.045646000	4.643209000
H	-3.969600000	0.865515000	5.229941000
H	0.191383000	4.403254000	1.183631000
H	0.617406000	2.273491000	-0.055601000
H	-4.216483000	-1.623451000	4.764328000

H	-3.558733000	-3.601051000	3.359021000
H	-1.931566000	-3.273482000	1.482073000
O	-2.257204000	-1.525939000	-1.610487000
C	-1.535418000	-2.608160000	-1.414777000
O	-0.573616000	-2.578173000	-0.627533000
C	-2.017555000	-3.860967000	-2.135942000
C	-3.087993000	-4.402306000	-1.221890000
C	-2.594467000	-3.500766000	-3.518260000
C	-0.845046000	-4.846818000	-2.262610000
H	-2.818355000	-5.083147000	-0.410441000
H	-4.101718000	-3.996854000	-1.272260000
H	-1.824596000	-3.029835000	-4.148661000
H	-2.936666000	-4.422086000	-4.011564000
H	-3.447713000	-2.813793000	-3.430706000
H	-1.203841000	-5.765486000	-2.748859000
H	-0.038316000	-4.416178000	-2.875026000
H	-0.435940000	-5.107660000	-1.276509000
N	-1.730398000	-0.383430000	-0.947521000
H	-2.536719000	0.233856000	-0.820998000

$TS_{3\rightarrow4}$

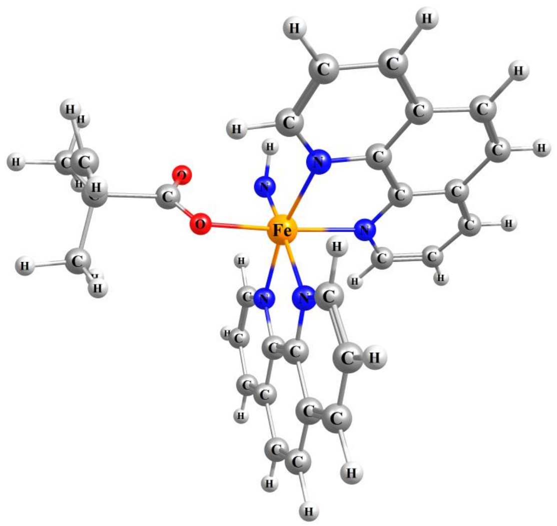

Fe	-0.718040000	-0.559789000	0.127417000
N	0.922414000	-1.151807000	1.320212000
N	-0.676609000	1.299243000	1.170069000
N	0.913405000	-0.056622000	-1.137099000
N	-2.052221000	-0.950086000	1.755859000
C	2.119719000	-0.328275000	-0.581850000
C	2.124522000	-0.906418000	0.730848000
C	-1.404639000	1.283905000	2.312737000
C	-2.141275000	0.088927000	2.622209000
C	-2.720255000	-2.073489000	1.998575000
C	-3.522227000	-2.225478000	3.145000000
C	-3.619892000	-1.180552000	4.048324000
C	-2.919020000	0.024528000	3.803687000
C	0.006110000	2.388007000	0.838425000
C	0.009911000	3.538792000	1.650228000
C	-0.719574000	3.542378000	2.826349000
C	-1.461091000	2.393090000	3.190999000
C	0.844187000	0.460832000	-2.358166000
C	0.898150000	-1.702402000	2.529699000
C	2.003066000	0.751132000	-3.103093000
C	3.246887000	0.494600000	-2.550021000
C	3.337342000	-0.063703000	-1.251656000
C	2.074365000	-2.033299000	3.229181000
C	3.304467000	-1.784534000	2.647870000

C	3.358093000	-1.206683000	1.357257000
C	-2.255240000	2.299562000	4.385328000
C	-2.955668000	1.163168000	4.679507000
C	4.575271000	-0.372461000	-0.590697000
C	4.583526000	-0.920183000	0.661915000
H	-0.158527000	0.652714000	-2.748503000
H	1.905001000	1.177341000	-4.102592000
H	4.162208000	0.715928000	-3.104362000
H	5.510176000	-0.160472000	-1.114028000
H	5.525926000	-1.156065000	1.161063000
H	4.233277000	-2.030271000	3.168198000
H	1.993643000	-2.482268000	4.220174000
H	-0.078337000	-1.897175000	2.974358000
H	-0.734207000	4.420847000	3.475849000
H	-2.286979000	3.162900000	5.053705000
H	-3.558475000	1.098662000	5.587935000
H	0.588410000	4.409347000	1.337492000
H	0.571606000	2.358705000	-0.095116000
H	-4.231697000	-1.273205000	4.948987000
H	-4.051209000	-3.166029000	3.305605000
H	-2.610597000	-2.883371000	1.274460000
O	-2.272718000	-1.734475000	-2.156831000
C	-1.597307000	-2.587390000	-1.587820000
O	-0.854079000	-2.441953000	-0.582084000
C	-1.628487000	-4.092453000	-2.252019000
C	-2.098925000	-4.926643000	-1.162930000
C	-2.550376000	-4.103718000	-3.471572000
C	-0.148238000	-4.333474000	-2.631040000
H	-1.442053000	-5.156466000	-0.319591000
H	-3.146269000	-5.238178000	-1.110139000
H	-2.209647000	-3.378476000	-4.223422000
H	-2.535249000	-5.108355000	-3.920574000
H	-3.584647000	-3.860335000	-3.189621000
H	-0.077563000	-5.295239000	-3.160559000
H	0.208672000	-3.538230000	-3.302437000
H	0.491617000	-4.365458000	-1.739182000

N	-1.949291000	-0.020626000	-0.973257000
H	-2.940807000	-0.045105000	-0.686243000

Int 4

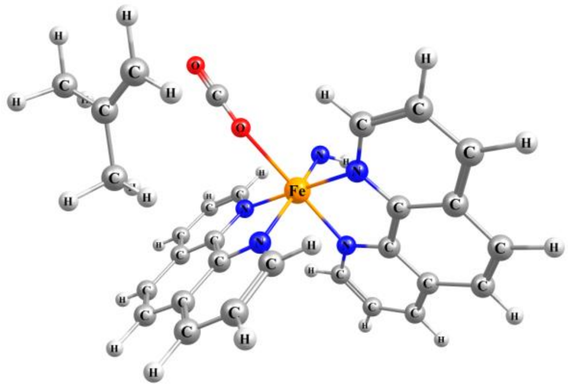

Fe	-1.136958000	0.111808000	0.123352000
N	0.381107000	-1.050460000	0.953234000
N	-0.592389000	1.634679000	1.514394000
N	0.278661000	0.345398000	-1.329732000
N	-2.432268000	-0.345171000	1.674923000
C	1.438983000	-0.308762000	-1.050044000
C	1.497562000	-1.045769000	0.175319000
C	-1.278343000	1.503270000	2.674769000
C	-2.263670000	0.459662000	2.755975000
C	-3.346168000	-1.310356000	1.701437000
C	-4.146221000	-1.538882000	2.836275000
C	-3.980902000	-0.743192000	3.956751000
C	-3.017650000	0.293654000	3.942480000
C	0.324808000	2.584724000	1.386636000
C	0.618259000	3.472570000	2.439775000
C	-0.068489000	3.357269000	3.636439000
C	-1.054694000	2.352288000	3.784902000
C	0.165884000	1.039624000	-2.459312000
C	0.388093000	-1.740101000	2.090039000
C	1.221648000	1.116547000	-3.385485000
C	2.409934000	0.455431000	-3.121101000
C	2.549444000	-0.287356000	-1.923397000
C	1.518619000	-2.460934000	2.519341000
C	2.662178000	-2.465102000	1.739689000

C	2.675831000	-1.746699000	0.520260000
C	-1.826739000	2.155734000	4.980757000
C	-2.770673000	1.169632000	5.054964000
C	3.735196000	-1.010973000	-1.554049000
C	3.796244000	-1.708397000	-0.379118000
H	-0.789790000	1.540693000	-2.630641000
H	1.087288000	1.696009000	-4.299989000
H	3.243554000	0.499408000	-3.826230000
H	4.588251000	-0.990011000	-2.235499000
H	4.699282000	-2.256740000	-0.102629000
H	3.550117000	-3.021565000	2.048594000
H	1.471824000	-3.009553000	3.461016000
H	-0.523590000	-1.733678000	2.689488000
H	0.139103000	4.034219000	4.468548000
H	-1.643074000	2.817339000	5.830185000
H	-3.357676000	1.026821000	5.964803000
H	1.381580000	4.238660000	2.295250000
H	0.846036000	2.650503000	0.428155000
H	-4.585432000	-0.903311000	4.852746000
H	-4.881698000	-2.344333000	2.814435000
H	-3.445423000	-1.928061000	0.806945000
O	-2.418285000	-2.556899000	-3.090310000
C	-2.067636000	-2.250418000	-2.033260000
O	-1.718425000	-1.912100000	-0.968786000
C	-0.407019000	-4.882911000	-1.745235000
C	-1.513614000	-5.156817000	-1.034608000
C	-0.279796000	-5.236738000	-3.203541000
C	0.779994000	-4.185231000	-1.135984000
H	-1.592480000	-4.884289000	0.022580000
H	-2.372219000	-5.661297000	-1.488987000
H	-0.101224000	-4.329485000	-3.806936000
H	0.589204000	-5.897510000	-3.369378000
H	-1.180804000	-5.737627000	-3.587058000
H	1.687611000	-4.808102000	-1.219181000
H	1.000234000	-3.247924000	-1.675558000
H	0.615570000	-3.950425000	-0.075464000

N	-2.318185000	1.081059000	-0.653141000
H	-2.609395000	2.037221000	-0.399080000

Int 5 (S=1)

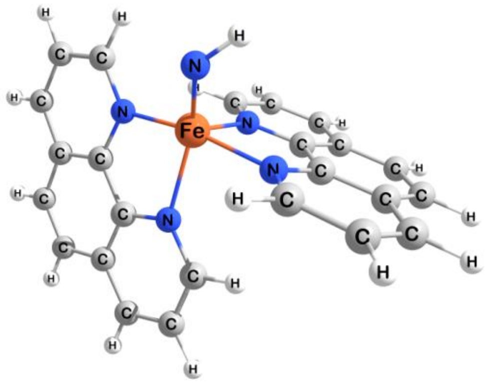

Fe	-1.139439000	0.164228000	0.155987000
N	0.238609000	-1.185698000	0.932407000
N	-0.606856000	1.616262000	1.340913000
N	0.298518000	0.482331000	-1.139378000
N	-2.341408000	-0.318629000	1.674220000
C	1.425280000	-0.240378000	-0.901177000
C	1.397902000	-1.129223000	0.217029000
C	-1.221605000	1.558421000	2.554587000
C	-2.162090000	0.507038000	2.733907000
C	-3.220293000	-1.314111000	1.754038000
C	-3.956073000	-1.542221000	2.933058000
C	-3.776066000	-0.716307000	4.031950000
C	-2.854977000	0.355706000	3.954523000
C	0.267160000	2.594801000	1.107698000
C	0.570996000	3.559671000	2.086089000
C	-0.039130000	3.506459000	3.328516000
C	-0.973344000	2.477733000	3.595700000
C	0.251467000	1.314096000	-2.180398000
C	0.179178000	-2.002253000	1.980810000
C	1.353400000	1.475430000	-3.038065000
C	2.518638000	0.763807000	-2.801396000
C	2.581578000	-0.129656000	-1.705937000
C	1.267995000	-2.805568000	2.370640000
C	2.449385000	-2.756496000	1.652277000

C	2.541911000	-1.897052000	0.531504000
C	-1.680555000	2.309147000	4.837518000
C	-2.580146000	1.291527000	5.011319000
C	3.733068000	-0.920249000	-1.366697000
C	3.713940000	-1.765634000	-0.291724000
H	-0.676108000	1.869247000	-2.329631000
H	1.272259000	2.167678000	-3.877331000
H	3.389036000	0.882667000	-3.451055000
H	4.625325000	-0.828877000	-1.989604000
H	4.591822000	-2.363665000	-0.037891000
H	3.307582000	-3.369628000	1.937019000
H	1.158878000	-3.455805000	3.239802000
H	-0.752834000	-2.038606000	2.544358000
H	0.189909000	4.246484000	4.098780000
H	-1.482121000	3.019036000	5.643368000
H	-3.110163000	1.174192000	5.958850000
H	1.294512000	4.339341000	1.843693000
H	0.750011000	2.625531000	0.131605000
H	-4.339915000	-0.879697000	4.953421000
H	-4.663922000	-2.371846000	2.960696000
H	-3.343314000	-1.945876000	0.871598000
N	-2.474912000	0.482178000	-0.684006000
H	-3.373031000	0.919276000	-0.401163000

Int 5 (S=3)

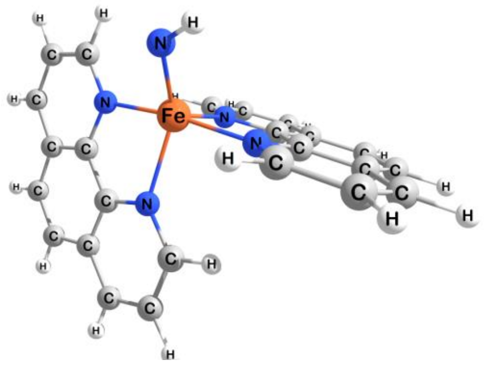

Fe	-1.185089000	0.089686000	0.183014000
N	0.252850000	-1.258633000	0.878240000

N	-0.531247000	1.611516000	1.414276000
N	0.262934000	0.426124000	-1.189340000
N	-2.392439000	-0.290219000	1.712134000
C	1.417710000	-0.238664000	-0.931651000
C	1.413296000	-1.143253000	0.175965000
C	-1.203251000	1.580978000	2.595913000
C	-2.195304000	0.562153000	2.755358000
C	-3.294274000	-1.265891000	1.811422000
C	-4.058776000	-1.440780000	2.978121000
C	-3.876237000	-0.584558000	4.051798000
C	-2.920685000	0.456025000	3.963779000
C	0.396826000	2.542121000	1.213977000
C	0.705821000	3.501818000	2.196443000
C	0.036137000	3.480965000	3.408390000
C	-0.955109000	2.496784000	3.642456000
C	0.192348000	1.262765000	-2.218593000
C	0.201042000	-2.099659000	1.908102000
C	1.305264000	1.499546000	-3.047651000
C	2.499785000	0.845118000	-2.793351000
C	2.584413000	-0.061954000	-1.709580000
C	1.311987000	-2.872123000	2.294963000
C	2.501700000	-2.758555000	1.595470000
C	2.581234000	-1.872073000	0.494662000
C	-1.708018000	2.377227000	4.861446000
C	-2.648906000	1.396633000	5.016537000
C	3.763962000	-0.805559000	-1.359750000
C	3.761521000	-1.674388000	-0.303016000
H	-0.770043000	1.751638000	-2.387904000
H	1.207954000	2.196815000	-3.881108000
H	3.376549000	1.016305000	-3.422518000
H	4.664076000	-0.661550000	-1.961200000
H	4.659992000	-2.238774000	-0.044182000
H	3.378516000	-3.344177000	1.881848000
H	1.217149000	-3.547358000	3.146526000
H	-0.740539000	-2.169097000	2.455462000
H	0.262841000	4.213688000	4.186506000

H	-1.508025000	3.090029000	5.664325000
H	-3.214407000	1.308262000	5.946468000
H	1.473681000	4.247573000	1.985690000
H	0.917588000	2.537508000	0.254732000
H	-4.460656000	-0.702135000	4.967482000
H	-4.786889000	-2.252144000	3.016332000
H	-3.416611000	-1.924133000	0.948291000
N	-2.444186000	0.294706000	-0.918341000
H	-3.436427000	0.144512000	-0.665514000

Int 5 (S_a=5)

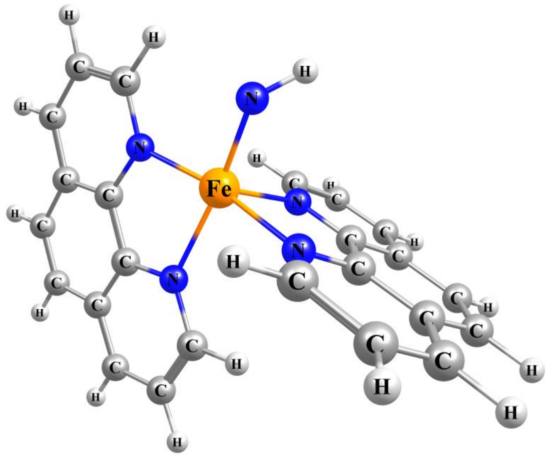

Fe	-1.061436000	0.200655000	0.171377000
N	0.403983000	-1.042563000	0.952641000
N	-0.569761000	1.707389000	1.565840000
N	0.278611000	0.308682000	-1.342096000
N	-2.382408000	-0.299760000	1.663926000
C	1.438475000	-0.353229000	-1.077371000
C	1.506771000	-1.076340000	0.153832000
C	-1.275587000	1.549797000	2.712156000
C	-2.247269000	0.492331000	2.759848000
C	-3.284894000	-1.276240000	1.653080000
C	-4.103365000	-1.532923000	2.768464000
C	-3.970605000	-0.753123000	3.904767000
C	-3.022299000	0.297539000	3.927437000
C	0.341273000	2.667914000	1.469053000
C	0.603097000	3.542722000	2.541060000
C	-0.106255000	3.402827000	3.722139000

C	-1.083254000	2.384379000	3.838020000
C	0.150929000	0.990358000	-2.478811000
C	0.423209000	-1.705040000	2.106798000
C	1.192678000	1.048448000	-3.421734000
C	2.382290000	0.384511000	-3.168510000
C	2.536088000	-0.347640000	-1.965800000
C	1.549624000	-2.436523000	2.527884000
C	2.677474000	-2.479011000	1.726071000
C	2.681031000	-1.785912000	0.491162000
C	-1.875735000	2.159377000	5.015576000
C	-2.808363000	1.160410000	5.056994000
C	3.721676000	-1.075112000	-1.602565000
C	3.790966000	-1.764060000	-0.422682000
H	-0.803008000	1.497997000	-2.639636000
H	1.047051000	1.617937000	-4.340768000
H	3.205033000	0.417644000	-3.886910000
H	4.567922000	-1.064822000	-2.292709000
H	4.694059000	-2.314930000	-0.151293000
H	3.562933000	-3.040711000	2.032986000
H	1.513580000	-2.960759000	3.483842000
H	-0.473389000	-1.664841000	2.727411000
H	0.077230000	4.070495000	4.567332000
H	-1.716941000	2.810070000	5.878265000
H	-3.410915000	0.996597000	5.953020000
H	1.360563000	4.319108000	2.423073000
H	0.880416000	2.750829000	0.521602000
H	-4.590569000	-0.937000000	4.785453000
H	-4.827804000	-2.347149000	2.719796000
H	-3.361578000	-1.878448000	0.744211000
N	-2.281423000	1.095936000	-0.631189000
H	-2.730045000	1.987002000	-0.370615000

Int 5 (S_b=5)

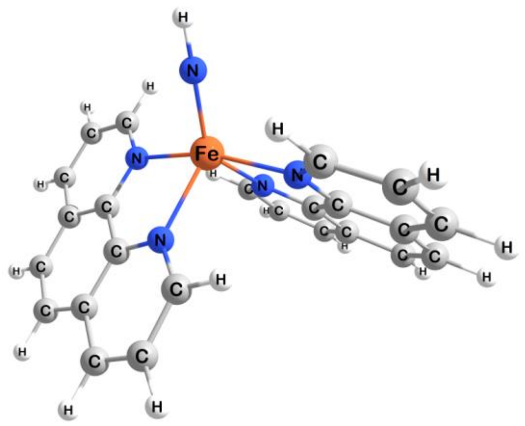

N	-2.528626000	-0.114562000	-1.081689000
Fe	-1.258663000	0.077458000	0.134651000
N	0.206988000	-1.326188000	0.722855000
N	-0.761255000	1.709535000	1.393341000
N	0.373989000	0.537802000	-1.217468000
N	-2.476881000	-0.306912000	1.881799000
C	1.489740000	-0.180103000	-0.947420000
C	1.403707000	-1.166086000	0.092969000
C	-1.291888000	1.613918000	2.643831000
C	-2.217807000	0.547286000	2.899991000
C	-3.375293000	-1.273034000	2.041739000
C	-4.048575000	-1.462290000	3.263804000
C	-3.770814000	-0.621376000	4.328705000
C	-2.834268000	0.428761000	4.167914000
C	0.089666000	2.698026000	1.121919000
C	0.462226000	3.650443000	2.086643000
C	-0.066962000	3.567850000	3.362955000
C	-0.977559000	2.530204000	3.675144000
C	0.383319000	1.426439000	-2.205422000
C	0.096238000	-2.234917000	1.690973000
C	1.540844000	1.674872000	-2.967756000
C	2.700411000	0.971135000	-2.688395000
C	2.701305000	0.002602000	-1.655413000
C	1.177159000	-3.042697000	2.084215000
C	2.399647000	-2.897287000	1.451672000
C	2.543044000	-1.938645000	0.420608000

C	-1.594268000	2.372086000	4.964273000
C	-2.489534000	1.366278000	5.200660000
C	3.844139000	-0.791908000	-1.299225000
C	3.767893000	-1.721572000	-0.300018000
H	-0.547749000	1.967527000	-2.394325000
H	1.507589000	2.419645000	-3.764369000
H	3.615067000	1.148020000	-3.259585000
H	4.775565000	-0.637285000	-1.848150000
H	4.637250000	-2.325776000	-0.031876000
H	3.255281000	-3.514278000	1.736344000
H	1.031876000	-3.772606000	2.881780000
H	-0.874450000	-2.333277000	2.178120000
H	0.205298000	4.294623000	4.132096000
H	-1.334810000	3.084893000	5.749990000
H	-2.962803000	1.258679000	6.178956000
H	1.164037000	4.438968000	1.811515000
H	0.498768000	2.744930000	0.112187000
H	-4.271294000	-0.751756000	5.291231000
H	-4.774637000	-2.271452000	3.355501000
H	-3.561603000	-1.923172000	1.182812000
H	-3.251252000	-0.199986000	-1.807658000

Int 5 (S=7)

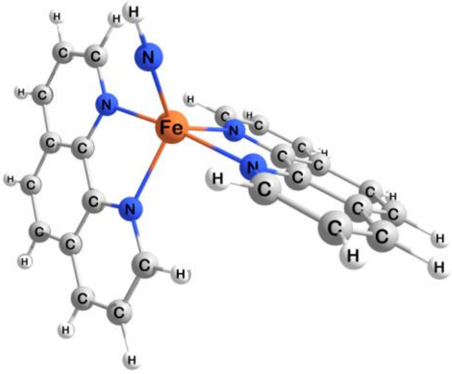

Fe	-1.187960000	0.105967000	0.195662000
N	0.296097000	-1.304035000	0.763240000
N	-0.700802000	1.713816000	1.493093000
N	0.370435000	0.491734000	-1.249955000

N	-2.489381000	-0.269971000	1.876712000
C	1.499942000	-0.216499000	-1.000533000
C	1.462090000	-1.168417000	0.075963000
C	-1.300999000	1.626940000	2.710793000
C	-2.262433000	0.577365000	2.911631000
C	-3.398162000	-1.231992000	2.004016000
C	-4.121794000	-1.420131000	3.197026000
C	-3.886719000	-0.579254000	4.271673000
C	-2.935055000	0.462667000	4.151267000
C	0.196504000	2.672437000	1.273208000
C	0.543826000	3.611275000	2.261110000
C	-0.060189000	3.541336000	3.504722000
C	-1.016399000	2.529476000	3.762801000
C	0.348979000	1.358685000	-2.258039000
C	0.227395000	-2.172540000	1.769819000
C	1.478298000	1.589663000	-3.066500000
C	2.645960000	0.889628000	-2.814412000
C	2.684007000	-0.052482000	-1.757880000
C	1.324731000	-2.967962000	2.145608000
C	2.518016000	-2.847003000	1.454545000
C	2.616224000	-1.927479000	0.382630000
C	-1.701658000	2.381328000	5.017702000
C	-2.625642000	1.391103000	5.203581000
C	3.842163000	-0.834946000	-1.424074000
C	3.809648000	-1.734067000	-0.394951000
H	-0.588088000	1.895095000	-2.429683000
H	1.416178000	2.316049000	-3.878259000
H	3.538997000	1.050135000	-3.423435000
H	4.750238000	-0.694584000	-2.014455000
H	4.691131000	-2.328282000	-0.144397000
H	3.386498000	-3.452353000	1.725202000
H	1.217002000	-3.666483000	2.976627000
H	-0.722872000	-2.246354000	2.302267000
H	0.190262000	4.257704000	4.290919000
H	-1.468218000	3.085444000	5.819322000
H	-3.147510000	1.286943000	6.157277000

H	1.284490000	4.378486000	2.030964000
H	0.661292000	2.704735000	0.285781000
H	-4.429244000	-0.704960000	5.211875000
H	-4.854272000	-2.226418000	3.258013000
H	-3.556585000	-1.880210000	1.138081000
N	-2.603993000	-0.093039000	-1.140847000
H	-3.342371000	-0.173733000	-1.853285000

Int 6

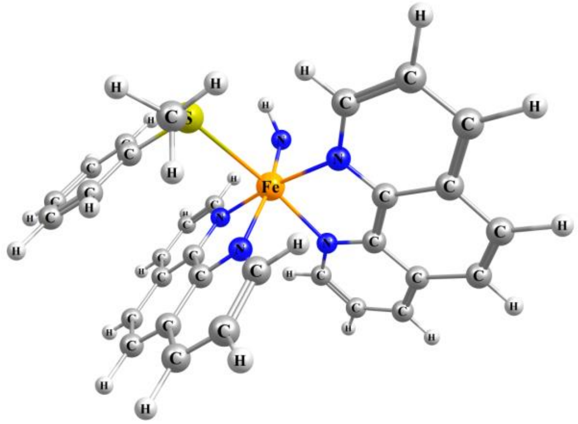

Fe	-2.417367000	0.836190000	0.816570000
N	-1.227504000	-0.884012000	0.716041000
N	-0.799905000	1.571759000	2.092348000
N	-1.430824000	1.231519000	-0.916470000
N	-3.166706000	0.381245000	2.688334000
C	-0.566963000	0.245600000	-1.277988000
C	-0.457555000	-0.884342000	-0.406341000
C	-1.033542000	1.242146000	3.385343000
C	-2.292908000	0.623352000	3.702561000
C	-4.372547000	-0.108923000	2.960235000
C	-4.761969000	-0.450124000	4.266990000
C	-3.872794000	-0.257033000	5.309717000
C	-2.600733000	0.304394000	5.048355000
C	0.344681000	2.151811000	1.758053000
C	1.339875000	2.443318000	2.711956000
C	1.120381000	2.118140000	4.039035000
C	-0.097159000	1.501346000	4.415930000

C	-1.559458000	2.313120000	-1.681071000
C	-1.133711000	-1.903404000	1.564913000
C	-0.818083000	2.471246000	-2.864115000
C	0.055036000	1.471299000	-3.259514000
C	0.204467000	0.313212000	-2.459772000
C	-0.272868000	-2.992676000	1.331904000
C	0.504754000	-3.015869000	0.186599000
C	0.429965000	-1.937841000	-0.726931000
C	-0.423918000	1.141985000	5.768033000
C	-1.627689000	0.571073000	6.071784000
C	1.082286000	-0.779221000	-2.775975000
C	1.190943000	-1.858673000	-1.943733000
H	-2.274751000	3.065740000	-1.341823000
H	-0.952832000	3.373834000	-3.461475000
H	0.630736000	1.562694000	-4.183466000
H	1.662914000	-0.725306000	-3.699168000
H	1.861849000	-2.684877000	-2.188256000
H	1.176182000	-3.852603000	-0.020204000
H	-0.236167000	-3.805423000	2.058634000
H	-1.761164000	-1.871671000	2.457808000
H	1.872452000	2.333210000	4.801938000
H	0.310765000	1.344919000	6.550354000
H	-1.877891000	0.308965000	7.102066000
H	2.266427000	2.921237000	2.390083000
H	0.490755000	2.397368000	0.703076000
H	-4.144193000	-0.519401000	6.335065000
H	-5.758313000	-0.861154000	4.435309000
H	-5.064173000	-0.232814000	2.124911000
N	-3.506817000	2.164258000	0.643950000
H	-4.459623000	2.076245000	0.255666000
C	-3.428375000	-0.965210000	-2.096230000
C	-3.589195000	0.074069000	-3.025203000
C	-2.654954000	-2.089585000	-2.416188000
C	-2.964236000	-0.011567000	-4.269952000
C	-2.045221000	-2.167851000	-3.671851000
C	-2.192383000	-1.131908000	-4.597862000

H	-4.192187000	0.948724000	-2.769322000
H	-2.509440000	-2.898420000	-1.700990000
H	-3.081742000	0.804698000	-4.986403000
H	-1.439084000	-3.042954000	-3.916372000
H	-1.704083000	-1.194673000	-5.572932000
S	-4.214866000	-0.742477000	-0.509275000
C	-4.247673000	-2.421931000	0.181137000
H	-4.801526000	-3.070664000	-0.510597000
H	-3.239784000	-2.816533000	0.353252000
H	-4.784918000	-2.355203000	1.135276000

$TS_{6\rightarrow7}$

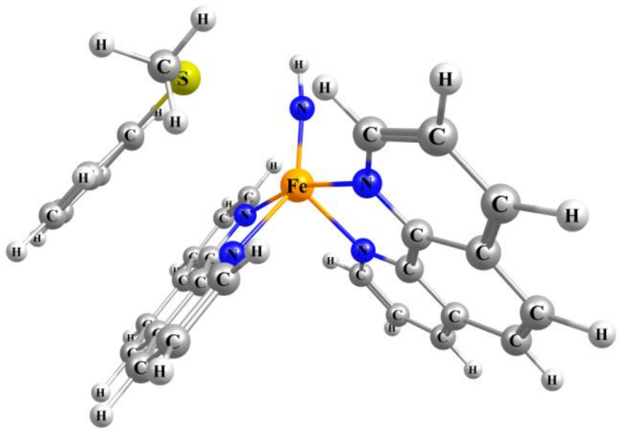

Fe	-2.529937000	0.859074000	0.909846000
N	-1.460396000	-1.002431000	0.580144000
N	-0.983770000	1.750053000	2.105765000
N	-1.408913000	1.258306000	-0.890763000
N	-3.029560000	0.174851000	2.925426000
C	-0.530864000	0.281909000	-1.217620000
C	-0.555477000	-0.921413000	-0.431826000
C	-0.998223000	1.341615000	3.403408000
C	-2.091630000	0.515753000	3.841776000
C	-4.093043000	-0.524857000	3.304430000
C	-4.262946000	-0.969411000	4.629362000
C	-3.297413000	-0.663044000	5.572625000
C	-2.171664000	0.108514000	5.196414000
C	0.005702000	2.532299000	1.681831000
C	1.046128000	2.954544000	2.528883000

C	1.049046000	2.548289000	3.851265000
C	0.004802000	1.721482000	4.328805000
C	-1.476117000	2.361096000	-1.628042000
C	-1.528169000	-2.122733000	1.296074000
C	-0.624840000	2.570624000	-2.728853000
C	0.302063000	1.597387000	-3.061090000
C	0.367014000	0.402991000	-2.304631000
C	-0.698686000	-3.230191000	1.043577000
C	0.236368000	-3.157754000	0.025956000
C	0.330698000	-1.978718000	-0.749638000
C	-0.085553000	1.269662000	5.690351000
C	-1.132803000	0.498413000	6.108872000
C	1.267515000	-0.678171000	-2.591163000
C	1.250609000	-1.820833000	-1.842455000
H	-2.222632000	3.102994000	-1.333377000
H	-0.708342000	3.493642000	-3.304304000
H	0.976576000	1.732245000	-3.909941000
H	1.957792000	-0.569638000	-3.430442000
H	1.927648000	-2.646810000	-2.070543000
H	0.898461000	-3.999439000	-0.191270000
H	-0.803504000	-4.125659000	1.657778000
H	-2.261473000	-2.156835000	2.103131000
H	1.843058000	2.859871000	4.534269000
H	0.701721000	1.567623000	6.386270000
H	-1.202771000	0.166749000	7.147185000
H	1.832519000	3.596204000	2.128891000
H	-0.014845000	2.842238000	0.635920000
H	-3.395931000	-0.999677000	6.607552000
H	-5.149227000	-1.549221000	4.890912000
H	-4.835655000	-0.749899000	2.536509000
N	-4.044138000	1.653609000	0.508831000
H	-4.958083000	1.995580000	0.200995000
C	-3.569009000	-0.849966000	-2.332422000
C	-3.247659000	0.055883000	-3.359483000
C	-2.935893000	-2.099955000	-2.271537000
C	-2.296606000	-0.292816000	-4.316628000

C	-1.989305000	-2.438580000	-3.242662000
C	-1.664488000	-1.541309000	-4.262911000
H	-3.738554000	1.031258000	-3.399832000
H	-3.161638000	-2.807669000	-1.474283000
H	-2.043738000	0.418101000	-5.106368000
H	-1.492724000	-3.409835000	-3.186450000
H	-0.913491000	-1.808857000	-5.009202000
S	-4.784527000	-0.301270000	-1.162122000
C	-5.153667000	-1.777282000	-0.175683000
H	-5.541977000	-2.568605000	-0.832089000
H	-4.273544000	-2.135415000	0.374193000
H	-5.934170000	-1.466734000	0.531712000

Int 7

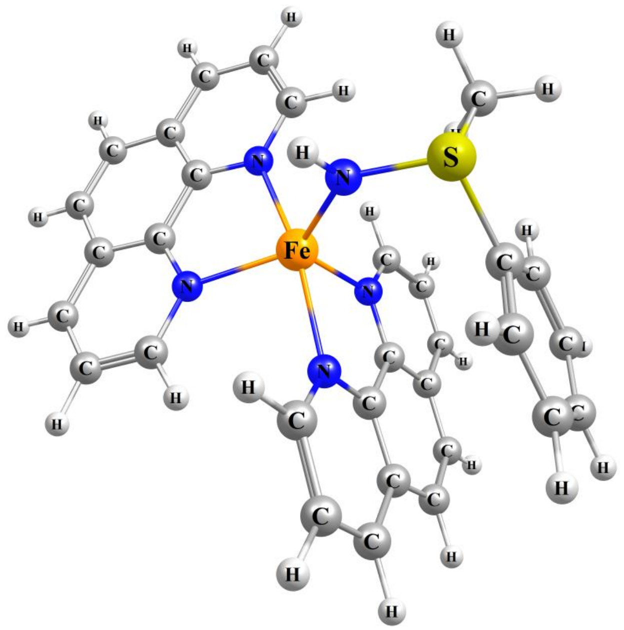

Fe	-2.229156000	0.527130000	0.925949000
N	-1.175195000	-1.278147000	0.445747000
N	-0.883473000	1.696562000	2.187631000
N	-1.093994000	1.093560000	-0.868126000
N	-2.820670000	-0.041245000	2.971859000
C	-0.296725000	0.096863000	-1.317306000
C	-0.345691000	-1.166890000	-0.624209000
C	-1.041885000	1.470600000	3.518328000
C	-2.073859000	0.552634000	3.935057000

C	-3.795435000	-0.868281000	3.331173000
C	-4.079956000	-1.169643000	4.676923000
C	-3.317048000	-0.585153000	5.671918000
C	-2.278239000	0.307853000	5.317598000
C	0.062077000	2.540912000	1.786275000
C	0.906739000	3.219668000	2.683924000
C	0.757171000	3.003804000	4.041647000
C	-0.238167000	2.108047000	4.498154000
C	-1.120763000	2.250591000	-1.517608000
C	-1.274853000	-2.445604000	1.072953000
C	-0.321964000	2.496795000	-2.650480000
C	0.517786000	1.497213000	-3.110490000
C	0.543160000	0.246969000	-2.449193000
C	-0.548331000	-3.581994000	0.670792000
C	0.313366000	-3.482906000	-0.407687000
C	0.437598000	-2.250575000	-1.091866000
C	-0.462652000	1.830170000	5.890042000
C	-1.443766000	0.966033000	6.284331000
C	1.343862000	-0.863567000	-2.883560000
C	1.290157000	-2.063906000	-2.233574000
H	-1.796253000	3.017715000	-1.128094000
H	-0.374998000	3.466359000	-3.148038000
H	1.150882000	1.653592000	-3.987238000
H	1.985782000	-0.729402000	-3.757036000
H	1.888568000	-2.911199000	-2.575594000
H	0.896545000	-4.344216000	-0.742665000
H	-0.670736000	-4.518927000	1.216329000
H	-1.951083000	-2.491680000	1.929572000
H	1.395644000	3.513759000	4.767353000
H	0.170790000	2.331393000	6.625533000
H	-1.615262000	0.759409000	7.343161000
H	1.664462000	3.902021000	2.295653000
H	0.168795000	2.692107000	0.711362000
H	-3.505686000	-0.799239000	6.726744000
H	-4.892139000	-1.858912000	4.913421000
H	-4.381185000	-1.319229000	2.528058000

N	-4.073283000	0.810426000	0.167579000
H	-4.522650000	1.726629000	0.230977000
C	-3.711723000	-0.653652000	-2.155680000
C	-3.336044000	0.285410000	-3.125722000
C	-3.186574000	-1.948525000	-2.161231000
C	-2.417120000	-0.079237000	-4.108782000
C	-2.268029000	-2.302169000	-3.154826000
C	-1.883578000	-1.373614000	-4.124676000
H	-3.753215000	1.295132000	-3.104176000
H	-3.465035000	-2.683540000	-1.407330000
H	-2.114472000	0.650467000	-4.862437000
H	-1.845183000	-3.308823000	-3.158045000
H	-1.159628000	-1.655632000	-4.891973000
S	-4.905487000	-0.058236000	-0.942963000
C	-5.392030000	-1.584243000	-0.105477000
H	-5.870163000	-2.246180000	-0.840630000
H	-4.520805000	-2.061471000	0.360808000
H	-6.116842000	-1.268371000	0.655839000

Int 8

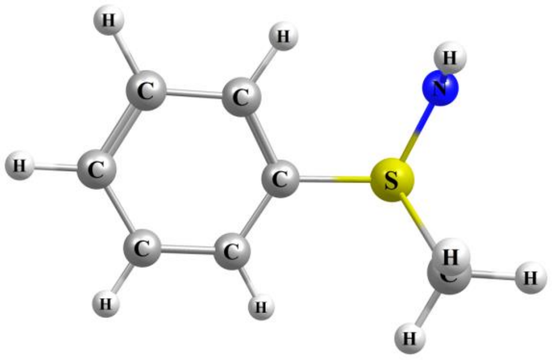

N	-5.125407000	1.329165000	-0.720361000
H	-5.760952000	1.193430000	-1.520488000
C	-3.478104000	-0.632956000	-1.911329000
C	-3.382720000	0.246218000	-2.989444000
C	-2.837894000	-1.877758000	-1.934806000
C	-2.660237000	-0.135154000	-4.125838000
C	-2.119371000	-2.251791000	-3.074219000
C	-2.031991000	-1.383807000	-4.170265000
H	-3.869779000	1.222315000	-2.922837000

H	-2.894344000	-2.554836000	-1.078488000
H	-2.586777000	0.547621000	-4.976372000
H	-1.622835000	-3.225099000	-3.104609000
H	-1.467550000	-1.681281000	-5.057628000
S	-4.379071000	-0.052248000	-0.420784000
C	-5.608340000	-1.415607000	-0.344886000
H	-6.163635000	-1.439384000	-1.293506000
H	-5.099598000	-2.371364000	-0.157714000
H	-6.273039000	-1.163669000	0.492749000

Int 9

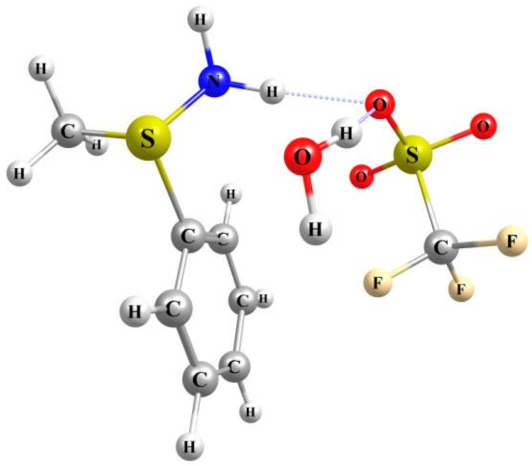

N	-5.307464000	0.560018000	0.372218000
H	-4.546360000	1.255305000	0.505814000
C	-3.601320000	-0.738987000	-1.272547000
C	-3.942752000	0.208741000	-2.237936000
C	-2.478855000	-1.563841000	-1.396274000
C	-3.129967000	0.325250000	-3.367249000
C	-1.684262000	-1.440670000	-2.537747000
C	-2.009901000	-0.497917000	-3.519778000
H	-4.804199000	0.860443000	-2.099565000
H	-2.229406000	-2.284610000	-0.614108000
H	-3.370752000	1.074641000	-4.124362000
H	-0.804129000	-2.076974000	-2.654486000
H	-1.379540000	-0.398806000	-4.406795000
S	-4.611071000	-0.987713000	0.182609000
C	-6.043158000	-1.840752000	-0.531096000

H	-6.426153000	-1.252709000	-1.374569000
H	-5.690681000	-2.833186000	-0.844896000
H	-6.788112000	-1.935118000	0.271773000
C	-1.234222000	2.856875000	-0.837179000
S	-2.940203000	3.482548000	-0.390638000
F	-1.254407000	1.541234000	-1.093060000
F	-0.778156000	3.486427000	-1.921084000
F	-0.384418000	3.068603000	0.171011000
O	-2.699247000	4.876347000	0.018449000
O	-3.325725000	2.578702000	0.745037000
O	-3.736272000	3.286062000	-1.615888000
H	-5.854730000	0.528955000	1.240037000
O	-2.245557000	0.138542000	1.408939000
H	-2.487473000	1.080055000	1.305289000
H	-1.546539000	0.023923000	0.749061000

Product

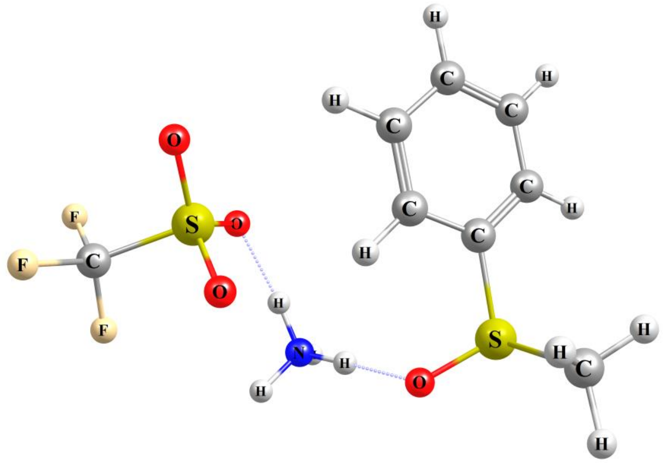

N	-3.977419000	-0.283701000	0.100257000
H	-3.288498000	-1.089329000	0.136576000
C	-1.771500000	-2.542880000	2.795989000
C	-0.887967000	-2.357989000	3.864251000
C	-3.144852000	-2.682581000	2.990304000
C	-1.402255000	-2.340444000	5.164056000
C	-3.649157000	-2.646892000	4.294456000
C	-2.779847000	-2.481399000	5.378287000
H	0.183597000	-2.230362000	3.687345000

H	-3.807958000	-2.801024000	2.132375000
H	-0.725810000	-2.203830000	6.011586000
H	-4.726964000	-2.732645000	4.450019000
H	-3.176850000	-2.452873000	6.396236000
S	-1.133084000	-2.526325000	1.099958000
C	-0.614203000	-4.265252000	0.991278000
H	0.194084000	-4.436071000	1.716420000
H	-1.491191000	-4.891400000	1.206056000
H	-0.255580000	-4.417454000	-0.036222000
C	-7.985980000	-0.960371000	1.203568000
S	-6.503626000	-1.651797000	2.104172000
F	-7.614579000	-0.476415000	0.012615000
F	-8.899745000	-1.914498000	1.005988000
F	-8.551715000	0.028318000	1.900470000
O	-7.068732000	-2.222215000	3.341785000
O	-5.653552000	-0.440444000	2.314588000
O	-5.945894000	-2.618838000	1.130555000
H	-4.553634000	-0.264132000	0.975676000
O	-2.369437000	-2.459166000	0.178426000
H	-4.610908000	-0.417017000	-0.694086000
H	-3.481340000	0.604433000	-0.018471000

References

[1] P. F. Kuijpers, J. I. van der Vlugt, S. Schneider, B. de Bruin, *Chem. Eur. J.* **2017**, *23*, 13819-13829.
[2] N. Guimond, S. I. Gorelsky, K. Fagnou, *J. Am. Chem. Soc.* **2011**, *133*, 6449-6457.
[3] S. Makai, E. Falk, B. Morandi, *Org. Synth.* **2020**, *97*, 207-216.
[4] M. R. Couto, J. L. Rodrigues, L. R. Rodrigues, *J. R. Soc. Interface.* **2017**, *14*, 20170470.
[5] J. Uenishi, T. Tanaka, K. Nishiwaki, S. Wakabayashi, S. Oae, H. Tsukube, *J. Org. Chem.* **1993**, *58*, 4382-4388.
[6] G. Li, Y. Nieves-Quinones, H. Zhang, Q. Liang, S. Su, Q. Liu, M. C. Kozlowski, T. Jia, *Nat. Commun.* **2020**, *11*, 2890.
[7] X. Li, Y. Wang, L. Yang, Z. Zhang, X. Xie, *Tetrahedron* **2022**, *110*, 132708.
[8] K. F. Morgan, I. A. Hollingsworth, J. A. Bull, *Chem. Commun.* **2014**, *50*, 5203-5205.
[9] A. E. Shiely, C. N. Slattery, A. Ford, K. S. Eccles, S. E. Lawrence, A. R. Maguire, *Org. Biomol. Chem.* **2017**, *15*, 2609-2628.
[10] J. Polster, P. Schieberle, *J. Agric. Food Chem.* **2015**, *63*, 1419-1432.
[11] S. Chatterjee, S. Makai, B. Morandi, *Angew Chem. Int. Ed.* **2021**, *60*, 758-765.
[12] A. Bismuto, T. Delcaillau, P. Müller, B. Morandi, *ACS Catalysis* **2020**, *10*, 4630-4639.
[13] H. Marom, S. Antonov, Y. Popowski, M. Gozin, *J. Org. Chem.* **2011**, *76*, 5240-5246.

[14] Y. Uetake, T. Niwa, T. Hosoya, *Org. Lett.* **2016**, *18*, 2758-2761.
[15] K. F. Morgan, I. A. Hollingsworth, J. A. Bull, *Org. Biomol. Chem.* **2015**, *13*, 5265-5272.
[16] D. Xie, Y. Wang, X. Zhang, Z. Fu, D. Niu, *Angew Chem. Int. Ed.* **2022**, *61*, e202204922.
[17] P. Pinacho, D. Loru, T. Šumanovac, M. Šekutor, M. Schnell, *ChemPhysChem* **2023**, *24*, e202300561.
[18] F. Neese, *Wiley Interdiscip. Rev. Comput. Mol. Sci.* **2012**, *2*, 73-78.
[19] F. Neese, *Wiley Interdiscip. Rev. Comput. Mol. Sci.* **2022**, *12*, e1606.
[20] A. D. Becke, *J. Chem. Phys.* **1993**, *98*, 5648-5652.
[21] C. Lee, W. Yang, R. G. Parr, *Phys. Rev. B* **1988**, *37*, 785-789.
[22] P. J. Stephens, F. J. Devlin, C. F. Chabalowski, M. J. Frisch, *J. Chem. Phys.* **1994**, *98*, 11623-11627.
[23] S. H. Vosko, L. Wilk, M. Nusair, *Can. J. Phys.* **1980**, *58*, 1200-1211.
[24] S. Grimme, J. Antony, S. Ehrlich, H. Krieg, *J. Chem. Phys.* **2010**, *132*, 154104.
[25] S. Grimme, S. Ehrlich, L. Goerigk, *J. Comput. Chem.* **2011**, *32*, 1456-1465.
[26] F. Weigend, R. Ahlrichs, *Phys. Chem. Chem. Phys.* **2005**, *7*, 3297-3305.
[27] F. Neese, F. Wennmohs, A. Hansen, U. Becker, *Chem. Phys.* **2009**, *356*, 98-109.
[28] V. Barone, M. Cossi, *J. Phys. Chem. A* **1998**, *102*, 1995-2001.
